# Transcriptional profiling reveals functional links between RasGrf1 and Pttg1 in pancreatic beta cells

**DOI:** 10.1186/1471-2164-15-1019

**Published:** 2014-11-25

**Authors:** Lara Manyes, Monica Arribas, Carmela Gomez, Nuria Calzada, Alberto Fernandez-Medarde, Eugenio Santos

**Affiliations:** Centro de Investigación del Cáncer, IBMCC (CSIC-USAL), University of Salamanca, Campus Unamuno, 37007 Salamanca, Spain

**Keywords:** Ras, ERK, RasGrf1, Beta cells, Pttg1, Pancreatic islets, Transcriptomics, Transcriptional factors

## Abstract

**Background:**

Our prior characterization of RasGrf1 deficient mice uncovered significant defects in pancreatic islet count and size as well as beta cell development and signaling function, raising question about the mechanisms linking RasGrf1 to the generation of those “pancreatic” phenotypes.

**Results:**

Here, we compared the transcriptional profile of highly purified pancreatic islets from RasGrf1 KO mice to that of WT control animals using commercial oligonucleotide microarrays. RasGrf1 elimination resulted in differential gene expression of numerous components of MAPK- and Calcium-signaling pathways, suggesting a relevant contribution of this GEF to modulation of cellular signaling in the cell lineages integrating the pancreatic islets. Whereas the overall transcriptional profile of pancreatic islets was highly specific in comparison to other organs of the same KO mice, a significant specific repression of Pttg1 was a common transcriptional alteration shared with other tissues of neuroectodermal origin. This observation, together with the remarkable pancreatic phenotypic similarities between RasGrf1 KO and Pttg1 KO mice suggested the possibility of proximal functional regulatory links between RasGrf1 and Pttg1 in pancreatic cell lineages expressing these proteins.

Analysis of the mPttg1 promoter region identified specific recognition sites for numerous transcription factors which were also found to be differentially expressed in RasGrf1 KO pancreatic islets and are known to be relevant for Ras-ERK signaling as well as beta cell function. Reporter luciferase assays in BT3 insulinoma cells demonstrated the ability of RasGrf1 to modulate mPttg1 promoter activity through ERK-mediated signals. Analysis of the phenotypic interplay between RasGrf1 and Pttg1 in double knockout RasGrf1/Pttg1 mice showed that combined elimination of the two loci resulted in dramatically reduced values of islet and beta cell count and glucose homeostasis function which neared those measured in single Pttg1 KO mice and were significantly lower than those observed in individual RasGrf1 KO mice.

**Conclusions:**

The specific transcriptional profile and signaling behavior of RasgGrf1 KO pancreatic islets, together with the dominance of Pttg1 over RasGrf1 with regards to the generation of these phenotypes in mouse pancreas, suggest that RasGrf1 is an important upstream component of signal transduction pathways regulating Pttg1 expression and controlling beta cell development and physiological responses.

**Electronic supplementary material:**

The online version of this article (doi:10.1186/1471-2164-15-1019) contains supplementary material, which is available to authorized users.

## Background

RasGrf1 is a mammalian guanine nucleotide exchange factor (GEF) for Ras GTPases [1–3] which is specifically expressed at central nervous system locations, testis and pancreas (CNS) [4, 5], although various isoforms thereof are also expressed in a variety of other tissues [6]. RasGrf1 specifically promotes GDP/GTP exchange on various members of the Ras GTPase family including the three canonical Ras isoforms (H, N and K), M-Ras, R-Ras, [7, 8], and Rac [9]. The RasGrf1 proteins act by coupling different upstream stimuli to activation of various intracellular pathways through the specific contribution of some of its structural domains, including the CDC25-H domain capable of activating Ras family members, the Ca^+2^/Calmodulin interacting IQ domain [5, 10], two PH domains, or a DH (Dbl homology) domain capable of activating Rho/Rac family GTPases [10]. Due to this complex domain structure, RasGrf1 is able to couple Ras activation to different signal transduction processes, such as those mediated by Ca^2+^/calmodulin [1, 11], heterotrimeric G proteins [12], cAMP activity [13], CDC42 [14], or even non-receptor tyrosine kinases [15].

RasGrf1 KO mice exhibit defects in memory consolidation [3], reduced body size, deficiency of IGF-1 and growth hormone, and hypoinsulinemia [16, 17]. Our analysis of the RasGrf1 KO mice showed significant reduction of pancreatic islet number and size, linked to diminished beta cell proliferation and neogenesis, and resulting in hypoinsulinemia, glucose intolerance and prediabetic state [17]. RasGrf1 elimination is also associated to defective eye phenotypes including impaired retinal photoreception [18] and altered lens growth [19].

Multiple reports suggest the participation of Ras signaling pathways, and RasGrf1 in particular, in the control of pancreatic islet and beta cell development and function. For example, male transgenic mice overexpressing human H-Ras develop β-cell degeneration and onset of diabetes, exhibiting hyperglycemia, glycosuria, and hypoinsulinemia [20], and Ras inhibition in mouse cells leads to increased insulin sensitivity and glucose uptake [21]. It has also been shown in human islet cells that glucose and glucagon-like peptide-1 activate the Rap/B-Raf signaling module, thus mediating ERK and phosphoinositide 3-kinase activation [22]. Additional reports have also demonstrated the participation of Ras signaling in the response of beta cells to prolactin [23] and the contribution of HNF-4a, RasGrf1 and other putative RasGEFs, such as ST5, as modulators of Ras/ERK activity in those cells [24]. Furthermore, RasGrf1 has also been implicated in insulin secretion induced by the transient diabetes mellitus gene Zac1 [25]. Finally, our observations in small-bodied RasGrf1 KO mice of hypoinsulinemia, reduced pancreatic beta cell mass, and defective activation of Ras signaling after stimulation of isolated islet cells with IGF1, are also strongly indicative of an essential role of RasGrf1 in beta cell proliferation and neogenesis [17, 26]. In this regard, the very strong similarity observable between the pancreatic phenotypes of our RasGrf1 KO mice and those exhibited by Cdk4 KO mice [27, 28], p70S6K KO mice [29, 30] or Pttg1KO mice [31] is very striking, suggesting the possible joint participation of RasGrf1 and those different signaling molecules in the same Ras-dependent signaling pathways [1].

In order to better understand the role of Ras signaling in pancreatic islet and beta cell function and to characterize the transcriptional changes underlying the phenotypic pancreatic alterations observed in our RasGrf1 KO mice, we aimed at characterizing the genomic expression profiles linked to the disappearance of RasGrf1 from pancreatic β cells in our KO mice. For this purpose, we used commercial oligonucleotide microarrays in order to carry out a detailed comparison between the genomic expression profiles of highly purified preparations of pancreatic islets isolated from our Wild Type (WT) and Knockout (KO) RasGrf1 mice [17, 26, 32]. The data generated here supports the notion of significant functional roles of RasGrf1 in control of pancreatic islet signaling processes, as the absence of this RasGEF was clearly linked to multiple transcriptomic alterations affecting genes whose products are known to be highly relevant for cellular signaling pathways of pancreatic beta cells involving Ras/ERK activation and intracellular calcium movement. In addition, our data also suggests that the functional role of RasGrf1 in pancreatic islets is mechanistically mediated, at least in part, by Pttg1, a known regulator of mitosis and gene expression.

## Results and discussion

### Differential gene expression in RasGrf1 KO pancreatic islets

**Figure 1:**
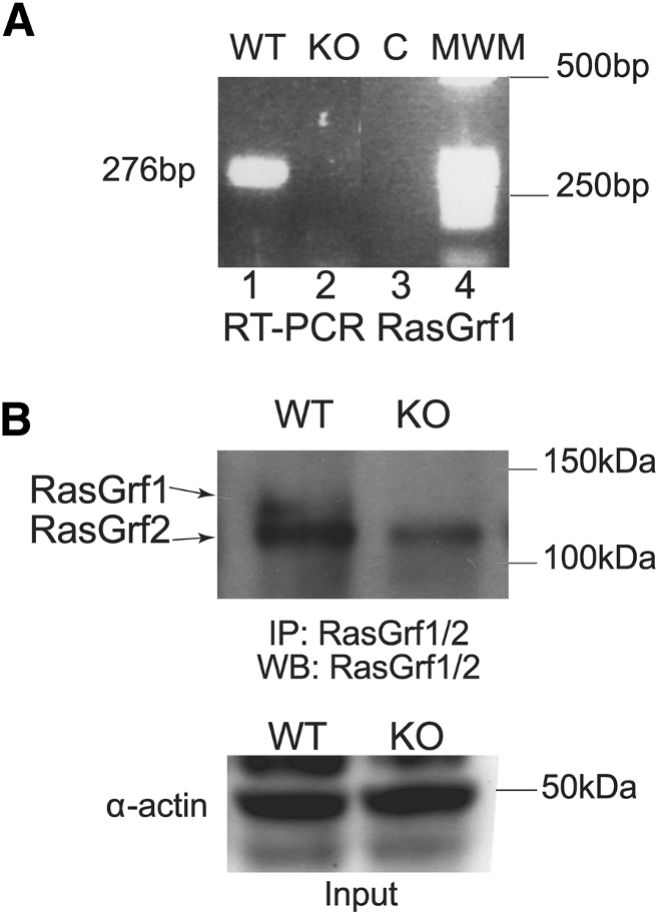
**RasGrf1 expression in pancreatic islets. (A)** RT-PCR amplification of the RasGrf1 locus. Total RNA samples from pancreatic islet preparations isolated from RasGrf1 knockout (KO) or wild type (WT) mice were tested by means of RT-PCR assays using oligonucleotides LM5 and LM85 (described in Methods) designed to amplify a specific 276 bp fragment corresponding to the catalytic domain of RasGrf1. C: negative control. MWM: molecular weight markers. **(B)** Analysis of RasGrf1/2 protein expression. Total protein extracts from WT and RasGrf1 KO pancreatic islets were immunoprecipitated with anti-RasGrf1/2 antibody and later submitted to SDS-PAGE followed by Western immunoblot with the same antibody (see Methods). The amount of protein in each lane was controlled and normalized by means of Western immunoblots using specific alpha-actin antibodies (lower panel).

For a full characterization of the transcriptional profiles of purified islets from the pancreas of Wild Type and RasGrf1 KO mice, we first determined RasGrf1 mRNA and protein expression levels in isolated mouse islets (Figure 1). Thus, RT-PCR analysis of purified RNA from wild type and RasGrf1 KO pancreatic islets showed that RasGrf1 mRNA is strongly expressed in wild type islets and, as expected, its expression was totally lost in the KO islets (Figure 1A). Furthermore, a similar RasGrf1 expression pattern was observed at the protein level, as Western blot analysis of islet protein immunoprecipitates allowed detection of the full size 140 kDa RasGrf1 protein band in wild type islets, which was completely absent in the RasGrf1 KO samples. Interestingly, RasGrf2 expression was also observed in pancreatic islets and appeared to be slightly diminished upon RasGrf1 elimination (Figure 1B). Smaller-sized RasGrf1 protein forms that have been described elsewhere [33, 34] were not detected by this technique in our mice islets.

**Figure 2:**
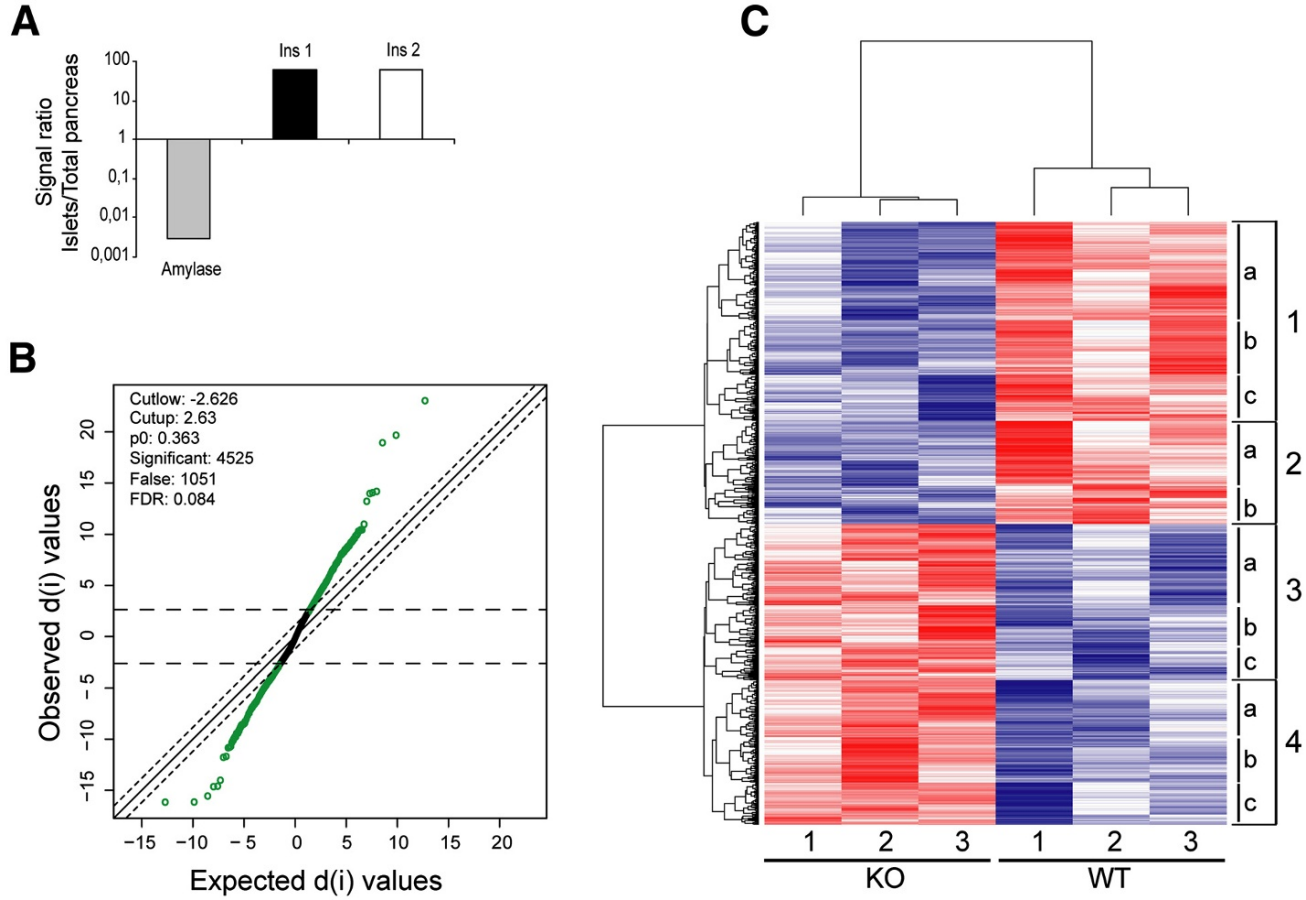
**Transcriptional analysis of WT and RasGrf1 KO pancreatic islets by microarrays. (A)** Quality analysis of islet RNA used in the microarrays. Real-time PCR analysis comparing Ct values of equal cDNA amounts from islet or total pancreas RNA. Bars represent the islet/total pancreas ratios of signals produced by specific oligonucleotides for amylase (grey bar), insulin 1 (black bar) or insulin 2 (clear bar), normalized to beta-2-microglobulin (unchanged in all conditions). Ratios are presented in a log scale. **(B)** Graphical display of statistical analysis performed in islet RNA from RasGrf1 KO microarray data compared to WT. The plot compares observed values against expected values in SAM-contrasting analyses of six independent microarray hybridizations. Distance of a spot from the no-change diagonal means differential expression for a probeset and it is measured by the d-value. Quantitative cutoff values and FDR parameters defined by the horizontal and diagonal dashed lines are specified in the upper left corner. **(C)** Hierarchical clustering of the 4525 probesets showing differential expression in RasGrf1 KO islets (listed in Table S1, FDR 0.084; threshold p-value: 0.046). Dendrogram was generated by cluster analysis of absolute expression values of probesets. Each row represents single probeset expression. Columns identify results from single microarray hybridizations. Each box represents the hybridization signal value of a probeset in the corresponding microarray assay. The intensity of color in each box provides a quantitative estimation of its expression change. Blue: repressed probesets. Red: overexpressed probesets. Black: unchanged expression. Most significant KEGG pathways and associated p-values for horizontal clusters: Clusters 1a and 1b: MAPK signaling (7.92E-4 and 2.3E-3 respectively) and Calcium signaling (1.9E-3 and 2.7E-3, respectively). Cluster 1c: cytokine-cytokine interaction genes (p = 7.2E-4). Cluster 3: metabolic processes, including riboflavin metabolism and Kreb’s cycle (Cluster 3a, p = 6.7E-4 and 1.6E-3) or pyruvate metabolism (Cluster 3b, p = 7E-3).

Ras signaling pathways have been shown to be important contributors to islet cell physiology in processes of regulation of cellular proliferation [17, 24] and response to prolactin [23]. To gain further insight into the molecular events occurring in islets modulated by RasGrf1, we compared the genomic expression profiles of Wild Type and RasGrf1 KO islets. Our preliminary studies indicated that minimal contamination of exocrine cells in our islet preparations would significantly bias data resulting from the microarray hybridizations and therefore we assessed an additional quantitative method to control the purity of islets samples as previously described [35]. Thus, before use in microarray hybridizations, the specificity and purity of the RNA preparations from islet cells used in our microarray hybridizations were tested by measuring the normalized signal ratios obtained in Real-time PCR analyses of the total RNA sample preparations with specific primer probes for insulin (specifically expressed in islet beta cells) and amylase (expressed throughout the pancreas), which indicated an enrichment of about 20000 fold for our islet RNA preparations in comparison to RNA extracted from total pancreases (Figure 2A).

Six independent microarray hybridizations were performed using total RNA preps extracted from pancreatic islets of WT and RasGrf1 KO mice. Global expression data were analyzed using RMA [36] as a tool allowing simultaneous background correction, multichip normalization and quantization of probeset expression signals in all separate microarray hybridizations. Statistically significant gene expression changes occurring in RasGrf1 KO islets were identified using the SAM algorithm [37]. In order to facilitate comparison of these data with our previous microarray analyses of other tissues of RasGrf1 KO mice [18, 32] and to get a manageable list of genes for further functional analysis, we set the false discovery rate (FDR) [38] value at 0.084, which allowed identification of a total of 4525 differentially expressed gene probesets (corresponding to 3686 distinct genes) in the RasGrf1 KO islets (Figure 2B). A dendrogram generated by hierarchical clustering of all microarray hybridization data sets generated with all our WT and RasGrf1 KO samples (Figure 2C) showed clear discrimination between the WT and the KO islet RNAs as well as a generally opposite pattern of differential mRNA expression between these two genotypes. In addition, functional annotation analysis identified significant enrichment of genes related to relevant biological functions in the horizontal clusters defined by the dendrogram (Figure 2C). These observations indicate that the elimination of RasGrf1 in pancreatic islets produces significant transcriptional changes in a wide set of genes and suggesting a critical role for this GEF in the regulation of specific pancreatic endocrine functions.

A detailed description of all the induced or repressed probesets detected in pancreatic islets is provided in Table S1 (Additional file 1), where the R-fold changes and statistical significance parameters relative to each of the 4525 differentially expressed probesets are provided. Overall, about half of differentially expressed loci (2268 probesets, corresponding to 1953 distinct genes) were downregulated, whereas the other half (2256 probesets, corresponding to 1799 distinct genes) were up-regulated in the mRNA population of the RasGrf1 KO islets as compared to their WT control counterparts (Additional file 1: Table S1, Figure 2C).

In view of the neuroendocrine nature of the hormone-making islet cells [39] it was remarkable that the two genes showing highest levels of transcriptional repression (by d-value and R-fold) in our RasGrf1 KO islet samples (Additional file 1: Table S1) are *Gdpd3* (glycerophosphodiester phosphodiesterase domain containing 3 protein), a locus functionally important in neural development and differentiation, and *Iapp* (Islet Amyloid Polypeptide or amylin), which is highly relevant for Beta cell functionality. Of note, IAPP KO mice show increased insulin release and glucose elimination responses, a behavior completely opposed to that exhibited by our RasGrf1KO mice [40], suggesting that *Iapp* downregulation in pancreatic islets may be a compensatory mechanism triggered by the absence of RasGrf1 in our KO mice. Interestingly, the *Gpdp3* gene is also found strongly repressed in the retina of RasGrf1 KO mice [18], suggesting the occurrence of common regulatory mechanisms for regulation of *Gdpd3* expression by RasGrf1 in different cellular lineages or environments.

It should be noted that one of the 3 probesets (1435614_x_at) designed by Affymetrix to recognize the RasGrf1 locus in the MOE 430A commercial microarrays used in this study produced a surprising result, yielding significantly higher signals (about 4-fold) when hybridized to RNA from the RasGrf1 KO islets than after hybridization to their WT counterparts (Additional file 1: Table S1, Additional file 2: Figure S1 panel A). Using RT-PCR assays and specific primers we found out that this apparent contradiction is accounted for by the fact that the specific genomic sequence recognized by this probeset is localized within the 3-UTR untranslated region of the RasGrf1 locus, a region that is not expressed in the WT samples but appears overexpressed in our KO mRNA samples, possibly as a result of neomycin-cassette-dependent RNA polymerase activity associated to the specific construct vector used to generate our KO mouse strain (Additional file 2: Figure S1, panels B, C) [17]. Consistent with this, the LM5/LM85 pair of primers, designed to recognize the catalytic domain of RasGrf1 (Additional file 2: Figure S1 panel C; see Methods) produced significant amplification of a specific 276 bp band in the WT samples but not in the RasGrf1 KO samples (Additional file 2: Figure S1, panel B). In contrast, primers MA1F/MA2R, designed to hybridize exclusively at the very end of the RasGrf1 3′ UTR region (Additional file 2: Figure S1, panel C) yielded a 140 bp amplification product only in KO islet samples, but not in the WT samples (Additional file 2: Figure S1, panel B). On the other hand, the combination of primers MA5F/MA2R, designed to amplify a region corresponding to the last two coding exons of RasGrf1 located downstream of the coding sequence recognized by probeset 1422600_at and upstream of the non-coding sequences recognized by probeset 1435614_x_at and primer MA2R (Additional file 2: Figure S1, panel C), amplified a 334 bp DNA fragment only in the WT samples, but not in the KO samples, showing that the mechanisms driving overexpression of the 3′UTR in our KO samples did not involve expression of the neighboring, coding RasGrf1 region.

### Functional annotation and mechanistic implications of the transcriptional signature of RasGrf1 KO pancreatic islets. Alteration of components of Ras/MAPK and Calcium signaling pathways

In order to try and unveil potentially relevant mechanistic and functional clues related to the specific transcriptional profile of RasGrf1 KO islet cells, we used functional annotation clustering software searching for co-occurrent annotations pinpointing specific subgroups within the list of differentially expressed genes identified in Table S1 (Additional file 1). Thus, separate analysis of the subsets of induced and repressed loci listed in that Table using the DAVID software package [41], identified a variety of GO Biological Processes (Additional file 3: Table S2A and Additional file 4: Table S2B) and KEGG Signaling Pathways (Additional file 5: Table S3A and Additional file 6: Tables S3B) that could be ascribed with high statistical significance (p-values < 0.05, ranging from E-17 to E-2) to particular subgroups of repressed (Additional file 3: Table S2A and Additional file 5: Table S3A) or induced (Additional file 4: Table S2B and Additional file 6: Table S3B) genes of the RasGrf1 KO islets.

**Figure 3:**
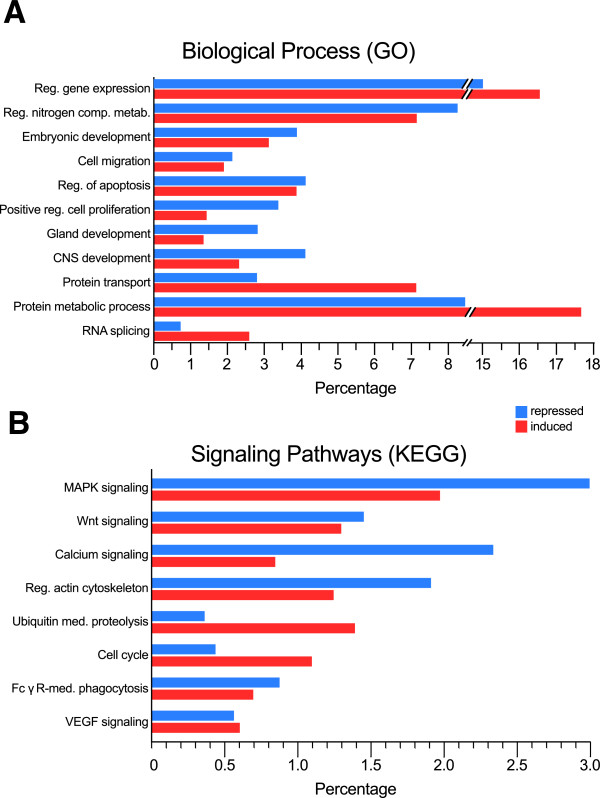
**Functional annotation analysis of differentially expressed genes in pancreatic islets of RasGrf1 KO mice.** The DAVID functional annotation tool (http://david.abcc.ncifcrf.gov/) was used to identify statistically significant functional associations linking particular gene subsets contained within the list of repressed (blue) or induced (red) loci occurring in RasGrf1 KO pancreatic islets (Additional file 1: Table S1, FDR = 0.084) to specific Biological Process categories (Gene Ontology (GO) database, panel **A**) or Signaling Pathways (KEGG pathway database, panel **B**). Red: Induction. Blue: repression. Percentage values were calculated by referring the number of genes in each individual subset to the total number of repressed (1777) or induced (1943) genes recognized by DAVID within the list of differentially expressed genes listed in Table S1 (Additional file 1). The complete functional annotation analyses are described in Tables S2A,B (search against Biological Process GO database) (Additional files 3 and 4) and Tables S3A,B (search against KEGG signaling pathways database) (Additional files 5 and 6).

Figure 3 summarizes data from Tables S2A,B (Additional files 3 and 4) and S3A,B (Additional files 5 and 6) by quantitating the percentage distribution of the most abundant subgroups of induced or repressed genes that were functionally ascribable to various GO BP categories (Figure 3A) or KEGG pathways (Figure 3B) identified by DAVID. As shown, the different GO BP Terms involving highest numbers of differentially expressed genes in the RasGrf1 KO pancreatic islets (Figure 3A) include a series of functional categories which are likely to be relevant for the functionality, development and/or proliferation of the specialized cell lineages composing the pancreatic islets. Some of these categories (transcriptional regulation, apoptosis) appeared to be more or less equally affected by differential gene induction or repression (Figure 3A). In contrast, other functions, such as cell proliferation or gland development, involved significant higher numbers of downregulated genes than up-regulated genes in the transcriptome of the RasGrf1 KO islets (Figure 3A). Conversely, functional processes related to protein transport, RNA splicing or protein metabolism implied significantly higher numbers of up-regulated genes than down-regulated genes in the transcriptome of the KO islets (Figure 3A). The pattern of prevalent downregulation of genes related to cell proliferation and development appears to mirror the consistent defects of beta cell development and proliferation previously described in this RasGrf1 KO mice strain [17]. All in all, these transcriptional alterations are consistent with a significant functional role of RasGrf1 in control of development and homeostasis of the specific cell lineages present in pancreatic islets.

This notion is further supported by the DAVID-based predictions of alterations in cellular (KEGG) signaling pathways significant for islet functionality that are potentially caused by the differential gene expression occurring in the RasGrf1 KO islets (Figure 3B). In particular, multiple components of the cross-talking MAPK signaling pathways and Calcium signaling pathways were very significantly affected by differential gene expression, particularly by gene repression (p-values 1.0E-06 and 2,7E-06) in the RasGrf1 KO islets (Figure 3B, Additional file 5: Table S3A). In addition, the Hedgehog (p-value 7, 5E-04) and WNT (p-value 9,4E-04) signaling pathways are also predicted to be significantly downregulated in our RasGrf1 KO islets (Figure 3B, Additional file 5: Table S3A) and it is possible that the predominant downregulation of the above mentioned pathways may also be related to the repression of regulatory components of the actin cytoskeleton also observed (p-value 1,2E-02) in our RasGrf1 KO islets (Figure 3B, Additional file 5: Table S3A). In contrast, the components of signaling pathways relevant for Ubiquitin mediated proteolysis (p-value 5.2E-06) and Cell cycle regulation (p-value 1.3E-03) are predicted to be more significantly affected by up-regulation of gene expression in the RasGrf1 KO islets (Figure 3B, Additional file 6: Table S3B). Interestingly most of the above mentioned cellular signaling pathways are known to be susceptible of regulation by different Ras-dependent signals and, specifically, RasGrf1 is a well-known mediator connecting Calcium signaling and the Ras-MAPK pathway [1, 11].

Focusing on individual repressed components of Ras/MAPK signaling pathways it was striking to observe the downregulation of multiple tyrosine kinase receptors including EGFR, PDGFRA and B, and FGFR2 and 3, as well as many of their specific ligands (including FGF2, FGF3, FGF4, FGF17, FGF18, FGF21, FGF22, FGF23) (Additional file 5: Table S3A). Of note in this regard, different studies have reported a positive paracrine/autocrine effect of FGF to promote beta cell proliferation and insulin secretion in pancreatic islets [42], with FGF21 in particular significantly improving pancreatic cell function and beta cell survival through activation of MAPK- and AKT- mediated signals [43]. It is therefore likely that this downregulation of the FGF ligands and receptors underlies the diminished number and smaller size of pancreatic islets observed in the pancreas of RasGrf1 KO mice [17]. Regarding MAPK signaling, it was also striking to observe the downregulation of several GEFs, such as RasGrf2, Sos1, RasGRP1 and RasGRP2, known for their ability to activate various Ras family members in different cellular contexts. While the absence of Ras-GEF activity may also be related to the defective functionality of beta cells in pancreatic islets of RasGrf1 KO mice, these observations also confirm the absence of compensatory transcriptional activation of other GEFs associated to the disappearance of RasGrf1 in those cells [17]. On the other hand, the possibility of mechanisms aimed at compensating at other levels the defective Ras-mediated signaling of RasGrf1 KO islet cells, might be consistent with the observed overexpression of different GTPase family members including Kras, Rac1, Gna1 and multiple Rab family members, as well as potential regulators and effectors thereof.

Calcium metabolism is crucial for proper islet metabolism [44], and the changes of expression in several important components in the regulation of calcium metabolism in RasGrf1 KO Langerhans islets suggest a link with the metabolic alterations previously described by our laboratory [17]. In this regard, it was also particularly striking the observed repression of up to 8 different calcium channels, including CACNG8, CACNG7, CACNG6, CACNG2, CACNB1, CACNA1S, CACNA2D2, CACNA1F in RasGrf1 KO islets (Additional file 5: Table S3A).

Finally, it was also very noticeable the dysregulation of the expression of multiple upstream components of the cascade of kinases involved in Ras/MAPK signaling pathways (including several isoforms of protein kinase C isoforms, as well as several MAPK cascade isoforms (reduced expression of Mapk13, Map2k2, Map2k7, Map3k7, Map3k8 and Map4k1, as well as overexpression of Mapk1, Mapk8, Mapp2k1, Map3k3, Map3k4 and Map4k3) (Additional file 1: Table S1, Additional file 5: Table S3A and Additional file 6: Table S3B). As an important number of the mitogen activated protein kinases have been related to JNK-dependent pathways [45], it is possible that this dysregulation may be related to cellular stress caused by the absence of RasGrf1 to the beta cells of our RasGrf1 KO mice.

The expression of various phosphatases participating in Ras/MAPK signaling pathways was also found dysregulated in the RasGrf1 KO islets (Additional file 5: Table S3A and Additional file 6: Table S3B). Thus, whereas most differentially expressed Ptpn isoforms were down-regulated, about half of the differentially expressed members of the Dusp family (dual specificity phosphatases acting on phosphorylated MAPK forms) were down-regulated, and the other half were up-regulated in RasGrf1 KO islets (Additional file 1: Table S1, Additional file 5: Table S3A and Additional file 6: Table S3B). All differentially expressed isoforms of the Ppm magnesium-dependent phosphatases were overexpressed in KO islets. On the other hand, whereas the calcineurin isoform Ppp3cc was downregulated, the isoforms Ppp3r1 and Ppp3ca were overexpressed in the RasGrf1 deficient islets (Additional file 1: Table S1, Additional file 5: Table S3A and Additional file 6: Table S3B). Calcineurin activity is likely to be relevant for pancreatic islet functionality as it has been reported to dephosphorylate and control localization and activity of NFAT transcription factors in pancreatic beta cells [46].

The transcriptional profile of phospholipase downstream effectors of Ras/MAPK signaling is also found significantly altered in the RasGrf1 KO islets (Additional file 1: Table S1, Additional file 5: Table S3A and Additional file 6: Table S3B). Interestingly, PLA2G6 has been reported to protect beta cells from apoptosis via the generation of arachidonic acid and prostaglandin E2 [47] and the repair of mitochondrial membrane peroxidation [48] as well as modulating oscillations and transients of [Ca^+2^]_i_ in response to ATP in these cells [49]. Accordingly, overexpression of PLA2G6 in the islets of our RasGrf1 KO mice would be consistent with our previous phenotypic characterization of reduced proliferation and unchanged apoptotic rates in beta cells of those animals [17].

Finally, several transcription factors known to act in Ras/MAPK signaling pathways were also significantly affected by differential gene expression in the RasGrf1 KO islets. In particular, ATF2, DDIT3, ETS1 and JUND [50–53] were up-regulated, whereas Elk1, RORA, USF, NFATC2 and NFATC4 [46, 52, 54–56] were significantly repressed (Additional file 1: Table S1, Additional file 5: Table S3A and Additional file 6: Table S3B). As NFAT/calcineurin signaling interactions (with calcineurin activating cytoplasmic NFAT by dephosphorylation) are very important for regulation of pancreatic beta-cell growth and function [46], the observed differential expression of several NFAT and calcineurin isoforms also points to significant alteration of related Ras/MAPK signals in our Ras defective KO islets.

In summary, all these functional implications and mechanistic predictions derived from analysis of the transcriptional patterns in pancreatic islets of RasGrf1 KO animals are indicative of alterations of Ras/MAPK signaling pathways and Calcium signaling, and are consistent with our previous characterization of pancreatic beta cell signaling phenotypes and preliminary, direct measurements of reduced ERK responses and Ca^++^ movements in isolated islets preparations of these KO mice [17].

### Inferred regulatory mechanisms responsible for differential gene expression in RasGrf1 KO islets. Potential role of Pttg1

Most genes showing differential expression in the RasGrf1 KO islets (Additional file 1: Table S1) appeared to undergo specific transcriptional modulation of their expression at this precise location by RasGrf1, since a comparison of the microarray-based transcriptional profile of RasGrf1 KO islets (Additional file 1: Table S1) with those previously described at other tissue locations of the same RasGrf1 KO mice (including retina, LCM-purified hippocampus pyramidal cells, and brain olfactory bulb, cerebral cortex or full hippocampus) [18, 32] documented the occurrence of specific transcriptional patterns at each of those tissue locations. Interestingly however, a limited number of transcripts showed consistently common patterns of differential expression (induction or repression) at two or more of those different tissue locations in our RasGrf1 KO mice (Additional file 7: Table S4). The fact that all those tissues share a common neuroectodermal origin suggests the possibility that the members of this gene group are parallely regulated loci playing a preferential, primary driver role in the mechanisms controlling the establishment of the overall mRNA expression profile associated with the absence of RasGrf1 expression in those tissues.

**Figure 4:**
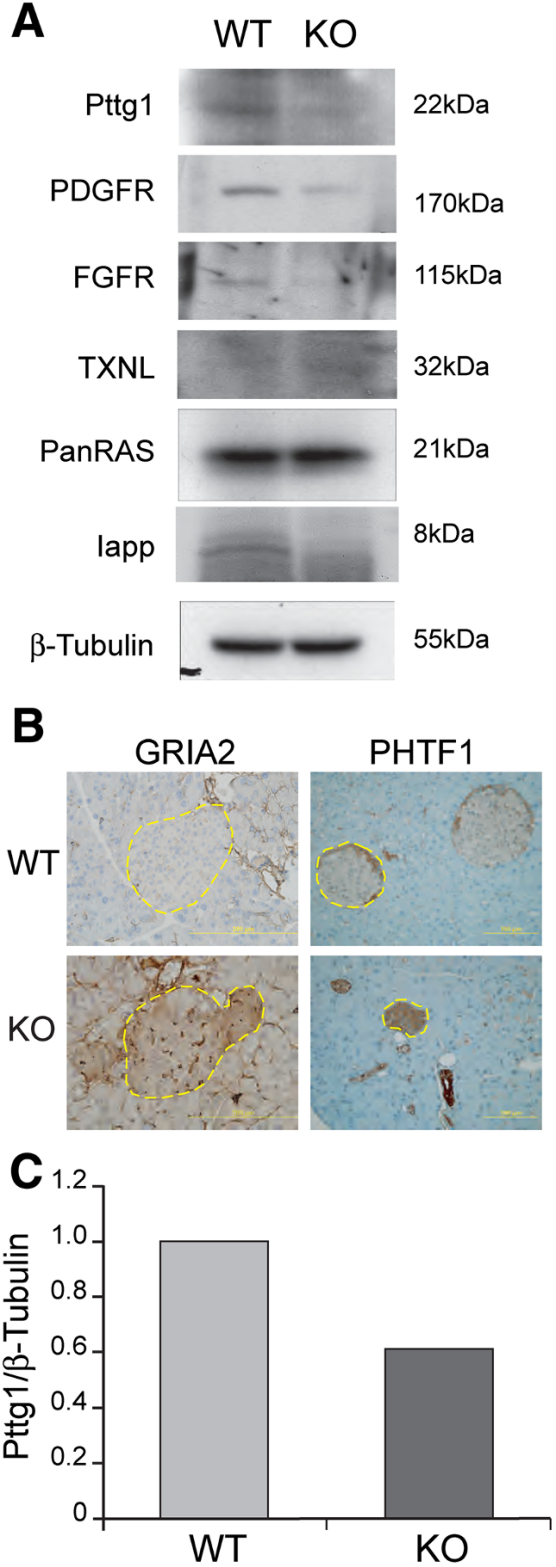
**Protein expression changes in pancreatic islets of RasGrf1 KO mice.** Western immunoblot and Immunohystochemistry assays confirming and validating several transcriptional alterations previously detected by means of microarray analysis in pancreatic islets from RasGrf1 KO mice. **(A)** Representative ECL exposures of WB assays performed in protein extracts of WT and KO pancreatic islets, using specific antibodies against IAPP, PDFR, FGFR, TXNL or PTTG1. Unchanged Ras and beta-tubulin signals are shown as internal loading controls. **(B)** Representative immunohystochemical staining of WT and KO pancreatic sections showing increased expression levels of the GRIA2 and PHTF1 proteins in pancreatic islets of RasGrf1 KO mice (islets outlined in yellow). **(C)** Normalized PTTG1 to Beta-tubulin signal ratios in Western blots of WT and KO islets quantitated using the image-J software program. For comparison purposes, this ratio was set to 1 in WT samples.

Remarkably, of all loci listed in Table S4 (Additional file 7), only Pttg1 showed the same pattern of transcriptional alteration (significant repression) in all different tissues analyzed in our RasGrf1 KO mice (Additional file 7: Table S4) [18, 32]. The repression of the Pttg1 locus at the transcriptional level was also confirmed at the level of protein expression by means of WB assays (Figure 4) which also confirmed and validated the patterns of microarray-based differential expression identified in pancreatic islets for several other randomly selected, repressed or induced loci listed in Table S1 (Additional file 1). The consistent pattern of Pttg1 repression observed in the variety of tissues mentioned above, suggests the existence of common, shared RasGrf1-sensitive mechanisms of transcriptional regulation of the Pttg1 locus in all those neuroectodermal tissue locations. The remarkable phenotypic similarities exhibited by Pttg1 KO mice and our RasGrf1 KO mice are also consistent with this notion, and support the possibility of proximal functional or regulatory interactions between RasGrf1 and Pttg1 [17, 31]. Although not all their phenotypes are exactly identical (qualitatively or quantitatively), the strong phenotypic similarities observed in both KO mice models (including lower body weight, hypoinsulinemia and altered responses to glucose and insulin responses) strongly suggest the possibility of interconnected regulatory mechanisms controlling expression of RasGrf1 and Pttg1 and/or functional proximity between these two proteins in signaling pathways of the pancreatic islets.

On the other hand, knockout mice for *Iapp*, another highly repressed locus in pancreatic islets (Additional file 1: Table S1), show increased insulin and glucose responses [40], a behavior completely opposed to that exhibited by our RasGrf1 KO mice [17, 40], suggesting the possibility that *Iapp* down-regulation might be a compensatory mechanism triggered by the absence of RasGrf1 in pancreatic islets and pointing to the possible existence of a wider, RasGrf1-dependent regulatory network involving transcriptional regulatory interactions modulating more genes than just Pttg1. This may be the case for the *Crb1* (Crumbs homolog 1) locus in view of the observation of significant transcriptional repression of this locus in the retina [18] of RasGrf1 KO mice and its simultaneous overexpression in the islets of the same animals (Additional file 7: Table S4) and the previous report in an animal model of Retinitis Pigmentosa, where a point mutation in *CRB1* that causes retinal degeneration, is linked to a strong reduction in the expression of *Pttg1*[57]. The existence of a wider network of transcriptional regulation dependent upon the expression of RasGrf1 in various neouroectodermal cellular lineages or environments, is also consistent with the transcriptional behavior of two other loci – *Aldh7a1* and *Gdpbp3* – which are found simultaneously repressed in retinas and islets (Additional file 1: Table S1 and Additional file 7: Table S4), [18] of our RasGrf1 KO mice.

Based on the highly consistent repression of *Pttg1* detected in all neouroectodermal tissues analyzed in our RasGrf1 KO mice (although other genes may display quantitatively higher levels of repression in pancreatic islets), in order to gain further mechanistic insights underlying the transcriptional observations mentioned above, we decided to carry out more-in-depth analyses of molecular or functional interactions between RasGrf1 and Pttg1 using (i) specific cell lines expressing these signaling molecules or (ii) KO animals devoid of their expression, either singly or in a combination.

To start our search for potential mechanisms behind the regulation of *Pttg1* expression by RasGrf1, we initially used TFSEARCH (http://www.cbrc.jp/research/db/TFSEARCH.html; [58] looking for Transcription Factors with consensus recognition sequences locatable within the mouse *Pttg1* promoter region (Table 1). Furthermore, we also applied GeneCodis gene set enrichment analysis [59] software to identify TFs potentially responsible for the patterns of repressed (Additional file 8: Table S5A) or induced (Additional file 9: Tables S5B) genes identified by means of microarrays (Additional file 1: Table S1) in our RasGrf1 KO islets.

**Table 1:** **Transcription factors with recognition binding sites located in the mPttg1 promoter**

**AML-1a**	E2F (E2F5*, E2F6*)	**HSF2**	***RORa***
**Brn-2**	**E4BP4**	**Ik-2**	**S8**
CdxA	***Elk-1***	**Ik-3**	***Sox-5***
**C/EBP**	**Evi-1**	**Lyf-1**	**Sp1**
**C/EBPb**	**GATA-1**	MEF-2 (MEF2a*, ***MEF2b*** , MEF2c* and ***MEF2d*** **)**	***SRY***
c-Ets (ETS1* and ETS2)	**GATA-2**	**MZF1**	**TATA**
**COUP-T**	**GATA-3**	Nkx-2 (NKX2-2*, ***NKX2-3*** **,** ***NKX2-4*** **,** ***NKX2-5*** **,** ***NKX2-6*** **)**	***USF***
**CP2**	**HFH-1**	**NRF-2**	v-Myb
**CRE-BP**	**HNF-3b**	**Oct-1**	**YY1**
deltaE	HNF-4 (HNF4a* and ***HNF4g*** **)**	**Pbx-1**	

Analysis of the relationship of transcription factors capable of binding to the Pttg1 promoter with their differential gene expression in RasGrf1 KO pancreatic islets. List of 39 transcription factors (TF) with potential binding sites locatable in the promoter region of the mPttg1 locus as determined by TFSEARCH (http://www.cbrc.jp/research/db/TFSEARCH.html) analysis of a 2,3 kbp region located 5′upstream of the transcription initiation site. TFs coincident with those identified by GeneCodis analysis as potentially responsible for the transcriptional profile of repressed (Additional file 8: Table S5A) or induced (Additional file 9: Table S5B) genes in the transcriptome of RasGrf1KO islets (Additional file 1: Table S1) are marked in bold. TFs whose expression is altered in the transcriptome of RasGrf1 KO islets (Additional file 1: Table S1) are marked with asterisks (overexpressed) or shown in italics (repressed).

Interestingly, comparison of Table 1 and S5 (Additional file 8) uncovered very significant coincidences, as many TF from Table 1 were also identified by GeneCodis as potentially responsible for differential expression of repressed or induced subsets of the transcriptome of KO islets (Additional file 8: Table S5A and Additional file 9: Table S5B). Specifically, GeneCodis analysis of the pool of downregulated loci in pancreatic islets of RasGrf1 KO mice identified several distinct groups of repressed genes (Additional file 8: Table S5A) that are known targets for transcriptional regulation by TF such as E12, LEF1, MAZ, SP1, NFAT and AP4 at exceptionally high levels of statistical significance (Additional file 8: Table S5A). In addition, several other subsets of repressed loci were also identified as specific targets for the TATA, FOXO4, PAX4, ETS2 or AP1 transcription factors at high levels of significance (p-values <1E-30 in all cases; Additional file 8: Table S5A). Similar GeneCodis analysis also identified, at very high levels of statistical significance (P-values <1E-24 in all cases), a number of distinct subsets of induced, overexpressed genes of the transcriptome of RasGrf1 KO islets that are recognized transcriptional targets of TF such as SP1, LEF1, MAZ, ELK1, E4F1, GABP, FOXO4, YY1, MYC, NFY or NFAT (Additional file 9: Table S5B). Furthermore, consistent with a suggested pattern of positive and negative transcriptional regulation, the actual mRNA levels quantitated with microarrays (Additional file 1: Table S1) for several of the transcription factors listed in Table 1 were indeed significantly amplified (ETS1, HNF4a, MEF2a, NKX2-2) or reduced (HNF4g, MEF2b, RORalp, SRY) in the transcriptome of pancreatic islets devoid of RasGrf1 protein (respective R-fold and D-values in Additional file 1: Table S1).

Of note, whereas many TFs with sequence recognition sites recognizable in the Pttg1 promoter region (Table 1) are known to be functionally linked to Ras-MAPK signaling pathways (Brn2, Cebp, COUP-t, CRE-BP, Elk1,ETS, E2F, E4BP4,GATA-1, -2 & -3, HNF3b, MEF-2, Nkx-2, NRF2, Oct1, RORA, Sp1, USF and YY1) [55, 60–68]. In addition, most of them (C/EBP, E2F, E4BP4, Elk-1, ETS, GATA-3, HNF3B(=FOXA2), HNF4, NKX2, NRF2, Oct1, Sp1, USF and YY1) have also been described in the literature to play, significant functional roles in pancreatic and beta cell physiology [27, 53, 69–79]. Thus, a potential mechanistic link between Pttg1 and the altered, differential gene expression linked to Ras-MAPK signaling in RasGrf1 KO islets is suggested by the observation that several of the TF related to Ras-MAPK signaling and listed in Table 1 because of the presence of sequence recognition sites in the promoter region of Pttg1 (i.e., ETS1, E2F5, E2F6), are also differentially expressed in the RasGrf1 KO islets (Additional file 1: Table S1). Likewise, a similar mechanistic link may be suggested for Pttg1 and differential gene expression in pancreatic islets of RasGrf1 KO mice, as several of the main transcription factors and regulatory gene networks and interactions involved in pancreatic islet development and Beta cell function [46, 74, 80] present sequence recognition sites in the promoter region of Pttg1 (i.e., Hnf3b (=FoxA2), Hnf4a and NKx2-2, Table 1) and/or are also differentially expressed in the RasGrf1 KO islets (Additional file 1: Table S1). It is highly striking to observe that most of these TF and regulatory genes, which are likely to be functionally relevant for Ras-MAPK signaling and/or beta cell function, are overexpressed (Additional file 1: Table S1). This observation suggests the interesting hypothesis that their overexpression (in particular, Hnf3b (=FoxA2), Hnf4a and NKx2-2) may represent a compensatory mechanism elicited in the developing pancreas in order to counterbalance the reduced number/size of the islets, and the hypoinsulinemia and reduced proliferation of beta cells exhibited by the RasGrf1 KO mice [17].

In any event, to uncover potential functional interactions between RasGrf1 and Pttg1 and in order to ascertain the involvement in those processes of Ras-ERK or other signaling pathways dependent upon RasGrf1, we aimed to study by means of reporter constructs the effect of RasGrf1 overexpression on Pttg1 promoter.

### RasGrf1 modulation of Pttg1 promoter activity through cytosolic kinase-mediated signaling

**Figure 5:**
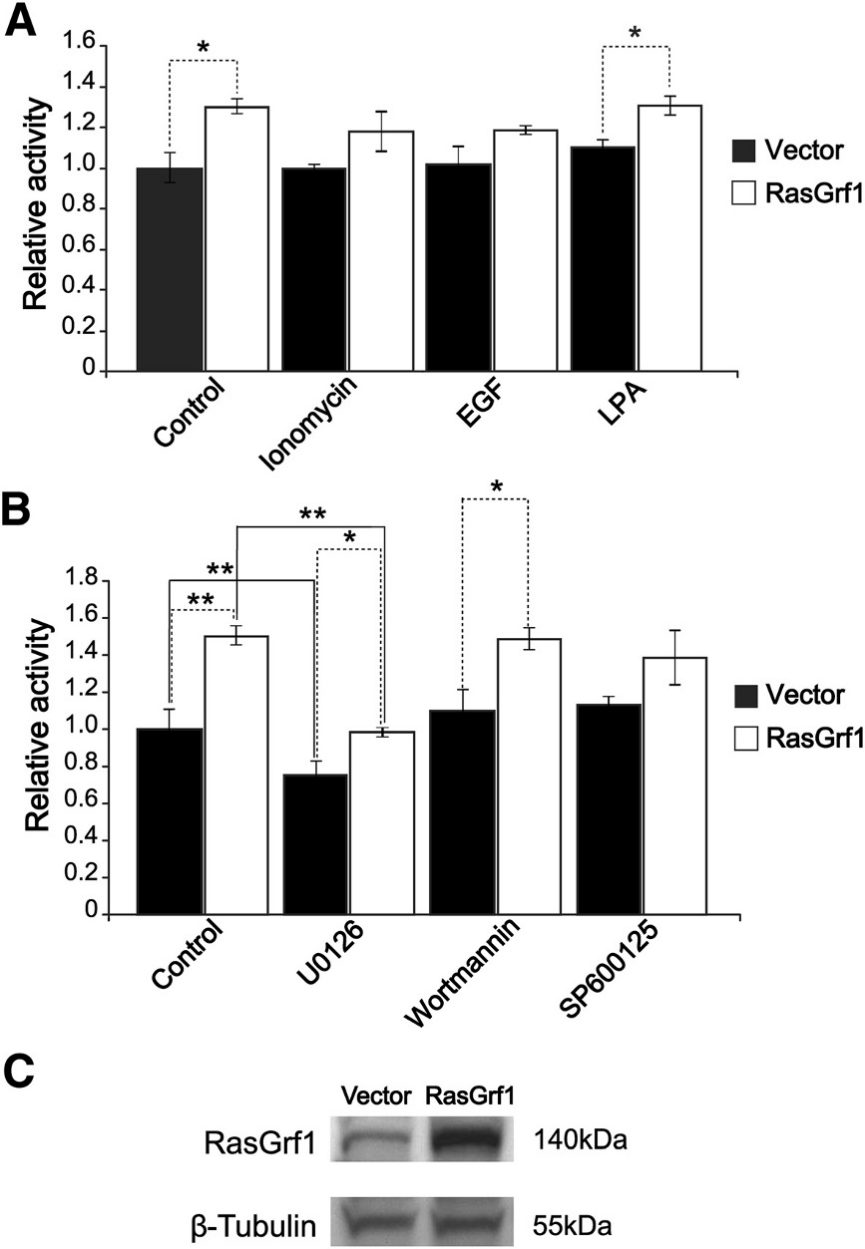
**Effect of RasGrf1 on mPttg1 promoter activity.** Luciferase reporter assays carried out in insulinoma BTC3 cells cotransfected with plasmid pGL3 (containing a 2,3 Kb promoter region of mPttg1) plus one additional construct, corresponding either to empty plasmid vector pBKCMV (black boxes) or to construct pBKCMV-RasGrf1, expressing a full length RasGrf1 cDNA clone (white boxes). **(A, B)** Assays were performed 48 hours after cotransfection and included treatment for 7 hours with the indicated agonists (panel **A**) or inhibitors (panel **B**) of signaling pathways, under conditions detailed in Methods. **(C)** Western immunoblot showing level of overexpression of the RasGrf1 full length protein in BT3 cells after 48 h transfection with pBKCMV-RasGrf1. Error bars indicate coefficient of variation. * p < 0.05; ** p < 0.01; n = 3 for Vector and RasGrf1 samples. Dotted lines: comparison between RasGrf1 overexpression and transfection with the control vector. Solid black lines: comparison between treated and non treated cells.

In order to ascertain whether or not RasGrf1 exerts any type of direct or indirect control over Pttg1 expression, we carried out luciferase assays analyzing the effect of different RasGrf1 expression levels as well as various agonist and inhibitors of RasGrf1-dependent signaling pathways in BTC3 cells transfected with a reporter construct containing a 2.3 Kb fragment of the mPttg1 5′untranslated region [81] (Figure 5). We chose this cell line as particularly appropriate for this kind of studies as it is known to express RasGrf1 in basal conditions and is also derived from an insulinoma [82], thus providing a cellular environment that is physiologically similar to the pancreatic beta cells of our animals.

We observed that RasGrf1 overexpression achieved through transfection of a RasGrf1 construct in these cells (Figure 5C) resulted in significant increase (about 30%) of the mPttg1 promoter activity both under basal, unstimulated conditions or after stimulation with various agonists known for their ability to activate RasGrf1-dependent signaling, (particularly ionomycin and LPA) [1] (Figure 5A). Interestingly, no significant changes could be detected when similar reporter assays were performed in other cell lines of different embryological origin which did not express RasGrf1 under basal conditions, such Cos1 or 293 T (data not shown), supporting the notion that the detected expression relationship between RasGrf1 and Pttg1 suggested by our microarray studies (Additional file 1: Table S1 and Additional file 7: Table S4) may be linked to this particular cellular environment of pancreatic cells.

We also tested the effect of inhibitors of various cytosolic kinases in our reporter assays of transcriptional activity dependent upon the mPttg1 promoter region in BTC3 cells (Figure 5B). Interestingly, of all inhibitors used, only the MEK1/2 inhibitor U0126 significantly reduced (about 50%) the transcriptional activity of the Pttg1 promoter when compared to untreated controls, whereas no significant changes were detected in the assays involving PI3K (wortmannin) or JNK (SP600125) inhibitors (Figure 5B). It was apparent that the MEK1/2 inhibitor did not block completely the Pttg1 promoter activity and therefore MEK activity cannot be the sole intermediary accounting for the relation between RasGrf1 and the Pttg1 promoter. However, we also observed that the reduction of Pttg1 promoter activity caused by MEK1/2 blockade was significantly higher (almost double) in RasGrf1-overexpressing cells than in the cells transfected with vector alone (Figure 5B) and that MEK2 expression was significantly repressed in RasGrf1 KO islets (about 40%, see Additional file 1: Table S1). These observations suggest that, although other pathways may be involved, there is at least a partial contribution of RasGrf1-ERK signaling to the control of Pttg1 promoter activity in these cells and are consistent with a previous report associating partial repression of PTTG1/Securin gene expression to the inhibition of the Ras/Raf/ERK pathway in HCT116 cells [83].

Altogether, our previous microarray expression data (Additional file 7: Table S4) together with these reporter assays (Figure 5) suggest that neuroectodermic, RasGrf1-expressing, pancreatic islet β-cells determine a specific transcriptional microenvironment that includes specific transcriptional machinery/mechanisms of control of Pttg1 expression by RasGrf1. Furthermore, our demonstration that the modulation of Pttg1 expression by RasGrf1 is mediated by ERK signaling in pancreatic cells is also consistent with previous similar observations in other cell types, such as the MCF7 and U87 breast cancer cell lines [84, 85].

### Phenotypic interplay between RasGrf1 and Pttg1 in RasGrf1/Pttg1 double knockout mice

In view of the above results and the phenotypic similarities exhibited by single RasGrf1 or Pttg1 KO mice, we wished to further explore any potential functional overlapping or interactions occurring between RasGrf1 and Pttg1 in the endocrine pancreas. For this purpose, we crossed our RasGrf1 KO mice [17] with Pttg1 null animals originated in S. Melmed’s lab [86]. WT and double knockout (DKO) mice arising from these crosses were interbred for several generations to produce a homogeneous genetic background allowing meaningful functional comparisons between the different genotypes.

**Figure 6:**
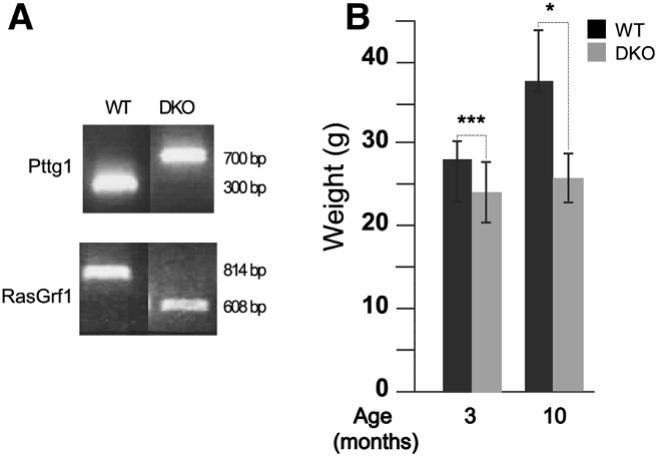
**Characterization of double knockout (DKO) RasGrf1/Pttg1 mice. (A)** Diagnostic PCR genotyping analyses of the RasGrf1 and Pttg1 loci in genomic DNA extracted from the tail of WT and double knockout (DKO) RasGrf1-Pttg1 mice. **(B)** Bar charts comparing the body weights of 3-month-old and 10-month-old WT and DKO RasGrf1-Pttg1 mice. Error bars indicate SD. * p < 0.05. ***; p < 0.001; n = 10 for WT; n = 13 for DKO.

Our initial analysis of the DKO RasGrf1/Pttg1 mice generated (Figure 6A) documented that they were viable and fertile, and did not show any gross phenotypic differences in comparison to their single KO parental ancestors. In particular, their birth rates, lifespan and body weight were rather similar to those of single RasGrf1 or Pttg1 KO mice [86]. Interestingly, we observed that the body mass of the RasGrf1-Pttg1 DKO mice was significantly reduced (about 60-70%) in both males and females at 3 and 10 months of age (Figure 6B and data not shown) as compared with their WT controls, but this reduction was quantitatively similar, and not stronger than that already previously described for individual RasGrf1 and Pttg1 KO mice [17, 86]. The observation that the simultaneous disappearance of RasGrf1 and Pttg1 did not cause any additive or synergistic effects over the reduction of body mass previously described in the individual RasGrf1 or Pttg1 KO mice may be consistent with the functional participation of these two proteins at different levels of the same cellular signaling pathways involved in controlling these body growth aspects. On the other hand, not all phenotypic aspects previously described for RasGrf1 or Pttg1 KO are shared. Thus, In spite of the association previously described between RasGrf1 and Pttg1 in the mice retina [16, 26, 86], no photoreception defects were observed in our single Pttg1 KO animals (**data not shown**).

**Figure 7:**
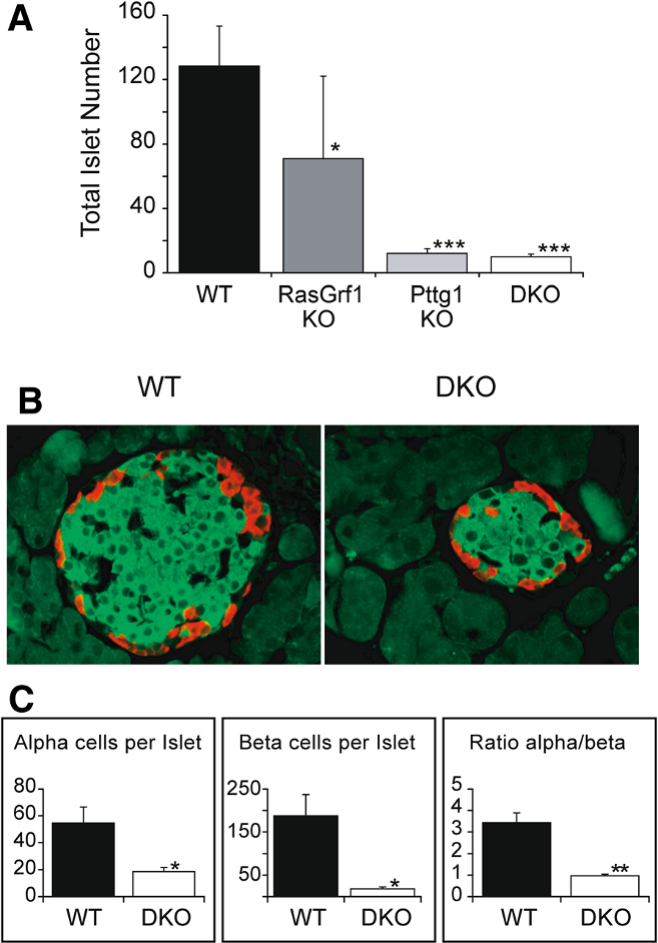
**Quantitation of pancreatic islets and related cell lineages in RasGrf1 and Pttg1 KO mice. (A)**. Total pancreatic islet count in 8 month-old animals of the indicated genotypes. Count was performed under a dissecting microscope analyzing collagenase digests of total pancreas from WT, single KO and double KO mice at 8 months of age. Error bars indicate SD. * p < 0.05; ** p < 0.01; *** p < 0.005; n = 10 for WT; n = 11 for RasGrf1 KO; n = 6 for Pttg1 KO and n = 4 for RasGrf1/Pttg1 DKO. **(B)**. Specific immunostaining of alpha (red) and beta (green) cells in pancreatic islets of WT and RasGrf1/Pttg1 DKO mice. Inmmunofluorescent staining for insulin (green) and glucagon (red) producing cells in paraformaldehyde-fixed pancreatic sections from WT and DKO mice (400 X). **(C)**. Normalized numbers of alpha and beta cells in pancreatic islets of WT and RasGrf1/Pttg1 DKO mice. Numbers of alpha or beta cells per individual islet, as well as beta/alpha ratio in total pancreas are represented. The data documents a significantly higher reduction of the beta cell compartment in the DKO mice. Error bars indicate SD. * p < 0.05; ** p < 0.01; *** p < 0.005; n = 3.

As some of the main phenotypic traits shared between single KO mice for the RasGrf1 and Pttg1 loci included reduced body weight, hypoinsulinemia and defective β-cell development and glucose tolerance responses [18], we also aimed at analyzing how the combination of both KO lineages affected these parameters in DKO animals, in order to further characterize potential functional interactions between RasGrf1 and Pttg1 in the cells of pancreatic islets. In particular, as a reduced number of pancreatic islets was previously reported in single RasGrf1 or Pttg1 KO animals when compared to their control counterparts [17, 31], we wished to evaluate the effect of the simultaneous disruption of both proteins on both the number and size of pancreatic islets and their content of alpha and beta cells in DKO mice. Our analysis of collagenase-digested pancreases of 8 month-old animals of the four different genotypes on the same genetic background (Figure 7) showed that single RasGrf1 KO mice exhibited about 50% reduction in the islet number per organ in comparison to the WT controls, whereas the single Pttg1 KO showed a dramatically higher reduction (about 90%) and the DKO RasGrf1/Pttg1 did not show statistically significant quantitative differences of such islet count in comparison to the single Pttg1 KO mice (Figure 7A,B).

In addition, specific immunostaining of the islets for glucagon-producing alpha cells and insulin-secreting beta cells documented that the observed reduction of the pancreatic islet pool in DKO mice was much more significantly linked to a reduction of the beta cell compartment than the alpha cell compartment in the pancreatic islets of DKO mice devoid of both RasGrf1 and Pttg1 (Figure 7C, D). These observations are consistent with, and confirm previous reports documenting a significant reduction of beta cell counts in the single KO RasGrf1 or Pttg1 KO mice [17, 31].

Although we have previously identified RasGrf1 as an important mediator of beta cell development and proliferation [17, 31], the much more drastic –decisive– quantitative effect of the absence of Pttg1 (about 90% inhibition) than RasGrf1 (about 50% inhibition) on development of beta cells in the endocrine pancreas, suggests a closer proximity of Pttg1 over RasGrf1 to the actual downstream mechanistic components of signaling pathways that control beta cell development and proliferation. This notion of RasGrf1 and Pttg1 participating in the same signaling pathways, but RasGrf1 acting more distally than Pttg1 to the final effectors of those pathways that are directly responsible for beta cell development would also be consistent with our previous observations that the RasGrf1 KO mice display a specific reduction of about 50% of Pttg1 expression (Additional file 1: Table S1, Figure 4), as opposed to the total absence of Pttg1 occurring in the Pttg1 KO mice.

**Figure 8:**
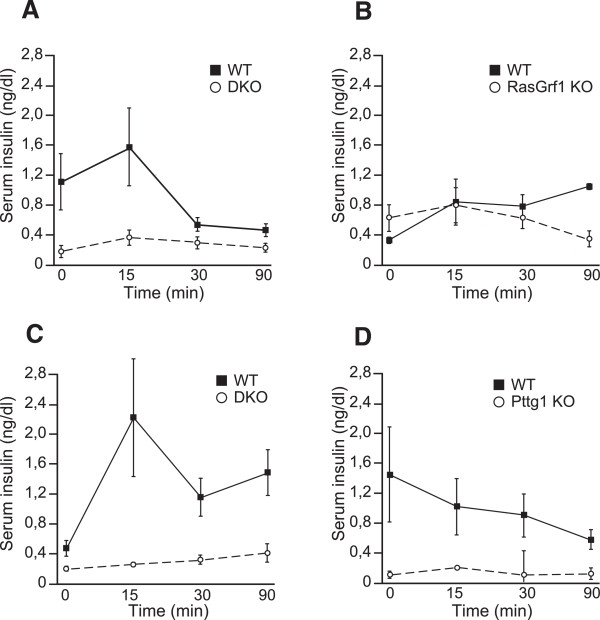
**Glucose tolerance tests in WT and single or DKO mice for the RasGrf1 and Pttg1 loci.** Male animals of the indicated ages and genotypes were fasted for 16 h and then injected intraperitoneally with a bolus of 2 mg glucose/g body weight. Serum levels of insulin were measured by ELISA after blood extraction at the times indicated. **(A)** Blood insulin in 3 month-old RasGrf1/Pttg1 DKO animals basal (0 min) and upon glucose injection. **(B)** Blood insulin induced by glucose injection in 10 month-old DKO mice. **(C)** Insulin levels in RasGrf1 KO mice at 10 months of age upon glucose injection. **(D)** Insulin levels in Pttg1 KO animals at 10 months upon intraperitoneal glucose injection. Error bars indicate SD. * p < 0.05; n = 7 for 3 month-old WT; n = 12 for 3 month-old DKO; n = 6 for 10 month-old WT; n = 12 for 10 month-old DKO; n = 6 for single RasGrf1 KO and n = 4 for their WT controls; n = 3 for single Pttg1 KO mice and n = 5 for their WT controls.

The preeminence of Pttg1 over RasGrf1 regarding the generation of the above mentioned islet- and beta cell-related phenotypes is also supported by the results of our assays of insulin release response and glucose tolerance tests carried out after administration of a bolus of glucose to the DKO mice (Figure 8). Indeed, analysis of serum insulin in our KO animals confirmed that both the single RasGrf1 and Pttg1 KO mice exhibited lower levels of serum insulin than their WT counterparts [17, 86], but the levels of individual Pttg1 KO mice were already very low and similar to those of the DKO mice and certainly lower than those of single RasGrf1 KO mice (Figure 8A-D). Parallel GTT assays (**not shown**) were also consistent with the insulin release assays, but did not show statistically significant differences between the behavior of the DKO mice in comparison to the previously described behavior of the individual KO for RasGrf1 and Pttg1.

Altogether, our results suggest that Pttg1 is an important signaling molecule acting downstream of RasGrf1 in signal transduction pathways relevant for control of beta cell development and physiological responses dependent upon pancreatic islet function.

## Conclusions

Besides defective memory/learning and impaired eye vision abilities, RasGrf1 KO mice also exhibit a reduced body size linked to a “pancreatic” phenotype that includes significantly reduced number and size of pancreatic islets, diminished beta cell proliferation and neogenesis, and associated hypoinsulinemia and glucose intolerance, suggesting an essential role of RasGrf1 in beta cell development and function.

The remarkable similarities existing among the “pancreatic” phenotypes of our RasGrf1 KO mice and those independently described for Cdk4 KO mice , p70S6K KO mice or Pttg1 KO mice, suggest the possibility that RasGrf1 and the other three different signaling molecules may contribute functionally at different levels of the same Ras-dependent signaling pathways acting in the islet cell lineages regulating pancreatic endocrine functions.

In order to try and ascertain the functional role of RasGrf1 in pancreatic islet and beta cell function, here we used commercial microarrays to characterize the specific transcriptional profile caused by the disappearance of RasGrf1 from the pancreatic islets of our RasGrf1 KO mice. Our data uncovered differential expression of multiple genes coding for components/regulators of MAPK- and calcium-signaling pathways in pancreatic islets, suggesting a relevant contribution of RasGrf1 to the control of endocrine functions of the different cell lineages present in pancreatic islets.

The transcriptional profile of pancreatic islets was significantly different from that of other neuroectodermal tissues (i.e., retina, olfactory bulb, cerebral cortex, or hippocampus) isolated from the same RasGrf1 KO mice but, remarkably, shared with all of them a common alteration involving the significant transcriptional repression of the Pttg1 locus.

Interestingly, analysis of the promoter region of mPttg1 identified specific recognition sites for numerous Transcription Factors which were also differentially expressed in the RasGrf1 KO pancreatic islets and are also known to be relevant for Ras-MAPK signaling as well as for pancreatic islet development and beta cell function. In addition, reporter luciferase assays in transfected BTC3 insulinoma cells overexpressing RasGrf1 documented the ability of RasGrf1 to modulate Pttg1 promoter activity through ERK-dependent signals. Furthermore, characterization of RasGrf1/Pttg1 double KO mice generated in our laboratory showed that the combined knockout of both loci caused pancreatic phenotypic alterations which were quantitatively close to those of single Pttg1 KO mice and much more prominent than those measured in single RasGrf1 KO mice.

Our data supports the existence of close functional and regulatory links between RasGrf1 and Pttg1 in the cell lineages of pancreatic islets, where RasGrf1 appears to be a critical signaling component acting upstream of Pttg1 in pathways controlling beta cell development and function.

## Methods

### Purification of pancreatic islets RNA isolation and RT-PCR analysis

3-Month-old male C57BL/6 N wild type and RasGrf1 KO mice were sacrificed and their pancreases carefully digested with collagenase P (Roche, Switzerland). Pancreatic islets were handpicked under a stereo microscope, counted and subsequently washed with HBSS buffer (Invitrogen, CA, USA) as previously described [87]. Islets isolated from three separate animals of the same genotype (wild type or RasGrf1 KO) were pooled in order to obtain sufficient amounts of RNA for analysis under each experimental condition. A total number of eighteen individual animals (9 WT and 9 KO) were used to generate the samples analyzed in this study.

Total RNA from either islets or total pancreas was then extracted as previously described [88]. Quantification and quality of total RNA was determined using RNA 6000 nanochips (Agilent Technologies, CA, USA). RNA sample purity from isolated pancreatic islets was estimated by comparing it with RNA extracted from total pancreas. For this purpose the Ct values of total pancreas RNA was compared to that of isolated islet RNA as previously described [35]. Quantitative real-time PCR analysis was made by measuring insulin and amylase expression using beta-2 microglobulin (B2MG) as internal control. The enrichment of the islets’ RNA was calculated using the ratio (islet insulin/pancreatic insulin)/(islet amylase/pancreatic amylase). Primers used in the analysis were: Insulin1, 5′-CCTGTTGGTGCACTTCCTAC-3′, 5′-TTGTGGGTCCTCCACTTCAC-3′, Insulin2 5′-TGGCCCTGTGGATGCGCTT-3′, 5′-GCGGGACATGGGTGTGTAGA-3′, amylase, 5′-GGAACATGGTTGCCTTCAGG-3′, 5′-GGAACATGGTTGCCTTCAGG-3′, B2MG 5′-GTGCTTGTCTCACTGACC-3′, 5′-CTGTCATGCTTAACTCTGC-3′

For RasGrf1 expression analysis, 3 μg islet RNA extracted from wild type or RasGrf1 KO islets were reverse-transcribed at 50°C for 1 h with RT-PCR kit according to manufacturer instructions (Invitrogen, CA, USA). 1/10 islet cDNA was used in subsequent PCR reactions. The oligonucleotides against RasGrf1 used in the study were: LM5 5′-GCCTGCACAACTACAATGCTGTGCTGGAGA-3′, LM85 5′-TGACCAGGCCGTCCTCTGTGTAATTGG-3′, MA5F 5′-CCTGGTCAACTTCTCCAAGA-3′, MA2R 5′-TATATTCTCGGGGAAGCACACGC-3′, MA1F 5′-GTTCTCCCCACCCACTGGCA-3′, GAPDH 5′-TGCACCACCAACTGCTTAGC-3′, 5′-TCTTCTGGGTGGCAGTGATG-3′.

### Microarray hybridization and data analysis

Labeled cRNA was obtained according to a protocol previously described [89]. Biotinylated cRNA from three different wild type samples and three RasGrf1 KO samples (each being an RNA pool obtained from three different animals of the relevant genotype), were checked for quality in Test3 arrays, before hybridization to MOE430A oligonucleotide arrays, containing 22690 oligonucleotide probe sets, corresponding to about 20000 mice genes (Affymetrix, CA, USA). Stained microarrays were scanned in a GeneArray Scanner (Hewlett Packard, CA, USA). All in all, data from 6 separate microarray hybridizations were analyzed in this study.

Using Bioconductor and R as computational tools (http://www.bioconductor.org), the RMA (Robust Microarray Analysis) algorithm [36] was applied for background correction and normalization of fluorescent hybridization signals as previously described [89]. Thus, we obtained quantization of expression level of probe sets in each microarray. SAM (Significance Analysis of Microarrays) algorithm [37] was used to identify probe sets displaying significant differential expression when comparing the KO samples to their respective controls using the calculation of the FDR (False Discovery Rate) parameter [90]. The matrix of expression values for all microarray hybridizations performed was analyzed using the *hclust* clustering algorithm implemented in R [91]. DAVID bioinformatics resources consist of an integrated biological knowledgebase and analytic tools aimed at systematically extracting biological meaning from large gene/protein lists [41]. Data analysis in this software used the EASE score, a modified (more stringent) Fisher exact p-value, as statistical filter [92]. As DAVID, Genecodis algorithm allows finding relationships among annotations based on co-occurrence patterns (http:/genecodis.dacya.ucm.es/; [59]. For transcription factor annotations it uses mouse TR list contained in the GSEA database [93], which are derived from a previous work by Xie and co-workers [94].

### Western blotting, immunoprecipitations and immunostaining

For immunobloting, manually isolated islets from 3 month-old male mice were homogenized in 100 μl RIPA buffer and separated in SDS-PAGE gels as previously described [32]. The gels were then transferred to nitrocellulose membranes using a semi-dry iBlot® system (Invitrogen). Finally, membranes were blocked in 2% BSA in TBS-T (20 mM Tris/HCl (pH 7.5), 150 mM NaCl and Tween-20 al 0.05%) from 30’ to 2 hours. Next step was incubation with the primary antibody of interest in 2% BSA in TBS-T at 4°C overnight or 2 hours at RT. We used anti-Pttg1 1:1000 (Immunostep); anti-RasGrf1/2 1:1000 (sc-224 Santa Cruz Inc, CA, USA); anti-PDGFR 1:500 (sc-338 Santa Cruz Inc, CA, USA); anti-FGFR3 1:500 (sc-123 Santa Cruz Inc, CA, USA);, anti-TXNL 1:200 (A22165A, Genway Biotech Inc. San Diego, CA, USA); anti-Ras (Ras M90 monoclonal antibody [95] and β-tubulin 1:5000 (Sigma).

For immunoprecipitation, manually selected islets were homogenized in RIPA buffer. Lysates were clarified by centrifugation at 15,000 × *g* for 10 min. After protein content determination, equal amounts of protein (500–600 μg) were immunoprecipitated with anti-RasGrf1/2 antibody (sc-224 Santa Cruz Inc, CA, USA) at 4°C.

The immune complexes were collected on protein A-agarose beads (Upstate, CA, USA). Immunoprecipitates were washed with lysis buffer and extracted for 5 min at 95°C in 4× SDS-PAGE sample buffer (200 mM Tris/HCl, 6% SDS, 2 mM EDTA, 4% 2-mercaptoethanol, 10% glycerol, pH 6.8) and analyzed by SDS-PAGE.

Wild Type and mutant adult mice were anesthetized with chloral hydrate (5% [wt/vol] in saline, 10 ml/g of body weight [given intraperitoneally]) and perfused through the aorta, first with heparinized saline (5 IU/ml; Byk Leo, Madrid, Spain) and then with a solution containing 4% (wt/vol) depolymerised paraformaldehyde and 0.2% (wt/vol) picric acid in 0.1 M phosphate buffer (pH 7.4) (PBS) for 20 min. Pancreas were dissected and postfixed in the same solution for 2 h at 4°C. Paraffin embedded Sections were stained using a Ventana Discovery XT Staining Station. First antibodies and dilutions used were as follows: Anti-Insulin (C27C9) Rabbit mAb (Cell signalling Cat. No. 3014); Anti-Glucagon clone K79bB10, ascites fluid (Sigma-aldrich G2654); Anti-Gria2 1:100 (Chemicon AB1768) and Anti-Phtf1 1:200 (generously donated by Dr. Raich [96]). FITC-goat anti-Rabbit; CY3-goat anti-mouse and HRP- goat anti-rabbit were used as secondary antibodies for immunofluorescence and immunocytochemistry respectively. Sections were then washed in PBS, mounted in fluoromount Vectashield, (Vector Laboratories) and cover slipped for viewing in a Leica DM 6000B microscope. Images were acquired using a Hamamatsu ORCA ER camera controlled with the Metamorph image acquisition software.

### Culture of pancreatic islets and BTC3 cells, transient transfections and luciferase reporter assays

Manually isolated islets obtained as described [17] were centrifuged in HBSS for 5 min at 250 × g. Supernatant was discarded and islets were resuspended in 1.5 ml HBSS 3 mM EGTA, 20 mg/ml BSA and 2.2 mg/ml glucose. Islet solution was mixed with 48.5 ml trypsin solution (0.25% trypsin in PBS) and incubated for 10 minutes at 37°C and 5% CO_2_. Islets were passed through a 20 gauge needle and centrifuged at 1500 rpm during 10 minutes. Cells were washed twice with PBS and cultured on 2 mg/ml collagen-coated (Roche, Switzerland) coverslips in RPMI 10% FBS medium.

BTC3 cells [97] were grown in RPMI (Gibco) supplemented with fetal bovine serum (10% FBS; Hyclone, Logan, Utah, USA), glutamine (2 mM), penicillin (100 U/ml) and streptomycin (100 μg/ml), in a humidified tissue culture incubator (Hera cell, Heraeus, Thermo Fisher Scientific Inc.) at 5% CO_2_ and 37°C.

pBK-CMV RasGrf1 containing the full-length RasGrf1 coding sequence, and the reporter plasmid construct pGL3-Pttg1 containing 2,3Kb promoter region, were kindly supplied by Drs. D. Lowy and S. Melmed [81] respectively. Cells were split into 12-well plates, and each well was cotransfected, using Lipofectamine 2000® (Invitrogene), with 1 μg of a plasmid containing the hPTTG1 promoter linked to luciferase and 1 μg pBK-CMV RasGrf1, or 1 μg empty pBK-CMV vector as control. pRL-Tk (Promega) encoding renilla luciferase (5 ng/well) was used as an internal control to assess transfection efficiency. 48 h hours after transfection cells were stimulated with the indicated agonists/inhibitors for 7 h [Ionomycin 500 nM (Sigma), Epidermal Growth Factor (EGF) 100 ng/ml (Peprotech), Lysophosphatidic acid (LPA) 10 μM (Sigma), Insulin Growth Factor (IGF) 10 ng/ml (Sigma), U0126 (25 μM) (Promega), Wortmannin 100 nM (Sigma), SP600125 20 μM (Calbiochem)]. Whole-cell lysates were collected for reporter detection by luciferase assays using a dual luciferase reporter kit (Promega, Madison, WI, USA). Reactions were measured using a Lumat LB 9507 Tube luminometer (Berthold Technologies GmbH & Co. KG).

### Metabolic studies

Animals of the same sex were housed in type IIL individually ventilated cages, in a temperature- and humidity-controlled room with a 12 h light/dark cycle, with food (Teklad 2014, Harlan Laboratories) and water available ad libitum. All testing was completed during the light phase. Animal housing and experimentation followed the general recommendations of the European Communities Council Directive of 86/609/EEC and the RD 1201/05 about the use of experimental animals with scientific aims. Maximal efforts were made to minimize the total number of animals as well as their suffering. All animal experimental procedures were approved by the local Animal Ethics Committee of the University of Salamanca.

Blood for measuring plasma insulin levels [98] was obtained by submandibular injection in mice anesthetized by isofluorane inhalation (Abbot laboratories Ltd, UK). Plasma insulin levels were measured by enzyme-linked immunosorbent assay (Rat/Mouse Insulin ELISA kit; Millipore). We performed glucose tolerance tests on animals after a 14–16 h overnight fast. For these studies, glucose (2 mg glucose/g body weight) was injected intraperitoneally. The One-way ANOVA test was used for statistical analyses but whenever Levene’s test was significant, we used Welch statistical result [99, 100]. Bonferroni and Gabriel *post hoc* tests were also used [101].

## Availability of supporting data

All microarray hybridization data have been deposited and are available at the NCBI, Gene Expression Omnibus database (GEO accession series GSE56371; http://www.ncbi.nlm.nih.gov/geo/query/acc.cgi?acc=GSE56371).

## Electronic supplementary material

Additional file 1: Table S1: Differential gene expression in pancreatic islets of RasGrf1 KO mice. List of 4525 differentially expressed probesets (3592 different genes) identified by means of SAM contrasts (FDR=0.084) comparing the microarray-generated transcriptional profile of purified pancreatic islets of RasGrf1 KO mice to those of WT control mice. Differentially expressed loci are identified by *Affymetrix Probeset ID, Symbol and Gene Name*, and listed according to degree of overexpression or repression, in decreasing order of d-values. Red: overexpression. Green: repression. *d-value* is a parameter measuring the statistical distance separating the calculated expression value of each gene probeset from the null hypothesis (no-change). *q-value* is the estimated FDR at the largest p-value for which the probe set would be statistically significant. *R-fold* is a measure of the fold change of a probeset in the collection of microarrays provided by the SAM algorithm. Values in red denote overexpression. Values in green denote transcriptional repression. *NULL* denotes probeset not recognizing any known transcribed mouse genomic sequence. (PDF 769 KB)

Additional file 2: Figure S1: Transcriptional behavior of genomic sequences located at the 3′ UTR terminal end of the RasGrf1 gene. **(A)** Hybridization signals produced by Affymetrix probeset *1435614_s_at* recognizing the 3′ UTR region of the RasGrf1 locus. Bar plot showing normalized hybridization signals produced by the *1435614_s_at* probeset in 6 independent, separate microarray hybridizations with RNA from pancreatic islets including 3 samples from RasGrf1 KO and 3 samples from WT mice. **(B)** Localization of specific genomic sequences of the 3′ terminal region of RasGrf1 gene that recognized by Affymetrix probesets and primer oligonucleotides used in this study. The coding region is shown in capitals and the 3′ UTR region is shown in italics. The position of the relevant oligonucleotides mentioned in the text (LM5F, LM85R, MA5F, MA1F and MA2R) is indicated by boxes and color changes as appropriate in each case. **(C)** Confirmatory RT-PCR analysis of WT and RasGrf1 KO RNAs from pancreatic islets. The primer set LM5/LM85 amplifies the 3554–3829 nt region in RasGrf1 mRNA sequence. Primer set MAF5/MA2R amplifies the 3830–4156 nt region, and the set MA1F/MA2R amplifies the 4012–4156 nt segment. Specific oligonucleotides for GAPDH amplified a 90 bp band in both WT and RasGrf1 KO RNA samples. Representative results of three independent experiments are shown. (JPEG 6 MB)

Additional file 3: Table S2A: Functional annotation of downregulated, differentially expressed genes in pancreatic islets of RasGrf1 knockout mice. The DAVID functional annotation tool (http://david.abcc.ncifcrf.gov/) was used to identify statistically significant functional associations (p-value <0.1) linking particular gene subsets contained within the list of repressed loci occurring in RasGrf1 KO pancreatic islets (Additional file 1: Table S1, FDR=0.084) to specific Gene Ontology (GO) terms. The column labelled “*Biological process*” identifies the functional GO terms (level 5) recognized in each case for the corresponding groups of loci listed under the column labelled “*Genes induced in RasGrf1KO pancreatic islets (from Additional file* 1 *: Table S1)*. The column labeled “*Gene Count*” indicates the specific number of genes annotated by DAVID to the indicated GO functionality within the list of repressed genes included in Table S1 (Additional file 1). The values under the column “*Percentage*” are calculated by referring the “*Gene Count*” numbers to the total number of genes recognized by DAVID (1942, out of a total 1953) within that list. The column labeled “*p-value*” refers to the statistical significance of the functional associations identified, and contains p-values calculated using the Hypergeometric Distribution and subsequently corrected by implementing the False Discovery Rate (FDR) method [90]. (PDF 466 KB)

Additional file 4: Table S2B: Functional annotation of upregulated, differentially expressed genes in pancreatic islets of RasGrf1 knockout mice. The DAVID functional annotation tool (http://david.abcc.ncifcrf.gov/) was used to identify statistically significant functional associations (p-value <0.1) linking particular gene subsets contained within the list of induced loci occurring in RasGrf1 KO pancreatic islets (Additional file 1: Table S1, FDR=0.084) to specific Gene Ontology (GO) terms. The column labelled “*Biological process*” identifies the functional GO terms (level 5) recognized in each case for the corresponding groups of loci listed under the column labeled “*Genes induced in RasGrf1 KO pancreatic islets (from Additional file* 1 *: Table S1)”*. The column labeled “*Gene Count*” indicates the specific number of genes annotated by DAVID to the indicated GO functionality within the list of induced genes included in Table S1 (Additional file 1). The values under the column “*Percentage*” are calculated by referring the “*Gene Count*” numbers to the total number of probesets recognized by DAVID (1781, out of a total 1799) within that list. The column labeled “*p-value*” refers to the statistical significance of the functional associations identified, and contains p-values calculated using the Hypergeometric Distribution and subsequently corrected by implementing the False Discovery Rate (FDR) method [90]. (PDF 312 KB)

Additional file 5: Table S3A: Altered KEGG pathways identified by DAVID analysis of down-regulated (S3A), differentially expressed genes in pancreatic islets of RasGrf1 knockout mice. The DAVID functional annotation tool (http://david.abcc.ncifcrf.gov/) to identify statistically significant functional associations linking particular gene subsets contained within the list of repressed loci occurring in RasGrf1 KO pancreatic islets (Additional file 1: Table S1, FDR=0.084) to specific KEGG pathways (Kyoto Encyclopaedia of Genes and Genomes; http://www.genome.jp/kegg). The “*KEGG Pathway*” column identifies the signaling pathway annotated in each case to the corresponding group of loci listed in the column labeled “*Genes repressed in RasGrf1 KO pancreatic islets (from Additional file* 1 *: Table S1)*”. The column labeled “*Gene Count*” indicates the specific number of genes linked to the indicated signaling pathway within the list of repressed genes included in Table S1 (Additional file 1). The values under the column “*Percentage*” are calculated by referring the “*Gene Count*” numbers to the total number of genes recognized by DAVID (1943, out of a total 1953 genes, corresponding to 2268 probesets) within that list. The column labeled “*p-value*” refers to the statistical significance of the functional associations identified, and contains p-values calculated cases using the Hypergeometric Distribution and subsequently corrected by implementing the False Discovery Rate method [90]. (PDF 172 KB)

Additional file 6: Table S3B: Altered KEGG pathways identified by DAVID analysis of up-regulated, differentially expressed genes in pancreatic islets of RasGrf1 knockout mice. The DAVID functional annotation tool (http://david.abcc.ncifcrf.gov/) to identify statistically significant functional associations linking particular gene subsets contained within the list of induced loci occurring in RasGrf1 KO pancreatic islets (Additional file 1: Table S1, FDR=0.084) to specific KEGG pathways (Kyoto Encyclopaedia of Genes and Genomes; http://www.genome.jp/kegg). The “*KEGG Pathway*” column identifies the signaling pathway annotated in each case to the corresponding group of loci listed in the column labeled “*Genes repressed in RasGrf1 KO pancreatic islets (from Additional file* 1 *: Table S1)*”. The column labeled “*Gene Count*” indicates the specific number of genes linked to the indicated signaling pathway within the list of induced genes included in Table S1 (Additional file 1). The values under the column “*Percentage*” are calculated by referring the “*Gene Count*” numbers to the total number of genes recognized by DAVID (1777, out of a total 1799 genes, corresponding to 2256 probesets) within that list. The column labeled “*p-value*” refers to the statistical significance of the functional associations identified, and contains p-values calculated cases using the Hypergeometric Distribution and subsequently corrected by implementing the False Discovery Rate method [90]. (PDF 166 KB)

Additional file 7: Table S4: Concurrent transcriptional alterations at different tissues of neuroectodermal origin in RasGrf1 KO mice. List of 29 different loci showing significant level of parallel, concomitant differential gene expression in two or more separate microarray hybridization analyses of samples from the indicated tissue locations of RasGrf1 KO mice. Origin of the microarray expression data for each tissue was as follows: Pancreatic islets: MOE430A arrays, Table S1 (Additional file 1) in this work. LCM-purified hippocampus pyramidal cells: MOE430A arrays, [32]. Retina: MOE430_2 arrays, [18]. Olfactory bulb, full hippocampus, and brain cortex: Mouse Exon 1.0 ST arrays, unpublished data. Red: Upregulation. Blue: Downregulation. LCM: Laser capture microdissection. No arrow: no significant transcriptional change detected. (PDF 99 KB)

Additional file 8: Table S5A: Transcription factors identified by functional annotation of differentially expressed, repressed genes in RasGrf1 KO pancreatic islets. The GeneCodis functional annotation tool (http://genecodis.cnb.csic.es/) was used to identify specific subsets within the list of repressed genes of RasGrf1 KO pancreatic islets (Additional file 1: Table S1, FDR=0.08; 2198 recognized repressed loci, out of a total 2268 probesets listed) that share co-occurrent functional annotations linking them to specific Transcription Factors (TransFac database) at high statistically significant p-values. The “*Transcription Factor*” column identifies individual transcription factors recognized by GeneCodis as capable of controlling expression of the corresponding groups of loci listed in each case under the column labeled “*Genes repressed in RasGrf1 KO pancreatic islets*”. The column labeled “*Gene Count*” indicates the specific number of genes identified in each of those groups. Values in the “*Percentage*” column are calculated referring the “*Gene Count*” column numbers to the total number of repressed, input genes (2198 repressed loci from Additional file 1: Table S1) recognized by the functional annotation software. The column labeled “*p-value*” refers to the statistical significance of the functional associations identified. (PDF 287 KB)

Additional file 9: Table S5B: Transcription factors identified by functional annotation of differentially expressed, induced genes in RasGrf1 KO pancreatic islets. The GeneCodis functional annotation tool tool (http://genecodis.cnb.csic.es/) was used to identify specific subsets within the list of induced genes of RasGrf1 KO pancreatic islets (Additional file 1: Table S1, FDR=0.08; 2230 recognized overexpressed loci, out of a total 2257 probesets listed) that share co-occurrent functional annotations linking them to specific Transcription Factors (TransFac database) at high statistically significant p-values. The “*Transcription Factor*” column identifies individual transcription factors recognized by GeneCodis as capable of controlling expression of the corresponding groups of loci listed in each case under the column labeled “*Genes induced in RasGrf1 KO pancreatic islets*”. The column labeled “*Gene Count*” indicates the specific number of genes identified in each of those groups. Values in the “*Percentage*” column are calculated referring the “*Gene Count*” column numbers to the total number of induced, input genes (2198 repressed loci from Additional file 1: Table S1) recognized by the functional annotation software. The column labeled “*p-value*” refers to the statistical significance of the functional associations identified. (PDF 322 KB)

## Abbreviations

GEF: Guanosine nucleotide exchange factor
WT: Wild type
KO: Knockout
DKO: Double knockout
CNS: Central Nervous System
FDR: False discovery rate
UTR: Un-Translated Region
GO: Gene ontology
BP: Biological processes
LCM: Laser capture microdissection
TF: Transcription factors
LPA: Lysophosphatydic acid
RMA: Robust microarray analysis
SAM: Significance analysis of microarrays
B2MG: Beta 2 microglobulin
RIPA: Radio immunoprecipitation assay
BSA: Bovine serum albumin
TBS-T: Tris buffer saline plus tween
RT: Room temperature
mAb: Monoclonal antibody
HRP: Horse radish peroxydase, qRT-PCR quantitative real time PCR
RPMI: Roswell Park Memorial Institute
HBSS: Hank’s balanced salt solution
FBS: fetal bovine serum
MEF: Mouse embryo fibroblast
GTT: glucose tolerance test
WB: Western blot
ELISA: Enzyme-linked Immunosorbent Assay
ANOVA: Analysis of variance.
