# Supplementary material for: Transcriptional profiling reveals functional links between RasGrf1 and Pttg1 in pancreatic beta cells
Source: BMC Genomics. 2014 Nov 25;15:1019. doi: 10.1186/1471-2164-15-1019 (PMC4301450; doi:10.1186/1471-2164-15-1019)
Supplement: Supplementary file 1 — Additional file 1: Table S1: Differential gene expression in pancreatic islets of RasGrf1 KO mice. List of 4525 differentially expressed probesets (3592 different genes) identified by means of SAM contrasts (FDR=0.084) comparing the microarray-generated transcriptional profile of purified pancreatic islets of RasGrf1 KO mice to those of WT control mice. Differentially expressed loci are identified by Affymetrix Probeset ID, Symbol and Gene Name, and listed according to degree of overexpression or repression, in decreasing order of d-values. Red: overexpression. Green: repression. d-value is a parameter measuring the statistical distance separating the calculated expression value of each gene probeset from the null hypothesis (no-change). q-value is the estimated FDR at the largest p-value for which the probe set would be statistically significant. R-fold is a measure of the fold change of a probeset in the collection of microarrays provided by the SAM algorithm. Values in red denote overexpression. Values in green denote transcriptional repression. NULL denotes probeset not recognizing any known transcribed mouse genomic sequence. (PDF 769 KB) [file 12864_2014_6838_MOESM1_ESM.pdf]

Table S1. Differential gene expression in pancreatic islets of RasGrf1 KO mice.
List of 4525 differentially expressed probesets (3592 different genes) identified by means of SAM contrasts (FDR=0.084) comparing the microarray-generated transcriptional profile of purified pancreatic islets of RasGrf1KO mice to those of WT control mice. The differentially expressed loci are identified by *Affymetrix Probeset ID, Symbol and Gene Name*, and listed according to degree of overexpression or repression, quantitated by *d-value*. Red: overexpression. Green: repression. *d-value* is a parameter measuring the statistical distance separating the calculated expression value of each gene probeset from the null hypothesis (no-change). *q-value* is the estimated FDR at the largest p-value for which the probe set would be statistically significant. *R-fold* is a measure of the fold change of a probeset in the collection of microarrays provided by the SAM algorithm. Values in red denote overexpression. Values in green denote transcriptional repression. *NULL* denotes probeset not recognizing any known transcribed mouse genomic sequence.

Probeset ID	***Symbol***	***Gene Name***	***d-value***	***q-value***	***R-fold***
1448406_at	Eid1	EP300 interacting inhibitor of differentiation 1	23,0286	0,0484	1,8885
1418829_a_at	Eno2	enolase 2, gamma neuronal	19,6674	0,0484	2,0056
1426790_at	Ssrp1	structure specific recognition protein 1	18,9316	0,0484	1,5767
1448803_at	Golga4	golgi autoantigen, golgin subfamily a, 4	14,1590	0,0521	1,6743
1460325_at	Pum1	pumilio 1 (Drosophila)	14,0381	0,0521	1,6734
1415880_a_at	Lamp1	lysosomal-associated membrane protein 1	13,9611	0,0521	1,3363
1422801_at	G3bp1	Ras-GTPase-activating protein SH3-domain binding protein 1	13,1939	0,0521	1,7431
1418514_at	Mtf2	metal response element binding transcription factor 2	10,9518	0,0521	1,4016
1427074_at	Pcmtd2	protein-L-isoaspartate (D-aspartate) O-methyltransferase domain containing 2	10,4286	0,0521	2,0033
1455868_a_at	Tubgcp2	tubulin, gamma complex associated protein 2	10,3961	0,0521	1,3188
1426918_at	Itgb1	integrin beta 1 (fibronectin receptor beta)	10,3396	0,0521	1,5878
1450851_at	Wdr1	WD repeat domain 1	10,2920	0,0529	1,7899
1455869_at	NULL	NULL	10,0775	0,0557	18,7987
1423591_at	Fgfr1op2	FGFR1 oncogene partner 2	9,9725	0,0557	1,6877
1434019_at	Pdap1	PDGFA associated protein 1	9,8878	0,0557	1,3434
1419169_at	Mapk6	mitogen-activated protein kinase 6	9,8216	0,0557	1,5937
1426486_at	Ubxn4	UBX domain protein 4	9,6003	0,0557	1,8459
1422485_at	Smad4	MAD homolog 4 (Drosophila)	9,5413	0,0557	1,8517
1432271_a_at	Dcun1d5	DCN1, defective in cullin neddylation 1, domain containing 5 (S. cerevisiae)	9,3620	0,0557	1,4775
1415727_at	Apoa1bp	apolipoprotein A-I binding protein	9,3542	0,0557	1,8268
1419496_at	Slco1a6	solute carrier organic anion transporter family, member 1a6	9,2434	0,0557	15,1592
1448240_at	Mbtps1	membrane-bound transcription factor peptidase, site 1	9,2244	0,0557	1,7852
1416918_at	Dlg3	discs, large homolog 3 (Drosophila)	9,1756	0,0557	1,6961
1460741_x_at	D17Wsu92e	DNA segment, Chr 17, Wayne State University 92, expressed	9,0618	0,0557	1,7269
1435614_s_at	Rasgrf1	RAS protein-specific guanine nucleotide-releasing factor 1	8,9720	0,0557	4,3680
1460665_a_at	Cnot7	CCR4-NOT transcription complex, subunit 7	8,8712	0,0557	1,3658
1438398_at	NULL	NULL	8,8413	0,0557	1,8224
1427108_at	9530068E07Rik	RIKEN cDNA 9530068E07 gene	8,8021	0,0557	1,8473
1428337_at	Mdp1	magnesium-dependent phosphatase 1	8,7989	0,0557	1,2284
1451972_at	NULL	NULL	8,7289	0,0557	1,7731
1427874_at	Rnf114	ring finger protein 114	8,6264	0,0557	1,6357
1450983_at	Akap8	A kinase (PRKA) anchor protein 8	8,5962	0,0557	1,5516
1453287_at	Ankrd33b	ankyrin repeat domain 33B	8,5917	0,0557	1,9108
1426245_s_at	Mapre2	microtubule-associated protein, RP/EB family, member 2	8,5215	0,0557	1,7233
1417030_at	Tmem206	transmembrane protein 206	8,5184	0,0557	2,2573
1452247_at	Fxr1	fragile X mental retardation gene 1, autosomal homolog	8,4807	0,0557	1,7802
1435680_a_at	Dpp7	dipeptidylpeptidase 7	8,4787	0,0557	2,0581
1455242_at	Foxp1	forkhead box P1	8,3903	0,0560	1,8937
1448464_at	Ykt6	YKT6 homolog (S. Cerevisiae)	8,3481	0,0562	1,2318
1430127_a_at	Ccnd2	cyclin D2	8,2741	0,0576	1,8543
1417646_a_at	Snx5	sorting nexin 5	8,2510	0,0576	1,2393
1426208_x_at	Plagl1	pleiomorphic adenoma gene-like 1	8,2234	0,0576	2,3045
1420679_a_at	Aig1	androgen-induced 1	8,2085	0,0576	1,5615
1450386_at	Kpna3	karyopherin (importin) alpha 3	8,1641	0,0576	1,6776
1451415_at	1810011O10Rik	RIKEN cDNA 1810011O10 gene	8,1204	0,0576	1,9635
1460182_at	Snx4	sorting nexin 4	8,1040	0,0576	1,7240
1423780_at	Hibadh	3-hydroxyisobutyrate dehydrogenase	8,0922	0,0576	1,6953
1452607_at	2610030H06Rik	RIKEN cDNA 2610030H06 gene	8,0895	0,0576	1,4949
1423936_at	Kctd5	potassium channel tetramerisation domain containing 5	8,0790	0,0576	1,2267
1433668_at	Pnrc1	proline-rich nuclear receptor coactivator 1	8,0062	0,0576	1,7916
1452709_at	Poldip3	polymerase (DNA-directed), delta interacting protein 3	7,9626	0,0581	1,9252
1454606_at	4933426M11Rik	RIKEN cDNA 4933426M11 gene	7,9609	0,0581	1,9304
1415780_a_at	Armcx2	armadillo repeat containing, X-linked 2	7,9570	0,0581	1,7846
1434103_at	Slc35e1	solute carrier family 35, member E1	7,7823	0,0581	1,4811
1454608_x_at	Ttr	transthyretin	7,7680	0,0581	1,7564
1417490_at	Ctsb	cathepsin B	7,7133	0,0581	1,4949
1450264_a_at	Chka	choline kinase alpha	7,6939	0,0581	1,9120

Probeset ID	Symbol	Gene Name	d-value	q-value	R-fold
1415766_at	Sec22b	SEC22 vesicle trafficking protein homolog B (S. cerevisiae)	7,6932	0,0581	1,7023
1419672_at	Spock1	sparc/osteonectin, cwcv and kazal-like domains proteoglycan 1	7,6653	0,0581	1,8269
1452666_a_at	Tmcc2	transmembrane and coiled-coil domains 2	7,6288	0,0581	2,3797
1415911_at	Impact	imprinted and ancient	7,5663	0,0594	1,5056
1437210_a_at	Brd2	bromodomain containing 2	7,5324	0,0594	2,1525
1429534_a_at	Immt	inner membrane protein, mitochondrial	7,5162	0,0594	1,6409
1426247_at	Stk24	serine/threonine kinase 24 (STE20 homolog, yeast)	7,4748	0,0594	1,7375
1425517_s_at	Ogt	O-linked N-acetylglucosamine (GlcNAc) transferase (UDP-N-acetylglucosamine:polypeptide-N-acetylglucosaminyl transferase)	7,4596	0,0594	1,5025
1423461_a_at	Ubl3	ubiquitin-like 3	7,4446	0,0594	1,6423
1448715_x_at	NULL	NULL	7,4215	0,0594	1,7111
1417594_at	Gkap1	G kinase anchoring protein 1	7,4198	0,0594	1,5227
1416903_at	Nucb1	nucleobindin 1	7,3847	0,0599	1,4547
1416122_at	Ccnd2	cyclin D2	7,3795	0,0599	1,5274
1452035_at	Col4a1	collagen, type IV, alpha 1	7,3718	0,0600	2,5138
1448211_at	Atp6v0e2	ATPase, H+ transporting, lysosomal V0 subunit E2	7,3480	0,0600	1,7586
1425914_a_at	Armcx1	armadillo repeat containing, X-linked 1	7,2976	0,0600	1,4656
1426894_s_at	Fam102a	family with sequence similarity 102, member A	7,2874	0,0600	1,3113
1416891_at	Numb	numb gene homolog (Drosophila)	7,2700	0,0600	1,5047
1424407_s_at	Cbx6	chromobox homolog 6	7,2581	0,0600	1,7594
1449117_at	Jund	Jun proto-oncogene related gene d	7,2315	0,0600	1,1769
1416359_at	Snx18	sorting nexin 18	7,2125	0,0600	1,8128
AFFX-MURINE_B2_at	NULL	NULL	7,1994	0,0600	1,2372
1451379_at	Rab22a	RAB22A, member RAS oncogene family	7,1965	0,0600	1,3738
1425543_s_at	Plekha5	pleckstrin homology domain containing, family A member 5	7,1918	0,0600	1,6375
1417376_a_at	Cadm1	cell adhesion molecule 1	7,1740	0,0600	1,9943
1416579_a_at	Epcam	epithelial cell adhesion molecule	7,1572	0,0600	3,2625
1428505_at	Ccdc90b	coiled-coil domain containing 90B	7,0285	0,0602	1,3763
1420909_at	Vegfa	vascular endothelial growth factor A	7,0110	0,0602	1,6968
1448247_at	Bcl7b	B-cell CLL/lymphoma 7B	6,9879	0,0602	1,2989
1424925_at	Sec63	SEC63-like (S. cerevisiae)	6,9124	0,0602	1,8438
1433460_at	Ttc7b	tetratricopeptide repeat domain 7B	6,9097	0,0602	2,1824
1424547_at	Car10	carbonic anhydrase 10	6,9040	0,0602	1,9281
1451152_a_at	Atp1b1	ATPase, Na+/K+ transporting, beta 1 polypeptide	6,8752	0,0602	1,5464
1433756_at	S100pbp	S100P binding protein	6,8585	0,0602	1,7637
1418067_at	Cfl2	cofilin 2, muscle	6,8202	0,0602	1,7822
1420617_at	Cpeb4	cytoplasmic polyadenylation element binding protein 4	6,8183	0,0602	2,6096
1421191_s_at	Gopc	golgi associated PDZ and coiled-coil motif containing	6,7922	0,0602	1,5539
1423992_at	Gatad2a	GATA zinc finger domain containing 2A	6,7864	0,0602	1,5142
1417070_at	Cyp4v3	cytochrome P450, family 4, subfamily v, polypeptide 3	6,7776	0,0602	1,4939
1438625_s_at	Cdk16	cyclin-dependent kinase 16	6,7754	0,0602	2,2440
1453004_at	Slc22a23	solute carrier family 22, member 23	6,7736	0,0602	2,0810
1434080_at	Aebp2	AE binding protein 2	6,7708	0,0602	2,0416
1421147_at	Terf2	telomeric repeat binding factor 2	6,7675	0,0602	1,5629
1433448_at	Slc25a44	solute carrier family 25, member 44	6,7635	0,0602	1,5071
1423394_at	Pcyox1	prenylcysteine oxidase 1	6,7625	0,0602	1,8426
1428531_at	Ints7	integrator complex subunit 7	6,7544	0,0602	1,7799
1450064_at	NULL	NULL	6,7350	0,0602	1,6428
1426760_at	Ipo8	importin 8	6,7251	0,0602	1,6742
1417210_at	Eif2s3y	eukaryotic translation initiation factor 2, subunit 3, structural gene Y-linked	6,7171	0,0602	1,3171
1427969_s_at	Zfp654	zinc finger protein 654	6,6942	0,0602	1,3438
1451984_at	Hnrnpul1	heterogeneous nuclear ribonucleoprotein U-like 1	6,6870	0,0602	1,5551
1417606_a_at	Calr	calreticulin	6,6755	0,0609	1,4103
1448525_a_at	Bnip3l	BCL2/adenovirus E1B interacting protein 3-like	6,6726	0,0609	2,0985
1426382_at	Ppm1b	protein phosphatase 1B, magnesium dependent, beta isoform	6,6546	0,0610	1,9548
1436947_a_at	Txnl1	thioredoxin-like 1	6,6446	0,0610	1,9858
1423159_at	Dld	dihydrolipoamide dehydrogenase	6,6201	0,0610	1,7019
1434370_s_at	NULL	NULL	6,6193	0,0610	1,3072
1452999_at	Smndc1	survival motor neuron domain containing 1	6,6136	0,0610	2,1161
1423911_at	Ppp2r5a	protein phosphatase 2, regulatory subunit B (B56), alpha isoform	6,5641	0,0610	2,0223
1423803_s_at	Gltscr2	glioma tumor suppressor candidate region gene 2	6,5523	0,0610	1,6766
1451387_s_at	Cuta	cutA divalent cation tolerance homolog (E. coli)	6,5428	0,0610	1,2790
1449148_a_at	Phtf1	putative homeodomain transcription factor 1	6,5323	0,0610	1,3466
1434924_at	Phf2	PHD finger protein 2	6,5311	0,0610	1,9340
1416215_at	Gosr1	golgi SNAP receptor complex member 1	6,5247	0,0610	1,6343
1429367_at	Wipi2	WD repeat domain, phosphoinositide interacting 2	6,4783	0,0617	1,5827
1427798_x_at	NULL	NULL	6,4425	0,0620	1,6500
1451078_at	2510039O18Rik	RIKEN cDNA 2510039O18 gene	6,4220	0,0621	1,4868
1448170_at	Siah2	seven in absentia 2	6,4025	0,0625	1,3765
1422429_at	Rnf14	ring finger protein 14	6,3603	0,0625	1,4865
1460213_at	Golga4	golgi autoantigen, golgin subfamily a, 4	6,3541	0,0625	1,8546
1451437_at	Zdhhc20	zinc finger, DHHC domain containing 20	6,3525	0,0625	1,7229
1420505_a_at	Stxbp1	syntaxin binding protein 1	6,3419	0,0625	2,2423
1416364_at	Hsp90ab1	heat shock protein 90 alpha (cytosolic), class B member 1	6,3044	0,0625	1,6367
1454699_at	NULL	NULL	6,2956	0,0625	1,8355
1420842_at	Ptprf	protein tyrosine phosphatase, receptor type, F	6,2909	0,0625	1,7358
1448404_at	Scamp2	secretory carrier membrane protein 2	6,2896	0,0625	1,6201

Probeset ID	Symbol	Gene Name	d-value	q-value	R-fold
1416133_at	Efr3a	EFR3 homolog A (S. cerevisiae)	6,2726	0,0625	1,9704
1448218_s_at	Ywhaz	tyrosine 3-monooxygenase/tryptophan 5-monooxygenase activation protein, zeta polypeptide	6,2584	0,0625	1,6688
1419243_at	Rab14	RAB14, member RAS oncogene family	6,2483	0,0627	1,7932
1426713_s_at	Eprs	glutamyl-prolyl-tRNA synthetase	6,2413	0,0632	1,5789
1423532_at	Rnf44	ring finger protein 44	6,2397	0,0632	1,6314
1422607_at	Etv1	ets variant gene 1	6,1746	0,0645	2,1815
1417542_at	Rps6ka2	ribosomal protein S6 kinase, polypeptide 2	6,1253	0,0652	1,6070
1423515_at	Scn8a	sodium channel, voltage-gated, type VIII, alpha	6,1252	0,0652	1,5628
1439018_at	Fhdc1	FH2 domain containing 1	6,0931	0,0652	1,6880
1434528_at	Aard	alanine and arginine rich domain containing protein	6,0925	0,0652	1,6319
1428871_at	NULL	NULL	6,0858	0,0652	1,6395
1431293_a_at	Cldnd1	claudin domain containing 1	6,0680	0,0652	2,2376
1438653_x_at	Atxn10	ataxin 10	6,0677	0,0652	1,4119
1421139_a_at	Zfp386	zinc finger protein 386 (Kruppel-like)	6,0620	0,0654	1,7768
1454628_at	Iffo1	intermediate filament family orphan 1	6,0560	0,0654	1,5194
1450102_a_at	NULL	NULL	6,0501	0,0654	1,9257
1433457_s_at	Grsf1	G-rich RNA sequence binding factor 1	6,0418	0,0654	2,4460
1417250_at	Rlim	ring finger protein, LIM domain interacting	6,0220	0,0655	1,5361
1451520_at	Spg20	spastic paraplegia 20, spartin (Troyer syndrome) homolog (human)	6,0177	0,0655	1,5527
1424861_at	NULL	NULL	5,9950	0,0656	1,2499
1421052_a_at	Sms	spermine synthase	5,9939	0,0656	1,1993
1425496_at	Abca3	ATP-binding cassette, sub-family A (ABC1), member 3	5,9844	0,0656	1,7330
1438999_a_at	Nfat5	nuclear factor of activated T-cells 5	5,9817	0,0656	2,0486
1437309_a_at	Rpa1	replication protein A1	5,9670	0,0658	1,7567
1416658_at	Frzb	frizzled-related protein	5,9436	0,0661	1,7172
1416079_a_at	Arpc1a	actin related protein 2/3 complex, subunit 1A	5,9361	0,0665	1,6568
1418175_at	Vdr	vitamin D receptor	5,9194	0,0666	1,7623
1417745_at	Cpn1	carboxypeptidase N, polypeptide 1	5,9175	0,0666	1,3672
1425350_a_at	Myef2	myelin basic protein expression factor 2, repressor	5,8936	0,0668	1,3509
1455195_at	Rps24	ribosomal protein S24	5,8770	0,0670	1,6234
1448944_at	Nrp1	neuropilin 1	5,8654	0,0670	1,2877
1416577_a_at	Rbx1	ring-box 1	5,8629	0,0670	1,2717
1415972_at	Marcks	myristoylated alanine rich protein kinase C substrate	5,8514	0,0673	2,5213
1423202_a_at	Ncor1	nuclear receptor co-repressor 1	5,8353	0,0679	1,7162
1427472_a_at	C8b	complement component 8, beta polypeptide	5,8138	0,0681	2,4066
1424039_at	Tmem66	transmembrane protein 66	5,7965	0,0681	1,6016
1450756_s_at	Cul3	cullin 3	5,7908	0,0681	1,7678
1460367_at	Hbp1	high mobility group box transcription factor 1	5,7627	0,0681	1,6542
1421833_at	Pip5k1b	phosphatidylinositol-4-phosphate 5-kinase, type 1 beta	5,7618	0,0681	1,2965
1453819_x_at	Stx18	syntaxin 18	5,7587	0,0681	1,4344
1424109_a_at	Glo1	glyoxalase 1	5,7503	0,0681	2,1459
1454635_at	NULL	NULL	5,7450	0,0681	1,4820
1423619_at	Rasd1	RAS, dexamethasone-induced 1	5,7403	0,0681	1,9599
1427087_at	Luc7l2	LUC7-like 2 (S. cerevisiae)	5,7207	0,0681	1,6780
1427887_at	Rprd1b	regulation of nuclear pre-mRNA domain containing 1B	5,6808	0,0683	1,3985
1419176_at	Vps37a	vacuolar protein sorting 37A (yeast)	5,6799	0,0683	3,2589
1460450_at	Ammecr1l	AMME chromosomal region gene 1-like	5,6742	0,0683	1,5802
1430527_a_at	Rnf167	ring finger protein 167	5,6705	0,0683	1,9302
1452694_at	Ip6k1	inositol hexaphosphate kinase 1	5,6640	0,0683	1,5430
1428063_at	Ankrd46	ankyrin repeat domain 46	5,6639	0,0683	1,3063
1456194_a_at	Park7	Parkinson disease (autosomal recessive, early onset) 7	5,6629	0,0683	1,3376
1460342_s_at	Mprip	myosin phosphatase Rho interacting protein	5,6469	0,0683	1,8560
1426453_at	Pitrm1	pitrilysin metallepetidase 1	5,6443	0,0683	1,5480
1427206_at	NULL	NULL	5,6361	0,0683	1,1642
1420810_at	1500003O03Rik	RIKEN cDNA 1500003O03 gene	5,6222	0,0687	1,5088
1436909_at	Slc25a44	solute carrier family 25, member 44	5,6187	0,0687	1,3826
1428108_x_at	Tmcc2	transmembrane and coiled-coil domains 2	5,6133	0,0687	1,3836
1423156_at	Gnpnat1	glucosamine-phosphate N-acetyltransferase 1	5,6117	0,0687	1,4083
1431844_at	Kcnmb2	potassium large conductance calcium-activated channel, subfamily M, beta member 2	5,6084	0,0687	1,7484
1417411_at	Nap1l5	nucleosome assembly protein 1-like 5	5,6068	0,0687	2,1772
1422576_at	Atxn10	ataxin 10	5,5994	0,0687	1,6897
1426216_at	Cog6	component of oligomeric golgi complex 6	5,5711	0,0690	1,5593
1448203_at	Atp5l	ATP synthase, H+ transporting, mitochondrial F0 complex, subunit g	5,5570	0,0690	1,5352
1427025_at	Mtmr7	myotubularin related protein 7	5,5556	0,0690	1,5415
1450755_at	Pafah1b2	platelet-activating factor acetylhydrolase, isoform 1b, subunit 2	5,5478	0,0690	1,6199
1423321_at	Myadm	myeloid-associated differentiation marker	5,5467	0,0690	1,5035
1448875_at	Zhx1	zinc fingers and homeoboxes 1	5,5401	0,0690	1,3938
1428237_at	NULL	NULL	5,5257	0,0690	1,6796
1418316_a_at	NULL	NULL	5,5182	0,0690	1,9949
1417442_a_at	Pex3	peroxisomal biogenesis factor 3	5,5178	0,0690	1,3056
1423413_at	Ndrg1	N-myc downstream regulated gene 1	5,5166	0,0690	1,7142
1424048_a_at	Cyb5r1	cytochrome b5 reductase 1	5,5143	0,0690	2,1948
1416769_s_at	Atp6v0b	ATPase, H+ transporting, lysosomal V0 subunit B	5,5018	0,0690	1,6776
1451240_a_at	Glo1	glyoxalase 1	5,5005	0,0690	3,9544
1419385_a_at	Ubqln1	ubiquilin 1	5,4983	0,0690	1,6116
1452249_at	Prickle1	prickle homolog 1 (Drosophila)	5,4916	0,0690	1,4228
1416724_x_at	Tcf4	transcription factor 4	5,4900	0,0690	1,5553

Probeset ID	Symbol	Gene Name	d-value	q-value	R-fold
1434311_at	Cnot6l	CCR4-NOT transcription complex, subunit 6-like	5,4885	0,0690	1,6408
1424188_at	Rabgap1	RAB GTPase activating protein 1	5,4846	0,0690	1,6602
1417866_at	Tnfaip1	tumor necrosis factor, alpha-induced protein 1 (endothelial)	5,4840	0,0690	1,5288
1418893_at	Pbx2	pre B-cell leukemia transcription factor 2	5,4826	0,0690	1,9952
1426824_at	Psme4	proteasome (prosome, macropain) activator subunit 4	5,4723	0,0690	1,6902
1416976_at	Stam2	signal transducing adaptor molecule (SH3 domain and ITAM motif) 2	5,4702	0,0690	1,2885
1431390_a_at	Grinl1a	glutamate receptor, ionotropic, N-methyl D-aspartate-like 1A	5,4681	0,0690	1,8384
1434745_at	Ccnd2	cyclin D2	5,4616	0,0690	2,0527
1425480_at	Cnot6l	CCR4-NOT transcription complex, subunit 6-like	5,4594	0,0690	1,4879
1419441_at	Rplp0	ribosomal protein, large, P0	5,4459	0,0691	1,3620
1460631_at	Ogt	O-linked N-acetylglucosamine (GlcNAc) transferase (UDP-N-acetylglucosamine:polypeptide-N-acetylglucosaminyl transferase)	5,4371	0,0692	2,3765
AFFX-GapdhMur/M32599_5_at	Gapdh	glyceraldehyde-3-phosphate dehydrogenase	5,4359	0,0692	1,6783
1419062_at	Epb4.1l3	erythrocyte protein band 4.1-like 3	5,4312	0,0692	1,6699
1417964_at	Ap3d1	adaptor-related protein complex 3, delta 1 subunit	5,4147	0,0692	1,7701
1448376_at	Wrnip1	Werner helicase interacting protein 1	5,4133	0,0692	1,4474
1428467_at	Tardbp	TAR DNA binding protein	5,4131	0,0692	1,4871
1448850_a_at	Dnajc5	DnaJ (Hsp40) homolog, subfamily C, member 5	5,3995	0,0692	1,7289
1423216_a_at	Fam32a	family with sequence similarity 32, member A	5,3978	0,0692	1,9772
1422449_s_at	Rcn2	reticulocalbin 2	5,3773	0,0692	2,1455
1452328_s_at	Pja2	praja 2, RING-H2 motif containing	5,3743	0,0692	1,7866
1448223_at	Fto	fat mass and obesity associated	5,3661	0,0692	1,4216
1426265_x_at	Dlat	dihydrolipoamide S-acetyltransferase (E2 component of pyruvate dehydrogenase complex)	5,3559	0,0692	1,6078
1418168_at	Zcchc14	zinc finger, CCHC domain containing 14	5,3504	0,0692	1,3271
1426556_at	Zfp280d	zinc finger protein 280D	5,3477	0,0692	1,8016
1419584_at	Ttc28	tetratricopeptide repeat domain 28	5,3453	0,0692	1,8042
1452161_at	Tiparp	TCDD-inducible poly(ADP-ribose) polymerase	5,3384	0,0692	1,3628
1417454_at	Cul4b	cullin 4B	5,3236	0,0692	2,0725
1423299_at	Txnl1	thioredoxin-like 1	5,2943	0,0692	2,0004
1454758_a_at	Tsc22d1	TSC22 domain family, member 1	5,2886	0,0692	1,5624
1417943_at	Gng4	guanine nucleotide binding protein (G protein), gamma 4	5,2862	0,0692	1,6092
1436266_x_at	Cbx1	chromobox homolog 1 (Drosophila HP1 beta)	5,2851	0,0692	1,2397
1448828_at	Smc6	structural maintenance of chromosomes 6	5,2830	0,0692	1,6339
1452866_at	Nars	asparaginyl-tRNA synthetase	5,2702	0,0692	1,5606
1425473_at	Med17	mediator complex subunit 17	5,2672	0,0692	1,3099
1418275_a_at	Elf2	E74-like factor 2	5,2637	0,0692	2,1684
1418979_at	Akr1c14	aldo-keto reductase family 1, member C14	5,2591	0,0692	2,6900
1434561_at	Asxl1	additional sex combs like 1 (Drosophila)	5,2554	0,0692	1,5803
1427136_s_at	Srsf12	serine/arginine-rich splicing factor 12	5,2541	0,0692	2,2316
1424847_at	Nefh	neurofilament, heavy polypeptide	5,2474	0,0692	1,2770
1448549_a_at	Dpagt1	dolichyl-phosphate (UDP-N-acetylglucosamine) acetylglucosaminephosphotransferase 1 (GlcNAc-1-P transferase)	5,2368	0,0692	1,4426
1418627_at	Gclm	glutamate-cysteine ligase, modifier subunit	5,2364	0,0692	1,4856
1426961_at	Phf20	PHD finger protein 20	5,2295	0,0692	1,4815
1426545_at	Tnrc6b	trinucleotide repeat containing 6b	5,2217	0,0692	1,3395
1452157_at	Eprs	glutamyl-prolyl-tRNA synthetase	5,2202	0,0692	1,8796
1421835_at	Mtap7	microtubule-associated protein 7	5,2083	0,0692	1,5660
1450634_at	Atp6v1a	ATPase, H+ transporting, lysosomal V1 subunit A	5,2029	0,0692	1,6060
1450769_s_at	Stard5	StAR-related lipid transfer (START) domain containing 5	5,2013	0,0692	1,4602
1436783_x_at	Ywhab	tyrosine 3-monooxygenase/tryptophan 5-monooxygenase activation protein, beta polypeptide	5,1919	0,0692	2,1646
1460689_at	Pppde2	PPPDE peptidase domain containing 2	5,1911	0,0692	1,9892
1460649_at	Irak1	interleukin-1 receptor-associated kinase 1	5,1804	0,0692	1,4978
1422795_at	Cul3	cullin 3	5,1726	0,0692	1,9102
1426160_a_at	Stk16	serine/threonine kinase 16	5,1720	0,0692	1,3513
1427134_at	Srsf12	serine/arginine-rich splicing factor 12	5,1702	0,0692	1,6913
1453474_at	Abhd15	abhydrolase domain containing 15	5,1668	0,0692	1,4714
1426776_at	Wasl	Wiskott-Aldrich syndrome-like (human)	5,1634	0,0692	1,6471
1448393_at	Cldn7	claudin 7	5,1572	0,0692	1,6254
1426946_at	Ipo5	importin 5	5,1487	0,0692	1,9834
1428675_at	NULL	NULL	5,1403	0,0692	1,4313
1423592_at	Rock2	Rho-associated coiled-coil containing protein kinase 2	5,1396	0,0692	1,2638
1423075_at	Lman2	lectin, mannose-binding 2	5,1334	0,0692	1,3913
1437738_at	NULL	NULL	5,1330	0,0692	2,1934
1448657_a_at	Dnajb2	DnaJ (Hsp40) homolog, subfamily B, member 2	5,1302	0,0692	1,6249
1424385_at	Gon4l	gon-4-like (C.elegans)	5,1295	0,0692	1,3264
1422508_at	Atp6v1a	ATPase, H+ transporting, lysosomal V1 subunit A	5,1237	0,0692	1,8314
1416229_at	Rfk	riboflavin kinase	5,1208	0,0692	1,6710
1421970_a_at	Gria2	glutamate receptor, ionotropic, AMPA2 (alpha 2)	5,1159	0,0692	2,1901
1426856_at	Hsdl2	hydroxysteroid dehydrogenase like 2	5,1110	0,0692	1,1729
1422456_at	Nsf	N-ethylmaleimide sensitive fusion protein	5,1104	0,0692	1,5751
1451096_at	Ndufs2	NADH dehydrogenase (ubiquinone) Fe-S protein 2	5,1047	0,0692	2,6436
1416387_at	Pip4k2c	phosphatidylinositol-5-phosphate 4-kinase, type II, gamma	5,1013	0,0692	1,6033
1426998_at	Zfand3	zinc finger, AN1-type domain 3	5,0948	0,0692	2,7542
1418587_at	Traf3	TNF receptor-associated factor 3	5,0935	0,0692	1,9105
1451723_at	Cnot6l	CCR4-NOT transcription complex, subunit 6-like	5,0912	0,0692	1,6436
1450746_at	Keap1	kelch-like ECH-associated protein 1	5,0889	0,0692	1,4920

Probeset ID	Symbol	Gene Name	d-value	q-value	R-fold
1454735_at	NULL	NULL	5,0835	0,0692	1,6138
1428648_at	Cand1	cullin associated and neddylation disassociated 1	5,0826	0,0692	1,6456
1451449_at	Erlec1	endoplasmic reticulum lectin 1	5,0810	0,0692	1,3652
1438045_at	Eea1	early endosome antigen 1	5,0716	0,0692	1,6667
1454142_a_at	Pwp1	PWP1 homolog (S. cerevisiae)	5,0679	0,0692	1,1653
1418883_a_at	Pabpc1	poly(A) binding protein, cytoplasmic 1	5,0679	0,0692	1,4872
1449336_a_at	Slk	STE20-like kinase (yeast)	5,0672	0,0692	1,4767
1423334_at	Ergic1	endoplasmic reticulum-golgi intermediate compartment (ERGIC) 1	5,0647	0,0692	1,9568
1452154_at	Iars	isoleucine-tRNA synthetase	5,0602	0,0692	2,1643
1434551_at	NULL	NULL	5,0529	0,0692	1,8376
1415885_at	Chgb	chromogranin B	5,0523	0,0692	1,5964
1427439_s_at	Prmt5	protein arginine N-methyltransferase 5	5,0434	0,0692	1,3938
1419516_at	Fam50a	family with sequence similarity 50, member A	5,0369	0,0692	1,6249
1452276_at	Smarcad1	SWI/SNF-related, matrix-associated actin-dependent regulator of chromatin, subfamily a, containing DEAD/H box 1	5,0359	0,0692	1,4973
1423144_at	Pik3ca	phosphatidylinositol 3-kinase, catalytic, alpha polypeptide	5,0354	0,0692	1,7863
1417124_at	Dstn	destrin	5,0340	0,0692	1,5153
1426516_a_at	Lpin1	lipin 1	5,0264	0,0692	1,4732
1427504_s_at	Srsf2	serine/arginine-rich splicing factor 2	5,0231	0,0692	1,5828
1460711_at	Dnajc21	DnaJ (Hsp40) homolog, subfamily C, member 21	5,0229	0,0692	2,0716
1424461_at	Dctn2	dynactin 2	5,0116	0,0692	1,3661
1449110_at	Rhob	ras homolog gene family, member B	4,9913	0,0692	1,5797
1452395_at	Med19	mediator of RNA polymerase II transcription, subunit 19 homolog (yeast)	4,9892	0,0692	1,2167
1448136_at	Enpp2	ectonucleotide pyrophosphatase/phosphodiesterase 2	4,9841	0,0692	2,1236
1416840_at	Mid1ip1	Mid1 interacting protein 1 (gastrulation specific G12-like (zebrafish))	4,9790	0,0692	1,5087
1418436_at	Stx7	syntaxin 7	4,9775	0,0692	1,3530
1417247_at	AI597479	expressed sequence AI597479	4,9706	0,0692	1,3986
1417037_at	Orc6l	origin recognition complex, subunit 6-like (S. cerevisiae)	4,9701	0,0692	1,1554
1428119_a_at	NULL	NULL	4,9666	0,0692	1,3301
1434784_s_at	Tmem106c	transmembrane protein 106C	4,9664	0,0692	1,4133
1428931_a_at	Parp6	poly (ADP-ribose) polymerase family, member 6	4,9663	0,0692	1,5903
1423200_at	Ncor1	nuclear receptor co-repressor 1	4,9649	0,0692	1,6060
1434986_a_at	Sec61a1	Sec61 alpha 1 subunit (S. cerevisiae)	4,9606	0,0692	1,4148
1423598_at	Atp8a1	ATPase, aminophospholipid transporter (APLT), class I, type 8A, member 1	4,9602	0,0692	1,1042
1417084_at	Eif4ebp2	eukaryotic translation initiation factor 4E binding protein 2	4,9601	0,0692	1,4546
1448116_at	Uba1	ubiquitin-like modifier activating enzyme 1	4,9596	0,0692	1,7788
1418139_at	Dcx	doublecortin	4,9586	0,0692	1,8797
1452438_s_at	NULL	NULL	4,9570	0,0692	1,4261
1448963_at	Nfyc	nuclear transcription factor-Y gamma	4,9560	0,0692	1,4893
1424076_at	Gdpd1	glycerophosphodiester phosphodiesterase domain containing 1	4,9504	0,0692	1,2257
1439017_x_at	Adipor1	adiponectin receptor 1	4,9498	0,0692	1,6138
1451393_at	Pex26	peroxisomal biogenesis factor 26	4,9379	0,0692	1,3783
1433521_at	Ankrd13c	ankyrin repeat domain 13c	4,9330	0,0692	1,8362
1454670_at	Rere	arginine glutamic acid dipeptide (RE) repeats	4,9320	0,0692	2,1827
1419089_at	Timp3	tissue inhibitor of metalloproteinase 3	4,9319	0,0692	1,5512
1426490_at	Bfar	bifunctional apoptosis regulator	4,9312	0,0692	1,7920
1450890_a_at	Abi1	abl-interactor 1	4,9273	0,0692	2,1286
1434034_at	NULL	NULL	4,9249	0,0692	1,9700
1431033_x_at	Agl	amylo-1,6-glucosidase, 4-alpha-glucanotransferase	4,9244	0,0692	1,7010
1431962_a_at	Stambp	STAM binding protein	4,9232	0,0692	1,3581
1419659_s_at	Chic2	cysteine-rich hydrophobic domain 2	4,9195	0,0692	1,5096
1426457_at	Slmap	sarcolemma associated protein	4,9189	0,0692	2,1132
1418288_at	Lpin1	lipin 1	4,9122	0,0692	1,6984
1435240_at	Baz2b	bromodomain adjacent to zinc finger domain, 2B	4,9105	0,0692	1,2427
1435946_at	Sepsecs	Sep (O-phosphoserine) tRNA:Sec (selenocysteine) tRNA synthase	4,9044	0,0693	1,4139
1437634_at	Thoc2	THO complex 2	4,8924	0,0693	1,7061
1424005_at	B230219D22Rik	RIKEN cDNA B230219D22 gene	4,8914	0,0693	1,5038
1423983_at	Sez6l2	seizure related 6 homolog like 2	4,8902	0,0693	1,7846
1419978_s_at	D10Ertd610e	DNA segment, Chr 10, ERATO Doi 610, expressed	4,8802	0,0696	1,2952
1426401_at	Ppp3ca	protein phosphatase 3, catalytic subunit, alpha isoform	4,8765	0,0696	2,1181
1448155_at	Pdcd6ip	programmed cell death 6 interacting protein	4,8757	0,0696	1,9375
1415946_at	Pigq	phosphatidylinositol glycan anchor biosynthesis, class Q	4,8748	0,0696	1,2832
1415840_at	Elovl5	ELOVL family member 5, elongation of long chain fatty acids (yeast)	4,8638	0,0696	1,9895
1446147_at	Rbm39	RNA binding motif protein 39	4,8638	0,0696	1,4536
1425516_at	Ogt	O-linked N-acetylglucosamine (GlcNAc) transferase (UDP-N-acetylglucosamine:polypeptide-N-acetylglucosaminyl transferase)	4,8615	0,0696	1,3485
1419159_at	Golga3	golgi autoantigen, golgin subfamily a, 3	4,8492	0,0696	1,1840
1437039_at	Cops2	COP9 (constitutive photomorphogenic) homolog, subunit 2 (Arabidopsis thaliana)	4,8429	0,0696	1,3317
1460168_at	Slbp	stem-loop binding protein	4,8388	0,0696	1,5931
1424391_at	Nrd1	nardilysin, N-arginine dibasic convertase, NRD convertase 1	4,8305	0,0696	1,8496
1424458_at	Kdm4c	lysine (K)-specific demethylase 4C	4,8248	0,0696	1,5891
1436510_a_at	Lrrfip2	leucine rich repeat (in FLII) interacting protein 2	4,8208	0,0696	1,7995
1449315_at	Odz3	odd Oz/ten-m homolog 3 (Drosophila)	4,8175	0,0696	1,7890
1423393_at	Clic4	chloride intracellular channel 4 (mitochondrial)	4,8134	0,0696	1,7659
1420897_at	Snap23	synaptosomal-associated protein 23	4,8132	0,0696	1,4681
1434998_at	Iqgap1	IQ motif containing GTPase activating protein 1	4,8057	0,0696	1,4780
1417965_at	Plekha1	pleckstrin homology domain containing, family A (phosphoinositide	4,8051	0,0696	1,5606

Probeset ID	Symbol	Gene Name	d-value	q-value	R-fold
		binding specific) member 1			
1444952_a_at	Nucks1	nuclear casein kinase and cyclin-dependent kinase substrate 1	4,8004	0,0696	2,1164
1424767_at	NULL	NULL	4,7976	0,0696	1,3034
1421487_a_at	Nck1	non-catalytic region of tyrosine kinase adaptor protein 1	4,7952	0,0696	1,6609
1452186_at	Rbm5	RNA binding motif protein 5	4,7763	0,0696	1,7670
1422174_at	Pdx1	pancreatic and duodenal homeobox 1	4,7745	0,0696	1,6600
1452066_a_at	Ndfip2	Nedd4 family interacting protein 2	4,7713	0,0696	1,7622
1426726_at	NULL	NULL	4,7683	0,0696	1,3897
1423982_at	Srsf10	serine/arginine-rich splicing factor 10	4,7671	0,0696	1,8491
1433574_at	Cdc37l1	cell division cycle 37 homolog (S. cerevisiae)-like 1	4,7628	0,0696	1,4865
1428380_at	0610007C21Rik	RIKEN cDNA 0610007C21 gene	4,7557	0,0696	1,3732
1422679_s_at	Ctr9	Ctr9, Paf1/RNA polymerase II complex component, homolog (S. cerevisiae)	4,7541	0,0696	1,2740
1427913_at	Rwdd1	RWD domain containing 1	4,7518	0,0696	1,4486
1426582_at	Atf2	activating transcription factor 2	4,7514	0,0696	2,7712
1419509_a_at	Nagk	N-acetylglucosamine kinase	4,7396	0,0696	1,3811
1455171_at	Suv420h1	suppressor of variegation 4-20 homolog 1 (Drosophila)	4,7381	0,0696	1,3553
1423212_at	Phc1	polyhomeotic-like 1 (Drosophila)	4,7378	0,0696	1,2997
1455815_a_at	Ywhab	tyrosine 3-monooxygenase/tryptophan 5-monooxygenase activation protein, beta polypeptide	4,7292	0,0696	2,1061
1424736_at	Eef2	eukaryotic translation elongation factor 2	4,7248	0,0696	1,4452
1417380_at	Iqgap1	IQ motif containing GTPase activating protein 1	4,7210	0,0696	2,6911
1423626_at	Dst	dystonin	4,7207	0,0696	2,0161
1433570_s_at	Naa35	N(alpha)-acetyltransferase 35, NatC auxiliary subunit	4,7162	0,0696	1,8578
1450037_at	Usp9x	ubiquitin specific peptidase 9, X chromosome	4,7138	0,0696	1,2172
1433951_at	Arl5a	ADP-ribosylation factor-like 5A	4,7119	0,0696	1,5344
1451003_at	Tab2	TGF-beta activated kinase 1/MAP3K7 binding protein 2	4,7107	0,0696	1,8770
1452377_at	Mll1	myeloid/lymphoid or mixed-lineage leukemia 1	4,7102	0,0696	2,2930
1417727_at	Srsf9	serine/arginine-rich splicing factor 9	4,7014	0,0696	1,5618
1452778_x_at	Nap1l1	nucleosome assembly protein 1-like 1	4,7010	0,0696	1,3800
1455066_s_at	Mia3	melanoma inhibitory activity 3	4,7002	0,0696	1,6223
1418564_s_at	Serbp1	serpine1 mRNA binding protein 1	4,6995	0,0696	1,2843
1425036_a_at	Tnrc6a	trinucleotide repeat containing 6a	4,6966	0,0696	1,8604
1425742_a_at	Tsc22d1	TSC22 domain family, member 1	4,6907	0,0696	1,4537
1452189_at	Wdr82	WD repeat domain containing 82	4,6898	0,0696	1,4976
1435133_at	Ugcg	UDP-glucose ceramide glucosyltransferase	4,6896	0,0696	1,3327
1427063_at	5330417C22Rik	RIKEN cDNA 5330417C22 gene	4,6865	0,0696	1,5440
1415689_s_at	Zkscan3	zinc finger with KRAB and SCAN domains 3	4,6826	0,0696	1,2446
1426015_s_at	Asph	aspartate-beta-hydroxylase	4,6758	0,0696	1,9052
1418087_at	Ufd1l	ubiquitin fusion degradation 1 like	4,6748	0,0696	1,6507
1437508_at	Sp4	trans-acting transcription factor 4	4,6715	0,0696	1,1964
1434340_at	NULL	NULL	4,6643	0,0696	1,6177
1426861_at	Aftph	aftiphilin	4,6612	0,0696	1,8326
1452105_a_at	Tsc2	tuberous sclerosis 2	4,6606	0,0696	1,5084
1450376_at	Mxi1	Max interacting protein 1	4,6603	0,0696	1,6664
1419428_a_at	Gaa	glucosidase, alpha, acid	4,6588	0,0696	1,5472
1433461_at	Sf3b2	splicing factor 3b, subunit 2	4,6509	0,0696	2,0274
1427245_at	Arfgap1	ADP-ribosylation factor GTPase activating protein 1	4,6476	0,0696	1,6402
1416831_at	Neu1	neuraminidase 1	4,6475	0,0696	1,8849
1425270_at	Kif1b	kinesin family member 1B	4,6466	0,0696	1,1942
1427088_at	Ccnt2	cyclin T2	4,6407	0,0696	1,3905
1436775_a_at	Ankrd17	ankyrin repeat domain 17	4,6399	0,0696	1,2870
1424402_at	Rufy3	RUN and FYVE domain containing 3	4,6394	0,0696	1,5247
1422794_at	Cul3	cullin 3	4,6363	0,0696	2,3567
1418257_at	Slc12a7	solute carrier family 12, member 7	4,6310	0,0696	1,6595
1448131_at	Mfn2	mitofusin 2	4,6310	0,0696	1,4218
1427441_a_at	Suclg2	succinate-Coenzyme A ligase, GDP-forming, beta subunit	4,6279	0,0696	1,4930
1451275_at	Uhrf1bp1l	UHRF1 (ICBP90) binding protein 1-like	4,6247	0,0696	1,9317
1435220_s_at	Cdc42se2	CDC42 small effector 2	4,6231	0,0696	1,3493
1416549_at	Slc35b4	solute carrier family 35, member B4	4,6195	0,0696	1,3213
1434336_s_at	Rcor1	REST corepressor 1	4,6133	0,0696	2,0652
1426510_at	NULL	NULL	4,6132	0,0696	1,2573
1417336_a_at	Sytl4	synaptotagmin-like 4	4,6122	0,0696	1,7642
1428714_at	Pgrmc2	progesterone receptor membrane component 2	4,6121	0,0696	1,4843
1415671_at	Atp6v0d1	ATPase, H+ transporting, lysosomal V0 subunit D1	4,6117	0,0696	1,5820
1436079_s_at	Vapb	vesicle-associated membrane protein, associated protein B and C	4,6105	0,0696	1,6247
1455913_x_at	Ttr	transthyretin	4,6086	0,0696	1,8735
1427889_at	Spna2	spectrin alpha 2	4,6075	0,0696	1,8941
1427876_at	Zc3h15	zinc finger CCCH-type containing 15	4,6075	0,0696	1,3192
1417090_at	Rcn1	reticulocalbin 1	4,6034	0,0696	2,0382
1422495_a_at	Hmgn1	high mobility group nucleosomal binding domain 1	4,5987	0,0696	1,4336
1434618_at	Crebzf	CREB/ATF bZIP transcription factor	4,5985	0,0696	1,3567
1423303_at	Paxip1	PAX interacting (with transcription-activation domain) protein 1	4,5894	0,0696	2,2109
1415769_at	Itch	itchy, E3 ubiquitin protein ligase	4,5874	0,0696	1,4804
1432827_x_at	Ubc	ubiquitin C	4,5866	0,0696	1,7923
1452235_at	Man1b1	mannosidase, alpha, class 1B, member 1	4,5779	0,0698	1,5295
1456083_x_at	Eif3c	eukaryotic translation initiation factor 3, subunit C	4,5764	0,0698	1,5083
1438192_s_at	Baz2a	bromodomain adjacent to zinc finger domain, 2A	4,5763	0,0698	1,6895
1418443_at	Xpo1	exportin 1, CRM1 homolog (yeast)	4,5738	0,0698	2,2552

Probeset ID	Symbol	Gene Name	d-value	q-value	R-fold
1415949_at	NULL	NULL	4,5731	0,0699	1,2715
1433546_at	Gns	glucosamine (N-acetyl)-6-sulfatase	4,5669	0,0700	1,4586
1434597_at	Larp4b	La ribonucleoprotein domain family, member 4B	4,5627	0,0700	1,3966
1451580_a_at	Ttr	transthyretin	4,5609	0,0700	1,8733
1428468_at	3110043O21Rik	RIKEN cDNA 3110043O21 gene	4,5607	0,0700	1,5253
1423707_at	Tmem50b	transmembrane protein 50B	4,5581	0,0700	1,4710
1437334_x_at	Parn	poly(A)-specific ribonuclease (deadenylation nuclease)	4,5555	0,0700	1,3195
1424455_at	Gprasp1	G protein-coupled receptor associated sorting protein 1	4,5544	0,0700	1,4823
1434877_at	Nptx1	neuronal pentraxin 1	4,5499	0,0702	1,3996
1454814_s_at	NULL	NULL	4,5487	0,0702	2,0273
1438006_at	4933439F18Rik	RIKEN cDNA 4933439F18 gene	4,5453	0,0702	1,5715
1454668_at	Ubr4	ubiquitin protein ligase E3 component n-recognin 4	4,5436	0,0702	2,0990
1428100_at	Srsf1	serine/arginine-rich splicing factor 1	4,5394	0,0702	1,5658
1433745_at	Trio	triple functional domain (PTPRF interacting)	4,5370	0,0702	2,2026
1415700_a_at	Ssr3	signal sequence receptor, gamma	4,5359	0,0702	1,3852
1426744_at	Srebf2	sterol regulatory element binding factor 2	4,5355	0,0702	1,6189
1419067_a_at	Rabgef1	RAB guanine nucleotide exchange factor (GEF) 1	4,5334	0,0702	1,2060
1423472_at	sep-02	septin 2	4,5316	0,0702	1,2658
1425462_at	Fbxw11	F-box and WD-40 domain protein 11	4,5247	0,0702	1,7304
1436898_at	Sfpq	splicing factor proline/glutamine rich (polypyrimidine tract binding protein associated)	4,5219	0,0702	2,7928
1429321_at	Rnf149	ring finger protein 149	4,5209	0,0702	1,4285
1451728_at	Wdr13	WD repeat domain 13	4,5186	0,0702	1,8931
1426518_at	Tubgcp5	tubulin, gamma complex associated protein 5	4,5157	0,0702	1,2434
1460350_at	Osbp	oxysterol binding protein	4,5140	0,0702	1,5586
1415825_s_at	NULL	NULL	4,5097	0,0702	1,8896
1448243_at	Napa	N-ethylmaleimide sensitive fusion protein attachment protein alpha	4,5067	0,0703	1,4773
1417908_s_at	Ube2l3	ubiquitin-conjugating enzyme E2L 3	4,5064	0,0703	1,2668
1428664_at	Vip	vasoactive intestinal polypeptide	4,5030	0,0703	2,2743
1419442_at	Matn2	matrilin 2	4,5014	0,0703	1,3057
1448884_at	Gtf2e2	general transcription factor II E, polypeptide 2 (beta subunit)	4,5005	0,0703	1,9566
1416648_at	Dync1h1	dynein cytoplasmic 1 heavy chain 1	4,4963	0,0703	1,6503
1436885_a_at	Cherp	calcium homeostasis endoplasmic reticulum protein	4,4956	0,0703	1,5084
1425518_at	Rapgef4	Rap guanine nucleotide exchange factor (GEF) 4	4,4937	0,0703	1,6230
1425315_at	Dock7	dedicator of cytokinesis 7	4,4933	0,0703	1,1512
1418032_at	Itfg2	integrin alpha FG-GAP repeat containing 2	4,4931	0,0703	1,6840
1431213_a_at	LOC67527	murine leukemia retrovirus	4,4925	0,0703	7,5538
1418234_s_at	Bcas2	breast carcinoma amplified sequence 2	4,4897	0,0703	1,3641
1456606_a_at	NULL	NULL	4,4890	0,0703	1,6790
1422064_a_at	Zbtb20	zinc finger and BTB domain containing 20	4,4774	0,0703	2,0428
1428128_at	Atxn7l3b	ataxin 7-like 3B	4,4686	0,0703	1,5594
1424015_at	Dennd5a	DENN/MADD domain containing 5A	4,4623	0,0703	1,5580
1416964_at	Eefsec	eukaryotic elongation factor, selenocysteine-tRNA-specific	4,4607	0,0703	1,1250
1418881_at	Necab2	N-terminal EF-hand calcium binding protein 2	4,4607	0,0703	1,2545
1452310_at	Tada2a	transcriptional adaptor 2A	4,4586	0,0703	1,3254
1437382_at	Acvr2a	activin receptor IIA	4,4527	0,0703	1,6264
1435742_at	Smek1	SMEK homolog 1, suppressor of mek1 (Dictyostelium)	4,4517	0,0703	1,6235
1422568_at	Ndel1	nuclear distribution gene E-like homolog 1 (A. nidulans)	4,4505	0,0703	1,7614
1436228_at	Zranb1	zinc finger, RAN-binding domain containing 1	4,4450	0,0703	2,0557
1436767_at	Luc7l2	LUC7-like 2 (S. cerevisiae)	4,4445	0,0703	1,6307
1428402_at	Zcchc3	zinc finger, CCHC domain containing 3	4,4434	0,0703	1,2846
1423502_at	Brd2	bromodomain containing 2	4,4417	0,0703	1,7770
1448224_at	Tfam	transcription factor A, mitochondrial	4,4386	0,0703	1,8753
1429370_a_at	Psmd11	proteasome (prosome, macropain) 26S subunit, non-ATPase, 11	4,4380	0,0703	2,1348
1438606_a_at	Clic4	chloride intracellular channel 4 (mitochondrial)	4,4364	0,0703	1,9258
1449122_at	Ubxn2b	UBX domain protein 2B	4,4325	0,0703	1,2747
1452787_a_at	Prmt1	protein arginine N-methyltransferase 1	4,4314	0,0703	1,5210
1428140_at	Oxct1	3-oxoacid CoA transferase 1	4,4298	0,0703	1,4246
1449552_at	Zfr	zinc finger RNA binding protein	4,4297	0,0703	2,5961
1451717_s_at	Senp2	SUMO/sentrin specific peptidase 2	4,4270	0,0703	1,2944
1424333_at	Rg9mtd1	RNA (guanine-9-) methyltransferase domain containing 1	4,4268	0,0703	1,3602
1437628_s_at	Rhoa	ras homolog gene family, member A	4,4239	0,0703	1,9496
1435018_at	5930434B04Rik	RIKEN cDNA 5930434B04 gene	4,4207	0,0703	1,3889
1417868_a_at	Ctsz	cathepsin Z	4,4198	0,0703	1,6145
1426761_at	Kdm1a	lysine (K)-specific demethylase 1A	4,4192	0,0703	2,0299
1448199_at	Ankrd10	ankyrin repeat domain 10	4,4169	0,0703	2,1867
1423916_s_at	NULL	NULL	4,4157	0,0703	2,1547
1420507_a_at	Srsf12ip1	serine/arginine-rich splicing factor 12, interacting protein 1	4,4136	0,0703	1,2085
1417665_a_at	Cpsf1	cleavage and polyadenylation specific factor 1	4,4116	0,0703	1,2862
1438931_s_at	NULL	NULL	4,4071	0,0703	1,5632
1460004_x_at	Stx6	syntaxin 6	4,4063	0,0703	1,9889
1416004_at	Ywhah	tyrosine 3-monooxygenase/tryptophan 5-monooxygenase activation protein, eta polypeptide	4,4053	0,0703	1,3471
1423819_s_at	Arl6ip1	ADP-ribosylation factor-like 6 interacting protein 1	4,3972	0,0703	1,9971
1437547_s_at	NULL	NULL	4,3961	0,0703	1,3742
1448324_at	Rnps1	ribonucleic acid binding protein S1	4,3911	0,0703	1,5123
1415869_a_at	Trim28	tripartite motif-containing 28	4,3883	0,0703	2,0350
1416138_at	Anxa7	annexin A7	4,3875	0,0703	2,1891
1437211_x_at	Elovl5	ELOVL family member 5, elongation of long chain fatty acids (yeast)	4,3851	0,0703	1,6196

Probeset ID	Symbol	Gene Name	d-value	q-value	R-fold
1426260_a_at	NULL	NULL	4,3833	0,0703	3,5799
1424662_at	Pmpca	peptidase (mitochondrial processing) alpha	4,3793	0,0703	1,9689
1426903_at	Fndc3a	fibronectin type III domain containing 3A	4,3783	0,0703	1,8484
1434403_at	Spred2	sprouty-related, EVH1 domain containing 2	4,3782	0,0703	1,5721
1450089_a_at	Srprb	signal recognition particle receptor, B subunit	4,3777	0,0703	1,7063
1452037_at	Mgat2	mannoside acetylglucosaminyltransferase 2	4,3769	0,0703	1,7828
1433430_s_at	Cdc23	CDC23 (cell division cycle 23, yeast, homolog)	4,3713	0,0705	1,6328
1416105_at	Nnt	nicotinamide nucleotide transhydrogenase	4,3661	0,0707	1,9053
1428172_at	Prpf39	PRP39 pre-mRNA processing factor 39 homolog (yeast)	4,3617	0,0709	1,7450
1449670_x_at	NULL	NULL	4,3586	0,0710	1,2214
1451676_at	Drap1	Dr1 associated protein 1 (negative cofactor 2 alpha)	4,3526	0,0712	1,5984
1424252_at	Hnrpdl	heterogeneous nuclear ribonucleoprotein D-like	4,3522	0,0712	1,6798
1434768_at	Tpp1	tripeptidyl peptidase I	4,3477	0,0713	1,4782
1428465_at	Tmem147	transmembrane protein 147	4,3442	0,0713	1,3618
1428847_a_at	Macf1	microtubule-actin crosslinking factor 1	4,3436	0,0713	1,8779
1453406_a_at	Rab28	RAB28, member RAS oncogene family	4,3383	0,0715	1,2631
1448548_at	Tulp4	tubby like protein 4	4,3308	0,0715	1,8768
1427078_at	Snx19	sorting nexin 19	4,3291	0,0715	1,2894
1456241_a_at	Slc38a10	solute carrier family 38, member 10	4,3258	0,0715	1,7283
1416785_at	Kcnip1	Kv channel-interacting protein 1	4,3201	0,0715	1,5138
1426402_at	Syncrip	synaptotagmin binding, cytoplasmic RNA interacting protein	4,3200	0,0715	1,9066
1456054_a_at	Pum1	pumilio 1 (Drosophila)	4,3160	0,0715	2,1655
1448706_at	Tdp2	tyrosyl-DNA phosphodiesterase 2	4,3147	0,0715	1,4384
1418500_at	Nap1l3	nucleosome assembly protein 1-like 3	4,3132	0,0715	1,4604
1422714_at	NULL	NULL	4,3097	0,0715	1,3103
1426342_at	Stt3b	STT3, subunit of the oligosaccharyltransferase complex, homolog B (S. cerevisiae)	4,3058	0,0715	1,6123
1427143_at	Kdm5b	lysine (K)-specific demethylase 5B	4,3054	0,0715	1,4828
1428187_at	Cd47	CD47 antigen (Rh-related antigen, integrin-associated signal transducer)	4,3052	0,0715	1,7886
1450877_at	BC005537	cDNA sequence BC005537	4,3022	0,0715	1,8467
1424586_at	Ehbp1	EH domain binding protein 1	4,2955	0,0718	1,7135
1423723_s_at	Tardbp	TAR DNA binding protein	4,2934	0,0719	2,2550
1448788_at	Cd200	CD200 antigen	4,2889	0,0720	1,4920
1426343_at	Stt3b	STT3, subunit of the oligosaccharyltransferase complex, homolog B (S. cerevisiae)	4,2781	0,0720	1,7343
1437465_a_at	P4hb	prolyl 4-hydroxylase, beta polypeptide	4,2764	0,0720	1,2526
1417364_at	NULL	NULL	4,2741	0,0720	1,3382
1416181_at	Mesdc2	mesoderm development candidate 2	4,2732	0,0720	1,6175
1426684_at	Cnot6	CCR4-NOT transcription complex, subunit 6	4,2711	0,0720	1,4152
1460429_at	Cdc5l	cell division cycle 5-like (S. pombe)	4,2695	0,0720	1,9816
1416719_a_at	Rps10	ribosomal protein S10	4,2591	0,0721	1,2639
1423382_a_at	NULL	NULL	4,2578	0,0721	1,6831
1421450_a_at	Map3k4	mitogen-activated protein kinase kinase kinase 4	4,2574	0,0721	1,2541
1423444_at	Rock1	Rho-associated coiled-coil containing protein kinase 1	4,2540	0,0722	2,3747
1422766_at	Stau1	staufen (RNA binding protein) homolog 1 (Drosophila)	4,2477	0,0723	1,4913
1423685_at	Aars	alanyl-tRNA synthetase	4,2460	0,0723	1,3913
1426826_at	Rbm16	RNA binding motif protein 16	4,2399	0,0723	1,8781
1434831_a_at	Foxo3	forkhead box O3	4,2373	0,0723	1,2969
1427148_at	Pja2	praja 2, RING-H2 motif containing	4,2359	0,0723	1,4673
1426804_at	Smarca4	SWI/SNF related, matrix associated, actin dependent regulator of chromatin, subfamily a, member 4	4,2355	0,0723	1,7909
1454602_s_at	Cnot2	CCR4-NOT transcription complex, subunit 2	4,2354	0,0723	1,4624
1451188_at	Wdr26	WD repeat domain 26	4,2312	0,0723	1,9788
1417743_at	Clk2	CDC-like kinase 2	4,2295	0,0723	1,4885
1423293_at	Rpa1	replication protein A1	4,2290	0,0723	1,5170
1417321_at	Zcchc7	zinc finger, CCHC domain containing 7	4,2197	0,0723	2,0975
1433689_s_at	Rps9	ribosomal protein S9	4,2167	0,0723	1,5293
1433830_at	Hnrnpa2b1	heterogeneous nuclear ribonucleoprotein A2/B1	4,2139	0,0723	2,0111
1424135_at	Rspry1	ring finger and SPRY domain containing 1	4,2128	0,0723	1,6527
1423113_a_at	Ube2d3	ubiquitin-conjugating enzyme E2D 3 (UBC4/5 homolog, yeast)	4,2085	0,0723	1,7879
1420023_at	Etf1	eukaryotic translation termination factor 1	4,2066	0,0723	1,0993
1433669_at	Akap8	A kinase (PRKA) anchor protein 8	4,2066	0,0723	1,7593
1460545_at	Thrap3	thyroid hormone receptor associated protein 3	4,2058	0,0723	1,4758
1430526_a_at	Smarca2	SWI/SNF related, matrix associated, actin dependent regulator of chromatin, subfamily a, member 2	4,2045	0,0723	2,1506
1427266_at	Pbrm1	polybromo 1	4,2037	0,0723	1,9244
1423058_at	Capza2	capping protein (actin filament) muscle Z-line, alpha 2	4,1933	0,0723	1,9601
1435178_x_at	Anapc5	anaphase-promoting complex subunit 5	4,1925	0,0723	1,5796
1454632_at	Tmem229b	transmembrane protein 229B	4,1890	0,0723	1,8725
1415890_at	Papss1	3'-phosphoadenosine 5'-phosphosulfate synthase 1	4,1864	0,0723	2,0370
1450711_at	Brd4	bromodomain containing 4	4,1818	0,0723	1,3264
1416462_at	Caprin1	cell cycle associated protein 1	4,1809	0,0723	1,5999
1420834_at	Vamp2	vesicle-associated membrane protein 2	4,1764	0,0723	1,5262
1452669_at	Fam53c	family with sequence similarity 53, member C	4,1755	0,0723	1,2275
1424028_at	Chtf8	CTF8, chromosome transmission fidelity factor 8 homolog (S. cerevisiae)	4,1739	0,0723	1,6442
1450024_at	Sufu	suppressor of fused homolog (Drosophila)	4,1725	0,0723	1,1969
1455491_at	Hnrnph3	heterogeneous nuclear ribonucleoprotein H3	4,1700	0,0723	1,7708
1433606_at	Dcp1a	DCP1 decapping enzyme homolog A (S. cerevisiae)	4,1680	0,0723	1,9352
1455631_at	H13	histocompatibility 13	4,1653	0,0723	1,1326

Probeset ID	Symbol	Gene Name	d-value	q-value	R-fold
1449437_at	D6Wsu163e	DNA segment, Chr 6, Wayne State University 163, expressed	4,1653	0,0723	1,6571
1417516_at	Ddit3	DNA-damage inducible transcript 3	4,1629	0,0723	1,5666
1448165_at	Casp2	caspase 2	4,1623	0,0723	1,2741
1425384_a_at	Ube4a	ubiquitination factor E4A, UFD2 homolog (S. cerevisiae)	4,1604	0,0723	1,1872
1453744_a_at	Ankrd40	ankyrin repeat domain 40	4,1580	0,0723	1,3959
1434025_at	NULL	NULL	4,1569	0,0723	1,6020
1454636_at	Cbx5	chromobox homolog 5 (Drosophila HP1a)	4,1569	0,0723	1,5612
1449273_at	Cyfip2	cytoplasmic FMR1 interacting protein 2	4,1532	0,0723	1,8505
1435177_a_at	Anapc5	anaphase-promoting complex subunit 5	4,1526	0,0723	1,6043
1451086_s_at	Rac1	RAS-related C3 botulinum substrate 1	4,1511	0,0723	1,8793
1417223_at	Cd2bp2	CD2 antigen (cytoplasmic tail) binding protein 2	4,1505	0,0723	1,3814
1450694_at	Fkbp2	FK506 binding protein 2	4,1504	0,0723	1,2724
1423974_at	Numa1	nuclear mitotic apparatus protein 1	4,1458	0,0723	1,4049
1440831_at	Bach1	BTB and CNC homology 1	4,1450	0,0723	1,8707
1418022_at	Naa15	N(alpha)-acetyltransferase 15, NatA auxiliary subunit	4,1418	0,0723	1,9421
1450995_at	Folr1	folate receptor 1 (adult)	4,1416	0,0723	1,5641
1422833_at	Foxa2	forkhead box A2	4,1355	0,0723	1,5986
1416205_at	Glb1	galactosidase, beta 1	4,1336	0,0723	2,0166
1418964_at	Pigm	phosphatidylinositol glycan anchor biosynthesis, class M	4,1314	0,0723	1,1889
1436165_at	Luc7l2	LUC7-like 2 (S. cerevisiae)	4,1312	0,0723	1,5300
1415702_a_at	NULL	NULL	4,1311	0,0723	1,5438
1455128_x_at	Tnrc6a	trinucleotide repeat containing 6a	4,1305	0,0723	2,4785
1451556_a_at	2700078E11Rik	RIKEN cDNA 2700078E11 gene	4,1247	0,0723	1,6029
1423948_at	Bag2	BCL2-associated athanogene 2	4,1241	0,0723	1,6416
1424091_at	Zfp868	zinc finger protein 868	4,1207	0,0723	1,3087
1420532_at	Magi2	membrane associated guanylate kinase, WW and PDZ domain containing 2	4,1175	0,0723	1,2585
1416559_at	Rrp8	ribosomal RNA processing 8, methyltransferase, homolog (yeast)	4,1171	0,0723	1,3168
1420711_a_at	Pxmp3	peroxisomal membrane protein 3	4,1164	0,0723	1,5596
1449048_s_at	Rab4a	RAB4A, member RAS oncogene family	4,1150	0,0723	1,8356
1448810_at	Gne	glucosamine	4,1094	0,0723	1,5729
1418109_at	Gspt2	G1 to S phase transition 2	4,1044	0,0723	1,3698
1460692_at	Ehmt2	euchromatic histone lysine N-methyltransferase 2	4,0928	0,0723	1,8590
1428125_at	Atxn7l3b	ataxin 7-like 3B	4,0918	0,0723	1,5725
1452036_a_at	Tmpo	thymopoietin	4,0916	0,0723	1,6857
1451214_at	Kbtbd2	kelch repeat and BTB (POZ) domain containing 2	4,0881	0,0723	1,5975
1448460_at	Acvr1	activin A receptor, type 1	4,0871	0,0723	1,8007
1426473_at	Dnajc9	DnaJ (Hsp40) homolog, subfamily C, member 9	4,0865	0,0723	1,5041
1451036_at	Spg21	spastic paraplegia 21 homolog (human)	4,0850	0,0723	1,8454
1448163_at	Gnpda1	glucosamine-6-phosphate deaminase 1	4,0848	0,0723	1,2327
1449343_s_at	Sin3a	transcriptional regulator, SIN3A (yeast)	4,0847	0,0723	1,4626
1428657_at	Rreb1	ras responsive element binding protein 1	4,0841	0,0723	1,3976
1426347_at	2010321M09Rik	RIKEN cDNA 2010321M09 gene	4,0840	0,0723	1,3846
1426519_at	P4ha1	procollagen-proline, 2-oxoglutarate 4-dioxygenase (proline 4-hydroxylase), alpha 1 polypeptide	4,0825	0,0723	1,4692
1423445_at	Rock1	Rho-associated coiled-coil containing protein kinase 1	4,0808	0,0723	1,6855
1460398_at	Phf8	PHD finger protein 8	4,0776	0,0723	1,5416
1450729_at	Hs2st1	heparan sulfate 2-O-sulfotransferase 1	4,0746	0,0723	1,6385
1460189_at	Dcaf11	DDB1 and CUL4 associated factor 11	4,0732	0,0723	1,8380
1417018_at	Efemp2	epidermal growth factor-containing fibulin-like extracellular matrix protein 2	4,0714	0,0723	1,1377
1418585_at	Ccnh	cyclin H	4,0684	0,0723	1,7306
1415961_at	Itm2c	integral membrane protein 2C	4,0671	0,0723	1,4709
1454711_at	Trio	triple functional domain (PTPRF interacting)	4,0637	0,0723	1,6234
1454725_at	Tra2a	transformer 2 alpha homolog (Drosophila)	4,0628	0,0723	1,6934
1455975_x_at	Rnf114	ring finger protein 114	4,0621	0,0723	1,5019
1433757_a_at	Nisch	nischarin	4,0621	0,0723	2,1932
1452064_at	Med23	mediator complex subunit 23	4,0620	0,0723	1,4598
1419392_at	Pclo	piccolo (presynaptic cytomatrix protein)	4,0617	0,0723	1,7366
1418128_at	Adcy6	adenylate cyclase 6	4,0593	0,0723	1,3203
1416395_at	Guk1	guanylate kinase 1	4,0592	0,0723	1,6427
1416277_a_at	Rplp1	ribosomal protein, large, P1	4,0588	0,0723	1,1920
1416005_at	Psmc1	protease (prosome, macropain) 26S subunit, ATPase 1	4,0564	0,0723	1,5954
1416372_at	NULL	NULL	4,0555	0,0723	2,1553
1453013_at	Zfp740	zinc finger protein 740	4,0549	0,0723	1,4750
1436871_at	Srsf7	serine/arginine-rich splicing factor 7	4,0516	0,0723	1,6174
1456040_at	Sf3b2	splicing factor 3b, subunit 2	4,0510	0,0723	1,4652
1426264_at	Dlat	dihydrolipoamide S-acetyltransferase (E2 component of pyruvate dehydrogenase complex)	4,0454	0,0723	1,4432
1448934_at	Ndufa10	NADH dehydrogenase (ubiquinone) 1 alpha subcomplex 10	4,0411	0,0723	1,4416
1423129_at	Shoc2	soc-2 (suppressor of clear) homolog (C. elegans)	4,0395	0,0723	1,2967
1449244_at	Cdh2	cadherin 2	4,0382	0,0723	1,9640
1416100_at	Eif3d	eukaryotic translation initiation factor 3, subunit D	4,0381	0,0723	1,6353
1448843_at	Ssr1	signal sequence receptor, alpha	4,0377	0,0723	1,2845
1423470_at	Ptbp2	polypyrimidine tract binding protein 2	4,0372	0,0723	1,9363
1454763_at	Ankrd17	ankyrin repeat domain 17	4,0367	0,0723	1,9735
1426832_at	Ddx26b	DEAD/H (Asp-Glu-Ala-Asp/His) box polypeptide 26B	4,0357	0,0723	1,6439
1454663_at	Eif5	eukaryotic translation initiation factor 5	4,0321	0,0723	2,2141
1460331_at	Tm9sf2	transmembrane 9 superfamily member 2	4,0320	0,0723	1,8080

Probeset ID	*Symbol*	*Gene Name*	*d-value*	*q-value*	*R-fold*
1437234_x_at	Prmt2	protein arginine N-methyltransferase 2	4,0319	0,0723	1,6047
1426207_at	Ikbkb	inhibitor of kappaB kinase beta	4,0308	0,0723	1,4089
1433758_at	Nisch	nischarin	4,0294	0,0723	1,3825
1425481_at	Cnot6l	CCR4-NOT transcription complex, subunit 6-like	4,0288	0,0723	1,4685
1426456_a_at	Pias2	protein inhibitor of activated STAT 2	4,0278	0,0723	1,8026
1416722_at	Hmg20a	high mobility group 20A	4,0266	0,0723	1,6365
1452093_at	Tmem185b	transmembrane protein 185B	4,0234	0,0723	1,3282
1417974_at	Kpna4	karyopherin (importin) alpha 4	4,0232	0,0723	1,5393
1427978_at	4732418C07Rik	RIKEN cDNA 4732418C07 gene	4,0230	0,0723	1,2779
1424876_s_at	Spg20	spastic paraplegia 20, spartin (Troyer syndrome) homolog (human)	4,0204	0,0723	1,1475
1424903_at	Kdm5d	lysine (K)-specific demethylase 5D	4,0186	0,0723	1,5321
1454971_x_at	Tsc22d1	TSC22 domain family, member 1	4,0183	0,0723	1,4336
1420631_a_at	Blcap	bladder cancer associated protein homolog (human)	4,0179	0,0723	1,3159
1434087_at	Mthfr	5,10-methylenetetrahydrofolate reductase	4,0144	0,0723	1,5901
1450998_at	Zfp110	zinc finger protein 110	4,0140	0,0723	1,3912
1448313_at	Tpp1	tripeptidyl peptidase I	4,0139	0,0723	1,4587
1451586_at	Tmbim6	transmembrane BAX inhibitor motif containing 6	4,0139	0,0723	1,4384
1449620_s_at	Adcy9	adenylate cyclase 9	4,0118	0,0723	1,4874
1423057_at	Capza2	capping protein (actin filament) muscle Z-line, alpha 2	4,0115	0,0723	1,5783
1422736_at	NULL	NULL	4,0101	0,0723	2,0368
1452664_a_at	Tm7sf3	transmembrane 7 superfamily member 3	4,0092	0,0723	1,4386
1416659_at	Eif3a	eukaryotic translation initiation factor 3, subunit A	4,0059	0,0723	1,5270
1418667_at	2410002O22Rik	RIKEN cDNA 2410002O22 gene	4,0015	0,0723	1,4556
1448797_at	Elk3	ELK3, member of ETS oncogene family	4,0010	0,0723	2,4903
1418258_s_at	NULL	NULL	3,9991	0,0723	1,5034
1435327_at	Lpgat1	lysophosphatidylglycerol acyltransferase 1	3,9966	0,0723	2,3254
1421847_at	Wsb2	WD repeat and SOCS box-containing 2	3,9959	0,0723	1,4687
1451339_at	Suox	sulfite oxidase	3,9951	0,0723	1,4494
1423671_at	Dner	delta/notch-like EGF-related receptor	3,9941	0,0723	1,6582
1452182_at	Galnt2	UDP-N-acetyl-alpha-D-galactosamine:polypeptide N-acetylgalactosaminyltransferase 2	3,9936	0,0723	1,3102
1422718_at	Ap3s2	adaptor-related protein complex 3, sigma 2 subunit	3,9911	0,0723	1,4640
1437398_a_at	Aldh9a1	aldehyde dehydrogenase 9, subfamily A1	3,9907	0,0723	1,7668
1450893_a_at	Ubap1	ubiquitin-associated protein 1	3,9888	0,0723	1,6423
1426439_at	Ddx3y	DEAD (Asp-Glu-Ala-Asp) box polypeptide 3, Y-linked	3,9880	0,0723	1,6063
1428374_at	Glce	glucuronyl C5-epimerase	3,9862	0,0723	1,7404
1431805_a_at	Rhpn2	rhophilin, Rho GTPase binding protein 2	3,9859	0,0723	1,6205
1434641_x_at	Sez6l2	seizure related 6 homolog like 2	3,9851	0,0723	1,8049
1417001_a_at	D4Wsu53e	DNA segment, Chr 4, Wayne State University 53, expressed	3,9845	0,0723	1,5046
1454887_at	Pak2	p21 protein (Cdc42/Rac)-activated kinase 2	3,9829	0,0723	1,4329
1434060_at	Herc1	hect (homologous to the E6-AP (UBE3A) carboxyl terminus) domain and RCC1 (CHC1)-like domain (RLD) 1	3,9824	0,0723	1,9705
1433908_a_at	Cttn	cortactin	3,9809	0,0723	1,6665
1452086_at	Trmt5	TRM5 tRNA methyltransferase 5 homolog (S. cerevisiae)	3,9808	0,0723	1,2013
1418294_at	Epb4.1l4b	erythrocyte protein band 4.1-like 4b	3,9772	0,0723	1,4005
1428853_at	Ptch1	patched homolog 1	3,9751	0,0723	2,0628
1449324_at	Ero1l	ERO1-like (S. cerevisiae)	3,9721	0,0723	1,5975
1421743_a_at	Pcbp2	poly(rC) binding protein 2	3,9702	0,0723	1,7377
1431232_a_at	Mga	MAX gene associated	3,9694	0,0723	1,8034
1424369_at	Psmf1	proteasome (prosome, macropain) inhibitor subunit 1	3,9688	0,0723	1,8611
1426437_s_at	Hdac3	histone deacetylase 3	3,9683	0,0723	1,3096
1416418_at	Gabarapl1	gamma-aminobutyric acid (GABA) A receptor-associated protein-like 1	3,9619	0,0723	1,9176
1420821_at	Sgpp1	sphingosine-1-phosphate phosphatase 1	3,9600	0,0723	1,4679
1425053_at	Isoc1	isochorismatase domain containing 1	3,9540	0,0723	1,5552
1437830_x_at	Zbed3	zinc finger, BED domain containing 3	3,9500	0,0723	1,3293
1426558_x_at	NULL	NULL	3,9457	0,0723	1,5679
1455611_at	Pias1	protein inhibitor of activated STAT 1	3,9426	0,0723	1,2286
1426482_at	Prkrir	protein-kinase, interferon-inducible double stranded RNA dependent inhibitor, repressor of (P58 repressor)	3,9420	0,0723	1,3868
1417437_at	Xrcc6	X-ray repair complementing defective repair in Chinese hamster cells 6	3,9410	0,0723	1,2409
1423664_at	Qdpr	quinoid dihydropteridine reductase	3,9403	0,0723	1,6651
1448838_at	Topors	topoisomerase I binding, arginine/serine-rich	3,9401	0,0723	1,3183
1452612_at	Rnf160	ring finger protein 160	3,9390	0,0723	1,3681
1426365_at	2810403A07Rik	RIKEN cDNA 2810403A07 gene	3,9374	0,0723	2,1987
1423533_a_at	Rhot1	ras homolog gene family, member T1	3,9359	0,0723	1,6695
1416829_at	Atp5b	ATP synthase, H+ transporting mitochondrial F1 complex, beta subunit	3,9356	0,0723	1,4097
1421399_at	Insm1	insulinoma-associated 1	3,9320	0,0723	2,1678
1415817_s_at	Cct7	chaperonin containing Tcp1, subunit 7 (eta)	3,9304	0,0723	1,7354
1429359_s_at	Rbpms	RNA binding protein gene with multiple splicing	3,9260	0,0723	1,7444
1427232_at	Tshz1	teashirt zinc finger family member 1	3,9248	0,0723	1,8796
1417440_at	Arid1a	AT rich interactive domain 1A (SWI-like)	3,9230	0,0723	1,9708
1416556_at	Tspan31	tetraspanin 31	3,9223	0,0723	1,4561
1417607_at	Cox6a2	cytochrome c oxidase, subunit VI a, polypeptide 2	3,9179	0,0723	1,5182
1426254_at	Tm2d1	TM2 domain containing 1	3,9169	0,0723	1,4520
1448305_at	Rab6	RAB6, member RAS oncogene family	3,9125	0,0723	2,6007
1416393_at	Emg1	EMG1 nucleolar protein homolog (S. cerevisiae)	3,9122	0,0723	1,3004
1448621_a_at	Smpd1	sphingomyelin phosphodiesterase 1, acid lysosomal	3,9119	0,0723	1,7224
1439411_a_at	Xpo7	exportin 7	3,9116	0,0723	1,9775
1423750_a_at	Sf1	splicing factor 1	3,9109	0,0723	2,3298

Probeset ID	Symbol	Gene Name	d-value	q-value	R-fold
1418698_a_at	Fech	ferrochelatase	3,9080	0,0723	1,6219
1449515_at	Zfp292	zinc finger protein 292	3,9058	0,0723	1,5548
1454657_s_at	Naa35	N(alpha)-acetyltransferase 35, NatC auxiliary subunit	3,9051	0,0723	1,1609
1424567_at	Tspan2	tetraspanin 2	3,9039	0,0723	1,9250
1415740_at	Psmc5	protease (prosome, macropain) 26S subunit, ATPase 5	3,9019	0,0723	1,4683
1421858_at	Adam17	a disintegrin and metallopeptidase domain 17	3,9011	0,0723	1,3176
1456042_s_at	Cramp1l	Crm, cramped-like (Drosophila)	3,9002	0,0723	1,5762
1450388_s_at	Twsg1	twisted gastrulation homolog 1 (Drosophila)	3,8999	0,0723	1,8169
1427310_at	Bptf	bromodomain PHD finger transcription factor	3,8964	0,0723	2,2496
1424559_at	Rpap2	RNA polymerase II associated protein 2	3,8962	0,0723	1,6330
1415758_at	Fryl	furry homolog-like (Drosophila)	3,8951	0,0723	1,8888
1426348_at	Col4a1	collagen, type IV, alpha 1	3,8928	0,0723	1,6350
1415937_s_at	Pdcd6ip	programmed cell death 6 interacting protein	3,8912	0,0723	1,3276
1418274_at	NULL	NULL	3,8882	0,0723	1,3688
1423180_at	Kcnb1	potassium voltage gated channel, Shab-related subfamily, member 1	3,8880	0,0723	1,6361
1429265_a_at	Rnf130	ring finger protein 130	3,8867	0,0723	1,6498
1433803_at	Jak1	Janus kinase 1	3,8821	0,0723	1,3979
1438480_a_at	Thyn1	thymocyte nuclear protein 1	3,8819	0,0723	1,4152
1422648_at	Slc7a2	solute carrier family 7 (cationic amino acid transporter, y+ system), member 2	3,8785	0,0723	1,5660
1426289_at	Qrich1	glutamine-rich 1	3,8751	0,0723	1,8714
1424294_at	Ppp4r1	protein phosphatase 4, regulatory subunit 1	3,8738	0,0723	2,1487
1415982_at	Herpud2	HERPUD family member 2	3,8725	0,0723	1,6083
1455956_x_at	Ccnd2	cyclin D2	3,8719	0,0723	1,5326
1460541_at	Slc7a6	solute carrier family 7 (cationic amino acid transporter, y+ system), member 6	3,8718	0,0723	1,2149
1450028_a_at	NULL	NULL	3,8686	0,0723	1,3092
1449001_at	Ivd	isovaleryl coenzyme A dehydrogenase	3,8652	0,0723	1,6741
1451654_x_at	Zfp825	zinc finger protein 825	3,8650	0,0723	1,0905
1423098_at	Capn7	calpain 7	3,8648	0,0723	1,5826
1453804_a_at	Orc4l	origin recognition complex, subunit 4-like (S. cerevisiae)	3,8630	0,0723	1,6327
1424478_at	Bbs2	Bardet-Biedl syndrome 2 (human)	3,8613	0,0723	1,3600
1428134_at	Coq9	coenzyme Q9 homolog (yeast)	3,8607	0,0723	1,7676
1455683_a_at	Tbc1d8	TBC1 domain family, member 8	3,8595	0,0723	1,0734
1417407_at	Fbxl14	F-box and leucine-rich repeat protein 14	3,8588	0,0723	1,4514
1437730_at	Ppp2r2a	protein phosphatase 2 (formerly 2A), regulatory subunit B (PR 52), alpha isoform	3,8544	0,0723	1,3308
1424255_at	Supt5h	suppressor of Ty 5 homolog (S. cerevisiae)	3,8540	0,0723	1,6570
1438430_at	Hbp1	high mobility group box transcription factor 1	3,8534	0,0723	1,6523
1425628_a_at	Gtf2i	general transcription factor II I	3,8512	0,0723	1,3896
1437708_x_at	Vamp3	vesicle-associated membrane protein 3	3,8478	0,0724	2,7899
1438234_at	Wdr26	WD repeat domain 26	3,8460	0,0724	1,3893
1418017_at	Pum2	pumilio 2 (Drosophila)	3,8454	0,0724	2,3398
1424693_at	Erlec1	endoplasmic reticulum lectin 1	3,8409	0,0724	1,2864
1437148_at	Arpc2	actin related protein 2/3 complex, subunit 2	3,8381	0,0724	1,3451
1452884_at	Srsf2ip	serine/arginine-rich splicing factor 2, interacting protein	3,8371	0,0725	1,7971
1416438_at	Puf60	poly-U binding splicing factor 60	3,8358	0,0725	1,6445
1456386_at	NULL	NULL	3,8304	0,0725	2,1756
1426585_s_at	Mapk1	mitogen-activated protein kinase 1	3,8284	0,0725	2,0022
1451356_at	Anp32e	acidic (leucine-rich) nuclear phosphoprotein 32 family, member E	3,8282	0,0725	1,6194
1448725_at	Parg	poly (ADP-ribose) glycohydrolase	3,8280	0,0725	1,4679
1425927_a_at	Atf5	activating transcription factor 5	3,8252	0,0725	1,8768
1438169_a_at	Frmd4b	FERM domain containing 4B	3,8242	0,0725	2,0747
1455826_a_at	Bace1	beta-site APP cleaving enzyme 1	3,8183	0,0725	2,1107
1455915_at	Galnt4	UDP-N-acetyl-alpha-D-galactosamine:polypeptide N-acetylgalactosaminyltransferase 4	3,8176	0,0725	2,0552
1428049_a_at	Nudt16l1	nudix (nucleoside diphosphate linked moiety X)-type motif 16-like 1	3,8146	0,0725	1,4198
1427125_s_at	Lrrc41	leucine rich repeat containing 41	3,8145	0,0725	1,5908
1450843_a_at	Serpinh1	serine (or cysteine) peptidase inhibitor, clade H, member 1	3,8139	0,0725	1,3925
1419803_s_at	Ccdc12	coiled-coil domain containing 12	3,8105	0,0725	1,2986
1415787_at	Ganab	alpha glucosidase 2 alpha neutral subunit	3,8092	0,0725	1,7520
1449080_at	Hdac2	histone deacetylase 2	3,8085	0,0725	1,8716
1423792_a_at	Cmtm6	CKLF-like MARVEL transmembrane domain containing 6	3,8083	0,0725	1,6825
1418815_at	Cdh2	cadherin 2	3,8082	0,0725	2,2364
1437188_at	Gabbr1	gamma-aminobutyric acid (GABA) B receptor, 1	3,8074	0,0725	1,5046
1434705_at	Ctbp2	C-terminal binding protein 2	3,8021	0,0725	1,5938
1423364_a_at	Aktip	thymoma viral proto-oncogene 1 interacting protein	3,8019	0,0725	1,5560
1422510_at	Ctdspl	CTD (carboxy-terminal domain, RNA polymerase II, polypeptide A) small phosphatase-like	3,8002	0,0725	1,6027
1415785_a_at	Cct8	chaperonin containing Tcp1, subunit 8 (theta)	3,8002	0,0725	1,7751
1451090_a_at	Eif2s3x	eukaryotic translation initiation factor 2, subunit 3, structural gene X-linked	3,7998	0,0725	1,6707
1423898_a_at	Trip12	thyroid hormone receptor interactor 12	3,7980	0,0725	1,5686
1434515_at	Ncoa1	nuclear receptor coactivator 1	3,7958	0,0725	1,7064
1437289_at	Impad1	inositol monophosphatase domain containing 1	3,7956	0,0725	1,6766
1416922_a_at	Bnip3l	BCL2/adenovirus E1B interacting protein 3-like	3,7947	0,0725	1,6119
1419063_at	Ugt8a	UDP galactosyltransferase 8A	3,7938	0,0725	1,1142
1428907_at	Rbm25	RNA binding motif protein 25	3,7922	0,0726	1,8983
1426750_at	Flnb	filamin, beta	3,7874	0,0727	1,8459

Probeset ID	*Symbol*	*Gene Name*	*d-value*	*q-value*	*R-fold*
1424645_at	Tnrc6c	trinucleotide repeat containing 6C	3,7869	0,0727	1,2423
1452584_at	1500032L24Rik	RIKEN cDNA 1500032L24 gene	3,7841	0,0727	1,1918
1433605_at	Inpp5a	inositol polyphosphate-5-phosphatase A	3,7824	0,0727	1,2806
1433604_x_at	Aldoa	aldolase A, fructose-bisphosphate	3,7819	0,0727	1,9554
1416094_at	Adam9	a disintegrin and metallopeptidase domain 9 (meltrin gamma)	3,7796	0,0727	2,1085
1418968_at	Rb1cc1	RB1-inducible coiled-coil 1	3,7760	0,0728	1,9622
1451534_at	Scgn	secretagogin, EF-hand calcium binding protein	3,7759	0,0728	1,2666
1419249_at	Cdk14	cyclin-dependent kinase 14	3,7746	0,0728	1,8632
1417686_at	Lgals12	lectin, galactose binding, soluble 12	3,7744	0,0728	1,1906
1426978_at	Klhl2	kelch-like 2, Mayven (Drosophila)	3,7672	0,0729	1,3672
1420831_at	Qsox1	quiescin Q6 sulfhydryl oxidase 1	3,7652	0,0730	1,4188
1451459_at	Ahctf1	AT hook containing transcription factor 1	3,7642	0,0730	1,3546
1417924_at	Pak3	p21 protein (Cdc42/Rac)-activated kinase 3	3,7635	0,0730	1,4718
1417191_at	Dnajb9	DnaJ (Hsp40) homolog, subfamily B, member 9	3,7601	0,0731	1,6126
1434821_at	Brd1	bromodomain containing 1	3,7591	0,0731	1,3306
1425503_at	Gcnt2	glucosaminyl (N-acetyl) transferase 2, I-branching enzyme	3,7587	0,0731	2,1939
1450650_at	Myo10	myosin X	3,7583	0,0731	1,6863
1427903_at	Phpt1	phosphohistidine phosphatase 1	3,7578	0,0731	1,6735
1421819_a_at	Set	SET translocation	3,7565	0,0731	1,7544
1451244_a_at	Zfp422	zinc finger protein 422	3,7537	0,0732	1,4112
1448873_at	Ocln	occludin	3,7532	0,0732	1,2751
1426881_at	Ube3c	ubiquitin protein ligase E3C	3,7515	0,0732	1,4427
1423384_s_at	Tex261	testis expressed gene 261	3,7507	0,0732	1,3869
1435056_x_at	Pofut2	protein O-fucosyltransferase 2	3,7484	0,0733	1,7845
1418502_a_at	Oxr1	oxidation resistance 1	3,7459	0,0733	2,2067
1431057_a_at	Prss23	protease, serine, 23	3,7442	0,0734	1,3195
1460303_at	Nr3c1	nuclear receptor subfamily 3, group C, member 1	3,7427	0,0736	1,8472
1448709_at	Arid1a	AT rich interactive domain 1A (SWI-like)	3,7422	0,0736	1,5934
1451474_a_at	Parp8	poly (ADP-ribose) polymerase family, member 8	3,7420	0,0736	1,6734
1416506_at	Psma6	proteasome (prosome, macropain) subunit, alpha type 6	3,7395	0,0738	1,4058
1418024_at	Naa15	N(alpha)-acetyltransferase 15, NatA auxiliary subunit	3,7393	0,0738	1,5120
1416162_at	Rad21	RAD21 homolog (S. pombe)	3,7391	0,0738	1,6715
1416315_at	Abhd4	abhydrolase domain containing 4	3,7371	0,0738	1,4135
1421529_a_at	Txnrd1	thioredoxin reductase 1	3,7361	0,0738	1,6496
1424532_at	Ylpm1	YLP motif containing 1	3,7355	0,0738	1,6302
1415692_s_at	Canx	calnexin	3,7305	0,0738	1,4235
1418195_at	Galnt10	UDP-N-acetyl-alpha-D-galactosamine:polypeptide N-acetylgalactosaminyltransferase 10	3,7301	0,0738	1,4898
1460344_at	NULL	NULL	3,7287	0,0738	1,3233
1452241_at	Topbp1	topoisomerase (DNA) II binding protein 1	3,7274	0,0739	1,3866
1418176_at	Vdr	vitamin D receptor	3,7261	0,0739	1,4977
1460411_s_at	Pkdcc	protein kinase domain containing, cytoplasmic	3,7251	0,0739	1,6084
1431037_a_at	Elavl1	ELAV (embryonic lethal, abnormal vision, Drosophila)-like 1 (Hu antigen R)	3,7232	0,0740	1,5167
1431032_at	Agl	amylo-1,6-glucosidase, 4-alpha-glucanotransferase	3,7231	0,0740	1,5961
1423617_at	Cog8	component of oligomeric golgi complex 8	3,7219	0,0740	1,2485
1426483_at	Prkrir	protein-kinase, interferon-inducible double stranded RNA dependent inhibitor, repressor of (P58 repressor)	3,7206	0,0740	1,2323
1420630_at	8430419L09Rik	RIKEN cDNA 8430419L09 gene	3,7152	0,0740	1,1243
1426643_at	Elp3	elongation protein 3 homolog (S. cerevisiae)	3,7147	0,0740	2,0673
1435951_at	Grip1	glutamate receptor interacting protein 1	3,7139	0,0740	1,3743
1450626_at	Manba	mannosidase, beta A, lysosomal	3,7121	0,0740	1,8435
1455765_a_at	Abcc8	ATP-binding cassette, sub-family C (CFTR/MRP), member 8	3,7120	0,0740	1,6391
1419811_at	Adcy9	adenylate cyclase 9	3,7097	0,0740	2,1426
1425580_a_at	Pik3c3	phosphoinositide-3-kinase, class 3	3,7097	0,0740	1,3771
1454639_x_at	Rpl41	ribosomal protein L41	3,7082	0,0740	1,2275
1437585_x_at	Zfp161	zinc finger protein 161	3,7076	0,0740	1,7150
1453412_a_at	Sec14l1	SEC14-like 1 (S. cerevisiae)	3,7059	0,0740	1,4006
1423488_at	Mmd	monocyte to macrophage differentiation-associated	3,7058	0,0740	1,2594
1426915_at	Dapk1	death associated protein kinase 1	3,7045	0,0740	1,4156
1417480_at	Fbxo9	f-box protein 9	3,7025	0,0740	1,5379
1423457_at	Slc35a5	solute carrier family 35, member A5	3,7015	0,0740	1,1685
1448103_s_at	Nono	non-POU-domain-containing, octamer binding protein	3,7001	0,0740	1,2576
1421129_a_at	Atp2a3	ATPase, Ca++ transporting, ubiquitous	3,6979	0,0740	1,5649
1428113_at	Tmtc4	transmembrane and tetratricopeptide repeat containing 4	3,6958	0,0740	1,5669
1451739_at	Klf5	Kruppel-like factor 5	3,6924	0,0740	1,1878
1450382_at	Nf2	neurofibromatosis 2	3,6911	0,0740	1,3472
1428845_at	Bclaf1	BCL2-associated transcription factor 1	3,6903	0,0740	2,1906
1452290_at	Tmem106b	transmembrane protein 106B	3,6887	0,0740	1,2747
1423644_at	Aco1	aconitase 1	3,6875	0,0740	1,4569
1416251_at	Mcm6	minichromosome maintenance deficient 6 (MIS5 homolog, S. pombe) (S. cerevisiae)	3,6859	0,0740	1,5866
1431822_a_at	Azi2	5-azacytidine induced gene 2	3,6848	0,0740	1,5636
1434436_at	Morc4	microrchidia 4	3,6816	0,0740	1,2628
1450938_at	Pnn	pinin	3,6814	0,0740	1,6557
1428272_at	Eif1b	eukaryotic translation initiation factor 1B	3,6813	0,0740	1,4333
1448221_at	Bat1a	HLA-B-associated transcript 1A	3,6796	0,0740	1,9061
1425577_at	Zmym5	zinc finger, MYM-type 5	3,6786	0,0740	1,7395
1451273_x_at	Ftsjd1	FtsJ methyltransferase domain containing 1	3,6777	0,0740	1,2267

Probeset ID	Symbol	Gene Name	d-value	q-value	R-fold
1452342_at	Apbb2	amyloid beta (A4) precursor protein-binding, family B, member 2	3,6773	0,0740	1,9368
1434177_at	Ece1	endothelin converting enzyme 1	3,6739	0,0740	1,7980
1424094_at	Nek9	NIMA (never in mitosis gene a)-related expressed kinase 9	3,6732	0,0740	1,6114
1427670_a_at	Tcf12	transcription factor 12	3,6727	0,0740	1,9110
1434620_s_at	Fam13b	family with sequence similarity 13, member B	3,6720	0,0740	2,0996
1423508_at	Myst4	MYST histone acetyltransferase monocytic leukemia 4	3,6696	0,0740	1,3381
1424410_at	Ttc8	tetratricopeptide repeat domain 8	3,6686	0,0740	2,0594
1434306_at	Rab3ip	RAB3A interacting protein	3,6658	0,0740	1,2704
1422838_at	Kcnu1	potassium channel, subfamily U, member 1	3,6632	0,0740	1,2722
1435643_x_at	Ubb	ubiquitin B	3,6604	0,0740	1,2324
1427174_at	NULL	NULL	3,6583	0,0740	1,4899
1454074_a_at	Rsrc2	arginine/serine-rich coiled-coil 2	3,6571	0,0740	1,6263
1451127_at	Vopp1	vesicular, overexpressed in cancer, prosurvival protein 1	3,6571	0,0740	1,6851
1418983_at	Inadl	InaD-like (Drosophila)	3,6569	0,0740	1,4959
1455655_a_at	Tardbp	TAR DNA binding protein	3,6568	0,0740	1,6727
1421546_a_at	Racgap1	Rac GTPase-activating protein 1	3,6560	0,0740	1,1106
1425952_a_at	Gcg	glucagon	3,6560	0,0740	1,2232
1438723_a_at	Rps10	ribosomal protein S10	3,6545	0,0740	1,4701
1451684_a_at	Bicd1	bicaudal D homolog 1 (Drosophila)	3,6510	0,0740	1,2118
1416108_a_at	Tmed3	transmembrane emp24 domain containing 3	3,6504	0,0740	1,4615
1427185_at	Mef2a	myocyte enhancer factor 2A	3,6483	0,0740	1,6354
1426685_a_at	Cnot6	CCR4-NOT transcription complex, subunit 6	3,6461	0,0740	2,4798
1448422_at	Tmed4	transmembrane emp24 protein transport domain containing 4	3,6452	0,0740	1,5941
1423275_at	Ints6	integrator complex subunit 6	3,6431	0,0740	1,7692
1423038_at	Stx6	syntaxin 6	3,6425	0,0740	1,4617
1454794_at	Spast	spastin	3,6401	0,0740	1,4761
1460460_a_at	Gorasp2	golgi reassembly stacking protein 2	3,6396	0,0740	1,7045
1434704_at	Mll5	myeloid/lymphoid or mixed-lineage leukemia 5	3,6393	0,0740	2,4291
1435714_x_at	NULL	NULL	3,6375	0,0740	1,1293
1426798_a_at	Ppp1r15b	protein phosphatase 1, regulatory (inhibitor) subunit 15b	3,6373	0,0740	1,8602
1452083_a_at	Pja1	praja1, RING-H2 motif containing	3,6358	0,0740	1,4209
1417568_at	Ncald	neurocalcin delta	3,6330	0,0740	1,6174
1448592_at	Crtap	cartilage associated protein	3,6320	0,0740	1,5515
1416248_at	Nadk	NAD kinase	3,6300	0,0740	1,1905
1425615_a_at	Pck2	phosphoenolpyruvate carboxykinase 2 (mitochondrial)	3,6290	0,0740	1,3338
1452894_at	Elavl4	ELAV (embryonic lethal, abnormal vision, Drosophila)-like 4 (Hu antigen D)	3,6282	0,0740	1,5906
1451469_at	Cntln	centlein, centrosomal protein	3,6280	0,0740	1,1813
1434016_at	Znrf2	zinc and ring finger 2	3,6277	0,0740	1,5989
1422147_a_at	Pla2g6	phospholipase A2, group VI	3,6240	0,0740	1,6987
1418479_at	Vps54	vacuolar protein sorting 54 (yeast)	3,6235	0,0740	1,7877
1431056_a_at	Lpl	lipoprotein lipase	3,6213	0,0740	1,3794
1417767_at	Cyb5b	cytochrome b5 type B	3,6212	0,0740	1,6180
1415948_at	Creg1	cellular repressor of E1A-stimulated genes 1	3,6193	0,0740	1,5252
1450950_at	Smc3	structural maintenace of chromosomes 3	3,6188	0,0740	1,8823
1417647_at	Snx5	sorting nexin 5	3,6178	0,0740	1,3492
1433806_x_at	Calr	calreticulin	3,6117	0,0740	1,4508
1420814_at	Gdi2	guanosine diphosphate (GDP) dissociation inhibitor 2	3,6093	0,0740	1,8022
1428091_at	Klhl7	kelch-like 7 (Drosophila)	3,6090	0,0740	1,7763
1417114_at	Gmcl1	germ cell-less homolog 1 (Drosophila)	3,6087	0,0740	1,5419
1452828_at	Fbxo21	F-box protein 21	3,6078	0,0740	1,1708
1438289_a_at	Sumo1	SMT3 suppressor of mif two 3 homolog 1 (yeast)	3,6076	0,0740	1,6689
1417501_at	Fbxo6	F-box protein 6	3,6071	0,0740	1,5271
1433736_at	Hcfc1	host cell factor C1	3,6047	0,0740	1,3329
1417872_at	Fhl1	four and a half LIM domains 1	3,6042	0,0740	1,9682
1424650_at	Pdia5	protein disulfide isomerase associated 5	3,6039	0,0740	1,2884
1417462_at	Cap1	CAP, adenylate cyclase-associated protein 1 (yeast)	3,6031	0,0740	1,6223
1426979_at	Mlxip	MLX interacting protein	3,6024	0,0740	1,8920
1415831_at	Psmd2	proteasome (prosome, macropain) 26S subunit, non-ATPase, 2	3,6023	0,0740	1,7030
1449444_a_at	NULL	NULL	3,6022	0,0740	1,5440
1452204_at	Anks1	ankyrin repeat and SAM domain containing 1	3,6020	0,0740	1,2612
1448106_at	Necap1	NECAP endocytosis associated 1	3,6018	0,0740	1,4834
1452094_at	P4ha1	procollagen-proline, 2-oxoglutarate 4-dioxygenase (proline 4-hydroxylase), alpha 1 polypeptide	3,6009	0,0740	1,4503
1431375_s_at	Parva	parvin, alpha	3,6005	0,0740	2,0680
1416389_a_at	Rcbtb2	regulator of chromosome condensation (RCC1) and BTB (POZ) domain containing protein 2	3,6000	0,0740	1,3515
1422502_at	Parp1	poly (ADP-ribose) polymerase family, member 1	3,5984	0,0740	1,2721
1424539_at	Ubl4	ubiquitin-like 4	3,5982	0,0740	1,6592
1417622_at	Slc12a2	solute carrier family 12, member 2	3,5971	0,0740	1,5170
1422676_at	Smarce1	SWI/SNF related, matrix associated, actin dependent regulator of chromatin, subfamily e, member 1	3,5964	0,0740	1,2441
1423149_at	Skp1a	S-phase kinase-associated protein 1A	3,5963	0,0740	1,7841
1436479_a_at	Dpp7	dipeptidylpeptidase 7	3,5904	0,0740	2,0774
1434149_at	Tcf4	transcription factor 4	3,5902	0,0740	1,3124
1423864_at	Nrbp1	nuclear receptor binding protein 1	3,5898	0,0740	1,3681
1424081_at	Pcgf6	polycomb group ring finger 6	3,5895	0,0740	1,4478
1448208_at	Smad1	MAD homolog 1 (Drosophila)	3,5891	0,0740	2,5884
1452648_at	Tbrg1	transforming growth factor beta regulated gene 1	3,5873	0,0740	1,9289

Probeset ID	Symbol	Gene Name	d-value	q-value	R-fold
1425460_at	Mtmr2	myotubularin related protein 2	3,5871	0,0740	1,6656
1448379_at	Pot1a	protection of telomeres 1A	3,5866	0,0740	1,2233
1451056_at	Psmd7	proteasome (prosome, macropain) 26S subunit, non-ATPase, 7	3,5863	0,0740	1,3125
1438083_at	Hhip	Hedgehog-interacting protein	3,5858	0,0740	1,1172
1420150_at	Spsb1	splA/ryanodine receptor domain and SOCS box containing 1	3,5834	0,0740	1,5197
1426011_a_at	Ggnbp2	gametogenetin binding protein 2	3,5829	0,0740	1,9157
1434612_s_at	Sbno1	sno, strawberry notch homolog 1 (Drosophila)	3,5827	0,0740	1,5729
1419077_at	Mpp3	membrane protein, palmitoylated 3 (MAGUK p55 subfamily member 3)	3,5813	0,0740	2,0536
1428171_at	Prpf39	PRP39 pre-mRNA processing factor 39 homolog (yeast)	3,5812	0,0740	1,3571
1418850_at	Epc1	enhancer of polycomb homolog 1 (Drosophila)	3,5807	0,0740	1,4933
1426298_at	NULL	NULL	3,5796	0,0740	1,2695
1426788_a_at	Ssrp1	structure specific recognition protein 1	3,5795	0,0740	1,4321
1417704_a_at	Arhgap6	Rho GTPase activating protein 6	3,5793	0,0740	1,1854
1452292_at	Ap2b1	adaptor-related protein complex 2, beta 1 subunit	3,5769	0,0740	1,2904
1423495_at	Decr2	2-4-dienoyl-Coenzyme A reductase 2, peroxisomal	3,5763	0,0740	1,3564
1424641_a_at	Thoc1	THO complex 1	3,5743	0,0740	1,3966
1418896_a_at	Rpn2	ribophorin II	3,5726	0,0740	1,7030
1451638_s_at	NULL	NULL	3,5692	0,0740	1,9731
1452255_at	Fbxo38	F-box protein 38	3,5686	0,0740	1,1368
AFFX-GapdhMur/M32599_3_at	Gapdh	glyceraldehyde-3-phosphate dehydrogenase	3,5677	0,0740	1,2895
1438578_a_at	NULL	NULL	3,5670	0,0740	1,8577
1420476_a_at	NULL	NULL	3,5663	0,0740	1,6949
1417005_at	Klc1	kinesin light chain 1	3,5662	0,0740	1,3439
1426047_a_at	Ptprr	protein tyrosine phosphatase, receptor type, R	3,5652	0,0740	1,2675
1450937_at	Lin7c	lin-7 homolog C (C. elegans)	3,5651	0,0740	2,0902
1434465_x_at	Vldlr	very low density lipoprotein receptor	3,5648	0,0740	1,2864
1426969_at	Trim23	tripartite motif-containing 23	3,5631	0,0740	1,5443
1417324_at	Mast2	microtubule associated serine/threonine kinase 2	3,5625	0,0740	1,4608
1428908_at	Rbm25	RNA binding motif protein 25	3,5623	0,0740	1,5505
1416445_at	Fam98a	family with sequence similarity 98, member A	3,5617	0,0740	2,0472
1451779_at	Pyroxd1	pyridine nucleotide-disulphide oxidoreductase domain 1	3,5606	0,0740	1,2714
1423248_at	Nktr	natural killer tumor recognition sequence	3,5587	0,0740	1,7573
1415703_at	Huwe1	HECT, UBA and WWE domain containing 1	3,5585	0,0740	1,9021
1416186_at	Pnrc2	proline-rich nuclear receptor coactivator 2	3,5572	0,0740	1,4205
1450112_a_at	Gas2	growth arrest specific 2	3,5570	0,0740	1,3227
1448702_at	Ier3ip1	immediate early response 3 interacting protein 1	3,5561	0,0740	1,3432
1433995_s_at	Ccdc50	coiled-coil domain containing 50	3,5553	0,0740	1,5431
1450026_a_at	B3gnt2	UDP-GlcNAc:betaGal beta-1,3-N-acetylglucosaminyltransferase 2	3,5551	0,0740	1,7691
1423777_at	Usp20	ubiquitin specific peptidase 20	3,5541	0,0741	1,6631
1448312_at	Pcsk2	proprotein convertase subtilisin/kexin type 2	3,5523	0,0741	1,4715
1460352_s_at	Pik3r4	phosphatidylinositol 3 kinase, regulatory subunit, polypeptide 4, p150	3,5516	0,0741	1,9617
1417927_at	Ddx19a	DEAD (Asp-Glu-Ala-Asp) box polypeptide 19a	3,5502	0,0742	2,0072
1437354_at	NULL	NULL	3,5490	0,0743	1,4588
1423250_a_at	Tgfb2	transforming growth factor, beta 2	3,5473	0,0743	1,1251
1427000_at	Hnf4a	hepatic nuclear factor 4, alpha	3,5416	0,0745	1,2254
1423295_at	Tm9sf2	transmembrane 9 superfamily member 2	3,5408	0,0745	1,4335
1426723_at	Wdr48	WD repeat domain 48	3,5385	0,0745	1,3095
1416161_at	Rad21	RAD21 homolog (S. pombe)	3,5382	0,0745	1,5842
1421140_a_at	Foxp1	forkhead box P1	3,5364	0,0745	1,2653
1451081_a_at	Tcf25	transcription factor 25 (basic helix-loop-helix)	3,5353	0,0745	1,4562
1437845_x_at	Pofut2	protein O-fucosyltransferase 2	3,5329	0,0745	1,8173
1426880_at	Etl4	enhancer trap locus 4	3,5327	0,0745	1,9178
1417907_at	Ube2l3	ubiquitin-conjugating enzyme E2L 3	3,5305	0,0745	1,3570
1456005_a_at	Bcl2l11	BCL2-like 11 (apoptosis facilitator)	3,5294	0,0745	1,6720
1448967_at	Nipsnap3b	nipsnap homolog 3B (C. elegans)	3,5286	0,0745	1,5021
1423335_at	1110004F10Rik	RIKEN cDNA 1110004F10 gene	3,5279	0,0745	1,5099
1419917_s_at	Tmed7	transmembrane emp24 protein transport domain containing 7	3,5271	0,0745	1,9391
1433784_at	Zfp871	zinc finger protein 871	3,5251	0,0745	1,9715
1455058_at	Mtmr9	myotubularin related protein 9	3,5251	0,0745	1,3646
1440201_at	Slc8a1	solute carrier family 8 (sodium/calcium exchanger), member 1	3,5240	0,0745	1,8998
1420494_x_at	NULL	NULL	3,5239	0,0745	1,8814
1426351_at	Hspd1	heat shock protein 1 (chaperonin)	3,5231	0,0745	1,7096
1425974_a_at	Trim25	tripartite motif-containing 25	3,5229	0,0745	2,3689
1416256_a_at	Tubb5	tubulin, beta 5	3,5228	0,0745	1,2694
1416792_at	Ppm1g	protein phosphatase 1G (formerly 2C), magnesium-dependent, gamma isoform	3,5222	0,0745	1,3418
1419587_s_at	Rp2h	retinitis pigmentosa 2 homolog (human)	3,5214	0,0745	1,1298
1434038_at	Dnajc13	DnaJ (Hsp40) homolog, subfamily C, member 13	3,5213	0,0745	1,9464
1415688_at	Ube2g1	ubiquitin-conjugating enzyme E2G 1 (UBC7 homolog, C. elegans)	3,5176	0,0745	2,2077
1423672_at	Ttc30b	tetratricopeptide repeat domain 30B	3,5174	0,0745	1,7189
1452000_s_at	Sars	seryl-aminoacyl-tRNA synthetase	3,5171	0,0745	1,4475
1451974_at	Osbpl2	oxysterol binding protein-like 2	3,5166	0,0745	1,4857
1418434_at	Mkrn1	makorin, ring finger protein, 1	3,5153	0,0745	1,5098
1426451_at	NULL	NULL	3,5152	0,0745	1,2797
1422993_s_at	NULL	NULL	3,5140	0,0745	1,8992
1451577_at	Zbtb20	zinc finger and BTB domain containing 20	3,5139	0,0745	1,7522
1423622_a_at	Ccnl1	cyclin L1	3,5133	0,0745	1,6039

Probeset ID	Symbol	Gene Name	d-value	q-value	R-fold
1426985_s_at	Fam76b	family with sequence similarity 76, member B	3,5133	0,0745	1,7245
1460740_at	NULL	NULL	3,5122	0,0745	1,9050
1433613_at	Pank3	pantothenate kinase 3	3,5122	0,0745	1,5927
1449138_at	Sf3b1	splicing factor 3b, subunit 1	3,5121	0,0745	1,8150
1427244_at	Ttc15	tetratricopeptide repeat domain 15	3,5116	0,0745	1,2319
1423961_at	Wdr26	WD repeat domain 26	3,5095	0,0745	1,5159
1416532_at	Trrap	transformation/transcription domain-associated protein	3,5087	0,0745	1,9797
1450848_at	Dap3	death associated protein 3	3,5068	0,0745	1,4708
1422698_s_at	Jarid2	jumonji, AT rich interactive domain 2	3,5068	0,0745	1,3467
1433711_s_at	NULL	NULL	3,5062	0,0745	1,5588
1423599_a_at	Pdcl	phosducin-like	3,5060	0,0745	1,3417
1424842_a_at	Arhgap24	Rho GTPase activating protein 24	3,5052	0,0745	1,4891
1452203_at	Obfc2a	oligonucleotide/oligosaccharide-binding fold containing 2A	3,5030	0,0745	1,3689
1417972_s_at	Pop5	processing of precursor 5, ribonuclease P/MRP family (S. cerevisiae)	3,5023	0,0745	1,4916
1452176_at	Nup153	nucleoporin 153	3,5020	0,0745	1,6208
1454652_at	Zranb2	zinc finger, RAN-binding domain containing 2	3,5002	0,0746	1,2278
1434757_at	Cbfa2t2	core-binding factor, runt domain, alpha subunit 2, translocated to, 2 (human)	3,4975	0,0746	1,4828
1425051_at	Isoc1	isochorismatase domain containing 1	3,4958	0,0746	1,7966
1434273_at	Fam174b	family with sequence similarity 174, member B	3,4950	0,0746	1,4115
1427991_s_at	Usp45	ubiquitin specific petidase 45	3,4935	0,0747	1,2490
1460211_a_at	Kdelr1	KDEL (Lys-Asp-Glu-Leu) endoplasmic reticulum protein retention receptor 1	3,4913	0,0747	1,4718
1423760_at	Cd44	CD44 antigen	3,4912	0,0747	1,7142
1423680_at	Fads1	fatty acid desaturase 1	3,4907	0,0747	1,5591
1420592_a_at	Anp32e	acidic (leucine-rich) nuclear phosphoprotein 32 family, member E	3,4907	0,0747	1,2150
1424315_at	1110004E09Rik	RIKEN cDNA 1110004E09 gene	3,4878	0,0747	1,2349
1421733_a_at	Tpst1	protein-tyrosine sulfotransferase 1	3,4857	0,0748	1,4244
1418408_at	Zfand1	zinc finger, AN1-type domain 1	3,4857	0,0748	1,0571
1434674_at	Lyst	lysosomal trafficking regulator	3,4834	0,0748	1,3269
1437984_x_at	Bat1a	HLA-B-associated transcript 1A	3,4820	0,0748	1,5983
1427226_at	Epn2	epsin 2	3,4818	0,0748	1,4732
1426864_a_at	Ncam1	neural cell adhesion molecule 1	3,4767	0,0749	1,4861
1450054_at	Add1	adducin 1 (alpha)	3,4741	0,0749	1,3300
1450734_at	Sec16b	SEC16 homolog B (S. cerevisiae)	3,4735	0,0749	2,1851
1434377_x_at	NULL	NULL	3,4726	0,0749	1,2018
1436213_a_at	1110028C15Rik	RIKEN cDNA 1110028C15 gene	3,4721	0,0749	1,1858
1415723_at	Eif5	eukaryotic translation initiation factor 5	3,4710	0,0749	1,9337
1417740_at	Cdc37l1	cell division cycle 37 homolog (S. cerevisiae)-like 1	3,4707	0,0749	1,2647
1434549_at	Rab11a	RAB11a, member RAS oncogene family	3,4697	0,0749	1,3660
1416673_at	Bace2	beta-site APP-cleaving enzyme 2	3,4688	0,0749	1,3793
1421023_at	Pik3c2a	phosphatidylinositol 3-kinase, C2 domain containing, alpha polypeptide	3,4669	0,0749	1,4736
1427604_a_at	Atp9a	ATPase, class II, type 9A	3,4668	0,0749	1,5354
1437175_at	Pdik1l	PDLIM1 interacting kinase 1 like	3,4664	0,0749	1,6661
1416489_at	Pi4k2b	phosphatidylinositol 4-kinase type 2 beta	3,4655	0,0749	1,1568
1419273_at	C80913	expressed sequence C80913	3,4646	0,0749	1,8887
1451574_at	Bcl9	B-cell CLL/lymphoma 9	3,4620	0,0749	1,7293
1434036_at	Mtss1	metastasis suppressor 1	3,4613	0,0749	1,9084
1416488_at	Ccng2	cyclin G2	3,4603	0,0749	1,4355
1448829_at	Smc6	structural maintenance of chromosomes 6	3,4600	0,0749	1,4045
1449614_s_at	AI314976	expressed sequence AI314976	3,4581	0,0749	1,1759
1456405_at	Dido1	death inducer-obliterator 1	3,4576	0,0749	1,3219
1451130_at	Use1	unconventional SNARE in the ER 1 homolog (S. cerevisiae)	3,4569	0,0749	1,6054
1416207_at	Taz	tafazzin	3,4568	0,0749	1,4264
1417308_at	Pkm2	pyruvate kinase, muscle	3,4564	0,0749	1,5487
1424674_at	Slc39a6	solute carrier family 39 (metal ion transporter), member 6	3,4560	0,0749	1,8887
1416007_at	Satb1	special AT-rich sequence binding protein 1	3,4547	0,0750	2,0879
1452197_at	Smc4	structural maintenance of chromosomes 4	3,4529	0,0750	2,3908
1427406_at	Trip11	thyroid hormone receptor interactor 11	3,4529	0,0750	1,4910
1421448_at	Ralgapa1	Ral GTPase activating protein, alpha subunit 1	3,4508	0,0750	1,4051
1427875_a_at	NULL	NULL	3,4491	0,0750	1,3434
1437358_at	Wdfy1	WD repeat and FYVE domain containing 1	3,4444	0,0750	1,5569
1424663_at	BC017647	cDNA sequence BC017647	3,4440	0,0750	1,7473
1451740_at	Paip1	polyadenylate binding protein-interacting protein 1	3,4429	0,0750	1,6819
1452660_s_at	Klhl7	kelch-like 7 (Drosophila)	3,4425	0,0750	1,3609
1419753_at	Nfx1	nuclear transcription factor, X-box binding 1	3,4422	0,0750	1,2457
1423552_at	Leprotl1	leptin receptor overlapping transcript-like 1	3,4414	0,0750	1,6655
1426463_at	Gphn	gephyrin	3,4410	0,0750	1,8134
1453111_a_at	Slc25a39	solute carrier family 25, member 39	3,4398	0,0750	1,3129
1452718_at	Ubr5	ubiquitin protein ligase E3 component n-recognin 5	3,4393	0,0750	1,4380
1433639_at	Fam117a	family with sequence similarity 117, memberA	3,4390	0,0750	1,7741
1418629_a_at	Khdrbs1	KH domain containing, RNA binding, signal transduction associated 1	3,4382	0,0750	1,5034
1421893_a_at	Tpp2	tripeptidyl peptidase II	3,4380	0,0750	1,1490
1418433_at	Cab39	calcium binding protein 39	3,4365	0,0750	1,8559
1450052_at	Kif2a	kinesin family member 2A	3,4326	0,0751	1,3942
1455819_at	Rod1	ROD1 regulator of differentiation 1 (S. pombe)	3,4293	0,0751	1,7907
1424824_at	Slain1	SLAIN motif family, member 1	3,4273	0,0751	1,5537
1417560_at	Sfxn1	sideroflexin 1	3,4245	0,0752	1,2843
1426765_at	NULL	NULL	3,4228	0,0752	1,4591

Probeset ID	*Symbol*	*Gene Name*	*d-value*	*q-value*	*R-fold*
1423670_a_at	Srpr	signal recognition particle receptor ('docking protein')	3,4216	0,0752	1,3101
1415834_at	Dusp6	dual specificity phosphatase 6	3,4207	0,0752	1,5067
1425114_at	Rbbp6	retinoblastoma binding protein 6	3,4206	0,0752	1,6535
1450724_at	Fam126a	family with sequence similarity 126, member A	3,4199	0,0752	1,5097
1424124_at	Mospd2	motile sperm domain containing 2	3,4197	0,0752	1,6271
1456695_x_at	Anapc5	anaphase-promoting complex subunit 5	3,4161	0,0753	1,4326
1433855_at	Abat	4-aminobutyrate aminotransferase	3,4142	0,0753	1,8633
1456388_at	Atp11a	ATPase, class VI, type 11A	3,4114	0,0753	1,9079
1434325_x_at	Prkar1b	protein kinase, cAMP dependent regulatory, type I beta	3,4114	0,0753	1,8088
1419382_a_at	Dhrs4	dehydrogenase/reductase (SDR family) member 4	3,4080	0,0753	1,2632
1424431_at	Csgalnact2	chondroitin sulfate N-acetylgalactosaminyltransferase 2	3,4080	0,0753	1,5508
1454837_at	Cln6	ceroid-lipofuscinosis, neuronal 6	3,4078	0,0753	1,4954
1424801_at	Enah	enabled homolog (Drosophila)	3,4075	0,0753	1,3995
1437035_x_at	Rnf14	ring finger protein 14	3,4070	0,0754	1,3766
1419867_a_at	NULL	NULL	3,4054	0,0754	1,5631
1420535_a_at	Nub1	negative regulator of ubiquitin-like proteins 1	3,4019	0,0754	1,1898
1420479_a_at	Nap1l1	nucleosome assembly protein 1-like 1	3,4018	0,0754	1,9837
1416285_at	Ndufc1	NADH dehydrogenase (ubiquinone) 1, subcomplex unknown, 1	3,4008	0,0754	1,2326
1433514_at	Etnk1	ethanolamine kinase 1	3,4004	0,0754	1,8993
1448418_s_at	Dcaf11	DDB1 and CUL4 associated factor 11	3,4000	0,0755	1,2523
1433476_at	C78339	expressed sequence C78339	3,3996	0,0755	1,4996
1417465_at	NULL	NULL	3,3989	0,0755	1,3601
1427235_at	Kdm6a	4lysine (K)-specific demethylase 6A	3,3975	0,0755	1,4112
1450632_at	NULL	NULL	3,3973	0,0755	1,7528
1427200_at	Zranb1	zinc finger, RAN-binding domain containing 1	3,3953	0,0755	1,7217
1423677_at	Fkbp9	FK506 binding protein 9	3,3933	0,0755	1,5660
1448685_at	2900010M23Rik	RIKEN cDNA 2900010M23 gene	3,3933	0,0755	1,3387
1448144_at	Hnrnpab	heterogeneous nuclear ribonucleoprotein A/B	3,3929	0,0755	1,8717
1425330_a_at	NULL	NULL	3,3926	0,0755	2,0949
1452095_a_at	H47	histocompatibility 47	3,3926	0,0755	1,4548
1448552_s_at	Tmem206	transmembrane protein 206	3,3919	0,0755	1,4953
1437236_a_at	Zfp110	zinc finger protein 110	3,3912	0,0755	2,0551
1415870_at	Calu	calumenin	3,3909	0,0755	1,3255
1415914_at	Hnrnpab	heterogeneous nuclear ribonucleoprotein A/B	3,3906	0,0755	1,4019
1438233_at	Fam178a	family with sequence similarity 178, member A	3,3903	0,0755	1,9595
1417967_at	Mms19	MMS19 (MET18 S. cerevisiae)	3,3900	0,0755	1,3227
1423331_a_at	NULL	NULL	3,3886	0,0755	1,9092
1421867_at	Nr3c1	nuclear receptor subfamily 3, group C, member 1	3,3882	0,0755	1,5301
1434045_at	Cdkn1b	cyclin-dependent kinase inhibitor 1B	3,3874	0,0755	1,7394
1452008_at	Ttc39b	tetratricopeptide repeat domain 39B	3,3871	0,0755	1,6747
1426724_at	NULL	NULL	3,3859	0,0755	1,3306
1451979_at	Kras	v-Ki-ras2 Kirsten rat sarcoma viral oncogene homolog	3,3844	0,0755	2,8304
1418965_at	Nosip	nitric oxide synthase interacting protein	3,3839	0,0755	1,3205
1424609_a_at	NULL	NULL	3,3839	0,0755	2,0031
1426756_at	Galnt2	UDP-N-acetyl-alpha-D-galactosamine:polypeptide N-acetylgalactosaminyltransferase 2	3,3837	0,0755	1,5183
1421998_at	Tor3a	torsin family 3, member A	3,3828	0,0755	1,5349
1429246_a_at	Anxa6	annexin A6	3,3821	0,0755	1,7823
1417349_at	Pldn	pallidin	3,3817	0,0755	1,2787
1426974_at	Os9	amplified in osteosarcoma	3,3816	0,0755	1,3242
1452373_at	NULL	NULL	3,3814	0,0755	1,4555
1416534_at	Dpf2	D4, zinc and double PHD fingers family 2	3,3798	0,0755	1,5230
1448101_s_at	Trim27	tripartite motif-containing 27	3,3784	0,0755	1,5844
1419101_at	Sin3a	transcriptional regulator, SIN3A (yeast)	3,3782	0,0755	1,3943
1456097_a_at	Itgb3bp	integrin beta 3 binding protein (beta3-endonexin)	3,3777	0,0755	1,1906
1448459_at	Kcnip1	Kv channel-interacting protein 1	3,3774	0,0755	1,6462
1423047_at	Tollip	toll interacting protein	3,3766	0,0755	1,3637
1417062_at	Armc10	armadillo repeat containing 10	3,3765	0,0755	1,5091
1455804_x_at	Oxct1	3-oxoacid CoA transferase 1	3,3763	0,0755	1,3827
1422520_at	Nefm	neurofilament, medium polypeptide	3,3749	0,0756	1,1472
1455741_a_at	Ece1	endothelin converting enzyme 1	3,3737	0,0756	1,5355
1426762_s_at	Kdm1a	lysine (K)-specific demethylase 1A	3,3736	0,0756	2,2586
1450023_at	Gtpbp1	GTP binding protein 1	3,3734	0,0756	1,2535
1426833_at	Eif4g3	eukaryotic translation initiation factor 4 gamma, 3	3,3733	0,0756	1,6589
1415788_at	Ublcp1	ubiquitin-like domain containing CTD phosphatase 1	3,3722	0,0756	1,5770
1450416_at	Cbx5	chromobox homolog 5 (Drosophila HP1a)	3,3721	0,0756	1,3830
1428267_at	Dhx40	DEAH (Asp-Glu-Ala-His) box polypeptide 40	3,3706	0,0756	1,5237
1423073_at	Cmpk1	cytidine monophosphate (UMP-CMP) kinase 1	3,3671	0,0757	1,3960
1450932_s_at	Dock9	dedicator of cytokinesis 9	3,3652	0,0757	1,8396
1451589_at	Gatsl2	GATS protein-like 2	3,3641	0,0757	1,5258
1427349_x_at	2810021G02Rik	RIKEN cDNA 2810021G02 gene	3,3618	0,0757	1,2375
1437045_at	Mapk8	mitogen-activated protein kinase 8	3,3613	0,0757	1,5958
1448372_a_at	Cnpy2	canopy 2 homolog (zebrafish)	3,3609	0,0757	1,3383
1427251_at	Atp2a2	ATPase, Ca++ transporting, cardiac muscle, slow twitch 2	3,3602	0,0757	1,9971
1416343_a_at	Lamp2	lysosomal-associated membrane protein 2	3,3585	0,0757	2,6136
1460726_at	Adss	adenylosuccinate synthetase, non muscle	3,3580	0,0757	1,7868
1451364_at	Polr3gl	polymerase (RNA) III (DNA directed) polypeptide G like	3,3578	0,0757	1,3538
1416562_at	Gad1	glutamic acid decarboxylase 1	3,3565	0,0757	1,7829
1422742_at	Hivep1	human immunodeficiency virus type I enhancer binding protein 1	3,3542	0,0757	1,4521

Probeset ID	Symbol	Gene Name	d-value	q-value	R-fold
1448184_at	Fkbp1a	FK506 binding protein 1a	3,3530	0,0758	1,5862
1429291_at	Psmd1	proteasome (prosome, macropain) 26S subunit, non-ATPase, 1	3,3526	0,0758	1,5419
1423876_at	Fam160b1	family with sequence similarity 160, member B1	3,3512	0,0758	1,3342
1448308_at	Ap3m1	adaptor-related protein complex 3, mu 1 subunit	3,3499	0,0759	1,3756
1456497_x_at	Rps10	ribosomal protein S10	3,3492	0,0759	1,4241
1452163_at	Ets1	E26 avian leukemia oncogene 1, 5' domain	3,3483	0,0759	1,3880
1425327_at	Fam76a	family with sequence similarity 76, member A	3,3475	0,0759	1,6029
1450063_at	NULL	NULL	3,3473	0,0759	1,4286
1423059_at	Ptk2	PTK2 protein tyrosine kinase 2	3,3461	0,0759	1,6369
1456170_x_at	Calr	calreticulin	3,3456	0,0759	1,6405
1434251_at	Cnot1	CCR4-NOT transcription complex, subunit 1	3,3451	0,0759	1,5622
1438167_x_at	Flcn	folliculin	3,3451	0,0759	1,8203
1427260_a_at	Tpm3	tropomyosin 3, gamma	3,3437	0,0759	1,5987
1419950_s_at	Tnpo3	transportin 3	3,3429	0,0759	1,4007
1426930_at	Celf4	CUGBP, Elav-like family member 4	3,3424	0,0759	1,2531
1419279_at	Pip4k2a	phosphatidylinositol-5-phosphate 4-kinase, type II, alpha	3,3420	0,0759	1,3311
1419181_at	Zfp326	zinc finger protein 326	3,3401	0,0760	1,3862
1416566_at	Strap	serine/threonine kinase receptor associated protein	3,3360	0,0760	1,6741
1436736_x_at	D0H4S114	DNA segment, human D4S114	3,3352	0,0760	1,4524
1424924_at	Sec63	SEC63-like (S. cerevisiae)	3,3352	0,0760	1,3380
1449342_at	Ptplb	protein tyrosine phosphatase-like (proline instead of catalytic arginine), member b	3,3352	0,0760	1,6635
1426917_s_at	Scrn3	secernin 3	3,3350	0,0760	1,3013
1422439_a_at	NULL	NULL	3,3341	0,0760	2,1173
1423274_at	Ints6	integrator complex subunit 6	3,3338	0,0760	1,5090
1424603_at	Sumf1	sulfatase modifying factor 1	3,3336	0,0760	1,6489
1418841_s_at	Cdk11b	cyclin-dependent kinase 11B	3,3329	0,0760	1,5731
1455958_s_at	NULL	NULL	3,3328	0,0760	1,8748
1425142_a_at	Hnrnpd	heterogeneous nuclear ribonucleoprotein D	3,3307	0,0760	1,3037
1424515_at	B230354K17Rik	RIKEN cDNA B230354K17 gene	3,3278	0,0760	1,3553
1417129_a_at	Meis2	Meis homeobox 2	3,3276	0,0760	2,0119
1428301_at	NULL	NULL	3,3272	0,0760	1,2119
1417848_at	Zfp704	zinc finger protein 704	3,3250	0,0760	2,0429
1416351_at	Map2k1	mitogen-activated protein kinase kinase 1	3,3249	0,0760	1,2720
1451422_at	Myo18a	myosin XVIIIA	3,3245	0,0760	1,5464
1417386_at	Npepps	aminopeptidase puromycin sensitive	3,3217	0,0760	1,6824
1418963_at	Fam188a	family with sequence similarity 188, member A	3,3212	0,0760	1,8093
1437545_at	Rcor1	REST corepressor 1	3,3206	0,0760	1,7870
1417923_at	Pak3	p21 protein (Cdc42/Rac)-activated kinase 3	3,3190	0,0760	1,1949
1416974_at	Stam2	signal transducing adaptor molecule (SH3 domain and ITAM motif) 2	3,3185	0,0760	1,3492
1453260_a_at	Ppp2r2a	protein phosphatase 2 (formerly 2A), regulatory subunit B (PR 52), alpha isoform	3,3184	0,0760	1,3858
1433942_at	Myo6	myosin VI	3,3178	0,0760	1,5217
1450903_at	Rad23b	RAD23b homolog (S. cerevisiae)	3,3169	0,0760	1,5800
1448236_at	Rdx	radixin	3,3154	0,0760	2,7948
1418525_at	Pcm1	pericentriolar material 1	3,3149	0,0760	1,2943
1452381_at	Creb3l2	cAMP responsive element binding protein 3-like 2	3,3148	0,0760	1,6928
1434332_at	Zzz3	zinc finger, ZZ domain containing 3	3,3147	0,0760	1,5882
1426413_at	Neurod1	neurogenic differentiation 1	3,3135	0,0760	1,5815
1420116_s_at	Golph3	golgi phosphoprotein 3	3,3118	0,0760	1,9084
1429109_at	Msl2	male-specific lethal 2 homolog (Drosophila)	3,3113	0,0760	1,3958
1452058_a_at	Rnf11	ring finger protein 11	3,3103	0,0760	1,7104
1426963_at	Pacs2	phosphofurin acidic cluster sorting protein 2	3,3100	0,0760	1,9060
1424918_at	Tbc1d19	TBC1 domain family, member 19	3,3097	0,0760	1,7688
1426406_at	Setd8	SET domain containing (lysine methyltransferase) 8	3,3070	0,0760	1,7162
1416786_at	Acvr1	activin A receptor, type 1	3,3070	0,0760	1,2757
1449664_s_at	Rnf20	ring finger protein 20	3,3066	0,0760	1,7929
1423737_at	Ndufs3	NADH dehydrogenase (ubiquinone) Fe-S protein 3	3,3063	0,0760	1,4627
1431804_a_at	Sp3	trans-acting transcription factor 3	3,3060	0,0760	1,3815
1460430_at	NULL	NULL	3,3048	0,0760	1,2564
1428904_at	Ammecr1l	AMME chromosomal region gene 1-like	3,3042	0,0760	1,4830
1450675_at	Smap2	stromal membrane-associated GTPase-activating protein 2	3,3007	0,0761	2,0894
1422764_at	Mapre1	microtubule-associated protein, RP/EB family, member 1	3,3001	0,0761	1,5820
1423653_at	Atp1a1	ATPase, Na+/K+ transporting, alpha 1 polypeptide	3,2997	0,0762	1,3817
1453731_a_at	Dram2	VDNA-damage regulated autophagy modulator 2	3,2958	0,0762	1,8981
1450655_at	Pten	phosphatase and tensin homolog	3,2947	0,0762	1,5958
1453849_s_at	Hnrnpab	heterogeneous nuclear ribonucleoprotein A/B	3,2937	0,0762	1,8565
1433591_at	Ppp3r1	protein phosphatase 3, regulatory subunit B, alpha isoform (calcineurin B, type I)	3,2936	0,0762	1,6729
1428388_at	Tnks2	tankyrase, TRF1-interacting ankyrin-related ADP-ribose polymerase 2	3,2932	0,0762	1,7417
1449181_at	Fech	ferrochelatase	3,2924	0,0762	1,5756
1423235_at	Ap3b1	adaptor-related protein complex 3, beta 1 subunit	3,2908	0,0762	1,4605
1420907_at	Cd2ap	CD2-associated protein	3,2900	0,0762	1,7631
1428099_a_at	NULL	NULL	3,2896	0,0762	1,9858
1416946_a_at	NULL	NULL	3,2889	0,0762	1,6632
1426068_at	Slc7a4	solute carrier family 7 (cationic amino acid transporter, y+ system), member 4	3,2859	0,0762	1,4330
1418364_a_at	Ftl1	ferritin light chain 1	3,2827	0,0762	1,7639
1433645_at	Slc44a1	solute carrier family 44, member 1	3,2822	0,0762	1,6955

Probeset ID	Symbol	Gene Name	d-value	q-value	R-fold
1451120_at	Polr1d	polymerase (RNA) I polypeptide D	3,2804	0,0762	1,4506
1452659_at	Dek	DEK oncogene (DNA binding)	3,2802	0,0762	2,4497
1434681_at	Txlng	taxilin gamma	3,2794	0,0762	1,2204
1433508_at	Klf6	Kruppel-like factor 6	3,2790	0,0762	1,2162
1439410_x_at	Slc25a39	solute carrier family 25, member 39	3,2784	0,0762	1,2966
1426774_at	Parp12	poly (ADP-ribose) polymerase family, member 12	3,2782	0,0762	1,4140
1433440_x_at	Uba2	ubiquitin-like modifier activating enzyme 2	3,2775	0,0762	1,3277
1416723_at	Tcf4	transcription factor 4	3,2774	0,0762	1,8096
1419436_at	Cfhr1	complement factor H-related 1	3,2773	0,0762	1,0988
1453571_at	Depdc6	DEP domain containing 6	3,2768	0,0762	1,5890
1451146_at	Zfp386	zinc finger protein 386 (Kruppel-like)	3,2768	0,0762	2,1382
1426376_at	Reep5	receptor accessory protein 5	3,2766	0,0762	1,3520
1423474_at	Top1	topoisomerase (DNA) I	3,2763	0,0762	2,1176
1448443_at	Serpini1	serine (or cysteine) peptidase inhibitor, clade I, member 1	3,2757	0,0762	1,4415
1416738_at	Brap	BRCA1 associated protein	3,2756	0,0762	1,2552
1417086_at	Pafah1b1	platelet-activating factor acetylhydrolase, isoform 1b, subunit 1	3,2752	0,0762	1,5204
1452164_at	Fam134a	family with sequence similarity 134, member A	3,2750	0,0762	1,4417
1427231_at	Robo1	roundabout homolog 1 (Drosophila)	3,2744	0,0762	1,3477
1442744_at	Rbm39	RNA binding motif protein 39	3,2737	0,0762	2,1972
1425542_a_at	Ppp2r5c	protein phosphatase 2, regulatory subunit B (B56), gamma isoform	3,2723	0,0762	1,5176
1452005_at	Dlat	dihydrolipoamide S-acetyltransferase (E2 component of pyruvate dehydrogenase complex)	3,2716	0,0762	1,3695
1428760_at	Snapc3	small nuclear RNA activating complex, polypeptide 3	3,2711	0,0762	1,1871
1449280_at	Esm1	endothelial cell-specific molecule 1	3,2711	0,0762	1,5048
1448124_at	Gusb	glucuronidase, beta	3,2710	0,0762	1,8528
1421323_a_at	G3bp2	GTPase activating protein (SH3 domain) binding protein 2	3,2706	0,0762	1,6541
1425591_a_at	Chmp2a	chromatin modifying protein 2A	3,2697	0,0762	1,5033
1423347_at	Sec23a	SEC23A (S. cerevisiae)	3,2693	0,0762	1,3999
1423432_at	Phip	pleckstrin homology domain interacting protein	3,2689	0,0762	2,0567
1434840_at	Agfg1	ArfGAP with FG repeats 1	3,2681	0,0762	1,9271
1416083_at	Zfand5	zinc finger, AN1-type domain 5	3,2672	0,0763	1,8243
1426775_s_at	Scamp1	secretory carrier membrane protein 1	3,2664	0,0763	1,6805
1436859_at	2700007P21Rik	RIKEN cDNA 2700007P21 gene	3,2639	0,0763	1,4472
1451587_a_at	Tiprl	TIP41, TOR signalling pathway regulator-like (S. cerevisiae)	3,2639	0,0763	1,5218
1424399_at	Uck1	uridine-cytidine kinase 1	3,2632	0,0763	1,2316
1424800_at	Enah	enabled homolog (Drosophila)	3,2630	0,0763	2,0627
1416383_a_at	Pcx	pyruvate carboxylase	3,2611	0,0763	1,6096
1416830_at	0610031J06Rik	RIKEN cDNA 0610031J06 gene	3,2606	0,0763	1,1260
1416800_at	Trpm7	transient receptor potential cation channel, subfamily M, member 7	3,2595	0,0763	1,3702
1423309_at	Tgoln1	trans-golgi network protein	3,2585	0,0763	1,6221
1460561_x_at	Sepw1	selenoprotein W, muscle 1	3,2552	0,0763	1,5968
1415676_a_at	Psmb5	proteasome (prosome, macropain) subunit, beta type 5	3,2542	0,0763	1,2674
1423152_at	Vapb	vesicle-associated membrane protein, associated protein B and C	3,2541	0,0763	1,9478
1452714_at	Tanc1	tetratricopeptide repeat, ankyrin repeat and coiled-coil containing 1	3,2531	0,0763	1,5148
1435322_at	Ankrd40	ankyrin repeat domain 40	3,2528	0,0763	1,4963
1415682_at	Xpo7	exportin 7	3,2510	0,0763	1,4845
1421943_at	Tgfa	transforming growth factor alpha	3,2501	0,0763	1,1473
1454984_at	Lifr	leukemia inhibitory factor receptor	3,2499	0,0763	1,7605
1418015_at	Pum2	pumilio 2 (Drosophila)	3,2494	0,0763	2,0150
1422184_a_at	Ak1	adenylate kinase 1	3,2475	0,0763	1,6762
1454831_at	Foxn2	forkhead box N2	3,2470	0,0763	1,3673
1438839_a_at	Ywhae	tyrosine 3-monooxygenase/tryptophan 5-monooxygenase activation protein, epsilon polypeptide	3,2470	0,0763	1,6234
1417179_at	Tspan5	tetraspanin 5	3,2464	0,0763	1,5209
1425052_at	Isoc1	isochorismatase domain containing 1	3,2463	0,0763	1,5119
1421059_a_at	Alg2	asparagine-linked glycosylation 2 homolog (yeast, alpha-1,3-mannosyltransferase)	3,2462	0,0763	1,6129
1422702_at	Azin1	antizyme inhibitor 1	3,2454	0,0763	1,4669
1424452_at	Sltm	SAFB-like, transcription modulator	3,2450	0,0763	1,3622
1439244_a_at	Tnrc6a	trinucleotide repeat containing 6a	3,2441	0,0763	1,5594
1435250_at	Ints8	integrator complex subunit 8	3,2429	0,0763	2,1796
1451748_a_at	Setd3	SET domain containing 3	3,2424	0,0763	1,3739
1419645_at	Cstf2	cleavage stimulation factor, 3' pre-RNA subunit 2	3,2418	0,0763	1,4785
1434000_at	Kras	v-Ki-ras2 Kirsten rat sarcoma viral oncogene homolog	3,2414	0,0763	1,5697
1421968_a_at	Nipa2	non imprinted in Prader-Willi/Angelman syndrome 2 homolog (human)	3,2407	0,0763	1,2012
1460210_at	Pkd1	polycystic kidney disease 1 homolog	3,2405	0,0763	1,5718
1417057_a_at	NULL	NULL	3,2398	0,0763	1,9681
1430128_a_at	Reep6	receptor accessory protein 6	3,2393	0,0763	1,5643
1418012_at	Sh3glb1	SH3-domain GRB2-like B1 (endophilin)	3,2388	0,0763	1,6819
1425186_at	Lmbrd1	LMBR1 domain containing 1	3,2376	0,0763	1,2878
1421205_at	Atm	ataxia telangiectasia mutated homolog (human)	3,2375	0,0763	1,6961
1416923_a_at	Bnip3l	BCL2/adenovirus E1B interacting protein 3-like	3,2369	0,0763	2,2667
1424269_a_at	NULL	NULL	3,2367	0,0763	1,3175
1416071_at	Ddx18	DEAD (Asp-Glu-Ala-Asp) box polypeptide 18	3,2357	0,0763	1,5049
1423451_at	Pgrmc1	progesterone receptor membrane component 1	3,2350	0,0763	1,4974
1460557_at	Supv3l1	suppressor of var1, 3-like 1 (S. cerevisiae)	3,2344	0,0763	1,4320
1428029_a_at	H2afv	H2A histone family, member V	3,2339	0,0763	1,4328
1455222_a_at	Ubp1	upstream binding protein 1	3,2317	0,0763	1,8518
1429554_at	NULL	NULL	3,2316	0,0763	1,5518

Probeset ID	Symbol	Gene Name	d-value	q-value	R-fold
1426671_a_at	Rbm39	RNA binding motif protein 39	3,2309	0,0763	1,9391
1449292_at	Rb1cc1	RB1-inducible coiled-coil 1	3,2306	0,0763	1,1890
1420930_s_at	Ctnnal1	catenin (cadherin associated protein), alpha-like 1	3,2303	0,0763	1,4986
1415687_a_at	Psap	prosaposin	3,2301	0,0763	1,4511
1451368_at	Alg1	asparagine-linked glycosylation 1 homolog (yeast, beta-1,4-mannosyltransferase)	3,2301	0,0763	1,4501
1454673_at	Wasf2	WAS protein family, member 2	3,2291	0,0763	1,7190
1416092_a_at	Mtap4	microtubule-associated protein 4	3,2286	0,0763	1,2266
1417847_at	Ulk2	Unc-51 like kinase 2 (C. elegans)	3,2282	0,0763	1,9076
1459987_s_at	Cct3	chaperonin containing Tcp1, subunit 3 (gamma)	3,2281	0,0763	1,8727
1434335_at	Dip2b	DIP2 disco-interacting protein 2 homolog B (Drosophila)	3,2280	0,0763	1,2664
1427552_a_at	Gstz1	glutathione transferase zeta 1 (maleylacetoacetate isomerase)	3,2280	0,0763	1,7272
1418230_a_at	Lims1	LIM and senescent cell antigen-like domains 1	3,2276	0,0763	1,0956
1418401_a_at	Dusp16	dual specificity phosphatase 16	3,2269	0,0763	1,3764
1422457_s_at	Sumo3	SMT3 suppressor of mif two 3 homolog 3 (yeast)	3,2263	0,0763	2,0214
1451545_at	Tdrd3	tudor domain containing 3	3,2261	0,0763	1,5059
1425485_at	Mtmr6	myotubularin related protein 6	3,2258	0,0763	1,5403
1451108_at	Rnf185	ring finger protein 185	3,2248	0,0763	1,2558
1423831_at	Prkag2	protein kinase, AMP-activated, gamma 2 non-catalytic subunit	3,2246	0,0763	1,2056
1416102_at	Ywhaz	tyrosine 3-monooxygenase/tryptophan 5-monooxygenase activation protein, zeta polypeptide	3,2240	0,0763	1,6820
1423795_at	Sfpq	splicing factor proline/glutamine rich (polypyrimidine tract binding protein associated)	3,2207	0,0763	2,5543
1427705_a_at	Nfkb1	nuclear factor of kappa light polypeptide gene enhancer in B-cells 1, p105	3,2204	0,0763	1,4380
1418926_at	Zeb1	zinc finger E-box binding homeobox 1	3,2195	0,0763	1,4514
1454862_at	Phldb2	pleckstrin homology-like domain, family B, member 2	3,2173	0,0764	1,7785
1417279_at	Itpr1	inositol 1,4,5-triphosphate receptor 1	3,2172	0,0764	1,8123
1426524_at	Gnpda2	glucosamine-6-phosphate deaminase 2	3,2169	0,0764	1,4565
1451274_at	Ogdh	oxoglutarate dehydrogenase (lipoamide)	3,2168	0,0764	1,6414
1453576_at	Nipbl	Nipped-B homolog (Drosophila)	3,2151	0,0764	1,3766
1424839_a_at	Nsun4	NOL1/NOP2/Sun domain family, member 4	3,2150	0,0764	1,3124
1427044_a_at	Amph	amphiphysin	3,2134	0,0764	1,2695
1455742_x_at	NULL	NULL	3,2104	0,0765	1,4152
1416377_at	Pdcd7	programmed cell death 7	3,2099	0,0765	1,2670
1424708_at	Tmed10	transmembrane emp24-like trafficking protein 10 (yeast)	3,2093	0,0765	1,3065
1433953_at	Zfp277	zinc finger protein 277	3,2093	0,0765	1,4623
1417540_at	Elf1	E74-like factor 1	3,2091	0,0765	1,6777
1423316_at	Tmem39a	transmembrane protein 39a	3,2074	0,0765	1,3997
1450012_x_at	Ywhag	tyrosine 3-monooxygenase/tryptophan 5-monooxygenase activation protein, gamma polypeptide	3,2051	0,0765	2,1745
1423395_at	NULL	NULL	3,2045	0,0765	1,3819
1416068_at	NULL	NULL	3,2039	0,0765	1,2550
1424033_at	Srsf7	serine/arginine-rich splicing factor 7	3,2038	0,0765	2,4211
1452251_at	Nbea	neurobeachin	3,2029	0,0765	2,0774
1451716_at	Mafb	v-maf musculoaponeurotic fibrosarcoma oncogene family, protein B (avian)	3,2016	0,0765	1,8843
1451311_a_at	Adipor1	adiponectin receptor 1	3,2012	0,0765	1,2460
1417766_at	Cyb5b	cytochrome b5 type B	3,2012	0,0765	1,4386
1418192_at	Mnt	max binding protein	3,2011	0,0765	1,4972
1437548_at	Bicd1	bicaudal D homolog 1 (Drosophila)	3,2001	0,0765	1,4403
1420628_at	Pura	purine rich element binding protein A	3,1997	0,0765	1,6149
1427902_at	NULL	NULL	3,1983	0,0765	2,5018
1415957_a_at	Rrp1	ribosomal RNA processing 1 homolog (S. cerevisiae)	3,1966	0,0765	1,6102
1435817_x_at	NULL	NULL	3,1961	0,0765	1,1883
1428246_at	Vps26b	vacuolar protein sorting 26 homolog B (yeast)	3,1955	0,0765	1,6570
1417166_at	Psip1	PC4 and SFRS1 interacting protein 1	3,1949	0,0765	1,8662
1427117_at	Mtmr3	myotubularin related protein 3	3,1943	0,0765	1,2119
1417365_a_at	Calm1	calmodulin 1	3,1941	0,0765	1,7667
1429063_s_at	Kif16b	kinesin family member 16B	3,1940	0,0765	1,4192
1415711_at	Arfgef1	ADP-ribosylation factor guanine nucleotide-exchange factor 1(brefeldin A-inhibited)	3,1934	0,0765	1,7595
1438511_a_at	1190002H23Rik	RIKEN cDNA 1190002H23 gene	3,1928	0,0765	2,4928
1417900_a_at	Vldlr	very low density lipoprotein receptor	3,1925	0,0765	1,4681
1428064_at	Arap1	ArfGAP with RhoGAP domain, ankyrin repeat and PH domain 1	3,1917	0,0765	1,3943
1418526_at	Srsf10	serine/arginine-rich splicing factor 10	3,1909	0,0765	1,1823
1420890_at	Hccs	holocytochrome c synthetase	3,1898	0,0765	1,6938
1439255_s_at	NULL	NULL	3,1885	0,0765	2,9270
1434117_at	Tceb3	transcription elongation factor B (SIII), polypeptide 3	3,1885	0,0765	1,4478
1427490_at	Abcb7	ATP-binding cassette, sub-family B (MDR/TAP), member 7	3,1884	0,0765	1,1875
1452124_at	Ank3	ankyrin 3, epithelial	3,1882	0,0765	1,2507
1426345_at	Prepl	prolyl endopeptidase-like	3,1867	0,0765	2,2447
1424192_at	1500011H22Rik	RIKEN cDNA 1500011H22 gene	3,1845	0,0766	1,6008
1418625_s_at	NULL	NULL	3,1830	0,0766	1,2391
1425498_at	Prpf4b	PRP4 pre-mRNA processing factor 4 homolog B (yeast)	3,1816	0,0766	1,8444
1427322_at	Brwd1	bromodomain and WD repeat domain containing 1	3,1813	0,0766	1,8378
1448360_s_at	Angel2	angel homolog 2 (Drosophila)	3,1800	0,0766	1,5581
1436427_at	Prpf4b	PRP4 pre-mRNA processing factor 4 homolog B (yeast)	3,1792	0,0767	1,5918
1416592_at	Glrx	glutaredoxin	3,1784	0,0767	1,3446

Probeset ID	Symbol	Gene Name	d-value	q-value	R-fold
1437666_x_at	Ubc	ubiquitin C	3,1768	0,0767	1,6073
1434435_s_at	Cox17	cytochrome c oxidase, subunit XVII assembly protein homolog (yeast)	3,1760	0,0767	1,2204
1449523_at	Bcl7c	B-cell CLL/lymphoma 7C	3,1759	0,0767	1,4619
1439111_at	Tsc22d1	TSC22 domain family, member 1	3,1752	0,0767	1,2873
1424721_at	Mfap3	microfibrillar-associated protein 3	3,1745	0,0767	1,9371
1417065_at	Egr1	early growth response 1	3,1740	0,0767	1,6265
1416443_a_at	Sae1	SUMO1 activating enzyme subunit 1	3,1739	0,0767	1,2938
1425956_a_at	Cdadc1	cytidine and dCMP deaminase domain containing 1	3,1735	0,0767	1,4312
1418801_at	Zkscan1	zinc finger with KRAB and SCAN domains 1	3,1731	0,0767	1,1362
1420977_at	Man1a2	mannosidase, alpha, class 1A, member 2	3,1717	0,0767	1,3227
1452671_s_at	Lman1	lectin, mannose-binding, 1	3,1715	0,0767	1,3350
1415963_at	Hnrnph2	heterogeneous nuclear ribonucleoprotein H2	3,1715	0,0767	1,8520
1425134_a_at	Pigx	phosphatidylinositol glycan anchor biosynthesis, class X	3,1704	0,0767	1,2826
1419650_at	Zfr	zinc finger RNA binding protein	3,1701	0,0767	1,4250
1418390_at	Phf21a	PHD finger protein 21A	3,1693	0,0767	1,3214
1448673_at	Pvrl3	poliovirus receptor-related 3	3,1692	0,0767	1,8238
1415892_at	Sgpl1	sphingosine phosphate lyase 1	3,1690	0,0767	2,4098
1428330_at	Dopey2	dopey family member 2	3,1681	0,0767	1,9937
1418371_at	Dynll2	dynein light chain LC8-type 2	3,1681	0,0767	1,4119
1421223_a_at	Anxa4	annexin A4	3,1678	0,0767	1,7881
1448837_at	Vil1	villin 1	3,1678	0,0767	1,6156
1448579_at	Glg1	golgi apparatus protein 1	3,1672	0,0767	1,8667
1426124_a_at	Clk1	CDC-like kinase 1	3,1655	0,0767	1,7228
1434832_at	Foxo3	forkhead box O3	3,1651	0,0767	1,4334
1417186_at	Ube2k	ubiquitin-conjugating enzyme E2K (UBC1 homolog, yeast)	3,1644	0,0767	1,3508
1451202_at	C330007P06Rik	RIKEN cDNA C330007P06 gene	3,1642	0,0767	1,4902
1448922_at	Dusp19	dual specificity phosphatase 19	3,1628	0,0768	1,5019
1416866_at	Bet1	blocked early in transport 1 homolog (S. cerevisiae)	3,1625	0,0768	1,5482
1422592_at	NULL	NULL	3,1618	0,0768	1,6944
1420895_at	Tgfbr1	transforming growth factor, beta receptor I	3,1612	0,0768	2,1907
1424654_at	Acp2	acid phosphatase 2, lysosomal	3,1609	0,0768	1,2520
1455987_at	Sec61a1	Sec61 alpha 1 subunit (S. cerevisiae)	3,1600	0,0768	1,6264
1454688_x_at	Tmed10	transmembrane emp24-like trafficking protein 10 (yeast)	3,1598	0,0768	1,9589
1460320_at	Becn1	beclin 1, autophagy related	3,1587	0,0768	1,6773
1418577_at	Trim8	tripartite motif-containing 8	3,1578	0,0768	1,4891
1419271_at	Pax6	paired box gene 6	3,1574	0,0768	1,7937
1425514_at	Pik3r1	phosphatidylinositol 3-kinase, regulatory subunit, polypeptide 1 (p85 alpha)	3,1537	0,0768	1,3261
1437032_x_at	Rbm14	RNA binding motif protein 14	3,1535	0,0768	1,3594
1419251_at	Eps15	epidermal growth factor receptor pathway substrate 15	3,1525	0,0768	1,2209
1416483_at	Ttc3	tetratricopeptide repeat domain 3	3,1521	0,0768	1,5143
1419454_x_at	Pias2	protein inhibitor of activated STAT 2	3,1501	0,0768	1,4676
1452077_at	Ddx3y	DEAD (Asp-Glu-Ala-Asp) box polypeptide 3, Y-linked	3,1501	0,0768	2,1201
1456582_x_at	Mff	mitochondrial fission factor	3,1487	0,0768	1,3127
1416024_x_at	Cct3	chaperonin containing Tcp1, subunit 3 (gamma)	3,1486	0,0768	1,3480
1426614_at	Zmynd8	zinc finger, MYND-type containing 8	3,1481	0,0768	2,4078
1415719_s_at	Armc1	armadillo repeat containing 1	3,1479	0,0768	1,4079
1452265_at	Clasp1	CLIP associating protein 1	3,1478	0,0768	1,9143
1416901_at	Npc2	Niemann Pick type C2	3,1476	0,0768	1,4454
1429859_a_at	Arl2bp	ADP-ribosylation factor-like 2 binding protein	3,1439	0,0770	1,5518
1416240_at	Psmb7	proteasome (prosome, macropain) subunit, beta type 7	3,1434	0,0770	1,3034
1426796_at	Kpna6	karyopherin (importin) alpha 6	3,1426	0,0770	1,2071
1460403_at	Psip1	PC4 and SFRS1 interacting protein 1	3,1421	0,0770	1,4144
1436234_at	4732471D19Rik	RIKEN cDNA 4732471D19 gene	3,1414	0,0770	1,3983
1423071_x_at	6720475J19Rik	RIKEN cDNA 6720475J19 gene	3,1408	0,0770	1,7566
1425228_a_at	Dguok	deoxyguanosine kinase	3,1404	0,0770	1,2262
1426209_at	Strn4	striatin, calmodulin binding protein 4	3,1392	0,0770	1,4998
1418501_a_at	Oxr1	oxidation resistance 1	3,1383	0,0770	3,2408
1416642_a_at	Tpt1	tumor protein, translationally-controlled 1	3,1381	0,0770	1,0743
1418011_a_at	Sh3glb1	SH3-domain GRB2-like B1 (endophilin)	3,1376	0,0770	1,7044
1448948_at	Rag1ap1	recombination activating gene 1 activating protein 1	3,1359	0,0770	1,4577
1426378_at	Eif4b	eukaryotic translation initiation factor 4B	3,1344	0,0771	1,5218
1460389_at	Cdk8	cyclin-dependent kinase 8	3,1339	0,0771	1,8194
1421908_a_at	Tcf12	transcription factor 12	3,1323	0,0771	1,4162
1427294_a_at	Slc38a10	solute carrier family 38, member 10	3,1322	0,0771	1,6032
1424370_s_at	Psmf1	proteasome (prosome, macropain) inhibitor subunit 1	3,1314	0,0771	1,2903
1449211_at	Bpnt1	bisphosphate 3'-nucleotidase 1	3,1305	0,0771	1,9741
1428453_at	Naa30	N(alpha)-acetyltransferase 30, NatC catalytic subunit	3,1290	0,0771	1,5636
1424129_at	Mfsd1	major facilitator superfamily domain containing 1	3,1288	0,0771	1,5813
1460653_at	NULL	NULL	3,1288	0,0771	1,0964
1423374_at	Ncoa6	nuclear receptor coactivator 6	3,1287	0,0771	1,5688
1448721_at	D1Ertd622e	DNA segment, Chr 1, ERATO Doi 622, expressed	3,1283	0,0771	1,5537
1451494_at	Wac	WW domain containing adaptor with coiled-coil	3,1230	0,0773	2,8564
1426586_at	Slc25a11	solute carrier family 25 (mitochondrial carrier oxoglutarate carrier), member 11	3,1217	0,0774	1,3697
1425929_a_at	Rnf14	ring finger protein 14	3,1204	0,0774	1,6236
1417430_at	Cdr2	cerebellar degeneration-related 2	3,1204	0,0774	1,5373
1425354_a_at	Aggf1	angiogenic factor with G patch and FHA domains 1	3,1183	0,0774	1,8970
1426845_at	Pdcd2l	programmed cell death 2-like	3,1177	0,0774	1,1902

Probeset ID	Symbol	Gene Name	d-value	q-value	R-fold
1438320_s_at	Mcm7	minichromosome maintenance deficient 7 (S. cerevisiae)	3,1160	0,0774	1,6436
1438312_s_at	NULL	NULL	3,1158	0,0774	1,4641
1423284_at	Mansc1	MANSC domain containing 1	3,1158	0,0774	1,2955
1415998_at	Vdac1	voltage-dependent anion channel 1	3,1158	0,0774	1,5949
1460548_a_at	Eral1	Era (G-protein)-like 1 (E. coli)	3,1154	0,0774	1,4969
1452430_s_at	NULL	NULL	3,1145	0,0775	2,3633
1436750_a_at	Oxct1	3-oxoacid CoA transferase 1	3,1129	0,0776	3,0501
1416543_at	Nfe2l2	nuclear factor, erythroid derived 2, like 2	3,1121	0,0776	2,1615
1448121_at	Wbp2	WW domain binding protein 2	3,1112	0,0776	1,5242
1426912_at	Rfwd2	ring finger and WD repeat domain 2	3,1102	0,0776	1,4468
1437308_s_at	F2r	coagulation factor II (thrombin) receptor	3,1099	0,0776	1,2133
1450455_s_at	NULL	NULL	3,1085	0,0776	1,4543
1421813_a_at	Psap	prosaposin	3,1073	0,0777	1,6400
1448325_at	Ppp1r15a	protein phosphatase 1, regulatory (inhibitor) subunit 15A	3,1071	0,0777	1,2844
1421662_a_at	Tusc3	tumor suppressor candidate 3	3,1041	0,0778	1,4395
1429041_at	2610005L07Rik	cadherin 11 pseudogene	3,1036	0,0778	1,2211
1433563_s_at	Derl1	Der1-like domain family, member 1	3,1036	0,0778	1,4536
1416620_at	Smarcal1	SWI/SNF related matrix associated, actin dependent regulator of chromatin, subfamily a-like 1	3,1023	0,0778	1,3837
1417087_at	Glg1	golgi apparatus protein 1	3,1009	0,0778	1,5538
1433518_at	Lcmt2	leucine carboxyl methyltransferase 2	3,1000	0,0779	1,3938
1426899_at	Tbc1d23	TBC1 domain family, member 23	3,0996	0,0779	1,4622
1423799_at	Eif1	eukaryotic translation initiation factor 1	3,0989	0,0779	1,5512
1433824_x_at	Grsf1	G-rich RNA sequence binding factor 1	3,0988	0,0779	1,8230
1420123_at	Tcta	T-cell leukemia translocation altered gene	3,0984	0,0779	1,4358
1419812_s_at	Ccdc56	coiled-coil domain containing 56	3,0975	0,0779	1,5515
1449738_s_at	Fam48a	family with sequence similarity 48, member A	3,0950	0,0779	1,6079
1429288_x_at	Stx18	syntaxin 18	3,0947	0,0779	1,2213
1415973_at	Marcks	myristoylated alanine rich protein kinase C substrate	3,0929	0,0779	1,3591
1435800_a_at	Csda	cold shock domain protein A	3,0928	0,0779	1,2481
1423581_at	Nmt2	N-myristoyltransferase 2	3,0926	0,0779	1,1148
1448335_s_at	Ccni	cyclin I	3,0922	0,0779	1,6569
1416369_at	Hiatl1	hippocampus abundant transcript-like 1	3,0913	0,0780	2,3744
1418530_at	Nup160	nucleoporin 160	3,0909	0,0780	1,8714
1417175_at	Csnk1e	casein kinase 1, epsilon	3,0888	0,0781	1,2326
1452896_at	Gtl3	gene trap locus 3	3,0885	0,0781	1,5706
1427974_s_at	Cacna1d	calcium channel, voltage-dependent, L type, alpha 1D subunit	3,0876	0,0781	1,4309
1433527_at	Ireb2	iron responsive element binding protein 2	3,0868	0,0781	1,6094
1453256_at	Polr3c	polymerase (RNA) III (DNA directed) polypeptide C	3,0865	0,0781	1,3514
1420948_s_at	Atrx	alpha thalassemia/mental retardation syndrome X-linked homolog (human)	3,0862	0,0781	1,2307
1424996_at	Cflar	CASP8 and FADD-like apoptosis regulator	3,0861	0,0781	1,6688
1449059_a_at	Oxct1	3-oxoacid CoA transferase 1	3,0861	0,0781	2,0235
1452147_at	Sec24c	Sec24 related gene family, member C (S. cerevisiae)	3,0851	0,0781	1,5803
1452767_at	Rrbp1	ribosome binding protein 1	3,0836	0,0781	1,6972
1433887_at	Dnajc3	DnaJ (Hsp40) homolog, subfamily C, member 3	3,0833	0,0781	1,7219
1416780_at	Pfkm	phosphofructokinase, muscle	3,0830	0,0781	1,2906
1450740_a_at	Mapre1	microtubule-associated protein, RP/EB family, member 1	3,0830	0,0781	1,7972
1435013_at	Heatr2	HEAT repeat containing 2	3,0829	0,0781	1,3185
1420203_at	NULL	NULL	3,0824	0,0781	1,0605
1416332_at	Cirbp	cold inducible RNA binding protein	3,0811	0,0781	1,7034
1420870_at	Mllt10	myeloid/lymphoid or mixed-lineage leukemia (trithorax homolog, Drosophila); translocated to, 10	3,0810	0,0781	1,4163
1452527_a_at	P2rx4	purinergic receptor P2X, ligand-gated ion channel 4	3,0789	0,0781	1,4968
1423840_at	Ccdc56	coiled-coil domain containing 56	3,0779	0,0781	1,7532
1435862_at	Son	Son DNA binding protein	3,0766	0,0781	1,9046
1450957_a_at	Sqstm1	sequestosome 1	3,0765	0,0781	1,4143
1415753_at	Fam108a	family with sequence similarity 108, member A	3,0760	0,0782	1,5541
1433502_s_at	Tsr1	TSR1, 20S rRNA accumulation, homolog (yeast)	3,0748	0,0782	1,3383
1426666_a_at	Sun1	Sad1 and UNC84 domain containing 1	3,0738	0,0782	1,2334
1423785_at	Egln1	EGL nine homolog 1 (C. elegans)	3,0736	0,0782	1,0859
1451296_x_at	Pabpc4	poly(A) binding protein, cytoplasmic 4	3,0724	0,0782	1,3975
1415704_a_at	Cdv3	carnitine deficiency-associated gene expressed in ventricle 3	3,0723	0,0782	1,7205
1455972_x_at	Hadh	hydroxyacyl-Coenzyme A dehydrogenase	3,0709	0,0782	1,4133
1454631_at	Gtf2a1	general transcription factor II A, 1	3,0704	0,0782	1,6701
1417715_a_at	Got2	glutamate oxaloacetate transaminase 2, mitochondrial	3,0689	0,0782	1,3546
1452237_at	Agfg1	ArfGAP with FG repeats 1	3,0687	0,0782	1,7848
1450927_at	Lztr1	leucine-zipper-like transcriptional regulator, 1	3,0687	0,0782	1,4762
1415888_at	Hdgf	hepatoma-derived growth factor	3,0678	0,0782	1,5405
1421828_at	Kpna3	karyopherin (importin) alpha 3	3,0670	0,0782	1,5162
1416726_s_at	Ube2s	ubiquitin-conjugating enzyme E2S	3,0669	0,0782	1,3278
1433888_at	Atp2b2	ATPase, Ca++ transporting, plasma membrane 2	3,0666	0,0782	1,4862
1448535_at	Elp4	elongation protein 4 homolog (S. cerevisiae)	3,0665	0,0782	1,1335
1426705_s_at	Iars	isoleucine-tRNA synthetase	3,0653	0,0782	2,0718
1423824_at	Wls	wntless homolog (Drosophila)	3,0647	0,0782	1,8784
1416330_at	Cd81	CD81 antigen	3,0625	0,0783	1,8376
1416065_a_at	Ankrd10	ankyrin repeat domain 10	3,0622	0,0783	1,8839
1418432_at	Cab39	calcium binding protein 39	3,0614	0,0783	1,4361
1425299_s_at	NULL	NULL	3,0614	0,0783	1,5484

Probeset ID	Symbol	Gene Name	d-value	q-value	R-fold
1426576_at	Sgms1	sphingomyelin synthase 1	3,0600	0,0783	1,3964
1418505_at	Nudt4	nudix (nucleoside diphosphate linked moiety X)-type motif 4	3,0598	0,0783	1,9268
1419460_at	Rpp14	ribonuclease P 14 subunit (human)	3,0597	0,0783	1,5192
1418854_at	Birc2	baculoviral IAP repeat-containing 2	3,0593	0,0783	1,6889
1448255_a_at	Surf4	surfeit gene 4	3,0582	0,0783	1,3894
1421583_at	Creb1	cAMP responsive element binding protein 1	3,0560	0,0783	1,1651
1424390_at	Nupl1	nucleoporin like 1	3,0556	0,0783	1,4971
1415986_at	Clcn4-2	chloride channel 4-2	3,0540	0,0783	1,8042
1418134_at	Slc25a46	solute carrier family 25, member 46	3,0539	0,0783	1,4248
1419642_at	Purb	purine rich element binding protein B	3,0535	0,0783	1,8306
1428244_at	Larp1	La ribonucleoprotein domain family, member 1	3,0529	0,0783	1,3104
1436222_at	Gas5	growth arrest specific 5	3,0527	0,0783	1,3946
1415739_at	Rbm42	RNA binding motif protein 42	3,0524	0,0783	1,5862
1423126_at	Atp1b3	ATPase, Na+/K+ transporting, beta 3 polypeptide	3,0519	0,0783	1,8138
1427189_at	Arih1	ariadne ubiquitin-conjugating enzyme E2 binding protein homolog 1 (Drosophila)	3,0517	0,0783	1,2986
1415710_at	Cox18	COX18 cytochrome c oxidase assembly homolog (S. cerevisiae)	3,0512	0,0783	1,3880
1426689_s_at	Sdha	succinate dehydrogenase complex, subunit A, flavoprotein (Fp)	3,0511	0,0783	1,2508
1460214_at	Pcp4	Purkinje cell protein 4	3,0499	0,0783	2,0026
1432003_a_at	Rnf41	ring finger protein 41	3,0498	0,0783	1,3882
1450074_at	Kif3b	kinesin family member 3B	3,0490	0,0783	1,2232
1432029_a_at	NULL	NULL	3,0478	0,0783	1,3255
1449942_a_at	Ilk	integrin linked kinase	3,0473	0,0783	1,5007
1452869_at	Prpf38b	PRP38 pre-mRNA processing factor 38 (yeast) domain containing B	3,0463	0,0783	1,5006
1455482_at	Ap2a2	adaptor protein complex AP-2, alpha 2 subunit	3,0448	0,0784	1,2726
1422538_at	Extl2	exostoses (multiple)-like 2	3,0426	0,0784	1,5160
1448022_at	NULL	NULL	3,0422	0,0784	1,0457
1423687_a_at	Man2c1	mannosidase, alpha, class 2C, member 1	3,0413	0,0784	1,7113
1428115_a_at	Rab2b	RAB2B, member RAS oncogene family	3,0407	0,0784	1,1140
1448864_at	Snrk	SNF related kinase	3,0402	0,0784	1,5120
1416324_s_at	Kctd20	potassium channel tetramerisation domain containing 20	3,0401	0,0784	1,5523
1450986_at	Nop58	NOP58 ribonucleoprotein homolog (yeast)	3,0392	0,0784	1,8862
1450915_at	Ap3b1	adaptor-related protein complex 3, beta 1 subunit	3,0381	0,0784	1,3388
1419300_at	Flt1	FMS-like tyrosine kinase 1	3,0371	0,0784	1,6287
1437290_at	Impad1	inositol monophosphatase domain containing 1	3,0369	0,0784	1,7082
1419866_s_at	NULL	NULL	3,0367	0,0784	1,6758
1451572_a_at	Mff	mitochondrial fission factor	3,0357	0,0784	1,2968
1424198_at	Dlg5	discs, large homolog 5 (Drosophila)	3,0343	0,0784	1,3511
1455988_a_at	Cct6a	chaperonin containing Tcp1, subunit 6a (zeta)	3,0326	0,0785	2,1447
1424882_a_at	Nt5dc2	5'-nucleotidase domain containing 2	3,0324	0,0785	1,7157
1436756_x_at	Hadh	hydroxyacyl-Coenzyme A dehydrogenase	3,0323	0,0785	1,1652
1415899_at	Junb	Jun-B oncogene	3,0308	0,0786	1,6481
1455204_at	Pitpnc1	phosphatidylinositol transfer protein, cytoplasmic 1	3,0305	0,0786	1,4036
1424200_s_at	Seh1l	SEH1-like (S. cerevisiae	3,0295	0,0786	1,7768
1452368_at	Bcr	breakpoint cluster region	3,0287	0,0786	1,3499
1424056_at	Usp48	ubiquitin specific peptidase 48	3,0285	0,0786	1,6131
1426884_at	Rmnd5a	required for meiotic nuclear division 5 homolog A (S. cerevisiae)	3,0283	0,0786	1,9551
1453095_at	Rab10	RAB10, member RAS oncogene family	3,0280	0,0786	1,8278
1427032_at	Herc4	hect domain and RLD 4	3,0274	0,0786	1,6285
1426966_at	NULL	NULL	3,0261	0,0786	1,5481
1460571_at	Dicer1	Dicer1, Dcr-1 homolog (Drosophila)	3,0252	0,0786	1,4455
1415858_at	Eif3c	eukaryotic translation initiation factor 3, subunit C	3,0248	0,0786	1,7351
1460583_at	Golt1b	golgi transport 1 homolog B (S. cerevisiae)	3,0244	0,0786	1,3795
1423337_at	Orc4l	origin recognition complex, subunit 4-like (S. cerevisiae)	3,0242	0,0786	1,3183
1448521_at	NULL	NULL	3,0241	0,0786	1,2353
1431900_a_at	Foxa3	forkhead box A3	3,0222	0,0787	1,5421
1426923_at	Agfg1	ArfGAP with FG repeats 1	3,0217	0,0787	1,5667
1423945_a_at	Pkig	protein kinase inhibitor, gamma	3,0212	0,0787	1,2097
1449076_x_at	Adi1	acireductone dioxygenase 1	3,0210	0,0787	1,4592
1434941_s_at	Esf1	ESF1, nucleolar pre-rRNA processing protein, homolog (S. cerevisiae)	3,0205	0,0787	1,3270
1430053_a_at	Ola1	Obg-like ATPase 1	3,0203	0,0787	1,4494
1448732_at	Ctsb	cathepsin B	3,0200	0,0787	1,4845
1418988_at	Pex7	peroxisomal biogenesis factor 7	3,0172	0,0788	1,5005
1424748_at	Galnt11	UDP-N-acetyl-alpha-D-galactosamine:polypeptide N-acetylgalactosaminyltransferase 11	3,0161	0,0788	1,2862
1455042_at	NULL	NULL	3,0148	0,0788	1,6790
1452150_at	AU040320	expressed sequence AU040320	3,0147	0,0788	1,3935
1433816_at	Mcart1	mitochondrial carrier triple repeat 1	3,0142	0,0788	1,5326
1423078_a_at	Sc4mol	sterol-C4-methyl oxidase-like	3,0137	0,0788	1,6266
1423301_at	Copb1	coatomer protein complex, subunit beta 1	3,0115	0,0788	1,8203
1424607_a_at	NULL	NULL	3,0113	0,0788	2,0414
1451154_a_at	Celf2	CUGBP, Elav-like family member 2	3,0112	0,0788	1,7238
1425340_a_at	Ptpra	protein tyrosine phosphatase, receptor type, A	3,0110	0,0788	1,4770
1415929_at	Map1lc3b	microtubule-associated protein 1 light chain 3 beta	3,0102	0,0788	1,4846
1451495_at	Wac	WW domain containing adaptor with coiled-coil	3,0101	0,0788	1,8118
1418292_at	Asna1	arsA arsenite transporter, ATP-binding, homolog 1 (bacterial)	3,0100	0,0788	1,4673
1419452_at	Uchl5	ubiquitin carboxyl-terminal esterase L5	3,0099	0,0788	1,3309
1417663_a_at	Ndrg3	N-myc downstream regulated gene 3	3,0093	0,0788	1,5746
1436747_at	Fam100b	family with sequence similarity 100, member B	3,0060	0,0789	1,2690

Probeset ID	Symbol	Gene Name	d-value	q-value	R-fold
1416591_at	Rab34	RAB34, member of RAS oncogene family	3,0054	0,0789	1,3045
1448737_at	Tspan7	tetraspanin 7	3,0046	0,0790	1,8513
1422450_at	Ctnnd1	catenin (cadherin associated protein), delta 1	3,0038	0,0790	1,3401
1428710_at	Rit1	Ras-like without CAAX 1	3,0033	0,0790	1,2093
1423536_at	Strn3	striatin, calmodulin binding protein 3	3,0033	0,0790	1,3560
1448684_at	Ppp1r2	protein phosphatase 1, regulatory (inhibitor) subunit 2	3,0015	0,0791	1,5151
1416189_a_at	Sec61a1	Sec61 alpha 1 subunit (S. cerevisiae)	3,0010	0,0791	1,3252
1420850_at	Crnkl1	Crn, crooked neck-like 1 (Drosophila)	3,0003	0,0791	1,4390
1417432_a_at	Gnb1	guanine nucleotide binding protein (G protein), beta 1	3,0003	0,0791	1,6640
1420368_at	Denr	density-regulated protein	2,9990	0,0791	1,4149
1448405_a_at	Eid1	EP300 interacting inhibitor of differentiation 1	2,9976	0,0791	1,1848
1422134_at	Fosb	FBJ osteosarcoma oncogene B	2,9953	0,0792	2,2079
1423114_at	Ube2d3	ubiquitin-conjugating enzyme E2D 3 (UBC4/5 homolog, yeast)	2,9946	0,0793	2,2050
1417410_s_at	Prkci	protein kinase C, iota	2,9936	0,0793	1,5328
1418628_at	Khdrbs1	KH domain containing, RNA binding, signal transduction associated 1	2,9905	0,0794	1,1547
1433487_at	Clcn3	chloride channel 3	2,9903	0,0794	1,5044
1431182_at	NULL	NULL	2,9900	0,0794	1,5637
1451070_at	Gdi1	guanosine diphosphate (GDP) dissociation inhibitor 1	2,9900	0,0794	1,4248
1415673_at	Psph	phosphoserine phosphatase	2,9899	0,0794	1,2167
1422791_at	Pafah1b2	platelet-activating factor acetylhydrolase, isoform 1b, subunit 2	2,9891	0,0795	1,3278
1449243_a_at	Rps19	ribosomal protein S19	2,9890	0,0795	1,2939
1440253_at	Psmd11	proteasome (prosome, macropain) 26S subunit, non-ATPase, 11	2,9885	0,0795	1,6213
1438917_x_at	NULL	NULL	2,9880	0,0795	1,4421
1422886_a_at	Clk4	CDC like kinase 4	2,9864	0,0795	1,5166
1421821_at	Ldlr	low density lipoprotein receptor	2,9863	0,0795	1,3671
1429193_at	Ankib1	ankyrin repeat and IBR domain containing 1	2,9862	0,0795	1,7227
1437917_at	D530037H12Rik	RIKEN cDNA D530037H12 gene	2,9854	0,0795	1,2524
1454078_a_at	Gal3st1	galactose-3-O-sulfotransferase 1	2,9826	0,0795	1,2331
1426752_at	Phf17	PHD finger protein 17	2,9811	0,0795	1,3735
1436674_at	Rundc3a	RUN domain containing 3A	2,9810	0,0795	1,1886
1426083_a_at	NULL	NULL	2,9807	0,0796	1,7427
1434134_at	Dcaf8	DDB1 and CUL4 associated factor 8	2,9776	0,0797	1,5855
1452656_at	Zdhhc2	zinc finger, DHHC domain containing 2	2,9762	0,0797	2,3241
1427059_at	Tmem184b	transmembrane protein 184b	2,9757	0,0797	1,3543
1434883_at	Mtdh	metadherin	2,9756	0,0797	1,2303
1448785_at	Runx1t1	runt-related transcription factor 1; translocated to, 1 (cyclin D-related)	2,9738	0,0797	1,8403
1423023_at	Sfrp5	secreted frizzled-related sequence protein 5	2,9729	0,0797	1,3720
1436645_a_at	Cnot4	CCR4-NOT transcription complex, subunit 4	2,9729	0,0797	1,9253
1434846_at	Dennd4c	DENN/MADD domain containing 4C	2,9706	0,0798	2,4832
1431827_a_at	Tlk2	tousled-like kinase 2 (Arabidopsis)	2,9699	0,0798	1,3283
1423490_at	Fbxo3	F-box protein 3	2,9692	0,0798	1,8342
1448551_a_at	Trim2	tripartite motif-containing 2	2,9684	0,0798	1,1038
1434751_at	Ids	iduronate 2-sulfatase	2,9680	0,0798	1,5346
1433708_at	Srp68	signal recognition particle 68	2,9664	0,0798	1,3543
1451583_a_at	Mmgt2	membrane magnesium transporter 2	2,9661	0,0799	1,2521
1418191_at	NULL	NULL	2,9636	0,0799	1,3449
1421989_s_at	Papss2	3'-phosphoadenosine 5'-phosphosulfate synthase 2	2,9635	0,0799	1,8417
1422946_a_at	Dnmt1	DNA methyltransferase (cytosine-5) 1	2,9631	0,0799	1,3670
1454136_a_at	4921524J17Rik	RIKEN cDNA 4921524J17 gene	2,9628	0,0799	1,6297
1451473_a_at	Cryzl1	crystallin, zeta (quinone reductase)-like 1	2,9626	0,0799	1,1994
1420843_at	Ptprf	protein tyrosine phosphatase, receptor type, F	2,9618	0,0799	1,4154
1424319_at	Oraov1	oral cancer overexpressed 1	2,9591	0,0800	1,2666
1450913_at	B4galt6	UDP-Gal:betaGlcNAc beta 1,4-galactosyltransferase, polypeptide 6	2,9586	0,0800	1,2592
1426369_at	Far1	fatty acyl CoA reductase 1	2,9583	0,0800	1,4873
1427171_at	Rlf	rearranged L-myc fusion sequence	2,9572	0,0800	1,2755
1416344_at	Lamp2	lysosomal-associated membrane protein 2	2,9569	0,0800	1,5497
1451137_a_at	Brd8	bromodomain containing 8	2,9568	0,0800	1,2829
1416060_at	Tbc1d15	TBC1 domain family, member 15	2,9567	0,0800	1,9642
1416252_at	Stk38	serine/threonine kinase 38	2,9566	0,0800	1,5938
1448626_at	Cdk5rap1	CDK5 regulatory subunit associated protein 1	2,9556	0,0800	1,4685
1451083_s_at	Aars	alanyl-tRNA synthetase	2,9553	0,0800	1,2526
1436908_at	Pcm1	pericentriolar material 1	2,9553	0,0800	1,6031
1451671_at	Gorasp1	golgi reassembly stacking protein 1	2,9549	0,0800	1,3615
1418660_at	Clock	circadian locomotor output cycles kaput	2,9525	0,0800	1,1871
1460697_s_at	Snrnp27	small nuclear ribonucleoprotein 27 (U4/U6.U5)	2,9522	0,0800	1,4356
1426408_at	Celf1	CUGBP, Elav-like family member 1	2,9517	0,0800	1,5363
1416953_at	Ctgf	connective tissue growth factor	2,9509	0,0800	1,1758
1423263_at	NULL	NULL	2,9503	0,0800	1,6912
1425194_a_at	6330577E15Rik	RIKEN cDNA 6330577E15 gene	2,9494	0,0801	1,8687
1420916_at	Prpf40a	PRP40 pre-mRNA processing factor 40 homolog A (yeast)	2,9479	0,0801	1,2926
1422712_a_at	NULL	NULL	2,9473	0,0801	1,4833
1437859_x_at	Eif5a	eukaryotic translation initiation factor 5A	2,9473	0,0801	1,3832
1427192_a_at	Brd8	bromodomain containing 8	2,9466	0,0801	1,2360
1448364_at	Ccng2	cyclin G2	2,9445	0,0801	1,2318
1428872_at	NULL	NULL	2,9443	0,0801	1,6427
1434968_a_at	Actr3	ARP3 actin-related protein 3 homolog (yeast)	2,9436	0,0801	1,8083
1424118_a_at	Spc25	SPC25, NDC80 kinetochore complex component, homolog (S. cerevisiae)	2,9431	0,0801	2,1442
1449618_s_at	2900092E17Rik	RIKEN cDNA 2900092E17 gene	2,9429	0,0801	1,5077
1426703_at	Gak	cyclin G associated kinase	2,9427	0,0801	1,2673

Probeset ID	***Symbol***	***Gene Name***	***d-value***	***q-value***	***R-fold***
1460184_at	Hadh	hydroxyacyl-Coenzyme A dehydrogenase	2,9423	0,0801	1,4671
1448628_at	Scg3	secretogranin III	2,9414	0,0801	1,4410
1420048_at	C78859	expressed sequence C78859	2,9411	0,0801	1,0838
1427982_s_at	Syne2	synaptic nuclear envelope 2	2,9411	0,0801	1,2917
1425508_s_at	Arfrp1	ADP-ribosylation factor related protein 1	2,9408	0,0802	1,3690
1415917_at	Mthfd1	methylenetetrahydrofolate dehydrogenase (NADP+ dependent), methenyltetrahydrofolate cyclohydrolase, formyltetrahydrofolate synthase	2,9405	0,0802	1,1617
1419879_s_at	Trim25	tripartite motif-containing 25	2,9393	0,0802	1,6378
1428286_at	2900097C17Rik	RIKEN cDNA 2900097C17 gene	2,9388	0,0802	1,3092
1456132_x_at	Tsc22d1	TSC22 domain family, member 1	2,9384	0,0802	1,1572
1420093_s_at	Hnrpdl	heterogeneous nuclear ribonucleoprotein D-like	2,9383	0,0802	1,7361
1434899_s_at	Tnrc6a	trinucleotide repeat containing 6a	2,9382	0,0802	2,2543
1426694_at	9030624J02Rik	RIKEN cDNA 9030624J02 gene	2,9379	0,0802	1,2851
1448546_at	Rassf3	Ras association (RalGDS/AF-6) domain family member 3	2,9377	0,0802	1,3602
1454796_at	Ficd	FIC domain containing	2,9376	0,0802	1,7778
1429043_at	Smndc1	survival motor neuron domain containing 1	2,9371	0,0802	1,2940
1418151_at	Mtmr4	myotubularin related protein 4	2,9367	0,0802	1,4358
1423262_a_at	NULL	NULL	2,9367	0,0802	1,6988
1416614_at	Eid1	EP300 interacting inhibitor of differentiation 1	2,9367	0,0802	2,2347
1426476_at	Rasa1	RAS p21 protein activator 1	2,9366	0,0802	2,2043
1427023_at	Phyhipl	phytanoyl-CoA hydroxylase interacting protein-like	2,9357	0,0802	1,2531
1421144_at	Rpgrip1	retinitis pigmentosa GTPase regulator interacting protein 1	2,9353	0,0802	2,2341
1438058_s_at	Ptov1	prostate tumor over expressed gene 1	2,9350	0,0802	1,5293
1435003_at	Pi4ka	phosphatidylinositol 4-kinase, catalytic, alpha polypeptide	2,9344	0,0802	1,4656
1416441_at	Pgcp	plasma glutamate carboxypeptidase	2,9338	0,0802	1,8976
1427305_at	Piga	phosphatidylinositol glycan anchor biosynthesis, class A	2,9336	0,0802	1,6239
1427881_at	Dnttip2	deoxynucleotidyltransferase, terminal, interacting protein 2	2,9332	0,0802	1,2294
1424275_s_at	Trim41	tripartite motif-containing 41	2,9325	0,0802	1,5143
1451348_at	Depdc6	DEP domain containing 6	2,9324	0,0802	1,8673
1429581_at	Acad9	acyl-Coenzyme A dehydrogenase family, member 9	2,9322	0,0802	1,3296
1425020_at	NULL	NULL	2,9296	0,0803	1,7479
1454897_at	6330509M05Rik	RIKEN cDNA 6330509M05 gene	2,9291	0,0803	1,4777
1419111_at	Ing2	inhibitor of growth family, member 2	2,9291	0,0803	1,4378
1418330_at	Ctcf	CCCTC-binding factor	2,9265	0,0803	2,1761
1423890_x_at	Atp1b1	ATPase, Na+/K+ transporting, beta 1 polypeptide	2,9263	0,0803	1,3594
1416611_at	Scamp2	secretory carrier membrane protein 2	2,9262	0,0803	1,5856
1450664_at	Gabpa	GA repeat binding protein, alpha	2,9261	0,0803	1,7527
1429219_at	Ikbip	IKBKB interacting protein	2,9257	0,0803	1,2728
1450042_at	NULL	NULL	2,9252	0,0803	1,4723
1416301_a_at	Ebf1	early B-cell factor 1	2,9232	0,0804	1,0901
1426481_at	Klhl22	kelch-like 22 (Drosophila)	2,9227	0,0804	1,2390
1434676_at	Mtmr9	myotubularin related protein 9	2,9219	0,0804	1,6097
1460378_a_at	Tes	testis derived transcript	2,9209	0,0804	1,2137
1453015_at	4933407C03Rik	RIKEN cDNA 4933407C03 gene	2,9208	0,0804	1,5980
1436522_at	Map3k3	mitogen-activated protein kinase kinase kinase 3	2,9200	0,0804	1,1870
1455026_at	Sbno1	sno, strawberry notch homolog 1 (Drosophila)	2,9196	0,0804	1,2729
1420011_s_at	Xbp1	X-box binding protein 1	2,9194	0,0804	1,5144
1427467_a_at	Rpgr	retinitis pigmentosa GTPase regulator	2,9194	0,0804	1,3739
1424827_a_at	Csnk1a1	casein kinase 1, alpha 1	2,9192	0,0804	1,5630
1448275_at	Tmem19	transmembrane protein 19	2,9175	0,0804	1,2724
1451310_a_at	Ctsl	cathepsin L	2,9174	0,0804	1,2108
1438968_x_at	Spint2	serine protease inhibitor, Kunitz type 2	2,9173	0,0804	1,4725
1424962_at	Tm4sf4	transmembrane 4 superfamily member 4	2,9171	0,0804	1,3430
1427142_s_at	Kdm5b	lysine (K)-specific demethylase 5B	2,9163	0,0805	1,2590
1451317_at	Ythdf2	YTH domain family 2	2,9158	0,0805	1,4140
1455571_x_at	Calm1	calmodulin 1	2,9155	0,0805	1,4477
1423484_at	Bicc1	bicaudal C homolog 1 (Drosophila)	2,9149	0,0805	1,5541
1423319_at	Hhex	hematopoietically expressed homeobox	2,9148	0,0805	1,4316
1416987_at	Elp4	elongation protein 4 homolog (S. cerevisiae)	2,9119	0,0806	1,1109
1416086_at	Tpst2	protein-tyrosine sulfotransferase 2	2,9117	0,0806	1,6085
1448370_at	Ulk1	Unc-51 like kinase 1 (C. elegans)	2,9112	0,0806	1,4733
1434824_at	Baz1b	bromodomain adjacent to zinc finger domain, 1B	2,9111	0,0806	1,4691
1432411_a_at	Fbxw2	F-box and WD-40 domain protein 2	2,9111	0,0806	1,1625
1415691_at	NULL	NULL	2,9098	0,0806	1,4213
1456744_x_at	Flcn	folliculin	2,9078	0,0806	1,2073
1449415_at	Chd1l	chromodomain helicase DNA binding protein 1-like	2,9068	0,0807	1,3025
1427432_a_at	Tra2b	transformer 2 beta homolog (Drosophila)	2,9060	0,0807	1,6726
1418000_a_at	Itm2b	integral membrane protein 2B	2,9058	0,0807	1,4074
1455141_at	Tnrc6a	trinucleotide repeat containing 6a	2,9056	0,0807	1,5235
1451098_at	Chmp1a	chromatin modifying protein 1A	2,9051	0,0807	1,3474
1448968_at	Ubfd1	ubiquitin family domain containing 1	2,9031	0,0807	1,2896
1425328_at	Fam76a	family with sequence similarity 76, member A	2,9027	0,0807	1,8004
1448332_at	Pex19	peroxisomal biogenesis factor 19	2,9027	0,0807	1,1059
1454905_at	Ibtk	inhibitor of Bruton agammaglobulinemia tyrosine kinase	2,9025	0,0807	1,1577
1420622_a_at	Hspa8	heat shock protein 8	2,9019	0,0807	2,1017
1456516_x_at	NULL	NULL	2,9019	0,0807	1,3425
1455696_a_at	Prpf4b	PRP4 pre-mRNA processing factor 4 homolog B (yeast)	2,9015	0,0807	1,8935
1426215_at	Ddc	dopa decarboxylase	2,8996	0,0807	1,5773

Probeset ID	Symbol	Gene Name	d-value	q-value	R-fold
1420745_a_at	Ccndbp1	cyclin D-type binding-protein 1	2,8995	0,0807	1,5438
1417791_a_at	Zfml	zinc finger, matrin-like	2,8992	0,0807	1,8284
1417565_at	Abhd5	abhydrolase domain containing 5	2,8986	0,0807	1,2507
1452753_at	Foxk2	forkhead box K2	2,8982	0,0807	1,2914
1426388_s_at	Ryk	receptor-like tyrosine kinase	2,8979	0,0807	1,3386
1415670_at	Copg	coatomer protein complex, subunit gamma	2,8979	0,0807	1,6508
1448100_at	4833439L19Rik	RIKEN cDNA 4833439L19 gene	2,8940	0,0808	1,3395
1423684_at	Hnrnpk	heterogeneous nuclear ribonucleoprotein K	2,8940	0,0808	1,5993
1455978_a_at	Matn2	matrilin 2	2,8937	0,0808	1,3556
1452125_at	Thrap3	thyroid hormone receptor associated protein 3	2,8935	0,0808	1,9148
1452286_at	Slain2	SLAIN motif family, member 2	2,8932	0,0808	1,7356
1436912_at	Cacnb4	calcium channel, voltage-dependent, beta 4 subunit	2,8926	0,0808	1,0757
1420618_at	Cpeb4	cytoplasmic polyadenylation element binding protein 4	2,8926	0,0808	2,6022
1442745_x_at	Rbm39	RNA binding motif protein 39	2,8922	0,0808	1,9017
1426814_at	Sec16a	SEC16 homolog A (S. cerevisiae)	2,8922	0,0808	1,5649
1418007_at	Gcfc1	GC-rich sequence DNA-binding factor 1	2,8919	0,0808	1,4711
1423531_a_at	Hnrnpa1	heterogeneous nuclear ribonucleoprotein A1	2,8917	0,0808	1,4494
1424503_at	Rab22a	RAB22A, member RAS oncogene family	2,8916	0,0808	1,1579
1416958_at	Nr1d2	nuclear receptor subfamily 1, group D, member 2	2,8914	0,0808	2,2837
1454604_s_at	Tspan12	tetraspanin 12	2,8905	0,0808	1,3633
1451113_a_at	Ik	IK cytokine	2,8902	0,0808	1,7633
1420091_s_at	Morc3	microrchidia 3	2,8897	0,0808	1,3904
1434694_at	Lrrc8a	leucine rich repeat containing 8A	2,8882	0,0808	1,5322
1460351_at	S100a11	S100 calcium binding protein A11 (calgizzarin)	2,8875	0,0808	1,3559
1419693_at	Colec12	collectin sub-family member 12	2,8875	0,0808	1,1690
1436388_a_at	3830406C13Rik	RIKEN cDNA 3830406C13 gene	2,8870	0,0808	1,3476
1448427_at	Ndufa6	NADH dehydrogenase (ubiquinone) 1 alpha subcomplex, 6 (B14)	2,8869	0,0808	1,3483
1448253_at	Glud1	glutamate dehydrogenase 1	2,8865	0,0808	1,6092
1426355_a_at	6330578E17Rik	RIKEN cDNA 6330578E17 gene	2,8833	0,0809	1,4918
1417082_at	Anp32b	acidic (leucine-rich) nuclear phosphoprotein 32 family, member B	2,8803	0,0809	1,5544
1428078_at	Rnft1	ring finger protein, transmembrane 1	2,8803	0,0809	1,2322
1450858_a_at	Ube2d3	ubiquitin-conjugating enzyme E2D 3 (UBC4/5 homolog, yeast)	2,8790	0,0810	1,4859
1423030_at	Vcp	valosin containing protein	2,8787	0,0810	1,7001
1424769_s_at	Cald1	caldesmon 1	2,8784	0,0810	2,2499
1423131_at	Nsa2	NSA2 ribosome biogenesis homolog (S. cerevisiae)	2,8770	0,0810	1,5007
1417031_at	Tmem206	transmembrane protein 206	2,8743	0,0811	1,5425
1417688_at	Fam20c	family with sequence similarity 20, member C	2,8740	0,0811	1,4360
1439148_a_at	Pfkl	phosphofructokinase, liver, B-type	2,8712	0,0811	1,9728
1423545_a_at	Zfp207	zinc finger protein 207	2,8708	0,0811	1,7502
1433627_at	Sec23ip	Sec23 interacting protein	2,8696	0,0812	1,5631
1435758_at	B4galt6	UDP-Gal:betaGlcNAc beta 1,4-galactosyltransferase, polypeptide 6	2,8686	0,0813	1,8526
1423399_a_at	Yaf2	YY1 associated factor 2	2,8676	0,0813	1,8030
1428077_at	NULL	NULL	2,8675	0,0813	1,5122
1451968_at	Xrcc5	X-ray repair complementing defective repair in Chinese hamster cells 5	2,8674	0,0813	1,4649
1418715_at	Pank1	pantothenate kinase 1	2,8668	0,0813	1,1932
1438011_at	Pcyt1a	phosphate cytidylyltransferase 1, choline, alpha isoform	2,8663	0,0813	1,2679
1418447_at	Golga5	golgi autoantigen, golgin subfamily a, 5	2,8663	0,0813	1,6517
1416814_at	Tia1	cytotoxic granule-associated RNA binding protein 1	2,8663	0,0813	1,6520
1424982_a_at	2700078E11Rik	RIKEN cDNA 2700078E11 gene	2,8662	0,0813	1,4190
1452774_at	Hnrnpa3	heterogeneous nuclear ribonucleoprotein A3	2,8656	0,0813	1,6329
1451346_at	Mtap	methylthioadenosine phosphorylase	2,8655	0,0813	1,4223
1451398_at	NULL	NULL	2,8651	0,0813	1,2646
1418154_at	N4bp1	NEDD4 binding protein 1	2,8641	0,0813	1,2804
1448280_at	Syp	synaptophysin	2,8638	0,0813	2,3450
1448883_at	Lgmn	legumain	2,8636	0,0813	1,3311
1424540_at	Hipk1	homeodomain interacting protein kinase 1	2,8636	0,0813	1,9019
1454643_at	Ubap2l	ubiquitin associated protein 2-like	2,8634	0,0813	1,5025
1419170_at	Fam174a	family with sequence similarity 174, member A	2,8613	0,0814	1,5902
1448526_at	Kpnb1	karyopherin (importin) beta 1	2,8606	0,0814	1,7447
1416386_a_at	M6pr	mannose-6-phosphate receptor, cation dependent	2,8605	0,0814	1,5840
1419472_s_at	NULL	NULL	2,8603	0,0814	1,4966
1450684_at	Etv1	ets variant gene 1	2,8603	0,0814	1,6211
1417507_at	Cyb561	cytochrome b-561	2,8593	0,0814	1,6486
1417749_a_at	Tjp1	tight junction protein 1	2,8584	0,0814	1,7638
1455030_at	Ptprj	protein tyrosine phosphatase, receptor type, J	2,8584	0,0814	1,5769
1425538_x_at	Ceacam1	carcinoembryonic antigen-related cell adhesion molecule 1	2,8584	0,0814	1,2022
1418331_at	1110031I02Rik	RIKEN cDNA 1110031I02 gene	2,8579	0,0814	1,6210
1427016_at	4932438A13Rik	RIKEN cDNA 4932438A13 gene	2,8575	0,0814	1,5319
1426827_at	Ythdc1	YTH domain containing 1	2,8565	0,0814	1,5737
1417504_at	Calb1	calbindin 1	2,8564	0,0814	1,9852
1455788_x_at	Poldip3	polymerase (DNA-directed), delta interacting protein 3	2,8551	0,0814	1,9867
1424001_at	Mki67ip	Mki67 (FHA domain) interacting nucleolar phosphoprotein	2,8537	0,0814	1,1260
1416637_at	Slc4a2	solute carrier family 4 (anion exchanger), member 2	2,8527	0,0814	1,2616
1448545_at	Sdc2	syndecan 2	2,8513	0,0814	1,4244
1426609_at	Dis3	DIS3 mitotic control homolog (S. cerevisiae)	2,8511	0,0814	1,2717
1434644_at	Tbl1x	transducin (beta)-like 1 X-linked	2,8503	0,0814	1,4212
1424071_s_at	BC018507	cDNA sequence BC018507	2,8501	0,0814	1,4671
1456048_at	Cpeb3	cytoplasmic polyadenylation element binding protein 3	2,8500	0,0814	1,4463
1415715_at	Tmem129	transmembrane protein 129	2,8500	0,0814	1,4578

Probeset ID	Symbol	Gene Name	d-value	q-value	R-fold
1418283_at	Cldn4	claudin 4	2,8500	0,0814	1,4575
1451345_at	Mtap	methylthioadenosine phosphorylase	2,8498	0,0814	1,1298
1429139_at	Otud7b	OTU domain containing 7B	2,8494	0,0814	1,5414
1417983_a_at	Ube2v2	ubiquitin-conjugating enzyme E2 variant 2	2,8488	0,0814	1,4396
1450940_at	Gdap1	ganglioside-induced differentiation-associated-protein 1	2,8485	0,0814	1,5077
1417733_at	Rnf146	ring finger protein 146	2,8478	0,0814	1,6612
1448429_at	Gyg	glycogenin	2,8459	0,0814	1,3003
1416772_at	Cpt2	carnitine palmitoyltransferase 2	2,8447	0,0814	1,4314
1424141_at	Hectd1	HECT domain containing 1	2,8442	0,0814	1,3878
1435768_at	Arid4b	AT rich interactive domain 4B (RBP1-like)	2,8440	0,0814	2,0417
1416973_at	Nhp2l1	NHP2 non-histone chromosome protein 2-like 1 (S. cerevisiae)	2,8440	0,0814	1,2474
1451217_a_at	Immp1l	IMP1 inner mitochondrial membrane peptidase-like (S. cerevisiae)	2,8431	0,0814	1,3519
1426487_a_at	Rbbp6	retinoblastoma binding protein 6	2,8429	0,0814	1,2415
1417293_at	NULL	NULL	2,8410	0,0814	1,5484
1450623_at	Gnb2	guanine nucleotide binding protein (G protein), beta 2	2,8397	0,0814	1,5584
1424875_at	Spg20	spastic paraplegia 20, spartin (Troyer syndrome) homolog (human)	2,8389	0,0814	1,1853
1428138_s_at	Arl8b	ADP-ribosylation factor-like 8B	2,8381	0,0814	1,5693
1433507_a_at	NULL	NULL	2,8372	0,0815	2,4499
1423597_at	Atp8a1	ATPase, aminophospholipid transporter (APLT), class I, type 8A, member 1	2,8368	0,0815	1,7175
1456175_a_at	Copb2	coatomer protein complex, subunit beta 2 (beta prime)	2,8359	0,0815	1,3550
1416595_at	Mrps22	mitochondrial ribosomal protein S22	2,8354	0,0815	1,2848
1418444_a_at	Gde1	glycerophosphodiester phosphodiesterase 1	2,8347	0,0815	1,3474
1434027_at	Rcan3	regulator of calcineurin 3	2,8342	0,0815	1,4642
1416904_at	Mbnl1	muscleblind-like 1 (Drosophila)	2,8333	0,0815	1,5029
1426307_at	Cyb5r4	cytochrome b5 reductase 4	2,8331	0,0815	1,8950
1426759_at	Map4k3	mitogen-activated protein kinase kinase kinase kinase 3	2,8327	0,0815	1,9113
1433541_a_at	Ubap2l	ubiquitin associated protein 2-like	2,8323	0,0815	1,4049
1427089_at	Ccnt2	cyclin T2	2,8322	0,0815	2,0846
1418016_at	Pum2	pumilio 2 (Drosophila)	2,8320	0,0815	1,4598
1433497_at	Aqr	aquarius	2,8311	0,0815	1,4921
1426674_at	Eif3b	eukaryotic translation initiation factor 3, subunit B	2,8301	0,0816	1,3581
1424358_at	Ube2e2	ubiquitin-conjugating enzyme E2E 2 (UBC4/5 homolog, yeast)	2,8291	0,0816	1,4129
1443856_at	Rabep1	rabaptin, RAB GTPase binding effector protein 1	2,8290	0,0816	1,5946
1448893_at	Ncor2	nuclear receptor co-repressor 2	2,8280	0,0816	1,6842
1415693_at	Derl1	Der1-like domain family, member 1	2,8277	0,0816	1,3729
1437061_at	Mbd1	methyl-CpG binding domain protein 1	2,8266	0,0816	1,2727
1428881_at	NULL	NULL	2,8266	0,0816	1,2083
1426887_at	Nudt11	nudix (nucleoside diphosphate linked moiety X)-type motif 11	2,8258	0,0816	1,4759
1423076_at	Snx9	sorting nexin 9	2,8252	0,0816	1,3870
1452675_at	Rbm22	RNA binding motif protein 22	2,8251	0,0816	1,3875
1418560_at	Pdha1	pyruvate dehydrogenase E1 alpha 1	2,8248	0,0816	1,1199
1448908_at	Ppap2b	phosphatidic acid phosphatase type 2B	2,8242	0,0816	1,6515
1434625_at	4930432O21Rik	RIKEN cDNA 4930432O21 gene	2,8234	0,0816	1,2284
1446148_x_at	Rbm39	RNA binding motif protein 39	2,8234	0,0816	1,4034
1423251_at	Luc7l2	LUC7-like 2 (S. cerevisiae)	2,8220	0,0816	2,2507
1421115_a_at	Zdhhc16	zinc finger, DHHC domain containing 16	2,8217	0,0816	1,2287
1433631_at	Eif5	eukaryotic translation initiation factor 5	2,8210	0,0816	2,0546
1415773_at	Ncl	nucleolin	2,8207	0,0816	1,9132
1438368_a_at	Matr3	matrin 3	2,8203	0,0816	1,5130
1422555_s_at	Gna13	guanine nucleotide binding protein, alpha 13	2,8195	0,0816	2,1054
1452122_at	AI314180	expressed sequence AI314180	2,8191	0,0816	1,6513
1452743_at	Pole3	polymerase (DNA directed), epsilon 3 (p17 subunit)	2,8182	0,0816	1,1402
1420342_at	Gdap10	ganglioside-induced differentiation-associated-protein 10	2,8182	0,0816	1,2441
1435524_at	Snhg8	small nucleolar RNA host gene 8	2,8176	0,0817	1,6410
1448819_at	Eif2s2	eukaryotic translation initiation factor 2, subunit 2 (beta)	2,8171	0,0817	1,2582
1425140_at	Lactb2	lactamase, beta 2	2,8171	0,0817	1,5897
1416152_a_at	Srsf3	serine/arginine-rich splicing factor 3	2,8169	0,0817	1,6931
1419404_s_at	NULL	NULL	2,8154	0,0817	1,2156
1417865_at	Tnfaip1	tumor necrosis factor, alpha-induced protein 1 (endothelial)	2,8133	0,0819	1,7794
1424760_a_at	Smyd2	SET and MYND domain containing 2	2,8131	0,0819	1,8410
1437885_at	D030029J20Rik	RIKEN cDNA D030029J20 gene	2,8115	0,0819	1,1341
1435468_at	B3gnt9-ps	UDP-GlcNAc:betaGal beta-1,3-N-acetylglucosaminyltransferase 9, pseudogene	2,8107	0,0819	1,0885
1426248_at	Stk24	serine/threonine kinase 24 (STE20 homolog, yeast)	2,8105	0,0819	1,3645
1448272_at	Btg2	B-cell translocation gene 2, anti-proliferative	2,8095	0,0819	1,5363
1426272_at	Lmbr1	limb region 1	2,8094	0,0819	1,5091
1449083_at	Ccdc91	coiled-coil domain containing 91	2,8091	0,0820	1,9093
1426754_x_at	Ckap4	cytoskeleton-associated protein 4	2,8086	0,0820	1,2609
1418232_s_at	Lims1	LIM and senescent cell antigen-like domains 1	2,8084	0,0820	1,1917
1460323_at	Tars	threonyl-tRNA synthetase	2,8077	0,0820	1,5800
1433516_a_at	Myeov2	myeloma overexpressed 2	2,8073	0,0820	1,3736
1427640_a_at	Runx1t1	runt-related transcription factor 1; translocated to, 1 (cyclin D-related)	2,8071	0,0820	1,6431
1436714_at	Lpp	LIM domain containing preferred translocation partner in lipoma	2,8065	0,0820	2,2736
1439443_x_at	Tkt	transketolase	2,8059	0,0820	1,8151
1448667_x_at	Tob2	transducer of ERBB2, 2	2,8057	0,0820	1,5992
1423922_s_at	Ints3	integrator complex subunit 3	2,8053	0,0820	1,1837
1424621_at	Krcc1	lysine-rich coiled-coil 1	2,8031	0,0821	1,9644
1449043_at	Naga	N-acetyl galactosaminidase, alpha	2,8031	0,0821	1,4967

Probeset ID	Symbol	Gene Name	d-value	q-value	R-fold
1427475_a_at	Pdlim5	PDZ and LIM domain 5	2,8016	0,0821	1,2718
1415879_a_at	Rplp2	ribosomal protein, large P2	2,8012	0,0821	1,4955
1416385_a_at	M6pr	mannose-6-phosphate receptor, cation dependent	2,8003	0,0821	1,5506
1425492_at	Bmpr1a	bone morphogenetic protein receptor, type 1A	2,8003	0,0821	1,4122
1416055_at	NULL	NULL	2,8001	0,0821	1,0796
1419244_a_at	Rab14	RAB14, member RAS oncogene family	2,8000	0,0821	1,9817
1455060_at	G3bp1	Ras-GTPase-activating protein SH3-domain binding protein 1	2,7997	0,0821	1,8968
1460329_at	B4galt6	UDP-Gal:betaGlcNAc beta 1,4-galactosyltransferase, polypeptide 6	2,7991	0,0821	1,9553
1427152_at	Qser1	glutamine and serine rich 1	2,7981	0,0821	1,2066
1423901_at	Trip12	thyroid hormone receptor interactor 12	2,7977	0,0821	1,4466
1449345_at	Ccdc34	coiled-coil domain containing 34	2,7968	0,0821	1,2557
1449128_at	Ccdc43	coiled-coil domain containing 43	2,7966	0,0821	1,1448
1448991_a_at	NULL	NULL	2,7961	0,0821	1,3696
1452620_at	Pck2	phosphoenolpyruvate carboxykinase 2 (mitochondrial)	2,7960	0,0821	1,2338
1415712_at	Zranb1	zinc finger, RAN-binding domain containing 1	2,7951	0,0821	1,8324
1422673_at	Prkd1	protein kinase D1	2,7945	0,0821	1,6046
1423800_at	Dars	aspartyl-tRNA synthetase	2,7944	0,0821	1,3756
1419700_a_at	Prom1	prominin 1	2,7940	0,0821	1,6587
1417833_at	Zc3h10	zinc finger CCCH type containing 10	2,7915	0,0821	1,3069
1418960_at	Phf20l1	PHD finger protein 20-like 1	2,7915	0,0821	1,6948
1454681_at	Esrp1	epithelial splicing regulatory protein 1	2,7914	0,0821	1,6599
1416230_at	Rfk	riboflavin kinase	2,7908	0,0821	1,5014
1417377_at	Cadm1	cell adhesion molecule 1	2,7890	0,0822	1,3750
1451943_a_at	Ppm1a	protein phosphatase 1A, magnesium dependent, alpha isoform	2,7886	0,0822	1,3382
1427949_at	Rnf160	ring finger protein 160	2,7876	0,0822	1,5915
1416860_s_at	Ing1	inhibitor of growth family, member 1	2,7875	0,0822	1,2067
1423053_at	Arf4	ADP-ribosylation factor 4	2,7863	0,0822	1,5737
1455012_s_at	Trim37	tripartite motif-containing 37	2,7849	0,0822	1,9526
1417161_at	Cdk2ap2	CDK2-associated protein 2	2,7845	0,0822	1,2820
1435866_s_at	Hist3h2a	histone cluster 3, H2a	2,7838	0,0822	1,5695
1449373_at	Dnajc3	DnaJ (Hsp40) homolog, subfamily C, member 3	2,7837	0,0822	1,4694
1426696_at	Lrpap1	low density lipoprotein receptor-related protein associated protein 1	2,7836	0,0822	1,2279
1455019_x_at	Ckap4	cytoskeleton-associated protein 4	2,7826	0,0822	1,3435
1448830_at	Dusp1	dual specificity phosphatase 1	2,7823	0,0822	1,6776
1429369_at	Tnpo3	transportin 3	2,7820	0,0822	1,6028
1426840_at	Ythdf3	YTH domain family 3	2,7818	0,0822	2,1821
1429709_at	Pmpcb	peptidase (mitochondrial processing) beta	2,7811	0,0822	1,6518
1452499_a_at	Kif2a	kinesin family member 2A	2,7797	0,0823	1,2018
1417134_at	Srpk2	serine/arginine-rich protein specific kinase 2	2,7794	0,0823	1,5397
1441023_at	Eif2s2	eukaryotic translation initiation factor 2, subunit 2 (beta)	2,7788	0,0823	1,2694
1417753_at	Pkd2	polycystic kidney disease 2	2,7788	0,0823	1,6780
1426722_at	Slc38a2	solute carrier family 38, member 2	2,7779	0,0823	2,5459
1422998_a_at	Glrx2	glutaredoxin 2 (thioltransferase)	2,7778	0,0823	1,9429
1435634_at	Pcmtd1	protein-L-isoaspartate (D-aspartate) O-methyltransferase domain containing 1	2,7776	0,0823	1,6473
1422895_at	Vamp4	vesicle-associated membrane protein 4	2,7753	0,0824	1,2385
1418150_at	Mtmr4	myotubularin related protein 4	2,7748	0,0824	1,3825
1425828_at	NULL	NULL	2,7743	0,0824	1,6873
1434856_at	Ankrd44	ankyrin repeat domain 44	2,7733	0,0824	1,8708
1424863_a_at	Hipk2	homeodomain interacting protein kinase 2	2,7732	0,0824	1,3876
1419308_at	Invs	inversin	2,7729	0,0824	1,2538
1426531_at	Zmynd11	zinc finger, MYND domain containing 11	2,7728	0,0824	1,5963
1415684_at	Atg5	autophagy-related 5 (yeast)	2,7727	0,0824	1,4386
1450389_s_at	Pip5k1b	phosphatidylinositol-4-phosphate 5-kinase, type 1 beta	2,7721	0,0824	1,3507
1422045_a_at	Ptpn12	protein tyrosine phosphatase, non-receptor type 12	2,7718	0,0824	1,5402
1436570_at	NULL	NULL	2,7713	0,0824	1,3666
1417356_at	Peg3	paternally expressed 3	2,7707	0,0824	2,0888
1460173_at	Lasp1	LIM and SH3 protein 1	2,7704	0,0824	1,4559
1434471_at	BC003331	cDNA sequence BC003331	2,7689	0,0824	1,5410
1451679_at	6530401D17Rik	RIKEN cDNA 6530401D17 gene	2,7682	0,0824	1,5107
1434469_at	Otud4	OTU domain containing 4	2,7679	0,0824	1,6420
1426819_at	Hipk3	homeodomain interacting protein kinase 3	2,7666	0,0824	2,0750
1424189_at	Pigc	phosphatidylinositol glycan anchor biosynthesis, class C	2,7653	0,0824	1,6435
1426922_s_at	Agfg1	ArfGAP with FG repeats 1	2,7653	0,0824	1,1668
1450001_a_at	Ush1c	Usher syndrome 1C homolog (human)	2,7650	0,0824	1,4569
1448778_at	Srsf4	serine/arginine-rich splicing factor 4	2,7648	0,0824	1,4539
1426972_at	Sec24d	Sec24 related gene family, member D (S. cerevisiae)	2,7642	0,0824	1,7523
1438835_a_at	Eftud2	elongation factor Tu GTP binding domain containing 2	2,7639	0,0824	1,5499
1421112_at	Nkx2-2	NK2 transcription factor related, locus 2 (Drosophila)	2,7634	0,0824	1,9124
1417384_at	Entpd5	ectonucleoside triphosphate diphosphohydrolase 5	2,7622	0,0824	1,2315
1417393_a_at	Fam132a	family with sequence similarity 132, member A	2,7613	0,0825	1,2312
1425464_at	Gata6	GATA binding protein 6	2,7598	0,0825	1,5520
1442006_at	NULL	NULL	2,7598	0,0825	1,5936
1423063_at	Dnmt3a	DNA methyltransferase 3A	2,7590	0,0825	1,4715
1425102_a_at	Ace2	angiotensin I converting enzyme (peptidyl-dipeptidase A) 2	2,7588	0,0825	1,2680
1460246_at	Mecp2	methyl CpG binding protein 2	2,7586	0,0825	1,7747
1424561_at	Ece2	endothelin converting enzyme 2	2,7586	0,0825	1,3977
1452735_at	Pcnp	PEST proteolytic signal containing nuclear protein	2,7563	0,0826	1,4464
1448244_at	Lypla1	lysophospholipase 1	2,7561	0,0826	1,5365

Probeset ID	Symbol	Gene Name	d-value	q-value	R-fold
1415863_at	Eif4g2	eukaryotic translation initiation factor 4, gamma 2	2,7557	0,0826	1,7444
1423266_at	2810405K02Rik	RIKEN cDNA 2810405K02 gene	2,7524	0,0826	1,4300
1416140_a_at	Dhx30	DEAH (Asp-Glu-Ala-His) box polypeptide 30	2,7521	0,0826	2,3136
1450108_at	Kif1a	kinesin family member 1A	2,7517	0,0826	1,4317
1422559_at	Ube2n	ubiquitin-conjugating enzyme E2N	2,7514	0,0827	1,5707
1432042_a_at	Smu1	smu-1 suppressor of mec-8 and unc-52 homolog (C. elegans)	2,7492	0,0827	1,3295
1415737_at	Rfk	riboflavin kinase	2,7490	0,0827	1,2464
1448784_at	Taf10	TAF10 RNA polymerase II, TATA box binding protein (TBP)-associated factor	2,7485	0,0827	1,4589
1452697_at	Ctdp1	CTD (carboxy-terminal domain, RNA polymerase II, polypeptide A) phosphatase, subunit 1	2,7479	0,0827	1,3061
1433899_x_at	Tsc22d1	TSC22 domain family, member 1	2,7479	0,0827	1,2127
1436420_a_at	Ipo4	importin 4	2,7476	0,0827	2,3510
1436846_x_at	NULL	NULL	2,7418	0,0829	1,4196
1455056_at	Lmo7	LIM domain only 7	2,7417	0,0829	1,6919
1421907_at	Med1	mediator complex subunit 1	2,7410	0,0829	1,6653
1460324_at	Dnmt3a	DNA methyltransferase 3A	2,7406	0,0829	1,3066
1427540_at	Zwint	ZW10 interactor	2,7399	0,0829	1,2139
1419933_at	Pdrg1	p53 and DNA damage regulated 1	2,7398	0,0829	1,0999
1434196_at	Dnaja4	DnaJ (Hsp40) homolog, subfamily A, member 4	2,7396	0,0829	2,4514
1423105_a_at	Yeats4	YEATS domain containing 4	2,7389	0,0829	1,4051
1435788_at	Actr10	ARP10 actin-related protein 10 homolog (S. cerevisiae)	2,7386	0,0829	1,1590
1427386_at	Arhgef16	Rho guanine nucleotide exchange factor (GEF) 16	2,7382	0,0829	1,5702
1417444_at	E2f5	E2F transcription factor 5	2,7368	0,0830	1,8300
1416533_at	Egln2	EGL nine homolog 2 (C. elegans)	2,7368	0,0830	1,1988
1417112_at	Arl2bp	ADP-ribosylation factor-like 2 binding protein	2,7367	0,0830	1,4344
1426853_at	Set	SET translocation	2,7331	0,0831	1,2971
1417371_at	Peli1	pellino 1	2,7328	0,0831	1,4575
1448565_at	Ppp1r11	protein phosphatase 1, regulatory (inhibitor) subunit 11	2,7326	0,0831	1,3448
1421751_a_at	Psmd14	proteasome (prosome, macropain) 26S subunit, non-ATPase, 14	2,7306	0,0831	1,3623
1452526_a_at	Pax6	paired box gene 6	2,7290	0,0831	1,7563
1435156_at	BC046331	cDNA sequence BC046331	2,7286	0,0831	1,4231
1420760_s_at	Ndrg1	N-myc downstream regulated gene 1	2,7284	0,0831	1,3272
1426225_at	Rbp4	retinol binding protein 4, plasma	2,7282	0,0831	1,4949
1420920_a_at	Arf1	ADP-ribosylation factor 1	2,7279	0,0831	1,4337
1433603_at	Ndufs6	NADH dehydrogenase (ubiquinone) Fe-S protein 6	2,7278	0,0831	1,2169
1454640_at	Chchd7	coiled-coil-helix-coiled-coil-helix domain containing 7	2,7270	0,0831	1,5165
1419869_s_at	Hdlbp	high density lipoprotein (HDL) binding protein	2,7269	0,0831	1,3386
1452173_at	Hadha	hydroxyacyl-Coenzyme A dehydrogenase/3-ketoacyl-Coenzyme A thiolase/enoyl-Coenzyme A hydratase (trifunctional protein), alpha subunit	2,7268	0,0831	1,3610
1460178_at	Lonp2	lon peptidase 2, peroxisomal	2,7268	0,0831	1,3973
1455054_a_at	Dcun1d1	DCN1, defective in cullin neddylation 1, domain containing 1 (S. cerevisiae)	2,7267	0,0831	1,3867
1427253_s_at	Suz12	suppressor of zeste 12 homolog (Drosophila)	2,7257	0,0831	1,5121
1423794_at	Atg13	ATG13 autophagy related 13 homolog (S. cerevisiae)	2,7249	0,0831	1,6970
1418622_at	Rab2a	RAB2A, member RAS oncogene family	2,7246	0,0831	1,7165
1432108_at	Pcgf6	polycomb group ring finger 6	2,7244	0,0831	1,1133
1437999_x_at	Pigq	phosphatidylinositol glycan anchor biosynthesis, class Q	2,7241	0,0831	1,3101
1452198_at	Kdm2b	lysine (K)-specific demethylase 2B	2,7235	0,0831	1,4281
1418244_at	Naa20	N(alpha)-acetyltransferase 20, NatB catalytic subunit	2,7234	0,0831	1,5304
1434011_a_at	Ints5	integrator complex subunit 5	2,7229	0,0831	1,8668
1416610_a_at	Clcn3	chloride channel 3	2,7223	0,0831	1,3785
1418890_a_at	Rab3d	RAB3D, member RAS oncogene family	2,7208	0,0831	1,4226
1415708_at	Tug1	taurine upregulated gene 1	2,7207	0,0831	2,3431
1452149_at	Ube3b	ubiquitin protein ligase E3B	2,7204	0,0831	1,1822
1448362_at	Dnajc7	DnaJ (Hsp40) homolog, subfamily C, member 7	2,7197	0,0832	1,3201
1429692_s_at	Gch1	GTP cyclohydrolase 1	2,7193	0,0832	2,4033
1416816_at	Nek7	NIMA (never in mitosis gene a)-related expressed kinase 7	2,7192	0,0832	1,7688
1415747_s_at	Riok3	RIO kinase 3 (yeast)	2,7191	0,0832	1,5114
1449708_s_at	Chek1	checkpoint kinase 1 homolog (S. pombe)	2,7186	0,0832	1,1867
1415686_at	Rab14	RAB14, member RAS oncogene family	2,7176	0,0832	1,4478
1450900_at	Smek2	SMEK homolog 2, suppressor of mek1 (Dictyostelium)	2,7173	0,0832	1,4687
1451179_a_at	NULL	NULL	2,7151	0,0832	1,6253
1423150_at	Scg5	secretogranin V	2,7138	0,0832	1,3416
1420840_at	Plekha3	pleckstrin homology domain-containing, family A (phosphoinositide binding specific) member 3	2,7137	0,0832	1,3212
1452371_at	Srsf11	serine/arginine-rich splicing factor 11	2,7136	0,0832	1,4456
1416885_at	1110038F14Rik	RIKEN cDNA 1110038F14 gene	2,7126	0,0833	1,1464
1425810_a_at	Csrp1	cysteine and glycine-rich protein 1	2,7110	0,0833	1,2408
1420004_s_at	NULL	NULL	2,7103	0,0833	1,2589
1417954_at	Sst	somatostatin	2,7100	0,0833	1,4063
1460707_at	Ptp4a2	protein tyrosine phosphatase 4a2	2,7098	0,0833	1,5259
1455904_at	Gas5	growth arrest specific 5	2,7095	0,0833	1,3313
1434229_a_at	Polb	polymerase (DNA directed), beta	2,7086	0,0833	1,3753
1449622_s_at	Atp6ap1	ATPase, H+ transporting, lysosomal accessory protein 1	2,7084	0,0833	1,4734
1459885_s_at	NULL	NULL	2,7079	0,0833	1,1736
1426831_at	Ahcyl1	S-adenosylhomocysteine hydrolase-like 1	2,7075	0,0833	1,8140
1426644_at	Tbc1d20	TBC1 domain family, member 20	2,7068	0,0833	1,1733

Probeset ID	Symbol	Gene Name	d-value	q-value	R-fold
1451720_at	Vps39	vacuolar protein sorting 39 (yeast)	2,7067	0,0833	1,5731
1448835_at	E2f6	E2F transcription factor 6	2,7044	0,0833	1,2586
1451253_at	Pxk	PX domain containing serine/threonine kinase	2,7044	0,0833	1,6626
1422578_at	Cs	citrate synthase	2,7031	0,0833	1,4038
1460544_at	Naa35	N(alpha)-acetyltransferase 35, NatC auxiliary subunit	2,7030	0,0833	1,6272
1450894_a_at	Ap2m1	adaptor protein complex AP-2, mu1	2,7025	0,0833	1,2655
1448128_at	Ctsa	cathepsin A	2,7025	0,0833	1,5102
1438370_x_at	Dos	downstream of Stk11	2,7024	0,0833	1,3824
1423151_at	Dnajb11	DnaJ (Hsp40) homolog, subfamily B, member 11	2,7021	0,0833	1,2838
1416096_at	Vipar	VPS33B interacting protein, apical-basolateral polarity regulator	2,7018	0,0833	1,2804
1433486_at	Clcn3	chloride channel 3	2,7014	0,0833	1,7780
1421874_a_at	Mrps23	mitochondrial ribosomal protein S23	2,7011	0,0833	1,4127
1426543_x_at	Endod1	endonuclease domain containing 1	2,7009	0,0833	1,2796
1417683_at	Diablo	diablo homolog (Drosophila)	2,7005	0,0834	1,4195
1431645_a_at	Gdi2	guanosine diphosphate (GDP) dissociation inhibitor 2	2,7004	0,0834	1,9033
1422901_at	Mgea5	meningioma expressed antigen 5 (hyaluronidase)	2,6998	0,0834	1,6468
1437143_a_at	Tmx1	thioredoxin-related transmembrane protein 1	2,6997	0,0834	1,6140
1418226_at	Orc2l	origin recognition complex, subunit 2-like (S. cerevisiae)	2,6990	0,0834	1,1073
1434604_at	Eif5b	eukaryotic translation initiation factor 5B	2,6986	0,0834	1,3192
1452293_at	Snx19	sorting nexin 19	2,6986	0,0834	1,3200
1439103_at	Cdc73	cell division cycle 73, Paf1/RNA polymerase II complex component, homolog (S. cerevisiae)	2,6978	0,0834	1,1291
1434037_s_at	Kat2b	K(lysine) acetyltransferase 2B	2,6972	0,0834	1,7617
1415685_at	Mtif2	mitochondrial translational initiation factor 2	2,6965	0,0834	1,6688
1452051_at	Actr3	ARP3 actin-related protein 3 homolog (yeast)	2,6964	0,0834	1,6980
1448021_at	NULL	NULL	2,6962	0,0834	1,8780
1416009_at	Tspan3	tetraspanin 3	2,6959	0,0834	1,5331
1416213_x_at	Surf4	surfeit gene 4	2,6949	0,0834	1,4799
1438264_a_at	Tpp2	tripeptidyl peptidase II	2,6947	0,0834	1,4515
1422453_at	Prpf8	pre-mRNA processing factor 8	2,6947	0,0834	1,5344
1424018_at	Hint1	histidine triad nucleotide binding protein 1	2,6931	0,0834	1,2959
1415757_at	Gbf1	golgi-specific brefeldin A-resistance factor 1	2,6925	0,0834	1,6937
1416907_at	Tsn	translin	2,6925	0,0834	1,6875
1424084_at	Rod1	ROD1 regulator of differentiation 1 (S. pombe)	2,6924	0,0834	1,2261
1435019_at	Atxn7l3	ataxin 7-like 3	2,6894	0,0834	1,3184
1435444_at	Atf6	activating transcription factor 6	2,6892	0,0834	1,6378
1434575_at	Epb4.1l1	erythrocyte protein band 4.1-like 1	2,6890	0,0834	1,3834
1419011_at	Cryba2	crystallin, beta A2	2,6889	0,0834	1,1523
1436884_x_at	Ewsr1	Ewing sarcoma breakpoint region 1	2,6887	0,0834	1,5154
1460315_s_at	Tbk1	TANK-binding kinase 1	2,6886	0,0834	1,6178
1434972_x_at	NULL	NULL	2,6881	0,0834	2,2611
1451221_at	BC018507	cDNA sequence BC018507	2,6880	0,0834	1,2734
1423050_s_at	Hnrnpu	heterogeneous nuclear ribonucleoprotein U	2,6879	0,0834	1,5769
1449823_at	Dach2	dachshund 2 (Drosophila)	2,6879	0,0834	1,1024
1419254_at	Mthfd2	methylenetetrahydrofolate dehydrogenase (NAD+ dependent), methenyltetrahydrofolate cyclohydrolase	2,6877	0,0834	2,0720
1434396_a_at	NULL	NULL	2,6876	0,0834	1,2851
1415794_a_at	Spin1	spindlin 1	2,6872	0,0834	1,5572
1417948_s_at	Ilf2	interleukin enhancer binding factor 2	2,6870	0,0834	1,3307
1438631_x_at	Ttc13	tetratricopeptide repeat domain 13	2,6867	0,0834	1,9117
1428073_a_at	Nup88	nucleoporin 88	2,6863	0,0834	1,2951
1437843_s_at	Nupl1	nucleoporin like 1	2,6860	0,0834	1,8144
1452120_at	Naa40	N(alpha)-acetyltransferase 40, NatD catalytic subunit, homolog (S. cerevisiae)	2,6856	0,0834	1,2430
1423147_at	Mat1a	methionine adenosyltransferase I, alpha	2,6856	0,0834	1,3241
1426683_at	Cnot6	CCR4-NOT transcription complex, subunit 6	2,6854	0,0834	1,3936
1436498_at	Arih1	ariadne ubiquitin-conjugating enzyme E2 binding protein homolog 1 (Drosophila)	2,6854	0,0834	1,6844
1421063_s_at	NULL	NULL	2,6841	0,0834	1,4195
1430172_a_at	NULL	NULL	2,6835	0,0834	1,1811
1435559_at	Myo6	myosin VI	2,6827	0,0834	1,4918
1438034_at	Fam82b	family with sequence similarity 82, member B	2,6812	0,0835	1,2588
1422480_at	Snx3	sorting nexin 3	2,6811	0,0835	1,3196
1420500_at	Dnajc1	DnaJ (Hsp40) homolog, subfamily C, member 1	2,6809	0,0835	1,5258
1419238_at	Abca7	ATP-binding cassette, sub-family A (ABC1), member 7	2,6807	0,0835	1,4172
1452146_a_at	Cox15	COX15 homolog, cytochrome c oxidase assembly protein (yeast)	2,6806	0,0835	1,2172
1422103_a_at	Stat5b	signal transducer and activator of transcription 5B	2,6802	0,0835	1,7484
1417539_at	NULL	NULL	2,6794	0,0835	1,5582
1426375_s_at	Oxnad1	oxidoreductase NAD-binding domain containing 1	2,6794	0,0835	1,2906
1422487_at	Smad4	MAD homolog 4 (Drosophila)	2,6788	0,0835	1,9615
1417113_at	Gmcl1	germ cell-less homolog 1 (Drosophila)	2,6787	0,0835	1,2363
1428082_at	Acsl5	acyl-CoA synthetase long-chain family member 5	2,6781	0,0835	1,2738
1423513_at	NULL	NULL	2,6765	0,0836	1,4851
1421118_a_at	Gpr56	G protein-coupled receptor 56	2,6761	0,0837	1,5388
1431771_a_at	Irak1bp1	interleukin-1 receptor-associated kinase 1 binding protein 1	2,6760	0,0837	1,3349
1435272_at	Itpkb	inositol 1,4,5-trisphosphate 3-kinase B	2,6734	0,0837	1,3684
1419287_at	Tmem208	transmembrane protein 208	2,6707	0,0837	1,4138
1452090_a_at	Olfm3	olfactomedin 3	2,6707	0,0837	1,1130
1452350_at	Brd8	bromodomain containing 8	2,6706	0,0837	1,1461

Probeset ID	Symbol	Gene Name	d-value	q-value	R-fold
1417453_at	Cul4b	cullin 4B	2,6703	0,0837	1,5857
1460314_s_at	NULL	NULL	2,6697	0,0837	1,5383
1426061_x_at	NULL	NULL	2,6697	0,0837	1,3676
1418430_at	Kif5b	kinesin family member 5B	2,6695	0,0837	1,6970
1451896_a_at	Cherp	calcium homeostasis endoplasmic reticulum protein	2,6691	0,0837	1,6396
1417820_at	Tor1b	torsin family 1, member B	2,6687	0,0837	1,4272
1451276_at	Uhrf1bp1l	UHRF1 (ICBP90) binding protein 1-like	2,6686	0,0837	1,5193
1451451_at	Gca	grancalcin	2,6682	0,0837	1,2508
1437837_x_at	Poldip3	polymerase (DNA-directed), delta interacting protein 3	2,6672	0,0837	1,9952
1418198_a_at	Tm9sf1	transmembrane 9 superfamily member 1	2,6658	0,0837	1,6537
1419819_s_at	Sec63	SEC63-like (S. cerevisiae)	2,6649	0,0838	1,3768
1417108_at	Klc4	kinesin light chain 4	2,6646	0,0838	1,1642
1436048_at	Exoc8	exocyst complex component 8	2,6645	0,0838	1,3705
1426977_at	Usp47	ubiquitin specific peptidase 47	2,6636	0,0838	1,4688
1417361_at	Asb3	ankyrin repeat and SOCS box-containing 3	2,6630	0,0838	1,4960
1423899_at	Trip12	thyroid hormone receptor interactor 12	2,6625	0,0838	1,4266
1452925_a_at	March5	membrane-associated ring finger (C3HC4) 5	2,6622	0,0838	1,4598
1452621_at	Pcbd2	pterin 4 alpha carbinolamine dehydratase/dimerization cofactor of hepatocyte nuclear factor 1 alpha (TCF1) 2	2,6620	0,0838	1,3978
1418008_at	Gcfc1	GC-rich sequence DNA-binding factor 1	2,6614	0,0838	1,3681
1447977_x_at	NULL	NULL	2,6606	0,0838	1,4636
1456056_a_at	D6Wsu116e	DNA segment, Chr 6, Wayne State University 116, expressed	2,6599	0,0838	1,9243
1423095_s_at	Crbn	cereblon	2,6590	0,0838	1,5216
1451251_at	Appbp2	amyloid beta precursor protein (cytoplasmic tail) binding protein 2	2,6574	0,0839	1,7807
1419927_s_at	Rabif	RAB interacting factor	2,6566	0,0839	1,7263
1437687_x_at	Fkbp9	FK506 binding protein 9	2,6558	0,0839	1,5302
1452026_a_at	Pla2g12a	phospholipase A2, group XIIA	2,6557	0,0839	1,1431
1432094_a_at	Ccdc132	coiled-coil domain containing 132	2,6557	0,0839	1,5101
1456577_x_at	Pitrm1	pitrilysin metallepetidase 1	2,6557	0,0839	1,1900
1425245_a_at	Rgs11	regulator of G-protein signaling 11	2,6545	0,0839	1,8850
1448578_at	Pafah1b1	platelet-activating factor acetylhydrolase, isoform 1b, subunit 1	2,6543	0,0839	1,6641
1451018_at	Leprotl1	leptin receptor overlapping transcript-like 1	2,6537	0,0840	1,6898
1450942_at	Zfp830	zinc finger protein 830	2,6525	0,0840	1,1562
1451144_at	Brix1	BRX1, biogenesis of ribosomes, homolog (S. cerevisiae)	2,6524	0,0840	1,7323
1435981_at	Nav2	neuron navigator 2	2,6518	0,0840	1,6360
1438164_x_at	Flot2	flotillin 2	2,6509	0,0840	2,3286
1416336_s_at	Snrpd1	small nuclear ribonucleoprotein D1	2,6504	0,0840	1,5008
1452506_a_at	Ilkap	integrin-linked kinase-associated serine/threonine phosphatase 2C	2,6500	0,0840	1,4838
1423724_at	Zwint	ZW10 interactor	2,6494	0,0840	1,4616
1435530_at	Camsap1	calmodulin regulated spectrin-associated protein 1	2,6491	0,0840	1,2873
1449376_at	Nicn1	nicolin 1	2,6487	0,0840	1,4995
1423615_at	Rnf115	ring finger protein 115	2,6474	0,0840	1,4836
1424768_at	Cald1	caldesmon 1	2,6471	0,0840	1,9167
1424873_at	Rnf2	ring finger protein 2	2,6470	0,0840	1,7967
1415764_at	Zc3h11a	zinc finger CCCH type containing 11A	2,6463	0,0840	1,7790
1435669_at	Zfp532	zinc finger protein 532	2,6463	0,0840	1,5180
1433443_a_at	Hmgcs1	3-hydroxy-3-methylglutaryl-Coenzyme A synthase 1	2,6460	0,0840	1,5853
1438369_x_at	NULL	NULL	2,6458	0,0841	1,2313
1423917_a_at	Cttn	cortactin	2,6456	0,0841	1,3962
1423997_at	Csde1	cold shock domain containing E1, RNA binding	2,6455	0,0841	1,8522
1423850_at	Nsun2	NOL1/NOP2/Sun domain family member 2	2,6449	0,0841	1,2969
1426830_a_at	Ahcyl1	S-adenosylhomocysteine hydrolase-like 1	2,6444	0,0841	1,4409
1420953_at	Add1	adducin 1 (alpha)	2,6429	0,0841	1,1859
1452116_s_at	Atf2	activating transcription factor 2	2,6428	0,0841	2,2584
1415771_at	Ncl	nucleolin	2,6424	0,0841	2,0484
1434445_at	Arhgap39	Rho GTPase activating protein 39	2,6408	0,0842	1,1163
1448399_at	Tax1bp1	Tax1 (human T-cell leukemia virus type I) binding protein 1	2,6402	0,0842	1,6207
1424244_at	Rwdd4a	RWD domain containing 4A	2,6400	0,0842	1,4177
1452240_at	Celf4	CUGBP, Elav-like family member 4	2,6399	0,0842	1,8605
1454736_at	Ankrd57	ankyrin repeat domain 57	2,6397	0,0842	1,4168
1460716_a_at	Cbfb	core binding factor beta	2,6396	0,0842	1,2312
1417041_at	Polr1c	polymerase (RNA) I polypeptide C	2,6384	0,0842	1,3381
1423117_at	Pum1	pumilio 1 (Drosophila)	2,6382	0,0842	1,9894
1451575_a_at	NULL	NULL	2,6382	0,0842	1,5327
1425497_a_at	Prpf4b	PRP4 pre-mRNA processing factor 4 homolog B (yeast)	2,6381	0,0842	1,7963
1426692_at	Ccdc97	coiled-coil domain containing 97	2,6352	0,0843	1,2627
1448516_at	Tsn	translin	2,6330	0,0843	1,2825
1415920_at	Cstf2t	cleavage stimulation factor, 3' pre-RNA subunit 2, tau	2,6322	0,0843	2,2725
1457272_at	NULL	NULL	2,6320	0,0843	1,2237
1451053_a_at	Mdm1	transformed mouse 3T3 cell double minute 1	2,6319	0,0843	2,2560
1452196_a_at	Nckap1	NCK-associated protein 1	2,6316	0,0843	1,4559
1450714_at	Azin1	antizyme inhibitor 1	2,6314	0,0843	1,9432
1418932_at	NULL	NULL	2,6309	0,0843	1,4745
1448026_at	Chd7	chromodomain helicase DNA binding protein 7	2,6307	0,0843	1,3959
1452830_s_at	Cad	carbamoyl-phosphate synthetase 2, aspartate transcarbamylase, and dihydroorotase	2,6298	0,0843	1,5521
1449064_at	Tdh	L-threonine dehydrogenase	-2,6260	0,0845	0,7652
1450346_at	Gpr50	G-protein-coupled receptor 50	-2,6271	0,0844	0,8202
1420664_s_at	Procr	protein C receptor, endothelial	-2,6272	0,0844	0,9042

Probeset ID	Symbol	Gene Name	d-value	q-value	R-fold
1431609_a_at	Acp5	acid phosphatase 5, tartrate resistant	-2,6278	0,0844	0,7217
1425393_a_at	Map2k7	mitogen-activated protein kinase kinase 7	-2,6284	0,0844	0,6994
1435804_at	Eif4e2	eukaryotic translation initiation factor 4E member 2	-2,6289	0,0843	0,7951
1427519_at	Adora2a	adenosine A2a receptor	-2,6290	0,0843	0,7069
1449918_at	Cd209g	CD209g antigen	-2,6290	0,0843	0,7371
1425253_a_at	Madcam1	mucosal vascular addressin cell adhesion molecule 1	-2,6298	0,0843	0,7515
1451268_at	Tram1l1	translocation associated membrane protein 1-like 1	-2,6301	0,0843	0,8521
1449917_at	Pitx3	paired-like homeodomain transcription factor 3	-2,6306	0,0843	0,6599
1435323_a_at	Mboat1	membrane bound O-acyltransferase domain containing 1	-2,6310	0,0843	0,8853
1415921_a_at	Tnfrsf19	tumor necrosis factor receptor superfamily, member 19	-2,6311	0,0843	0,7170
1418599_at	Col11a1	collagen, type XI, alpha 1	-2,6319	0,0843	0,9177
1437379_x_at	Trap1	TNF receptor-associated protein 1	-2,6328	0,0843	0,7474
1420272_at	Samhd1	SAM domain and HD domain, 1	-2,6331	0,0843	0,6060
1421497_at	Gpha2	glycoprotein hormone alpha 2	-2,6337	0,0843	0,7299
1419720_at	Gp9	glycoprotein 9 (platelet)	-2,6342	0,0843	0,7714
1460383_at	Gnao1	guanine nucleotide binding protein, alpha O	-2,6342	0,0843	0,7622
1418098_at	Adcy4	adenylate cyclase 4	-2,6359	0,0842	0,7011
1449996_a_at	Tpm3	tropomyosin 3, gamma	-2,6361	0,0842	0,6782
1451697_a_at	Evc	Ellis van Creveld gene homolog (human)	-2,6361	0,0842	0,6895
1451636_at	Ankrd33	ankyrin repeat domain 33	-2,6367	0,0842	0,7292
1431808_a_at	Itih4	inter alpha-trypsin inhibitor, heavy chain 4	-2,6368	0,0842	0,6932
1425403_at	Dnm3	dynamin 3	-2,6372	0,0842	0,7300
1452846_at	Ppfia4	protein tyrosine phosphatase, receptor type, f polypeptide (PTPRF), interacting protein (liprin), alpha 4	-2,6379	0,0842	0,5956
1418293_at	Ifit2	interferon-induced protein with tetratricopeptide repeats 2	-2,6381	0,0842	0,8662
1427139_at	Adamts10	a disintegrin-like and metallopeptidase (reprolysin type) with thrombospondin type 1 motif, 10	-2,6385	0,0842	0,6381
1438446_x_at	Inpp5k	inositol polyphosphate 5-phosphatase K	-2,6386	0,0842	0,7935
1453249_a_at	NULL	NULL	-2,6388	0,0842	0,8468
1425355_at	Acsf2	acyl-CoA synthetase family member 2	-2,6392	0,0842	0,7613
1419628_at	Vsx2	visual system homeobox 2	-2,6395	0,0842	0,8381
1460713_at	BC048355	cDNA sequence BC048355	-2,6417	0,0841	0,6588
1449826_a_at	Fgf2	fibroblast growth factor 2	-2,6418	0,0841	0,9336
1438743_at	Cyp7a1	cytochrome P450, family 7, subfamily a, polypeptide 1	-2,6420	0,0841	0,8501
1435733_x_at	Rnaseh2c	ribonuclease H2, subunit C	-2,6421	0,0841	0,8174
1419284_at	Cyhr1	cysteine and histidine rich 1	-2,6431	0,0841	0,8242
1423954_at	C3	complement component 3	-2,6433	0,0841	0,7782
1452415_at	Actn1	actinin, alpha 1	-2,6434	0,0841	0,8784
1451637_a_at	Prol1	proline rich, lacrimal 1	-2,6438	0,0841	0,8207
1431124_at	Auh	AU RNA binding protein/enoyl-coenzyme A hydratase	-2,6441	0,0841	0,7499
1423327_at	Rpl39l	ribosomal protein L39-like	-2,6446	0,0841	0,8944
1419774_at	D2Ertd239e	DNA segment, Chr 2, ERATO Doi 239, expressed	-2,6446	0,0841	0,7793
1420289_at	NULL	NULL	-2,6452	0,0841	0,8345
1432453_a_at	Ms4a10	membrane-spanning 4-domains, subfamily A, member 10	-2,6465	0,0840	0,6309
1448914_a_at	Csf1	colony stimulating factor 1 (macrophage)	-2,6468	0,0840	0,6586
1455424_at	Wnk2	WNK lysine deficient protein kinase 2	-2,6478	0,0840	0,9301
1426638_at	Six3	sine oculis-related homeobox 3 homolog (Drosophila)	-2,6478	0,0840	0,8760
1420534_at	Gucy1a3	guanylate cyclase 1, soluble, alpha 3	-2,6492	0,0840	0,8360
1452028_a_at	Cdh23	cadherin 23 (otocadherin)	-2,6494	0,0840	0,5884
1416900_s_at	NULL	NULL	-2,6497	0,0840	0,5706
1425277_at	Slit1	slit homolog 1 (Drosophila)	-2,6499	0,0840	0,7561
1448544_at	Crat	carnitine acetyltransferase	-2,6507	0,0840	0,7453
1419600_at	Defb4	defensin beta 4	-2,6509	0,0840	0,8930
1421338_at	NULL	NULL	-2,6516	0,0840	0,8612
1418818_at	NULL	NULL	-2,6518	0,0840	0,7572
1420348_at	Lhx5	LIM homeobox protein 5	-2,6521	0,0840	0,7123
1417309_at	Tob2	transducer of ERBB2, 2	-2,6524	0,0840	0,7447
1454369_a_at	Nfatc4	nuclear factor of activated T-cells, cytoplasmic, calcineurin-dependent 4	-2,6525	0,0840	0,7119
1419424_at	Ptf1a	pancreas specific transcription factor, 1a	-2,6526	0,0840	0,6558
1418385_at	Cuedc2	CUE domain containing 2	-2,6532	0,0840	0,8263
1421959_s_at	Adcy3	adenylate cyclase 3	-2,6534	0,0840	0,7026
1452566_at	NULL	NULL	-2,6537	0,0840	0,6122
1448152_at	Igf2	insulin-like growth factor 2	-2,6547	0,0839	0,7185
1432821_at	Nfatc4	nuclear factor of activated T-cells, cytoplasmic, calcineurin-dependent 4	-2,6550	0,0839	0,7462
1460730_at	Eif2b1	eukaryotic translation initiation factor 2B, subunit 1 (alpha)	-2,6550	0,0839	0,7371
1439370_x_at	Sf3b5	splicing factor 3b, subunit 5	-2,6551	0,0839	0,6832
1437841_x_at	Csdc2	cold shock domain containing C2, RNA binding	-2,6564	0,0839	0,6343
1450796_at	Atoh7	atonal homolog 7 (Drosophila)	-2,6566	0,0839	0,6740
1439323_a_at	Map4k1	mitogen-activated protein kinase kinase kinase kinase 1	-2,6568	0,0839	0,7650
1419965_at	NULL	NULL	-2,6574	0,0839	0,8018
1417021_a_at	Spo11	sporulation protein, meiosis-specific, SPO11 homolog (S. cerevisiae)	-2,6590	0,0838	0,9201
1422228_at	Wnt8a	wingless-related MMTV integration site 8A	-2,6591	0,0838	0,6533
1417304_at	Chrd	chordin	-2,6591	0,0838	0,6329
1450594_at	Magea4	melanoma antigen, family A, 4	-2,6595	0,0838	0,8646
1436880_at	Afp	alpha fetoprotein	-2,6595	0,0838	0,7497
1448257_at	Slc29a2	solute carrier family 29 (nucleoside transporters), member 2	-2,6599	0,0838	0,6871
1421072_at	Irx5	Iroquois related homeobox 5 (Drosophila)	-2,6606	0,0838	0,8536
1421364_at	Lrfn1	leucine rich repeat and fibronectin type III domain containing 1	-2,6607	0,0838	0,7816
1426520_at	Btg4	B-cell translocation gene 4	-2,6612	0,0838	0,7236

Probeset ID	Symbol	Gene Name	d-value	q-value	R-fold
1459085_at	D1Ertd259e	DNA segment, Chr 1, ERATO Doi 259, expressed	-2,6612	0,0838	0,8190
1451336_at	Lgals4	lectin, galactose binding, soluble 4	-2,6612	0,0838	0,8824
1425950_at	Slc17a9	solute carrier family 17, member 9	-2,6622	0,0838	0,8413
1448426_at	Sardh	sarcosine dehydrogenase	-2,6622	0,0838	0,8502
1450802_at	Prss28	protease, serine, 28	-2,6624	0,0838	0,8308
1420045_at	NULL	NULL	-2,6628	0,0838	0,8103
1427611_at	Ghrhr	growth hormone releasing hormone receptor	-2,6632	0,0838	0,7302
1426664_x_at	Slc45a3	solute carrier family 45, member 3	-2,6636	0,0838	0,6644
1420301_at	AA414903	expressed sequence AA414903	-2,6642	0,0838	0,6360
1450060_at	Pigr	polymeric immunoglobulin receptor	-2,6653	0,0838	0,8787
1420026_at	Aak1	AP2 associated kinase 1	-2,6657	0,0837	0,8013
1425655_at	Krtap16-1	keratin associated protein 16-1	-2,6660	0,0837	0,8473
1422790_at	Nppc	natriuretic peptide precursor type C	-2,6661	0,0837	0,6944
1449572_at	Trhr	thyrotropin releasing hormone receptor	-2,6663	0,0837	0,7699
1428635_at	Comtd1	catechol-O-methyltransferase domain containing 1	-2,6667	0,0837	0,7430
1460256_at	Car3	carbonic anhydrase 3	-2,6693	0,0837	0,7633
1450357_a_at	Ccr6	chemokine (C-C motif) receptor 6	-2,6696	0,0837	0,7072
1449883_at	Fxyd2	FXYD domain-containing ion transport regulator 2	-2,6698	0,0837	0,7285
1452001_at	Nfe2	nuclear factor, erythroid derived 2	-2,6705	0,0837	0,8021
1448840_at	Tmub1	transmembrane and ubiquitin-like domain containing 1	-2,6706	0,0837	0,8265
1449499_at	Hoxa7	homeobox A7	-2,6707	0,0837	0,5785
1448090_at	NULL	NULL	-2,6710	0,0837	0,8681
1452091_a_at	Rbm28	RNA binding motif protein 28	-2,6714	0,0837	0,7393
1419135_at	Ltb	lymphotoxin B	-2,6714	0,0837	0,7478
1417931_at	Ndst2	N-deacetylase/N-sulfotransferase (heparan glucosaminyl) 2	-2,6716	0,0837	0,7565
1420848_at	Sufu	suppressor of fused homolog (Drosophila)	-2,6719	0,0837	0,8478
1427840_at	NULL	NULL	-2,6725	0,0837	0,7569
1459546_s_at	Enpp1	ectonucleotide pyrophosphatase/phosphodiesterase 1	-2,6733	0,0837	0,7954
1421001_a_at	Car6	carbonic anhydrase 6	-2,6735	0,0837	0,8373
1420063_at	NULL	NULL	-2,6736	0,0837	0,7557
1422372_at	Olfr15	olfactory receptor 15	-2,6739	0,0837	0,8478
1448251_at	9030425E11Rik	RIKEN cDNA 9030425E11 gene	-2,6745	0,0837	0,8829
1422226_at	NULL	NULL	-2,6753	0,0837	0,8213
1430293_a_at	NULL	NULL	-2,6755	0,0837	0,8530
1431630_a_at	NULL	NULL	-2,6756	0,0837	0,6265
1422254_a_at	Dyrk1b	dual-specificity tyrosine-(Y)-phosphorylation regulated kinase 1b	-2,6756	0,0837	0,5373
1426639_a_at	Tcf7l2	transcription factor 7-like 2, T-cell specific, HMG-box	-2,6758	0,0837	0,6785
1418550_x_at	Defa-rs1	defensin, alpha, related sequence 1	-2,6766	0,0836	0,7411
1416523_at	Rnase1	ribonuclease, RNase A family, 1 (pancreatic)	-2,6767	0,0836	0,8439
1420442_at	Cacna1s	calcium channel, voltage-dependent, L type, alpha 1S subunit	-2,6768	0,0836	0,6992
1448499_a_at	Ephx2	epoxide hydrolase 2, cytoplasmic	-2,6781	0,0835	0,8567
1419718_at	4921530L21Rik	RIKEN cDNA 4921530L21 gene	-2,6782	0,0835	0,7887
1451371_at	Mrap	melanocortin 2 receptor accessory protein	-2,6782	0,0835	0,6095
1437294_at	Exosc10	exosome component 10	-2,6784	0,0835	0,7599
1450651_at	Myo10	myosin X	-2,6785	0,0835	0,8259
1422757_at	Slc5a4b	solute carrier family 5 (neutral amino acid transporters, system A), member 4b	-2,6790	0,0835	0,8705
1438245_at	Nfib	nuclear factor I/B	-2,6791	0,0835	0,9250
1425570_at	Slamf1	signaling lymphocytic activation molecule family member 1	-2,6791	0,0835	0,8070
1421361_at	NULL	NULL	-2,6797	0,0835	0,6886
1419473_a_at	Cck	cholecystokinin	-2,6798	0,0835	0,8505
1420055_at	Slc35c2	solute carrier family 35, member C2	-2,6802	0,0835	0,7275
1448329_at	Adam3	a disintegrin and metallopeptidase domain 3 (cyritestin)	-2,6802	0,0835	0,6715
1416995_at	Pacsin3	protein kinase C and casein kinase substrate in neurons 3	-2,6804	0,0835	0,6520
1422878_at	Syt12	synaptotagmin XII	-2,6805	0,0835	0,6948
1433964_s_at	Fermt3	fermitin family homolog 3 (Drosophila)	-2,6816	0,0835	0,7013
1418289_at	Nes	nestin	-2,6817	0,0835	0,6829
1421371_at	NULL	NULL	-2,6817	0,0835	0,7363
1426505_at	NULL	NULL	-2,6820	0,0835	0,7898
1427732_s_at	Abcg4	ATP-binding cassette, sub-family G (WHITE), member 4	-2,6833	0,0834	0,6333
1460613_x_at	Gh	growth hormone	-2,6833	0,0834	0,5955
1422148_at	Matn3	matrilin 3	-2,6839	0,0834	0,7391
1419960_at	Cphx	cytoplasmic polyadenylated homeobox	-2,6843	0,0834	0,6010
1417178_at	Gipc2	GIPC PDZ domain containing family, member 2	-2,6844	0,0834	0,7928
1421981_at	Kcnc3	potassium voltage gated channel, Shaw-related subfamily, member 3	-2,6852	0,0834	0,6342
1425417_x_at	Klra8	killer cell lectin-like receptor, subfamily A, member 8	-2,6853	0,0834	0,7914
1431791_a_at	Ptpn13	protein tyrosine phosphatase, non-receptor type 13	-2,6855	0,0834	0,7512
1448197_at	Ceacam11	carcinoembryonic antigen-related cell adhesion molecule 11	-2,6862	0,0834	0,8453
1419708_at	Wnt6	wingless-related MMTV integration site 6	-2,6862	0,0834	0,7600
1426391_at	NULL	NULL	-2,6868	0,0834	0,8539
1451949_at	NULL	NULL	-2,6875	0,0834	0,7831
1420973_at	NULL	NULL	-2,6878	0,0834	0,7909
1420359_at	Sva	seminal vesicle antigen	-2,6880	0,0834	0,9344
1426091_a_at	Wnt10b	wingless related MMTV integration site 10b	-2,6891	0,0834	0,7436
1420160_s_at	Myo1e	myosin IE	-2,6892	0,0834	0,7690
1420263_at	NULL	NULL	-2,6892	0,0834	0,6711
1437568_at	Mmp16	matrix metallopeptidase 16	-2,6899	0,0834	0,8257
1422007_at	Aqp3	aquaporin 3	-2,6909	0,0834	0,7010
1425959_x_at	Klra16	killer cell lectin-like receptor, subfamily A, member 16	-2,6911	0,0834	0,8485

Probeset ID	***Symbol***	***Gene Name***	***d-value***	***q-value***	***R-fold***
1451702_at	Cmtm7	CKLF-like MARVEL transmembrane domain containing 7	-2,6913	0,0834	0,8082
1421742_at	Gdi2	guanosine diphosphate (GDP) dissociation inhibitor 2	-2,6915	0,0834	0,8492
1424959_at	Anxa13	annexin A13	-2,6916	0,0834	0,8715
1450177_at	Ngfr	nerve growth factor receptor (TNFR superfamily, member 16)	-2,6916	0,0834	0,7392
1424795_a_at	1700001O22Rik	RIKEN cDNA 1700001O22 gene	-2,6926	0,0834	0,6410
1421239_at	Il6st	interleukin 6 signal transducer	-2,6930	0,0834	0,8250
1422379_x_at	Vmn1r41	vomeronasal 1 receptor 41	-2,6935	0,0834	0,8376
1424105_a_at	Pttg1	pituitary tumor-transforming gene 1	-2,6944	0,0834	0,5915
1430197_a_at	Pitpnm2	phosphatidylinositol transfer protein, membrane-associated 2	-2,6946	0,0834	0,6283
1428477_at	Elac2	elaC homolog 2 (E. coli)	-2,6955	0,0834	0,8415
1437691_at	Anxa2	annexin A2	-2,6966	0,0834	0,8529
1426658_x_at	Phgdh	3-phosphoglycerate dehydrogenase	-2,6970	0,0834	0,7498
1428612_at	Atg7	autophagy-related 7 (yeast)	-2,6971	0,0834	0,8310
1422160_at	H2-T24	histocompatibility 2, T region locus 24	-2,6972	0,0834	0,8172
1422512_a_at	Ogfr	opioid growth factor receptor	-2,6975	0,0834	0,7850
1417305_at	Speg	SPEG complex locus	-2,6976	0,0834	0,8423
1460311_at	Srst	simple repeat sequence-containing transcript	-2,6976	0,0834	0,8874
1450486_a_at	Oprl1	opioid receptor-like 1	-2,6977	0,0834	0,7461
1417382_at	Entpd5	ectonucleoside triphosphate diphosphohydrolase 5	-2,6985	0,0834	0,6678
1421244_at	Esr1	estrogen receptor 1 (alpha)	-2,6993	0,0834	0,7053
1451228_a_at	Sycn	syncollin	-2,7002	0,0834	0,7234
1421753_a_at	Lhx3	LIM homeobox protein 3	-2,7006	0,0834	0,5952
1452337_at	4930427A07Rik	RIKEN cDNA 4930427A07 gene	-2,7013	0,0833	0,6988
1428123_at	Fam125b	family with sequence similarity 125, member B	-2,7014	0,0833	0,7309
1423561_at	Nell2	NEL-like 2 (chicken)	-2,7025	0,0833	0,7962
1417601_at	Rgs1	regulator of G-protein signaling 1	-2,7027	0,0833	0,7700
1448456_at	Cln8	ceroid-lipofuscinosis, neuronal 8	-2,7031	0,0833	0,8238
1460289_at	Nrg3	neuregulin 3	-2,7032	0,0833	0,8419
1460382_at	BC020535	cDNA sequence BC020535	-2,7037	0,0833	0,7246
1419304_at	T	brachyury	-2,7046	0,0833	0,9124
1420197_at	Gadd45b	growth arrest and DNA-damage-inducible 45 beta	-2,7049	0,0833	0,7330
1417524_at	NULL	NULL	-2,7050	0,0833	0,7068
1448288_at	Nfib	nuclear factor I/B	-2,7062	0,0833	0,8747
1450524_at	Cldn9	claudin 9	-2,7064	0,0833	0,6697
1425819_at	Zbtb7c	zinc finger and BTB domain containing 7C	-2,7064	0,0833	0,6149
1427592_at	Pcdh7	protocadherin 7	-2,7067	0,0833	0,7165
1457147_at	Etl4	enhancer trap locus 4	-2,7069	0,0833	0,7617
1417838_at	NULL	NULL	-2,7070	0,0833	0,6642
1416178_a_at	Plekhb1	pleckstrin homology domain containing, family B (evectins) member 1	-2,7074	0,0833	0,8710
1435782_at	Gm12617	Uba52 pseudogene	-2,7075	0,0833	0,7690
1415843_at	Mlst8	MTOR associated protein, LST8 homolog (S. cerevisiae)	-2,7081	0,0833	0,8314
1425849_at	Chrnb4	cholinergic receptor, nicotinic, beta polypeptide 4	-2,7082	0,0833	0,7236
1452546_x_at	Defb11	defensin beta 11	-2,7090	0,0833	0,9450
1452622_a_at	Tradd	TNFRSF1A-associated via death domain	-2,7092	0,0833	0,8278
1450621_a_at	Hbb-y	hemoglobin Y, beta-like embryonic chain	-2,7094	0,0833	0,8288
1449074_at	1700019N12Rik	RIKEN cDNA 1700019N12 gene	-2,7098	0,0833	0,6906
1425180_at	Sgip1	SH3-domain GRB2-like (endophilin) interacting protein 1	-2,7098	0,0833	0,9350
1425953_at	NULL	NULL	-2,7109	0,0833	0,8120
1451517_at	Rhobtb2	Rho-related BTB domain containing 2	-2,7110	0,0833	0,7952
1449920_at	Cyp19a1	cytochrome P450, family 19, subfamily a, polypeptide 1	-2,7121	0,0833	0,7773
1427652_x_at	Synj2	synaptojanin 2	-2,7125	0,0833	0,8589
1424761_at	Fam115c	family with sequence similarity 115, member C	-2,7134	0,0832	0,7989
1419498_at	Tmigd1	transmembrane and immunoglobulin domain containing 1	-2,7135	0,0832	0,9215
1428040_at	Onecut3	one cut domain, family member 3	-2,7137	0,0832	0,7844
1449623_at	Txnrd3	thioredoxin reductase 3	-2,7140	0,0832	0,7005
1426164_a_at	Usf1	upstream transcription factor 1	-2,7140	0,0832	0,7848
1416740_at	Col5a1	collagen, type V, alpha 1	-2,7160	0,0832	0,7656
1451235_at	Cend1	cell cycle exit and neuronal differentiation 1	-2,7160	0,0832	0,7335
1425123_at	Klhl36	kelch-like 36 (Drosophila)	-2,7162	0,0832	0,7047
1450801_at	Adam21	a disintegrin and metallopeptidase domain 21	-2,7162	0,0832	0,7616
1454618_at	C86187	expressed sequence C86187	-2,7163	0,0832	0,6100
1421302_a_at	Gna15	guanine nucleotide binding protein, alpha 15	-2,7165	0,0832	0,7631
1418993_s_at	F10	coagulation factor X	-2,7166	0,0832	0,7694
1456062_at	Nppa	natriuretic peptide precursor type A	-2,7167	0,0832	0,7956
1419741_at	Supt16h	suppressor of Ty 16 homolog (S. cerevisiae)	-2,7167	0,0832	0,7580
1432096_at	Snrpn	small nuclear ribonucleoprotein N	-2,7170	0,0832	0,5229
1422176_at	Fgf23	fibroblast growth factor 23	-2,7187	0,0832	0,8378
1416528_at	Sh3bgrl3	SH3 domain binding glutamic acid-rich protein-like 3	-2,7193	0,0832	0,8022
1420530_at	Dpf1	D4, zinc and double PHD fingers family 1	-2,7197	0,0832	0,8447
1448137_at	Aldh7a1	aldehyde dehydrogenase family 7, member A1	-2,7205	0,0831	0,7696
1423500_a_at	Sox5	SRY-box containing gene 5	-2,7211	0,0831	0,6503
1427076_at	Mpeg1	macrophage expressed gene 1	-2,7211	0,0831	0,7970
1419302_at	Heyl	hairy/enhancer-of-split related with YRPW motif-like	-2,7215	0,0831	0,8765
1419941_at	C030018P15Rik	RIKEN cDNA C030018P15 gene	-2,7219	0,0831	0,7779
1460272_at	Ubl4b	ubiquitin-like 4B	-2,7227	0,0831	0,8373
1449895_at	Acr	acrosin prepropeptide	-2,7229	0,0831	0,7614
1449420_at	Pde1b	phosphodiesterase 1B, Ca2+-calmodulin dependent	-2,7230	0,0831	0,7492
1450032_at	Slco2a1	solute carrier organic anion transporter family, member 2a1	-2,7230	0,0831	0,8069
1427329_a_at	Igh-6	immunoglobulin heavy chain 6 (heavy chain of IgM)	-2,7237	0,0831	0,7424

Probeset ID	*Symbol*	*Gene Name*	*d-value*	*q-value*	*R-fold*
1420428_at	Ager	advanced glycosylation end product-specific receptor	-2,7240	0,0831	0,6509
1448741_at	Slc3a1	solute carrier family 3, member 1	-2,7251	0,0831	0,7955
1420554_a_at	Rac3	RAS-related C3 botulinum substrate 3	-2,7254	0,0831	0,7534
1451688_s_at	Cant1	calcium activated nucleotidase 1	-2,7260	0,0831	0,6334
1450543_at	NULL	NULL	-2,7275	0,0831	0,7435
1424580_at	NULL	NULL	-2,7276	0,0831	0,9376
1418595_at	Plin4	perilipin 4	-2,7286	0,0831	0,6879
1454159_a_at	Igfbp2	insulin-like growth factor binding protein 2	-2,7294	0,0831	0,6889
1419414_at	Gng13	guanine nucleotide binding protein (G protein), gamma 13	-2,7299	0,0831	0,6510
1423813_at	Kif22	kinesin family member 22	-2,7300	0,0831	0,6208
1422010_at	Tlr7	toll-like receptor 7	-2,7303	0,0831	0,7838
1450578_at	Sry	sex determining region of Chr Y	-2,7315	0,0831	0,7876
1419099_x_at	Stom	stomatin	-2,7315	0,0831	0,6651
1427362_x_at	Hoxc6	homeobox C6	-2,7317	0,0831	0,5521
1419178_at	Cd3g	CD3 antigen, gamma polypeptide	-2,7317	0,0831	0,7633
1421802_at	Ear1	eosinophil-associated, ribonuclease A family, member 1	-2,7325	0,0831	0,9034
1451564_at	Parp14	poly (ADP-ribose) polymerase family, member 14	-2,7336	0,0831	0,7519
1420677_x_at	Lce1a1	late cornified envelope 1A1	-2,7338	0,0831	0,8559
1452485_at	Phospho1	phosphatase, orphan 1	-2,7338	0,0831	0,6447
1422179_at	Gjb4	gap junction protein, beta 4	-2,7340	0,0831	0,7270
1453107_s_at	NULL	NULL	-2,7344	0,0831	0,6617
1428394_at	NULL	NULL	-2,7349	0,0830	0,7602
1449284_at	1700008P20Rik	RIKEN cDNA 1700008P20 gene	-2,7352	0,0830	0,6312
1448000_at	Cdca3	cell division cycle associated 3	-2,7354	0,0830	0,8798
1438492_at	Socs7	suppressor of cytokine signaling 7	-2,7359	0,0830	0,6883
1428238_at	NULL	NULL	-2,7363	0,0830	0,9027
1450075_at	Polh	polymerase (DNA directed), eta (RAD 30 related)	-2,7381	0,0829	0,8142
1451686_x_at	Amelx	amelogenin X chromosome	-2,7396	0,0829	0,6829
1449330_at	Pdzd3	PDZ domain containing 3	-2,7397	0,0829	0,7669
1452308_a_at	Atp1a2	ATPase, Na+/K+ transporting, alpha 2 polypeptide	-2,7398	0,0829	0,6707
1449424_at	Plek2	pleckstrin 2	-2,7408	0,0829	0,8902
1460227_at	Timp1	tissue inhibitor of metalloproteinase 1	-2,7413	0,0829	0,6510
1427892_at	Myo1g	myosin IG	-2,7417	0,0829	0,7818
1427424_at	NULL	NULL	-2,7424	0,0829	0,7246
1418267_at	Mst1	macrophage stimulating 1 (hepatocyte growth factor-like)	-2,7445	0,0827	0,7097
1460372_at	Duoxa1	dual oxidase maturation factor 1	-2,7451	0,0827	0,7536
1451256_at	Gpr172b	G protein-coupled receptor 172B	-2,7452	0,0827	0,8432
1427083_a_at	Map4k5	mitogen-activated protein kinase kinase kinase kinase 5	-2,7455	0,0827	0,8306
1425081_at	Zfp286	zinc finger protein 286	-2,7459	0,0827	0,8598
1425375_at	Tdpoz1	TD and POZ domain containing 1	-2,7460	0,0827	0,9129
1427912_at	Cbr3	carbonyl reductase 3	-2,7461	0,0827	0,6929
1427751_a_at	Krt36	keratin 36	-2,7463	0,0827	0,7614
1418751_at	Sit1	suppression inducing transmembrane adaptor 1	-2,7467	0,0827	0,7981
1422514_at	Aebp1	AE binding protein 1	-2,7467	0,0827	0,7244
1425645_s_at	Cyp2b10	cytochrome P450, family 2, subfamily b, polypeptide 10	-2,7470	0,0827	0,8979
1431527_at	Cd164	CD164 antigen	-2,7472	0,0827	0,8034
1422699_at	Alox12	arachidonate 12-lipoxygenase	-2,7472	0,0827	0,7546
1447427_at	Gm2717	predicted gene 2717	-2,7472	0,0827	0,9404
1419970_at	Slc35a5	solute carrier family 35, member A5	-2,7486	0,0827	0,6788
1418457_at	Cxcl14	chemokine (C-X-C motif) ligand 14	-2,7488	0,0827	0,7722
1419877_x_at	2810449G22Rik	RIKEN cDNA 2810449G22 gene	-2,7490	0,0827	0,6192
1428026_at	Tshz2	teashirt zinc finger family member 2	-2,7493	0,0827	0,8540
1420393_at	Nos2	nitric oxide synthase 2, inducible	-2,7494	0,0827	0,9008
1427855_at	Igk-V38	immunoglobulin kappa chain variable 38(V38)	-2,7496	0,0827	0,7561
1423639_at	Hrh2	histamine receptor H2	-2,7499	0,0827	0,6898
1420269_at	2610201A13Rik	RIKEN cDNA 2610201A13 gene	-2,7508	0,0827	0,7696
1416028_a_at	Hn1	hematological and neurological expressed sequence 1	-2,7517	0,0826	0,8183
1422080_at	Il7	interleukin 7	-2,7521	0,0826	0,9184
1449156_at	Ly9	lymphocyte antigen 9	-2,7528	0,0826	0,8364
1427803_at	NULL	NULL	-2,7538	0,0826	0,7789
1419604_at	Zbp1	Z-DNA binding protein 1	-2,7548	0,0826	0,7818
1449472_at	Gpr12	G-protein coupled receptor 12	-2,7552	0,0826	0,7424
1436241_s_at	Hira	histone cell cycle regulation defective homolog A (S. cerevisiae)	-2,7553	0,0826	0,8505
1417414_at	sep-03	septin 3	-2,7554	0,0826	0,7508
1425689_at	Dpys	dihydropyrimidinase	-2,7556	0,0826	0,8483
1448386_a_at	Wap	whey acidic protein	-2,7559	0,0826	0,7381
1455965_at	Adamts4	a disintegrin-like and metallopeptidase (reprolysin type) with thrombospondin type 1 motif, 4	-2,7560	0,0826	0,6903
1450619_x_at	NULL	NULL	-2,7565	0,0826	0,7904
1422952_at	Ng23	Ng23 protein	-2,7569	0,0826	0,8077
1460414_at	Obsl1	obscurin-like 1	-2,7575	0,0825	0,8378
1455805_x_at	Ccdc22	coiled-coil domain containing 22	-2,7590	0,0825	0,8573
1416275_at	Slc26a6	solute carrier family 26, member 6	-2,7601	0,0825	0,8696
1423703_at	Ppan	peter pan homolog (Drosophila)	-2,7601	0,0825	0,8034
1451780_at	Blnk	B-cell linker	-2,7604	0,0825	0,8171
1453811_at	Hspa9	heat shock protein 9	-2,7610	0,0825	0,6717
1450366_at	Hrk	harakiri, BCL2 interacting protein (contains only BH3 domain)	-2,7621	0,0824	0,7621
1448560_at	Bid	BH3 interacting domain death agonist	-2,7624	0,0824	0,7843
1450128_at	Pla2g2a	phospholipase A2, group IIA (platelets, synovial fluid)	-2,7627	0,0824	0,8293

Probeset ID	Symbol	Gene Name	d-value	q-value	R-fold
1421192_a_at	Itsn1	intersectin 1 (SH3 domain protein 1A)	-2,7629	0,0824	0,7638
1428031_at	Mchr1	melanin-concentrating hormone receptor 1	-2,7636	0,0824	0,8093
1437145_s_at	2310002J15Rik	RIKEN cDNA 2310002J15 gene	-2,7637	0,0824	0,6964
1450182_at	Clcnka	chloride channel Ka	-2,7648	0,0824	0,6527
1419388_at	Tm4sf20	transmembrane 4 L six family member 20	-2,7657	0,0824	0,8062
1426563_at	Zfp553	zinc finger protein 553	-2,7658	0,0824	0,7564
1419886_at	DXErtd223e	DNA segment, Chr X, ERATO Doi 223, expressed	-2,7660	0,0824	0,9155
1425836_a_at	Limk1	LIM-domain containing, protein kinase	-2,7664	0,0824	0,6599
1423243_at	Mpp1	membrane protein, palmitoylated	-2,7667	0,0824	0,8428
1431331_at	Mgll	monoglyceride lipase	-2,7669	0,0824	0,7727
1425926_a_at	Otx2	orthodenticle homolog 2 (Drosophila)	-2,7677	0,0824	0,8246
1415902_at	Aldh7a1	aldehyde dehydrogenase family 7, member A1	-2,7681	0,0824	0,8587
1428363_at	Eif4g2	eukaryotic translation initiation factor 4, gamma 2	-2,7681	0,0824	0,8571
1434748_at	Ckap2	cytoskeleton associated protein 2	-2,7690	0,0824	0,8089
1451455_at	Thnsl2	threonine synthase-like 2 (bacterial)	-2,7702	0,0824	0,7821
1420362_a_at	Bik	BCL2-interacting killer	-2,7705	0,0824	0,7322
1419796_at	NULL	NULL	-2,7712	0,0824	0,7599
1422913_at	Prm3	protamine 3	-2,7718	0,0824	0,7257
1419705_at	Car5b	carbonic anhydrase 5b, mitochondrial	-2,7719	0,0824	0,8754
1425902_a_at	Nfkb2	nuclear factor of kappa light polypeptide gene enhancer in B-cells 2, p49/p100	-2,7719	0,0824	0,5787
1450469_at	Ddc8	differential display clone 8	-2,7721	0,0824	0,7758
1420022_s_at	Suz12	suppressor of zeste 12 homolog (Drosophila)	-2,7723	0,0824	0,8415
1415927_at	NULL	NULL	-2,7727	0,0824	0,8138
1417043_at	Lcat	lecithin cholesterol acyltransferase	-2,7735	0,0824	0,8271
1420672_at	Kcne1	potassium voltage-gated channel, Isk-related subfamily, member 1	-2,7739	0,0824	0,7765
1427542_at	Sobp	sine oculis-binding protein homolog (Drosophila)	-2,7743	0,0824	0,5732
1421705_at	Scn3a	sodium channel, voltage-gated, type III, alpha	-2,7750	0,0824	0,8007
1418725_at	Dnajc5b	DnaJ (Hsp40) homolog, subfamily C, member 5 beta	-2,7753	0,0824	0,6391
1452907_at	Galc	galactosylceramidase	-2,7757	0,0824	0,8426
1418204_s_at	Aif1	allograft inflammatory factor 1	-2,7764	0,0823	0,7151
1438494_at	NULL	NULL	-2,7766	0,0823	0,7127
1429752_x_at	Clip4	CAP-GLY domain containing linker protein family, member 4	-2,7766	0,0823	0,7228
1418765_at	Timd2	T-cell immunoglobulin and mucin domain containing 2	-2,7768	0,0823	0,7307
1427603_at	Atf7ip2	activating transcription factor 7 interacting protein 2	-2,7773	0,0823	0,8433
1454972_at	Atcay	ataxia, cerebellar, Cayman type homolog (human)	-2,7780	0,0823	0,6357
1417217_at	Magel2	melanoma antigen, family L, 2	-2,7780	0,0823	0,8184
1425100_a_at	Pde6g	phosphodiesterase 6G, cGMP-specific, rod, gamma	-2,7795	0,0823	0,6543
1417128_at	Plekho1	pleckstrin homology domain containing, family O member 1	-2,7808	0,0822	0,7752
1424327_at	3200002M19Rik	RIKEN cDNA 3200002M19 gene	-2,7810	0,0822	0,6508
1427201_at	Mustn1	musculoskeletal, embryonic nuclear protein 1	-2,7810	0,0822	0,6546
1421261_at	Lipg	lipase, endothelial	-2,7811	0,0822	0,7372
1449835_at	Pdcd1	programmed cell death 1	-2,7813	0,0822	0,6505
1451768_a_at	Slc20a2	solute carrier family 20, member 2	-2,7815	0,0822	0,6535
1430447_a_at	Lair1	leukocyte-associated Ig-like receptor 1	-2,7817	0,0822	0,9132
1422988_at	Sgsh	N-sulfoglucosamine sulfohydrolase (sulfamidase)	-2,7822	0,0822	0,6786
1452421_at	NULL	NULL	-2,7823	0,0822	0,8898
1452019_at	Cyyr1	cysteine and tyrosine-rich protein 1	-2,7823	0,0822	0,5967
1421291_at	Il18rap	interleukin 18 receptor accessory protein	-2,7824	0,0822	0,8467
1427759_a_at	Art5	ADP-ribosyltransferase 5	-2,7826	0,0822	0,7768
1448807_at	Hrh3	histamine receptor H3	-2,7839	0,0822	0,8243
1449959_x_at	Lce1h	late cornified envelope 1H	-2,7843	0,0822	0,7635
1421683_at	NULL	NULL	-2,7844	0,0822	0,8314
1450613_x_at	Ifnab	interferon alpha B	-2,7849	0,0822	0,8020
1427807_at	Gm10083	predicted gene 10083	-2,7858	0,0822	0,7882
1427805_at	NULL	NULL	-2,7858	0,0822	0,6312
1418756_at	Trh	thyrotropin releasing hormone	-2,7859	0,0822	0,6136
1426336_at	Cacng7	calcium channel, voltage-dependent, gamma subunit 7	-2,7862	0,0822	0,8953
1438836_at	Eftud2	elongation factor Tu GTP binding domain containing 2	-2,7866	0,0822	0,6873
1451549_at	Cngb1	cyclic nucleotide gated channel beta 1	-2,7869	0,0822	0,5533
1436500_at	NULL	NULL	-2,7875	0,0822	0,5468
1451713_a_at	Fcer2a	Fc receptor, IgE, low affinity II, alpha polypeptide	-2,7883	0,0822	0,8145
1428361_x_at	NULL	NULL	-2,7886	0,0822	0,5094
1421928_at	Epha4	Eph receptor A4	-2,7893	0,0822	0,8422
1418989_at	Ctse	cathepsin E	-2,7897	0,0821	0,7267
1427018_at	Tsnaxip1	translin-associated factor X (Tsnax) interacting protein 1	-2,7907	0,0821	0,7322
1452389_at	Cd27	CD27 antigen	-2,7909	0,0821	0,8186
1418642_at	Lcp2	lymphocyte cytosolic protein 2	-2,7910	0,0821	0,8483
1455093_a_at	Ahsg	alpha-2-HS-glycoprotein	-2,7912	0,0821	0,7319
1449969_at	Tmod4	tropomodulin 4	-2,7914	0,0821	0,8011
1417092_at	Pth1r	parathyroid hormone 1 receptor	-2,7921	0,0821	0,7983
1450402_at	Med1	mediator complex subunit 1	-2,7925	0,0821	0,7612
1421397_a_at	Lrdd	leucine-rich and death domain containing	-2,7934	0,0821	0,8098
1422276_at	P2ry4	pyrimidinergic receptor P2Y, G-protein coupled, 4	-2,7937	0,0821	0,7206
1450829_at	Tnfaip3	tumor necrosis factor, alpha-induced protein 3	-2,7940	0,0821	0,7101
1451822_a_at	Scrn2	secernin 2	-2,7940	0,0821	0,6522
1453182_a_at	Smpd4	sphingomyelin phosphodiesterase 4	-2,7943	0,0821	0,6929
1428096_at	Ipo11	importin 11	-2,7943	0,0821	0,7661
1421896_at	Elk1	ELK1, member of ETS oncogene family	-2,7945	0,0821	0,7923

Probeset ID	Symbol	Gene Name	d-value	q-value	R-fold
1420805_at	Myl10	myosin, light chain 10, regulatory	-2,7947	0,0821	0,7170
1423011_at	Mecom	MDS1 and EVI1 complex locus	-2,7954	0,0821	0,9020
1448871_at	Mapk13	mitogen-activated protein kinase 13	-2,7958	0,0821	0,7237
1433407_at	Mcm10	minichromosome maintenance deficient 10 (S. cerevisiae)	-2,7962	0,0821	0,8231
1420990_at	Chd1	chromodomain helicase DNA binding protein 1	-2,7963	0,0821	0,7373
1453659_at	Cenpo	centromere protein O	-2,7967	0,0821	0,8615
1453614_a_at	Nfe2l3	nuclear factor, erythroid derived 2, like 3	-2,7969	0,0821	0,8371
1452425_at	Tnfrsf14	tumor necrosis factor receptor superfamily, member 14 (herpesvirus entry mediator)	-2,7975	0,0821	0,8328
1438949_at	Ric8	resistance to inhibitors of cholinesterase 8 homolog (C. elegans)	-2,7987	0,0821	0,7248
1453991_at	Cenpo	centromere protein O	-2,7987	0,0821	0,7885
1450562_at	Ly6f	lymphocyte antigen 6 complex, locus F	-2,7989	0,0821	0,8386
1425883_at	Smg6	Smg-6 homolog, nonsense mediated mRNA decay factor (C. elegans)	-2,8000	0,0821	0,7557
1427843_at	NULL	NULL	-2,8007	0,0821	0,7570
1450544_at	Ccdc48	coiled-coil domain containing 48	-2,8008	0,0821	0,7222
1451492_at	Sla2	Src-like-adaptor 2	-2,8015	0,0821	0,7621
1421299_a_at	Lef1	lymphoid enhancer binding factor 1	-2,8023	0,0821	0,8235
1435476_a_at	Fcgr2b	Fc receptor, IgG, low affinity IIb	-2,8026	0,0821	0,7401
1422082_a_at	Nfya	nuclear transcription factor-Y alpha	-2,8038	0,0821	0,9239
1425696_at	Nxnl1	nucleoredoxin-like 1	-2,8043	0,0821	0,7209
1424338_at	Slc6a13	solute carrier family 6 (neurotransmitter transporter, GABA), member 13	-2,8053	0,0820	0,6321
1420732_at	Rwdd3	RWD domain containing 3	-2,8054	0,0820	0,8355
1426588_at	Tspan10	tetraspanin 10	-2,8061	0,0820	0,7730
1427825_at	NULL	NULL	-2,8071	0,0820	0,8917
1422482_at	Ruvbl2	RuvB-like protein 2	-2,8075	0,0820	0,8451
1452040_a_at	Cdca3	cell division cycle associated 3	-2,8078	0,0820	0,8091
1418554_at	Gpr182	G protein-coupled receptor 182	-2,8086	0,0820	0,8758
1417785_at	Pla1a	phospholipase A1 member A	-2,8102	0,0819	0,7084
1430772_at	NULL	NULL	-2,8111	0,0819	0,7905
1422231_a_at	Tnfrsf25	tumor necrosis factor receptor superfamily, member 25	-2,8111	0,0819	0,7629
1426341_at	Slc1a3	solute carrier family 1 (glial high affinity glutamate transporter), member 3	-2,8113	0,0819	0,5913
1418148_at	Abhd1	abhydrolase domain containing 1	-2,8118	0,0819	0,6191
1426122_a_at	Coro6	coronin 6	-2,8124	0,0819	0,7713
1421444_at	Pgr	progesterone receptor	-2,8126	0,0819	0,8107
1418056_at	Iqcf3	IQ motif containing F3	-2,8137	0,0818	0,6527
1419097_a_at	Stom	stomatin	-2,8153	0,0817	0,8113
1425871_a_at	NULL	NULL	-2,8167	0,0817	0,7673
1448553_at	Myh7	myosin, heavy polypeptide 7, cardiac muscle, beta	-2,8170	0,0817	0,6404
1450364_a_at	Havcr1	hepatitis A virus cellular receptor 1	-2,8171	0,0817	0,7526
1450833_at	Chrm1	cholinergic receptor, muscarinic 1, CNS	-2,8171	0,0817	0,6460
1422872_at	Bmpr1b	bone morphogenetic protein receptor, type 1B	-2,8191	0,0816	0,6571
1436442_at	Ptpn14	protein tyrosine phosphatase, non-receptor type 14	-2,8194	0,0816	0,7479
1459927_at	4833445I07Rik	RIKEN cDNA 4833445I07 gene	-2,8194	0,0816	0,8164
1449656_at	D930048N14Rik	RIKEN cDNA D930048N14 gene	-2,8201	0,0816	0,7775
1452976_a_at	Slc9a3r2	solute carrier family 9 (sodium/hydrogen exchanger), member 3 regulator 2	-2,8206	0,0816	0,6011
1427562_a_at	Prkca	protein kinase C, alpha	-2,8213	0,0816	0,6840
1434959_at	Dhh	desert hedgehog	-2,8214	0,0816	0,7720
1419194_s_at	Gmfg	glia maturation factor, gamma	-2,8219	0,0816	0,6708
1453527_a_at	Neurl1a	neuralized homolog 1A (Drosophila)	-2,8227	0,0816	0,7829
1421089_a_at	Pinx1	PIN2/TERF1 interacting, telomerase inhibitor 1	-2,8233	0,0816	0,5897
1418649_at	Egln3	EGL nine homolog 3 (C. elegans)	-2,8234	0,0816	0,9107
1422055_at	Mid1	midline 1	-2,8237	0,0816	0,6709
1422125_at	Htr2b	5-hydroxytryptamine (serotonin) receptor 2B	-2,8238	0,0816	0,7495
1427890_a_at	Lrrc56	leucine rich repeat containing 56	-2,8241	0,0816	0,7905
1460365_a_at	Dnm1	dynamin 1	-2,8249	0,0816	0,8604
1425833_a_at	Hpca	hippocalcin	-2,8251	0,0816	0,5535
1434227_at	Krtdap	keratinocyte differentiation associated protein	-2,8276	0,0816	0,8304
1460212_at	Gnat1	guanine nucleotide binding protein, alpha transducing 1	-2,8287	0,0816	0,8324
1422101_at	Tnfrsf23	tumor necrosis factor receptor superfamily, member 23	-2,8304	0,0815	0,8297
1428575_at	Fcho1	FCH domain only 1	-2,8317	0,0815	0,7733
1427626_at	Muc5b	mucin 5, subtype B, tracheobronchial	-2,8327	0,0815	0,7555
1417148_at	Pdgfrb	platelet derived growth factor receptor, beta polypeptide	-2,8334	0,0815	0,6781
1422919_at	Hrasls	HRAS-like suppressor	-2,8335	0,0815	0,7925
1446829_at	D12Ertd216e	DNA segment, Chr 12, ERATO Doi 216, expressed	-2,8335	0,0815	0,7834
1451565_s_at	Uroc1	urocanase domain containing 1	-2,8340	0,0815	0,7763
1420604_at	Hesx1	homeobox gene expressed in ES cells	-2,8344	0,0815	0,7705
1420192_at	Tmem191c	transmembrane protein 191C	-2,8347	0,0815	0,8212
1417808_at	2310050C09Rik	RIKEN cDNA 2310050C09 gene	-2,8354	0,0815	0,8178
1431214_at	LOC67527	murine leukemia retrovirus	-2,8355	0,0815	0,4322
1455867_at	Sox4	SRY-box containing gene 4	-2,8357	0,0815	0,6354
1417756_a_at	Lsp1	lymphocyte specific 1	-2,8357	0,0815	0,7580
1448662_at	Fzd6	frizzled homolog 6 (Drosophila)	-2,8365	0,0815	0,7336
1419747_at	Asgr2	asialoglycoprotein receptor 2	-2,8365	0,0815	0,7629
1419722_at	Klk8	kallikrein related-peptidase 8	-2,8382	0,0814	0,6631
1433573_x_at	Prss2	protease, serine, 2	-2,8382	0,0814	0,9194
1448470_at	Fbp1	fructose bisphosphatase 1	-2,8384	0,0814	0,7383
1427667_s_at	Tcrb-J	T-cell receptor beta, joining region	-2,8406	0,0814	0,8740

Probeset ID	*Symbol*	*Gene Name*	*d-value*	*q-value*	*R-fold*
1421898_a_at	Mr1	major histocompatibility complex, class I-related	-2,8413	0,0814	0,7069
1418836_at	Qprt	quinolinate phosphoribosyltransferase	-2,8414	0,0814	0,7112
1422288_at	Htr1b	5-hydroxytryptamine (serotonin) receptor 1B	-2,8422	0,0814	0,7798
1448945_at	Pllp	plasma membrane proteolipid	-2,8425	0,0814	0,7069
1450304_at	Klrc2	killer cell lectin-like receptor subfamily C, member 2	-2,8426	0,0814	0,8401
1420525_a_at	Otc	ornithine transcarbamylase	-2,8427	0,0814	0,8221
1420047_at	Maf1	MAF1 homolog (S. cerevisiae)	-2,8431	0,0814	0,8196
1421760_at	Ptcra	pre T-cell antigen receptor alpha	-2,8435	0,0814	0,8313
1419775_at	NULL	NULL	-2,8443	0,0814	0,8169
1418200_at	Zbtb48	zinc finger and BTB domain containing 48	-2,8446	0,0814	0,9085
1424722_at	1300017J02Rik	RIKEN cDNA 1300017J02 gene	-2,8447	0,0814	0,7715
1421978_at	Gad2	glutamic acid decarboxylase 2	-2,8448	0,0814	0,7420
1451503_at	Nol3	nucleolar protein 3 (apoptosis repressor with CARD domain)	-2,8457	0,0814	0,8115
1456870_at	Rasal3	RAS protein activator like 3	-2,8462	0,0814	0,6557
1427374_at	Muc3	mucin 3, intestinal	-2,8467	0,0814	0,6429
1424367_a_at	Homer2	homer homolog 2 (Drosophila)	-2,8473	0,0814	0,7275
1424393_s_at	Adhfe1	alcohol dehydrogenase, iron containing, 1	-2,8473	0,0814	0,7929
1428030_at	Adam34	a disintegrin and metallopeptidase domain 34	-2,8475	0,0814	0,8303
1423590_at	Napsa	napsin A aspartic peptidase	-2,8480	0,0814	0,7292
1432075_a_at	Tekt1	tektin 1	-2,8487	0,0814	0,6935
1424162_at	Trim29	tripartite motif-containing 29	-2,8488	0,0814	0,7836
1420353_at	Lta	lymphotoxin A	-2,8488	0,0814	0,7576
1418044_at	Hmgxb3	HMG box domain containing 3	-2,8489	0,0814	0,6647
1426213_at	Imp4	IMP4, U3 small nucleolar ribonucleoprotein, homolog (yeast)	-2,8493	0,0814	0,8328
1425695_at	Tbx5	T-box 5	-2,8500	0,0814	0,6891
1426115_a_at	Kcnj9	potassium inwardly-rectifying channel, subfamily J, member 9	-2,8501	0,0814	0,7602
1421929_at	Epha4	Eph receptor A4	-2,8504	0,0814	0,7688
1449761_at	AA589418	expressed sequence AA589418	-2,8508	0,0814	0,8591
1422353_at	Pou4f3	POU domain, class 4, transcription factor 3	-2,8511	0,0814	0,6898
1438608_at	Tnni2	troponin I, skeletal, fast 2	-2,8516	0,0814	0,7108
1445172_at	Lgtn	ligatin	-2,8524	0,0814	0,8106
1424527_at	Ppp2r2d	protein phosphatase 2, regulatory subunit B, delta isoform	-2,8528	0,0814	0,8374
1419689_at	Gpc6	glypican 6	-2,8542	0,0814	0,6955
1427799_x_at	NULL	NULL	-2,8555	0,0814	0,7710
1421446_at	Prkcc	protein kinase C, gamma	-2,8560	0,0814	0,7772
1451116_at	Aacs	acetoacetyl-CoA synthetase	-2,8564	0,0814	0,8160
1422012_at	Crhr2	corticotropin releasing hormone receptor 2	-2,8566	0,0814	0,7168
1449611_at	Cd82	CD82 antigen	-2,8574	0,0814	0,7528
1418655_at	B4galnt1	beta-1,4-N-acetyl-galactosaminyl transferase 1	-2,8578	0,0814	0,5732
1449285_at	Cst9	cystatin 9	-2,8579	0,0814	0,8900
1422968_at	Ip6k1	inositol hexaphosphate kinase 1	-2,8584	0,0814	0,6611
1449905_at	Clec4f	C-type lectin domain family 4, member f	-2,8596	0,0814	0,5943
1450820_a_at	Ntng2	netrin G2	-2,8599	0,0814	0,6883
1426153_a_at	Dsg2	desmoglein 2	-2,8612	0,0814	0,9080
1420807_a_at	Dlk2	delta-like 2 homolog (Drosophila)	-2,8625	0,0813	0,5764
1425712_at	BC025446	cDNA sequence BC025446	-2,8625	0,0813	0,7782
1451369_at	Commd5	COMM domain containing 5	-2,8635	0,0813	0,6282
1425729_at	Best2	bestrophin 2	-2,8637	0,0813	0,6802
1420446_at	Odf3	outer dense fiber of sperm tails 3	-2,8639	0,0813	0,6352
1416930_at	Ly6d	lymphocyte antigen 6 complex, locus D	-2,8643	0,0813	0,8357
1449392_at	Hsd17b1	hydroxysteroid (17-beta) dehydrogenase 1	-2,8648	0,0813	0,8387
1431862_x_at	Odf2l	outer dense fiber of sperm tails 2-like	-2,8649	0,0813	0,8023
1420183_at	Lor	loricrin	-2,8650	0,0813	0,8950
1419851_at	Slc4a8	solute carrier family 4 (anion exchanger), member 8	-2,8652	0,0813	0,8143
1422328_at	Gja10	gap junction protein, alpha 10	-2,8690	0,0813	0,8006
1424936_a_at	Dnahc8	dynein, axonemal, heavy chain 8	-2,8696	0,0812	0,6880
1456456_x_at	Mela	melanoma antigen	-2,8704	0,0811	0,7641
1449382_at	Slc6a12	solute carrier family 6 (neurotransmitter transporter, betaine/GABA), member 12	-2,8709	0,0811	0,8316
1451834_at	Cacnb1	calcium channel, voltage-dependent, beta 1 subunit	-2,8712	0,0811	0,5594
1449421_a_at	Kcne2	potassium voltage-gated channel, Isk-related subfamily, gene 2	-2,8729	0,0811	0,8700
1431936_a_at	Neu2	neuraminidase 2	-2,8731	0,0811	0,8166
1422258_at	Chrm3	cholinergic receptor, muscarinic 3, cardiac	-2,8732	0,0811	0,8023
1450126_at	Gata5	GATA binding protein 5	-2,8739	0,0811	0,8009
1449865_at	Sema3a	sema domain, immunoglobulin domain (Ig), short basic domain, secreted, (semaphorin) 3A	-2,8741	0,0811	0,8163
1418233_a_at	Trappc5	trafficking protein particle complex 5	-2,8755	0,0810	0,7716
1460596_at	Agtrap	angiotensin II, type I receptor-associated protein	-2,8756	0,0810	0,6717
1425044_at	Kcnj6	potassium inwardly-rectifying channel, subfamily J, member 6	-2,8769	0,0810	0,7321
1421045_at	Mrc2	mannose receptor, C type 2	-2,8775	0,0810	0,7001
1450225_at	Insr	insulin receptor	-2,8786	0,0810	0,8036
1450719_at	Mep1a	meprin 1 alpha	-2,8788	0,0810	0,7166
1426016_a_at	Tro	trophinin	-2,8804	0,0809	0,8063
1436454_x_at	Fen1	flap structure specific endonuclease 1	-2,8814	0,0809	0,6145
1447287_at	C77137	expressed sequence C77137	-2,8822	0,0809	0,7492
1452386_at	Sall3	sal-like 3 (Drosophila)	-2,8834	0,0809	0,5721
1417017_at	Cyp17a1	cytochrome P450, family 17, subfamily a, polypeptide 1	-2,8838	0,0809	0,6982
1417596_at	B9d1	B9 protein domain 1	-2,8840	0,0809	0,7976
1450390_x_at	Rps18	ribosomal protein S18	-2,8841	0,0809	0,8998

Probeset ID	Symbol	Gene Name	d-value	q-value	R-fold
1427716_at	Prss44	protease, serine, 44	-2,8844	0,0809	0,6995
1427571_at	Shh	sonic hedgehog	-2,8847	0,0809	0,7851
1420793_at	Mup4	major urinary protein 4	-2,8857	0,0808	0,8381
1420260_at	Phf7	PHD finger protein 7	-2,8865	0,0808	0,7698
1449881_a_at	Casr	calcium-sensing receptor	-2,8868	0,0808	0,8231
1438545_at	NULL	NULL	-2,8869	0,0808	0,6549
1427725_a_at	Pou2f2	POU domain, class 2, transcription factor 2	-2,8873	0,0808	0,7165
1460290_at	Lpin2	lipin 2	-2,8876	0,0808	0,6889
1454940_at	NULL	NULL	-2,8878	0,0808	0,8076
1450590_at	Olfr159	olfactory receptor 159	-2,8879	0,0808	0,8007
1451798_at	Il1rn	interleukin 1 receptor antagonist	-2,8879	0,0808	0,8753
1417979_at	Tnmd	tenomodulin	-2,8881	0,0808	0,8816
1425688_a_at	Dpys	dihydropyrimidinase	-2,8887	0,0808	0,8221
1427989_at	Amhr2	anti-Mullerian hormone type 2 receptor	-2,8894	0,0808	0,6750
1453258_at	NULL	NULL	-2,8905	0,0808	0,7004
1451655_at	Slfn8	schlafen 8	-2,8916	0,0808	0,6646
1418641_at	Lcp2	lymphocyte cytosolic protein 2	-2,8920	0,0808	0,6648
1416707_a_at	Pmf1	polyamine-modulated factor 1	-2,8924	0,0808	0,6727
1451662_x_at	Akap4	A kinase (PRKA) anchor protein 4	-2,8928	0,0808	0,6804
1451093_at	Polr2e	polymerase (RNA) II (DNA directed) polypeptide E	-2,8937	0,0808	0,6769
1438731_at	Sgsh	N-sulfoglucosamine sulfohydrolase (sulfamidase)	-2,8941	0,0808	0,8502
1422750_a_at	Zmynd10	zinc finger, MYND domain containing 10	-2,8956	0,0808	0,6934
1449307_at	Dbndd1	dysbindin (dystrobrevin binding protein 1) domain containing 1	-2,8957	0,0808	0,7507
1425178_s_at	Shmt1	serine hydroxymethyltransferase 1 (soluble)	-2,8967	0,0808	0,7084
1417909_at	Serpinc1	serine (or cysteine) peptidase inhibitor, clade C (antithrombin), member 1	-2,8976	0,0807	0,8714
1425743_at	Trim7	tripartite motif-containing 7	-2,8987	0,0807	0,7462
1417523_at	Plek	pleckstrin	-2,8989	0,0807	0,8602
1460415_a_at	Cd40	CD40 antigen	-2,9000	0,0807	0,7076
1423866_at	Serpina3k	serine (or cysteine) peptidase inhibitor, clade A, member 3K	-2,9000	0,0807	0,9303
1419675_at	Ngf	nerve growth factor	-2,9008	0,0807	0,6437
1418216_at	Ggt5	gamma-glutamyltransferase 5	-2,9010	0,0807	0,8166
1422269_at	Insm2	insulinoma-associated 2	-2,9020	0,0807	0,7623
1421757_at	Htr6	5-hydroxytryptamine (serotonin) receptor 6	-2,9024	0,0807	0,8301
1423593_a_at	Csf1r	colony stimulating factor 1 receptor	-2,9034	0,0807	0,6993
1449528_at	Figf	c-fos induced growth factor	-2,9035	0,0807	0,8309
1460355_at	Bud13	BUD13 homolog (yeast)	-2,9037	0,0807	0,7391
1437890_at	Btbd17	BTB (POZ) domain containing 17	-2,9038	0,0807	0,7602
1423029_at	Hes2	hairy and enhancer of split 2 (Drosophila)	-2,9040	0,0807	0,6326
1426526_s_at	Ovgp1	oviductal glycoprotein 1	-2,9051	0,0807	0,8267
1415991_a_at	Klhdc3	kelch domain containing 3	-2,9051	0,0807	0,7980
1448070_at	Xpo1	exportin 1, CRM1 homolog (yeast)	-2,9052	0,0807	0,8032
1418912_at	Plxdc2	plexin domain containing 2	-2,9058	0,0807	0,8615
1420804_s_at	Clec4d	C-type lectin domain family 4, member d	-2,9070	0,0807	0,7766
1427555_at	Mll2	myeloid/lymphoid or mixed-lineage leukemia 2	-2,9080	0,0806	0,6982
1427808_at	Nfya	nuclear transcription factor-Y alpha	-2,9083	0,0806	0,8213
1422856_at	Slc12a3	solute carrier family 12, member 3	-2,9086	0,0806	0,7559
1448506_at	Serpina6	serine (or cysteine) peptidase inhibitor, clade A, member 6	-2,9093	0,0806	0,7765
1451910_a_at	Cd6	CD6 antigen	-2,9097	0,0806	0,8036
1420451_at	Accn5	amiloride-sensitive cation channel 5, intestinal	-2,9100	0,0806	0,8794
1416169_at	Tpbpb	trophoblast specific protein beta	-2,9102	0,0806	0,8794
1420064_s_at	Tktl1	transketolase-like 1	-2,9102	0,0806	0,8808
1450439_at	Hcfc1	host cell factor C1	-2,9102	0,0806	0,7582
1420855_at	Eln	elastin	-2,9106	0,0806	0,7874
1448998_at	Lpo	lactoperoxidase	-2,9119	0,0806	0,8542
1423271_at	Gjb2	gap junction protein, beta 2	-2,9127	0,0806	0,6506
1449191_at	Wfdc12	WAP four-disulfide core domain 12	-2,9144	0,0805	0,6167
1421153_at	Loxl4	lysyl oxidase-like 4	-2,9147	0,0805	0,8373
1419119_at	Hcst	hematopoietic cell signal transducer	-2,9152	0,0805	0,6678
1420030_at	Slu7	SLU7 splicing factor homolog (S. cerevisiae)	-2,9155	0,0805	0,8838
1450670_at	Dbh	dopamine beta hydroxylase	-2,9177	0,0804	0,6743
1428089_at	Slitrk1	SLIT and NTRK-like family, member 1	-2,9184	0,0804	0,8559
1451868_at	Kcnj6	potassium inwardly-rectifying channel, subfamily J, member 6	-2,9187	0,0804	0,8629
1450571_a_at	NULL	NULL	-2,9190	0,0804	0,7917
1431597_a_at	Nrip3	nuclear receptor interacting protein 3	-2,9195	0,0804	0,8750
1421110_at	Mtdh	metadherin	-2,9196	0,0804	0,8636
1456658_at	Acta2	actin, alpha 2, smooth muscle, aorta	-2,9199	0,0804	0,8803
1422829_at	Drd4	dopamine receptor D4	-2,9201	0,0804	0,7785
1421862_a_at	Vamp1	vesicle-associated membrane protein 1	-2,9212	0,0804	0,7804
1440963_at	Cbfa2t3	core-binding factor, runt domain, alpha subunit 2, translocated to, 3 (human)	-2,9214	0,0804	0,8134
1451055_at	Slc45a2	solute carrier family 45, member 2	-2,9214	0,0804	0,6620
1450178_at	Brdt	bromodomain, testis-specific	-2,9221	0,0804	0,7469
1418666_at	Ptx3	pentraxin related gene	-2,9241	0,0804	0,8534
1427015_at	Nckap5l	NCK-associated protein 5-like	-2,9255	0,0803	0,8331
1460275_at	Gpr3	G-protein coupled receptor 3	-2,9260	0,0803	0,5942
1421826_at	Dll4	delta-like 4 (Drosophila)	-2,9262	0,0803	0,8463
1419799_at	Rpl27a	ribosomal protein L27A	-2,9266	0,0803	0,7762
1449871_at	Tbx18	T-box18	-2,9268	0,0803	0,8420

Probeset ID	Symbol	Gene Name	d-value	q-value	R-fold
1427391_a_at	Col12a1	collagen, type XII, alpha 1	-2,9272	0,0803	0,7314
1419776_at	NULL	NULL	-2,9282	0,0803	0,7867
1423285_at	Coch	coagulation factor C homolog (Limulus polyphemus)	-2,9282	0,0803	0,8703
1451862_a_at	Prf1	perforin 1 (pore forming protein)	-2,9283	0,0803	0,6745
1428456_at	Bola1	bolA-like 1 (E. coli)	-2,9293	0,0803	0,7698
1429884_at	Srgap2	SLIT-ROBO Rho GTPase activating protein 2	-2,9319	0,0802	0,6858
1421548_at	Pcdhb2	protocadherin beta 2	-2,9325	0,0802	0,6744
1456030_at	Klf13	Kruppel-like factor 13	-2,9326	0,0802	0,7526
1427613_at	AY074887	cDNA sequence AY074887	-2,9338	0,0802	0,8386
1416363_at	NULL	NULL	-2,9346	0,0802	0,7049
1416888_at	Fadd	Fas (TNFRSF6)-associated via death domain	-2,9347	0,0802	0,7693
1421902_at	Med29	mediator complex subunit 29	-2,9359	0,0802	0,7790
1431062_a_at	Exoc4	exocyst complex component 4	-2,9360	0,0802	0,6882
1448561_at	Ncf2	neutrophil cytosolic factor 2	-2,9362	0,0802	0,8278
1423013_at	Foxf1a	forkhead box F1a	-2,9368	0,0802	0,7923
1456546_at	1700097N02Rik	RIKEN cDNA 1700097N02 gene	-2,9373	0,0802	0,8122
1419309_at	Pdpn	podoplanin	-2,9373	0,0802	0,5895
1460375_at	Trmt112	tRNA methyltransferase 11-2 homolog (S. cerevisiae)	-2,9375	0,0802	0,8038
1449467_at	NULL	NULL	-2,9375	0,0802	0,7687
1420799_at	Ntsr1	neurotensin receptor 1	-2,9381	0,0802	0,7327
1450092_at	Ighmbp2	immunoglobulin mu binding protein 2	-2,9389	0,0802	0,7145
1451488_at	Fitm1	fat storage-inducing transmembrane protein 1	-2,9394	0,0802	0,7528
1454265_a_at	NULL	NULL	-2,9407	0,0802	0,6071
1421427_at	Col25a1	collagen, type XXV, alpha 1	-2,9415	0,0801	0,6778
1424195_a_at	Inpp5d	inositol polyphosphate-5-phosphatase D	-2,9417	0,0801	0,5647
1435853_at	NULL	NULL	-2,9418	0,0801	0,8040
1449163_at	Sigirr	single immunoglobulin and toll-interleukin 1 receptor (TIR) domain	-2,9420	0,0801	0,6842
1431549_at	NULL	NULL	-2,9431	0,0801	0,6523
1421290_at	Hspb7	heat shock protein family, member 7 (cardiovascular)	-2,9434	0,0801	0,8103
1427367_at	Efcab4a	EF-hand calcium binding domain 4A	-2,9439	0,0801	0,7712
1435830_a_at	5430435G22Rik	RIKEN cDNA 5430435G22 gene	-2,9450	0,0801	0,7558
1428054_at	Slc8a2	solute carrier family 8 (sodium/calcium exchanger), member 2	-2,9454	0,0801	0,6856
1424546_at	BC003965	cDNA sequence BC003965	-2,9456	0,0801	0,9412
1449260_at	Rab3d	RAB3D, member RAS oncogene family	-2,9458	0,0801	0,8182
1453026_at	Fam166a	family with sequence similarity 166, member A	-2,9468	0,0801	0,7309
1420740_at	Il25	interleukin 25	-2,9474	0,0801	0,8097
1431419_at	Prelid1	PRELI domain containing 1	-2,9481	0,0801	0,8447
1426193_at	Otos	otospiralin	-2,9490	0,0801	0,7743
1431878_at	Grhl2	grainyhead-like 2 (Drosophila)	-2,9497	0,0801	0,7360
1426116_at	Asb18	ankyrin repeat and SOCS box-containing 18	-2,9510	0,0800	0,7884
1459990_at	Ddr1	discoidin domain receptor family, member 1	-2,9520	0,0800	0,6433
1427993_at	Rufy2	RUN and FYVE domain-containing 2	-2,9521	0,0800	0,9185
1418864_at	Gata4	GATA binding protein 4	-2,9526	0,0800	0,7221
1453553_at	NULL	NULL	-2,9526	0,0800	0,6949
1420169_at	AA517841	expressed sequence AA517841	-2,9534	0,0800	0,7404
1450014_at	Cldn1	claudin 1	-2,9538	0,0800	0,7875
1427957_at	9530008L14Rik	RIKEN cDNA 9530008L14 gene	-2,9547	0,0800	0,7684
1456411_at	NULL	NULL	-2,9555	0,0800	0,7353
1420658_at	Ucp3	uncoupling protein 3 (mitochondrial, proton carrier)	-2,9555	0,0800	0,6632
1434147_at	Rell2	RELT-like 2	-2,9564	0,0800	0,6921
1417841_at	Pxmp2	peroxisomal membrane protein 2	-2,9572	0,0800	0,7915
1420148_at	Slc6a6	solute carrier family 6 (neurotransmitter transporter, taurine), member 6	-2,9582	0,0800	0,6482
1422290_at	Htr1d	5-hydroxytryptamine (serotonin) receptor 1D	-2,9584	0,0800	0,7530
1427724_at	Top2a	topoisomerase (DNA) II alpha	-2,9587	0,0800	0,6155
1451685_at	Mllt6	myeloid/lymphoid or mixed-lineage leukemia (trithorax homolog, Drosophila); translocated to, 6	-2,9590	0,0800	0,9127
1451297_at	Gulo	gulonolactone (L-) oxidase	-2,9592	0,0800	0,8030
1419086_at	Fgfbp1	fibroblast growth factor binding protein 1	-2,9592	0,0800	0,8908
1452314_at	Kif11	kinesin family member 11	-2,9596	0,0800	0,8549
1450239_at	Glra3	glycine receptor, alpha 3 subunit	-2,9596	0,0800	0,8319
1417760_at	Nr0b1	nuclear receptor subfamily 0, group B, member 1	-2,9619	0,0799	0,8462
1422831_at	Fbn2	fibrillin 2	-2,9621	0,0799	0,7237
1417735_at	1810030J14Rik	RIKEN cDNA 1810030J14 gene	-2,9622	0,0799	0,7738
1450477_at	Htr2c	5-hydroxytryptamine (serotonin) receptor 2C	-2,9625	0,0799	0,9157
1450552_at	Atp6v1e2	ATPase, H+ transporting, lysosomal V1 subunit E2	-2,9628	0,0799	0,8027
1425763_x_at	NULL	NULL	-2,9630	0,0799	0,4765
1431624_a_at	Rnf219	ring finger protein 219	-2,9633	0,0799	0,8904
1425846_a_at	Caln1	calneuron 1	-2,9644	0,0799	0,6471
1451384_at	Jmjd5	jumonji domain containing 5	-2,9644	0,0799	0,8468
1423342_at	Barx1	BarH-like homeobox 1	-2,9645	0,0799	0,6226
1428005_at	NULL	NULL	-2,9650	0,0799	0,9136
1417561_at	Apoc1	apolipoprotein C-I	-2,9651	0,0799	0,7599
1419567_at	Fank1	fibronectin type 3 and ankyrin repeat domains 1	-2,9655	0,0799	0,8245
1437420_at	Baz1b	bromodomain adjacent to zinc finger domain, 1B	-2,9660	0,0799	0,7223
1421010_at	Mobp	myelin-associated oligodendrocytic basic protein	-2,9679	0,0798	0,9559
1419012_at	Zfpm2	zinc finger protein, multitype 2	-2,9694	0,0798	0,7638
1421165_at	Mycbp	c-myc binding protein	-2,9701	0,0798	0,8748
1422084_at	Bmx	BMX non-receptor tyrosine kinase	-2,9708	0,0798	0,9184
1450327_at	P2rx6	purinergic receptor P2X, ligand-gated ion channel, 6	-2,9712	0,0797	0,6593

Probeset ID	Symbol	Gene Name	d-value	q-value	R-fold
1436952_at	Klf9	Kruppel-like factor 9	-2,9716	0,0797	0,8917
1449081_at	Ces3	carboxylesterase 3	-2,9717	0,0797	0,5958
1449570_at	Klrb1c	killer cell lectin-like receptor subfamily B member 1C	-2,9718	0,0797	0,7735
1451386_at	Blvrb	biliverdin reductase B (flavin reductase (NADPH))	-2,9718	0,0797	0,6689
1432845_at	NULL	NULL	-2,9720	0,0797	0,7880
1418950_at	Drd2	dopamine receptor D2	-2,9722	0,0797	0,7221
1425842_at	Edil3	EGF-like repeats and discoidin I-like domains 3	-2,9732	0,0797	0,8648
1417421_at	S100a1	S100 calcium binding protein A1	-2,9736	0,0797	0,7848
1437054_x_at	Prm1	protamine 1	-2,9739	0,0797	0,8843
1449540_at	Rhox9	reproductive homeobox 9	-2,9749	0,0797	0,6421
1426795_at	Ptprs	protein tyrosine phosphatase, receptor type, S	-2,9751	0,0797	0,6533
1421511_at	Itgb3	integrin beta 3	-2,9768	0,0797	0,7482
1450146_at	Ncs1	neuronal calcium sensor 1	-2,9776	0,0797	0,7565
1423856_at	NULL	NULL	-2,9781	0,0797	0,7196
1450438_at	Ncam1	neural cell adhesion molecule 1	-2,9781	0,0797	0,6744
1422409_at	Hes3	hairy and enhancer of split 3 (Drosophila)	-2,9783	0,0797	0,7937
1449236_at	Dll3	delta-like 3 (Drosophila)	-2,9796	0,0797	0,6935
1427527_a_at	Pthlh	parathyroid hormone-like peptide	-2,9798	0,0797	0,6796
1421527_at	Epha6	Eph receptor A6	-2,9804	0,0796	0,8517
1449756_at	Dnajc17	DnaJ (Hsp40) homolog, subfamily C, member 17	-2,9811	0,0795	0,7203
1435944_s_at	NULL	NULL	-2,9812	0,0795	0,6092
1425089_at	Kcnc4	potassium voltage gated channel, Shaw-related subfamily, member 4	-2,9818	0,0795	0,7868
1420345_at	Cldn14	claudin 14	-2,9819	0,0795	0,7378
1419539_at	Irx4	Iroquois related homeobox 4 (Drosophila)	-2,9825	0,0795	0,7771
1421166_at	Atrn	attractin	-2,9826	0,0795	0,7482
1452441_at	Phf3	PHD finger protein 3	-2,9835	0,0795	0,8127
1448030_at	NULL	NULL	-2,9846	0,0795	0,7843
1419629_at	Mesp2	mesoderm posterior 2	-2,9847	0,0795	0,8832
1426131_at	Prss44	protease, serine, 44	-2,9853	0,0795	0,6659
1433489_s_at	Fgfr2	fibroblast growth factor receptor 2	-2,9853	0,0795	0,7934
1421632_at	Kcnn3	potassium intermediate/small conductance calcium-activated channel, subfamily N, member 3	-2,9855	0,0795	0,7582
1422136_at	NULL	NULL	-2,9856	0,0795	0,7049
1449060_at	NULL	NULL	-2,9856	0,0795	0,7898
1438950_x_at	Ric8	resistance to inhibitors of cholinesterase 8 homolog (C. elegans)	-2,9877	0,0795	0,7024
1457905_at	D7Ertd59e	DNA segment, Chr 7, ERATO Doi 59, expressed	-2,9882	0,0795	0,7099
1441350_at	Fgf3	fibroblast growth factor 3	-2,9884	0,0795	0,7424
1425921_a_at	1810055G02Rik	RIKEN cDNA 1810055G02 gene	-2,9884	0,0795	0,8250
1448877_at	Dlx2	distal-less homeobox 2	-2,9896	0,0795	0,8328
1419020_at	Gif	gastric intrinsic factor	-2,9933	0,0793	0,8048
1423594_a_at	Ednrb	endothelin receptor type B	-2,9936	0,0793	0,8415
1421294_at	Hdgfl1	hepatoma derived growth factor-like 1	-2,9962	0,0792	0,7298
1454737_at	Dusp9	dual specificity phosphatase 9	-2,9971	0,0791	0,8306
1460322_at	Chst3	carbohydrate (chondroitin 6/keratan) sulfotransferase 3	-2,9976	0,0791	0,7503
1420876_a_at	sep-06	septin 6	-2,9979	0,0791	0,7831
1451298_at	Plekhh3	pleckstrin homology domain containing, family H (with MyTH4 domain) member 3	-2,9987	0,0791	0,6697
1420416_at	Sema3a	sema domain, immunoglobulin domain (Ig), short basic domain, secreted, (semaphorin) 3A	-2,9990	0,0791	0,7994
1424304_at	Tpcn2	two pore segment channel 2	-2,9991	0,0791	0,8144
1429783_at	Pdlim5	PDZ and LIM domain 5	-2,9992	0,0791	0,8629
1420853_at	Sdc3	syndecan 3	-2,9994	0,0791	0,6047
1426151_a_at	Stx3	syntaxin 3	-3,0003	0,0791	0,7693
1431074_a_at	Pitpnc1	phosphatidylinositol transfer protein, cytoplasmic 1	-3,0015	0,0791	0,7440
1452018_at	Nkx2-6	NK2 transcription factor related, locus 6 (Drosophila)	-3,0018	0,0791	0,6359
1453924_a_at	Ptgfr	prostaglandin F receptor	-3,0023	0,0791	0,8728
1420538_at	Gprc5d	G protein-coupled receptor, family C, group 5, member D	-3,0033	0,0790	0,7551
1425668_a_at	St3gal4	ST3 beta-galactoside alpha-2,3-sialyltransferase 4	-3,0058	0,0789	0,8063
1426080_a_at	Kcnq2	potassium voltage-gated channel, subfamily Q, member 2	-3,0063	0,0789	0,7225
1451006_at	Xdh	xanthine dehydrogenase	-3,0074	0,0789	0,6798
1419514_at	Pitx1	paired-like homeodomain transcription factor 1	-3,0093	0,0788	0,8269
1424744_at	Sds	serine dehydratase	-3,0101	0,0788	0,7379
1422639_at	Calcb	calcitonin-related polypeptide, beta	-3,0101	0,0788	0,7971
1428752_at	Slc5a11	solute carrier family 5 (sodium/glucose cotransporter), member 11	-3,0102	0,0788	0,8104
1419670_at	Ftcd	formiminotransferase cyclodeaminase	-3,0109	0,0788	0,6817
1420771_at	Sprr2d	small proline-rich protein 2D	-3,0113	0,0788	0,8646
1419596_at	Eda	ectodysplasin-A	-3,0116	0,0788	0,7783
1421168_at	Abcg3	ATP-binding cassette, sub-family G (WHITE), member 3	-3,0124	0,0788	0,7328
1423417_at	Smarcc1	SWI/SNF related, matrix associated, actin dependent regulator of chromatin, subfamily c, member 1	-3,0124	0,0788	0,8010
1423929_at	Ubac2	ubiquitin associated domain containing 2	-3,0127	0,0788	0,8344
1450764_at	Aoah	acyloxyacyl hydrolase	-3,0133	0,0788	0,7455
1452803_at	Glipr2	GLI pathogenesis-related 2	-3,0135	0,0788	0,6696
1415801_at	Gja1	gap junction protein, alpha 1	-3,0138	0,0788	0,7652
1419387_s_at	Muc13	mucin 13, epithelial transmembrane	-3,0141	0,0788	0,6357
1425905_at	NULL	NULL	-3,0152	0,0788	0,8483
1438014_at	Mrpl34	mitochondrial ribosomal protein L34	-3,0154	0,0788	0,7044
1452530_a_at	Runx1	runt related transcription factor 1	-3,0163	0,0788	0,6884
1448599_s_at	Miip	migration and invasion inhibitory protein	-3,0166	0,0788	0,6010

Probeset ID	Symbol	Gene Name	d-value	q-value	R-fold
1450005_x_at	Dlk2	delta-like 2 homolog (Drosophila)	-3,0177	0,0788	0,6935
1425306_at	BC027072	cDNA sequence BC027072	-3,0195	0,0787	0,8085
1417828_at	Aqp8	aquaporin 8	-3,0198	0,0787	0,7275
1450233_a_at	Rsph6a	radial spoke head 6 homolog A (Chlamydomonas)	-3,0200	0,0787	0,6540
1420536_at	Crybb2	crystallin, beta B2	-3,0204	0,0787	0,6293
1432863_a_at	Nkx2-4	NK2 transcription factor related, locus 4 (Drosophila)	-3,0222	0,0787	0,7081
1459935_at	AA517562	EST AA517562	-3,0234	0,0786	0,8019
1425922_a_at	Mycn	v-myc myelocytomatosis viral related oncogene, neuroblastoma derived (avian)	-3,0236	0,0786	0,7326
1422984_at	Clip2	CAP-GLY domain containing linker protein 2	-3,0237	0,0786	0,6849
1417944_at	Gng4	guanine nucleotide binding protein (G protein), gamma 4	-3,0241	0,0786	0,6718
1421453_at	Jph2	junctophilin 2	-3,0243	0,0786	0,6956
1426839_at	Pold3	polymerase (DNA-directed), delta 3, accessory subunit	-3,0254	0,0786	0,7471
1417184_s_at	NULL	NULL	-3,0263	0,0786	0,5919
1425273_s_at	Emp2	epithelial membrane protein 2	-3,0282	0,0786	0,8466
1438914_at	NULL	NULL	-3,0290	0,0786	0,8873
1450213_at	Pde7b	phosphodiesterase 7B	-3,0297	0,0786	0,8505
1451919_a_at	Mpl	myeloproliferative leukemia virus oncogene	-3,0297	0,0786	0,6607
1425730_at	Cacng6	calcium channel, voltage-dependent, gamma subunit 6	-3,0307	0,0786	0,6093
1427623_at	Herc2	hect (homologous to the E6-AP (UBE3A) carboxyl terminus) domain and RCC1 (CHC1)-like domain (RLD) 2	-3,0323	0,0785	0,7745
1427757_at	NULL	NULL	-3,0328	0,0785	0,6854
1450633_at	Calm4	calmodulin 4	-3,0331	0,0785	0,8288
1417940_s_at	Rad51ap1	RAD51 associated protein 1	-3,0337	0,0785	0,8075
1420773_at	Dub1	deubiquitinating enzyme 1	-3,0351	0,0784	0,7800
1425768_at	Diablo	diablo homolog (Drosophila)	-3,0372	0,0784	0,8906
1418240_at	Gbp2	guanylate binding protein 2	-3,0376	0,0784	0,8622
1421523_at	Fgf17	fibroblast growth factor 17	-3,0377	0,0784	0,5510
1421791_at	NULL	NULL	-3,0379	0,0784	0,8289
1421526_at	Tll2	tolloid-like 2	-3,0385	0,0784	0,8426
1451815_at	B3gnt4	UDP-GlcNAc:betaGal beta-1,3-N-acetylglucosaminyltransferase 4	-3,0390	0,0784	0,7230
1416992_at	NULL	NULL	-3,0397	0,0784	0,6492
1416049_at	Gldc	glycine decarboxylase	-3,0405	0,0784	0,7306
1456148_a_at	Fam100b	family with sequence similarity 100, member B	-3,0408	0,0784	0,7105
1451502_at	Pla2g10	phospholipase A2, group X	-3,0421	0,0784	0,6512
1432167_at	NULL	NULL	-3,0422	0,0784	0,8386
1421941_at	Camk4	calcium/calmodulin-dependent protein kinase IV	-3,0445	0,0784	0,7961
1422575_at	Mxd4	Max dimerization protein 4	-3,0464	0,0783	0,7617
1420802_at	Il13	interleukin 13	-3,0468	0,0783	0,8409
1419609_at	Ccr1	chemokine (C-C motif) receptor 1	-3,0473	0,0783	0,8654
1422827_x_at	Slc35a2	solute carrier family 35 (UDP-galactose transporter), member A2	-3,0476	0,0783	0,8151
1418411_at	Fbxl8	F-box and leucine-rich repeat protein 8	-3,0485	0,0783	0,6317
1450715_at	Cyp1a2	cytochrome P450, family 1, subfamily a, polypeptide 2	-3,0500	0,0783	0,8152
1420701_at	Klk1b1	kallikrein 1-related peptidase b1	-3,0500	0,0783	0,7536
1453989_at	Stxbp4	syntaxin binding protein 4	-3,0516	0,0783	0,7054
1418845_at	Proc	protein C	-3,0526	0,0783	0,7827
1448158_at	Sdc1	syndecan 1	-3,0535	0,0783	0,7301
1420761_at	Chrnd	cholinergic receptor, nicotinic, delta polypeptide	-3,0540	0,0783	0,5377
1426032_at	Nfatc2	nuclear factor of activated T-cells, cytoplasmic, calcineurin-dependent 2	-3,0545	0,0783	0,7245
1422775_at	Blk	B lymphoid kinase	-3,0563	0,0783	0,8402
1427900_at	Pip5kl1	phosphatidylinositol-4-phosphate 5-kinase-like 1	-3,0567	0,0783	0,6979
1449649_at	Atp6v1b2	ATPase, H+ transporting, lysosomal V1 subunit B2	-3,0573	0,0783	0,7928
1434777_at	Mycl1	v-myc myelocytomatosis viral oncogene homolog 1, lung carcinoma derived (avian)	-3,0574	0,0783	0,8285
1455639_at	Slc25a39	solute carrier family 25, member 39	-3,0580	0,0783	0,6974
1425636_at	Hhat	hedgehog acyltransferase	-3,0581	0,0783	0,8352
1427402_at	Polr2f	polymerase (RNA) II (DNA directed) polypeptide F	-3,0597	0,0783	0,6761
1437134_at	Gm15393	predicted gene 15393	-3,0601	0,0783	0,7082
1460364_at	Gtf2ird1	general transcription factor II I repeat domain-containing 1	-3,0605	0,0783	0,6942
1425286_at	Crygn	crystallin, gamma N	-3,0606	0,0783	0,7405
1448860_at	Rem2	rad and gem related GTP binding protein 2	-3,0611	0,0783	0,7577
1421493_a_at	Rgs20	regulator of G-protein signaling 20	-3,0627	0,0783	0,8320
1427466_at	Pigu	phosphatidylinositol glycan anchor biosynthesis, class U	-3,0635	0,0783	0,7667
1424248_at	Arpp21	cyclic AMP-regulated phosphoprotein, 21	-3,0636	0,0783	0,8615
1450538_s_at	NULL	NULL	-3,0656	0,0782	0,7344
1417427_at	Rnaseh2c	ribonuclease H2, subunit C	-3,0666	0,0782	0,7144
1455167_at	Cox8c	cytochrome c oxidase, subunit VIIIc	-3,0668	0,0782	0,6525
1449545_at	Fgf18	fibroblast growth factor 18	-3,0675	0,0782	0,6287
1450441_at	Lrrc50	leucine rich repeat containing 50	-3,0678	0,0782	0,7962
1426285_at	Lama2	laminin, alpha 2	-3,0689	0,0782	0,8988
1432426_a_at	Ube2f	ubiquitin-conjugating enzyme E2F (putative)	-3,0698	0,0782	0,7938
1449921_s_at	Cpne6	copine VI	-3,0699	0,0782	0,5413
1426791_at	Rusc2	RUN and SH3 domain containing 2	-3,0714	0,0782	0,6763
1417836_at	Gpx7	glutathione peroxidase 7	-3,0715	0,0782	0,6391
1417155_at	Mycn	v-myc myelocytomatosis viral related oncogene, neuroblastoma derived (avian)	-3,0720	0,0782	0,7788
1425295_at	Ear11	eosinophil-associated, ribonuclease A family, member 11	-3,0726	0,0782	0,7981
1438223_at	NULL	NULL	-3,0726	0,0782	0,8448
1438711_at	Pklr	pyruvate kinase liver and red blood cell	-3,0731	0,0782	0,7338

Probeset ID	***Symbol***	***Gene Name***	***d-value***	***q-value***	***R-fold***
1422709_a_at	Wdr46	WD repeat domain 46	-3,0735	0,0782	0,7340
1452305_s_at	Cenpn	centromere protein N	-3,0747	0,0782	0,9209
1421668_x_at	Speer3	spermatogenesis associated glutamate (E)-rich protein 3	-3,0747	0,0782	0,5779
1429090_at	Ggct	gamma-glutamyl cyclotransferase	-3,0753	0,0782	0,8911
1449058_at	Gli1	GLI-Kruppel family member GLI1	-3,0757	0,0782	0,7625
1434119_at	D2Wsu81e	DNA segment, Chr 2, Wayne State University 81, expressed	-3,0788	0,0781	0,7626
1428060_at	NULL	NULL	-3,0794	0,0781	0,8437
1436512_at	NULL	NULL	-3,0796	0,0781	0,7518
1460223_a_at	Epb4.9	erythrocyte protein band 4.9	-3,0799	0,0781	0,7560
1419232_a_at	Apoa1	apolipoprotein A-I	-3,0802	0,0781	0,6655
1456154_at	Zfp444	zinc finger protein 444	-3,0804	0,0781	0,6398
1420496_at	F12	coagulation factor XII (Hageman factor)	-3,0815	0,0781	0,7492
1425654_a_at	Lima1	LIM domain and actin binding 1	-3,0819	0,0781	0,6956
1425672_a_at	Trpc2	transient receptor potential cation channel, subfamily C, member 2	-3,0826	0,0781	0,7404
1455423_at	Khdc1a	KH domain containing 1A	-3,0831	0,0781	0,8385
1417151_a_at	Ntsr2	neurotensin receptor 2	-3,0844	0,0781	0,8226
1450586_at	Bdkrb1	bradykinin receptor, beta 1	-3,0876	0,0781	0,7885
1438483_at	Nos1	nitric oxide synthase 1, neuronal	-3,0886	0,0781	0,8403
1417118_a_at	Naa10	N(alpha)-acetyltransferase 10, NatA catalytic subunitNalpha acetyltransferase 10	-3,0890	0,0781	0,7404
1427465_at	Atp1a2	ATPase, Na+/K+ transporting, alpha 2 polypeptide	-3,0895	0,0781	0,7602
1449470_at	Dlx1	distal-less homeobox 1	-3,0903	0,0781	0,7220
1433941_at	Inpp5j	inositol polyphosphate 5-phosphatase J	-3,0938	0,0779	0,6570
1423611_at	Alpl	alkaline phosphatase, liver/bone/kidney	-3,0939	0,0779	0,7604
1419607_at	Tnf	tumor necrosis factor	-3,0941	0,0779	0,7516
1431396_at	Iqgap1	IQ motif containing GTPase activating protein 1	-3,0951	0,0779	0,6968
1427811_at	Tyms	thymidylate synthase	-3,0954	0,0779	0,8301
1418688_at	Calcr	calcitonin receptor	-3,0961	0,0779	0,8370
1452407_at	Spag4	sperm associated antigen 4	-3,0964	0,0779	0,5973
1419223_a_at	Dtna	dystrobrevin alpha	-3,0982	0,0779	0,7997
1430078_a_at	Ogg1	8-oxoguanine DNA-glycosylase 1	-3,1008	0,0779	0,7136
1423946_at	Pdlim2	PDZ and LIM domain 2	-3,1010	0,0778	0,7562
1425700_at	NULL	NULL	-3,1013	0,0778	0,6999
1419208_at	Map3k8	mitogen-activated protein kinase kinase kinase 8	-3,1015	0,0778	0,9093
1448064_at	NULL	NULL	-3,1015	0,0778	0,8202
1434927_at	Hspb7	heat shock protein family, member 7 (cardiovascular)	-3,1022	0,0778	0,7061
1424751_at	Abt1	activator of basal transcription 1	-3,1027	0,0778	0,8412
1422083_at	Tlr9	toll-like receptor 9	-3,1041	0,0778	0,6438
1421414_a_at	Sema6a	sema domain, transmembrane domain (TM), and cytoplasmic domain, (semaphorin) 6A	-3,1048	0,0778	0,6243
1431591_s_at	NULL	NULL	-3,1080	0,0776	0,8351
1426633_s_at	Kctd14	potassium channel tetramerisation domain containing 14	-3,1087	0,0776	0,6236
1460642_at	Traf4	TNF receptor associated factor 4	-3,1088	0,0776	0,7206
1423056_at	Nsg1	neuron specific gene family member 1	-3,1092	0,0776	0,7286
1421578_at	Ccl4	chemokine (C-C motif) ligand 4	-3,1101	0,0776	0,7154
1418870_at	4930579J09Rik	RIKEN cDNA 4930579J09 gene	-3,1107	0,0776	0,5592
1422252_a_at	Cdc25c	cell division cycle 25 homolog C (S. pombe)	-3,1125	0,0776	0,7887
1424989_at	Orai1	ORAI calcium release-activated calcium modulator 1	-3,1126	0,0776	0,8403
1426434_at	Tmem43	transmembrane protein 43	-3,1142	0,0775	0,7437
1423886_at	Lamc1	laminin, gamma 1	-3,1157	0,0774	0,7183
1417358_s_at	Sorbs1	sorbin and SH3 domain containing 1	-3,1158	0,0774	0,6994
1450624_at	Bhmt	betaine-homocysteine methyltransferase	-3,1160	0,0774	0,6720
1427093_at	Zfp707	zinc finger protein 707	-3,1178	0,0774	0,7837
1419840_at	Krt72-ps	keratin 72, pseudogene	-3,1178	0,0774	0,7699
1425061_at	Wasf3	WAS protein family, member 3	-3,1189	0,0774	0,7479
1434239_at	Rrp12	ribosomal RNA processing 12 homolog (S. cerevisiae)	-3,1195	0,0774	0,7106
1422779_at	Smpd3	sphingomyelin phosphodiesterase 3, neutral	-3,1197	0,0774	0,9207
1430406_at	Dennd1b	DENN/MADD domain containing 1B	-3,1203	0,0774	0,6479
1427813_at	Ids	iduronate 2-sulfatase	-3,1206	0,0774	0,8400
1448693_at	Gltpd1	glycolipid transfer protein domain containing 1	-3,1217	0,0774	0,8085
1455834_x_at	Tacc3	transforming, acidic coiled-coil containing protein 3	-3,1220	0,0774	0,8662
1419326_at	1700028P14Rik	RIKEN cDNA 1700028P14 gene	-3,1239	0,0773	0,7971
1426616_at	Tlcd1	TLC domain containing 1	-3,1273	0,0772	0,7796
1417096_at	Rrp15	ribosomal RNA processing 15 homolog (S. cerevisiae)	-3,1275	0,0772	0,7662
1419992_x_at	Rab3il1	RAB3A interacting protein (rabin3)-like 1	-3,1276	0,0772	0,8163
1426185_at	Cacna2d2	calcium channel, voltage-dependent, alpha 2/delta subunit 2	-3,1280	0,0771	0,8266
1421713_at	Pramel1	preferentially expressed antigen in melanoma-like 1	-3,1292	0,0771	0,7970
1426105_a_at	Ubxn11	UBX domain protein 11	-3,1296	0,0771	0,6855
1421988_at	Papss2	3'-phosphoadenosine 5'-phosphosulfate synthase 2	-3,1301	0,0771	0,7870
1426021_a_at	Cdc7	cell division cycle 7 (S. cerevisiae)	-3,1305	0,0771	0,7613
1423009_at	Sec1	secretory blood group 1	-3,1321	0,0771	0,8576
1450306_at	Zp1	zona pellucida glycoprotein 1	-3,1324	0,0771	0,7531
1424972_at	NULL	NULL	-3,1364	0,0770	0,7638
1451496_at	Mtss1	metastasis suppressor 1	-3,1364	0,0770	0,7042
1418402_at	NULL	NULL	-3,1365	0,0770	0,8889
1424964_at	Rp1	retinitis pigmentosa 1 (human)	-3,1367	0,0770	0,8673
1418544_at	Kcnip3	Kv channel interacting protein 3, calsenilin	-3,1371	0,0770	0,6944
1422990_at	Met	met proto-oncogene	-3,1372	0,0770	0,8367
1426502_s_at	Gpt	glutamic pyruvic transaminase, soluble	-3,1379	0,0770	0,7660

Probeset ID	Symbol	Gene Name	d-value	q-value	R-fold
1450475_at	Dlx3	distal-less homeobox 3	-3,1384	0,0770	0,6427
1418472_at	Aspa	aspartoacylase	-3,1385	0,0770	0,8122
1419335_at	4930441O14Rik	RIKEN cDNA 4930441O14 gene	-3,1385	0,0770	0,8255
1420774_a_at	4930583H14Rik	RIKEN cDNA 4930583H14 gene	-3,1395	0,0770	0,6966
1449451_at	Serpinb11	serine (or cysteine) peptidase inhibitor, clade B (ovalbumin), member 11	-3,1399	0,0770	0,8370
1419652_s_at	Nkain1	Na+/K+ transporting ATPase interacting 1	-3,1403	0,0770	0,7392
1422194_at	Scn5a	sodium channel, voltage-gated, type V, alpha	-3,1420	0,0770	0,7150
1422691_at	Sptlc1	serine palmitoyltransferase, long chain base subunit 1	-3,1431	0,0770	0,7990
1420716_at	Cd247	CD247 antigen	-3,1438	0,0770	0,8056
1420763_at	Klk4	kallikrein related-peptidase 4 (prostase, enamel matrix, prostate)	-3,1440	0,0770	0,7419
1421761_a_at	Barx2	BarH-like homeobox 2	-3,1478	0,0768	0,5671
1451014_at	Ror1	receptor tyrosine kinase-like orphan receptor 1	-3,1481	0,0768	0,5276
1427747_a_at	Lcn2	lipocalin 2	-3,1487	0,0768	0,8266
1425965_at	Ubc	ubiquitin C	-3,1490	0,0768	0,6227
1450359_at	Fut1	fucosyltransferase 1	-3,1496	0,0768	0,7160
1451440_at	Chodl	chondrolectin	-3,1500	0,0768	0,8029
1421086_at	Per3	period homolog 3 (Drosophila)	-3,1518	0,0768	0,6853
1428790_at	Nfam1	Nfat activating molecule with ITAM motif 1	-3,1531	0,0768	0,7822
1422923_at	Fgf3	fibroblast growth factor 3	-3,1536	0,0768	0,6322
1452452_at	NULL	NULL	-3,1538	0,0768	0,6548
1460262_a_at	1700037H04Rik	RIKEN cDNA 1700037H04 gene	-3,1539	0,0768	0,9215
1451329_at	Nudt22	nudix (nucleoside diphosphate linked moiety X)-type motif 22	-3,1545	0,0768	0,7101
1454016_at	NULL	NULL	-3,1548	0,0768	0,8666
1423352_at	Crispld1	cysteine-rich secretory protein LCCL domain containing 1	-3,1551	0,0768	0,8094
1425608_at	Dusp3	dual specificity phosphatase 3 (vaccinia virus phosphatase VH1-related)	-3,1554	0,0768	0,7268
1426197_at	NULL	NULL	-3,1568	0,0768	0,9422
1424870_at	NULL	NULL	-3,1572	0,0768	0,6895
1426038_at	NULL	NULL	-3,1592	0,0768	0,8262
1417590_at	Cyp27a1	cytochrome P450, family 27, subfamily a, polypeptide 1	-3,1604	0,0768	0,8223
1419617_at	Kcnn1	potassium intermediate/small conductance calcium-activated channel, subfamily N, member 1	-3,1614	0,0768	0,6694
1432083_a_at	Lrrc23	leucine rich repeat containing 23	-3,1615	0,0768	0,6335
1459903_at	Sema7a	sema domain, immunoglobulin domain (Ig), and GPI membrane anchor, (semaphorin) 7A	-3,1623	0,0768	0,8205
1423343_at	Slco1c1	solute carrier organic anion transporter family, member 1c1	-3,1629	0,0768	0,7309
1424469_a_at	Cpsf4	cleavage and polyadenylation specific factor 4	-3,1643	0,0767	0,7744
1448855_at	Rassf1	Ras association (RalGDS/AF-6) domain family member 1	-3,1651	0,0767	0,6240
1453389_a_at	Sh2b2	SH2B adaptor protein 2	-3,1656	0,0767	0,6111
1450556_at	Spnb1	spectrin beta 1	-3,1675	0,0767	0,6410
1460293_at	Ncs1	neuronal calcium sensor 1	-3,1689	0,0767	0,7678
1427146_at	NULL	NULL	-3,1694	0,0767	0,6725
1419415_a_at	Rarg	retinoic acid receptor, gamma	-3,1695	0,0767	0,7694
1451030_at	Akr1c21	aldo-keto reductase family 1, member C21	-3,1705	0,0767	0,8307
1421726_at	Ap4b1	adaptor-related protein complex AP-4, beta 1	-3,1707	0,0767	0,7030
1421667_at	Nmur1	neuromedin U receptor 1	-3,1710	0,0767	0,5393
1450557_at	Scn4a	sodium channel, voltage-gated, type IV, alpha	-3,1724	0,0767	0,7885
1437368_at	Gtf2h4	general transcription factor II H, polypeptide 4	-3,1730	0,0767	0,9296
1449339_at	Anapc16	anaphase promoting complex subunit 16	-3,1734	0,0767	0,8970
1455691_at	Cyp21a1	cytochrome P450, family 21, subfamily a, polypeptide 1	-3,1735	0,0767	0,8140
1432517_a_at	Nnmt	nicotinamide N-methyltransferase	-3,1741	0,0767	0,6876
1449365_at	S1pr5	sphingosine-1-phosphate receptor 5	-3,1746	0,0767	0,8418
1425728_at	Gm12185	predicted gene 12185	-3,1749	0,0767	0,6736
1424536_at	Oas1e	2'-5' oligoadenylate synthetase 1E	-3,1753	0,0767	0,7741
1449103_at	Tex101	testis expressed gene 101	-3,1757	0,0767	0,8263
1449381_a_at	Pacsin1	protein kinase C and casein kinase substrate in neurons 1	-3,1758	0,0767	0,7487
1448029_at	Tbx3	T-box 3	-3,1761	0,0767	0,8983
1452023_at	Asb16	ankyrin repeat and SOCS box-containing 16	-3,1784	0,0767	0,6974
1424631_a_at	Ighg	Immunoglobulin heavy chain (gamma polypeptide)	-3,1793	0,0767	0,7514
1420138_at	Slc19a1	solute carrier family 19 (sodium/hydrogen exchanger), member 1	-3,1806	0,0766	0,7459
1450300_at	Gabrr1	gamma-aminobutyric acid (GABA) C receptor, subunit rho 1	-3,1814	0,0766	0,7272
1420722_at	Elovl3	elongation of very long chain fatty acids (FEN1/Elo2, SUR4/Elo3, yeast)-like 3	-3,1814	0,0766	0,9088
1430979_a_at	Prdx2	peroxiredoxin 2	-3,1816	0,0766	0,4874
1455932_at	Mtdh	metadherin	-3,1832	0,0766	0,5584
1450408_at	Clcn7	chloride channel 7	-3,1834	0,0766	0,7242
1427754_a_at	Dnm1	dynamin 1	-3,1837	0,0766	0,7779
1452531_at	Runx1	runt related transcription factor 1	-3,1843	0,0766	0,6686
1451426_at	Dhx58	DEXH (Asp-Glu-X-His) box polypeptide 58	-3,1852	0,0765	0,8198
1455264_at	Fam132a	family with sequence similarity 132, member A	-3,1856	0,0765	0,6510
1448911_at	Atp4b	ATPase, H+/K+ exchanging, beta polypeptide	-3,1859	0,0765	0,7928
1437746_at	Lrrtm1	leucine rich repeat transmembrane neuronal 1	-3,1859	0,0765	0,7878
1425386_at	4833422F24Rik	RIKEN cDNA 4833422F24 gene	-3,1862	0,0765	0,6061
1426190_at	Aym1	activator of yeast meiotic promoters 1	-3,1871	0,0765	0,6991
1453806_at	Ndufb2	NADH dehydrogenase (ubiquinone) 1 beta subcomplex, 2	-3,1871	0,0765	0,7218
1430571_s_at	Armc6	armadillo repeat containing 6	-3,1875	0,0765	0,5710
1427753_at	Gm4964	predicted gene 4964	-3,1877	0,0765	0,8496
1455832_a_at	Umps	uridine monophosphate synthetase	-3,1885	0,0765	0,7799
1420328_at	Rep15	RAB15 effector protein	-3,1885	0,0765	0,6682
1421270_at	Sh3rf1	SH3 domain containing ring finger 1	-3,1903	0,0765	0,8413

Probeset ID	*Symbol*	*Gene Name*	*d-value*	*q-value*	*R-fold*
1423427_at	Adcyap1	adenylate cyclase activating polypeptide 1	-3,1904	0,0765	0,8238
1437344_x_at	Krt13	keratin 13	-3,1920	0,0765	0,5798
1428059_at	NULL	NULL	-3,1925	0,0765	0,8066
1450131_a_at	Bspry	B-box and SPRY domain containing	-3,1930	0,0765	0,8240
1450910_at	Cap2	CAP, adenylate cyclase-associated protein, 2 (yeast)	-3,1931	0,0765	0,8695
1421685_at	Clec4b1	C-type lectin domain family 4, member b1	-3,1934	0,0765	0,8701
1417748_x_at	Foxm1	forkhead box M1	-3,1948	0,0765	0,8537
1427839_at	Igh-VJ558	immunoglobulin heavy chain (J558 family)	-3,1954	0,0765	0,9100
1431604_a_at	Ccdc97	coiled-coil domain containing 97	-3,1963	0,0765	0,7465
1419408_at	Six6	sine oculis-related homeobox 6 homolog (Drosophila)	-3,1968	0,0765	0,8792
1421314_at	Cttn	cortactin	-3,1976	0,0765	0,8035
1452618_at	Cdk5rap2	CDK5 regulatory subunit associated protein 2	-3,1992	0,0765	0,8187
1450765_a_at	Pde6h	phosphodiesterase 6H, cGMP-specific, cone, gamma	-3,1994	0,0765	0,8272
1452418_at	NULL	NULL	-3,1998	0,0765	0,7276
1421114_a_at	Epyc	epiphycan	-3,2000	0,0765	0,8186
1420755_a_at	Park2	Parkinson disease (autosomal recessive, juvenile) 2, parkin	-3,2012	0,0765	0,7225
1451955_a_at	Cacna2d2	calcium channel, voltage-dependent, alpha 2/delta subunit 2	-3,2019	0,0765	0,6498
1436097_x_at	Arhgap9	Rho GTPase activating protein 9	-3,2031	0,0765	0,7014
1427503_at	AI324046	expressed sequence AI324046	-3,2043	0,0765	0,7714
1450361_at	Prop1	paired like homeodomain factor 1	-3,2060	0,0765	0,7946
1418052_at	Mvk	mevalonate kinase	-3,2075	0,0765	0,8023
1452171_at	Grwd1	glutamate-rich WD repeat containing 1	-3,2079	0,0765	0,8093
1424361_at	BC019943	cDNA sequence BC019943	-3,2089	0,0765	0,7600
1448316_at	NULL	NULL	-3,2091	0,0765	0,8806
1450473_at	Pcdh12	protocadherin 12	-3,2092	0,0765	0,6912
1450867_at	Mrpl17	mitochondrial ribosomal protein L17	-3,2097	0,0765	0,7244
1424793_a_at	Pbp2	phosphatidylethanolamine binding protein 2	-3,2098	0,0765	0,6894
1425526_a_at	Prrx1	paired related homeobox 1	-3,2098	0,0765	0,8047
1452571_at	Tuba-rs1	tubulin alpha, related sequence 1	-3,2102	0,0765	0,7156
1420676_at	Lce1a1	late cornified envelope 1A1	-3,2105	0,0765	0,8922
1419481_at	Sell	selectin, lymphocyte	-3,2121	0,0765	0,7438
1421641_at	Slc6a2	solute carrier family 6 (neurotransmitter transporter, noradrenalin), member 2	-3,2135	0,0764	0,8540
1448713_at	Stat4	signal transducer and activator of transcription 4	-3,2138	0,0764	0,7976
1427566_at	Wfikkn1	WAP, FS, Ig, KU, and NTR-containing protein 1	-3,2145	0,0764	0,6680
1423326_at	Entpd1	ectonucleoside triphosphate diphosphohydrolase 1	-3,2157	0,0764	0,8345
1426181_a_at	Il24	interleukin 24	-3,2174	0,0764	0,8008
1427082_at	Sdr42e1	short chain dehydrogenase/reductase family 42E, member 1	-3,2176	0,0764	0,8126
1418598_at	Ubox5	U box domain containing 5	-3,2180	0,0764	0,7489
1420600_at	Tpo	thyroid peroxidase	-3,2183	0,0764	0,8114
1427547_a_at	Slc26a3	solute carrier family 26, member 3	-3,2196	0,0763	0,8193
1421124_at	Cdk5r1	cyclin-dependent kinase 5, regulatory subunit 1 (p35)	-3,2198	0,0763	0,7929
1455873_a_at	Vps18	vacuolar protein sorting 18 (yeast)	-3,2199	0,0763	0,6739
1415955_x_at	Prm1	protamine 1	-3,2201	0,0763	0,7200
1427633_a_at	Pappa	pregnancy-associated plasma protein A	-3,2206	0,0763	0,7634
1419599_s_at	Ms4a6d	membrane-spanning 4-domains, subfamily A, member 6D	-3,2206	0,0763	0,8424
1421738_at	Gabra2	gamma-aminobutyric acid (GABA) A receptor, subunit alpha 2	-3,2207	0,0763	0,8699
1450339_a_at	Bcl11b	B-cell leukemia/lymphoma 11B	-3,2209	0,0763	0,7211
1421265_a_at	Rbm38	RNA binding motif protein 38	-3,2229	0,0763	0,7112
1427715_a_at	Nt5c1b	5'-nucleotidase, cytosolic IB	-3,2231	0,0763	0,8814
1418278_at	Apoc3	apolipoprotein C-III	-3,2236	0,0763	0,7190
1426132_at	NULL	NULL	-3,2246	0,0763	0,8148
1449832_at	1700091H14Rik	RIKEN cDNA 1700091H14 gene	-3,2249	0,0763	0,8208
1451766_at	Hmgcr	3-hydroxy-3-methylglutaryl-Coenzyme A reductase	-3,2291	0,0763	0,9014
1454750_a_at	Dcaf15	DDB1 and CUL4 associated factor 15	-3,2293	0,0763	0,8365
1451258_at	Psca	prostate stem cell antigen	-3,2293	0,0763	0,6540
1424849_at	Wdr62	WD repeat domain 62	-3,2295	0,0763	0,6623
1452491_at	Gm11767	predicted gene 11767	-3,2300	0,0763	0,7762
1460271_at	Trem3	triggering receptor expressed on myeloid cells 3	-3,2318	0,0763	0,6978
1419594_at	Ctsg	cathepsin G	-3,2322	0,0763	0,8094
1425337_at	Slc12a5	solute carrier family 12, member 5	-3,2325	0,0763	0,7374
1424592_a_at	Dnase1	deoxyribonuclease I	-3,2326	0,0763	0,6064
1430734_at	Cyb5r3	cytochrome b5 reductase 3	-3,2327	0,0763	0,7156
1425946_at	Gstm7	glutathione S-transferase, mu 7	-3,2328	0,0763	0,8012
1432849_at	NULL	NULL	-3,2332	0,0763	0,6967
1448207_at	Lasp1	LIM and SH3 protein 1	-3,2347	0,0763	0,6358
1450256_at	Cer1	cerberus 1 homolog (Xenopus laevis)	-3,2349	0,0763	0,8741
1420910_at	Ppap2c	phosphatidic acid phosphatase type 2C	-3,2360	0,0763	0,7132
1449657_at	Gm6787	proteasome (prosome, macropain) subunit, beta type 7 pseudogene	-3,2373	0,0763	0,7254
1426108_s_at	Cacnb1	calcium channel, voltage-dependent, beta 1 subunit	-3,2384	0,0763	0,7212
1427768_s_at	Myl3	myosin, light polypeptide 3	-3,2385	0,0763	0,6275
1424742_at	Creb3	cAMP responsive element binding protein 3	-3,2390	0,0763	0,8119
1427710_at	Ccdc114	coiled-coil domain containing 114	-3,2391	0,0763	0,7935
1451246_s_at	Aurkb	aurora kinase B	-3,2399	0,0763	0,8074
1420396_at	Cd160	CD160 antigen	-3,2401	0,0763	0,8114
1452590_a_at	NULL	NULL	-3,2406	0,0763	0,8157
1460237_at	Trim8	tripartite motif-containing 8	-3,2432	0,0763	0,6683
1451241_at	Lamb1	laminin B1	-3,2432	0,0763	0,6708
1420163_at	AA511261	expressed sequence AA511261	-3,2465	0,0763	0,8864

Probeset ID	***Symbol***	***Gene Name***	***d-value***	***q-value***	***R-fold***
1419113_at	Ap1g2	adaptor protein complex AP-1, gamma 2 subunit	-3,2473	0,0763	0,7416
1426802_at	sep-08	septin 8	-3,2475	0,0763	0,8217
1449129_a_at	Kcnip3	Kv channel interacting protein 3, calsenilin	-3,2483	0,0763	0,7057
1449005_at	Slc16a3	solute carrier family 16 (monocarboxylic acid transporters), member 3	-3,2490	0,0763	0,6420
1457738_at	Lsm14a	LSM14 homolog A (SCD6, S. cerevisiae)	-3,2499	0,0763	0,8898
1415936_at	Bcar3	breast cancer anti-estrogen resistance 3	-3,2500	0,0763	0,7838
1426970_a_at	NULL	NULL	-3,2514	0,0763	0,7123
1435420_at	Slc4a5	solute carrier family 4, sodium bicarbonate cotransporter, member 5	-3,2530	0,0763	0,9012
1419836_at	NULL	NULL	-3,2546	0,0763	0,6917
1451180_a_at	Nt5c3l	5'-nucleotidase, cytosolic III-like	-3,2549	0,0763	0,7550
1418833_at	Agxt	alanine-glyoxylate aminotransferase	-3,2563	0,0763	0,7615
1421226_at	Trem2	triggering receptor expressed on myeloid cells 2	-3,2566	0,0763	0,6104
1427697_a_at	Trp73	transformation related protein 73	-3,2573	0,0763	0,6022
1422667_at	Krt15	keratin 15	-3,2596	0,0763	0,5700
1440499_at	D9Ertd26e	DNA segment, Chr 9, ERATO Doi 26, expressed	-3,2605	0,0763	0,7985
1452250_a_at	Col6a2	collagen, type VI, alpha 2	-3,2614	0,0763	0,8596
1421718_at	Strm	striamin	-3,2632	0,0763	0,8443
1426203_at	Krtap16-4	keratin associated protein 16-4	-3,2633	0,0763	0,7724
1448906_at	Cdh16	cadherin 16	-3,2638	0,0763	0,7517
1422236_at	Ttc39d	tetratricopeptide repeat domain 39D	-3,2647	0,0763	0,7910
1418555_x_at	Spic	Spi-C transcription factor (Spi-1/PU.1 related)	-3,2653	0,0763	0,7693
1419876_at	2810449G22Rik	RIKEN cDNA 2810449G22 gene	-3,2662	0,0763	0,7167
1421381_a_at	Col9a1	collagen, type IX, alpha 1	-3,2670	0,0763	0,6274
1420336_at	Adamtsl5	ADAMTS-like 5	-3,2676	0,0762	0,6337
1448154_at	Ndrg2	N-myc downstream regulated gene 2	-3,2684	0,0762	0,8159
1435685_x_at	Abcc5	ATP-binding cassette, sub-family C (CFTR/MRP), member 5	-3,2689	0,0762	0,9398
1448519_at	Tead2	TEA domain family member 2	-3,2691	0,0762	0,7153
AFFX-TrpnX-5_at	NULL	NULL	-3,2701	0,0762	0,9325
1422306_at	Lcn3	lipocalin 3	-3,2702	0,0762	0,6486
1416954_at	Slc25a10	solute carrier family 25 (mitochondrial carrier, dicarboxylate transporter), member 10	-3,2710	0,0762	0,7547
1420700_s_at	Folr4	folate receptor 4 (delta)	-3,2732	0,0762	0,8334
1416820_at	Cdc37	cell division cycle 37 homolog (S. cerevisiae)	-3,2750	0,0762	0,7876
1424837_at	Rnf113a1	ring finger protein 113A1	-3,2755	0,0762	0,8075
1450334_at	Il21	interleukin 21	-3,2791	0,0762	0,9199
1449844_at	Slco1a1	solute carrier organic anion transporter family, member 1a1	-3,2808	0,0762	0,8681
1423497_at	Klhl10	kelch-like 10 (Drosophila)	-3,2809	0,0762	0,6786
1422499_at	Lima1	LIM domain and actin binding 1	-3,2822	0,0762	0,7291
1452404_at	Phactr2	phosphatase and actin regulator 2	-3,2826	0,0762	0,8653
1422560_at	NULL	NULL	-3,2833	0,0762	0,6365
1422373_at	Olfr71	olfactory receptor 71	-3,2844	0,0762	0,6735
1425220_x_at	NULL	NULL	-3,2845	0,0762	0,6219
1451546_s_at	Tmem40	transmembrane protein 40	-3,2860	0,0762	0,7028
1449481_at	Slc25a13	solute carrier family 25 (mitochondrial carrier, adenine nucleotide translocator), member 13	-3,2860	0,0762	0,8252
1422323_a_at	Lbx1	ladybird homeobox homolog 1 (Drosophila)	-3,2878	0,0762	0,6667
1421916_at	Pdgfra	platelet derived growth factor receptor, alpha polypeptide	-3,2885	0,0762	0,6668
1418683_at	Lin7b	lin-7 homolog B (C. elegans)	-3,2893	0,0762	0,6870
1450278_at	Tacr3	tachykinin receptor 3	-3,2894	0,0762	0,8346
1449498_at	Marco	macrophage receptor with collagenous structure	-3,2909	0,0762	0,6875
1417643_at	Rsph1	radial spoke head 1 homolog (Chlamydomonas)	-3,2914	0,0762	0,6167
1456075_at	Prkd2	protein kinase D2	-3,2917	0,0762	0,7387
1422776_at	Serpinb8	serine (or cysteine) peptidase inhibitor, clade B, member 8	-3,2922	0,0762	0,8622
1427920_at	Phf19	PHD finger protein 19	-3,2922	0,0762	0,6636
1419818_x_at	Ankzf1	ankyrin repeat and zinc finger domain containing 1	-3,2926	0,0762	0,7652
1427118_at	5430421N21Rik	RIKEN cDNA 5430421N21 gene	-3,2929	0,0762	0,6681
1460318_at	Csrp3	cysteine and glycine-rich protein 3	-3,2933	0,0762	0,7471
1423562_at	Prrt1	proline-rich transmembrane protein 1	-3,2936	0,0762	0,7073
1450587_at	H2-M10.1	histocompatibility 2, M region locus 10.1	-3,2943	0,0762	0,7193
1454504_at	Kcnip1	Kv channel-interacting protein 1	-3,2951	0,0762	0,6884
1431973_at	sep-06	septin 6	-3,2953	0,0762	0,8481
1427528_a_at	Epha7	Eph receptor A7	-3,2953	0,0762	0,8502
1426196_at	Igh-6	immunoglobulin heavy chain 6 (heavy chain of IgM)	-3,2960	0,0762	0,8742
1450311_at	Slc8a3	solute carrier family 8 (sodium/calcium exchanger), member 3	-3,2982	0,0762	0,8499
1420350_at	Lce1a2	late cornified envelope 1A2	-3,3002	0,0761	0,7192
1427354_at	Hoxa4	homeobox A4	-3,3004	0,0761	0,7851
1451191_at	Crabp2	cellular retinoic acid binding protein II	-3,3011	0,0761	0,5581
1424566_s_at	Polr3d	polymerase (RNA) III (DNA directed) polypeptide D	-3,3021	0,0761	0,6569
1424011_at	Aqp9	aquaporin 9	-3,3028	0,0761	0,7162
1449205_at	Ovol2	ovo-like 2 (Drosophila)	-3,3042	0,0760	0,7241
1427366_at	Krtap3-1	keratin associated protein 3-1	-3,3053	0,0760	0,6549
1449516_a_at	Rgs3	regulator of G-protein signaling 3	-3,3053	0,0760	0,7606
1437340_x_at	Gkn1	gastrokine 1	-3,3059	0,0760	0,6157
1417884_at	Slc16a6	solute carrier family 16 (monocarboxylic acid transporters), member 6	-3,3067	0,0760	0,6648
1422201_at	H2-Ob	histocompatibility 2, O region beta locus	-3,3072	0,0760	0,5772
1419520_at	Nat8	N-acetyltransferase 8 (GCN5-related, putative)	-3,3073	0,0760	0,6537
1424978_at	Odf4	outer dense fiber of sperm tails 4	-3,3075	0,0760	0,8145
1420749_a_at	Pou6f1	POU domain, class 6, transcription factor 1	-3,3087	0,0760	0,7864
1460386_a_at	Slc1a1	solute carrier family 1 (neuronal/epithelial high affinity glutamate	-3,3113	0,0760	0,6208

Probeset ID	Symbol	Gene Name	d-value	q-value	R-fold
		transporter, system Xag), member 1			
1427823_at	NULL	NULL	-3,3125	0,0760	0,6820
1419426_s_at	NULL	NULL	-3,3129	0,0760	0,6675
1420423_at	Tcl1b4	T-cell leukemia/lymphoma 1B, 4	-3,3142	0,0760	0,6825
1426072_at	Cmklr1	chemokine-like receptor 1	-3,3161	0,0760	0,7855
1419202_at	Cst7	cystatin F (leukocystatin)	-3,3161	0,0760	0,8581
1417614_at	Ckm	creatine kinase, muscle	-3,3166	0,0760	0,7639
1421480_a_at	Adarb1	adenosine deaminase, RNA-specific, B1	-3,3173	0,0760	0,7062
1425435_at	Msr1	macrophage scavenger receptor 1	-3,3173	0,0760	0,7644
1417758_at	Itga2b	integrin alpha 2b	-3,3174	0,0760	0,7164
1442597_at	C79122	expressed sequence C79122	-3,3179	0,0760	0,7623
1451341_s_at	NULL	NULL	-3,3189	0,0760	0,7848
1425035_s_at	Dnmt3l	DNA (cytosine-5-)-methyltransferase 3-like	-3,3220	0,0760	0,7937
1419518_at	Tuba8	tubulin, alpha 8	-3,3222	0,0760	0,6361
1422377_at	Vmn1r44	vomeronasal 1 receptor 44	-3,3225	0,0760	0,5781
1422107_at	2410066E13Rik	RIKEN cDNA 2410066E13 gene	-3,3226	0,0760	0,7372
1419240_at	Tex14	testis expressed gene 14	-3,3240	0,0760	0,7241
1460277_at	Dmbx1	diencephalon/mesencephalon homeobox 1	-3,3244	0,0760	0,6781
1419316_s_at	Gnb1l	guanine nucleotide binding protein (G protein), beta polypeptide 1-like	-3,3248	0,0760	0,8327
1448383_at	Mmp14	matrix metallopeptidase 14 (membrane-inserted)	-3,3258	0,0760	0,7362
1435683_a_at	Abcc5	ATP-binding cassette, sub-family C (CFTR/MRP), member 5	-3,3265	0,0760	0,7244
1422086_at	Tbx19	T-box 19	-3,3283	0,0760	0,5866
1417554_at	Hsd3b4	hydroxy-delta-5-steroid dehydrogenase, 3 beta- and steroid delta-isomerase 4	-3,3290	0,0760	0,8160
1416646_at	Afp	alpha fetoprotein	-3,3305	0,0760	0,8794
1426990_at	Cubn	cubilin (intrinsic factor-cobalamin receptor)	-3,3311	0,0760	0,8074
1438509_at	Psme3	proteaseome (prosome, macropain) 28 subunit, 3	-3,3319	0,0760	0,6536
1426024_a_at	Dbn1	drebrin 1	-3,3321	0,0760	0,5861
1421621_at	Rasgrf2	RAS protein-specific guanine nucleotide-releasing factor 2	-3,3336	0,0760	0,7137
1432628_at	Cbx3	chromobox homolog 3 (Drosophila HP1 gamma)	-3,3348	0,0760	0,6765
1431240_at	Clec2h	C-type lectin domain family 2, member h	-3,3355	0,0760	0,8880
1420414_at	Hoxa11	homeobox A11	-3,3363	0,0760	0,8941
1455239_at	6330512M04Rik	RIKEN cDNA 6330512M04 gene	-3,3379	0,0760	0,6699
1451088_a_at	Oxa1l	oxidase assembly 1-like	-3,3379	0,0760	0,7970
1433517_at	Myeov2	myeloma overexpressed 2	-3,3384	0,0760	0,8671
1427328_a_at	Clasp2	CLIP associating protein 2	-3,3384	0,0760	0,7125
1422634_a_at	Vsig2	V-set and immunoglobulin domain containing 2	-3,3388	0,0760	0,7022
1421131_a_at	Zfp111	zinc finger protein 111	-3,3398	0,0760	0,8583
1419980_at	NULL	NULL	-3,3418	0,0759	0,8714
1423560_at	Nell2	NEL-like 2 (chicken)	-3,3435	0,0759	0,5448
1424889_at	Nupl2	nucleoporin like 2	-3,3448	0,0759	0,8114
1422113_at	Slc5a5	solute carrier family 5 (sodium iodide symporter), member 5	-3,3466	0,0759	0,6761
1425827_at	Nkx2-3	NK2 transcription factor related, locus 3 (Drosophila)	-3,3480	0,0759	0,7574
1422646_at	Mga	MAX gene associated	-3,3481	0,0759	0,7883
1420741_x_at	Lce1i	late cornified envelope 1I	-3,3488	0,0759	0,6803
1427609_at	Sox14	SRY-box containing gene 14	-3,3534	0,0758	0,5933
1460663_at	Cckbr	cholecystokinin B receptor	-3,3538	0,0758	0,6004
1450507_at	Pou2f3	POU domain, class 2, transcription factor 3	-3,3542	0,0757	0,5848
1419203_at	Gm16516	predicted gene, Gm16516	-3,3545	0,0757	0,7820
1449955_at	Cacna1f	calcium channel, voltage-dependent, alpha 1F subunit	-3,3549	0,0757	0,6741
1424931_s_at	NULL	NULL	-3,3564	0,0757	0,8313
1435172_at	Eomes	eomesodermin homolog (Xenopus laevis)	-3,3572	0,0757	0,8729
1435418_at	Slc22a8	solute carrier family 22 (organic anion transporter), member 8	-3,3582	0,0757	0,8431
1421154_at	Hcn2	hyperpolarization-activated, cyclic nucleotide-gated K+ 2	-3,3595	0,0757	0,7294
1417693_a_at	Gab1	growth factor receptor bound protein 2-associated protein 1	-3,3605	0,0757	0,6736
1451781_at	Nfatc2ip	nuclear factor of activated T-cells, cytoplasmic, calcineurin-dependent 2 interacting protein	-3,3607	0,0757	0,7142
1422421_at	Defa-rs12	defensin, alpha, related sequence 12	-3,3613	0,0757	0,7575
1421747_at	Esrrg	estrogen-related receptor gamma	-3,3623	0,0757	0,7627
1423968_at	Ugt3a2	UDP glycosyltransferases 3 family, polypeptide A2	-3,3627	0,0757	0,8701
1422329_a_at	NULL	NULL	-3,3629	0,0757	0,8217
1416077_at	Adm	adrenomedullin	-3,3629	0,0757	0,7322
1449654_s_at	C77545	expressed sequence C77545	-3,3638	0,0757	0,7700
1425073_at	Plekhg6	pleckstrin homology domain containing, family G (with RhoGef domain) member 6	-3,3641	0,0757	0,6784
1439199_at	Gm2769	predicted gene 2769	-3,3645	0,0757	0,6592
1418754_at	Adcy8	adenylate cyclase 8	-3,3655	0,0757	0,7308
1450200_s_at	NULL	NULL	-3,3660	0,0757	0,6125
1416677_at	Apoh	apolipoprotein H	-3,3665	0,0757	0,7795
1419336_at	Lcn5	lipocalin 5	-3,3672	0,0757	0,7834
1448164_at	Klhdc3	kelch domain containing 3	-3,3674	0,0757	0,6883
1418882_at	Nlrp5	NLR family, pyrin domain containing 5	-3,3688	0,0757	0,7255
1436639_at	Bola2	bolA-like 2 (E. coli)	-3,3689	0,0757	0,6083
1418903_at	Aqp2	aquaporin 2	-3,3696	0,0757	0,5147
1448677_at	Cox4nb	COX4 neighbor	-3,3702	0,0756	0,7996
1450340_a_at	Clcnkb	chloride channel Kb	-3,3719	0,0756	0,8267
1451118_a_at	Fam53a	family with sequence similarity 53, member A	-3,3722	0,0756	0,7897
1432410_a_at	Bmp7	bone morphogenetic protein 7	-3,3730	0,0756	0,8121
1431877_a_at	Grhl2	grainyhead-like 2 (Drosophila)	-3,3738	0,0756	0,5476

Probeset ID	Symbol	Gene Name	d-value	q-value	R-fold
1450296_at	Klrb1a	killer cell lectin-like receptor subfamily B member 1A	-3,3768	0,0755	0,8766
1420170_at	Myh9	myosin, heavy polypeptide 9, non-muscle	-3,3774	0,0755	0,5247
1453278_a_at	Clip4	CAP-GLY domain containing linker protein family, member 4	-3,3787	0,0755	0,7413
1420168_at	AA517841	expressed sequence AA517841	-3,3795	0,0755	0,7019
1450310_at	Grid2ip	glutamate receptor, ionotropic, delta 2 (Grid2) interacting protein 1	-3,3796	0,0755	0,6650
1417856_at	Relb	avian reticuloendotheliosis viral (v-rel) oncogene related B	-3,3840	0,0755	0,6498
1452470_at	Cep350	centrosomal protein 350	-3,3842	0,0755	0,7676
1424809_at	Crb3	crumbs homolog 3 (Drosophila)	-3,3849	0,0755	0,8033
1419724_at	Edar	ectodysplasin-A receptor	-3,3861	0,0755	0,8109
1427827_at	NULL	NULL	-3,3873	0,0755	0,8213
1419580_at	Dlg4	discs, large homolog 4 (Drosophila)	-3,3890	0,0755	0,7113
1422299_a_at	Styxl1	serine/threonine/tyrosine interacting-like 1	-3,3902	0,0755	0,8515
1451585_x_at	Spsb2	splA/ryanodine receptor domain and SOCS box containing 2	-3,3931	0,0755	0,8280
1417812_a_at	Lamb3	laminin, beta 3	-3,3941	0,0755	0,7634
1426866_at	Chst14	carbohydrate (N-acetylgalactosamine 4-0) sulfotransferase 14	-3,3952	0,0755	0,7397
1419979_s_at	Creb3	cAMP responsive element binding protein 3	-3,4011	0,0754	0,5449
1430259_at	Tnfrsf11a	tumor necrosis factor receptor superfamily, member 11a	-3,4019	0,0754	0,7876
1419949_at	Tmem38b	transmembrane protein 38B	-3,4022	0,0754	0,7690
1430111_a_at	Bcat1	branched chain aminotransferase 1, cytosolic	-3,4026	0,0754	0,8508
1420222_at	NULL	NULL	-3,4034	0,0754	0,7638
1426201_at	NULL	NULL	-3,4037	0,0754	0,8714
1448951_at	Tnfrsf1b	tumor necrosis factor receptor superfamily, member 1b	-3,4059	0,0754	0,6192
1450443_at	Ptbp1	polypyrimidine tract binding protein 1	-3,4060	0,0754	0,7902
1428135_a_at	Eef1d	eukaryotic translation elongation factor 1 delta (guanine nucleotide exchange protein)	-3,4075	0,0753	0,6494
1449693_at	Map3k7	mitogen-activated protein kinase kinase kinase 7	-3,4082	0,0753	0,7685
1417034_at	Trappc6a	trafficking protein particle complex 6A	-3,4093	0,0753	0,7257
1425650_at	Tle4	transducin-like enhancer of split 4, homolog of Drosophila E(spl)	-3,4111	0,0753	0,8955
1458613_at	Arrdc1	arrestin domain containing 1	-3,4117	0,0753	0,5549
1450088_a_at	Mobp	myelin-associated oligodendrocytic basic protein	-3,4122	0,0753	0,7870
1417283_at	Lynx1	Ly6/neurotoxin 1	-3,4130	0,0753	0,7978
1421663_at	Dhrs2	dehydrogenase/reductase member 2	-3,4141	0,0753	0,7008
1422322_at	Chml	choroideremia-like	-3,4224	0,0752	0,8147
1421535_a_at	Pde4a	phosphodiesterase 4A, cAMP specific	-3,4235	0,0752	0,7249
1422970_at	Mxd3	Max dimerization protein 3	-3,4237	0,0752	0,7418
1450448_at	Stc1	stanniocalcin 1	-3,4240	0,0752	0,8067
1450830_a_at	Pde6c	phosphodiesterase 6C, cGMP specific, cone, alpha prime	-3,4277	0,0751	0,7315
1448377_at	Slpi	secretory leukocyte peptidase inhibitor	-3,4291	0,0751	0,6454
1449496_at	2010109I03Rik	RIKEN cDNA 2010109I03 gene	-3,4292	0,0751	0,8466
1452330_a_at	Mxra8	matrix-remodelling associated 8	-3,4298	0,0751	0,7287
1427657_at	Ccdc134	coiled-coil domain containing 134	-3,4307	0,0751	0,7360
1460280_at	NULL	NULL	-3,4310	0,0751	0,8019
1426056_at	Pigq	phosphatidylinositol glycan anchor biosynthesis, class Q	-3,4318	0,0751	0,6512
1419391_at	Myog	myogenin	-3,4327	0,0751	0,6437
1416025_at	Fgg	fibrinogen gamma chain	-3,4343	0,0751	0,7103
1418203_at	Pmaip1	phorbol-12-myristate-13-acetate-induced protein 1	-3,4364	0,0750	0,8233
1420176_x_at	Igll1	immunoglobulin lambda-like polypeptide 1	-3,4372	0,0750	0,4835
1421401_at	Kcnmb1	potassium large conductance calcium-activated channel, subfamily M, beta member 1	-3,4388	0,0750	0,9159
1416518_at	H1foo	H1 histone family, member O, oocyte-specific	-3,4394	0,0750	0,6047
1427356_at	Fam89a	family with sequence similarity 89, member A	-3,4398	0,0750	0,8381
1456694_x_at	Ptpn6	protein tyrosine phosphatase, non-receptor type 6	-3,4401	0,0750	0,8444
1416554_at	NULL	NULL	-3,4403	0,0750	0,9042
1450508_at	Foxn1	forkhead box N1	-3,4404	0,0750	0,7331
1421699_at	Enam	enamelin	-3,4407	0,0750	0,8178
1427453_at	BC018473	cDNA sequence BC018473	-3,4432	0,0750	0,8783
1422916_at	Fgf21	fibroblast growth factor 21	-3,4443	0,0750	0,7220
1420287_at	NULL	NULL	-3,4450	0,0750	0,7348
1439283_at	Osbpl9	oxysterol binding protein-like 9	-3,4457	0,0750	0,8039
1419651_at	Nkain1	Na+/K+ transporting ATPase interacting 1	-3,4468	0,0750	0,7158
1451808_at	Kcnj4	potassium inwardly-rectifying channel, subfamily J, member 4	-3,4488	0,0750	0,6503
1419634_a_at	Ghrh	growth hormone releasing hormone	-3,4505	0,0750	0,7400
1420156_at	AA408954	expressed sequence AA408954	-3,4509	0,0750	0,8232
1427506_at	Ppil5	peptidylprolyl isomerase (cyclophilin) like 5	-3,4514	0,0750	0,7632
1437159_at	Glb1	galactosidase, beta 1	-3,4526	0,0750	0,7045
1420421_s_at	Klrb1b	killer cell lectin-like receptor subfamily B member 1B	-3,4535	0,0750	0,8950
1417708_at	Syt3	synaptotagmin III	-3,4540	0,0750	0,8163
1439287_at	NULL	NULL	-3,4551	0,0750	0,6123
1418001_at	Itm2b	integral membrane protein 2B	-3,4597	0,0749	0,8152
1422543_at	Ropn1	ropporin, rhophilin associated protein 1	-3,4604	0,0749	0,7957
1420735_at	Gabrr2	gamma-aminobutyric acid (GABA) C receptor, subunit rho 2	-3,4606	0,0749	0,7639
1421451_at	Crb1	crumbs homolog 1 (Drosophila)	-3,4615	0,0749	0,6805
1421360_at	Inpp4a	inositol polyphosphate-4-phosphatase, type I	-3,4615	0,0749	0,7327
1429982_at	Rimklb	ribosomal modification protein rimK-like family member B	-3,4617	0,0749	0,8216
1452484_at	Car7	carbonic anhydrase 7	-3,4635	0,0749	0,7309
1421885_at	Sos1	son of sevenless homolog 1 (Drosophila)	-3,4658	0,0749	0,7614
1422922_at	Recql4	RecQ protein-like 4	-3,4661	0,0749	0,6812
1418184_at	Cenpm	centromere protein M	-3,4678	0,0749	0,8213
1459880_at	Hnrnpab	heterogeneous nuclear ribonucleoprotein A/B	-3,4691	0,0749	0,8540

Probeset ID	Symbol	Gene Name	d-value	q-value	R-fold
1455857_a_at	Rab2b	RAB2B, member RAS oncogene family	-3,4704	0,0749	0,6741
1418639_at	Sftpc	surfactant associated protein C	-3,4719	0,0749	0,6676
1422466_at	Nxn	nucleoredoxin	-3,4730	0,0749	0,8533
1420730_a_at	Tcp11	t-complex protein 11	-3,4752	0,0749	0,6926
1448609_at	Tst	thiosulfate sulfurtransferase, mitochondrial	-3,4763	0,0749	0,7678
1426096_at	Prmt7	protein arginine N-methyltransferase 7	-3,4786	0,0749	0,8553
1421036_at	Npas2	neuronal PAS domain protein 2	-3,4790	0,0749	0,7212
1451864_at	Cacng8	calcium channel, voltage-dependent, gamma subunit 8	-3,4806	0,0748	0,6466
1450800_at	Syt8	synaptotagmin VIII	-3,4829	0,0748	0,6383
1439121_at	NULL	NULL	-3,4837	0,0748	0,8601
1421538_at	Kcnd1	potassium voltage-gated channel, Shal-related family, member 1	-3,4845	0,0748	0,7264
1438609_x_at	Tnni2	troponin I, skeletal, fast 2	-3,4845	0,0748	0,5534
1449326_x_at	Saa2	serum amyloid A 2	-3,4851	0,0748	0,7732
1421890_at	St3gal2	ST3 beta-galactoside alpha-2,3-sialyltransferase 2	-3,4855	0,0748	0,6456
1424173_at	Tmem48	transmembrane protein 48	-3,4863	0,0747	0,8815
1420588_at	Spinlw1	serine protease inhibitor-like, with Kunitz and WAP domains 1 (eppin)	-3,4879	0,0747	0,8099
1416051_at	C2	complement component 2 (within H-2S)	-3,4892	0,0747	0,6879
1426766_at	6330403K07Rik	RIKEN cDNA 6330403K07 gene	-3,4905	0,0747	0,8348
1420573_at	Hoxd1	homeobox D1	-3,4921	0,0747	0,9286
1425150_at	Acnat2	acyl-coenzyme A amino acid N-acyltransferase 2	-3,4923	0,0747	0,7607
1451194_at	Aldob	aldolase B, fructose-bisphosphate	-3,4923	0,0747	0,6581
1418623_at	Rab2a	RAB2A, member RAS oncogene family	-3,4926	0,0747	0,7248
1449593_at	NULL	NULL	-3,4927	0,0747	0,7841
1418620_at	Phox2a	paired-like homeobox 2a	-3,4934	0,0747	0,8762
1460371_at	Hspa12b	heat shock protein 12B	-3,4975	0,0746	0,8171
1425901_at	Nfatc2	nuclear factor of activated T-cells, cytoplasmic, calcineurin-dependent 2	-3,4976	0,0746	0,5808
1450564_x_at	Ifna1	interferon alpha 1	-3,4979	0,0746	0,5601
1425269_at	Apbb1ip	amyloid beta (A4) precursor protein-binding, family B, member 1 interacting protein	-3,4987	0,0746	0,5997
1417616_at	St6galnac2	ST6 (alpha-N-acetyl-neuraminyl-2,3-beta-galactosyl-1,3)-N-acetylgalactosaminide alpha-2,6-sialyltransferase 2	-3,5009	0,0745	0,7571
1419233_x_at	Apoa1	apolipoprotein A-I	-3,5012	0,0745	0,5935
1421822_at	NULL	NULL	-3,5014	0,0745	0,7525
1431795_a_at	Sema3b	sema domain, immunoglobulin domain (Ig), short basic domain, secreted, (semaphorin) 3B	-3,5031	0,0745	0,6715
1424502_at	Oit1	oncoprotein induced transcript 1	-3,5033	0,0745	0,7161
1422672_at	Sprr1b	small proline-rich protein 1B	-3,5034	0,0745	0,5829
1449646_s_at	Tigd5	tigger transposable element derived 5	-3,5039	0,0745	0,5325
1450692_at	Kif4	kinesin family member 4	-3,5041	0,0745	0,7392
1450579_x_at	Sry	sex determining region of Chr Y	-3,5048	0,0745	0,7680
1449532_at	Chrng	cholinergic receptor, nicotinic, gamma polypeptide	-3,5059	0,0745	0,7651
1425671_at	Homer1	homer homolog 1 (Drosophila)	-3,5067	0,0745	0,7255
1423853_at	6330527O06Rik	RIKEN cDNA 6330527O06 gene	-3,5082	0,0745	0,7512
1451400_at	Gemin8	gem (nuclear organelle) associated protein 8	-3,5083	0,0745	0,9054
1419503_at	Stc2	stanniocalcin 2	-3,5084	0,0745	0,7313
1424624_at	2900011O08Rik	RIKEN cDNA 2900011O08 gene	-3,5088	0,0745	0,7671
1437697_at	Rad23a	RAD23a homolog (S. cerevisiae)	-3,5090	0,0745	0,6963
1418572_x_at	Tnfrsf12a	tumor necrosis factor receptor superfamily, member 12a	-3,5100	0,0745	0,7182
1422214_at	Npffr2	neuropeptide FF receptor 2	-3,5101	0,0745	0,7737
1425437_a_at	Kcnk7	potassium channel, subfamily K, member 7	-3,5108	0,0745	0,6976
1451823_at	Clca4	chloride channel calcium activated 4	-3,5118	0,0745	0,9206
1419323_at	Padi1	peptidyl arginine deiminase, type I	-3,5124	0,0745	0,7222
1451843_a_at	Ggta1	glycoprotein galactosyltransferase alpha 1, 3	-3,5128	0,0745	0,6959
1420057_at	Jmjd6	jumonji domain containing 6	-3,5129	0,0745	0,7955
1451761_at	Hoxb4	homeobox B4	-3,5145	0,0745	0,7429
1421092_at	Serpina12	serine (or cysteine) peptidase inhibitor, clade A (alpha-1 antiproteinase, antitrypsin), member 12	-3,5155	0,0745	0,7550
1422938_at	Bcl2	B-cell leukemia/lymphoma 2	-3,5160	0,0745	0,6787
1424096_at	Krt5	keratin 5	-3,5168	0,0745	0,8086
1420089_at	Nfkbia	nuclear factor of kappa light polypeptide gene enhancer in B-cells inhibitor, alpha	-3,5170	0,0745	0,8113
1420710_at	Rel	reticuloendotheliosis oncogene	-3,5179	0,0745	0,7532
1422114_at	Erf	Ets2 repressor factor	-3,5188	0,0745	0,6562
1451933_a_at	Cts7	cathepsin 7	-3,5230	0,0745	0,8232
1417370_at	Tff3	trefoil factor 3, intestinal	-3,5234	0,0745	0,7522
1425880_x_at	Zfp352	zinc finger protein 352	-3,5242	0,0745	0,8981
1422304_at	Lcn4	lipocalin 4	-3,5245	0,0745	0,8254
1451584_at	Havcr2	hepatitis A virus cellular receptor 2	-3,5249	0,0745	0,8193
1418517_at	Irx3	Iroquois related homeobox 3 (Drosophila)	-3,5251	0,0745	0,8552
1422068_at	NULL	NULL	-3,5254	0,0745	0,5288
1432312_a_at	Prss41	protease, serine, 41	-3,5266	0,0745	0,6920
1448514_at	Cox5b	cytochrome c oxidase, subunit Vb	-3,5279	0,0745	0,8710
1456173_at	Il10ra	interleukin 10 receptor, alpha	-3,5286	0,0745	0,6811
1421151_a_at	Epha2	Eph receptor A2	-3,5291	0,0745	0,8402
1420050_at	NULL	NULL	-3,5330	0,0745	0,8208
1430634_a_at	Pfkp	phosphofructokinase, platelet	-3,5333	0,0745	0,6763
1427812_at	Ids	iduronate 2-sulfatase	-3,5339	0,0745	0,7784
1460309_at	Tal2	T-cell acute lymphocytic leukemia 2	-3,5340	0,0745	0,7606
1450516_a_at	Rab17	RAB17, member RAS oncogene family	-3,5361	0,0745	0,7703

Probeset ID	***Symbol***	***Gene Name***	***d-value***	***q-value***	***R-fold***
1420074_at	D8Ertd738e	DNA segment, Chr 8, ERATO Doi 738, expressed	-3,5381	0,0745	0,7564
1449834_at	Magix	MAGI family member, X-linked	-3,5408	0,0745	0,7957
1424351_at	Wfdc2	WAP four-disulfide core domain 2	-3,5415	0,0745	0,8160
1438302_at	NULL	NULL	-3,5415	0,0745	0,6424
1460202_at	Myoz1	myozenin 1	-3,5436	0,0745	0,6640
1452015_at	6330416G13Rik	RIKEN cDNA 6330416G13 gene	-3,5439	0,0745	0,8277
1450410_a_at	Slc48a1	solute carrier family 48 (heme transporter), member 1	-3,5440	0,0745	0,9165
1452610_at	Zkscan14	zinc finger with KRAB and SCAN domains 14	-3,5455	0,0744	0,7568
1426399_at	Vwa1	von Willebrand factor A domain containing 1	-3,5472	0,0743	0,7330
1427838_at	Tubb2a	tubulin, beta 2A	-3,5483	0,0743	0,8422
1422762_at	Kif17	kinesin family member 17	-3,5503	0,0742	0,6268
1450096_at	Gna12	guanine nucleotide binding protein, alpha 12	-3,5543	0,0741	0,8271
1421374_a_at	Fxyd1	FXYD domain-containing ion transport regulator 1	-3,5543	0,0741	0,7305
1460710_at	Adora2a	adenosine A2a receptor	-3,5550	0,0740	0,6988
1421614_at	Zan	zonadhesin	-3,5552	0,0740	0,6608
1423836_at	Zfp503	zinc finger protein 503	-3,5554	0,0740	0,6767
1418163_at	Tlr4	toll-like receptor 4	-3,5555	0,0740	0,7435
1418481_at	Pkmyt1	protein kinase, membrane associated tyrosine/threonine 1	-3,5574	0,0740	0,7734
1425026_at	Sft2d2	SFT2 domain containing 2	-3,5598	0,0740	0,7657
1419593_at	NULL	NULL	-3,5603	0,0740	0,8996
1427444_at	Abcf1	ATP-binding cassette, sub-family F (GCN20), member 1	-3,5609	0,0740	0,7581
1453086_at	6330408A02Rik	RIKEN cDNA 6330408A02 gene	-3,5610	0,0740	0,7813
1453574_at	Hba-a1	hemoglobin alpha, adult chain 1	-3,5614	0,0740	0,7768
1419410_at	Batf	basic leucine zipper transcription factor, ATF-like	-3,5629	0,0740	0,6888
1446790_at	C78948	expressed sequence C78948	-3,5631	0,0740	0,7848
1423344_at	Epor	erythropoietin receptor	-3,5632	0,0740	0,7517
1427790_at	Adam1a	a disintegrin and metallopeptidase domain 1a	-3,5639	0,0740	0,9022
1422029_at	Ccl20	chemokine (C-C motif) ligand 20	-3,5641	0,0740	0,6655
1432086_a_at	Ribc2	RIB43A domain with coiled-coils 2	-3,5662	0,0740	0,6469
1421679_a_at	Cdkn1a	cyclin-dependent kinase inhibitor 1A (P21)	-3,5701	0,0740	0,7426
1421159_at	Cgnl1	cingulin-like 1	-3,5702	0,0740	0,7478
1421762_at	Kcnj5	potassium inwardly-rectifying channel, subfamily J, member 5	-3,5708	0,0740	0,6722
1420801_at	Npas1	neuronal PAS domain protein 1	-3,5713	0,0740	0,7251
1449814_at	Frat1	frequently rearranged in advanced T-cell lymphomas	-3,5717	0,0740	0,5910
1448062_at	Stk11	serine/threonine kinase 11	-3,5722	0,0740	0,6256
1460255_at	Tnfsf13b	tumor necrosis factor (ligand) superfamily, member 13b	-3,5765	0,0740	0,8577
1449961_at	Rph3a	rabphilin 3A	-3,5766	0,0740	0,6401
1422825_at	Cartpt	CART prepropeptide	-3,5769	0,0740	0,7847
1419826_at	Kif17	kinesin family member 17	-3,5773	0,0740	0,7009
1422283_at	Cd40lg	CD40 ligand	-3,5798	0,0740	0,6729
1454770_at	Cckbr	cholecystokinin B receptor	-3,5827	0,0740	0,7015
1452303_at	Arhgef10	Rho guanine nucleotide exchange factor (GEF) 10	-3,5839	0,0740	0,8820
1417093_a_at	Gtf2h4	general transcription factor II H, polypeptide 4	-3,5844	0,0740	0,8936
1422934_x_at	Defa-rs7	defensin, alpha, related sequence 7	-3,5881	0,0740	0,4185
1456289_at	NULL	NULL	-3,5890	0,0740	0,7274
1451876_a_at	Trp63	transformation related protein 63	-3,5896	0,0740	0,7803
1449012_s_at	Fndc4	fibronectin type III domain containing 4	-3,5900	0,0740	0,8578
1423450_a_at	Hs3st1	heparan sulfate (glucosamine) 3-O-sulfotransferase 1	-3,5904	0,0740	0,7696
1428121_at	Fam125b	family with sequence similarity 125, member B	-3,5918	0,0740	0,7415
1450022_at	Gtpbp1	GTP binding protein 1	-3,5925	0,0740	0,7136
1416737_at	Gys1	glycogen synthase 1, muscle	-3,5945	0,0740	0,7075
1425798_a_at	Recql	RecQ protein-like	-3,5947	0,0740	0,7539
1425723_at	Nr1i2	nuclear receptor subfamily 1, group I, member 2	-3,5956	0,0740	0,8077
1450262_at	Clcf1	cardiotrophin-like cytokine factor 1	-3,5975	0,0740	0,7321
1432099_a_at	Prodh2	proline dehydrogenase (oxidase) 2	-3,5977	0,0740	0,7037
1417641_at	Galntl5	UDP-N-acetyl-alpha-D-galactosamine:polypeptide N-acetylgalactosaminyltransferase-like 5	-3,5990	0,0740	0,7365
1449395_at	Ankrd61	ankyrin repeat domain 61	-3,5995	0,0740	0,6924
1416378_at	Pnkp	polynucleotide kinase 3'- phosphatase	-3,6010	0,0740	0,7754
1427712_at	Ceacam1	carcinoembryonic antigen-related cell adhesion molecule 1	-3,6033	0,0740	0,8113
1418272_at	Oxct2a	3-oxoacid CoA transferase 2A	-3,6045	0,0740	0,7492
1448433_a_at	Pcolce	procollagen C-endopeptidase enhancer protein	-3,6058	0,0740	0,7360
1419448_at	Tbc1d1	TBC1 domain family, member 1	-3,6066	0,0740	0,7028
1452369_at	Magi1	membrane associated guanylate kinase, WW and PDZ domain containing 1	-3,6076	0,0740	0,6750
1449034_at	Klkb1	kallikrein B, plasma 1	-3,6078	0,0740	0,8254
1425447_at	Dkk4	dickkopf homolog 4 (Xenopus laevis)	-3,6079	0,0740	0,8399
1420371_at	Sntb2	syntrophin, basic 2	-3,6087	0,0740	0,6787
1422334_a_at	Sftpa1	surfactant associated protein A1	-3,6098	0,0740	0,7346
1421293_at	Hdgfl1	hepatoma derived growth factor-like 1	-3,6114	0,0740	0,7227
1422898_s_at	Slc22a12	solute carrier family 22 (organic anion/cation transporter), member 12	-3,6118	0,0740	0,7121
1422867_at	Gzmg	granzyme G	-3,6123	0,0740	0,7451
1420431_at	Rptn	repetin	-3,6125	0,0740	0,8771
1450615_at	NULL	NULL	-3,6145	0,0740	0,8155
1451898_a_at	Sema6c	sema domain, transmembrane domain (TM), and cytoplasmic domain, (semaphorin) 6C	-3,6152	0,0740	0,6232
1421486_at	Egr3	early growth response 3	-3,6206	0,0740	0,6693
1447653_x_at	NULL	NULL	-3,6219	0,0740	0,5785
1442941_at	C77027	expressed sequence C77027	-3,6231	0,0740	0,6283

Probeset ID	Symbol	Gene Name	d-value	q-value	R-fold
1418811_at	Barhl1	BarH-like 1 (Drosophila)	-3,6247	0,0740	0,8042
1424187_at	Ccdc80	coiled-coil domain containing 80	-3,6248	0,0740	0,5266
1422415_at	Ang2	angiogenin, ribonuclease A family, member 2	-3,6253	0,0740	0,6650
1420921_at	Cd151	CD151 antigen	-3,6261	0,0740	0,6358
1421406_at	Entpd7	ectonucleoside triphosphate diphosphohydrolase 7	-3,6273	0,0740	0,8302
1438261_at	Cited4	Cbp/p300-interacting transactivator, with Glu/Asp-rich carboxy-terminal domain, 4	-3,6305	0,0740	0,7200
1421281_at	Gabra1	gamma-aminobutyric acid (GABA) A receptor, subunit alpha 1	-3,6312	0,0740	0,8684
1420257_at	1700073E17Rik	ribosomal protein L7 pseudogene	-3,6312	0,0740	0,8134
1425122_at	Fam3b	family with sequence similarity 3, member B	-3,6326	0,0740	0,8127
1421181_at	NULL	NULL	-3,6330	0,0740	0,6303
1418922_at	Cadm3	cell adhesion molecule 3	-3,6365	0,0740	0,8389
1451749_at	Irak4	interleukin-1 receptor-associated kinase 4	-3,6373	0,0740	0,7838
1418077_at	Trim21	tripartite motif-containing 21	-3,6378	0,0740	0,8637
1420250_at	NULL	NULL	-3,6388	0,0740	0,8717
1425454_a_at	Il12a	interleukin 12a	-3,6389	0,0740	0,7956
1449713_at	Cbx7	chromobox homolog 7	-3,6395	0,0740	0,7449
1427701_a_at	Six4	sine oculis-related homeobox 4 homolog (Drosophila)	-3,6416	0,0740	0,6667
1420511_at	Prph2	peripherin 2	-3,6431	0,0740	0,8501
1420364_at	Gpr87	G protein-coupled receptor 87	-3,6435	0,0740	0,7892
1425298_a_at	Naip1	NLR family, apoptosis inhibitory protein 1	-3,6436	0,0740	0,8750
1424249_a_at	Arhgap9	Rho GTPase activating protein 9	-3,6459	0,0740	0,6325
1425353_at	Enpp3	ectonucleotide pyrophosphatase/phosphodiesterase 3	-3,6484	0,0740	0,7027
1450563_at	Mgst3	microsomal glutathione S-transferase 3	-3,6488	0,0740	0,7093
1450228_a_at	Pip5k1c	phosphatidylinositol-4-phosphate 5-kinase, type 1 gamma	-3,6510	0,0740	0,6348
1419882_at	NULL	NULL	-3,6519	0,0740	0,9078
1425947_at	Ifng	interferon gamma	-3,6551	0,0740	0,8476
1422188_s_at	NULL	NULL	-3,6595	0,0740	0,8714
1427750_at	NULL	NULL	-3,6599	0,0740	0,7702
1420106_at	Siah1a	seven in absentia 1A	-3,6604	0,0740	0,7435
1422151_at	Gatsl2	GATS protein-like 2	-3,6619	0,0740	0,8489
1425877_at	Hyal3	hyaluronoglucosaminidase 3	-3,6633	0,0740	0,7469
1421420_at	Ccr10	chemokine (C-C motif) receptor 10	-3,6640	0,0740	0,7290
1421781_at	Upk2	uroplakin 2	-3,6644	0,0740	0,7635
1426650_at	Myh8	myosin, heavy polypeptide 8, skeletal muscle, perinatal	-3,6649	0,0740	0,8461
1450567_a_at	Col2a1	collagen, type II, alpha 1	-3,6651	0,0740	0,6331
1448511_at	Ptprcap	protein tyrosine phosphatase, receptor type, C polypeptide-associated protein	-3,6655	0,0740	0,5436
1426606_at	Crtac1	cartilage acidic protein 1	-3,6661	0,0740	0,6566
1449836_x_at	Bik	BCL2-interacting killer	-3,6668	0,0740	0,7918
1428786_at	Nckap1l	NCK associated protein 1 like	-3,6683	0,0740	0,8655
1416606_s_at	Nhp2	NHP2 ribonucleoprotein homolog (yeast)	-3,6687	0,0740	0,6926
1460636_at	Map2k2	mitogen-activated protein kinase kinase 2	-3,6697	0,0740	0,6219
1420673_a_at	Acox2	acyl-Coenzyme A oxidase 2, branched chain	-3,6701	0,0740	0,7176
1419044_at	Cntnap4	contactin associated protein-like 4	-3,6711	0,0740	0,9016
1417863_at	Spata19	spermatogenesis associated 19	-3,6729	0,0740	0,8067
1418454_at	Mfap5	microfibrillar associated protein 5	-3,6765	0,0740	0,8185
1420448_at	Rhox2a	reproductive homeobox 2A	-3,6777	0,0740	0,5261
1417498_at	Serpinf2	serine (or cysteine) peptidase inhibitor, clade F, member 2	-3,6782	0,0740	0,7624
1450944_at	Cspg4	chondroitin sulfate proteoglycan 4	-3,6784	0,0740	0,7079
1427451_a_at	BC018473	cDNA sequence BC018473	-3,6804	0,0740	0,7353
1418617_x_at	Clgn	calmegin	-3,6837	0,0740	0,7000
1422343_at	Olfr155	olfactory receptor 155	-3,6839	0,0740	0,8909
1452553_at	NULL	NULL	-3,6840	0,0740	0,8653
1427421_at	NULL	NULL	-3,6847	0,0740	0,8096
1422983_at	Itgb6	integrin beta 6	-3,6859	0,0740	0,7522
1454908_x_at	Serf1	small EDRK-rich factor 1	-3,6869	0,0740	0,5698
1421300_at	Adarb2	adenosine deaminase, RNA-specific, B2	-3,6880	0,0740	0,7674
1415847_at	Ldhc	lactate dehydrogenase C	-3,6901	0,0740	0,7430
1438918_at	NULL	NULL	-3,6909	0,0740	0,5470
1418328_at	NULL	NULL	-3,6912	0,0740	0,6018
1449970_at	Capn12	calpain 12	-3,6912	0,0740	0,7515
1450294_a_at	Kcnj6	potassium inwardly-rectifying channel, subfamily J, member 6	-3,6914	0,0740	0,8370
1418696_at	Ttc36	tetratricopeptide repeat domain 36	-3,6922	0,0740	0,6810
1421440_at	Wnt8b	wingless related MMTV integration site 8b	-3,6951	0,0740	0,8331
1419268_at	Agr2	anterior gradient 2 (Xenopus laevis)	-3,6953	0,0740	0,7954
1425132_at	Neto1	neuropilin (NRP) and tolloid (TLL)-like 1	-3,6968	0,0740	0,7044
1434074_x_at	Arf4	ADP-ribosylation factor 4	-3,6978	0,0740	0,9075
1448941_at	B4galt2	UDP-Gal:betaGlcNAc beta 1,4- galactosyltransferase, polypeptide 2	-3,7010	0,0740	0,5598
1448390_a_at	Dhrs3	dehydrogenase/reductase (SDR family) member 3	-3,7024	0,0740	0,8653
1449625_at	NULL	NULL	-3,7026	0,0740	0,8180
1424037_at	Itpka	inositol 1,4,5-trisphosphate 3-kinase A	-3,7031	0,0740	0,8947
1421562_at	Cd209c	CD209c antigen	-3,7068	0,0740	0,6975
1424814_a_at	Bcl2l14	BCL2-like 14 (apoptosis facilitator)	-3,7112	0,0740	0,6551
1427034_at	Ace	angiotensin I converting enzyme (peptidyl-dipeptidase A) 1	-3,7114	0,0740	0,8408
1438152_at	NULL	NULL	-3,7116	0,0740	0,6644
1452399_at	Rgs6	regulator of G-protein signaling 6	-3,7126	0,0740	0,7763
1427853_a_at	Hspb1	heat shock protein 1	-3,7134	0,0740	0,7289
1454907_at	Serf1	small EDRK-rich factor 1	-3,7138	0,0740	0,6032

Probeset ID	***Symbol***	***Gene Name***	***d-value***	***q-value***	***R-fold***
1421787_at	NULL	NULL	-3,7143	0,0740	0,7476
1427209_at	Baz2a	bromodomain adjacent to zinc finger domain, 2A	-3,7144	0,0740	0,6980
1420515_a_at	Pglyrp2	peptidoglycan recognition protein 2	-3,7150	0,0740	0,7674
1421635_at	Cnnm4	cyclin M4	-3,7187	0,0740	0,4377
1421489_a_at	2010106E10Rik	RIKEN cDNA 2010106E10 gene	-3,7205	0,0740	0,9058
1420155_at	AA408954	expressed sequence AA408954	-3,7258	0,0739	0,8948
1439379_x_at	Prm1	protamine 1	-3,7285	0,0738	0,8927
1448072_at	2010010I01Rik	RIKEN cDNA 2010010I01 gene	-3,7289	0,0738	0,8903
1421039_at	Mip	major intrinsic protein of eye lens fiber	-3,7293	0,0738	0,6061
1418743_a_at	NULL	NULL	-3,7295	0,0738	0,6959
1421722_at	NULL	NULL	-3,7302	0,0738	0,9364
1427625_a_at	Herc2	hect (homologous to the E6-AP (UBE3A) carboxyl terminus) domain and RCC1 (CHC1)-like domain (RLD) 2	-3,7304	0,0738	0,7291
1422995_at	Bmf	BCL2 modifying factor	-3,7310	0,0738	0,7981
1454260_at	LOC100047923	hypothetical protein LOC100047923	-3,7335	0,0738	0,5607
1422841_at	Agpat6	1-acylglycerol-3-phosphate O-acyltransferase 6 (lysophosphatidic acid acyltransferase, zeta)	-3,7360	0,0738	0,7956
1448216_at	Syngr3	synaptogyrin 3	-3,7388	0,0738	0,5517
1449992_at	Prss29	protease, serine, 29	-3,7410	0,0737	0,7302
1425710_a_at	Homer1	homer homolog 1 (Drosophila)	-3,7429	0,0736	0,7999
1428058_at	Ahnak	AHNAK nucleoprotein (desmoyokin)	-3,7462	0,0733	0,6880
1451878_a_at	Jmy	junction-mediating and regulatory protein	-3,7470	0,0733	0,8388
1437582_at	Acss1	acyl-CoA synthetase short-chain family member 1	-3,7471	0,0733	0,9047
1436784_x_at	Sf3b4	splicing factor 3b, subunit 4	-3,7473	0,0733	0,5887
1449458_at	Foxi1	forkhead box I1	-3,7501	0,0732	0,8611
1425786_a_at	Hsf4	heat shock transcription factor 4	-3,7507	0,0732	0,7020
1424047_at	Dera	2-deoxyribose-5-phosphate aldolase homolog (C. elegans)	-3,7515	0,0732	0,8070
1449795_at	D2Ertd63e	DNA segment, Chr 2, ERATO Doi 63, expressed	-3,7534	0,0732	0,8494
1423527_at	4921510H08Rik	RIKEN cDNA 4921510H08 gene	-3,7573	0,0731	0,7749
1451579_at	BC024139	cDNA sequence BC024139	-3,7604	0,0731	0,6973
1415995_at	Casp6	caspase 6	-3,7620	0,0730	0,7741
1419695_at	St8sia1	ST8 alpha-N-acetyl-neuraminide alpha-2,8-sialyltransferase 1	-3,7708	0,0728	0,8423
1425423_at	Glis1	GLIS family zinc finger 1	-3,7712	0,0728	0,8124
1450462_at	Crhr2	corticotropin releasing hormone receptor 2	-3,7719	0,0728	0,8348
1436861_at	Il7	interleukin 7	-3,7722	0,0728	0,8575
1423551_at	Cdh13	cadherin 13	-3,7777	0,0727	0,6513
1425260_at	Alb	albumin	-3,7782	0,0727	0,7249
1449091_at	Cldn8	claudin 8	-3,7796	0,0727	0,9300
1425232_x_at	Arr3	arrestin 3, retinal	-3,7798	0,0727	0,8450
1449804_at	Pnmt	phenylethanolamine-N-methyltransferase	-3,7799	0,0727	0,6774
1420648_at	NULL	NULL	-3,7806	0,0727	0,7841
1415931_at	Igf2	insulin-like growth factor 2	-3,7817	0,0727	0,8300
1420687_at	4932438H23Rik	RIKEN cDNA 4932438H23 gene	-3,7820	0,0727	0,7700
1451830_a_at	Spnb2	spectrin beta 2	-3,7822	0,0727	0,8771
1420758_at	5830411J07Rik	RIKEN cDNA 5830411J07 gene	-3,7830	0,0727	0,8477
1427841_at	NULL	NULL	-3,7836	0,0727	0,7445
1453410_at	Angptl4	angiopoietin-like 4	-3,7864	0,0727	0,9065
1423578_at	Col11a2	collagen, type XI, alpha 2	-3,7866	0,0727	0,7091
1422655_at	Ptch2	patched homolog 2	-3,7955	0,0725	0,6856
1448946_at	Kif3c	kinesin family member 3C	-3,7973	0,0725	0,7500
1419934_at	NULL	NULL	-3,7979	0,0725	0,7805
1421783_a_at	Kcnip2	Kv channel-interacting protein 2	-3,8036	0,0725	0,7341
1422654_at	Sgca	sarcoglycan, alpha (dystrophin-associated glycoprotein)	-3,8039	0,0725	0,6636
1453777_a_at	Ndst3	N-deacetylase/N-sulfotransferase (heparan glucosaminyl) 3	-3,8056	0,0725	0,8754
1425904_at	Satb2	special AT-rich sequence binding protein 2	-3,8071	0,0725	0,8171
1418740_at	Spert	spermatid associated	-3,8074	0,0725	0,5888
1421687_at	Msmb	beta-microseminoprotein	-3,8079	0,0725	0,8425
1416935_at	Trpv2	transient receptor potential cation channel, subfamily V, member 2	-3,8083	0,0725	0,8003
1421464_at	Nkx3-2	NK3 homeobox 2	-3,8090	0,0725	0,7470
1420885_a_at	Sez6	seizure related gene 6	-3,8109	0,0725	0,6529
1426470_at	Tbp	TATA box binding protein	-3,8113	0,0725	0,8526
1451914_a_at	Add2	adducin 2 (beta)	-3,8116	0,0725	0,7415
1422338_at	Gpr63	G protein-coupled receptor 63	-3,8119	0,0725	0,7973
1420593_a_at	Tead3	TEA domain family member 3	-3,8123	0,0725	0,6518
1418378_at	Bpil1	bactericidal/permeability-increasing protein-like 1	-3,8126	0,0725	0,7539
1460514_s_at	Ascl2	achaete-scute complex homolog 2 (Drosophila)	-3,8145	0,0725	0,8241
1422348_at	NULL	NULL	-3,8165	0,0725	0,7137
1420586_at	Zfp493	zinc finger protein 493	-3,8169	0,0725	0,7940
1422722_at	1700001K19Rik	RIKEN cDNA 1700001K19 gene	-3,8182	0,0725	0,5322
1425677_a_at	Ank1	ankyrin 1, erythroid	-3,8191	0,0725	0,7984
1438286_at	Otud7a	OTU domain containing 7A	-3,8196	0,0725	0,7637
1433958_at	Cntrob	centrobin, centrosomal BRCA2 interacting protein	-3,8207	0,0725	0,7628
1429287_a_at	Prl	prolactin	-3,8226	0,0725	0,8633
1432490_a_at	Pde10a	phosphodiesterase 10A	-3,8260	0,0725	0,6445
1422536_at	Tnni3	troponin I, cardiac 3	-3,8273	0,0725	0,6606
1429168_at	Zbtb46	zinc finger and BTB domain containing 46	-3,8293	0,0725	0,8080
1449822_at	Atoh1	atonal homolog 1 (Drosophila)	-3,8323	0,0725	0,6923
1433949_x_at	Pla2g1b	phospholipase A2, group IB, pancreas	-3,8343	0,0725	0,8378
1426077_at	Moxd2	monooxygenase, DBH-like 2	-3,8384	0,0724	0,7070

Probeset ID	*Symbol*	*Gene Name*	*d-value*	*q-value*	*R-fold*
1419995_at	Anapc16	anaphase promoting complex subunit 16	-3,8393	0,0724	0,7677
1455828_at	Gprin1	G protein-regulated inducer of neurite outgrowth 1	-3,8400	0,0724	0,5693
1416969_at	Gtse1	G two S phase expressed protein 1	-3,8416	0,0724	0,6981
1422220_at	Pou1f1	POU domain, class 1, transcription factor 1	-3,8465	0,0724	0,7701
1431400_a_at	Gas7	growth arrest specific 7	-3,8475	0,0724	0,6161
1427818_at	NULL	NULL	-3,8503	0,0723	0,8881
1448327_at	Actn2	actinin alpha 2	-3,8516	0,0723	0,7072
1419644_at	Cstf2	cleavage stimulation factor, 3' pre-RNA subunit 2	-3,8545	0,0723	0,7337
1450198_at	Dusp13	dual specificity phosphatase 13	-3,8559	0,0723	0,6789
1425638_at	Adap2	ArfGAP with dual PH domains 2	-3,8563	0,0723	0,9004
1419527_at	Comp	cartilage oligomeric matrix protein	-3,8572	0,0723	0,6176
1421080_at	Nr4a3	nuclear receptor subfamily 4, group A, member 3	-3,8572	0,0723	0,5984
1451210_at	Ppap2c	phosphatidic acid phosphatase type 2C	-3,8574	0,0723	0,8164
1421158_at	Cgnl1	cingulin-like 1	-3,8575	0,0723	0,6920
1422300_at	Nog	noggin	-3,8613	0,0723	0,7247
1431668_at	Capzb	capping protein (actin filament) muscle Z-line, beta	-3,8626	0,0723	0,7544
1418747_at	Sfpi1	SFFV proviral integration 1	-3,8658	0,0723	0,7619
1418418_a_at	Prl8a6	prolactin family 8, subfamily a, member 6	-3,8663	0,0723	0,8277
1418075_at	St6galnac4	ST6 (alpha-N-acetyl-neuraminyl-2,3-beta-galactosyl-1,3)-N-acetylgalactosaminide alpha-2,6-sialyltransferase 4	-3,8669	0,0723	0,7206
1427103_at	Plekho2	pleckstrin homology domain containing, family O member 2	-3,8702	0,0723	0,7720
1426006_at	Kcnq2	potassium voltage-gated channel, subfamily Q, member 2	-3,8712	0,0723	0,6320
1419342_at	Tram2	translocating chain-associating membrane protein 2	-3,8725	0,0723	0,7824
1420435_at	1700011A15Rik	RIKEN cDNA 1700011A15 gene	-3,8726	0,0723	0,8213
1451306_at	Cdca7l	cell division cycle associated 7 like	-3,8741	0,0723	0,7804
1416224_at	Zbtb17	zinc finger and BTB domain containing 17	-3,8753	0,0723	0,7763
1422986_at	Esrrb	estrogen related receptor, beta	-3,8763	0,0723	0,7389
1422458_at	Tcl1	T-cell lymphoma breakpoint 1	-3,8783	0,0723	0,7234
1419066_at	Ier5l	immediate early response 5-like	-3,8803	0,0723	0,7279
1431749_a_at	Rasgrp1	RAS guanyl releasing protein 1	-3,8807	0,0723	0,7565
1431335_a_at	Wfdc1	WAP four-disulfide core domain 1	-3,8829	0,0723	0,7060
1424263_at	Aif1l	allograft inflammatory factor 1-like	-3,8834	0,0723	0,8177
1450490_at	Kcna7	potassium voltage-gated channel, shaker-related subfamily, member 7	-3,8858	0,0723	0,8089
1418805_at	Sct	secretin	-3,8858	0,0723	0,6632
1429212_a_at	Lrrc51	leucine rich repeat containing 51	-3,8862	0,0723	0,7284
1425047_a_at	4921517L17Rik	RIKEN cDNA 4921517L17 gene	-3,8862	0,0723	0,7155
1418867_at	Cyp24a1	cytochrome P450, family 24, subfamily a, polypeptide 1	-3,8872	0,0723	0,6311
1432331_a_at	Prrx2	paired related homeobox 2	-3,8875	0,0723	0,7486
1420566_at	Sphk1	sphingosine kinase 1	-3,8895	0,0723	0,7408
1419466_at	Nkd2	naked cuticle 2 homolog (Drosophila)	-3,8895	0,0723	0,7163
1418031_at	Myo9b	myosin IXb	-3,8911	0,0723	0,8698
1422060_at	NULL	NULL	-3,8915	0,0723	0,6819
1422207_at	Htr5a	5-hydroxytryptamine (serotonin) receptor 5A	-3,8918	0,0723	0,8390
1432322_at	Ost4	oligosaccharyltransferase 4 homolog (S. cerevisiae)	-3,8932	0,0723	0,7617
1427389_at	BC021785	cDNA sequence BC021785	-3,8936	0,0723	0,7566
1420001_at	D7Ertd1e	DNA segment, Chr 7, ERATO Doi 1, expressed	-3,8941	0,0723	0,6134
1419182_at	Svep1	sushi, von Willebrand factor type A, EGF and pentraxin domain containing 1	-3,8951	0,0723	0,6970
1452275_at	Zfand3	zinc finger, AN1-type domain 3	-3,8976	0,0723	0,7702
1417546_at	Il2rb	interleukin 2 receptor, beta chain	-3,9005	0,0723	0,8048
1451904_a_at	Adam33	a disintegrin and metallopeptidase domain 33	-3,9020	0,0723	0,6905
1423544_at	Ptpn5	protein tyrosine phosphatase, non-receptor type 5	-3,9034	0,0723	0,7896
1420380_at	Ccl2	chemokine (C-C motif) ligand 2	-3,9052	0,0723	0,6594
1456578_x_at	Lasp1	LIM and SH3 protein 1	-3,9058	0,0723	0,7142
1418069_at	Apoc2	apolipoprotein C-II	-3,9095	0,0723	0,6349
1450997_at	Stk17b	serine/threonine kinase 17b (apoptosis-inducing)	-3,9108	0,0723	0,9222
1427939_s_at	Mycbp	c-myc binding protein	-3,9136	0,0723	0,8246
1422865_at	Runx1	runt related transcription factor 1	-3,9156	0,0723	0,8111
1428988_at	Abcc3	ATP-binding cassette, sub-family C (CFTR/MRP), member 3	-3,9175	0,0723	0,7862
1418464_at	Matn4	matrilin 4	-3,9196	0,0723	0,7222
1451410_a_at	Crip3	cysteine-rich protein 3	-3,9210	0,0723	0,7797
1417709_at	Cyp46a1	cytochrome P450, family 46, subfamily a, polypeptide 1	-3,9217	0,0723	0,7419
1419167_at	Prap1	proline-rich acidic protein 1	-3,9219	0,0723	0,6712
1451320_at	Arhgap8	Rho GTPase activating protein 8	-3,9248	0,0723	0,5328
1436289_x_at	1600014C10Rik	RIKEN cDNA 1600014C10 gene	-3,9253	0,0723	0,8597
1448977_at	Tcfap2c	transcription factor AP-2, gamma	-3,9286	0,0723	0,8203
1427223_a_at	Acsm2	acyl-CoA synthetase medium-chain family member 2	-3,9303	0,0723	0,5638
1420780_at	Ascl3	achaete-scute complex homolog 3 (Drosophila)	-3,9308	0,0723	0,6751
1421773_at	Etv2	ets variant gene 2	-3,9310	0,0723	0,6692
1439332_at	Ddit4l	DNA-damage-inducible transcript 4-like	-3,9345	0,0723	0,8373
1427521_a_at	9930023K05Rik	RIKEN cDNA 9930023K05 gene	-3,9386	0,0723	0,6371
1421002_at	Angptl2	angiopoietin-like 2	-3,9426	0,0723	0,7321
1426331_a_at	Frmd4b	FERM domain containing 4B	-3,9430	0,0723	0,7154
1421060_at	Mllt1	myeloid/lymphoid or mixed-lineage leukemia (trithorax homolog, Drosophila); translocated to, 1	-3,9491	0,0723	0,8544
1421520_at	Jph1	junctophilin 1	-3,9529	0,0723	0,7616
1452400_a_at	Hoxa11as	HOXA11 antisense RNA (non-protein coding)	-3,9535	0,0723	0,7570
1422513_at	Ccnf	cyclin F	-3,9583	0,0723	0,5136
1432236_a_at	Suv39h1	suppressor of variegation 3-9 homolog 1 (Drosophila)	-3,9622	0,0723	0,7263

Probeset ID	Symbol	Gene Name	d-value	q-value	R-fold
1449891_a_at	Dcbld2	discoidin, CUB and LCCL domain containing 2	-3,9640	0,0723	0,7810
1431705_a_at	Mcoln2	mucolipin 2	-3,9647	0,0723	0,8306
1437614_x_at	Zdhhc14	zinc finger, DHHC domain containing 14	-3,9660	0,0723	0,7281
1421501_a_at	NULL	NULL	-3,9666	0,0723	0,5798
1421390_at	Slc12a1	solute carrier family 12, member 1	-3,9668	0,0723	0,8205
1456218_at	Snx22	sorting nexin 22	-3,9688	0,0723	0,6412
1425309_at	Catsper2	cation channel, sperm associated 2	-3,9691	0,0723	0,7679
1421465_at	Wnt2b	wingless related MMTV integration site 2b	-3,9713	0,0723	0,7158
1417291_at	Tnfrsf1a	tumor necrosis factor receptor superfamily, member 1a	-3,9717	0,0723	0,6397
1417958_at	Tspan1	tetraspanin 1	-3,9730	0,0723	0,8033
1428439_at	Nub1	negative regulator of ubiquitin-like proteins 1	-3,9745	0,0723	0,7607
1438840_x_at	Apoa1	apolipoprotein A-I	-3,9853	0,0723	0,6032
1450260_at	Grpr	gastrin releasing peptide receptor	-3,9860	0,0723	0,7850
1422700_at	Alox12	arachidonate 12-lipoxygenase	-3,9867	0,0723	0,8505
1418571_at	Tnfrsf12a	tumor necrosis factor receptor superfamily, member 12a	-3,9894	0,0723	0,6979
1419647_a_at	Ier3	immediate early response 3	-3,9912	0,0723	0,8651
1421554_at	Lmx1a	LIM homeobox transcription factor 1 alpha	-3,9924	0,0723	0,7731
1451719_at	Med17	mediator complex subunit 17	-3,9934	0,0723	0,8141
1419173_at	Acy1	aminoacylase 1	-3,9945	0,0723	0,6912
1419785_at	AA516738	expressed sequence AA516738	-3,9970	0,0723	0,7959
1422738_at	Ddr2	discoidin domain receptor family, member 2	-3,9984	0,0723	0,6408
1419999_at	Igbp1	immunoglobulin (CD79A) binding protein 1	-4,0002	0,0723	0,5625
1452456_at	Nrip2	nuclear receptor interacting protein 2	-4,0023	0,0723	0,6611
1427953_at	Fanci	Fanconi anemia, complementation group I	-4,0042	0,0723	0,8248
1452641_at	Yjefn3	YjeF N-terminal domain containing 3	-4,0072	0,0723	0,5602
1454018_at	Tlk2	tousled-like kinase 2 (Arabidopsis)	-4,0089	0,0723	0,9100
1424150_at	Gdpd5	glycerophosphodiester phosphodiesterase domain containing 5	-4,0105	0,0723	0,6007
1423007_a_at	Gfra2	glial cell line derived neurotrophic factor family receptor alpha 2	-4,0109	0,0723	0,5831
1426427_at	Ttll1	tubulin tyrosine ligase-like 1	-4,0114	0,0723	0,7813
1452202_at	Pde2a	phosphodiesterase 2A, cGMP-stimulated	-4,0121	0,0723	0,6937
1420407_at	Ltb4r1	leukotriene B4 receptor 1	-4,0128	0,0723	0,7661
1460744_at	NULL	NULL	-4,0137	0,0723	0,7515
1423556_at	Akr1b7	aldo-keto reductase family 1, member B7	-4,0163	0,0723	0,8350
1427669_a_at	Cit	citron	-4,0164	0,0723	0,7732
1418949_at	Gdf15	growth differentiation factor 15	-4,0191	0,0723	0,6232
1421626_at	Tmc1	transmembrane channel-like gene family 1	-4,0222	0,0723	0,9346
1436112_at	AI118078	expressed sequence AI118078	-4,0238	0,0723	0,7190
1420549_at	Gbp1	guanylate binding protein 1	-4,0247	0,0723	0,8796
1452442_at	Usp13	ubiquitin specific peptidase 13 (isopeptidase T-3)	-4,0268	0,0723	0,7804
1460358_s_at	Nudt22	nudix (nucleoside diphosphate linked moiety X)-type motif 22	-4,0269	0,0723	0,7738
1419570_at	Slc28a3	solute carrier family 28 (sodium-coupled nucleoside transporter), member 3	-4,0284	0,0723	0,7355
1430025_at	Ppp3cc	protein phosphatase 3, catalytic subunit, gamma isoform	-4,0291	0,0723	0,8536
1424879_at	NULL	NULL	-4,0301	0,0723	0,7651
1433844_a_at	Dusp9	dual specificity phosphatase 9	-4,0311	0,0723	0,6593
1417661_at	Rdm1	RAD52 motif 1	-4,0327	0,0723	0,6602
1450606_at	Pnmt	phenylethanolamine-N-methyltransferase	-4,0349	0,0723	0,7487
1452535_at	Igh-6	immunoglobulin heavy chain 6 (heavy chain of IgM)	-4,0349	0,0723	0,7006
1449592_at	Tcf15	transcription factor 15	-4,0354	0,0723	0,6671
1421353_at	Pde7b	phosphodiesterase 7B	-4,0355	0,0723	0,8292
1426107_at	Prdm9	PR domain containing 9	-4,0356	0,0723	0,6880
1460312_at	Olfr157	olfactory receptor 157	-4,0409	0,0723	0,8304
1457379_at	NULL	NULL	-4,0419	0,0723	0,7350
1450529_at	H2-M9	histocompatibility 2, M region locus 9	-4,0449	0,0723	0,7747
1416741_at	Col5a1	collagen, type V, alpha 1	-4,0462	0,0723	0,7273
1426800_at	Rab8b	RAB8B, member RAS oncogene family	-4,0488	0,0723	0,7491
1417416_at	Kcna1	potassium voltage-gated channel, shaker-related subfamily, member 1	-4,0491	0,0723	0,7201
1450530_at	B3galt1	UDP-Gal:betaGlcNAc beta 1,3-galactosyltransferase, polypeptide 1	-4,0495	0,0723	0,7767
1422175_at	Mmp1a	matrix metallopeptidase 1a (interstitial collagenase)	-4,0522	0,0723	0,9124
1425218_a_at	Scgb3a2	secretoglobin, family 3A, member 2	-4,0539	0,0723	0,8387
1418977_at	Sap30bp	SAP30 binding protein	-4,0542	0,0723	0,7427
1419305_a_at	Fbxo36	F-box protein 36	-4,0545	0,0723	0,6232
1452595_at	Adamts4	a disintegrin-like and metallopeptidase (reprolysin type) with thrombospondin type 1 motif, 4	-4,0563	0,0723	0,8448
1432295_a_at	2310035K24Rik	RIKEN cDNA 2310035K24 gene	-4,0620	0,0723	0,7300
1418266_at	NULL	NULL	-4,0620	0,0723	0,7275
1427749_at	Galnt3	UDP-N-acetyl-alpha-D-galactosamine:polypeptide N-acetylgalactosaminyltransferase 3	-4,0660	0,0723	0,7770
1427052_at	NULL	NULL	-4,0702	0,0723	0,5706
1420241_at	NULL	NULL	-4,0715	0,0723	0,6635
1421455_at	Sntb1	syntrophin, basic 1	-4,0771	0,0723	0,6098
1418931_at	Reg4	regenerating islet-derived family, member 4	-4,0785	0,0723	0,6294
1423453_at	Nol12	nucleolar protein 12	-4,0800	0,0723	0,8527
1449455_at	Hck	hemopoietic cell kinase	-4,0843	0,0723	0,7718
1455910_at	Klhdc3	kelch domain containing 3	-4,0853	0,0723	0,7087
1424445_at	Tm4sf5	transmembrane 4 superfamily member 5	-4,0855	0,0723	0,7426
1422093_at	Wnt3a	wingless-related MMTV integration site 3A	-4,0855	0,0723	0,6353
1425505_at	Mylk	myosin, light polypeptide kinase	-4,0857	0,0723	0,7009
1421078_at	Tcf23	transcription factor 23	-4,0886	0,0723	0,8657

Probeset ID	*Symbol*	*Gene Name*	*d-value*	*q-value*	*R-fold*
1450249_s_at	Kif5a	kinesin family member 5A	-4,0900	0,0723	0,6984
1427688_a_at	Ptprs	protein tyrosine phosphatase, receptor type, S	-4,0901	0,0723	0,6700
1416204_at	Gpd1	glycerol-3-phosphate dehydrogenase 1 (soluble)	-4,0919	0,0723	0,6122
1451696_at	Zfp64	zinc finger protein 64	-4,0927	0,0723	0,6090
1426792_s_at	Rusc2	RUN and SH3 domain containing 2	-4,0929	0,0723	0,8629
1451481_s_at	Osta	organic solute transporter alpha	-4,0936	0,0723	0,8752
1420625_at	Gtf3c6	general transcription factor IIIC, polypeptide 6, alpha	-4,0944	0,0723	0,8577
1422198_a_at	Shmt1	serine hydroxymethyltransferase 1 (soluble)	-4,0963	0,0723	0,6390
1430559_at	Nt5c3l	5'-nucleotidase, cytosolic III-like	-4,0966	0,0723	0,7943
1421951_at	Lhx1	LIM homeobox protein 1	-4,0971	0,0723	0,6999
1431362_a_at	Smoc2	SPARC related modular calcium binding 2	-4,0980	0,0723	0,8920
1422586_at	Ecel1	endothelin converting enzyme-like 1	-4,0988	0,0723	0,7420
1426594_at	Frmd4b	FERM domain containing 4B	-4,1003	0,0723	0,8191
1430483_a_at	Tmem79	transmembrane protein 79	-4,1010	0,0723	0,7057
1421518_at	Kcns1	K+ voltage-gated channel, subfamily S, 1	-4,1037	0,0723	0,6768
1416431_at	Tubb6	tubulin, beta 6	-4,1043	0,0723	0,7747
1451447_at	Cuedc1	CUE domain containing 1	-4,1060	0,0723	0,5267
1427734_a_at	Dscaml1	Down syndrome cell adhesion molecule-like 1	-4,1067	0,0723	0,6665
1424919_at	Erbb2	v-erb-b2 erythroblastic leukemia viral oncogene homolog 2, neuro/glioblastoma derived oncogene homolog (avian)	-4,1095	0,0723	0,5454
1448213_at	Anxa1	annexin A1	-4,1096	0,0723	0,8051
1437721_at	Coro1c	coronin, actin binding protein 1C	-4,1107	0,0723	0,7818
1419004_s_at	NULL	NULL	-4,1116	0,0723	0,7869
1420707_a_at	Traip	TRAF-interacting protein	-4,1133	0,0723	0,7780
1426653_at	NULL	NULL	-4,1182	0,0723	0,7545
1451699_at	B230354O11Rik	RIKEN cDNA B230354O11 gene	-4,1224	0,0723	0,8374
1420596_at	Cacng2	calcium channel, voltage-dependent, gamma subunit 2	-4,1244	0,0723	0,7245
1421331_at	Hs3st3b1	heparan sulfate (glucosamine) 3-O-sulfotransferase 3B1	-4,1254	0,0723	0,6565
1426103_a_at	Esr2	estrogen receptor 2 (beta)	-4,1258	0,0723	0,7660
1418897_at	F2	coagulation factor II	-4,1322	0,0723	0,7554
1417611_at	Tmem37	transmembrane protein 37	-4,1323	0,0723	0,6981
1417100_at	Cd320	CD320 antigen	-4,1334	0,0723	0,6287
1448512_at	Hils1	histone H1-like protein in spermatids 1	-4,1350	0,0723	0,6573
1416916_at	Elf3	E74-like factor 3	-4,1417	0,0723	0,6671
1420666_at	Doc2b	double C2, beta	-4,1430	0,0723	0,7966
1421419_at	Kcnk4	potassium channel, subfamily K, member 4	-4,1440	0,0723	0,8049
1422089_at	Ncr1	natural cytotoxicity triggering receptor 1	-4,1457	0,0723	0,7222
1418782_at	Rxrg	retinoid X receptor gamma	-4,1480	0,0723	0,7468
1421343_at	Bach2	BTB and CNC homology 2	-4,1492	0,0723	0,8138
1419632_at	Tecta	tectorin alpha	-4,1532	0,0723	0,7228
1428578_s_at	Ppfia4	protein tyrosine phosphatase, receptor type, f polypeptide (PTPRF), interacting protein (liprin), alpha 4	-4,1538	0,0723	0,7349
1421262_at	Lipg	lipase, endothelial	-4,1564	0,0723	0,6701
1431505_at	Ppih	peptidyl prolyl isomerase H	-4,1582	0,0723	0,7865
1452136_at	Slc5a9	solute carrier family 5 (sodium/glucose cotransporter), member 9	-4,1584	0,0723	0,6965
1448756_at	S100a9	S100 calcium binding protein A9 (calgranulin B)	-4,1619	0,0723	0,7830
1419890_at	NULL	NULL	-4,1620	0,0723	0,8519
1421551_s_at	Ifi202b	interferon activated gene 202B	-4,1663	0,0723	0,7747
1419847_at	NULL	NULL	-4,1682	0,0723	0,7645
1420785_at	Gab2	growth factor receptor bound protein 2-associated protein 2	-4,1695	0,0723	0,7150
1451160_s_at	Pvr	poliovirus receptor	-4,1699	0,0723	0,6175
1435950_at	Hr	hairless	-4,1729	0,0723	0,7316
1417571_at	Mpg	N-methylpurine-DNA glycosylase	-4,1741	0,0723	0,7147
1424295_at	Dppa3	developmental pluripotency-associated 3	-4,1788	0,0723	0,7135
1452486_a_at	Cryaa	crystallin, alpha A	-4,1790	0,0723	0,6968
1428216_s_at	Tomm7	translocase of outer mitochondrial membrane 7 homolog (yeast)	-4,1817	0,0723	0,8181
1448085_at	D5Ertd102e	DNA segment, Chr 5, ERATO Doi 102, expressed	-4,1847	0,0723	0,9252
1424558_a_at	Cabyr	calcium-binding tyrosine-(Y)-phosphorylation regulated (fibrousheathin 2)	-4,1857	0,0723	0,8498
1419221_a_at	Rgs14	regulator of G-protein signaling 14	-4,1915	0,0723	0,8194
1427240_at	Dock6	dedicator of cytokinesis 6	-4,1924	0,0723	0,6295
1448037_at	Zcchc6	zinc finger, CCHC domain containing 6	-4,1928	0,0723	0,8404
1422030_at	Atp6v0a4	ATPase, H+ transporting, lysosomal V0 subunit A4	-4,1931	0,0723	0,7021
1416159_at	Nr2f2	nuclear receptor subfamily 2, group F, member 2	-4,1986	0,0723	0,7198
1423379_at	Nfatc4	nuclear factor of activated T-cells, cytoplasmic, calcineurin-dependent 4	-4,2082	0,0723	0,7473
1449428_at	Cldn18	claudin 18	-4,2128	0,0723	0,7290
1450003_at	Adra2b	adrenergic receptor, alpha 2b	-4,2149	0,0723	0,7475
1426113_x_at	NULL	NULL	-4,2181	0,0723	0,7366
1424608_a_at	NULL	NULL	-4,2185	0,0723	0,7707
1417867_at	Cfd	complement factor D (adipsin)	-4,2188	0,0723	0,6134
1438620_x_at	Sfrp1	secreted frizzled-related protein 1	-4,2189	0,0723	0,6245
1420768_a_at	Dhx58	DEXH (Asp-Glu-X-His) box polypeptide 58	-4,2198	0,0723	0,8020
1418037_at	C4bp	complement component 4 binding protein	-4,2206	0,0723	0,7944
1421194_at	Itga4	integrin alpha 4	-4,2264	0,0723	0,7371
1449566_at	Nkx2-5	NK2 transcription factor related, locus 5 (Drosophila)	-4,2266	0,0723	0,5653
1421975_a_at	Add2	adducin 2 (beta)	-4,2332	0,0723	0,6222
1427420_at	Nkx6-2	NK6 homeobox 2	-4,2353	0,0723	0,8032
1451632_a_at	NULL	NULL	-4,2366	0,0723	0,8065
1450205_at	Fgf22	fibroblast growth factor 22	-4,2368	0,0723	0,5353

Probeset ID	***Symbol***	***Gene Name***	***d-value***	***q-value***	***R-fold***
1439054_at	Zfp36l2	zinc finger protein 36, C3H type-like 2	-4,2437	0,0723	0,7212
1420304_x_at	NULL	NULL	-4,2465	0,0723	0,6381
1449562_at	1700001G17Rik	RIKEN cDNA 1700001G17 gene	-4,2472	0,0723	0,6507
1417405_at	Stard3	START domain containing 3	-4,2483	0,0723	0,7479
1451916_s_at	Trim15	tripartite motif-containing 15	-4,2486	0,0723	0,7900
1417036_at	Sac3d1	SAC3 domain containing 1	-4,2549	0,0722	0,7500
1431828_a_at	Synj2	synaptojanin 2	-4,2622	0,0721	0,8070
1448296_x_at	Tuba3a	tubulin, alpha 3A	-4,2638	0,0721	0,8833
1425509_at	Mark1	MAP/microtubule affinity-regulating kinase 1	-4,2647	0,0721	0,5926
1449509_at	Serf1	small EDRK-rich factor 1	-4,2658	0,0721	0,6658
1434116_at	Cbx2	chromobox homolog 2 (Drosophila Pc class)	-4,2670	0,0721	0,8654
1433029_at	Kcnj9	potassium inwardly-rectifying channel, subfamily J, member 9	-4,2670	0,0721	0,7105
1427330_at	Cars	cysteinyl-tRNA synthetase	-4,2689	0,0720	0,6885
1451752_at	Foxk1	forkhead box K1	-4,2689	0,0720	0,7626
1456972_at	C80256	expressed sequence C80256	-4,2696	0,0720	0,9163
1422122_at	Fcer2a	Fc receptor, IgE, low affinity II, alpha polypeptide	-4,2712	0,0720	0,7087
1422423_at	Magea7-ps	melanoma antigen, family A, 7, pseudogene	-4,2719	0,0720	0,5565
1438812_x_at	Usp19	ubiquitin specific peptidase 19	-4,2775	0,0720	0,6358
1420027_at	C80171	expressed sequence C80171	-4,2794	0,0720	0,8823
1420602_a_at	Esx1	extraembryonic, spermatogenesis, homeobox 1	-4,2814	0,0720	0,7919
1422402_a_at	Asz1	ankyrin repeat, SAM and basic leucine zipper domain containing 1	-4,2888	0,0720	0,7131
1421212_at	Abcc6	ATP-binding cassette, sub-family C (CFTR/MRP), member 6	-4,3005	0,0716	0,8202
1418415_at	Hoxb5	homeobox B5	-4,3044	0,0715	0,8868
1416596_at	Slc44a4	solute carrier family 44, member 4	-4,3078	0,0715	0,8620
1449165_at	Capn5	calpain 5	-4,3080	0,0715	0,6863
1451944_a_at	Tnfsf11	tumor necrosis factor (ligand) superfamily, member 11	-4,3099	0,0715	0,7649
1423244_at	Cyp2c68	cytochrome P450, family 2, subfamily c, polypeptide 68	-4,3113	0,0715	0,7135
1421391_at	Vipr2	vasoactive intestinal peptide receptor 2	-4,3144	0,0715	0,7999
1450680_at	Rag1	recombination activating gene 1	-4,3152	0,0715	0,7919
1422941_at	Wnt16	wingless-related MMTV integration site 16	-4,3212	0,0715	0,6318
1449449_at	Ptges	prostaglandin E synthase	-4,3231	0,0715	0,7988
1418297_at	Dpysl4	dihydropyrimidinase-like 4	-4,3272	0,0715	0,7229
1424530_at	Sec14l2	SEC14-like 2 (S. cerevisiae)	-4,3330	0,0715	0,6685
1417281_a_at	Mmp23	matrix metallopeptidase 23	-4,3330	0,0715	0,8371
1417732_at	Anxa8	annexin A8	-4,3337	0,0715	0,6468
1423305_at	Extl1	exostoses (multiple)-like 1	-4,3396	0,0715	0,6982
1450501_at	Itga2	integrin alpha 2	-4,3456	0,0713	0,7931
1418296_at	Fxyd5	FXYD domain-containing ion transport regulator 5	-4,3512	0,0712	0,6965
1423003_at	Setd7	SET domain containing (lysine methyltransferase) 7	-4,3525	0,0712	0,7897
1438297_at	AA545190	EST AA545190	-4,3642	0,0708	0,9130
1425291_at	Foxj1	forkhead box J1	-4,3659	0,0707	0,7323
1450236_at	Foxo3	forkhead box O3	-4,3755	0,0704	0,8293
1441433_at	NULL	NULL	-4,3790	0,0703	0,7491
1418722_at	Ngp	neutrophilic granule protein	-4,3809	0,0703	0,7012
1427392_at	Dscaml1	Down syndrome cell adhesion molecule-like 1	-4,3844	0,0703	0,5731
1422566_at	Tcfeb	transcription factor EB	-4,3933	0,0703	0,6666
1426730_a_at	Prl2b1	prolactin family 2, subfamily b, member 1	-4,3962	0,0703	0,7700
1419015_at	Wisp2	WNT1 inducible signaling pathway protein 2	-4,3964	0,0703	0,6787
1420759_s_at	NULL	NULL	-4,3970	0,0703	0,8691
1436336_at	Ptrh1	peptidyl-tRNA hydrolase 1 homolog (S. cerevisiae)	-4,3998	0,0703	0,8067
1436030_at	Cachd1	cache domain containing 1	-4,4020	0,0703	0,7466
1419884_at	NULL	NULL	-4,4024	0,0703	0,6713
1421436_at	Grid2	glutamate receptor, ionotropic, delta 2	-4,4026	0,0703	0,8372
1420177_at	Igll1	immunoglobulin lambda-like polypeptide 1	-4,4047	0,0703	0,8298
1451966_at	Mrap	melanocortin 2 receptor accessory protein	-4,4059	0,0703	0,8153
1427780_at	Defb35	defensin beta 35	-4,4154	0,0703	0,8335
1426097_a_at	Ccdc106	coiled-coil domain containing 106	-4,4186	0,0703	0,7315
1421657_a_at	Sox17	SRY-box containing gene 17	-4,4292	0,0703	0,6059
1448813_at	Aadac	arylacetamide deacetylase (esterase)	-4,4318	0,0703	0,8052
1448663_s_at	Mvd	mevalonate (diphospho) decarboxylase	-4,4358	0,0703	0,6464
1416980_at	Mettl7b	methyltransferase like 7B	-4,4372	0,0703	0,6862
1455109_at	Tbl1xr1	transducin (beta)-like 1X-linked receptor 1	-4,4380	0,0703	0,8572
1425151_a_at	Noxo1	NADPH oxidase organizer 1	-4,4434	0,0703	0,7312
1429947_a_at	Zbp1	Z-DNA binding protein 1	-4,4467	0,0703	0,5255
1421488_at	Rabgap1l	RAB GTPase activating protein 1-like	-4,4473	0,0703	0,8172
1424317_at	Slc25a19	solute carrier family 25 (mitochondrial thiamine pyrophosphate carrier), member 19	-4,4511	0,0703	0,6934
1423387_at	Psmd9	proteasome (prosome, macropain) 26S subunit, non-ATPase, 9	-4,4630	0,0703	0,7100
1450792_at	Tyrobp	TYRO protein tyrosine kinase binding protein	-4,4635	0,0703	0,7912
1422117_s_at	Khdrbs2	KH domain containing, RNA binding, signal transduction associated 2	-4,4685	0,0703	0,7096
1447957_at	D7Ertd128e	DNA segment, Chr 7, ERATO Doi 128, expressed	-4,4794	0,0703	0,9030
1425796_a_at	Fgfr3	fibroblast growth factor receptor 3	-4,4847	0,0703	0,5664
1422305_at	Ifnb1	interferon beta 1, fibroblast	-4,4862	0,0703	0,7209
1456115_at	Phf3	PHD finger protein 3	-4,4864	0,0703	0,8612
1427045_at	Synpo	synaptopodin	-4,4893	0,0703	0,7711
1430778_a_at	Nubp1	nucleotide binding protein 1	-4,4928	0,0703	0,6150
1448926_at	Hoxa5	homeobox A5	-4,4928	0,0703	0,8455
1452406_x_at	Erdr1	erythroid differentiation regulator 1	-4,4933	0,0703	0,5035
1420356_at	NULL	NULL	-4,4999	0,0703	0,7101

Probeset ID	Symbol	Gene Name	d-value	q-value	R-fold
1422212_at	Foxh1	forkhead box H1	-4,5009	0,0703	0,5724
1449707_at	Nr5a2	nuclear receptor subfamily 5, group A, member 2	-4,5022	0,0703	0,8939
1432848_a_at	Frmd8	FERM domain containing 8	-4,5089	0,0703	0,7580
1420062_at	NULL	NULL	-4,5098	0,0702	0,7160
1449399_a_at	Il1b	interleukin 1 beta	-4,5130	0,0702	0,7073
1452367_at	Coro2a	coronin, actin binding protein 2A	-4,5134	0,0702	0,8113
1422291_at	Ccr8	chemokine (C-C motif) receptor 8	-4,5135	0,0702	0,7454
1422585_at	Odf1	outer dense fiber of sperm tails 1	-4,5141	0,0702	0,7797
1423139_at	Wdr4	WD repeat domain 4	-4,5152	0,0702	0,8151
1422847_a_at	Prkcd	protein kinase C, delta	-4,5172	0,0702	0,8079
1425128_at	B3gnt8	UDP-GlcNAc:betaGal beta-1,3-N-acetylglucosaminyltransferase 8	-4,5183	0,0702	0,7575
1451629_at	Lbh	limb-bud and heart	-4,5240	0,0702	0,7345
1423580_at	Ankrd36	ankyrin repeat domain 36	-4,5243	0,0702	0,7877
1455435_s_at	Chdh	choline dehydrogenase	-4,5282	0,0702	0,7720
1449925_at	Cxcr3	chemokine (C-X-C motif) receptor 3	-4,5291	0,0702	0,7271
1432862_at	Nkx2-4	NK2 transcription factor related, locus 4 (Drosophila)	-4,5297	0,0702	0,6507
1421615_at	Myo15	myosin XV	-4,5315	0,0702	0,7251
1419136_at	Akr1c18	aldo-keto reductase family 1, member C18	-4,5318	0,0702	0,6713
1418306_at	Crybb1	crystallin, beta B1	-4,5364	0,0702	0,6297
1419329_at	Sorbs3	sorbin and SH3 domain containing 3	-4,5396	0,0702	0,7247
1419715_at	1700029F12Rik	RIKEN cDNA 1700029F12 gene	-4,5466	0,0702	0,6896
1418613_at	Kcnj1	potassium inwardly-rectifying channel, subfamily J, member 1	-4,5480	0,0702	0,9196
1450574_at	Cyp11b2	cytochrome P450, family 11, subfamily b, polypeptide 2	-4,5558	0,0700	0,6574
1427848_at	NULL	NULL	-4,5619	0,0700	0,8096
1427977_x_at	Oog1	oogenesin 1	-4,5640	0,0700	0,8847
1425467_a_at	Plp1	proteolipid protein (myelin) 1	-4,5666	0,0700	0,8159
1426044_a_at	Prkcq	protein kinase C, theta	-4,5689	0,0700	0,8148
1431914_at	Pde3a	phosphodiesterase 3A, cGMP inhibited	-4,5740	0,0698	0,8257
1420854_at	Eln	elastin	-4,5776	0,0698	0,6102
1425062_at	Fcrl1	Fc receptor-like 1	-4,5885	0,0696	0,8205
1424479_at	Cst8	cystatin 8 (cystatin-related epididymal spermatogenic)	-4,5939	0,0696	0,8084
1417266_at	Ccl6	chemokine (C-C motif) ligand 6	-4,5953	0,0696	0,8320
1449073_at	Flnc	filamin C, gamma	-4,5974	0,0696	0,6444
1433775_at	C77080	expressed sequence C77080	-4,6072	0,0696	0,8010
1416876_at	Parvg	parvin, gamma	-4,6117	0,0696	0,6731
1428766_at	Rnmtl1	RNA methyltransferase like 1	-4,6126	0,0696	0,5999
1420103_at	NULL	NULL	-4,6177	0,0696	0,8140
1455466_at	Gpr133	G protein-coupled receptor 133	-4,6205	0,0696	0,8578
1419051_at	Ovol1	OVO homolog-like 1 (Drosophila)	-4,6209	0,0696	0,7348
1454903_at	Ngfr	nerve growth factor receptor (TNFR superfamily, member 16)	-4,6241	0,0696	0,7535
1451880_at	Gm9779	predicted gene 9779	-4,6306	0,0696	0,7586
1417553_at	Plac1	placental specific protein 1	-4,6309	0,0696	0,7810
1453748_a_at	Kif23	kinesin family member 23	-4,6355	0,0696	0,8249
1430487_at	Auh	AU RNA binding protein/enoyl-coenzyme A hydratase	-4,6401	0,0696	0,7487
1417130_s_at	Angptl4	angiopoietin-like 4	-4,6419	0,0696	0,6984
1448612_at	NULL	NULL	-4,6428	0,0696	0,7265
1450837_at	Prp2	proline rich protein 2	-4,6453	0,0696	0,7598
1445459_at	Sstr5	somatostatin receptor 5	-4,6456	0,0696	0,8091
1427931_s_at	Pdxk	pyridoxal (pyridoxine, vitamin B6) kinase	-4,6482	0,0696	0,8233
1422960_at	Srd5a2	steroid 5 alpha-reductase 2	-4,6504	0,0696	0,7622
1419311_at	Trim10	tripartite motif-containing 10	-4,6613	0,0696	0,6826
1420388_at	Prss12	protease, serine, 12 neurotrypsin (motopsin)	-4,6618	0,0696	0,6072
1422209_s_at	NULL	NULL	-4,6672	0,0696	0,6729
1419555_at	Elf5	E74-like factor 5	-4,6712	0,0696	0,8699
1449206_at	Sypl2	synaptophysin-like 2	-4,6730	0,0696	0,7272
1419790_at	LOC100048336	hypothetical protein LOC100048336	-4,6734	0,0696	0,8117
1423378_at	NULL	NULL	-4,6768	0,0696	0,7551
1424981_at	Nln	neurolysin (metallopeptidase M3 family)	-4,6790	0,0696	0,7990
1419433_at	Nthl1	nth (endonuclease III)-like 1 (E.coli)	-4,6902	0,0696	0,7762
1422936_at	NULL	NULL	-4,6943	0,0696	0,8741
1420529_at	Dpf1	D4, zinc and double PHD fingers family 1	-4,6982	0,0696	0,7030
1422282_at	Tacr1	tachykinin receptor 1	-4,7014	0,0696	0,7168
1454970_at	Bud31	BUD31 homolog (yeast)	-4,7072	0,0696	0,8674
1438933_x_at	Rasgrp2	RAS, guanyl releasing protein 2	-4,7075	0,0696	0,7411
1441182_at	Brwd3	bromodomain and WD repeat domain containing 3	-4,7181	0,0696	0,7827
1421807_at	Defb6	defensin beta 6	-4,7226	0,0696	0,7952
1417973_at	Itih1	inter-alpha trypsin inhibitor, heavy chain 1	-4,7316	0,0696	0,6636
1420008_s_at	Wwc1	WW, C2 and coiled-coil domain containing 1	-4,7333	0,0696	0,6772
1450220_a_at	Spdef	SAM pointed domain containing ets transcription factor	-4,7333	0,0696	0,6980
1451660_a_at	Hoxb6	homeobox B6	-4,7371	0,0696	0,8393
1451835_at	Sox21	SRY-box containing gene 21	-4,7382	0,0696	0,8185
1426697_a_at	Lrpap1	low density lipoprotein receptor-related protein associated protein 1	-4,7411	0,0696	0,5815
1425449_at	Ppap2a	phosphatidic acid phosphatase type 2A	-4,7436	0,0696	0,8041
1453528_at	Lta4h	leukotriene A4 hydrolase	-4,7488	0,0696	0,5931
1450951_at	Smc3	structural maintenace of chromosomes 3	-4,7494	0,0696	0,7934
1420598_x_at	Defa-rs2	defensin, alpha, related sequence 2	-4,7523	0,0696	0,6143
1451616_at	Psg-ps1	pregnancy specific glycoprotein pseudogene 1	-4,7537	0,0696	0,8110
1423135_at	Thy1	thymus cell antigen 1, theta	-4,7554	0,0696	0,8199
1427119_at	Spink4	serine peptidase inhibitor, Kazal type 4	-4,7797	0,0696	0,6607

Probeset ID	Symbol	Gene Name	d-value	q-value	R-fold
1433483_s_at	C86187	expressed sequence C86187	-4,7898	0,0696	0,7170
1454007_a_at	Zfp142	zinc finger protein 142	-4,7927	0,0696	0,7564
1421568_at	Kcna6	potassium voltage-gated channel, shaker-related, subfamily, member 6	-4,7952	0,0696	0,8413
1416798_a_at	Nme4	non-metastatic cells 4, protein expressed in	-4,8034	0,0696	0,7321
1421599_at	Hs6st3	heparan sulfate 6-O-sulfotransferase 3	-4,8040	0,0696	0,6093
1427401_at	Chrna5	cholinergic receptor, nicotinic, alpha polypeptide 5	-4,8062	0,0696	0,8605
1426508_at	Gfap	glial fibrillary acidic protein	-4,8088	0,0696	0,6698
1431761_at	Entpd4	ectonucleoside triphosphate diphosphohydrolase 4	-4,8172	0,0696	0,7006
1456100_at	BC089491	cDNA sequence BC089491	-4,8181	0,0696	0,8035
1455989_at	Gjc2	gap junction protein, gamma 2	-4,8198	0,0696	0,7350
1448816_at	Ptgis	prostaglandin I2 (prostacyclin) synthase	-4,8203	0,0696	0,5949
1422602_a_at	Wnt5b	wingless-related MMTV integration site 5B	-4,8220	0,0696	0,7966
1418940_at	Sult1b1	sulfotransferase family 1B, member 1	-4,8288	0,0696	0,8352
1417959_at	Pdlim7	PDZ and LIM domain 7	-4,8399	0,0696	0,6512
1425352_at	Rcor3	REST corepressor 3	-4,8442	0,0696	0,9129
1421303_at	Ikzf1	IKAROS family zinc finger 1	-4,8442	0,0696	0,6360
1422985_at	Fzd1	frizzled homolog 1 (Drosophila)	-4,8473	0,0696	0,7797
1429794_a_at	P2rx1	purinergic receptor P2X, ligand-gated ion channel, 1	-4,8550	0,0696	0,7087
1449204_at	Gjb5	gap junction protein, beta 5	-4,8604	0,0696	0,6756
1425794_at	Pola2	polymerase (DNA directed), alpha 2	-4,8606	0,0696	0,8179
1456182_x_at	Mela	melanoma antigen	-4,8717	0,0696	0,9035
1459211_at	Gli2	GLI-Kruppel family member GLI2	-4,8726	0,0696	0,7923
1438760_x_at	Adam15	a disintegrin and metallopeptidase domain 15 (metargidin)	-4,8761	0,0696	0,7129
1450511_at	Musk	muscle, skeletal, receptor tyrosine kinase	-4,8799	0,0696	0,8643
1460405_at	Arhgef10l	Rho guanine nucleotide exchange factor (GEF) 10-like	-4,8832	0,0696	0,6832
1456534_at	Man1a2	mannosidase, alpha, class 1A, member 2	-4,8958	0,0693	0,5745
1451895_a_at	Dhcr24	24-dehydrocholesterol reductase	-4,8990	0,0693	0,8213
1425385_a_at	Ighg	Immunoglobulin heavy chain (gamma polypeptide)	-4,9007	0,0693	0,6004
1421967_at	B4galt5	UDP-Gal:betaGlcNAc beta 1,4-galactosyltransferase, polypeptide 5	-4,9090	0,0692	0,7356
1422430_at	Fignl1	fidgetin-like 1	-4,9119	0,0692	0,8379
1425994_a_at	Asah2	N-acylsphingosine amidohydrolase 2	-4,9146	0,0692	0,7538
1438138_a_at	Pex6	peroxisomal biogenesis factor 6	-4,9174	0,0692	0,7911
1449781_at	AA517650	expressed sequence AA517650	-4,9187	0,0692	0,8416
1453914_at	NULL	NULL	-4,9220	0,0692	0,8914
1421541_a_at	Mef2b	myocyte enhancer factor 2B	-4,9252	0,0692	0,6838
1419487_at	Mybph	myosin binding protein H	-4,9353	0,0692	0,7905
1419908_at	Fcrla	Fc receptor-like A	-4,9398	0,0692	0,8438
1426223_at	Ttc39c	tetratricopeptide repeat domain 39C	-4,9421	0,0692	0,8254
1416961_at	Bub1b	budding uninhibited by benzimidazoles 1 homolog, beta (S. cerevisiae)	-4,9567	0,0692	0,6812
1455201_x_at	Apoa1	apolipoprotein A-I	-4,9806	0,0692	0,7221
1418356_at	Mpst	mercaptopyruvate sulfurtransferase	-4,9905	0,0692	0,6793
1422904_at	Fmo2	flavin containing monooxygenase 2	-4,9920	0,0692	0,5228
1417172_at	Ube2l6	ubiquitin-conjugating enzyme E2L 6	-5,0003	0,0692	0,7737
1427627_at	BC038268	cDNA sequence BC038268	-5,0213	0,0692	0,7225
1450823_at	Sebox	SEBOX homeobox	-5,0225	0,0692	0,7985
1417871_at	Hsd17b7	hydroxysteroid (17-beta) dehydrogenase 7	-5,0256	0,0692	0,8323
1448800_at	Rtn4ip1	reticulon 4 interacting protein 1	-5,0323	0,0692	0,7259
1426995_a_at	Gfer	growth factor, erv1 (S. cerevisiae)-like (augmenter of liver regeneration)	-5,0325	0,0692	0,7318
1425434_a_at	Msr1	macrophage scavenger receptor 1	-5,0385	0,0692	0,7401
1417899_at	Zp3r	zona pellucida 3 receptor	-5,0542	0,0692	0,8869
1427744_at	Ccnb3	cyclin B3	-5,0576	0,0692	0,8631
1427755_at	Mcpt-ps1	mast cell protease, pseudogene 1	-5,0626	0,0692	0,8393
1427962_at	Ccdc102a	coiled-coil domain containing 102A	-5,0727	0,0692	0,5729
1423027_at	Foxl1	forkhead box L1	-5,0731	0,0692	0,6869
1421619_at	Kcnh3	potassium voltage-gated channel, subfamily H (eag-related), member 3	-5,0759	0,0692	0,6479
1422846_at	Rbp2	retinol binding protein 2, cellular	-5,0896	0,0692	0,7175
1456618_at	Mark4	MAP/microtubule affinity-regulating kinase 4	-5,0911	0,0692	0,5418
1422834_at	Kcnd2	potassium voltage-gated channel, Shal-related family, member 2	-5,1009	0,0692	0,8021
1427480_at	Leap2	liver-expressed antimicrobial peptide 2	-5,1018	0,0692	0,7678
1425233_at	2210407C18Rik	RIKEN cDNA 2210407C18 gene	-5,1032	0,0692	0,6694
1451304_at	Tmem143	transmembrane protein 143	-5,1168	0,0692	0,7564
1422121_at	Oprd1	opioid receptor, delta 1	-5,1216	0,0692	0,7825
1422333_at	Cyp21a1	cytochrome P450, family 21, subfamily a, polypeptide 1	-5,1260	0,0692	0,6601
1450426_at	Chrna6	cholinergic receptor, nicotinic, alpha polypeptide 6	-5,1315	0,0692	0,6507
1460673_at	Fpgs	folylpolyglutamyl synthetase	-5,1329	0,0692	0,8007
1424136_a_at	NULL	NULL	-5,1403	0,0692	0,7835
1425791_at	Pon2	paraoxonase 2	-5,1404	0,0692	0,8534
1418761_at	Igf2bp1	insulin-like growth factor 2 mRNA binding protein 1	-5,1437	0,0692	0,7922
1424035_at	Rora	RAR-related orphan receptor alpha	-5,1498	0,0692	0,8955
1421352_at	Tlr6	toll-like receptor 6	-5,1535	0,0692	0,8644
1448010_at	A430104N18Rik	RIKEN cDNA A430104N18 gene	-5,1567	0,0692	0,7446
1426019_at	Plaa	phospholipase A2, activating protein	-5,1604	0,0692	0,7824
1425268_a_at	Pear1	platelet endothelial aggregation receptor 1	-5,1751	0,0692	0,7348
1450158_at	Rem1	rad and gem related GTP binding protein 1	-5,1766	0,0692	0,6748
1452642_at	NULL	NULL	-5,1770	0,0692	0,7038
1431701_a_at	Pdzk1	PDZ domain containing 1	-5,2003	0,0692	0,7988
1424991_s_at	NULL	NULL	-5,2085	0,0692	0,8048
1415959_at	Slc2a4	solute carrier family 2 (facilitated glucose transporter), member 4	-5,2146	0,0692	0,7211
1434616_at	Slc38a10	solute carrier family 38, member 10	-5,2173	0,0692	0,6067

Probeset ID	Symbol	Gene Name	d-value	q-value	R-fold
1424279_at	Fga	fibrinogen alpha chain	-5,2242	0,0692	0,8664
1450134_at	Loxl4	lysyl oxidase-like 4	-5,2246	0,0692	0,6156
1419613_at	Col7a1	collagen, type VII, alpha 1	-5,2521	0,0692	0,5863
1452142_at	Slc6a1	solute carrier family 6 (neurotransmitter transporter, GABA), member 1	-5,2816	0,0692	0,8959
1425799_at	Fmo4	flavin containing monooxygenase 4	-5,2862	0,0692	0,7671
1448457_at	Krt71	keratin 71	-5,2957	0,0692	0,6268
1417099_at	NULL	NULL	-5,3009	0,0692	0,7892
1419147_at	Rec8	REC8 homolog (yeast)	-5,3162	0,0692	0,7179
1417969_at	Fbxo31	F-box protein 31	-5,3164	0,0692	0,7675
1427513_at	BC024137	cDNA sequence BC024137	-5,3216	0,0692	0,7251
1422233_at	Elk4	ELK4, member of ETS oncogene family	-5,3439	0,0692	0,6597
1457475_at	C80993	expressed sequence C80993	-5,3620	0,0692	0,8394
1421774_at	Vax1	ventral anterior homeobox containing gene 1	-5,3771	0,0692	0,6394
1418600_at	Klf1	Kruppel-like factor 1 (erythroid)	-5,3826	0,0692	0,6804
1450683_at	Tagln3	transgelin 3	-5,3910	0,0692	0,6549
1418677_at	Actn3	actinin alpha 3	-5,3975	0,0692	0,5850
1422383_at	NULL	NULL	-5,4019	0,0692	0,6883
1428538_s_at	Rarres2	retinoic acid receptor responder (tazarotene induced) 2	-5,4059	0,0692	0,5876
1449872_at	Hspb3	heat shock protein 3	-5,4126	0,0692	0,7077
1425954_a_at	Apex2	apurinic/apyrimidinic endonuclease 2	-5,4157	0,0692	0,7701
1422152_at	Hmx1	H6 homeobox 1	-5,4164	0,0692	0,6330
1425041_at	Lhx3	LIM homeobox protein 3	-5,4423	0,0692	0,6692
1422159_at	Ppef2	protein phosphatase, EF hand calcium-binding domain 2	-5,4480	0,0691	0,6765
1449621_s_at	Thsd1	thrombospondin, type I, domain 1	-5,4535	0,0690	0,7883
1427561_a_at	Afm	afamin	-5,4671	0,0690	0,6685
1420346_at	Asb12	ankyrin repeat and SOCS box-containing 12	-5,4674	0,0690	0,8403
1421177_at	Thap2	THAP domain containing, apoptosis associated protein 2	-5,4715	0,0690	0,8306
1427676_a_at	Grik1	glutamate receptor, ionotropic, kainate 1	-5,4834	0,0690	0,8530
1440865_at	Ifitm6	interferon induced transmembrane protein 6	-5,5033	0,0690	0,8371
1422183_a_at	Adra1b	adrenergic receptor, alpha 1b	-5,5173	0,0690	0,5968
1460433_at	Entpd6	ectonucleoside triphosphate diphosphohydrolase 6	-5,5279	0,0690	0,7636
1424340_at	Lrrc48	leucine rich repeat containing 48	-5,5302	0,0690	0,6104
1420697_at	Slc15a3	solute carrier family 15, member 3	-5,5492	0,0690	0,7929
1421069_at	Phf2	PHD finger protein 2	-5,5551	0,0690	0,6887
1427257_at	Vcan	versican	-5,5630	0,0690	0,7376
1433809_at	Ddx5	DEAD (Asp-Glu-Ala-Asp) box polypeptide 5	-5,5667	0,0690	0,5184
1419726_at	Adam18	a disintegrin and metallopeptidase domain 18	-5,5685	0,0690	0,6425
1416887_at	C1d	C1D nuclear receptor co-repressor	-5,5830	0,0690	0,7871
1433140_a_at	NULL	NULL	-5,5903	0,0690	0,7436
1418021_at	C4b	complement component 4B (Childo blood group)	-5,5930	0,0690	0,6128
1431857_at	Phf19	PHD finger protein 19	-5,5985	0,0687	0,6559
1422929_s_at	Atoh7	atonal homolog 7 (Drosophila)	-5,6136	0,0687	0,8100
1430005_a_at	Batf2	basic leucine zipper transcription factor, ATF-like 2	-5,6315	0,0683	0,7319
1422253_at	Col10a1	collagen, type X, alpha 1	-5,6370	0,0683	0,8209
1456066_a_at	Polr1a	polymerase (RNA) I polypeptide A	-5,6381	0,0683	0,7910
1422975_at	Mme	membrane metallo endopeptidase	-5,6461	0,0683	0,8018
1417391_a_at	Il16	interleukin 16	-5,6638	0,0683	0,7600
1450929_at	Zfp57	zinc finger protein 57	-5,7127	0,0683	0,6525
1417876_at	Fcgr1	Fc receptor, IgG, high affinity I	-5,7273	0,0681	0,7460
1418647_at	Gnl1	guanine nucleotide binding protein-like 1	-5,7370	0,0681	0,8524
1448792_a_at	Cyp2f2	cytochrome P450, family 2, subfamily f, polypeptide 2	-5,7398	0,0681	0,8568
1418269_at	Loxl3	lysyl oxidase-like 3	-5,7557	0,0681	0,6641
1450282_at	Fgf4	fibroblast growth factor 4	-5,7769	0,0681	0,6116
1432492_a_at	Haao	3-hydroxyanthranilate 3,4-dioxygenase	-5,7935	0,0681	0,5726
1417369_at	Hsd17b4	hydroxysteroid (17-beta) dehydrogenase 4	-5,8600	0,0670	0,7017
1434212_at	Ndufs8	NADH dehydrogenase (ubiquinone) Fe-S protein 8	-5,8652	0,0670	0,7674
1428022_at	Lcn13	lipocalin 13	-5,8810	0,0670	0,6632
1454974_at	Ntn1	netrin 1	-5,9119	0,0666	0,7396
1460233_at	Guca2b	guanylate cyclase activator 2b (retina)	-5,9160	0,0666	0,6939
1426073_at	Twsg1	twisted gastrulation homolog 1 (Drosophila)	-5,9193	0,0666	0,8467
1417545_at	Trpv4	transient receptor potential cation channel, subfamily V, member 4	-5,9231	0,0666	0,6167
1448727_at	Tle6	transducin-like enhancer of split 6, homolog of Drosophila E(spl)	-5,9443	0,0661	0,6078
1424985_a_at	Sox10	SRY-box containing gene 10	-5,9550	0,0658	0,8162
1455776_x_at	Bola2	bolA-like 2 (E. coli)	-5,9770	0,0656	0,6706
1422006_at	Eif2ak2	eukaryotic translation initiation factor 2-alpha kinase 2	-5,9901	0,0656	0,8010
1450570_a_at	Cd19	CD19 antigen	-5,9975	0,0656	0,6490
1418521_a_at	Mtx1	metaxin 1	-6,0200	0,0655	0,8071
1431336_at	Scara5	scavenger receptor class A, member 5 (putative)	-6,0428	0,0654	0,8062
1421589_at	Krt31	keratin 31	-6,0484	0,0654	0,7043
1422314_at	Clcn6	chloride channel 6	-6,0711	0,0652	0,7572
1425584_x_at	NULL	NULL	-6,0731	0,0652	0,7311
1451771_at	Tpcn1	two pore channel 1	-6,0917	0,0652	0,8774
1453517_at	Metapl1	methionine aminopeptidase-like 1	-6,1002	0,0652	0,8181
1448016_at	Sass6	spindle assembly 6 homolog (C. elegans)	-6,1096	0,0652	0,8265
1452117_a_at	Fyb	FYN binding protein	-6,1171	0,0652	0,6154
1419857_at	NULL	NULL	-6,1315	0,0652	0,8953
1420347_at	Plunc	palate, lung, and nasal epithelium associated	-6,1487	0,0650	0,8243
1425800_at	Rad9b	RAD9 homolog B (S. cerevisiae)	-6,1654	0,0648	0,8249
1419318_at	Saa4	serum amyloid A 4	-6,1749	0,0645	0,8364

Probeset ID	*Symbol*	*Gene Name*	*d-value*	*q-value*	*R-fold*
1420470_at	Sult1c1	sulfotransferase family, cytosolic, 1C, member 1	-6,1767	0,0645	0,7622
1448982_at	Klk6	kallikrein related-peptidase 6	-6,1805	0,0645	0,6574
1420378_at	Sftpd	surfactant associated protein D	-6,1821	0,0645	0,7077
1459934_at	Usp42	ubiquitin specific peptidase 42	-6,1976	0,0645	0,8539
1455992_at	Vgll4	vestigial like 4 (Drosophila)	-6,2635	0,0625	0,7138
1425381_a_at	Trfr2	transferrin receptor 2	-6,2718	0,0625	0,6150
1420891_at	Wnt7b	wingless-related MMTV integration site 7B	-6,2792	0,0625	0,6699
1429531_at	Smpd4	sphingomyelin phosphodiesterase 4	-6,2847	0,0625	0,6847
1419378_a_at	Fxyd2	FXYD domain-containing ion transport regulator 2	-6,2852	0,0625	0,8431
1417714_x_at	NULL	NULL	-6,2974	0,0625	0,5289
1421710_at	Zfp92	zinc finger protein 92	-6,3004	0,0625	0,6419
1455106_a_at	Ckb	creatine kinase, brain	-6,3157	0,0625	0,4588
1449367_at	Trex2	three prime repair exonuclease 2	-6,3159	0,0625	0,6848
1419827_s_at	Kif17	kinesin family member 17	-6,3178	0,0625	0,6820
1426139_a_at	Ccrl1	chemokine (C-C motif) receptor-like 1	-6,3319	0,0625	0,6495
1427986_a_at	Col16a1	collagen, type XVI, alpha 1	-6,3320	0,0625	0,5671
1434092_at	Atg9b	ATG9 autophagy related 9 homolog B (S. cerevisiae)	-6,3349	0,0625	0,6306
1419204_at	Dll1	delta-like 1 (Drosophila)	-6,3705	0,0625	0,7042
1421211_a_at	Ciita	class II transactivator	-6,3717	0,0625	0,6627
1451661_at	Akap4	A kinase (PRKA) anchor protein 4	-6,3774	0,0625	0,5571
1424831_at	Cpne2	copine II	-6,3921	0,0625	0,6941
1420437_at	Ido1	indoleamine 2,3-dioxygenase 1	-6,4007	0,0625	0,7954
1449423_at	Mast1	microtubule associated serine/threonine kinase 1	-6,4087	0,0625	0,6215
1420991_at	Ankrd1	ankyrin repeat domain 1 (cardiac muscle)	-6,4369	0,0620	0,8000
1449223_at	Dnajb8	DnaJ (Hsp40) homolog, subfamily B, member 8	-6,4373	0,0620	0,7778
1421600_a_at	Trim26	tripartite motif-containing 26	-6,4797	0,0617	0,7655
1422416_s_at	NULL	NULL	-6,5061	0,0616	0,7266
1416825_at	Snta1	syntrophin, acidic 1	-6,5264	0,0610	0,6493
1456608_at	Sac3d1	SAC3 domain containing 1	-6,5577	0,0610	0,6005
1439483_at	AI506816	expressed sequence AI506816	-6,5816	0,0610	0,7711
1422109_at	Rfx1	regulatory factor X, 1 (influences HLA class II expression)	-6,5974	0,0610	0,8181
1419541_at	4930403L05Rik	protein phosphatase 1 regulatory subunit 2 pseudogene	-6,6344	0,0610	0,7534
1418559_at	Prss39	protease, serine, 39	-6,6573	0,0610	0,8189
1450962_at	Pdha2	pyruvate dehydrogenase E1 alpha 2	-6,6647	0,0610	0,7722
1420770_at	Klk1b24	kallikrein 1-related peptidase b24	-6,6660	0,0610	0,6502
1449476_at	Stk30	serine/threonine kinase 30	-6,6663	0,0610	0,6805
1422108_at	Ppp1r3a	protein phosphatase 1, regulatory (inhibitor) subunit 3A	-6,6897	0,0602	0,7112
1418863_at	Gata4	GATA binding protein 4	-6,6906	0,0602	0,6994
1423125_at	Dclk1	doublecortin-like kinase 1	-6,6987	0,0602	0,5462
1431917_at	Pigq	phosphatidylinositol glycan anchor biosynthesis, class Q	-6,7037	0,0602	0,6756
1436058_at	Rsad2	radical S-adenosyl methionine domain containing 2	-6,7121	0,0602	0,7146
1450505_a_at	Fam134b	family with sequence similarity 134, member B	-6,7925	0,0602	0,6698
1426014_a_at	Cdhr5	cadherin-related family member 5	-6,7925	0,0602	0,7187
1422098_at	Acvr1b	activin A receptor, type 1B	-6,8148	0,0602	0,7647
1452309_at	Cgnl1	cingulin-like 1	-6,8607	0,0602	0,6618
1448156_at	Tff1	trefoil factor 1	-6,8750	0,0602	0,6861
1425971_at	NULL	NULL	-6,8974	0,0602	0,8035
1455934_at	Ndufb9	NADH dehydrogenase (ubiquinone) 1 beta subcomplex, 9	-6,9091	0,0602	0,8859
1453943_a_at	Acpp	acid phosphatase, prostate	-6,9880	0,0602	0,7970
1449241_at	Klhl1	kelch-like 1 (Drosophila)	-7,1508	0,0600	0,6830
1431386_s_at	Mbtps1	membrane-bound transcription factor peptidase, site 1	-7,1639	0,0600	0,7600
1417942_at	Lypd3	Ly6/Plaur domain containing 3	-7,1640	0,0600	0,7553
1418770_at	Cd2	CD2 antigen	-7,1702	0,0600	0,6701
1449535_at	Znrf4	zinc and ring finger 4	-7,1705	0,0600	0,5977
1431693_a_at	Il17b	interleukin 17B	-7,2049	0,0600	0,7494
1419430_at	Cyp26a1	cytochrome P450, family 26, subfamily a, polypeptide 1	-7,2639	0,0600	0,5593
1421247_at	Pax9	paired box gene 9	-7,2742	0,0600	0,6593
1427458_at	Bmp1	bone morphogenetic protein 1	-7,3997	0,0594	0,8504
1427228_at	Palld	palladin, cytoskeletal associated protein	-7,4150	0,0594	0,7197
1418237_s_at	Col18a1	collagen, type XVIII, alpha 1	-7,4305	0,0594	0,7865
1436670_x_at	1700019G17Rik	RIKEN cDNA 1700019G17 gene	-7,4473	0,0594	0,8034
1419289_a_at	Syngr1	synaptogyrin 1	-7,5802	0,0593	0,5761
1417805_at	Xpnpep2	X-prolyl aminopeptidase (aminopeptidase P) 2, membrane-bound	-7,6228	0,0581	0,7540
1454312_at	Gm9684	predicted gene 9684	-7,7192	0,0581	0,7491
1425609_at	Ncf1	neutrophil cytosolic factor 1	-7,7410	0,0581	0,7786
1425117_at	Aspdh	aspartate dehydrogenase domain containing	-7,7581	0,0581	0,6249
1416511_a_at	Cdc42ep4	CDC42 effector protein (Rho GTPase binding) 4	-7,8602	0,0581	0,7629
1451515_s_at	Glyat	glycine-N-acyltransferase	-7,8965	0,0581	0,8691
1421484_at	NULL	NULL	-8,0759	0,0576	0,8316
1427100_at	Metrn	meteorin, glial cell differentiation regulator	-8,1182	0,0576	0,7386
1427925_at	Stx17	syntaxin 17	-8,1234	0,0576	0,7684
1423323_at	Tacstd2	tumor-associated calcium signal transducer 2	-8,1261	0,0576	0,8537
1421248_at	Syn3	synapsin III	-8,1392	0,0576	0,5963
1452119_at	Rrp1b	ribosomal RNA processing 1 homolog B (S. cerevisiae)	-8,1456	0,0576	0,7156
1435301_at	1110004E09Rik	RIKEN cDNA 1110004E09 gene	-8,2227	0,0576	0,6436
1424838_at	A330049M08Rik	RIKEN cDNA A330049M08 gene	-8,2559	0,0576	0,7039
1416368_at	Gsta4	glutathione S-transferase, alpha 4	-8,4757	0,0557	0,7884
1432227_at	NULL	NULL	-8,4802	0,0557	0,8786
1423854_a_at	Rasl11b	RAS-like, family 11, member B	-8,5177	0,0557	0,7925

Probeset ID	*Symbol*	*Gene Name*	*d-value*	*q-value*	*R-fold*
1450775_at	Mos	Moloney sarcoma oncogene	-8,5547	0,0557	0,7878
1420298_at	NULL	NULL	-8,5860	0,0557	0,8648
1425727_at	Cldn19	claudin 19	-8,5877	0,0557	0,6805
1460420_a_at	Egfr	epidermal growth factor receptor	-8,5890	0,0557	0,5936
1423415_at	Gpr83	G protein-coupled receptor 83	-8,6357	0,0557	0,7001
1452835_a_at	Polrmt	polymerase (RNA) mitochondrial (DNA directed)	-8,7305	0,0557	0,8307
1438332_at	Slc22a6	solute carrier family 22 (organic anion transporter), member 6	-8,8389	0,0557	0,8886
1451993_at	9130404D08Rik	RIKEN cDNA 9130404D08 gene	-8,8818	0,0557	0,7050
1418027_at	Exo1	exonuclease 1	-9,0391	0,0557	0,8051
1435477_s_at	Fcgr2b	Fc receptor, IgG, low affinity IIb	-9,1945	0,0557	0,8062
1422137_at	Duoxa2	dual oxidase maturation factor 2	-9,2194	0,0557	0,8307
1426809_at	Tprn	taperin	-9,4286	0,0557	0,7049
1426150_at	Gipc3	GIPC PDZ domain containing family, member 3	-9,4522	0,0557	0,6754
1416464_at	Slc4a1	solute carrier family 4 (anion exchanger), member 1	-9,4561	0,0557	0,8041
1431363_at	Nhedc1	Na+/H+ exchanger domain containing 1	-9,7876	0,0557	0,6772
1450518_at	Hnf4g	hepatocyte nuclear factor 4, gamma	-9,7927	0,0557	0,8250
1438184_a_at	Ankrd5	ankyrin repeat domain 5	-9,9710	0,0557	0,8378
1420883_at	Sln	sarcolipin	-10,1186	0,0557	0,7896
1449805_at	Prss29	protease, serine, 29	-10,1919	0,0557	0,6776
1420933_a_at	Eya3	eyes absent 3 homolog (Drosophila)	-10,3403	0,0521	0,7085
1452636_x_at	Gtpbp5	GTP binding protein 5	-10,7144	0,0521	0,7502
1451694_at	Ttll3	tubulin tyrosine ligase-like family, member 3	-10,8054	0,0521	0,7637
1418383_at	Apcdd1	adenomatosis polyposis coli down-regulated 1	-10,8524	0,0521	0,8501
1421627_at	Evx1	even skipped homeotic gene 1 homolog	-11,7003	0,0521	0,8398
1421202_at	Chrna4	cholinergic receptor, nicotinic, alpha polypeptide 4	-11,7877	0,0521	0,7791
1449475_at	Atp12a	ATPase, H+/K+ transporting, nongastric, alpha polypeptide	-14,0343	0,0521	0,7883
1416752_at	Ldb3	LIM domain binding 3	-14,6119	0,0521	0,7488
1422278_at	Drd3	dopamine receptor D3	-14,6564	0,0521	0,7515
1449526_a_at	Gdpd3	glycerophosphodiester phosphodiesterase domain containing 3	-15,5773	0,0521	0,2048
1418878_at	Acrv1	acrosomal vesicle protein 1	-16,1620	0,0521	0,8669
1423509_a_at	Iapp	islet amyloid polypeptide	-16,1798	0,0521	0,2133
