# Supplementary figures and images for: Transcriptional profiling reveals functional links between RasGrf1 and Pttg1 in pancreatic beta cells
Source: BMC Genomics. 2014 Nov 25;15:1019. doi: 10.1186/1471-2164-15-1019 (PMC4301450; doi:10.1186/1471-2164-15-1019)

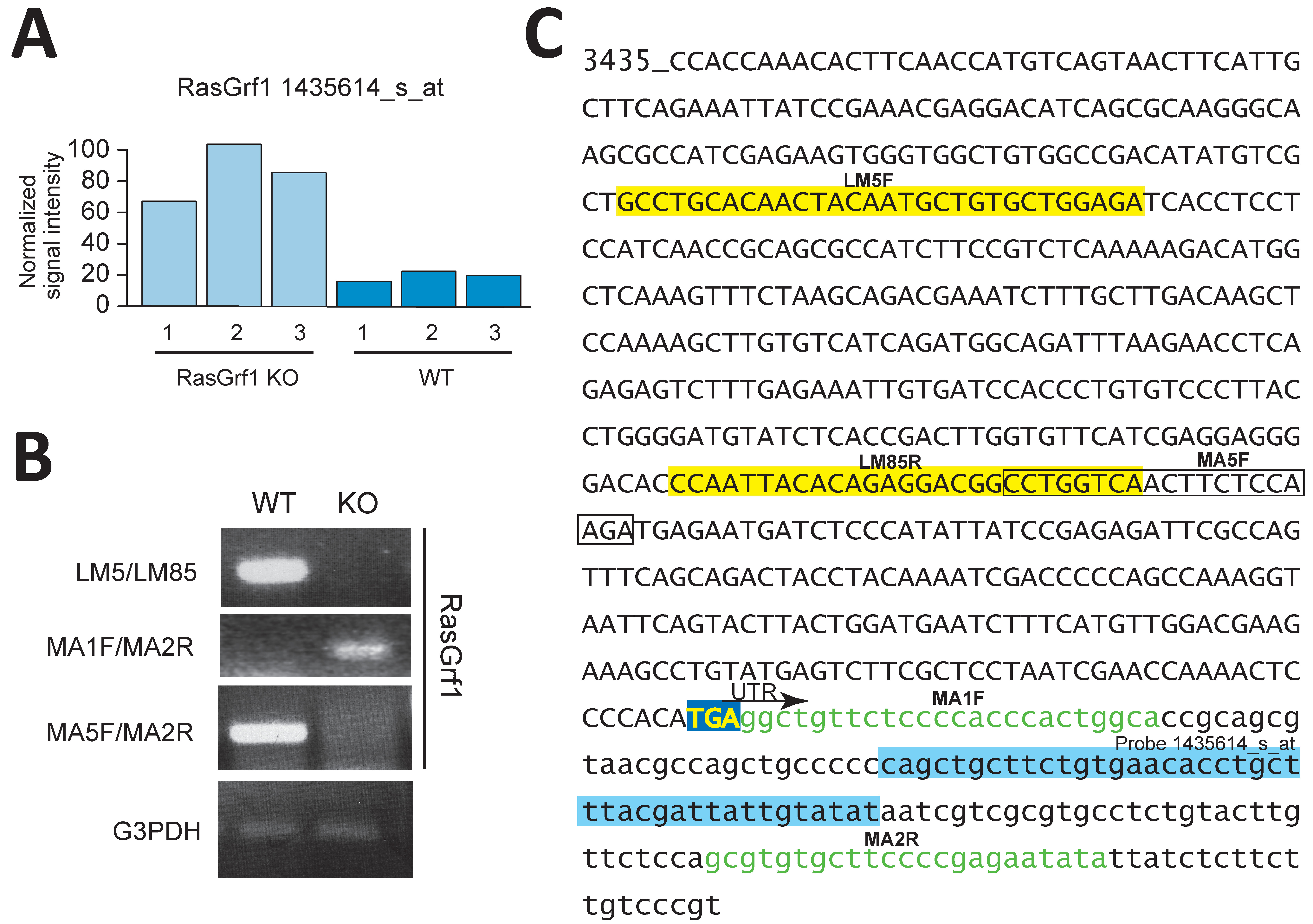

Supplement: Supplementary file 2 — Additional file 2: Figure S1: Transcriptional behavior of genomic sequences located at the 3′ UTR terminal end of the RasGrf1 gene. (A) Hybridization signals produced by Affymetrix probeset 1435614_s_at recognizing the 3′ UTR region of the RasGrf1 locus. Bar plot showing normalized hybridization signals produced by the 1435614_s_at probeset in 6 independent, separate microarray hybridizations with RNA from pancreatic islets including 3 samples from RasGrf1 KO and 3 samples from WT mice. (B) Localization of specific genomic sequences of the 3′ terminal region of RasGrf1 gene that recognized by Affymetrix probesets and primer oligonucleotides used in this study. The coding region is shown in capitals and the 3′ UTR region is shown in italics. The position of the relevant oligonucleotides mentioned in the text (LM5F, LM85R, MA5F, MA1F and MA2R) is indicated by boxes and color changes as appropriate in each case. (C) Confirmatory RT-PCR analysis of WT and RasGrf1 KO RNAs from pancreatic islets. The primer set LM5/LM85 amplifies the 3554–3829 nt region in RasGrf1 mRNA sequence. Primer set MAF5/MA2R amplifies the 3830–4156 nt region, and the set MA1F/MA2R amplifies the 4012–4156 nt segment. Specific oligonucleotides for GAPDH amplified a 90 bp band in both WT and RasGrf1 KO RNA samples. Representative results of three independent experiments are shown. (JPEG 6 MB) [file 12864_2014_6838_MOESM2_ESM.jpeg]
