# Supplementary material for: Transcriptional profiling reveals functional links between RasGrf1 and Pttg1 in pancreatic beta cells
Source: BMC Genomics. 2014 Nov 25;15:1019. doi: 10.1186/1471-2164-15-1019 (PMC4301450; doi:10.1186/1471-2164-15-1019)
Supplement: Supplementary file 3 — Additional file 3: Table S2A: Functional annotation of downregulated, differentially expressed genes in pancreatic islets of RasGrf1 knockout mice. The DAVID functional annotation tool (http://david.abcc.ncifcrf.gov/) was used to identify statistically significant functional associations (p-value <0.1) linking particular gene subsets contained within the list of repressed loci occurring in RasGrf1 KO pancreatic islets (Additional file 1: Table S1, FDR=0.084) to specific Gene Ontology (GO) terms. The column labelled “Biological process” identifies the functional GO terms (level 5) recognized in each case for the corresponding groups of loci listed under the column labelled “Genes induced in RasGrf1KO pancreatic islets (from Additional file 1 : Table S1). The column labeled “Gene Count” indicates the specific number of genes annotated by DAVID to the indicated GO functionality within the list of repressed genes included in Table S1 (Additional file 1). The values under the column “Percentage” are calculated by referring the “Gene Count” numbers to the total number of genes recognized by DAVID (1942, out of a total 1953) within that list. The column labeled “p-value” refers to the statistical significance of the functional associations identified, and contains p-values calculated using the Hypergeometric Distribution and subsequently corrected by implementing the False Discovery Rate (FDR) method [90]. (PDF 466 KB) [file 12864_2014_6838_MOESM3_ESM.pdf]

Table S2A. Functional annotation of downregulated, differentially expressed genes in pancreatic islets of RasGrf1 knockout mice.

The DAVID functional annotation tool (http://david.abcc.ncifcrf.gov/) was used to identify statistically significant functional associations (p-value <0.1) linking particular gene subsets contained within the list of repressed loci occurring in RasGrf1 KO pancreatic islets (Additional file 1: Table S1, FDR=0.084) to specific Gene Ontology (GO) terms.

The column labelled "*Biological process*" identifies the functional GO terms (level 5) recognized in each case for the corresponding groups of loci listed under the column labelled "*Genes induced in RasGrf1 KO pancreatic islets (from Additional file 1: Table S1)*. The column labeled "*Gene Count*" indicates the specific number of loci annotated by DAVID to the indicated GO functionality within the list of repressed genes included in Additional file 1: Table S1. The values under the column "*Percentage*" are calculated by referring the "*Gene Count*" numbers to the total number of genes recognized by DAVID (1942, out of a total 1953) within that list. The column labeled "*p-value*" refers to the statistical significance of the functional associations identified, and contains p-values calculated using the Hypergeometric Distribution and subsequently corrected by implementing the False Discovery Rate (FDR) method (Hochberg and Benjamini, 1990).

Biological process	***Gene Count***	***Percentage***	***p-Value***	***Genes repressed in RasGrf1 KO pancreatic islets (from Additional file 1: Table S1)***
GO:0009888<br>tissue development	123	6,33	9,79E-12	LOR, FGF18, NOG, WNT3A, ENAM, SHH, MAP3K7, PGR, ATG7, GATA4, GAB1, TRP63, PITX1, EGFR, SATB2, RARG, NKX2-6, EOMES, RXRG, KRTDAP, FOXN1, DLL3, CYP26A1, SIX4, MECOM, PTHLH, PDGFRB, MADCAM1, EDA, FGFR2, TWSG1, FGFR3, ENPP1, ERBB2, HOXA11, PTH1R, ELN, SOX5, FOXH1, MUSK, OVOL2, HOXA5, LHX1, OVOL1, NKX2-5, HNRNPAB, NKX2-3, COL18A1, BMP1, MET, CRB3, NR4A3, HOMER1, CSRP3, CACNA2D2, CACNA1S, EPHA2, HOXB4, DLX2, FOXF1A, SFRP1, HOXB5, BMP7, CHRD, TCF15, ELF3, ELF5, PRRX1, GJA1, PRRX2, GLI2, ACVR1B, EDNRB, AGPAT6, BARX1, BARX2, PAX9, SEMA3A, NR2F2, COL11A2, COL11A1, LTB, FGF2, DHCR24, FGF4, PRKCA, PTF1A, OTX2, ESR1, LEF1, CACNG2, EDAR, ESR2, DDR1, ADM, ZFPM2, NGFR, TBX18, MED1, GPRC5D, CSF1, COL2A1, ZBTB17, SUFU, COL9A1, SPRR2D, AMELX, BCL2, POU2F3, KCNE1, TNFRSF19, MYOG, CNNM4, TBX3, TBX5, FZD1, COL5A1, FZD6, SPRR1B, EPOR, BMPR1B, KRT71, SNTA1
GO:0048732<br>gland development	54	2,78	4,35E-11	NOG, NRG3, ELF3, ELF5, WNT3A, GLI2, SHH, GHRHR, GLI1, PGR, AGPAT6, PROP1, APOA1, TNFRSF11A, TRP63, SEMA3A, NEURL1A, PRL, FGF2, PITX1, EGFR, RARG, ESR1, LEF1, EDAR, ESR2, NR0B1, PTHLH, DDR1, GHRH, EDA, TBX19, MED1, FGFR2, XDH, TWSG1, TNF, DRD2, CSF1, ASZ1, POU1F1, HOXA5, BCL2, LHX3, NKX2-5, CYP19A1, NKX2-3, TBX3, IKZF1, MET, NTN1, TNFSF11, SFRP1, BMP7
GO:0009887<br>organ morphogenesis	113	5,82	1,79E-10	FGF18, NOG, WNT3A, SHH, MAP3K7, PGR, ATOH1, PROP1, GATA4, TRP63, APOH, WNT6, INSR, CDH23, EGFR, WNT10B, SATB2, LBX1, RARG, CRYAA, SIX3, EOMES, DLL1, SIX4, THY1, PTHLH, GABRR2, PDGFRA, PDGFRB, TRP73, EDA, FGFR2, TWSG1, FGFR3, WNT5B, HOXA11, ACP5, AHSG, HESX1, CRB1, OVOL2, HOXA4, HOXA5, HOXA7, NKX3-2, NKX2-5, NKX2-3, GNAT1, RECQL4, CRB3, NR4A3, EPHA2, HOXB4, DLX2, DLX1, FOXF1A, TNFSF11, SFRP1, HOXB5, SMARCC1, MYO15, HOXB6, BMP7, CHRD, FOXI1, TCF15, PRRX1, SOBP, GJA1, PRRX2, GLI2, GLI1, BARX2, PAX9, POU4F3, SEMA3A, COL11A2, COL11A1, FGF4, SOX10, NLRP5, PTF1A, OTX2, ESR1, LEF1, ESR2, EDAR, DDR1, ADM, KLHL10, NGFR, TBX18, MED1, TNF, IRX5, CSF1, COL2A1, TCF7L2, SUFU, AMELX, BCL2, WNT8B, FOXL1, TBX3, TBX5, IGF2, VSX2, COL5A1, FZD6, WNT2B, BMPR1B, HTR2C, KRT71

Biological process	*Gene Count*	*Percentage*	*p-Value*	*Genes repressed in RasGrf1 KO pancreatic islets (from Additional file 1: Table S1)*
GO:0008284<br>positive regulation of cell proliferation	67	3,45	2,06E-10	FGF18, NOG, IL6ST, FGF17, PRRX1, TLR4, PRRX2, GLI2, DDR2, SHH, GHRHR, GLI1, IFNG, PRRT1, TRP63, HSF4, FGF2, PRL, LTA, FGF3, FGF4, EGFR, CCKBR, NKX2-6, ESR1, FGF21, CD40, MYCN, PTHLH, SUZ12, PRKCQ, TNFSF13B, ADM, GHRH, IL12A, CDCA7L, NGFR, TBX18, ALOX12, MED1, FGFR2, FGFR3, TNF, CCL2, DRD2, FOXM1, CSF1, PTH1R, ST8SIA1, POU1F1, LHX1, BCL2, LHX5, FIGF, FGFBP1, NKX2-5, RECQL4, COL18A1, TBX3, IL7, SPHK1, IGH-6, VSX2, AGER, NTN1, CDKN1A, GKN1
GO:0007417<br>central nervous system development	80	4,12	2,42E-10	NOG, EVX1, WNT3A, RORA, GLI2, GHRHR, SHH, KLHL1, CKB, GLI1, IGHMBP2, ATOH1, PROP1, NKX6-2, ATG7, PRRT1, PITX3, NR2F2, PITX1, EGFR, PHOX2A, SOX10, LBX1, NKX2-6, BARHL1, OTX2, PTF1A, SIX3, EOMES, LEF1, CYP26A1, ESR2, VAX1, MECOM, TACC3, ABT1, GHRH, HES3, TRP73, NGFR, CLN8, TBX19, MED1, FGFR2, TWSG1, CDK5R1, FGFR3, ADORA2A, DRD2, ASZ1, SOX5, POU1F1, SUFU, TPM3, AHSG, TAL2, HESX1, LHX1, BCL11B, BCL2, LHX3, LHX5, RUNX1, DCLK1, DMBX1, PLP1, IKZF1, TBX3, MET, SPHK1, LMX1A, AGER, EIF2B1, SALL3, DLX2, DLX1, PHGDH, EPOR, CHRD, NFIB
GO:0009891<br>positive regulation of biosynthetic process	103	5,30	6,14E-09	TLR4, RORA, FOXO3, TLR6, TLR7, CITED4, SHH, TLR9, ATOH1, PROP1, GATA5, GATA4, IFNG, TRP63, IL1B, CIITA, WNT10B, SATB2, RARG, RXRG, SIX3, FGF23, SIX4, IL21, MECOM, PTHLH, PRDM9, HES3, MED17, TRP73, ADORA2A, SOX5, SOX4, ELK1, SFPI1, FOXH1, OVOL2, HOXA7, RUNX1, NKX2-5, HNRNPAB, NKX2-3, IKZF1, KLF13, ESRRG, TEAD2, NR4A3, TEAD3, DDX5, USF1, ABCG4, DLX2, BMP7, FOXI1, KLF1, EVX1, FOXK1, GLI2, GLI1, BARX2, PAX9, GUCY1A3, HSF4, LTB, FGF2, TOP2A, PSMD9, SOX10, OTX2, PTF1A, LEF1, PRKCQ, AYM1, GHRH, ZFPM2, TBX19, MED1, TNF, FOXM1, GLIS1, NFYA, ZBTB17, POU1F1, TCF7L2, SEC14L2, TNFRSF1A, NPAS2, REL, SORBS1, BCL11B, POU2F3, POU2F2, MYOG, NFATC2, TCFEB, PTX3, CD27, TBX3, TBX5, VSX2, NR5A2, MESP2, NFIB
GO:0031328<br>positive regulation of cellular biosynthetic process	101	5,20	1,59E-08	TLR4, RORA, FOXO3, TLR6, TLR7, CITED4, SHH, TLR9, ATOH1, PROP1, GATA5, GATA4, IFNG, TRP63, IL1B, CIITA, WNT10B, SATB2, RARG, RXRG, SIX3, FGF23, SIX4, IL21, MECOM, PTHLH, PRDM9, HES3, MED17, TRP73, ADORA2A, SOX5, SOX4, ELK1, SFPI1, FOXH1, OVOL2, HOXA7, RUNX1, NKX2-5, NKX2-3, HNRNPAB, IKZF1, KLF13, ESRRG, TEAD2, NR4A3, TEAD3, DDX5, USF1, DLX2, BMP7, FOXI1, KLF1, EVX1, FOXK1, GLI2, GLI1, BARX2, PAX9, GUCY1A3, HSF4, LTB, FGF2, TOP2A, PSMD9, SOX10, OTX2, PTF1A, LEF1, PRKCQ, AYM1, GHRH, ZFPM2, TBX19, MED1, TNF, FOXM1, GLIS1, NFYA, ZBTB17, POU1F1, TCF7L2, TNFRSF1A, NPAS2, REL, SORBS1, BCL11B, POU2F3, POU2F2, MYOG, NFATC2, TCFEB, PTX3, CD27, TBX3, TBX5, VSX2, NR5A2, MESP2, NFIB
GO:0007420<br>brain development	63	3,24	1,72E-08	NOG, WNT3A, RORA, GLI2, SHH, GHRHR, KLHL1, CKB, GLI1, ATOH1, PROP1, ATG7, PRRT1, NR2F2, PITX3, PITX1, PHOX2A, EGFR, NKX2-6, BARHL1, PTF1A, OTX2, SIX3, EOMES, LEF1, ESR2, VAX1, MECOM, TACC3, GHRH, HES3, TRP73, TBX19, MED1, FGFR2, TWSG1, CDK5R1, FGFR3, DRD2, ASZ1, POU1F1, AHSG, TPM3, TAL2, HESX1, LHX1, BCL2, LHX3, LHX5, DCLK1, DMBX1, IKZF1, TBX3, MET, SPHK1, LMX1A, AGER, SALL3, DLX2, DLX1, EPOR, CHRD, NFIB

Biological process	*Gene Count*	*Percentage*	*p-Value*	*Genes repressed in RasGrf1 KO pancreatic islets (from Additional file 1: Table S1)*
GO:0010557 positive regulation of macromolecule biosynthetic process	97	4,99	3,13E-08	TLR4, RORA, FOXO3, TLR6, TLR7, CITED4, SHH, TLR9, ATOH1, PROP1, GATA5, IFNG, GATA4, TRP63, IL1B, CIITA, WNT10B, SATB2, RARG, RXRG, SIX3, FGF23, SIX4, IL21, MECOM, PRDM9, HES3, MED17, TRP73, SOX5, ELK1, SOX4, SFPI1, FOXH1, OVOL2, HOXA7, RUNX1, NKX2-5, NKX2-3, HNRNPAB, IKZF1, KLF13, ESRRG, TEAD2, NR4A3, TEAD3, DDX5, USF1, DLX2, BMP7, FOXI1, KLF1, EVX1, FOXK1, GLI2, GLI1, BARX2, PAX9, HSF4, LTB, FGF2, TOP2A, PSMD9, SOX10, OTX2, PTF1A, LEF1, PRKCQ, AYM1, GHRH, ZFPM2, TBX19, MED1, TNF, FOXM1, GLIS1, NFYA, ZBTB17, POU1F1, TCF7L2, TNFRSF1A, NPAS2, REL, SORBS1, BCL11B, POU2F3, POU2F2, MYOG, NFATC2, TCFEB, CD27, TBX3, TBX5, VSX2, NR5A2, MESP2, NFIB
GO:0031325 positive regulation of cellular metabolic process	112	5,77	4,22E-08	TLR4, RORA, FOXO3, TLR6, TLR7, CITED4, GHRHR, SHH, TLR9, ATOH1, PROP1, GATA5, ATG7, GATA4, IFNG, TRP63, IL1B, MLST8, CIITA, WNT10B, SATB2, RARG, SIX3, RXRG, FGF23, TNFRSF14, SIX4, CD40, MECOM, IL21, PTHLH, PRDM9, HES3, MED17, TRP73, FGFR3, ADORA2A, SOX5, SOX4, ELK1, SFPI1, FOXH1, OVOL2, HOXA7, RUNX1, NKX2-5, HNRNPAB, NKX2-3, IKZF1, KLF13, ESRRG, IGH-6, TEAD2, NR4A3, TEAD3, DDX5, USF1, DLX2, ADRA1B, BMP7, FOXI1, KLF1, FOXK1, EVX1, GLI2, GLI1, BARX2, PAX9, GUCY1A3, HSF4, LTB, FGF2, TOP2A, PSMD9, PRKCA, SOX10, OTX2, PTF1A, LEF1, EYA3, PRKCQ, AYM1, GHRH, ZFPM2, TBX19, MED1, TNF, FOXM1, GLIS1, NFYA, ZBTB17, POU1F1, TCF7L2, TNFRSF1A, NPAS2, REL, SORBS1, BCL11B, POU2F3, BCL2, POU2F2, MYOG, TCFEB, NFATC2, PTX3, CD27, TBX3, TBX5, VSX2, NR5A2, MESP2, NFIB
GO:0010604 positive regulation of macromolecule metabolic process	110	5,66	5,92E-08	TLR4, RORA, FOXO3, TLR6, TLR7, CITED4, SHH, TLR9, ATOH1, PROP1, GATA5, ATG7, GATA4, IFNG, TRP63, IL1B, MLST8, CIITA, WNT10B, SATB2, RARG, SIX3, RXRG, FGF23, TNFRSF14, SIX4, CD40, MECOM, IL21, PRDM9, HES3, MED17, TRP73, FGFR3, SOX5, SOX4, ELK1, SFPI1, FOXH1, OVOL2, HOXA7, RUNX1, NKX2-5, HNRNPAB, NKX2-3, IKZF1, KLF13, ESRRG, IGH-6, TEAD2, NR4A3, TEAD3, DDX5, USF1, DLX2, ADRA1B, BMP7, FOXI1, KLF1, EVX1, FOXK1, GLI2, GLI1, BARX2, PAX9, HSF4, LTB, FGF2, TOP2A, PSMD9, PRKCA, SOX10, OTX2, PTF1A, ESR1, LEF1, EYA3, PRKCQ, AYM1, TNFSF13B, GHRH, ZFPM2, TBX19, MED1, TNF, FOXM1, GLIS1, NFYA, ZBTB17, POU1F1, TCF7L2, TNFRSF1A, NPAS2, REL, SORBS1, BCL11B, POU2F3, BCL2, POU2F2, MYOG, NFATC2, TCFEB, CD27, IL2RB, TBX3, TBX5, VSX2, NR5A2, MESP2, NFIB
GO:0045893 positive regulation of transcription, DNA-dependent	80	4,12	7,08E-08	FOXK1, EVX1, FOXO3, RORA, GLI2, SHH, GLI1, ATOH1, BARX2, PROP1, PAX9, GATA4, IFNG, TRP63, HSF4, FGF2, TOP2A, CIITA, SOX10, WNT10B, SATB2, RARG, OTX2, SIX3, RXRG, FGF23, LEF1, SIX4, MECOM, AYM1, PRDM9, MED17, HES3, ZFPM2, TRP73, TBX19, MED1, TNF, FOXM1, GLIS1, SOX5, SOX4, ELK1, SFPI1, NFYA, ZBTB17, POU1F1, TCF7L2, FOXH1, TNFRSF1A, NPAS2, REL, OVOL2, BCL11B, POU2F3, HOXA7, MYOG, RUNX1, NFATC2, TCFEB, NKX2-5, HNRNPAB, NKX2-3, IKZF1, TBX3, KLF13, TBX5, ESRRG, TEAD2, NR4A3, TEAD3, VSX2, USF1, DLX2, NR5A2, BMP7, MESP2, FOXI1, KLF1, NFIB

Biological process	*Gene Count*	*Percentage*	*p-Value*	*Genes repressed in RasGrf1 KO pancreatic islets (from Additional file 1: Table S1)*
GO:0010628 positive regulation of gene expression	90	4,63	7,36E-08	FOXK1, EVX1, FOXO3, RORA, GLI2, CITED4, SHH, GLI1, ATOH1, BARX2, PROP1, GATA5, PAX9, GATA4, IFNG, TRP63, HSF4, FGF2, TOP2A, PSMD9, CIITA, SOX10, WNT10B, SATB2, RARG, OTX2, PTF1A, SIX3, ESR1, RXRG, FGF23, LEF1, SIX4, MECOM, AYM1, PRDM9, TNFSF13B, GHRH, HES3, MED17, ZFPM2, TRP73, TBX19, MED1, TNF, FOXM1, GLIS1, SOX5, SOX4, ELK1, SFPI1, NFYA, ZBTB17, POU1F1, TCF7L2, FOXH1, TNFRSF1A, NPAS2, REL, OVOL2, BCL11B, POU2F3, POU2F2, HOXA7, MYOG, NFATC2, RUNX1, TCFEB, NKX2-5, HNRNPAB, NKX2-3, IL2RB, IKZF1, TBX3, KLF13, TBX5, ESRRG, TEAD2, NR4A3, TEAD3, DDX5, VSX2, USF1, DLX2, NR5A2, BMP7, MESP2, KLF1, FOXI1, NFIB
GO:0045935 positive regulation of nucleobase, nucleoside, nucleotide and nucleic acid metabolic process	93	4,79	7,49E-08	FOXK1, EVX1, FOXO3, RORA, GLI2, CITED4, GHRHR, SHH, GLI1, ATOH1, BARX2, PROP1, GATA5, PAX9, GATA4, IFNG, TRP63, GUCY1A3, HSF4, FGF2, TOP2A, PSMD9, CIITA, SOX10, WNT10B, SATB2, RARG, OTX2, PTF1A, SIX3, RXRG, FGF23, LEF1, SIX4, CD40, MECOM, EYA3, PTHLH, AYM1, PRDM9, GHRH, HES3, MED17, ZFPM2, TRP73, TBX19, MED1, TNF, ADORA2A, FOXM1, GLIS1, SOX5, SOX4, ELK1, SFPI1, NFYA, ZBTB17, POU1F1, TCF7L2, FOXH1, TNFRSF1A, NPAS2, REL, OVOL2, BCL11B, POU2F3, POU2F2, HOXA7, MYOG, NFATC2, RUNX1, TCFEB, NKX2-5, HNRNPAB, NKX2-3, IKZF1, TBX3, KLF13, TBX5, ESRRG, TEAD2, NR4A3, TEAD3, DDX5, VSX2, USF1, DLX2, NR5A2, BMP7, MESP2, KLF1, FOXI1, NFIB
GO:0051173 positive regulation of nitrogen compound metabolic process	95	4,89	8,48E-08	FOXK1, EVX1, TLR4, FOXO3, RORA, GLI2, CITED4, GHRHR, SHH, GLI1, ATOH1, BARX2, PROP1, GATA5, PAX9, GATA4, IFNG, TRP63, GUCY1A3, HSF4, FGF2, TOP2A, PSMD9, CIITA, SOX10, WNT10B, SATB2, RARG, OTX2, PTF1A, SIX3, RXRG, FGF23, LEF1, SIX4, CD40, MECOM, EYA3, PTHLH, AYM1, PRDM9, GHRH, HES3, MED17, ZFPM2, TRP73, TBX19, MED1, TNF, ADORA2A, FOXM1, GLIS1, SOX5, SOX4, ELK1, SFPI1, NFYA, ZBTB17, POU1F1, TCF7L2, FOXH1, TNFRSF1A, NPAS2, REL, OVOL2, BCL11B, POU2F3, POU2F2, HOXA7, MYOG, NFATC2, RUNX1, PTX3, TCFEB, NKX2-5, HNRNPAB, NKX2-3, IKZF1, TBX3, KLF13, TBX5, ESRRG, TEAD2, NR4A3, TEAD3, DDX5, VSX2, USF1, DLX2, NR5A2, BMP7, MESP2, KLF1, FOXI1, NFIB
GO:0048568 embryonic organ development	54	2,78	9,02E-08	NOG, WNT3A, PRRX1, SOBP, PRRX2, GLI2, SHH, GLI1, ATOH1, GATA4, GAB1, POU4F3, COL11A1, CDH23, EGFR, SATB2, RARG, OTX2, EOMES, LEF1, DLL1, SIX4, ADM, ZFPM2, MED1, FGFR2, COL2A1, TCF7L2, HESX1, OVOL2, HOXA4, HOXA5, HOXA7, NKX3-2, TCFEB, RUNX1, ESRRB, PCDH12, GJB5, ITGA4, ESX1, NR4A3, EPHA2, FZD6, DLX2, HOXB4, WNT7B, FOXF1A, HOXB5, HOXB6, MYO15, BMP7, FOXI1, KLF1

Biological process	*Gene Count*	*Percentage*	*p-Value*	*Genes repressed in RasGrf1 KO pancreatic islets (from Additional file 1: Table S1)*
GO:0051254 positive regulation of RNA metabolic process	80	4,12	9,83E-08	FOXK1, EVX1, FOXO3, RORA, GLI2, SHH, GLI1, ATOH1, BARX2, PROP1, PAX9, GATA4, IFNG, TRP63, HSF4, FGF2, TOP2A, CIITA, SOX10, WNT10B, SATB2, RARG, OTX2, SIX3, RXRG, FGF23, LEF1, SIX4, MECOM, AYM1, PRDM9, MED17, HES3, ZFPM2, TRP73, TBX19, MED1, TNF, FOXM1, GLIS1, SOX5, SOX4, ELK1, SFPI1, NFYA, ZBTB17, POU1F1, TCF7L2, FOXH1, TNFRSF1A, NPAS2, REL, OVOL2, BCL11B, POU2F3, HOXA7, MYOG, RUNX1, NFATC2, TCFEB, NKX2-5, HNRNPAB, NKX2-3, IKZF1, TBX3, KLF13, TBX5, ESRRG, TEAD2, NR4A3, TEAD3, VSX2, USF1, DLX2, NR5A2, BMP7, MESP2, FOXI1, KLF1, NFIB
GO:0045941 positive regulation of transcription	87	4,48	1,72E-07	FOXK1, EVX1, FOXO3, RORA, GLI2, CITED4, SHH, GLI1, ATOH1, BARX2, PROP1, GATA5, PAX9, GATA4, IFNG, TRP63, HSF4, FGF2, TOP2A, PSMD9, CIITA, SOX10, WNT10B, SATB2, RARG, OTX2, PTF1A, SIX3, RXRG, FGF23, LEF1, SIX4, MECOM, AYM1, PRDM9, GHRH, MED17, HES3, ZFPM2, TRP73, TBX19, MED1, TNF, FOXM1, GLIS1, SOX5, SOX4, ELK1, SFPI1, NFYA, ZBTB17, POU1F1, TCF7L2, FOXH1, TNFRSF1A, NPAS2, REL, OVOL2, BCL11B, POU2F3, POU2F2, HOXA7, MYOG, RUNX1, NFATC2, TCFEB, NKX2-5, HNRNPAB, NKX2-3, IKZF1, TBX3, KLF13, TBX5, ESRRG, TEAD2, NR4A3, TEAD3, DDX5, VSX2, USF1, DLX2, NR5A2, BMP7, MESP2, KLF1, FOXI1, NFIB
GO:0022612 gland morphogenesis	27	1,39	1,99E-07	FGFR2, TWSG1, NOG, TNF, NRG3, ELF3, CSF1, GLI2, SHH, PGR, PROP1, BCL2, TRP63, SEMA3A, NKX2-3, EGFR, RARG, TBX3, ESR1, ESR2, EDAR, PTHLH, DDR1, SFRP1, EDA, BMP7, MED1
GO:0051252 regulation of RNA metabolic process	212	10,92	6,55E-07	MEF2B, HIRA, RORA, SHH, PGR, ZFP92, NKX6-2, IFNG, PITX3, PITX1, PHOX2A, WNT10B, RARG, NKX2-6, BARHL1, RXRG, EOMES, HNF4G, MECOM, CPHX, PRDM9, MED17, HES3, HES2, HOXA11, SFPI1, HESX1, FOXH1, MUSK, LHX1, LHX3, LHX5, NKX2-4, NKX2-5, NKX2-3, HNRNPAB, DMBX1, IKZF1, ESRRB, KLF13, ESRRG, USF1, ZFP444, DLX3, DLX2, DLX1, FOXF1A, NR1I2, BMP7, KLF1, FOXI1, BACH2, EVX1, ELF3, ELF5, NFKB2, GLI2, KCNIP3, GLI1, HSF4, SPIC, NR2F2, TOP2A, FGF2, CSDC2, BATF2, PTF1A, GTF2H4, TLE4, MYCN, JMY, TBX18, KCNH3, TBX19, MED1, MTDH, ZBTB17, SUFU, ZFP111, MYCL1, ZFP36L2, SORBS3, STAT4, BCL11B, ETV2, MLLT1, PER3, TCFEB, BAZ2A, TBX3, ZFP57, TBX5, LMX1A, ZFP707, RHOX9, NR5A2, HMX1, CBX3, TBP, CBX2, ANKRD1, HOXD1, FOXO3, MAF1, BATF, HOXC6, ATOH1, PROP1, GATA5, GATA4, TRP63, CIITA, SATB2, LBX1, TCFAP2C, RELB, SIX3, FOXN1, FGF23, VAX1, SIX4, NR0B1, SIX6, SUZ12, SPDEF, MGA, TRP73, NFE2L3, ASCL3, TSHZ2, ADORA2A, ONECUT3, SOX5, SOX4, ELK1, HOXA4, OVOL2, ELK4, HOXA5, HOXA7, OVOL1, NKX3-2, RUNX1, NFE2, SUV39H1, TEAD2, TEAD3, NR4A3, ESX1, HOXB4, SEBOX, HOXB5, HOXB6, RFX1, POU6F1, AEBP1, SOX21, FOXK1, PRRX1, PRRX2, CBFA2T3, NFATC2IP, BARX1, BARX2, LBH, PAX9, POU4F3, TBL1XR1, SOX10, NLRP5, FOXJ1, SOX14, SLA2, OTX2, ESR1, LEF1, ESR2, NRIP2, AYM1, ZFPM2, CALCR, IRX4, IRX3, IRX5, TNF, FOXM1, GLIS1, NFYA, POU1F1, TCF7L2, ZFP493, NPAS1, TNFRSF1A, NPAS2, TNFRSF1B, ZKSCAN14, REL, POU2F3, RHOX2A, POU2F2, NFATC4, MYOG, NFATC2, ERF, FOXL1, CREB3, VSX2, ZFP286, HEYL, SETD7, MESP2, NFIB

Biological process	*Gene Count*	*Percentage*	*p-Value*	*Genes repressed in RasGrf1 KO pancreatic islets (from Additional file 1: Table S1)*
GO:0022008 neurogenesis	93	4,79	8,32E-07	GPRIN1, NOG, EVX1, WNT3A, CSPG4, PIP5K1C, GJA1, RORA, GLI2, SHH, KLHL1, IGHMBP2, METRN, ATOH1, SLC1A3, NKX6-2, ATG7, POU4F3, SEMA3A, PITX3, NR2F2, FGF2, CDH23, PHOX2A, SOX10, SATB2, LBX1, LIMK1, BARHL1, OTX2, PTF1A, EOMES, DLL1, ESR2, VAX1, MMP14, TACC3, SLIT1, THY1, GABRR2, SLITRK1, ABT1, HES3, TRP73, NGFR, CLN8, NGF, IRX3, CDK5R1, FGFR3, CCK, IRX5, DRD3, ADORA2A, TNFRSF12A, DRD2, ERBB2, ASZ1, SOX5, CRB1, LHX1, RAC3, INPP5J, BCL11B, BCL2, LHX3, LHX5, RUNX1, DCLK1, GNAT1, KLK8, PLP1, PLA2G10, MET, NTNG2, LMX1A, VSX2, EIF2B1, GAS7, NTN1, EPHA2, SALL3, SEMA6A, EPHA4, DLX2, DLX1, EPHA7, SEMA6C, PHGDH, CACNA1F, CIT, BMP7, BMPR1B
GO:0006355 regulation of transcription, DNA-dependent	208	10,71	1,05E-06	MEF2B, HIRA, RORA, SHH, PGR, ZFP92, NKX6-2, IFNG, PITX3, PITX1, PHOX2A, WNT10B, RARG, NKX2-6, BARHL1, RXRG, EOMES, HNF4G, MECOM, CPHX, PRDM9, MED17, HES3, HES2, HOXA11, SFPI1, HESX1, FOXH1, MUSK, LHX1, LHX3, LHX5, NKX2-4, NKX2-5, NKX2-3, HNRNPAB, DMBX1, IKZF1, ESRRB, KLF13, ESRRG, USF1, ZFP444, DLX3, DLX2, DLX1, FOXF1A, NR1I2, BMP7, KLF1, FOXI1, BACH2, EVX1, ELF3, ELF5, NFKB2, GLI2, KCNIP3, GLI1, HSF4, SPIC, NR2F2, FGF2, TOP2A, CSDC2, BATF2, PTF1A, GTF2H4, TLE4, MYCN, JMY, TBX18, KCNH3, TBX19, MED1, MTDH, ZBTB17, SUFU, ZFP111, MYCL1, SORBS3, STAT4, BCL11B, ETV2, MLLT1, PER3, TCFEB, BAZ2A, TBX3, ZFP57, TBX5, LMX1A, ZFP707, RHOX9, NR5A2, HMX1, CBX3, TBP, CBX2, ANKRD1, HOXD1, FOXO3, MAF1, BATF, HOXC6, ATOH1, PROP1, GATA5, GATA4, TRP63, CIITA, SATB2, LBX1, TCFAP2C, RELB, SIX3, FOXN1, FGF23, VAX1, SIX4, NR0B1, SIX6, SUZ12, SPDEF, MGA, TRP73, NFE2L3, ASCL3, TSHZ2, ADORA2A, ONECUT3, SOX5, SOX4, ELK1, HOXA4, OVOL2, ELK4, HOXA5, OVOL1, HOXA7, NKX3-2, RUNX1, NFE2, SUV39H1, TEAD2, TEAD3, NR4A3, ESX1, HOXB4, SEBOX, HOXB5, HOXB6, RFX1, POU6F1, AEBP1, SOX21, FOXK1, PRRX1, PRRX2, CBFA2T3, NFATC2IP, BARX1, BARX2, LBH, PAX9, POU4F3, TBL1XR1, SOX10, FOXJ1, SOX14, SLA2, OTX2, ESR1, LEF1, ESR2, NRIP2, AYM1, ZFPM2, IRX4, IRX3, IRX5, TNF, FOXM1, GLIS1, NFYA, POU1F1, TCF7L2, ZFP493, NPAS1, TNFRSF1A, NPAS2, ZKSCAN14, REL, POU2F3, RHOX2A, POU2F2, NFATC4, MYOG, NFATC2, ERF, FOXL1, CREB3, VSX2, ZFP286, HEYL, SETD7, MESP2, NFIB
GO:0048699 generation of neurons	86	4,43	2,39E-06	GPRIN1, NOG, EVX1, WNT3A, PIP5K1C, GJA1, RORA, GLI2, SHH, KLHL1, IGHMBP2, METRN, ATOH1, SLC1A3, NKX6-2, ATG7, POU4F3, SEMA3A, PITX3, NR2F2, CDH23, PHOX2A, SATB2, LBX1, LIMK1, BARHL1, OTX2, PTF1A, EOMES, DLL1, ESR2, VAX1, SLIT1, THY1, GABRR2, SLITRK1, ABT1, HES3, TRP73, NGFR, CLN8, NGF, IRX3, CDK5R1, FGFR3, CCK, IRX5, DRD3, ADORA2A, TNFRSF12A, DRD2, ERBB2, ASZ1, SOX5, CRB1, LHX1, RAC3, INPP5J, BCL11B, BCL2, LHX3, LHX5, RUNX1, DCLK1, GNAT1, KLK8, PLA2G10, MET, NTNG2, LMX1A, VSX2, GAS7, NTN1, EPHA2, SALL3, SEMA6A, EPHA4, DLX2, DLX1, EPHA7, SEMA6C, PHGDH, CACNA1F, CIT, BMP7, BMPR1B
GO:0030900 forebrain development	39	2,01	2,48E-06	TWSG1, NOG, FGFR3, DRD2, WNT3A, POU1F1, GLI2, SHH, GHRHR, AHSG, GLI1, TAL2, HESX1, PROP1, LHX1, ATG7, LHX3, NR2F2, DCLK1, PITX1, EGFR, IKZF1, TBX3, NKX2-6, OTX2, EOMES, SIX3, LEF1, LMX1A, MECOM, TACC3, SALL3, DLX2, DLX1, GHRH, TRP73, CHRD, TBX19, NFIB
GO:0030879 mammary gland development	25	1,29	4,55E-06	FGFR2, XDH, NRG3, ELF3, ELF5, CSF1, WNT3A, ASZ1, GLI2, GHRHR, PGR, AGPAT6, TNFRSF11A, NEURL1A, PRL, FGF2, TBX3, MET, ESR1, LEF1, NTN1, PTHLH, DDR1, TNFSF11, MED1

Biological process	***Gene Count***	***Percentage***	***p-Value***	***Genes repressed in RasGrf1 KO pancreatic islets (from Additional file 1: Table S1)***
GO:0048562 embryonic organ morphogenesis	37	1,91	6,92E-06	FGFR2, NOG, WNT3A, PRRX1, SOBP, COL2A1, PRRX2, GLI2, TCF7L2, SHH, GLI1, HESX1, ATOH1, HOXA4, OVOL2, HOXA5, GATA4, HOXA7, NKX3-2, POU4F3, COL11A1, CDH23, SATB2, RARG, OTX2, DLL1, NR4A3, SIX4, EPHA2, FZD6, HOXB4, DLX2, HOXB5, MYO15, HOXB6, BMP7, FOXI1
GO:0051216 cartilage development	23	1,18	9,33E-06	PRKCA, FGF18, NOG, SATB2, BMP1, RARG, FGFR3, HOXA11, PTH1R, SOX5, PRRX1, COL2A1, PRRX2, DLX2, COL9A1, BARX2, HOXA5, BMPR1B, BMP7, COL11A2, COL11A1, PITX1, FGF4
GO:0043009 chordate embryonic development	73	3,76	1,41E-05	NOG, WNT3A, GNA12, PRRX1, GJA1, PRRX2, HOXD1, GLI2, SHH, MAP3K7, HOXC6, ACVR1B, GATA4, GAB1, ETL4, COL11A1, MLL2, EGFR, SOX10, SATB2, NLRP5, NKX2-6, EOMES, DLL3, LEF1, DLL1, SIX4, MYH9, MECOM, ASCL2, ADM, HES3, PDGFRA, PDGFRB, ZFPM2, TBX18, MED1, FGFR2, RIC8, TCF7L2, SUFU, INPP5K, OVOL2, HOXA4, HOXA5, DNMT3L, HOXA7, NKX3-2, RUNX1, TCFEB, NKX2-5, TBX3, ESRRB, MYO1E, PCDH12, GJB5, ITGA4, ESX1, EPHA2, FZD6, HBA-A1, HOXB4, DLX2, DLX1, FOXF1A, WNT7B, SFRP1, HOXB5, HOXB6, PHGDH, MESP2, KLF1, TCF15
GO:0060429 epithelium development	52	2,68	1,92E-05	LOR, NOG, ELF3, ELF5, GJA1, GLI2, SHH, MAP3K7, PGR, BARX1, AGPAT6, TRP63, SEMA3A, FGF2, EGFR, RARG, NKX2-6, ESR1, FOXN1, ESR2, MECOM, PTHLH, DDR1, ADM, MADCAM1, TBX18, MED1, FGFR2, GPRC5D, FGFR3, CSF1, HOXA11, SUFU, OVOL2, HOXA5, SPRR2D, POU2F3, BCL2, KCNE1, NKX2-3, COL18A1, TBX3, TBX5, FZD1, CRB3, FZD6, HOXB4, FOXF1A, SFRP1, HOXB5, SPRR1B, BMP7
GO:0007423 sensory organ development	50	2,57	1,95E-05	WNT3A, PRRX1, SOBP, PRRX2, SHH, ATOH1, POU4F3, HSF4, COL11A1, CDH23, CRYBB2, RARG, CRYAA, NKX2-6, PTF1A, OTX2, SIX3, DLL1, VAX1, SIX4, THY1, GABRR2, DDR1, CLN8, MED1, FGFR2, TWSG1, IRX5, FGFR3, COL2A1, HESX1, MIP, CRB1, BCL11B, BCL2, NKX3-2, NKX2-5, GNAT1, IKZF1, NR4A3, VSX2, PRPH2, CACNA1S, FZD6, JMJD6, MYO15, BMPR1B, BMP7, FOXI1, TCF15
GO:0010647 positive regulation of cell communication	40	2,06	2,16E-05	FGFR2, TWSG1, FGF18, TNF, FGFR3, GRIK1, DRD3, ADORA2A, CSF1, PRRX1, NCS1, SOX4, TLR4, PRRX2, ITSN1, TLR6, MAP3K7, SORBS3, SLC1A3, SOS1, IFNG, GAB1, IL1B, PRL, LTB, FGFBP1, CD27, LTA, FGF23, IGH-6, FGF21, NFAM1, PDE6H, PDE6G, TRADD, NCAM1, LAMA2, TNFSF11, CARTPT, EDA
GO:0008202 steroid metabolic process	35	1,80	4,35E-05	CYB5R3, CYP24A1, MVD, HMGCR, HSD17B1, CYP11B2, HSD3B4, APOC1, SHH, STARD3, APOA1, SERPINA6, CYP7A1, LCAT, APOC3, SRD5A2, HSD17B7, DHCR24, CYP19A1, CUBN, CYP46A1, OSBPL9, CYP21A1, ESR1, AKR1C21, AFP, CYP17A1, SULT1B1, AKR1C18, SDR42E1, MVK, BMPR1B, NR5A2, MBTPS1, CLN8

Biological process	*Gene Count*	*Percentage*	*p-Value*	*Genes repressed in RasGrf1 KO pancreatic islets (from Additional file 1: Table S1)*
GO:0045596 negative regulation of cell differentiation	38	1,96	4,87E-05	TWSG1, IRX3, NOG, DRD3, ENPP1, NFKBIA, PRDX2, GLI2, TCF7L2, SHH, IAPP, NKX6-2, HOXA7, TRP63, SEMA3A, INPP5D, FGF4, KLK8, LBX1, TBX3, ESRRB, FOXJ1, DLL1, VAX1, LMX1A, NR0B1, NTN1, TCL1, THY1, PTHLH, DLX2, DLX1, PLA2G2A, CARTPT, BMP7, CIT, CHRD, TOB2
GO:0048546 digestive tract morphogenesis	13	0,67	6,82E-05	FGFR2, EGFR, SOX10, GLI2, TCF7L2, SHH, FOXF1A, OVOL2, BCL2, GATA4, TRP63, TRP73, NKX2-3
GO:0051952 regulation of amine transport	11	0,57	1,09E-04	TNF, DRD3, GRIK1, ADORA2A, DRD2, CARTPT, CHRNA4, CHRNA6, HTR2C, LTB, LTA
GO:0016481 negative regulation of transcription	63	3,24	1,18E-04	HMX1, FOXK1, HR, STRM, PRRX1, CBX3, CBX2, GLI2, MAF1, SHH, KCNIP3, BARX2, PROP1, NKX6-2, TRP63, HSF4, NR2F2, CIITA, TBL1XR1, SATB2, RARG, SOX14, FOXJ1, SLA2, SIX3, LEF1, TLE4, VAX1, NR0B1, NRIP2, MXD3, MXD4, SUZ12, HES3, ZFPM2, C1D, MTDH, TNF, GLIS1, POU1F1, SUFU, FOXH1, NPAS1, SORBS3, OVOL2, RHOX2A, HOXA7, OVOL1, PER3, NKX2-5, BAZ2A, HNRNPAB, DMBX1, INSM2, IKZF1, TBX3, ZFP57, SUV39H1, FZD1, HOXB4, DLX2, DLX1, NR1I2
GO:0006820 anion transport	29	1,49	1,40E-04	SLC5A5, SLC22A12, SLC20A2, GLRA3, CLCNKA, ASZ1, CLCNKB, SLCO1A1, SLC1A3, P2RY4, SLC4A8, SLC22A6, SLC4A1, SLC4A5, GABRA2, SLC12A1, GABRA1, SLC12A3, SLC12A5, TST, GABRR2, SLC16A3, SLC26A3, SLC26A6, SLC25A13, CLCN6, CLCN7, CLN8, MPST
GO:0045892 negative regulation of transcription, DNA-dependent	54	2,78	1,59E-04	HMX1, FOXK1, PRRX1, CBX3, CBX2, MAF1, GLI2, SHH, KCNIP3, BARX2, PROP1, NKX6-2, TRP63, HSF4, NR2F2, CIITA, TBL1XR1, SATB2, RARG, SOX14, FOXJ1, SLA2, LEF1, TLE4, VAX1, NR0B1, NRIP2, SUZ12, HES3, ZFPM2, MTDH, TNF, GLIS1, POU1F1, SUFU, FOXH1, NPAS1, SORBS3, OVOL2, RHOX2A, HOXA7, OVOL1, PER3, NKX2-5, BAZ2A, HNRNPAB, DMBX1, TBX3, IKZF1, ZFP57, SUV39H1, DLX2, HOXB4, DLX1
GO:0060348 bone development	27	1,39	1,62E-04	FGFR2, TWSG1, FGF18, NOG, PDLIM7, HOXA11, PTH1R, ACP5, COL2A1, GLI2, SHH, AHSG, TNFRSF11A, BCL2, MYOG, PAPSS2, SATB2, RARG, BMP1, IGF2, MMP14, GM12617, PTHLH, HOXB4, TNFSF11, UBC, BMP7, CHRD

Biological process	*Gene Count*	*Percentage*	*p-Value*	*Genes repressed in RasGrf1 KO pancreatic islets (from Additional file 1: Table S1)*
GO:0042475 odontogenesis of dentine-containing tooth	14	0,72	1,72E-04	LEF1, DLL1, EDAR, GLI2, SHH, DLX2, DLX1, AMELX, TRP63, PDGFRA, EDA, BMP7, FGF4, NKX2-3
GO:0051050 positive regulation of transport	29	1,49	1,84E-04	ORAI1, TNF, GRIK1, DRD2, ADORA2A, C3, GHRHR, SHH, AHSG, SORBS1, IFNG, PTX3, NKX2-5, LTB, LTA, PSMD9, IGH-6, EDAR, HOMER1, FCGR1, SYNGR3, THY1, IGHG, FCGR2B, P2RX1, GHRH, CARTPT, EDA, HTR2C
GO:0051253 negative regulation of RNA metabolic process	54	2,78	1,88E-04	HMX1, FOXK1, PRRX1, CBX3, CBX2, MAF1, GLI2, SHH, KCNIP3, BARX2, PROP1, NKX6-2, TRP63, HSF4, NR2F2, CIITA, TBL1XR1, SATB2, RARG, SOX14, FOXJ1, SLA2, LEF1, TLE4, VAX1, NR0B1, NRIP2, SUZ12, HES3, ZFPM2, MTDH, TNF, GLIS1, POU1F1, SUFU, FOXH1, NPAS1, SORBS3, OVOL2, RHOX2A, HOXA7, OVOL1, PER3, NKX2-5, BAZ2A, HNRNPAB, DMBX1, TBX3, IKZF1, ZFP57, SUV39H1, DLX2, HOXB4, DLX1
GO:0007398 ectoderm development	29	1,49	2,11E-04	LOR, GPRC5D, ELF5, ZBTB17, SUFU, SHH, ACVR1B, BARX2, LHX1, SPRR2D, BCL2, POU2F3, OVOL1, GAB1, TRP63, TNFRSF19, LTB, DHCR24, EGFR, FOXN1, KRTDAP, EDAR, COL5A1, SPRR1B, MADCAM1, NGFR, EDA, TCF15, KRT71
GO:0030001 metal ion transport	71	3,66	2,17E-04	SLC8A3, SLC5A5, KCNC4, KCNC3, CACHD1, SCN3A, SLC20A2, TRPV2, KCNIP2, KCNIP1, KCNIP3, KCNK7, CHRNA4, TRPV4, KCNQ2, KCND2, KCND1, ATP4B, CACNG8, CACNG7, CACNG6, GIF, CACNG2, TMEM38B, BSPRY, CATSPER2, KCNH3, FXYD2, ORAI1, KCNA1, CACNB1, SLC38A10, KCNA6, ATP12A, 1300017J02RIK, KCNA7, KCNMB1, TPCN1, BC021785, KCNJ1, TPCN2, TMEM37, SLC5A4B, KCNS1, SLC4A8, KCNE1, KCNE2, SCN5A, TRPC2, HCN2, SLC8A2, SLC12A1, SLC12A3, SLC12A5, ATP1A2, CACNA1S, CACNA2D2, KCNK4, KCNJ5, KCNJ4, KCNJ6, KCNJ9, KCNN1, KCNN3, SLC5A9, CACNA1F, KCTD14, SCARA5, ACCN5, SCN4A, SLC5A11
GO:0007596 blood coagulation	19	0,98	2,18E-04	F12, F10, PLEK, MST1, SHH, PROC, GP9, FGG, ANXA8, PROCR, P2RX1, FGA, CD40LG, KLKB1, F2, SERPINC1, APOH, ENTPD1, PAPSS2
GO:0007498 mesoderm development	17	0,88	2,49E-04	FGFR2, TWSG1, TBX3, WNT3A, EOMES, DLL3, LEF1, NR4A3, EPHA2, FOXH1, ACVR1B, FOXF1A, OVOL1, TRP63, BMP7, CHRD, TCF15

Biological process	*Gene Count*	*Percentage*	*p-Value*	*Genes repressed in RasGrf1 KO pancreatic islets (from Additional file 1: Table S1)*
GO:0000904 cell morphogenesis involved in differentiation	40	2,06	2,88E-04	NOG, CDK5R1, CCK, DRD2, WNT3A, ERBB2, PIP5K1C, GLI2, SHH, ATOH1, SLC1A3, BCL2, BCL11B, LHX3, POU4F3, SEMA3A, DCLK1, CDH23, HNRNPAB, COL18A1, CRYAA, PLA2G10, ESRRB, EOMES, NTNG2, LEF1, VAX1, LMX1A, MYH9, NTN1, SLIT1, SEMA6A, SLITRK1, EPHA4, EPHA7, SEMA6C, NGFR, CACNA1F, BMPR1B, BMP7
GO:0000902 cell morphogenesis	53	2,73	3,20E-04	NOG, WNT3A, PIP5K1C, GLI2, SHH, TTLL3, ATOH1, SLC1A3, POU4F3, SEMA3A, LRRC50, CDH23, EGFR, CD3G, CRYAA, FOXJ1, PDPN, EOMES, LEF1, VAX1, MYH9, SLIT1, SLITRK1, NGFR, KLHL10, CDK5R1, CCK, ADORA2A, DRD2, ERBB2, BCL11B, BCL2, LHX3, CLASP2, DCLK1, HNRNPAB, COL18A1, KLK8, PLA2G10, ESRRB, NTNG2, CRB3, LMX1A, GAS7, NTN1, SEMA6A, EPHA4, FOXF1A, EPHA7, SEMA6C, CACNA1F, BMP7, BMPR1B
GO:0051172 negative regulation of nitrogen compound metabolic process	65	3,35	3,24E-04	HMX1, FOXK1, HR, STRM, PRRX1, CBX3, CBX2, GLI2, MAF1, SHH, KCNIP3, HTR1B, BARX2, PROP1, NKX6-2, TRP63, HSF4, NR2F2, CIITA, TBL1XR1, SATB2, RARG, SOX14, FOXJ1, SLA2, SIX3, LEF1, TLE4, VAX1, NR0B1, NRIP2, MXD3, MXD4, SUZ12, HES3, ZFPM2, C1D, MTDH, TNF, GLIS1, POU1F1, SUFU, FOXH1, NPAS1, SORBS3, OVOL2, RHOX2A, HOXA7, OVOL1, PER3, BAZ2A, NKX2-5, HNRNPAB, DMBX1, INSM2, IKZF1, TBX3, ZFP57, SUV39H1, FZD1, HOXB4, DLX2, DLX1, NR1I2, HTR2C
GO:0048705 skeletal system morphogenesis	28	1,44	3,41E-04	FGFR2, FGF18, HOXA11, PRRX1, ACP5, COL2A1, PRRX2, BARX2, HOXA4, HOXA5, HOXA7, NKX3-2, COL11A2, COL11A1, FGF4, RECQL4, SATB2, RARG, SIX4, PTHLH, DLX2, HOXB4, HOXB5, HOXB6, PDGFRA, PDGFRB, BMPR1B, TCF15
GO:0009967 positive regulation of signal transduction	34	1,75	3,77E-04	FGFR2, TWSG1, FGF18, FGFR3, TNF, DRD3, CSF1, PRRX1, SOX4, TLR4, PRRX2, ITSN1, TLR6, MAP3K7, SORBS3, SOS1, IFNG, GAB1, IL1B, PRL, LTB, FGFBP1, CD27, LTA, FGF23, IGH-6, FGF21, NFAM1, PDE6H, PDE6G, TRADD, NCAM1, TNFSF11, EDA
GO:0042472 inner ear morphogenesis	18	0,93	3,84E-04	FGFR2, WNT3A, OTX2, PRRX1, SOBP, COL2A1, DLL1, PRRX2, NR4A3, SIX4, FZD6, HESX1, ATOH1, MYO15, POU4F3, FOXI1, COL11A1, CDH23

Biological process	*Gene Count*	*Percentage*	*p-Value*	*Genes repressed in RasGrf1 KO pancreatic islets (from Additional file 1: Table S1)*
GO:0048565 gut development	13	0,67	4,16E-04	EGFR, SOX10, FOXL1, NKX2-6, GLI2, TCF7L2, SHH, EDNRB, FOXF1A, OVOL2, BCL2, GATA4, NKX2-3
GO:0060284 regulation of cell development	32	1,65	4,19E-04	IRX3, NOG, DRD3, DRD2, TNFRSF12A, HOXA11, SOX5, GLI2, SHH, ATOH1, METRN, MUSK, INPP5J, BCL2, NKX6-2, SEMA3A, FGF2, KLK8, LBX1, TBX3, LIMK1, DLL1, VAX1, LMX1A, NTN1, THY1, DLX2, DLX1, HES3, BMP7, CIT, NGF
GO:0001894 tissue homeostasis	15	0,77	4,19E-04	XDH, IL7, STK11, CSF1, PTH1R, ACP5, COL2A1, PTHLH, COL9A1, TNFSF11, BCL2, SFTPD, PDGFRB, KLHL10, COL11A2
GO:0045934 negative regulation of nucleobase, nucleoside, nucleotide and nucleic acid metabolic process	64	3,30	4,21E-04	HMX1, FOXK1, HR, STRM, PRRX1, CBX3, CBX2, GLI2, MAF1, SHH, KCNIP3, HTR1B, BARX2, PROP1, NKX6-2, TRP63, HSF4, NR2F2, CIITA, TBL1XR1, SATB2, RARG, SOX14, FOXJ1, SLA2, SIX3, LEF1, TLE4, VAX1, NR0B1, NRIP2, MXD3, MXD4, SUZ12, HES3, ZFPM2, C1D, MTDH, TNF, GLIS1, POU1F1, SUFU, FOXH1, NPAS1, SORBS3, OVOL2, RHOX2A, HOXA7, OVOL1, PER3, NKX2-5, BAZ2A, HNRNPAB, DMBX1, INSM2, IKZF1, TBX3, ZFP57, SUV39H1, FZD1, HOXB4, DLX2, DLX1, NR1I2
GO:0009966 regulation of signal transduction	97	4,99	4,26E-04	FGF18, NOG, IL6ST, HMGCR, CSPG4, TLR4, TLR6, ITSN1, GHRHR, IQGAP1, MAP3K7, IFNG, GAB1, IL1B, MLST8, SIX3, SOCS7, FGF23, FGF21, THY1, NCAM1, RASGRF2, IGBP1, TRP73, EDA, FGFR2, TWSG1, FGFR3, DRD3, DRD2, STK11, RIC8, RABGAP1L, NFKBIA, SOX4, CDC37, AHSG, RASAL3, ARHGEF10L, PTPN6, MET, IGH-6, PDE6H, PDE6G, TRADD, TNFSF11, RGS1, ADAP2, RGS3, ADRA1B, RGS6, BMP7, CHRD, GH, SH3RF1, PRRX1, PRDX2, PRRX2, GLI2, BARX1, SOX17, LTB, PRL, LTA, CEACAM1, PRKCA, FOXJ1, SLA2, LEF1, ESR2, NFAM1, PRKCD, ARHGEF10, SIGIRR, NGFR, CER1, TNF, CSF1, 4930403L05RIK, POU1F1, SUFU, SORBS3, SOS1, PLEKHG6, RASGRP1, RASGRP2, INPP5D, DDIT4L, TBC1D1, FGFBP1, CD27, FOXL1, RGS14, DKK4, RGS20, DUSP9, IGFBP2
GO:0048729 tissue morphogenesis	43	2,21	4,30E-04	FGFR2, TWSG1, NOG, FGFR3, WNT3A, CSF1, HOXA11, GLI2, SHH, SUFU, PGR, MAP3K7, OVOL2, HOXA5, BCL2, TRP63, SEMA3A, COL11A1, NKX2-3, EGFR, RARG, TBX3, TBX5, EOMES, ESR1, CRB3, LEF1, ESR2, NR4A3, EPHA2, FZD6, PTHLH, DDR1, HOXB4, FOXF1A, ADM, SFRP1, NGFR, BMP7, TBX18, CHRD, KRT71, MED1

Biological process	*Gene Count*	*Percentage*	*p-Value*	*Genes repressed in RasGrf1 KO pancreatic islets (from Additional file 1: Table S1)*
GO:0050767 regulation of neurogenesis	28	1,44	4,41E-04	IRX3, NOG, DRD3, DRD2, TNFRSF12A, SOX5, GLI2, SHH, ATOH1, METRN, INPP5J, NKX6-2, BCL2, SEMA3A, KLK8, LBX1, LIMK1, DLL1, VAX1, LMX1A, NTN1, THY1, DLX2, DLX1, HES3, CIT, BMP7, NGF
GO:0021983 pituitary gland development	11	0,57	4,53E-04	NOG, PROP1, GHRH, DRD2, LHX3, POU1F1, GLI2, TBX19, GHRHR, PITX1, GLI1
GO:0042476 odontogenesis	14	0,72	4,80E-04	LEF1, DLL1, EDAR, GLI2, SHH, DLX2, DLX1, AMELX, TRP63, PDGFRA, EDA, BMP7, FGF4, NKX2-3
GO:0048666 neuron development	50	2,57	5,02E-04	GPRIN1, WNT3A, PIP5K1C, GLI2, SHH, KLHL1, ATOH1, SLC1A3, ATG7, POU4F3, SEMA3A, PITX3, CDH23, VAX1, SLIT1, THY1, GABRR2, SLITRK1, NGFR, TRP73, CDK5R1, IRX5, CCK, DRD2, ADORA2A, ERBB2, CRB1, RAC3, BCL11B, BCL2, LHX3, RUNX1, DCLK1, GNAT1, KLK8, PLA2G10, NTNG2, LMX1A, GAS7, NTN1, SALL3, SEMA6A, EPHA4, EPHA7, SEMA6C, PHGDH, CACNA1F, BMPR1B, BMP7, CIT
GO:0031324 negative regulation of cellular metabolic process	75	3,86	5,85E-04	HMX1, FOXK1, HR, STRM, PRRX1, CBX3, PRDX2, CBX2, GLI2, MAF1, SHH, KCNIP3, HTR1B, BARX2, PROP1, NKX6-2, TRP63, HSF4, NR2F2, PRKCA, CIITA, TBL1XR1, SATB2, RARG, SOX14, FOXJ1, SLA2, SIX3, LEF1, TLE4, VAX1, NR0B1, PRKCD, MXD3, NRIP2, MXD4, SIGIRR, SUZ12, HES3, ZFPM2, C1D, WFIKKN1, MTDH, TNF, GLIS1, POU1F1, SUFU, FOXH1, NPAS1, SORBS3, INPP5K, OVOL2, INPP5J, RHOX2A, HOXA7, OVOL1, INPP5D, PER3, BAZ2A, NKX2-5, HNRNPAB, DMBX1, INSM2, PTPN6, IKZF1, TBX3, ZFP57, SUV39H1, FZD1, HOXB4, DLX2, DLX1, NR1I2, ADRA1B, HTR2C
GO:0010740 positive regulation of protein kinase cascade	21	1,08	5,86E-04	FGFR2, FGFR3, TNF, FGF23, IGH-6, TLR4, FGF21, ITSN1, TLR6, TRADD, MAP3K7, SORBS3, TNFSF11, IFNG, GAB1, IL1B, EDA, LTB, PRL, LTA, CD27
GO:0042471 ear morphogenesis	19	0,98	6,44E-04	FGFR2, WNT3A, OTX2, PRRX1, SOBP, COL2A1, DLL1, PRRX2, NR4A3, SIX4, FZD6, HESX1, ATOH1, MYO15, POU4F3, NKX3-2, FOXI1, COL11A1, CDH23

Biological process	*Gene Count*	*Percentage*	*p-Value*	*Genes repressed in RasGrf1 KO pancreatic islets (from Additional file 1: Table S1)*
GO:0034754 cellular hormone metabolic process	15	0,77	6,49E-04	HSD17B1, CYP11B2, CRABP2, ESR1, CYP26A1, SHH, AFP, CYP17A1, APOA1, SERPINA6, AKR1C18, LCN5, SRD5A2, BMPR1B, CYP19A1
GO:0015672 monovalent inorganic cation transport	51	2,63	6,56E-04	KCNC4, SLC5A5, KCNC3, SCN3A, SLC20A2, KCNIP2, KCNIP1, KCNIP3, ANK1, KCNK7, KCNQ2, KCND2, KCND1, ATP4B, TMEM38B, KCNH3, FXYD2, KCNA1, SLC38A10, KCNA6, ATP6V1B2, ATP12A, KCNA7, KCNMB1, KCNJ1, BC021785, SLC5A4B, KCNS1, KCNE1, SLC4A8, KCNE2, SCN5A, HCN2, SLC12A1, SLC12A3, SLC12A5, ATP1A2, KCNK4, KCNJ5, KCNJ4, KCNJ6, KCNJ9, KCNN1, KCNN3, ATP6V1E2, SLC5A9, ATP6V0A4, ACCN5, KCTD14, SLC5A11, SCN4A
GO:0019219 regulation of nucleobase, nucleoside, nucleotide and nucleic acid metabolic process	290	14,93	6,70E-04	ADCY3, ADCY4, MEF2B, STRM, HIRA, RORA, CITED4, SHH, ZFP92, PGR, HTR1B, MED29, NKX6-2, IFNG, TIGD5, PITX3, PITX1, PHOX2A, WNT10B, RARG, RCOR3, NKX2-6, BARHL1, RXRG, EOMES, CD40, HNF4G, MECOM, CPHX, PRDM9, BAZ1B, ABT1, PARP14, HES3, MED17, HES2, CDCA7L, VGLL4, ACR, HOXA11, PTH1R, NFKBIA, SFPI1, ADCYAP1, FOXH1, HESX1, TAL2, MUSK, LHX1, LHX3, LHX5, NKX2-4, NKX2-5, NKX2-3, HNRNPAB, TCF23, DMBX1, ZBTB48, ZBTB46, KLF9, IKZF1, KLF13, ESRRB, ESRRG, PMF1, DDX5, ABCG4, ZFP444, USF1, DLX3, DLX2, DLX1, FOXF1A, NR1I2, SUPT16H, BMP7, FOXI1, KLF1, TCF15, BACH2, EVX1, ELF3, ELF5, NFKB2, GLI2, KCNIP3, GLI1, GUCY1A3, SPIC, HSF4, NR2F2, TOP2A, FGF2, CSDC2, BATF2, PTF1A, GTF2H4, TLE4, NFAM1, MXD3, SIGIRR, MYCN, JMY, MXD4, BRDT, RUVBL2, KCNH3, TBX18, TBX19, MED1, MTDH, ZFP64, ZBTB17, SEC14L2, SUFU, ZFP111, MYCL1, ZFP36L2, STAT4, SORBS3, BCL11B, ETV2, MLLT1, PER3, TCFEB, BAZ2A, TBX3, ZFP57, TBX5, FZD1, LMX1A, ZFP707, RHOX9, PHF19, JMJD6, NR5A2, HMX1, IL16, CBX3, CBX2, TLR4, TBP, FOXO3, HOXD1, ANKRD1, MAF1, CBX7, GHRHR, IGHMBP2, HOXC6, BATF, ATOH1, PROP1, GATA5, GATA4, TRP63, ZFP503, ATOH7, CIITA, MAGEL2, SATB2, LBX1, TCFAP2C, RELB, SIX3, FOXN1, FGF23, SIX4, VAX1, NR0B1, GRHL2, SIX6, SUZ12, PTHLH, ASCL2, GTF2IRD1, SPDEF, MGA, TRP73, NFE2L3, ASCL3, TSHZ2, DRD3, ADORA2A, DRD2, ONECUT3, DRD4, SOX5, SOX4, ELK1, SRY, OVOL2, HOXA4, ELK4, HOXA5, HOXA7, OVOL1, NKX3-2, RUNX1, NFE2, SUV39H1, TEAD2, TEAD3, NR4A3, ESX1, ATF7IP2, SALL3, HOXB4, HILS1, SEBOX, HOXB5, SMARCC1, HOXB6, RFX1, POU6F1, AEBP1, SOX21, FOXK1, HR, PRRX1, PRDX2, PRRX2, CBFA2T3, NFATC2IP, BARX1, BARX2, LBH, PAX9, PRMT7, POU4F3, SOX17, PSMD9, SOX10, TBL1XR1, NLRP5, EGR3, ZFP553, KHDRBS2, SOX14, FOXJ1, SLA2, OTX2, ESR1, LEF1, ESR2, NRIP2, EYA3, PRKCQ, AYM1, GHRH, ZFPM2, C1D, IRX4, CALCR, IRX3, IRX5, TNF, FOXM1, GLIS1, NFYA, POU1F1, TCF7L2, ZFP493, DPF1, NPAS1, TNFRSF1A, TNFRSF1B, NPAS2, ZKSCAN14, REL, HRH3, POU2F3, POU2F2, RHOX2A, MYCBP, MYOG, NFATC4, NFATC2, ERF, INSM2, FOXL1, CREB3, SAP30BP, VSX2, ZFP286, HEYL, SETD7, MESP2, OPRD1, NFIB
GO:0009968 negative regulation of signal transduction	33	1,70	7,19E-04	CER1, TWSG1, NOG, DRD3, DRD2, NFKBIA, PRDX2, SUFU, AHSG, BARX1, IL1B, INPP5D, DDIT4L, SOX17, PRKCA, PTPN6, SLA2, SIX3, SOCS7, ESR2, PRKCD, RGS14, DKK4, SIGIRR, THY1, RGS20, RGS1, RGS3, RGS6, ADRA1B, NGFR, BMP7, CHRD

Biological process	*Gene Count*	*Percentage*	*p-Value*	*Genes repressed in RasGrf1 KO pancreatic islets (from Additional file 1: Table S1)*
GO:0015698<br>inorganic anion transport	20	1,03	7,24E-04	SLC5A5, GABRA2, GABRA1, SLC12A1, SLC20A2, SLC12A3, GLRA3, SLC12A5, CLCNKA, ASZ1, CLCNKB, GABRR2, TST, SLC26A3, SLC26A6, P2RY4, SLC4A1, CLCN6, CLCN7, MPST
GO:0030855<br>epithelial cell differentiation	26	1,34	7,79E-04	FGFR2, LOR, GPRC5D, ELF3, ELF5, GJA1, PGR, AGPAT6, BARX1, SPRR2D, POU2F3, TRP63, KCNE1, FGF2, COL18A1, FOXN1, ESR1, FZD1, CRB3, ESR2, PTHLH, FOXF1A, HOXB5, SPRR1B, MADCAM1, BMP7
GO:0048547<br>gut morphogenesis	10	0,51	8,45E-04	EGFR, SOX10, FOXF1A, OVOL2, BCL2, GATA4, GLI2, TCF7L2, SHH, NKX2-3
GO:0008285<br>negative regulation of cell proliferation	40	2,06	8,86E-04	CER1, NOG, TNF, FGFR3, ADORA2A, DRD2, STK11, ERBB2, PTH1R, ASZ1, PMAIP1, SHH, OVOL2, PTGES, BCL2, BCL11B, TFF1, INPP5D, FGF2, LTB, LTA, PRKCA, PTPN6, RARG, LBX1, FOXJ1, TBX5, TNFRSF14, ESR2, VAX1, IDO1, VSX2, DDR1, CDKN1A, FCGR2B, PLA2G2A, BMP7, EIF2AK2, AY074887, NFIB
GO:0043583<br>ear development	23	1,18	9,03E-04	FGFR2, FGFR3, WNT3A, OTX2, PRRX1, SOBP, COL2A1, DLL1, SIX4, PRRX2, NR4A3, FZD6, HESX1, DDR1, ATOH1, BCL2, MYO15, POU4F3, NKX3-2, FOXI1, COL11A1, TCF15, CDH23
GO:0019932<br>second-messenger-mediated signaling	25	1,29	9,73E-04	CALCR, ADCY4, CASR, GNA15, TNF, DRD3, DRD2, ADORA2A, PTH1R, DRD4, GNG13, GHRHR, NOS2, LTB, HTR5A, LTA, GNAT1, NTSR2, PTHLH, ADAP2, CHRM3, GHRH, HTR2B, HTR2C, OPRD1
GO:0008544<br>epidermis development	26	1,34	9,95E-04	LOR, GPRC5D, SUFU, SHH, ACVR1B, BARX2, SPRR2D, BCL2, POU2F3, OVOL1, GAB1, TRP63, TNFRSF19, LTB, DHCR24, EGFR, FOXN1, KRTDAP, EDAR, COL5A1, SPRR1B, MADCAM1, NGFR, EDA, KRT71, TCF15

Biological process	***Gene Count***	***Percentage***	***p-Value***	***Genes repressed in RasGrf1 KO pancreatic islets (from Additional file 1: Table S1)***
GO:0010558<br>negative regulation of macromolecule biosynthetic process	65	3,35	9,95E-04	HMX1, FOXK1, HR, STRM, PRRX1, CBX3, CBX2, GLI2, MAF1, SHH, KCNIP3, BARX2, PROP1, NKX6-2, TRP63, HSF4, NR2F2, CIITA, TBL1XR1, SATB2, RARG, SOX14, FOXJ1, SLA2, SIX3, LEF1, TLE4, VAX1, NR0B1, NRIP2, MXD3, MXD4, SIGIRR, SUZ12, HES3, ZFPM2, C1D, MTDH, TNF, GLIS1, POU1F1, SUFU, FOXH1, NPAS1, SORBS3, OVOL2, RHOX2A, HOXA7, OVOL1, INPP5D, PER3, BAZ2A, NKX2-5, HNRNPAB, DMBX1, INSM2, IKZF1, TBX3, ZFP57, SUV39H1, FZD1, HOXB4, DLX2, DLX1, NR1I2
GO:0031175<br>neuron projection development	39	2,01	1,00E-03	GPRIN1, CDK5R1, CCK, ADORA2A, DRD2, WNT3A, ERBB2, PIP5K1C, GLI2, SHH, KLHL1, ATOH1, RAC3, ATG7, BCL2, BCL11B, LHX3, POU4F3, SEMA3A, DCLK1, KLK8, PLA2G10, NTNG2, VAX1, LMX1A, NTN1, GAS7, SLIT1, SEMA6A, SLITRK1, EPHA4, EPHA7, SEMA6C, PHGDH, CACNA1F, NGFR, BMPR1B, BMP7, CIT
GO:0031326<br>regulation of cellular biosynthetic process	301	15,50	1,02E-03	ADCY3, ADCY4, MEF2B, STRM, HIRA, RORA, CITED4, SHH, ZFP92, PGR, HTR1B, MED29, NKX6-2, IFNG, TIGD5, PITX3, PITX1, PHOX2A, WNT10B, RARG, RCOR3, NKX2-6, BARHL1, EOMES, RXRG, HNF4G, MECOM, CPHX, PRDM9, BAZ1B, ABT1, PARP14, HES3, MED17, HES2, CDCA7L, VGLL4, ACR, HOXA11, PTH1R, IGF2BP1, NFKBIA, SFPI1, ADCYAP1, FOXH1, HESX1, TAL2, MUSK, LHX1, LHX3, LHX5, NKX2-4, NKX2-5, NKX2-3, HNRNPAB, TCF23, DMBX1, ZBTB48, ZBTB46, KLF9, IKZF1, KLF13, ESRRB, ESRRG, PMF1, DDX5, ABCG4, ZFP444, USF1, DLX3, DLX2, DLX1, FOXF1A, NR1I2, SUPT16H, BMP7, FOXI1, KLF1, TCF15, BACH2, EVX1, ELF3, ELF5, NFKB2, GLI2, GLI1, KCNIP3, GUCY1A3, SPIC, HSF4, NR2F2, TOP2A, FGF2, CSDC2, BATF2, PTF1A, GTF2H4, TLE4, NFAM1, MXD3, SIGIRR, MYCN, JMY, MXD4, BRDT, RUVBL2, KCNH3, TBX18, TBX19, MED1, MTDH, ZFP64, ZBTB17, SEC14L2, SUFU, ZFP111, MYCL1, STAT4, SORBS3, SORBS1, BCL11B, BCL2, ETV2, MLLT1, PER3, TCFEB, PTX3, BAZ2A, CD27, TBX3, ZFP57, TBX5, FZD1, LMX1A, ZFP707, RHOX9, PHF19, JMJD6, NR5A2, HMX1, IL16, CBX3, CBX2, TLR4, TBP, FOXO3, HOXD1, ANKRD1, TLR6, MAF1, TLR7, CBX7, GHRHR, TLR9, IGHMBP2, HOXC6, BATF, ATOH1, PROP1, GATA5, GATA4, TRP63, IL1B, ZFP503, ATOH7, CIITA, MAGEL2, SATB2, LBX1, TCFAP2C, RELB, SIX3, FOXN1, FGF23, SIX4, VAX1, NR0B1, IL21, GRHL2, SIX6, SUZ12, PTHLH, ASCL2, GTF2IRD1, SPDEF, MGA, TRP73, NFE2L3, ASCL3, TSHZ2, DRD3, ADORA2A, DRD2, ONECUT3, DRD4, SOX5, SOX4, ELK1, SRY, OVOL2, HOXA4, ELK4, HOXA5, HOXA7, OVOL1, NKX3-2, ADAM33, RUNX1, NFE2, SUV39H1, TEAD2, TEAD3, NR4A3, ESX1, ATF7IP2, SALL3, HOXB4, HILS1, SEBOX, HOXB5, SMARCC1, HOXB6, RFX1, EIF4E2, POU6F1, AEBP1, SOX21, FOXK1, HR, PRRX1, PRDX2, PRRX2, CBFA2T3, NFATC2IP, BARX1, BARX2, LBH, PAX9, PRMT7, POU4F3, SOX17, LTB, PSMD9, SOX10, TBL1XR1, EGR3, ZFP553, KHDRBS2, SOX14, FOXJ1, SLA2, OTX2, ESR1, LEF1, ESR2, NRIP2, EYA3, EIF4G2, PRKCQ, AYM1, GHRH, ZFPM2, C1D, IRX4, CALCR, IRX3, IRX5, TNF, FOXM1, GLIS1, NFYA, POU1F1, TCF7L2, ZFP493, DPF1, TNFRSF1A, NPAS1, NPAS2, ZKSCAN14, REL, HRH3, POU2F3, POU2F2, RHOX2A, MYCBP, MYOG, NFATC4, INPP5D, NFATC2, ERF, INSM2, FOXL1, CREB3, SAP30BP, VSX2, ZFP286, HEYL, SETD7, MESP2, OPRD1, NFIB
GO:0021940<br>positive regulation of granule cell precursor proliferation	6	0,31	1,07E-03	LHX1, LHX5, GLI2, FGF2, SHH, GLI1

Biological process	*Gene Count*	*Percentage*	*p-Value*	*Genes repressed in RasGrf1 KO pancreatic islets (from Additional file 1: Table S1)*
GO:0021936 regulation of granule cell precursor proliferation	6	0,31	1,07E-03	LHX1, LHX5, GLI2, FGF2, SHH, GLI1
GO:0045597 positive regulation of cell differentiation	33	1,70	1,08E-03	FGF18, IRX3, MSR1, FGFR3, DRD2, TNFRSF12A, HOXA11, CSF1, FOXO3, GLI2, SHH, ATOH1, METRN, NKX6-2, BCL2, GATA4, HSF4, INPP5D, RUNX1, FGF2, NKX2-5, CD27, IKZF1, IL7, LIMK1, TBX5, EOMES, IL21, NTN1, WNT7B, TNFSF11, BMP7, NGF
GO:0051971 positive regulation of transmission of nerve impulse	9	0,46	1,14E-03	LAMA2, TNF, SLC1A3, GRIK1, ADORA2A, NCS1, CARTPT, LTB, LTA
GO:0007517 muscle organ development	33	1,70	1,19E-03	MTSS1, FOXK1, ERBB2, ELN, TAGLN3, SHH, MUSK, ATG7, GATA4, TRP63, MYOG, NR2F2, NKX2-5, COL11A1, JPH1, PITX1, TCF23, LBX1, NKX2-6, TBX5, MET, RXRG, CACNG2, SIX4, CD164, HOMER1, CACNA1S, CSRP3, CACNA2D2, PDGFRB, ZFPM2, TCF15, SNTA1
GO:0048812 neuron projection morphogenesis	33	1,70	1,19E-03	CDK5R1, CCK, ADORA2A, DRD2, WNT3A, ERBB2, PIP5K1C, GLI2, SHH, ATOH1, BCL2, BCL11B, LHX3, POU4F3, SEMA3A, DCLK1, KLK8, PLA2G10, NTNG2, VAX1, LMX1A, GAS7, NTN1, SLIT1, SEMA6A, SLITRK1, EPHA4, EPHA7, SEMA6C, CACNA1F, NGFR, BMPR1B, BMP7
GO:0050768 negative regulation of neurogenesis	12	0,62	1,27E-03	DLX2, KLK8, DLX1, NOG, DRD3, NKX6-2, SEMA3A, VAX1, BMP7, CIT, NTN1, THY1

Biological process	*Gene Count*	*Percentage*	*p-Value*	*Genes repressed in RasGrf1 KO pancreatic islets (from Additional file 1: Table S1)*
GO:0031327 negative regulation of cellular biosynthetic process	66	3,40	1,28E-03	HMX1, FOXK1, HR, STRM, PRRX1, CBX3, CBX2, GLI2, MAF1, SHH, KCNIP3, HTR1B, BARX2, PROP1, NKX6-2, TRP63, HSF4, NR2F2, CIITA, TBL1XR1, SATB2, RARG, SOX14, FOXJ1, SLA2, SIX3, LEF1, TLE4, VAX1, NR0B1, NRIP2, MXD3, MXD4, SIGIRR, SUZ12, HES3, ZFPM2, C1D, MTDH, TNF, GLIS1, POU1F1, SUFU, FOXH1, NPAS1, SORBS3, OVOL2, RHOX2A, HOXA7, OVOL1, INPP5D, PER3, BAZ2A, NKX2-5, HNRNPAB, DMBX1, INSM2, IKZF1, TBX3, ZFP57, SUV39H1, FZD1, HOXB4, DLX2, DLX1, NR1I2
GO:0001890 placenta development	20	1,03	1,32E-03	EGFR, FGFR2, ESRRB, PLAC1, MET, CCNF, EOMES, PCDH12, LEF1, GJB5, ESX1, ITGA4, DLX3, WNT7B, ADM, OVOL2, GAB1, EPOR, TCFEB, MED1
GO:0060443 mammary gland morphogenesis	11	0,57	1,42E-03	PGR, PTHLH, FGFR2, DDR1, NRG3, TBX3, ELF3, CSF1, ESR1, GLI2, MED1
GO:0002526 acute inflammatory response	19	0,98	1,42E-03	C4B, C3, EPHX2, SAA4, PRDX2, TLR4, IDO1, FCGR1, SIGIRR, AHSG, IGHG, C4BP, P2RX1, SAA2, SERPINF2, F2, IL1B, C2, CFD
GO:0021937 Purkinje cell-granule cell precursor cell signaling involved in regulation of granule cell precursor cell proliferation	5	0,26	1,48E-03	LHX1, LHX5, GLI2, SHH, GLI1

Biological process	*Gene Count*	*Percentage*	*p-Value*	*Genes repressed in RasGrf1 KO pancreatic islets (from Additional file 1: Table S1)*
GO:0010648 negative regulation of cell communication	34	1,75	1,54E-03	CER1, TWSG1, NOG, GRIK1, DRD3, DRD2, NFKBIA, PRDX2, SUFU, AHSG, BARX1, IL1B, INPP5D, DDIT4L, SOX17, PRKCA, PTPN6, SLA2, SIX3, SOCS7, ESR2, PRKCD, RGS14, DKK4, SIGIRR, THY1, RGS20, RGS1, RGS3, RGS6, ADRA1B, NGFR, BMP7, CHRD
GO:0048806 genitalia development	9	0,46	1,58E-03	TBX3, TRP63, PDGFRA, KLHL10, SRD5A2, TCF7L2, SHH, GJB2, DHCR24
GO:0006812 cation transport	76	3,91	1,60E-03	SLC8A3, SLC5A5, KCNC4, KCNC3, CACHD1, SCN3A, SLC20A2, TRPV2, KCNIP2, KCNIP1, KCNIP3, ANK1, KCNK7, CHRNA4, TRPV4, KCNQ2, KCND2, KCND1, ATP4B, CACNG8, CACNG7, CACNG6, GIF, CACNG2, TMEM38B, BSPRY, CATSPER2, KCNH3, FXYD2, ORAI1, KCNA1, CACNB1, SLC38A10, KCNA6, ATP6V1B2, ATP12A, KCNA7, 1300017J02RIK, KCNMB1, TPCN1, BC021785, KCNJ1, TPCN2, TMEM37, SLC5A4B, KCNS1, SLC4A8, KCNE1, KCNE2, SCN5A, TRPC2, HCN2, SLC8A2, SLC12A1, SLC12A3, SLC12A5, ATP1A2, CACNA1S, CACNA2D2, KCNK4, KCNJ5, KCNJ4, KCNJ6, KCNJ9, KCNN1, KCNN3, NHEDC1, ATP6V1E2, SLC5A9, CACNA1F, ATP6V0A4, SCARA5, KCTD14, ACCN5, SCN4A, SLC5A11
GO:0009890 negative regulation of biosynthetic process	66	3,40	1,61E-03	HMX1, FOXK1, HR, STRM, PRRX1, CBX3, CBX2, GLI2, MAF1, SHH, KCNIP3, HTR1B, BARX2, PROP1, NKX6-2, TRP63, HSF4, NR2F2, CIITA, TBL1XR1, SATB2, RARG, SOX14, FOXJ1, SLA2, SIX3, LEF1, TLE4, VAX1, NR0B1, NRIP2, MXD3, MXD4, SIGIRR, SUZ12, HES3, ZFPM2, C1D, MTDH, TNF, GLIS1, POU1F1, SUFU, FOXH1, NPAS1, SORBS3, OVOL2, RHOX2A, HOXA7, OVOL1, INPP5D, PER3, BAZ2A, NKX2-5, HNRNPAB, DMBX1, INSM2, IKZF1, TBX3, ZFP57, SUV39H1, FZD1, HOXB4, DLX2, DLX1, NR1I2
GO:0010629 negative regulation of gene expression	63	3,24	1,61E-03	HMX1, FOXK1, HR, STRM, PRRX1, CBX3, CBX2, GLI2, MAF1, SHH, KCNIP3, BARX2, PROP1, NKX6-2, TRP63, HSF4, NR2F2, CIITA, TBL1XR1, SATB2, RARG, SOX14, FOXJ1, SLA2, SIX3, LEF1, TLE4, VAX1, NR0B1, NRIP2, MXD3, MXD4, SUZ12, HES3, ZFPM2, C1D, MTDH, TNF, GLIS1, POU1F1, SUFU, FOXH1, NPAS1, SORBS3, OVOL2, RHOX2A, HOXA7, OVOL1, PER3, NKX2-5, BAZ2A, HNRNPAB, DMBX1, INSM2, IKZF1, TBX3, ZFP57, SUV39H1, FZD1, HOXB4, DLX2, DLX1, NR1I2
GO:0048858 cell projection morphogenesis	36	1,85	1,73E-03	CDK5R1, CCK, ADORA2A, DRD2, WNT3A, ERBB2, PIP5K1C, GLI2, SHH, TTLL3, ATOH1, BCL2, BCL11B, LHX3, POU4F3, SEMA3A, DCLK1, LRRC50, KLK8, PLA2G10, FOXJ1, NTNG2, VAX1, LMX1A, GAS7, NTN1, SLIT1, SEMA6A, SLITRK1, EPHA4, EPHA7, SEMA6C, NGFR, CACNA1F, BMPR1B, BMP7

Biological process	*Gene Count*	*Percentage*	*p-Value*	*Genes repressed in RasGrf1 KO pancreatic islets (from Additional file 1: Table S1)*
GO:0002009 morphogenesis of an epithelium	32	1,65	1,79E-03	FGFR2, NOG, FGFR3, HOXA11, CSF1, GLI2, SUFU, SHH, PGR, MAP3K7, OVOL2, HOXA5, BCL2, TRP63, SEMA3A, NKX2-3, EGFR, RARG, TBX3, TBX5, ESR1, CRB3, ESR2, FZD6, PTHLH, DDR1, HOXB4, FOXF1A, ADM, SFRP1, TBX18, MED1
GO:0016477 cell migration	41	2,11	1,80E-03	PVR, CER1, CDK5R1, TNF, CCK, IL16, DRD2, TNFRSF12A, CCR1, S100A9, ASZ1, CSPG4, GJA1, SHH, EDNRB, ATOH1, APOA1, IL17B, OVOL2, GAB2, IFNG, IL1B, CLASP2, NR2F2, DCLK1, NKX2-3, PRKCA, EGFR, SATB2, FOXJ1, BARHL1, MET, ESR2, SIX4, ITGA4, VAX1, MYH9, MMP14, DBH, NTN1, LAMC1
GO:0030193 regulation of blood coagulation	7	0,36	1,81E-03	F12, PLEK, KLKB1, APOH, TLR4, PROC, ANXA2
GO:0045449 regulation of transcription	272	14,01	1,81E-03	MEF2B, STRM, HIRA, RORA, CITED4, SHH, ZFP92, PGR, MED29, NKX6-2, IFNG, TIGD5, PITX3, PITX1, PHOX2A, WNT10B, RARG, RCOR3, NKX2-6, BARHL1, RXRG, EOMES, HNF4G, MECOM, CPHX, PRDM9, BAZ1B, ABT1, PARP14, HES3, MED17, HES2, CDCA7L, VGLL4, HOXA11, NFKBIA, SFPI1, FOXH1, HESX1, TAL2, MUSK, LHX1, LHX3, LHX5, NKX2-4, NKX2-5, NKX2-3, HNRNPAB, TCF23, DMBX1, ZBTB48, ZBTB46, KLF9, IKZF1, KLF13, ESRRB, ESRRG, PMF1, DDX5, ABCG4, ZFP444, USF1, DLX3, DLX2, DLX1, FOXF1A, NR1I2, SUPT16H, BMP7, FOXI1, KLF1, TCF15, BACH2, EVX1, ELF3, ELF5, NFKB2, GLI2, KCNIP3, GLI1, SPIC, HSF4, NR2F2, TOP2A, FGF2, CSDC2, BATF2, PTF1A, GTF2H4, TLE4, NFAM1, MXD3, SIGIRR, MYCN, JMY, MXD4, BRDT, RUVBL2, KCNH3, TBX18, TBX19, MED1, MTDH, ZFP64, ZBTB17, SEC14L2, SUFU, ZFP111, MYCL1, STAT4, SORBS3, BCL11B, ETV2, MLLT1, PER3, TCFEB, BAZ2A, TBX3, ZFP57, TBX5, FZD1, LMX1A, ZFP707, RHOX9, PHF19, JMJD6, NR5A2, HMX1, IL16, CBX3, CBX2, TLR4, TBP, FOXO3, HOXD1, ANKRD1, MAF1, CBX7, IGHMBP2, HOXC6, BATF, ATOH1, PROP1, GATA5, GATA4, TRP63, ZFP503, ATOH7, CIITA, MAGEL2, SATB2, LBX1, TCFAP2C, RELB, SIX3, FOXN1, FGF23, SIX4, VAX1, NR0B1, GRHL2, SIX6, PTHLH, SUZ12, ASCL2, GTF2IRD1, SPDEF, MGA, TRP73, NFE2L3, ASCL3, TSHZ2, ADORA2A, ONECUT3, SOX5, SOX4, ELK1, SRY, OVOL2, HOXA4, ELK4, HOXA5, HOXA7, OVOL1, NKX3-2, RUNX1, NFE2, SUV39H1, TEAD2, TEAD3, NR4A3, ESX1, ATF7IP2, SALL3, HOXB4, HILS1, SEBOX, HOXB5, SMARCC1, HOXB6, RFX1, POU6F1, AEBP1, SOX21, FOXK1, HR, PRRX1, PRDX2, PRRX2, CBFA2T3, NFATC2IP, BARX1, BARX2, LBH, PAX9, PRMT7, POU4F3, SOX17, PSMD9, SOX10, TBL1XR1, EGR3, ZFP553, KHDRBS2, SOX14, FOXJ1, SLA2, OTX2, ESR1, LEF1, ESR2, NRIP2, EYA3, PRKCQ, AYM1, GHRH, ZFPM2, C1D, IRX4, IRX3, IRX5, TNF, FOXM1, GLIS1, NFYA, POU1F1, TCF7L2, ZFP493, DPF1, NPAS1, TNFRSF1A, NPAS2, ZKSCAN14, REL, POU2F3, POU2F2, RHOX2A, MYCBP, MYOG, NFATC4, NFATC2, ERF, INSM2, FOXL1, CREB3, SAP30BP, VSX2, ZFP286, HEYL, SETD7, MESP2, NFIB
GO:0030808 regulation of nucleotide biosynthetic process	17	0,88	1,88E-03	ADCY3, ACR, CALCR, ADCY4, DRD3, DRD2, ADORA2A, PTH1R, DRD4, GHRHR, ADCYAP1, PTHLH, HTR1B, HRH3, GHRH, GUCY1A3, OPRD1

Biological process	*Gene Count*	*Percentage*	*p-Value*	*Genes repressed in RasGrf1 KO pancreatic islets (from Additional file 1: Table S1)*
GO:0010721 negative regulation of cell development	12	0,62	2,00E-03	DLX2, KLK8, DLX1, NOG, DRD3, NKX6-2, SEMA3A, VAX1, BMP7, CIT, NTN1, THY1
GO:0009952 anterior/posterior pattern formation	29	1,49	2,12E-03	CER1, HOXA11, WNT3A, SHH, HOXC6, FOXH1, BARX1, HOXA4, HOXA5, LHX1, GATA4, HOXA7, NR2F2, RARG, TBX3, OTX2, SIX3, DLL3, LEF1, CYP26A1, DLL1, HOXB4, SFRP1, HES3, HOXB5, HOXB6, MESP2, TBX18, TCF15
GO:0045137 development of primary sexual characteristics	22	1,13	2,16E-03	DHH, TBX3, ESR1, FOXO3, ESR2, SPO11, NR0B1, MMP14, SHH, GJB2, AHSG, PGR, AFP, BCL2, TRP63, PDGFRA, BIK, HSD17B4, SRD5A2, KLHL10, BMPR1B, DHCR24
GO:0031646 positive regulation of neurological system process	9	0,46	2,16E-03	LAMA2, TNF, SLC1A3, GRIK1, ADORA2A, NCS1, CARTPT, LTB, LTA
GO:0006694 steroid biosynthetic process	17	0,88	2,20E-03	CYB5R3, MVD, HSD17B1, HMGCR, HSD3B4, CYP11B2, CYP21A1, AKR1C21, STARD3, CYP17A1, APOA1, SDR42E1, MVK, SRD5A2, BMPR1B, HSD17B7, DHCR24
GO:0048534 hemopoietic or lymphoid organ development	46	2,37	2,20E-03	CALCR, TWSG1, TNF, CCR1, WNT3A, RAG1, SOX4, SFPI1, PRDX2, NFKB2, TRIM10, CBFA2T3, POU1F1, TIMP1, BARX1, ANK1, TNFRSF11A, CLCF1, BCL2, BCL11B, POU2F2, NKX3-2, RUNX1, NKX2-5, LTB, FGF3, LTA, NKX2-3, IKZF1, IL7, MYO1E, RELB, EOMES, IL25, NFAM1, SIX4, MYH9, TACC3, HBA-A1, HOXB4, TNFSF11, CD40LG, JMJD6, KLF1, ADD2, MED1

Biological process	*Gene Count*	*Percentage*	*p-Value*	*Genes repressed in RasGrf1 KO pancreatic islets (from Additional file 1: Table S1)*
GO:0008217<br>regulation of blood pressure	16	0,82	2,33E-03	NOS1, DRD3, ACTA2, DRD2, CYP11B2, EPHX2, ATP1A2, TNNI3, AGTRAP, AQP2, EDNRB, ACE, ADRA1B, CARTPT, GUCY1A3, GUCA2B
GO:0046546<br>development of primary male sexual characteristics	14	0,72	2,52E-03	DHH, TBX3, ESR1, NR0B1, SHH, AHSG, GJB2, BCL2, PDGFRA, BIK, HSD17B4, SRD5A2, KLHL10, DHCR24
GO:0030539<br>male genitalia development	7	0,36	2,75E-03	TBX3, PDGFRA, KLHL10, SRD5A2, SHH, GJB2, DHCR24
GO:0007409<br>axonogenesis	30	1,54	2,76E-03	CDK5R1, CCK, DRD2, ERBB2, WNT3A, PIP5K1C, GLI2, SHH, ATOH1, BCL2, BCL11B, LHX3, POU4F3, SEMA3A, DCLK1, PLA2G10, NTNG2, VAX1, LMX1A, NTN1, SLIT1, SEMA6A, SLITRK1, EPHA4, EPHA7, SEMA6C, CACNA1F, NGFR, BMPR1B, BMP7
GO:0010556<br>regulation of macromolecule biosynthetic process	287	14,78	2,83E-03	MEF2B, S100A9, STRM, HIRA, RORA, CITED4, SHH, ZFP92, PGR, MED29, NKX6-2, IFNG, TIGD5, PITX3, PITX1, PHOX2A, WNT10B, RARG, RCOR3, NKX2-6, BARHL1, RXRG, EOMES, HNF4G, MECOM, CPHX, PRDM9, BAZ1B, ABT1, PARP14, HES3, MED17, HES2, CDCA7L, VGLL4, HOXA11, IGF2BP1, NFKBIA, SFPI1, FOXH1, HESX1, TAL2, MUSK, LHX1, LHX3, LHX5, NKX2-4, NKX2-5, NKX2-3, HNRNPAB, TCF23, DMBX1, ZBTB48, ZBTB46, KLF9, IKZF1, KLF13, ESRRB, ESRRG, PMF1, DDX5, ABCG4, ZFP444, USF1, DLX3, DLX2, DLX1, FOXF1A, NR1I2, SUPT16H, BMP7, FOXI1, KLF1, TCF15, BACH2, EVX1, ELF3, ELF5, NFKB2, GLI2, GLI1, KCNIP3, SPIC, HSF4, NR2F2, TOP2A, FGF2, CSDC2, BATF2, PTF1A, GTF2H4, TLE4, NFAM1, MXD3, SIGIRR, MYCN, JMY, MXD4, BRDT, RUVBL2, KCNH3, TBX18, TBX19, MED1, MTDH, ZFP64, ZBTB17, SEC14L2, SUFU, ZFP111, MYCL1, STAT4, SORBS3, SORBS1, BCL11B, BCL2, ETV2, MLLT1, PER3, TCFEB, BAZ2A, CD27, TBX3, ZFP57, TBX5, FZD1, LMX1A, ZFP707, RHOX9, PHF19, JMJD6, NR5A2, HMX1, IL16, CBX3, CBX2, TLR4, TBP, FOXO3, HOXD1, ANKRD1, TLR6, MAF1, TLR7, CBX7, TLR9, IGHMBP2, HOXC6, BATF, ATOH1, PROP1, GATA5, GATA4, TRP63, IL1B, ZFP503, ATOH7, CIITA, MAGEL2, SATB2, LBX1, TCFAP2C, RELB, SIX3, FOXN1, FGF23, SIX4, VAX1, NR0B1, IL21, GRHL2, SIX6, SUZ12, PTHLH, ASCL2, GTF2IRD1, SPDEF, MGA, TRP73, NFE2L3, ASCL3, TSHZ2, ADORA2A, ONECUT3, SOX5, SOX4, ELK1, SRY, OVOL2, HOXA4, ELK4, HOXA5, HOXA7, OVOL1, NKX3-2, ADAM33, RUNX1, NFE2, SUV39H1, TEAD2, TEAD3, NR4A3, ESX1, ATF7IP2, SALL3, HOXB4, HILS1, SEBOX, HOXB5, SMARCC1, HOXB6, RFX1, EIF4E2, POU6F1, AEBP1, SOX21, FOXK1, HR, PRRX1, PRDX2, PRRX2, CBFA2T3, NFATC2IP, BARX1, BARX2, LBH, PAX9, PRMT7, POU4F3, SOX17, LTB, PSMD9, SOX10, TBL1XR1, EGR3, ZFP553, KHDRBS2, SOX14, FOXJ1, SLA2, OTX2, ESR1, LEF1, ESR2, NRIP2, EYA3, EIF4G2, PRKCQ, AYM1, GHRH, ZFPM2, C1D, IRX4, IRX3, IRX5, TNF, FOXM1, GLIS1, NFYA, POU1F1, TCF7L2, ZFP493, DPF1, TNFRSF1A, NPAS1, NPAS2, ZKSCAN14, REL, POU2F3, POU2F2, RHOX2A, MYCBP, MYOG, NFATC4, INPP5D, NFATC2, ERF, INSM2, FOXL1, CREB3, SAP30BP, VSX2, ZFP286, HEYL, SETD7, MESP2, NFIB

Biological process	*Gene Count*	*Percentage*	*p-Value*	*Genes repressed in RasGrf1 KO pancreatic islets (from Additional file 1: Table S1)*
GO:0045761<br>regulation of adenylate cyclase activity	15	0,77	2,89E-03	ACR, CALCR, ADCY3, ADCY4, DRD3, DRD2, ADORA2A, PTH1R, DRD4, GHRHR, ADCYAP1, PTHLH, HRH3, GHRH, OPRD1
GO:0043067<br>regulation of programmed cell death	80	4,12	2,92E-03	PRDX2, FOXO3, PMAIP1, ITSN1, KCNIP3, MAP3K7, CASP6, PROP1, ATG7, TRP63, GRID2, FGF2, LTB, LTA, DHCR24, FGF4, PRKCA, CD3G, RARG, NOL3, BCL2L14, NKX2-6, BARHL1, HRK, ESR1, FADD, ESR2, PRKCD, JMY, PROC, TNFSF13B, CD40LG, PSME3, TRP73, NGFR, CLN8, NGF, ALOX12, AY074887, BID, CDK5R1, FGFR3, TNF, ADORA2A, STK17B, RAG1, COL2A1, ITM2B, TIMP1, ALB, BCL11B, BCL2, LHX3, NKX3-2, DIABLO, INPP5D, BMF, PTCRA, NKX2-5, TRAF4, CD27, ANGPTL4, COL18A1, PTPN6, IL2RB, TBX3, IL7, TBX5, SPHK1, IDO1, SAP30BP, DBH, FCGR1, TRADD, DLX1, EPHA7, CDKN1A, P2RX1, BIK, BMP7
GO:0050801<br>ion homeostasis	47	2,42	2,95E-03	CALCR, GNA15, JPH2, CCL2, GRIK1, ADORA2A, DRD2, CYP11B2, ERBB2, DRD4, PTH1R, ATP12A, 1300017J02RIK, SYPL2, CKB, BEST2, EDNRB, BCL2, NKX6-2, CCR10, GRID2, TRPV4, CHRNA4, IL1B, CHRNA6, TRFR2, TRPC2, PRKCA, HCN2, PLP1, CCKBR, ATP4B, FGF23, CACNG2, ATP1A2, TNNI3, CSRP3, NTSR2, GHRH, CHRNB4, CHRND, STC1, EPOR, CACNA1F, SCARA5, GPR12, CHRNG
GO:0031281<br>positive regulation of cyclase activity	12	0,62	3,05E-03	ADCY3, ACR, CALCR, PTHLH, ADCY4, DRD3, GHRH, ADORA2A, PTH1R, GHRHR, OPRD1, ADCYAP1
GO:0051349<br>positive regulation of lyase activity	12	0,62	3,05E-03	ADCY3, ACR, CALCR, PTHLH, ADCY4, DRD3, GHRH, ADORA2A, PTH1R, GHRHR, OPRD1, ADCYAP1

Biological process	*Gene Count*	*Percentage*	*p-Value*	*Genes repressed in RasGrf1 KO pancreatic islets (from Additional file 1: Table S1)*
GO:0010468 regulation of gene expression	292	15,04	3,08E-03	MEF2B, STRM, HIRA, RORA, CITED4, SHH, ZFP92, PGR, MED29, NKX6-2, IFNG, TIGD5, PITX3, PITX1, PHOX2A, WNT10B, RARG, RCOR3, NKX2-6, BARHL1, RXRG, EOMES, CYP1A2, HNF4G, MECOM, CPHX, PRDM9, BAZ1B, ABT1, PARP14, HES3, MED17, HES2, CDCA7L, VGLL4, HOXA11, IGF2BP1, NFKBIA, SFPI1, FOXH1, HESX1, TAL2, MUSK, LHX1, LHX3, LHX5, NKX2-4, NKX2-5, NKX2-3, HNRNPAB, TCF23, DMBX1, ZBTB48, ZBTB46, KLF9, IKZF1, KLF13, ESRRB, ESRRG, PMF1, DDX5, ABCG4, ZFP444, USF1, DLX3, DLX2, DLX1, FOXF1A, NR1I2, SUPT16H, BMP7, FOXI1, KLF1, TCF15, BACH2, EVX1, ELF3, ELF5, NFKB2, GLI2, GLI1, KCNIP3, ASGR2, SPIC, HSF4, NR2F2, TOP2A, FGF2, CSDC2, BATF2, PTF1A, GTF2H4, TLE4, NFAM1, MXD3, SIGIRR, MYCN, JMY, MXD4, BRDT, RUVBL2, KCNH3, TBX18, TBX19, MED1, MTDH, ZFP64, COL2A1, ZBTB17, SEC14L2, SUFU, ZFP111, MYCL1, ZFP36L2, STAT4, SORBS3, BCL11B, BCL2, ETV2, MLLT1, PER3, TCFEB, BAZ2A, TBX3, IL7, ZFP57, TBX5, FZD1, LMX1A, ZFP707, RHOX9, PHF19, JMJD6, PHGDH, NR5A2, TOB2, HMX1, IL16, CBX3, CBX2, TLR4, TBP, FOXO3, HOXD1, ANKRD1, MAF1, CBX7, IGHMBP2, HOXC6, BATF, ATOH1, PROP1, GATA5, GATA4, TRP63, ZFP503, ATOH7, CIITA, MAGEL2, SATB2, LBX1, TCFAP2C, RELB, SIX3, FOXN1, FGF23, SIX4, VAX1, NR0B1, GRHL2, SIX6, SUZ12, PTHLH, ASCL2, GTF2IRD1, SPDEF, MGA, CARTPT, TRP73, NFE2L3, ASCL3, TSHZ2, DRD3, ADORA2A, DRD2, ONECUT3, SOX5, SOX4, ELK1, SRY, OVOL2, HOXA4, ELK4, HOXA5, HOXA7, OVOL1, NKX3-2, RUNX1, NFE2, SUV39H1, TEAD2, TEAD3, NR4A3, ESX1, ATF7IP2, SALL3, HOXB4, HILS1, SEBOX, HOXB5, SMARCC1, HOXB6, RFX1, EIF4E2, POU6F1, AEBP1, SOX21, FOXK1, HR, PRRX1, PRDX2, PRRX2, CBFA2T3, NFATC2IP, BARX1, BARX2, LBH, PAX9, PRMT7, POU4F3, SOX17, PSMD9, SOX10, TBL1XR1, NLRP5, EGR3, ZFP553, KHDRBS2, SOX14, FOXJ1, SLA2, OTX2, ESR1, LEF1, ESR2, NRIP2, EYA3, EIF4G2, PRKCQ, AYM1, TNFSF13B, GHRH, ZFPM2, NGFR, C1D, IRX4, CALCR, IRX3, IRX5, TNF, FOXM1, GLIS1, NFYA, POU1F1, TCF7L2, ZFP493, DPF1, NPAS1, TNFRSF1A, TNFRSF1B, NPAS2, ZKSCAN14, REL, POU2F3, POU2F2, RHOX2A, MYCBP, MYOG, NFATC4, NFATC2, ERF, INSM2, IL2RB, FOXL1, CREB3, SAP30BP, VSX2, ZFP286, HEYL, SETD7, MESP2, NFIB
GO:0042981 regulation of apoptosis	79	4,07	3,17E-03	PRDX2, FOXO3, PMAIP1, ITSN1, KCNIP3, MAP3K7, CASP6, PROP1, ATG7, TRP63, GRID2, LTB, LTA, DHCR24, FGF4, PRKCA, CD3G, RARG, NOL3, BCL2L14, NKX2-6, BARHL1, HRK, ESR1, FADD, ESR2, PRKCD, JMY, PROC, TNFSF13B, CD40LG, PSME3, TRP73, NGFR, CLN8, NGF, ALOX12, AY074887, BID, CDK5R1, FGFR3, TNF, ADORA2A, STK17B, RAG1, COL2A1, ITM2B, TIMP1, ALB, BCL11B, BCL2, LHX3, NKX3-2, DIABLO, INPP5D, BMF, PTCRA, NKX2-5, TRAF4, CD27, ANGPTL4, COL18A1, PTPN6, IL2RB, TBX3, IL7, TBX5, SPHK1, IDO1, SAP30BP, DBH, FCGR1, TRADD, DLX1, EPHA7, CDKN1A, P2RX1, BIK, BMP7
GO:0001892 embryonic placenta development	15	0,77	3,39E-03	FGFR2, EGFR, ESRRB, EOMES, PCDH12, LEF1, GJB5, ESX1, ITGA4, WNT7B, OVOL2, ADM, GAB1, TCFEB, MED1
GO:0007167 enzyme linked receptor protein signaling pathway	44	2,27	3,73E-03	FGFR2, TWSG1, FGF18, NOG, FGFR3, CCL2, ERBB2, FGF17, CSPG4, DDR2, MAP3K7, FOXH1, MUSK, SORBS1, SOS1, SPNB2, GAB1, SH2B2, INSR, FIGF, FGF2, FGF3, CSF1R, FGF4, AMHR2, EGFR, MYO1E, MET, FGF23, SOCS7, FGF21, STXBP4, EPHA2, DDR1, EPHA4, EPHA7, EPHA6, PDGFRA, ROR1, PDGFRB, BMPR1B, BMP7, GFRA2, AKAP4

Biological process	*Gene Count*	*Percentage*	*p-Value*	*Genes repressed in RasGrf1 KO pancreatic islets (from Additional file 1: Table S1)*
GO:0060603 mammary gland duct morphogenesis	9	0,46	3,80E-03	PGR, PTHLH, FGFR2, DDR1, TBX3, CSF1, ESR1, GLI2, MED1
GO:0001947 heart looping	9	0,46	3,80E-03	LBX1, TBX3, OVOL2, WNT3A, GATA4, GJA1, NKX2-5, SHH, SUFU
GO:0060740 prostate gland epithelium morphogenesis	9	0,46	3,80E-03	FGFR2, NOG, RARG, SFRP1, TRP63, ESR1, ESR2, GLI2, SHH
GO:0001944 vasculature development	41	2,11	3,82E-03	FGFR2, FGF18, TNFRSF12A, FOXM1, PRRX1, CSPG4, GJA1, PRRX2, SHH, MAP3K7, PROP1, OVOL2, IL1B, NOS2, SOX17, ADRA2B, NR2F2, FIGF, FGF2, NKX2-5, COL18A1, TBX3, PDPN, MYO1E, SPHK1, ITGA4, ESX1, TNNI3, MYH9, MMP14, DBH, EPHA2, COL5A1, THY1, ANXA2, DLX3, FOXF1A, JMJD6, DLL4, ADRA1B, ZFPM2
GO:0032990 cell part morphogenesis	36	1,85	3,94E-03	CDK5R1, CCK, ADORA2A, DRD2, WNT3A, ERBB2, PIP5K1C, GLI2, SHH, TTLL3, ATOH1, BCL2, BCL11B, LHX3, POU4F3, SEMA3A, DCLK1, LRRC50, KLK8, PLA2G10, FOXJ1, NTNG2, VAX1, LMX1A, GAS7, NTN1, SLIT1, SEMA6A, SLITRK1, EPHA4, EPHA7, SEMA6C, NGFR, CACNA1F, BMPR1B, BMP7
GO:0006140 regulation of nucleotide metabolic process	17	0,88	3,95E-03	ADCY3, ACR, CALCR, ADCY4, DRD3, DRD2, ADORA2A, PTH1R, DRD4, GHRHR, ADCYAP1, PTHLH, HTR1B, HRH3, GHRH, GUCY1A3, OPRD1
GO:0043269 regulation of ion transport	15	0,77	3,97E-03	ORAI1, CASR, NOS1, TNF, ATP4B, DRD2, ADORA2A, HOMER1, THY1, SLN, P2RX1, BCL2, LTB, NKX2-5, LTA

Biological process	*Gene Count*	*Percentage*	*p-Value*	*Genes repressed in RasGrf1 KO pancreatic islets (from Additional file 1: Table S1)*
GO:0048667 cell morphogenesis involved in neuron differentiation	32	1,65	4,00E-03	CDK5R1, CCK, DRD2, ERBB2, WNT3A, PIP5K1C, GLI2, SHH, ATOH1, SLC1A3, BCL2, BCL11B, LHX3, POU4F3, SEMA3A, DCLK1, CDH23, PLA2G10, NTNG2, VAX1, LMX1A, NTN1, SLIT1, SEMA6A, SLITRK1, EPHA4, EPHA7, SEMA6C, CACNA1F, NGFR, BMPR1B, BMP7
GO:0030168 platelet activation	7	0,36	4,02E-03	FGG, PLEK, FGA, P2RX1, CD40LG, F2, ENTPD1
GO:0030307 positive regulation of cell growth	7	0,36	4,02E-03	TNFRSF12A, LIMK1, BCL2, BC048355, NTN1, NGF, ALOX12
GO:0060571 morphogenesis of an epithelial fold	7	0,36	4,02E-03	PTHLH, EGFR, FGFR2, NOG, OVOL2, TRP63, GLI2
GO:0050806 positive regulation of synaptic transmission	8	0,41	4,08E-03	LAMA2, TNF, SLC1A3, GRIK1, ADORA2A, NCS1, LTB, LTA
GO:0001701 in utero embryonic development	43	2,21	4,22E-03	FGFR2, NOG, WNT3A, RIC8, GNA12, GJA1, GLI2, ACVR1B, OVOL2, INPP5K, GATA4, DNMT3L, GAB1, TCFEB, RUNX1, MLL2, EGFR, SOX10, NLRP5, TBX3, ESRRB, MYO1E, DLL3, EOMES, PCDH12, LEF1, GJB5, DLL1, ESX1, ITGA4, MYH9, MECOM, ASCL2, HBA-A1, WNT7B, FOXF1A, ADM, HES3, PDGFRA, PDGFRB, ZFPM2, KLF1, MED1
GO:0051146 striated muscle cell differentiation	19	0,98	4,25E-03	NKX2-6, TBX5, ERBB2, MET, CACNG2, MYH9, HOMER1, FLNC, CACNA1S, CACNA2D2, SHH, MUSK, BARX2, DYRK1B, ATG7, GATA4, MYOG, NKX2-5, SNTA1

Biological process	*Gene Count*	*Percentage*	*p-Value*	*Genes repressed in RasGrf1 KO pancreatic islets (from Additional file 1: Table S1)*
GO:0001568 blood vessel development	40	2,06	4,32E-03	FGFR2, FGF18, TNFRSF12A, FOXM1, PRRX1, CSPG4, GJA1, PRRX2, SHH, MAP3K7, PROP1, OVOL2, IL1B, NOS2, SOX17, ADRA2B, NR2F2, FIGF, FGF2, NKX2-5, COL18A1, TBX3, MYO1E, SPHK1, ITGA4, ESX1, TNNI3, MYH9, MMP14, DBH, EPHA2, COL5A1, THY1, ANXA2, DLX3, FOXF1A, JMJD6, DLL4, ADRA1B, ZFPM2
GO:0055082 cellular chemical homeostasis	43	2,21	4,51E-03	CALCR, GNA15, JPH2, CCL2, GRIK1, ADORA2A, ERBB2, DRD4, PTH1R, SYPL2, 1300017J02RIK, CKB, BEST2, BCL2, NKX6-2, CCR10, GRID2, TRPV4, CHRNA4, IL1B, CHRNA6, TRFR2, TRPC2, PRKCA, HCN2, PLP1, CCKBR, FGF23, CACNG2, ATP1A2, TNNI3, CSRP3, NTSR2, GHRH, CHRNB4, CARTPT, CHRND, STC1, EPOR, CACNA1F, SCARA5, GPR12, CHRNG
GO:0048706 embryonic skeletal system development	18	0,93	4,70E-03	SATB2, PRRX1, PRRX2, HOXD1, SIX4, SHH, HOXC6, HOXB4, DLX2, DLX1, HOXA4, HOXA5, HOXB5, HOXA7, HOXB6, NKX3-2, ETL4, COL11A1
GO:0007435 salivary gland morphogenesis	9	0,46	4,92E-03	EGFR, FGFR2, TWSG1, TNF, SEMA3A, EDAR, BMP7, EDA, SHH
GO:0060512 prostate gland morphogenesis	9	0,46	4,92E-03	FGFR2, NOG, RARG, SFRP1, TRP63, ESR1, ESR2, GLI2, SHH
GO:0046661 male sex differentiation	14	0,72	4,95E-03	DHH, TBX3, ESR1, NR0B1, SHH, AHSG, GJB2, BCL2, PDGFRA, BIK, HSD17B4, SRD5A2, KLHL10, DHCR24
GO:0050433 regulation of catecholamine secretion	6	0,31	5,14E-03	DRD3, ADORA2A, DRD2, CARTPT, CHRNA4, CHRNA6

Biological process	***Gene Count***	***Percentage***	***p-Value***	***Genes repressed in RasGrf1 KO pancreatic islets (from Additional file 1: Table S1)***
GO:0010829 negative regulation of glucose transport	6	0,31	5,14E-03	PRKCA, TNF, IL1B, SH2B2, LTB, LTA
GO:0014902 myotube differentiation	6	0,31	5,14E-03	BARX2, DYRK1B, MET, MYH9, CACNA1S, SHH
GO:0042035 regulation of cytokine biosynthetic process	15	0,77	5,37E-03	FOXJ1, TLR4, IL21, TLR6, TLR7, TLR9, SIGIRR, PRKCQ, REL, IFNG, ADAM33, IL1B, INPP5D, LTB, CD27
GO:0010605 negative regulation of macromolecule metabolic process	72	3,71	5,38E-03	HMX1, FOXK1, HR, STRM, PRRX1, CBX3, CBX2, GLI2, MAF1, SHH, KCNIP3, BARX2, PROP1, NKX6-2, TRP63, HSF4, NR2F2, PRKCA, CIITA, TBL1XR1, SATB2, RARG, SOX14, FOXJ1, SLA2, SIX3, LEF1, TLE4, VAX1, NR0B1, PRKCD, MXD3, NRIP2, MXD4, SIGIRR, SUZ12, HES3, ZFPM2, C1D, WFIKKN1, MTDH, TNF, GLIS1, POU1F1, SUFU, FOXH1, NPAS1, SORBS3, INPP5K, OVOL2, INPP5J, RHOX2A, HOXA7, OVOL1, INPP5D, PER3, BAZ2A, NKX2-5, HNRNPAB, DMBX1, INSM2, PTPN6, IKZF1, TBX3, ZFP57, SUV39H1, FZD1, HOXB4, DLX2, DLX1, NR1I2, ADRA1B
GO:0060713 labyrinthine layer morphogenesis	7	0,36	5,67E-03	FGFR2, WNT7B, ADM, LEF1, GJB5, ESX1, ITGA4
GO:0051954 positive regulation of amine transport	5	0,26	5,81E-03	GRIK1, ADORA2A, DRD2, CARTPT, HTR2C
GO:0030323 respiratory tube development	22	1,13	6,00E-03	FGFR2, FGF18, MAN1A2, PDPN, TBX5, ASZ1, CYP1A2, GLI2, MMP14, SHH, GLI1, MYCN, PTHLH, FOXF1A, HOXA5, JMJD6, PDGFRA, SFTPD, ZFPM2, FGF2, TRAF4, NFIB

Biological process	*Gene Count*	*Percentage*	*p-Value*	*Genes repressed in RasGrf1 KO pancreatic islets (from Additional file 1: Table S1)*
GO:0021510 spinal cord development	12	0,62	6,47E-03	IGHMBP2, PHOX2A, LBX1, EVX1, ABT1, NKX6-2, LHX3, PHGDH, GLI2, CLN8, SHH, SUFU
GO:0060537 muscle tissue development	25	1,29	6,77E-03	ERBB2, ELN, SHH, MUSK, ATG7, GATA4, TRP63, MYOG, NR2F2, NKX2-5, COL11A1, PITX1, NKX2-6, TBX5, MET, RXRG, SIX4, CACNG2, HOMER1, CACNA2D2, CSRP3, CACNA1S, PDGFRB, ZFPM2, SNTA1
GO:0032944 regulation of mononuclear cell proliferation	17	0,88	7,59E-03	PTPN6, IL7, FOXJ1, ERBB2, IGH-6, TLR4, TNFRSF14, IDO1, CD40, SHH, PRKCQ, CDKN1A, TNFSF13B, FCGR2B, IFNG, IL12A, INPP5D
GO:0050670 regulation of lymphocyte proliferation	17	0,88	7,59E-03	PTPN6, IL7, FOXJ1, ERBB2, IGH-6, TLR4, TNFRSF14, IDO1, CD40, SHH, PRKCQ, CDKN1A, TNFSF13B, FCGR2B, IFNG, IL12A, INPP5D
GO:0007589 body fluid secretion	6	0,31	7,65E-03	ADORA2A, DRD2, CLCNKA, TRP73, GUCA2B, AGR2
GO:0045408 regulation of interleukin-6 biosynthetic process	6	0,31	7,65E-03	FOXJ1, IFNG, IL1B, TLR4, INPP5D, TLR6
GO:0048514 blood vessel morphogenesis	33	1,70	7,78E-03	FGFR2, FGF18, TNFRSF12A, FOXM1, PRRX1, CSPG4, GJA1, PRRX2, SHH, MAP3K7, OVOL2, IL1B, NOS2, SOX17, ADRA2B, NR2F2, FIGF, NKX2-5, FGF2, COL18A1, MYO1E, ITGA4, TNNI3, MYH9, DBH, MMP14, EPHA2, THY1, ANXA2, FOXF1A, DLL4, ADRA1B, ZFPM2

Biological process	***Gene Count***	***Percentage***	***p-Value***	***Genes repressed in RasGrf1 KO pancreatic islets (from Additional file 1: Table S1)***
GO:0045664 regulation of neuron differentiation	20	1,03	8,47E-03	IRX3, KLK8, LBX1, TNFRSF12A, LIMK1, SOX5, DLL1, LMX1A, GLI2, NTN1, SHH, THY1, ATOH1, METRN, INPP5J, HES3, BCL2, SEMA3A, CIT, NGF
GO:0007507 heart development	36	1,85	8,77E-03	IRX4, WNT3A, ERBB2, SOX4, GJA1, COL2A1, GLI2, SUFU, SHH, FOXH1, OVOL2, ATG7, GATA4, GAB1, GYS1, NFATC4, NKX2-5, COL11A1, LBX1, TBX3, NKX2-6, TBX5, ITGA4, TNNI3, MECOM, CSRP3, COL5A1, ADAP2, ADM, JMJD6, ADRA1B, EPOR, ZFPM2, VCAN, HTR2B, MED1
GO:0043066 negative regulation of apoptosis	38	1,96	8,94E-03	ADORA2A, RAG1, COL2A1, PRDX2, ITSN1, TIMP1, MAP3K7, PROP1, ALB, ATG7, BCL2, BCL11B, LHX3, TRP63, NKX3-2, PTCRA, NKX2-5, CD27, ANGPTL4, DHCR24, FGF4, IL2RB, TBX3, NKX2-6, IL7, BARHL1, SPHK1, ESR1, IDO1, PROC, DLX1, CDKN1A, TNFSF13B, CD40LG, TRP73, CLN8, NGF, ALOX12
GO:0042108 positive regulation of cytokine biosynthetic process	11	0,57	9,53E-03	PRKCQ, REL, IFNG, IL1B, TLR4, TLR6, IL21, TLR7, LTB, CD27, TLR9
GO:0048469 cell maturation	16	0,82	9,56E-03	SOX10, PLP1, IRX5, PTH1R, GJA1, PDE3A, FOXO3, ESR2, GHRHR, TIMP1, PGR, REC8, AGPAT6, POU2F2, KCNE1, KLF1
GO:0045124 regulation of bone resorption	5	0,26	9,60E-03	CALCR, IAPP, CARTPT, INPP5D, AHSG
GO:0021984 adenohypophysis development	5	0,26	9,60E-03	PROP1, GHRH, DRD2, POU1F1, GHRHR
GO:0046850 regulation of bone remodeling	5	0,26	9,60E-03	CALCR, IAPP, CARTPT, INPP5D, AHSG

Biological process	*Gene Count*	*Percentage*	*p-Value*	*Genes repressed in RasGrf1 KO pancreatic islets (from Additional file 1: Table S1)*
GO:0070663 regulation of leukocyte proliferation	17	0,88	9,65E-03	PTPN6, IL7, FOXJ1, ERBB2, IGH-6, TLR4, TNFRSF14, IDO1, CD40, SHH, PRKCQ, CDKN1A, TNFSF13B, FCGR2B, IFNG, IL12A, INPP5D
GO:0010627 regulation of protein kinase cascade	27	1,39	9,67E-03	FGFR2, SH3RF1, FGFR3, TNF, DRD3, STK11, DRD2, TLR4, TLR6, ITSN1, MAP3K7, SORBS3, IFNG, GAB1, IL1B, PRL, LTB, LTA, CD27, PTPN6, FGF23, IGH-6, FGF21, TRADD, TNFSF11, TRP73, EDA
GO:0043588 skin development	9	0,46	9,79E-03	TRP63, NGFR, EDA, LTB, COL5A1, SHH, TCF15, SUFU, DHCR24
GO:0050864 regulation of B cell activation	13	0,67	9,80E-03	PTPN6, FOXJ1, IL7, IGH-6, TLR4, NFAM1, CD40, IL21, CDKN1A, FCGR2B, TNFSF13B, IFNG, INPP5D
GO:0030324 lung development	21	1,08	1,02E-02	FGFR2, FGF18, MAN1A2, PDPN, TBX5, ASZ1, CYP1A2, GLI2, MMP14, SHH, GLI1, MYCN, PTHLH, FOXF1A, HOXA5, JMJD6, PDGFRA, SFTPD, ZFPM2, FGF2, NFIB
GO:0030901 midbrain development	7	0,36	1,04E-02	TAL2, FGFR2, BARHL1, HES3, OTX2, PITX3, SHH
GO:0048535 lymph node development	7	0,36	1,04E-02	TNFRSF11A, TNFSF11, IKZF1, NFKB2, LTB, LTA, NKX2-3
GO:0014013 regulation of gliogenesis	6	0,31	1,09E-02	DLX2, DLX1, NOG, DRD3, NKX6-2, SHH
GO:0050900 leukocyte migration	11	0,57	1,13E-02	PRKCA, IL17B, TNF, IL16, FOXJ1, CCR1, IFNG, S100A9, IL1B, DBH, NKX2-3

Biological process	*Gene Count*	*Percentage*	*p-Value*	*Genes repressed in RasGrf1 KO pancreatic islets (from Additional file 1: Table S1)*
GO:0014706 striated muscle tissue development	23	1,18	1,15E-02	NKX2-6, ERBB2, TBX5, ELN, MET, RXRG, SIX4, CACNG2, HOMER1, CACNA1S, CSRP3, CACNA2D2, SHH, MUSK, ATG7, GATA4, MYOG, ZFPM2, NR2F2, NKX2-5, COL11A1, PITX1, SNTA1
GO:0002064 epithelial cell development	8	0,41	1,17E-02	COL18A1, PGR, AGPAT6, TRP63, ESR1, KCNE1, GJA1, ESR2
GO:0007431 salivary gland development	9	0,46	1,20E-02	EGFR, FGFR2, TWSG1, TNF, SEMA3A, EDAR, BMP7, EDA, SHH
GO:0043069 negative regulation of programmed cell death	38	1,96	1,23E-02	ADORA2A, RAG1, COL2A1, PRDX2, ITSN1, TIMP1, MAP3K7, PROP1, ALB, ATG7, BCL2, BCL11B, LHX3, TRP63, NKX3-2, PTCRA, NKX2-5, CD27, ANGPTL4, DHCR24, FGF4, IL2RB, TBX3, NKX2-6, IL7, BARHL1, SPHK1, ESR1, IDO1, PROC, DLX1, CDKN1A, TNFSF13B, CD40LG, TRP73, CLN8, NGF, ALOX12
GO:0060548 negative regulation of cell death	38	1,96	1,31E-02	ADORA2A, RAG1, COL2A1, PRDX2, ITSN1, TIMP1, MAP3K7, PROP1, ALB, ATG7, BCL2, BCL11B, LHX3, TRP63, NKX3-2, PTCRA, NKX2-5, CD27, ANGPTL4, DHCR24, FGF4, IL2RB, TBX3, NKX2-6, IL7, BARHL1, SPHK1, ESR1, IDO1, PROC, DLX1, CDKN1A, TNFSF13B, CD40LG, TRP73, CLN8, NGF, ALOX12
GO:0007242 intracellular signaling cascade	117	6,02	1,32E-02	ADCY3, ADCY4, RP1, CASR, GNA15, ADCY8, GNA12, CSPG4, TLR4, TLR6, ITSN1, IQGAP1, GHRHR, MAP3K7, PGR, PRRT1, IFNG, GAB1, TRP63, TLK2, MAP2K7, RARG, RXRG, SOCS7, CYP26A1, NTSR2, MARK1, PTHLH, MAP4K5, RASGRF2, HRASLS, RAB17, REM2, REM1, CARTPT, 5430435G22RIK, FGFR3, DRD3, DRD2, ADORA2A, PTH1R, DRD4, STK17B, ASB12, MAP4K1, MYO9B, TRH, ASB16, ASB18, RAC3, RHOBTB2, ADRA2B, CDC42EP4, ARHGEF10L, GNAT1, PTPN6, RAB8B, KLF9, MET, BMX, IGH-6, PDE6H, PDE6G, AGER, ADAP2, ARF4, RGS6, FBXO31, NRG3, PRDX2, CLCF1, GUCY1A3, NOS2, LTB, PRL, FGF2, LTA, HTR5A, PRKCA, PTF1A, ESR2, NFAM1, PRKCD, PRKCC, ARHGEF10, PRKD2, PRKCQ, MAST1, CHRM3, GHRH, GADD45B, CALCR, TNF, RAB3D, PLEK2, GNG13, SOS1, PLEKHG6, RASGRP1, RASGRP2, SH2B2, INPP5D, DCLK1, RAB2A, RAB2B, GDI2, PLEK, SPSB2, RASL11B, MAPK13, RASSF1, DUSP9, CIT, HTR2B, HTR2C, BCAR3, OPRD1

Biological process	*Gene Count*	*Percentage*	*p-Value*	*Genes repressed in RasGrf1 KO pancreatic islets (from Additional file 1: Table S1)*
GO:0009953 dorsal/ventral pattern formation	14	0,72	1,34E-02	EVX1, HOXA11, OTX2, GLI2, SHH, SUFU, GLI1, PROP1, OVOL2, LHX1, NKX6-2, LHX3, BMPR1B, CHRD
GO:0014075 response to amine stimulus	7	0,36	1,35E-02	SLC1A3, DRD3, ADORA2A, DRD2, DRD4, ADRA1B, DBH
GO:0010827 regulation of glucose transport	7	0,36	1,35E-02	PRKCA, TNF, SORBS1, IL1B, SH2B2, LTB, LTA
GO:0021522 spinal cord motor neuron differentiation	7	0,36	1,35E-02	IGHMBP2, PHOX2A, ABT1, NKX6-2, LHX3, GLI2, CLN8
GO:0051249 regulation of lymphocyte activation	25	1,29	1,36E-02	ADORA2A, ERBB2, PRDX2, TLR4, SHH, IFNG, INPP5D, CD27, PTPN6, SIT1, IKZF1, IL7, FOXJ1, IGH-6, TNFRSF14, CD40, IDO1, NFAM1, IL21, THY1, PRKCQ, CDKN1A, TNFSF13B, FCGR2B, IL12A
GO:0042113 B cell activation	16	0,82	1,36E-02	EXO1, IKZF1, RAG1, NFAM1, CD40, POU1F1, PRKCD, CD40LG, CLCF1, BCL2, IGBP1, POU2F2, CHRNA4, CD27, BLNK, NKX2-3
GO:0048332 mesoderm morphogenesis	10	0,51	1,41E-02	FGFR2, TWSG1, TBX3, WNT3A, EOMES, LEF1, NR4A3, BMP7, CHRD, EPHA2
GO:0060191 regulation of lipase activity	9	0,46	1,46E-02	GNA15, CHRM3, DRD2, PTH1R, GNG13, HTR2B, HTR2C, NTSR2, ANGPTL4

Biological process	*Gene Count*	*Percentage*	*p-Value*	*Genes repressed in RasGrf1 KO pancreatic islets (from Additional file 1: Table S1)*
GO:0002070<br>epithelial cell maturation	5	0,26	1,47E-02	PGR, AGPAT6, KCNE1, GJA1, ESR2
GO:0032890<br>regulation of organic acid transport	5	0,26	1,47E-02	TNF, GRIK1, HTR2C, LTB, LTA
GO:0051955<br>regulation of amino acid transport	5	0,26	1,47E-02	TNF, GRIK1, HTR2C, LTB, LTA
GO:0016055<br>Wnt receptor signaling pathway	23	1,18	1,49E-02	WNT10B, WNT16, WNT5B, NKD2, STK11, WNT3A, FZD1, SOX4, LEF1, TLE4, TCF7L2, FZD6, WNT2B, DKK4, SDC1, WNT7B, PROP1, NXN, SFRP1, FRAT1, WNT6, WNT8A, WNT8B
GO:0001975<br>response to amphetamine	6	0,31	1,50E-02	DRD3, ADORA2A, DRD2, DRD4, ADRA1B, DBH
GO:0032655<br>regulation of interleukin-12 production	6	0,31	1,50E-02	REL, IFNG, IDO1, CD40, TLR6, LTB
GO:0051017<br>actin filament bundle formation	6	0,31	1,50E-02	LIMA1, SORBS1, AIF1, ELN, ACTN1, GAS7

Biological process	***Gene Count***	***Percentage***	***p-Value***	***Genes repressed in RasGrf1 KO pancreatic islets (from Additional file 1: Table S1)***
GO:0019724<br>B cell mediated immunity	14	0,72	1,52E-02	EXO1, C4B, C3, IGH-6, PRKCD, FCGR1, IGHG, C4BP, FCGR2B, CD40LG, POU2F2, INPP5D, C2, IGH-VJ558
GO:0051051<br>negative regulation of transport	15	0,77	1,53E-02	PRKCA, NOS1, TNF, FGF23, TACC3, SUFU, PACSIN1, PACSIN3, OIT1, BCL2, IL1B, SH2B2, LTB, LTA, PSMD9
GO:0042742<br>defense response to bacterium	20	1,03	1,54E-02	TNF, NGP, NCF1, HCK, TLR4, FCGR1, LEAP2, DEFB6, TNFRSF1A, WFDC12, DEFA-RS7, IFNA1, IFNB1, PGLYRP2, IFNG, NOS2, DEFB11, DEFB35, CTSG, DEFB4
GO:0002694<br>regulation of leukocyte activation	26	1,34	1,63E-02	ADORA2A, ERBB2, PRDX2, TLR4, RORA, SHH, IFNG, INPP5D, CD27, PTPN6, SIT1, IKZF1, IL7, FOXJ1, TNFRSF14, IGH-6, CD40, IDO1, NFAM1, IL21, THY1, PRKCQ, CDKN1A, TNFSF13B, FCGR2B, IL12A
GO:0030850<br>prostate gland development	10	0,51	1,67E-02	FGFR2, NOG, RARG, SFRP1, TRP63, ESR1, ESR2, GLI2, SHH, CYP19A1
GO:0060438<br>trachea development	4	0,21	1,77E-02	FOXF1A, RARG, HOXA5, SHH
GO:0060601<br>lateral sprouting from an epithelium	4	0,21	1,77E-02	FGFR2, NOG, TRP63, GLI2
GO:0048305<br>immunoglobulin secretion	4	0,21	1,77E-02	TNFSF13B, CD40LG, POU2F2, CD27

Biological process	*Gene Count*	*Percentage*	*p-Value*	*Genes repressed in RasGrf1 KO pancreatic islets (from Additional file 1: Table S1)*
GO:0048570 notochord morphogenesis	4	0,21	1,77E-02	NOG, GLI2, EPHA2, GLI1
GO:0040018 positive regulation of multicellular organism growth	8	0,41	1,79E-02	IKZF1, GHRH, DRD2, BCL2, CSF1, POU1F1, GH, GHRHR
GO:0051046 regulation of secretion	22	1,13	1,84E-02	TNF, GRIK1, DRD3, RAB3D, DRD2, ADORA2A, FGF23, PARK2, CD40, GHRHR, SSTR5, OIT1, GHRH, CD40LG, IFNG, CARTPT, CHRNA4, IL1B, CHRNA6, HTR2C, PSMD9, NGF
GO:0051348 negative regulation of transferase activity	12	0,62	1,89E-02	PRKCA, PTPN6, CDKN1A, HMGCR, ADORA2A, MLLT1, IL1B, TRP73, DUSP9, GADD45B, CLN8, THY1
GO:0007369 gastrulation	15	0,77	1,93E-02	CER1, FGFR2, TWSG1, NAT8, WNT3A, HIRA, EOMES, LEF1, NR4A3, ZBTB17, EPHA2, LHX1, GATA4, BMP7, CHRD
GO:0030902 hindbrain development	14	0,72	1,94E-02	PHOX2A, PTF1A, LMX1A, RORA, GLI2, SHH, GLI1, KLHL1, LHX1, HES3, BCL2, ATG7, LHX5, NFIB
GO:0051258 protein polymerization	10	0,51	1,96E-02	MTSS1, TUBA8, FGG, FGA, TUBA-RS1, TUBB2A, TUBA3A, ASZ1, TUBB6, GAS7
GO:0001704 formation of primary germ layer	10	0,51	1,96E-02	FGFR2, TWSG1, LHX1, WNT3A, EOMES, LEF1, NR4A3, BMP7, CHRD, EPHA2

Biological process	*Gene Count*	*Percentage*	*p-Value*	*Genes repressed in RasGrf1 KO pancreatic islets (from Additional file 1: Table S1)*
GO:0035050 embryonic heart tube development	6	0,31	1,99E-02	FOXH1, NKX2-6, GATA4, GJA1, NKX2-5, MED1
GO:0001654 eye development	26	1,34	2,04E-02	TWSG1, IRX5, SHH, MIP, CRB1, BCL11B, HSF4, CRYBB2, GNAT1, RARG, CRYAA, IKZF1, OTX2, PTF1A, SIX3, VAX1, VSX2, PRPH2, CACNA1S, THY1, GABRR2, JMJD6, BMP7, BMPR1B, CLN8, MED1
GO:0048663 neuron fate commitment	11	0,57	2,08E-02	DLX2, DLX1, ATOH1, LBX1, EVX1, NKX6-2, PTF1A, OTX2, DLL1, GLI2, SHH
GO:0002573 myeloid leukocyte differentiation	9	0,46	2,09E-02	CALCR, TNF, TNFSF11, RELB, IL25, SFPI1, CBFA2T3, MYH9, NKX2-3
GO:0002076 osteoblast development	5	0,26	2,12E-02	PTHLH, SATB2, PTH1R, GLI2, SHH
GO:0048557 embryonic digestive tract morphogenesis	5	0,26	2,12E-02	FGFR2, OVOL2, GATA4, TCF7L2, SHH
GO:0046325 negative regulation of glucose import	5	0,26	2,12E-02	PRKCA, TNF, SH2B2, LTB, LTA

Biological process	*Gene Count*	*Percentage*	*p-Value*	*Genes repressed in RasGrf1 KO pancreatic islets (from Additional file 1: Table S1)*
GO:0045981 positive regulation of nucleotide metabolic process	5	0,26	2,12E-02	PTHLH, GHRH, ADORA2A, GUCY1A3, GHRHR
GO:0009063 cellular amino acid catabolic process	12	0,62	2,15E-02	BCAT1, GAD2, FTCD, PRODH2, MOXD2, UROC1, NOS2, IDO1, DBH, SARDH, GLDC, AUH
GO:0002449 lymphocyte mediated immunity	15	0,77	2,39E-02	EXO1, PTPN6, C4B, C3, IGH-6, PRKCD, FCGR1, IGHG, C4BP, FCGR2B, CD40LG, POU2F2, INPP5D, C2, IGH-VJ558
GO:0010959 regulation of metal ion transport	11	0,57	2,40E-02	ORAI1, CASR, SLN, NOS1, P2RX1, ADORA2A, DRD2, BCL2, HOMER1, NKX2-5, THY1
GO:0045670 regulation of osteoclast differentiation	6	0,31	2,59E-02	TNF, TNFSF11, CSF1, CARTPT, INPP5D, TOB2
GO:0002761 regulation of myeloid leukocyte differentiation	8	0,41	2,63E-02	TNF, TNFSF11, IKZF1, CSF1, CARTPT, INPP5D, RUNX1, TOB2
GO:0008542 visual learning	8	0,41	2,63E-02	DRD3, PDE1B, HMGCR, DRD2, RIC8, ADRA1B, ATP1A2, DBH

Biological process	***Gene Count***	***Percentage***	***p-Value***	***Genes repressed in RasGrf1 KO pancreatic islets (from Additional file 1: Table S1)***
GO:0016202 regulation of striated muscle tissue development	10	0,51	2,65E-02	FGFR2, NOG, MUSK, TBX3, BCL2, TBX5, GATA4, GJA1, LEF1, SHH
GO:0016125 sterol metabolic process	15	0,77	2,65E-02	CYB5R3, CUBN, MVD, CYP46A1, HMGCR, APOC1, APOA1, LCAT, CYP7A1, APOC3, MVK, CLN8, MBTPS1, HSD17B7, DHCR24
GO:0006695 cholesterol biosynthetic process	7	0,36	2,69E-02	CYB5R3, APOA1, MVD, HMGCR, MVK, HSD17B7, DHCR24
GO:0048536 spleen development	7	0,36	2,69E-02	HOXB4, BARX1, BCL2, NKX3-2, NFKB2, NKX2-5, NKX2-3
GO:0021695 cerebellar cortex development	7	0,36	2,69E-02	LHX1, ATG7, LHX5, RORA, GLI2, KLHL1, GLI1
GO:0050678 regulation of epithelial cell proliferation	13	0,67	2,76E-02	FGFR2, EGFR, NOG, FGFR3, STK11, ESR2, SHH, PGR, PLA2G2A, TBX18, PRL, FGF2, MED1
GO:0008209 androgen metabolic process	4	0,21	2,86E-02	ESR1, SRD5A2, SHH, CYP19A1

Biological process	*Gene Count*	*Percentage*	*p-Value*	*Genes repressed in RasGrf1 KO pancreatic islets (from Additional file 1: Table S1)*
GO:0046627 negative regulation of insulin receptor signaling pathway	4	0,21	2,86E-02	PRKCA, IL1B, PRKCD, AHSG
GO:0045410 positive regulation of interleukin-6 biosynthetic process	4	0,21	2,86E-02	IFNG, IL1B, TLR4, TLR6
GO:0045773 positive regulation of axon extension	4	0,21	2,86E-02	TNFRSF12A, LIMK1, NTN1, NGF
GO:0032845 negative regulation of homeostatic process	4	0,21	2,86E-02	IAPP, BCL2, CARTPT, INPP5D
GO:0045084 positive regulation of interleukin-12 biosynthetic process	4	0,21	2,86E-02	REL, IFNG, TLR6, LTB

Biological process	*Gene Count*	*Percentage*	*p-Value*	*Genes repressed in RasGrf1 KO pancreatic islets (from Additional file 1: Table S1)*
GO:0060687 regulation of branching involved in prostate gland morphogenesis	4	0,21	2,86E-02	FGFR2, SFRP1, ESR1, BMP7
GO:0034655 nucleobase, nucleoside, nucleotide and nucleic acid catabolic process	9	0,46	2,90E-02	ASPDH, NCF2, NCF1, ENTPD7, DERA, MYH7, BC048355, ENTPD1, CANT1
GO:0001707 mesoderm formation	9	0,46	2,90E-02	FGFR2, TWSG1, WNT3A, EOMES, LEF1, NR4A3, BMP7, CHRD, EPHA2
GO:0033559 unsaturated fatty acid metabolic process	9	0,46	2,90E-02	TNFRSF1A, GGT5, PTGIS, PDPN, NCF1, PTGES, EPHX2, LTA4H, ALOX12
GO:0034656 nucleobase, nucleoside and nucleotide catabolic process	9	0,46	2,90E-02	ASPDH, NCF2, NCF1, ENTPD7, DERA, MYH7, BC048355, ENTPD1, CANT1

Biological process	***Gene Count***	***Percentage***	***p-Value***	***Genes repressed in RasGrf1 KO pancreatic islets (from Additional file 1: Table S1)***
GO:0006833<br>water transport	5	0,26	2,93E-02	MIP, AQP9, AQP8, AQP3, AQP2
GO:0048645<br>organ formation	5	0,26	2,93E-02	NOG, NKX3-2, TRP63, GLI2, SHH
GO:0021513<br>spinal cord dorsal/ventral patterning	5	0,26	2,93E-02	EVX1, NKX6-2, GLI2, SHH, SUFU
GO:0030097<br>hemopoiesis	37	1,91	2,93E-02	CALCR, TWSG1, TNF, CCR1, WNT3A, RAG1, SOX4, SFPI1, TRIM10, CBFA2T3, POU1F1, TIMP1, ANK1, CLCF1, BCL2, BCL11B, POU2F2, RUNX1, NKX2-5, NKX2-3, IKZF1, IL7, MYO1E, RELB, IL25, EOMES, NFAM1, MYH9, TACC3, HBA-A1, HOXB4, TNFSF11, CD40LG, JMJD6, KLF1, ADD2, MED1
GO:0070201<br>regulation of establishment of protein localization	14	0,72	3,02E-02	TNF, ADORA2A, CD40, EDAR, TACC3, SHH, SUFU, SORBS1, CD40LG, IFNG, EDA, LTB, MBTPS1, LTA
GO:0060513<br>prostatic bud formation	3	0,15	3,03E-02	NOG, TRP63, GLI2
GO:0019364<br>pyridine nucleotide catabolic process	3	0,15	3,03E-02	ASPDH, NCF2, NCF1
GO:0048755<br>branching morphogenesis of a nerve	3	0,15	3,03E-02	DLX2, EPHA7, DRD2

Biological process	***Gene Count***	***Percentage***	***p-Value***	***Genes repressed in RasGrf1 KO pancreatic islets (from Additional file 1: Table S1)***
GO:0060523 prostate epithelial cord elongation	3	0,15	3,03E-02	FGFR2, ESR1, SHH
GO:0042365 water-soluble vitamin catabolic process	3	0,15	3,03E-02	ASPDH, NCF2, NCF1
GO:0006742 NADP catabolic process	3	0,15	3,03E-02	ASPDH, NCF2, NCF1
GO:0008016 regulation of heart contraction	11	0,57	3,11E-02	PRKCA, IRX5, NOS1, GNAO1, DRD2, ADRA1B, GJA1, SEMA3A, ATP1A2, NKX2-5, CSRP3
GO:0060485 mesenchyme development	11	0,57	3,11E-02	FGFR2, EDNRB, NOG, OVOL2, HOXA5, BCL2, EOMES, LEF1, CYP26A1, SHH, HNRNPAB
GO:0001942 hair follicle development	11	0,57	3,11E-02	EGFR, ACVR1B, BARX2, BCL2, TRP63, TNFRSF19, EDAR, NGFR, EDA, KRT71, SHH
GO:0050871 positive regulation of B cell activation	9	0,46	3,37E-02	CDKN1A, TNFSF13B, IL7, IFNG, TLR4, IGH-6, INPP5D, CD40, IL21

Biological process	***Gene Count***	***Percentage***	***p-Value***	***Genes repressed in RasGrf1 KO pancreatic islets (from Additional file 1: Table S1)***
GO:0051223<br>regulation of protein transport	13	0,67	3,44E-02	TNF, ADORA2A, CD40, EDAR, TACC3, SHH, SUFU, CD40LG, IFNG, EDA, LTB, MBTPS1, LTA
GO:0031347<br>regulation of defense response	18	0,93	3,69E-02	PRKCA, C3, ADORA2A, SAMHD1, NFKBIA, TLR4, IDO1, IL21, FCGR1, POLR3D, AHSG, IGHG, TNFRSF1A, TNFRSF1B, FCGR2B, AOAH, KLRB1B, KLRB1C
GO:0007632<br>visual behavior	8	0,41	3,69E-02	DRD3, PDE1B, HMGCR, DRD2, RIC8, ADRA1B, ATP1A2, DBH
GO:0050679<br>positive regulation of epithelial cell proliferation	8	0,41	3,69E-02	EGFR, FGFR2, NOG, TBX18, FGF2, PRL, SHH, MED1
GO:0007126<br>meiosis	16	0,82	3,74E-02	EXO1, ADCY3, SPO11, PTTG1, MYH9, SMC3, KLHDC3, REC8, CCNB3, PRDM9, CLGN, RSPH1, SIAH1A, OVOL1, CLASP2, H1FOO
GO:0051327<br>M phase of meiotic cell cycle	16	0,82	3,74E-02	EXO1, ADCY3, SPO11, PTTG1, MYH9, SMC3, KLHDC3, REC8, CCNB3, PRDM9, CLGN, RSPH1, SIAH1A, OVOL1, CLASP2, H1FOO
GO:0060562<br>epithelial tube morphogenesis	19	0,98	3,78E-02	FGFR2, TBX3, CSF1, HOXA11, ESR1, GLI2, SHH, SUFU, FZD6, MAP3K7, PTHLH, PGR, DDR1, FOXF1A, ADM, OVOL2, HOXA5, BCL2, MED1
GO:0014073<br>response to tropane	5	0,26	3,89E-02	DRD3, DRD2, DRD4, ADRA1B, DLG4
GO:0042220<br>response to cocaine	5	0,26	3,89E-02	DRD3, DRD2, DRD4, ADRA1B, DLG4

Biological process	*Gene Count*	*Percentage*	*p-Value*	*Genes repressed in RasGrf1 KO pancreatic islets (from Additional file 1: Table S1)*
GO:0048747 muscle fiber development	9	0,46	3,90E-02	MUSK, ERBB2, MYOG, CACNG2, HOMER1, FLNC, CACNA2D2, CACNA1S, SNTA1
GO:0001708 cell fate specification	12	0,62	3,91E-02	ATOH1, LBX1, EVX1, HOXA11, NKX6-2, OTX2, SIX3, POU1F1, GLI2, FGF2, FGF3, SHH
GO:0051090 regulation of transcription factor activity	12	0,62	3,91E-02	PTHLH, PRKCQ, MTDH, TNF, FOXJ1, NFKBIA, PRDX2, TLR4, NFAM1, SHH, SIGIRR, JMY
GO:0009954 proximal/distal pattern formation	7	0,36	3,95E-02	DLX2, IRX3, DLX1, HOXA11, TRP63, GLI2, GLI1
GO:0060688 regulation of morphogenesis of a branching structure	7	0,36	3,95E-02	FGFR2, TNF, SFRP1, MET, ESR1, BMP7, SHH
GO:0006940 regulation of smooth muscle contraction	7	0,36	3,95E-02	CHRM3, P2RX1, NCF1, CHRNB4, GUCY1A3, ATP1A2, TNNI3
GO:0042063 gliogenesis	11	0,57	3,97E-02	SOX10, METRN, PLP1, ADORA2A, NKX6-2, CSPG4, PHGDH, MMP14, EIF2B1, FGF2, SHH
GO:0060538 skeletal muscle organ development	14	0,72	4,08E-02	ERBB2, MET, ELN, RXRG, CACNG2, SIX4, HOMER1, CACNA2D2, CACNA1S, MUSK, MYOG, NR2F2, PITX1, SNTA1

Biological process	*Gene Count*	*Percentage*	*p-Value*	*Genes repressed in RasGrf1 KO pancreatic islets (from Additional file 1: Table S1)*
GO:0046324<br>regulation of glucose import	6	0,31	4,09E-02	PRKCA, TNF, SORBS1, SH2B2, LTB, LTA
GO:0060444<br>branching involved in mammary gland duct morphogenesis	6	0,31	4,09E-02	PGR, DDR1, TBX3, CSF1, ESR1, MED1
GO:0048665<br>neuron fate specification	6	0,31	4,09E-02	ATOH1, LBX1, EVX1, NKX6-2, GLI2, SHH
GO:0021514<br>ventral spinal cord interneuron differentiation	4	0,21	4,23E-02	EVX1, NKX6-2, GLI2, SHH
GO:0021521<br>ventral spinal cord interneuron specification	4	0,21	4,23E-02	EVX1, NKX6-2, GLI2, SHH
GO:0031017<br>exocrine pancreas development	4	0,21	4,23E-02	PTF1A, SOX4, IGF2, INSR

Biological process	*Gene Count*	*Percentage*	*p-Value*	*Genes repressed in RasGrf1 KO pancreatic islets (from Additional file 1: Table S1)*
GO:0060579 ventral spinal cord interneuron fate commitment	4	0,21	4,23E-02	EVX1, NKX6-2, GLI2, SHH
GO:0050778 positive regulation of immune response	22	1,13	4,23E-02	PTPN6, FCER2A, C3, C4B, CD247, NFKBIA, IGH-6, TLR4, IDO1, NFAM1, IL21, FCGR1, POLR3D, THY1, IGHG, C4BP, CD19, TNFSF13B, SH2B2, C2, CFD, KLRB1C
GO:0030003 cellular cation homeostasis	24	1,24	4,26E-02	TRPC2, CALCR, PRKCA, GNA15, JPH2, CCL2, CCKBR, PTH1R, ATP1A2, TNNI3, CSRP3, SYPL2, 1300017J02RIK, GHRH, BCL2, CCR10, IL1B, TRPV4, STC1, EPOR, CACNA1F, SCARA5, GPR12, TRFR2
GO:0030522 intracellular receptor-mediated signaling pathway	8	0,41	4,31E-02	PGR, RARG, KLF9, PTF1A, RXRG, CYP26A1, ESR2, NOS2
GO:0048741 skeletal muscle fiber development	8	0,41	4,31E-02	MUSK, ERBB2, MYOG, CACNG2, HOMER1, CACNA2D2, CACNA1S, SNTA1
GO:0032675 regulation of interleukin-6 production	8	0,41	4,31E-02	TNF, FOXJ1, IFNG, IL1B, TLR4, INPP5D, TLR6, TLR7
GO:0060349 bone morphogenesis	7	0,36	4,70E-02	PTHLH, FGFR2, FGF18, RARG, HOXA11, ACP5, COL2A1

Biological process	*Gene Count*	*Percentage*	*p-Value*	*Genes repressed in RasGrf1 KO pancreatic islets (from Additional file 1: Table S1)*
GO:0055001 muscle cell development	12	0,62	4,84E-02	MUSK, NKX2-6, ERBB2, ATG7, MYOG, CACNG2, HOMER1, FLNC, NKX2-5, CACNA2D2, CACNA1S, SNTA1
GO:0031345 negative regulation of cell projection organization	6	0,31	5,00E-02	KLK8, INPP5J, SEMA3A, CIT, NTN1, THY1
GO:0007439 ectodermal gut development	5	0,26	5,00E-02	FOXF1A, GATA4, GLI2, TCF7L2, SHH
GO:0021680 cerebellar Purkinje cell layer development	5	0,26	5,00E-02	LHX1, ATG7, LHX5, RORA, KLHL1
GO:0021511 spinal cord patterning	5	0,26	5,00E-02	EVX1, NKX6-2, GLI2, SHH, SUFU
GO:0048567 ectodermal gut morphogenesis	5	0,26	5,00E-02	FOXF1A, GATA4, GLI2, TCF7L2, SHH
GO:0051969 regulation of transmission of nerve impulse	18	0,93	5,04E-02	KLK8, TNF, GRIK1, DRD2, ADORA2A, NCS1, PARK2, SYNGR1, CACNA2D2, LAMA2, SLC1A3, IFNG, DLG4, CARTPT, HTR2C, LTB, LTA, NGF

Biological process	***Gene Count***	***Percentage***	***p-Value***	***Genes repressed in RasGrf1 KO pancreatic islets (from Additional file 1: Table S1)***
GO:0010975 regulation of neuron projection development	10	0,51	5,08E-02	KLK8, METRN, INPP5J, TNFRSF12A, LIMK1, SEMA3A, CIT, NTN1, NGF, THY1
GO:0010720 positive regulation of cell development	10	0,51	5,08E-02	METRN, DRD2, TNFRSF12A, LIMK1, HOXA11, NKX6-2, BCL2, NTN1, SHH, NGF
GO:0030308 negative regulation of cell growth	10	0,51	5,08E-02	DCBLD2, FOXK1, IL7, BCL2, TRO, TRP63, SEMA3A, TRP73, NTN1, AHSG
GO:0035108 limb morphogenesis	19	0,98	5,10E-02	FGFR2, RARG, TBX3, TBX5, HOXA11, CRABP2, GNA12, PRRX1, LEF1, COL2A1, PRRX2, GLI2, MECOM, SHH, TRP63, FBN2, PITX1, MED1, FGF4
GO:0030595 leukocyte chemotaxis	7	0,36	5,53E-02	PRKCA, IL17B, IL16, CCR1, IFNG, S100A9, IL1B
GO:0060326 cell chemotaxis	7	0,36	5,53E-02	PRKCA, IL17B, IL16, CCR1, IFNG, S100A9, IL1B
GO:0030852 regulation of granulocyte differentiation	3	0,15	5,64E-02	IKZF1, INPP5D, RUNX1

Biological process	***Gene Count***	***Percentage***	***p-Value***	***Genes repressed in RasGrf1 KO pancreatic islets (from Additional file 1: Table S1)***
GO:0021938 smoothened signaling pathway involved in regulation of granule cell precursor cell proliferation	3	0,15	5,64E-02	GLI2, SHH, GLI1
GO:0060737 prostate gland morphogenetic growth	3	0,15	5,64E-02	FGFR2, ESR1, SHH
GO:0060124 positive regulation of growth hormone secretion	3	0,15	5,64E-02	GHRH, DRD2, GHRHR
GO:0001547 antral ovarian follicle growth	3	0,15	5,64E-02	ESR1, FOXO3, BMPR1B
GO:0001764 neuron migration	13	0,67	5,64E-02	SATB2, CDK5R1, CCK, BARHL1, MET, ASZ1, GJA1, VAX1, ESR2, NTN1, ATOH1, NR2F2, DCLK1
GO:0006937 regulation of muscle contraction	9	0,46	5,78E-02	PRKCA, CHRM3, P2RX1, NCF1, CHRNB4, GUCY1A3, ATP1A2, TNNI3, NKX2-5

Biological process	*Gene Count*	*Percentage*	*p-Value*	*Genes repressed in RasGrf1 KO pancreatic islets (from Additional file 1: Table S1)*
GO:0035094 response to nicotine	4	0,21	5,86E-02	CHRNA5, CHRNB4, CHRNA4, HTR2C
GO:0014059 regulation of dopamine secretion	4	0,21	5,86E-02	DRD3, DRD2, CHRNA4, CHRNA6
GO:0045075 regulation of interleukin-12 biosynthetic process	4	0,21	5,86E-02	REL, IFNG, TLR6, LTB
GO:0007271 synaptic transmission, cholinergic	4	0,21	5,86E-02	LYNX1, CHRM3, ADORA2A, CHRNA4
GO:0045078 positive regulation of interferon-gamma biosynthetic process	4	0,21	5,86E-02	IL21, TLR7, CD27, TLR9
GO:0050819 negative regulation of coagulation	4	0,21	5,86E-02	PROCR, KLKB1, PROC, ANXA2
GO:0002062 chondrocyte differentiation	6	0,31	6,02E-02	PRKCA, FGF18, PTH1R, COL2A1, COL11A2, COL11A1

Biological process	***Gene Count***	***Percentage***	***p-Value***	***Genes repressed in RasGrf1 KO pancreatic islets (from Additional file 1: Table S1)***
GO:0042446<br>hormone biosynthetic process	6	0,31	6,02E-02	CYP17A1, HSD17B1, CYP11B2, TPO, SRD5A2, BMPR1B
GO:0002253<br>activation of immune response	15	0,77	6,02E-02	PTPN6, C4B, C3, CD247, NFKBIA, IGH-6, TLR4, NFAM1, THY1, IGHG, CD19, C4BP, SH2B2, C2, CFD
GO:0002819<br>regulation of adaptive immune response	11	0,57	6,15E-02	IGHG, PTPN6, FCER2A, TNFSF13B, FCGR2B, C3, FOXJ1, IFNG, IDO1, CD40, FCGR1
GO:0019221<br>cytokine-mediated signaling pathway	11	0,57	6,15E-02	IRAK4, TNFRSF1A, PTPN6, STAT4, IL2RB, TNF, CCL2, IL6ST, GAB1, IL1B, SH2B2
GO:0002822<br>regulation of adaptive immune response based on somatic recombination of immune receptors built from immunoglobulin superfamily domains	11	0,57	6,15E-02	IGHG, PTPN6, FCER2A, TNFSF13B, FCGR2B, C3, FOXJ1, IFNG, IDO1, CD40, FCGR1

Biological process	***Gene Count***	***Percentage***	***p-Value***	***Genes repressed in RasGrf1 KO pancreatic islets (from Additional file 1: Table S1)***
GO:0002521 leukocyte differentiation	22	1,13	6,23E-02	CALCR, TNF, IKZF1, IL7, RELB, IL25, RAG1, EOMES, SOX4, SFPI1, NFAM1, MYH9, POU1F1, CBFA2T3, TNFSF11, CD40LG, JMJD6, CLCF1, BCL11B, BCL2, POU2F2, NKX2-3
GO:0050772 positive regulation of axonogenesis	5	0,26	6,28E-02	METRN, TNFRSF12A, LIMK1, NTN1, NGF
GO:0030010 establishment of cell polarity	5	0,26	6,28E-02	FOXF1A, FOXJ1, CRB3, MYH9, SHH
GO:0048762 mesenchymal cell differentiation	10	0,51	6,36E-02	FGFR2, EDNRB, NOG, OVOL2, BCL2, EOMES, LEF1, CYP26A1, SHH, HNRNPAB
GO:0030336 negative regulation of cell migration	7	0,36	6,45E-02	DRD2, DLL4, BCL2, TBX5, SEMA3A, SHH, THY1
GO:0046660 female sex differentiation	12	0,62	6,49E-02	PGR, AFP, TBX3, BCL2, TRP63, ESR1, FOXO3, ESR2, SPO11, BMPR1B, MMP14, MED1
GO:0048704 embryonic skeletal system morphogenesis	12	0,62	6,49E-02	HOXB4, DLX2, SATB2, HOXA4, HOXA5, HOXB5, HOXB6, HOXA7, PRRX1, SIX4, PRRX2, COL11A1

Biological process	*Gene Count*	*Percentage*	*p-Value*	*Genes repressed in RasGrf1 KO pancreatic islets (from Additional file 1: Table S1)*
GO:0050672 negative regulation of lymphocyte proliferation	8	0,41	6,57E-02	PTPN6, FCGR2B, FOXJ1, ERBB2, TNFRSF14, IDO1, INPP5D, SHH
GO:0070664 negative regulation of leukocyte proliferation	8	0,41	6,57E-02	PTPN6, FCGR2B, FOXJ1, ERBB2, TNFRSF14, IDO1, INPP5D, SHH
GO:0032945 negative regulation of mononuclear cell proliferation	8	0,41	6,57E-02	PTPN6, FCGR2B, FOXJ1, ERBB2, TNFRSF14, IDO1, INPP5D, SHH
GO:0021549 cerebellum development	8	0,41	6,57E-02	LHX1, ATG7, LHX5, LMX1A, RORA, GLI2, KLHL1, GLI1
GO:0050727 regulation of inflammatory response	11	0,57	6,80E-02	PRKCA, TNFRSF1A, IGHG, TNFRSF1B, FCGR2B, C3, ADORA2A, AOAH, IDO1, FCGR1, AHSG
GO:0031349 positive regulation of defense response	11	0,57	6,80E-02	PRKCA, TNFRSF1A, IGHG, C3, NFKBIA, TLR4, IDO1, IL21, KLRB1C, FCGR1, POLR3D

Biological process	*Gene Count*	*Percentage*	*p-Value*	*Genes repressed in RasGrf1 KO pancreatic islets (from Additional file 1: Table S1)*
GO:0046545 development of primary female sexual characteristics	11	0,57	6,80E-02	PGR, AFP, TBX3, BCL2, TRP63, ESR1, FOXO3, ESR2, SPO11, BMPR1B, MMP14
GO:0008406 gonad development	15	0,77	7,04E-02	DHH, ESR1, FOXO3, SPO11, ESR2, NR0B1, MMP14, AHSG, PGR, AFP, BCL2, BIK, HSD17B4, KLHL10, BMPR1B
GO:0009310 amine catabolic process	12	0,62	7,11E-02	BCAT1, GAD2, FTCD, PRODH2, MOXD2, UROC1, NOS2, IDO1, DBH, SARDH, GLDC, AUH
GO:0002706 regulation of lymphocyte mediated immunity	12	0,62	7,11E-02	IGHG, PTPN6, FCER2A, FCGR2B, C3, FOXJ1, IFNG, CD40, KLRB1B, IL21, KLRB1C, FCGR1
GO:0030099 myeloid cell differentiation	16	0,82	7,12E-02	CALCR, TNF, CCR1, RELB, IL25, SFPI1, MYH9, CBFA2T3, TRIM10, TIMP1, HBA-A1, ANK1, TNFSF11, JMJD6, KLF1, NKX2-3
GO:0048538 thymus development	6	0,31	7,14E-02	IKZF1, BCL2, BCL11B, PRDX2, SIX4, FGF3
GO:0002053 positive regulation of mesenchymal cell proliferation	6	0,31	7,14E-02	FGFR2, TRP63, PRRX1, PRRX2, TBX18, SHH

Biological process	***Gene Count***	***Percentage***	***p-Value***	***Genes repressed in RasGrf1 KO pancreatic islets (from Additional file 1: Table S1)***
GO:0009306<br>protein secretion	6	0,31	7,14E-02	PLEK, TNFSF13B, CD40LG, POU2F2, CD27, LCP2
GO:0051346<br>negative regulation of hydrolase activity	6	0,31	7,14E-02	APOA1, NOS1, ADORA2A, RAG1, ANGPTL4, DHCR24
GO:0007243<br>protein kinase cascade	33	1,70	7,19E-02	NRG3, FGFR3, TNF, GNA12, STK17B, CSPG4, MAP4K1, PRDX2, TLR4, TLR6, MAP3K7, CLCF1, IFNG, PRRT1, GAB1, ADRA2B, FGF2, PRL, MAP2K7, PRKCA, PLEK, MET, IGH-6, PDE6H, AGER, PDE6G, MARK1, MAP4K5, MAST1, MAPK13, CARTPT, GADD45B, DUSP9
GO:0015674<br>di-, tri-valent inorganic cation transport	24	1,24	7,27E-02	SLC8A3, TRPC2, ORAI1, SLC8A2, CACHD1, CACNG8, CACNG7, TRPV2, CACNG6, CACNB1, GIF, CACNG2, CACNA1S, CACNA2D2, 1300017J02RIK, TPCN1, TPCN2, TMEM37, BSPRY, CATSPER2, TRPV4, CHRNA4, CACNA1F, SCARA5
GO:0001570<br>vasculogenesis	9	0,46	7,31E-02	FOXF1A, FOXM1, MYO1E, ZFPM2, SOX17, TNNI3, NKX2-5, EPHA2, SHH
GO:0046883<br>regulation of hormone secretion	8	0,41	7,46E-02	SSTR5, OIT1, GHRH, DRD2, IL1B, FGF23, GHRHR, PSMD9
GO:0032102<br>negative regulation of response to external stimulus	8	0,41	7,46E-02	KLK8, TNFRSF1B, FCGR2B, ADORA2A, DRD2, AOAH, GRID2, CARTPT

Biological process	*Gene Count*	*Percentage*	*p-Value*	*Genes repressed in RasGrf1 KO pancreatic islets (from Additional file 1: Table S1)*
GO:0051222 positive regulation of protein transport	8	0,41	7,46E-02	TNF, ADORA2A, IFNG, EDAR, EDA, LTB, LTA, SHH
GO:0045619 regulation of lymphocyte differentiation	11	0,57	7,48E-02	PTPN6, IKZF1, IL7, FOXJ1, ERBB2, PRDX2, NFAM1, INPP5D, IL21, CD27, SHH
GO:0046395 carboxylic acid catabolic process	14	0,72	7,49E-02	BCAT1, ACOX2, FTCD, MOXD2, IDO1, DBH, GLDC, AUH, GAD2, PRODH2, HSD17B4, UROC1, NOS2, SARDH
GO:0035113 embryonic appendage morphogenesis	16	0,82	7,65E-02	RARG, TBX3, TBX5, HOXA11, GNA12, CRABP2, PRRX1, LEF1, PRRX2, GLI2, MECOM, SHH, TRP63, FBN2, MED1, FGF4
GO:0030326 embryonic limb morphogenesis	16	0,82	7,65E-02	RARG, TBX3, TBX5, HOXA11, GNA12, CRABP2, PRRX1, LEF1, PRRX2, GLI2, MECOM, SHH, TRP63, FBN2, MED1, FGF4
GO:0031644 regulation of neurological system process	18	0,93	7,66E-02	KLK8, TNF, GRIK1, DRD2, ADORA2A, NCS1, PARK2, SYNGR1, CACNA2D2, LAMA2, SLC1A3, IFNG, DLG4, CARTPT, HTR2C, LTB, LTA, NGF

Biological process	***Gene Count***	***Percentage***	***p-Value***	***Genes repressed in RasGrf1 KO pancreatic islets (from Additional file 1: Table S1)***
GO:0001836 release of cytochrome c from mitochondria	5	0,26	7,71E-02	BID, GGCT, BCL2, PMAIP1, TRP73
GO:0046330 positive regulation of JNK cascade	5	0,26	7,71E-02	MAP3K7, TNF, IL1B, TLR4, CD27
GO:0001502 cartilage condensation	5	0,26	7,71E-02	BARX2, COL2A1, BMPR1B, COL11A1, FGF4
GO:0048821 erythrocyte development	5	0,26	7,71E-02	HBA-A1, ANK1, JMJD6, KLF1, TIMP1
GO:0070304 positive regulation of stress-activated protein kinase signaling pathway	5	0,26	7,71E-02	MAP3K7, TNF, IL1B, TLR4, CD27
GO:0060602 branch elongation of an epithelium	4	0,21	7,74E-02	FGFR2, ESR1, SHH, MED1

Biological process	***Gene Count***	***Percentage***	***p-Value***	***Genes repressed in RasGrf1 KO pancreatic islets (from Additional file 1: Table S1)***
GO:0046626<br>regulation of insulin receptor signaling pathway	4	0,21	7,74E-02	PRKCA, IL1B, PRKCD, AHSG
GO:0030810<br>positive regulation of nucleotide biosynthetic process	4	0,21	7,74E-02	PTHLH, GHRH, ADORA2A, GUCY1A3
GO:0045637<br>regulation of myeloid cell differentiation	10	0,51	7,84E-02	TNF, TNFSF11, IKZF1, CSF1, CARTPT, NFKBIA, FOXO3, INPP5D, RUNX1, TOB2
GO:0050766<br>positive regulation of phagocytosis	6	0,31	8,38E-02	IGHG, FCGR2B, C3, PTX3, FCGR1, AHSG
GO:0021782<br>glial cell development	6	0,31	8,38E-02	PLP1, ADORA2A, NKX6-2, PHGDH, EIF2B1, SHH
GO:0010464<br>regulation of mesenchymal cell proliferation	6	0,31	8,38E-02	FGFR2, TRP63, PRRX1, PRRX2, TBX18, SHH

Biological process	*Gene Count*	*Percentage*	*p-Value*	*Genes repressed in RasGrf1 KO pancreatic islets (from Additional file 1: Table S1)*
GO:0016126 sterol biosynthetic process	7	0,36	8,53E-02	CYB5R3, APOA1, MVD, HMGCR, MVK, HSD17B7, DHCR24
GO:0051250 negative regulation of lymphocyte activation	10	0,51	8,65E-02	PTPN6, FCGR2B, ADORA2A, FOXJ1, ERBB2, TNFRSF14, PRDX2, IDO1, INPP5D, SHH
GO:0045348 positive regulation of MHC class II biosynthetic process	3	0,15	8,75E-02	CIITA, IFNG, TLR4
GO:0043586 tongue development	3	0,15	8,75E-02	NKX2-6, NKX2-5, SHH
GO:0046851 negative regulation of bone remodeling	3	0,15	8,75E-02	IAPP, CARTPT, INPP5D
GO:0051590 positive regulation of neurotransmitter transport	3	0,15	8,75E-02	ADORA2A, DRD2, HTR2C
GO:0048636 positive regulation of muscle development	3	0,15	8,75E-02	BCL2, GJA1, SHH

Biological process	*Gene Count*	*Percentage*	*p-Value*	*Genes repressed in RasGrf1 KO pancreatic islets (from Additional file 1: Table S1)*
GO:0060123 regulation of growth hormone secretion	3	0,15	8,75E-02	GHRH, DRD2, GHRHR
GO:0060662 salivary gland cavitation	3	0,15	8,75E-02	EDAR, EDA, SHH
GO:0045683 negative regulation of epidermis development	3	0,15	8,75E-02	HOXA7, TRP63, NGFR
GO:0045844 positive regulation of striated muscle development	3	0,15	8,75E-02	BCL2, GJA1, SHH
GO:0032922 circadian regulation of gene expression	3	0,15	8,75E-02	DRD3, DRD2, CARTPT
GO:0030859 polarized epithelial cell differentiation	3	0,15	8,75E-02	FOXF1A, TRP63, CRB3

Biological process	***Gene Count***	***Percentage***	***p-Value***	***Genes repressed in RasGrf1 KO pancreatic islets (from Additional file 1: Table S1)***
GO:0045779 negative regulation of bone resorption	3	0,15	8,75E-02	IAPP, CARTPT, INPP5D
GO:0060605 tube lumen cavitation	3	0,15	8,75E-02	EDAR, EDA, SHH
GO:0051890 regulation of cardioblast differentiation	3	0,15	8,75E-02	TBX5, GATA4, NKX2-5
GO:0051891 positive regulation of cardioblast differentiation	3	0,15	8,75E-02	TBX5, GATA4, NKX2-5
GO:0019220 regulation of phosphate metabolic process	40	2,06	8,87E-02	TNF, FGFR3, ADORA2A, HMGCR, CSF1, 4930403L05RIK, CSPG4, PRDX2, TLR4, TLR6, APOA1, INPP5K, INPP5J, BCL2, IFNG, GAB1, ARR3, MLLT1, IL1B, MLST8, FGF2, CEACAM1, PRKCA, EGFR, PTPN6, SPHK1, MET, TNFRSF14, IGH-6, PDE6H, PDE6G, PRKCD, THY1, CDKN1A, PDGFRB, CARTPT, TRP73, BMP7, DUSP9, GADD45B
GO:0051174 regulation of phosphorus metabolic process	40	2,06	8,87E-02	TNF, FGFR3, ADORA2A, HMGCR, CSF1, 4930403L05RIK, CSPG4, PRDX2, TLR4, TLR6, APOA1, INPP5K, INPP5J, BCL2, IFNG, GAB1, ARR3, MLLT1, IL1B, MLST8, FGF2, CEACAM1, PRKCA, EGFR, PTPN6, SPHK1, MET, TNFRSF14, IGH-6, PDE6H, PDE6G, PRKCD, THY1, CDKN1A, PDGFRB, CARTPT, TRP73, BMP7, DUSP9, GADD45B
GO:0046328 regulation of JNK cascade	9	0,46	9,07E-02	MAP3K7, SH3RF1, TNF, GAB1, IL1B, TLR4, TRP73, TLR6, CD27

Biological process	*Gene Count*	*Percentage*	*p-Value*	*Genes repressed in RasGrf1 KO pancreatic islets (from Additional file 1: Table S1)*
GO:0043068 positive regulation of programmed cell death	34	1,75	9,11E-02	BID, CDK5R1, FGFR3, TNF, ADORA2A, STK17B, FOXO3, PMAIP1, ITM2B, CASP6, TRP63, DIABLO, INPP5D, LTB, CD27, LTA, COL18A1, PRKCA, PTPN6, RARG, TBX5, ESR2, IDO1, SAP30BP, PRKCD, FCGR1, JMY, TRADD, CDKN1A, EPHA7, BIK, NGFR, BMP7, AY074887
GO:0007339 binding of sperm to zona pellucida	5	0,26	9,28E-02	ACR, ADAM3, ZP3R, ADAM1A, ZAN
GO:0050863 regulation of T cell activation	17	0,88	9,34E-02	PTPN6, SIT1, IKZF1, IL7, FOXJ1, ADORA2A, ERBB2, PRDX2, TNFRSF14, IDO1, SHH, THY1, PRKCQ, TNFSF13B, IFNG, IL12A, CD27
GO:0030334 regulation of cell migration	15	0,77	9,41E-02	COL18A1, PDPN, DRD2, TBX5, CSF1, ITGB3, NTN1, SHH, THY1, LAMA2, BCL2, DLL4, GAB1, SEMA3A, ARHGAP8
GO:0030278 regulation of ossification	10	0,51	9,50E-02	CALCR, TWSG1, WNT7B, ENPP1, BCL2, CSF1, FGF23, CHRD, TOB2, AHSG
GO:0021543 pallium development	10	0,51	9,50E-02	EGFR, DLX2, DLX1, WNT3A, ATG7, LEF1, LMX1A, TRP73, TACC3, AHSG
GO:0002695 negative regulation of leukocyte activation	10	0,51	9,50E-02	PTPN6, FCGR2B, ADORA2A, FOXJ1, ERBB2, TNFRSF14, PRDX2, IDO1, INPP5D, SHH

Biological process	*Gene Count*	*Percentage*	*p-Value*	*Genes repressed in RasGrf1 KO pancreatic islets (from Additional file 1: Table S1)*
GO:0050866 negative regulation of cell activation	10	0,51	9,50E-02	PTPN6, FCGR2B, ADORA2A, FOXJ1, ERBB2, TNFRSF14, PRDX2, IDO1, INPP5D, SHH
GO:0007163 establishment or maintenance of cell polarity	7	0,36	9,69E-02	FOXF1A, CD3G, FOXJ1, CRB3, CLASP2, MYH9, SHH
GO:0021766 hippocampus development	6	0,31	9,72E-02	DLX2, DLX1, WNT3A, LEF1, LMX1A, TRP73
GO:0050729 positive regulation of inflammatory response	6	0,31	9,72E-02	PRKCA, TNFRSF1A, IGHG, C3, IDO1, FCGR1
GO:0030834 regulation of actin filament depolymerization	6	0,31	9,72E-02	LIMA1, PLEK, SPNB2, SPNB1, CAPZB, EPB4,9
GO:0006941 striated muscle contraction	6	0,31	9,72E-02	NOS1, MYH7, TNNI3, HOMER1, CACNA1S, MYH8

Biological process	*Gene Count*	*Percentage*	*p-Value*	*Genes repressed in RasGrf1 KO pancreatic islets (from Additional file 1: Table S1)*
GO:0008277 regulation of G-protein coupled receptor protein signaling pathway	6	0,31	9,72E-02	DRD3, DRD2, RIC8, PDE6H, PDE6G, RGS14
GO:0045072 regulation of interferon-gamma biosynthetic process	4	0,21	9,85E-02	IL21, TLR7, CD27, TLR9
GO:0055024 regulation of cardiac muscle tissue development	4	0,21	9,85E-02	FGFR2, NOG, TBX5, GATA4
GO:0030903 notochord development	4	0,21	9,85E-02	NOG, GLI2, EPHA2, GLI1
GO:0001738 morphogenesis of a polarized epithelium	4	0,21	9,85E-02	FOXF1A, TRP63, CRB3, FZD6
GO:0002793 positive regulation of peptide secretion	4	0,21	9,85E-02	GHRH, DRD2, GHRHR, PSMD9

Biological process	*Gene Count*	*Percentage*	*p-Value*	*Genes repressed in RasGrf1 KO pancreatic islets (from Additional file 1: Table S1)*
GO:0055021 regulation of cardiac muscle growth	4	0,21	9,85E-02	FGFR2, NOG, TBX5, GATA4
GO:0060043 regulation of cardiac muscle cell proliferation	4	0,21	9,85E-02	FGFR2, NOG, TBX5, GATA4
GO:0009261 ribonucleotide catabolic process	4	0,21	9,85E-02	ENTPD7, MYH7, ENTPD1, CANT1
GO:0009075 histidine family amino acid metabolic process	4	0,21	9,85E-02	FTCD, MOXD2, UROC1, DBH
GO:0010942 positive regulation of cell death	34	1,75	9,88E-02	BID, CDK5R1, FGFR3, TNF, ADORA2A, STK17B, FOXO3, PMAIP1, ITM2B, CASP6, TRP63, DIABLO, INPP5D, LTB, CD27, LTA, COL18A1, PRKCA, PTPN6, RARG, TBX5, ESR2, IDO1, SAP30BP, PRKCD, FCGR1, JMY, TRADD, CDKN1A, EPHA7, BIK, NGFR, BMP7, AY074887
GO:0006935 chemotaxis	17	0,88	9,95E-02	PRKCA, CCL2, IL16, CREB3, CCR1, S100A9, CCL4, CCL6, LSP1, CCR8, IL17B, CCL20, IFNG, CCR10, IL1B, CMTM7, SEMA3A
