# Supplementary material for: Transcriptional profiling reveals functional links between RasGrf1 and Pttg1 in pancreatic beta cells
Source: BMC Genomics. 2014 Nov 25;15:1019. doi: 10.1186/1471-2164-15-1019 (PMC4301450; doi:10.1186/1471-2164-15-1019)
Supplement: Supplementary file 4 — Additional file 4: Table S2B: Functional annotation of upregulated, differentially expressed genes in pancreatic islets of RasGrf1 knockout mice. The DAVID functional annotation tool (http://david.abcc.ncifcrf.gov/) was used to identify statistically significant functional associations (p-value <0.1) linking particular gene subsets contained within the list of induced loci occurring in RasGrf1 KO pancreatic islets (Additional file 1: Table S1, FDR=0.084) to specific Gene Ontology (GO) terms. The column labelled “Biological process” identifies the functional GO terms (level 5) recognized in each case for the corresponding groups of loci listed under the column labeled “Genes induced in RasGrf1 KO pancreatic islets (from Additional file 1 : Table S1)”. The column labeled “Gene Count” indicates the specific number of genes annotated by DAVID to the indicated GO functionality within the list of induced genes included in Table S1 (Additional file 1). The values under the column “Percentage” are calculated by referring the “Gene Count” numbers to the total number of probesets recognized by DAVID (1781, out of a total 1799) within that list. The column labeled “p-value” refers to the statistical significance of the functional associations identified, and contains p-values calculated using the Hypergeometric Distribution and subsequently corrected by implementing the False Discovery Rate (FDR) method [90]. (PDF 312 KB) [file 12864_2014_6838_MOESM4_ESM.pdf]

Table S2B. Functional annotation of the up-regulated, differentially expressed genes in pancreatic islets of RasGrf1 knockout mice.

The DAVID functional annotation tool (http://david.abcc.ncifcrf.gov/) was used to identify statistically significant functional associations (p-value <0.1) linking particular gene subsets contained within the list of induced loci occurring in RasGrf1 KO pancreatic islets (Additional file 1: Table S1, FDR=0.084) to specific Gene Ontology (GO) terms.

The column labelled "*Biological process*" identifies the functional GO terms (level 5) recognized in each case for the corresponding groups of loci listed under the column labelled "*Genes induced in RasGrf1 KO pancreatic islets (from Additional file 1: Table S1)*. The column labeled "*Gene Count*" indicates the specific number of loci annotated by DAVID to the indicated GO functionality within the list of induced genes included in Additional file 1: Table S1. The values under the column "*Percentage*" are calculated by referring the "*Gene Count*" numbers to the total number of genes recognized by DAVID (1781, out of a total 1799) within that list. The column labeled "*p-value*" refers to the statistical significance of the functional associations identified, and contains p-values calculated using the Hypergeometric Distribution and subsequently corrected by implementing the False Discovery Rate (FDR) method of Hochberg and Benjamini (Hochberg and Benjamini, 1990).

Biological process	***Gene Count***	***Percentage***	***p-Value***	***Genes induced in RasGrf1 KO pancreatic islets (from Additional file 1: Table S1)***
GO:0044267<br>cellular protein metabolic process	314	17,63	3,63E-17	PPP2R5C, RPLP2, CCT3, CLK1, CD2AP, ERLEC1, ZNRF2, CUL3, EPC1, MAP3K4, DNAJB11, CLK2, RPLP0, PIK3C3, RPLP1, ILK, CLK4, PIK3CA, TLK2, OGT, PTPRJ, BCR, PTPRF, ROCK1, ANAPC5, ROCK2, AARS, PTPRR, MECP2, UBR4, DPAGT1, BRAP, NKTR, MAPK1, MGAT2, RPS19, BAZ1B, HUWE1, MAPK6, UBR5, BACE2, EIF2S2, NEK9, MAPK8, PIAS2, PIAS1, EEFSEC, NEK7, SUV420H1, RAD23B, RAB6, CHEK1, PXK, CALR, MTIF2, ARIH1, DUSP19, DUSP16, FBXO6, FBXO3, FBXO9, RPS24, YEATS4, MAP2K1, LCMT2, TGFBR1, TRIO, RPS9, CDC23, EPRS, PCNP, UBE2L3, ATM, PTPN12, CCT7, TULP4, UBA1, RNF2, UBA2, CCT8, PARP1, NARS, UBE2G1, RLIM, PTEN, CANX, P4HA1, MAP1LC3B, SLMAP, 6530401D17RIK, TGFA, BRD4, FBXL14, BRD8, ZDHHC2, SRPK2, EIF2S3X, EIF2S3Y, UFD1L, HERC4, PKDCC, EIF1B, DGUOK, CCT6A, HERC1, RBBP6, SENP2, PJA2, WDR48, PJA1, PDIK1L, MAST2, RPL41, ADAM17, USP20, CAND1, CDK11B, SIAH2, GANAB, RAB3D, ABI1, FKBP1A, STT3B, SUMO3, SUMO1, GPHN, GALNT10, SNRK, MORC3, SEPSECS, BAZ2A, QSOX1, TRIP12, TES, C80913, CSNK1A1, SPSB1, LRRC41, TRIM28, TRIM23, ETF1, MARCH5, PHF17, PSMD14, DUSP1, CSNK1E, RPS6KA2, PTP4A2, USP47, USP48, RBM14, USP45, DUSP6, MKRN1, ILKAP, PRPF4B, NRBP1, STK38, TBK1, CAPZA2, EIF5, EIF5B, EIF5A, SAE1, CNOT4, TGFB2, KDM1A, MLL5, ATG5, RABGEF1, DNAJC5, EIF1, PDRG1, ITCH, ASPH, RPN2, RNF149, FBXO21, DNAJC1, MYST4, SATB1, DARS, STK24, EEF2, MAP4K3, SUZ12, DCUN1D1, KDM2B, PSMA6, RIOK3, PCMTD2, PCMTD1, ASB3, RNF20, STK16, DERL1, UBE2V2, TRRAP, SKP1A, RBX1, PSMB5, EIF3C, EIF3D, PTK2, PSMB7, UBE2D3, EIF3A, EIF3B, OTUD7B, FBXW2, RNF11, RNF167, ERO1L, B3GNT2, DNAJA4, RNF14, HECTD1, P4HB, RYK, DENR, EHMT2, EIF4B, FBXO38, NMT2, HDAC3, NOSIP, PSMC5, HDAC2, DNAJB9, VCP, AKTIP, ULK1, PRKAR1B, ULK2, HSPD1, IKBKB, UBE2E2, HSP90AB1, MAN1B1, CAD, OS9, TOR3A, PRMT1, PIGM, SLK, PAK2, PAK3, PRMT5, PIGC, CDK16, CDK14, NFX1, PIGA, IRAK1, TRPM7, PIGX, SARS, CDK8, PRKCI, TOPORS, PIGQ, DAPK1, GAK, UBE2N, RFWD2, PRKD1, EIF4G2, ACVR2A, PPM1G, TARS, EIF4G3, BTG2, HIPK1, UBE2K, HIPK3, HIPK2, CD81, UBC, UCHL5, UBB, UBE2S, ACVR1, FKBP2, GALNT2, FKBP9, UBE3B, USP9X, TIPARP, PPM1A, UBE3C, PPM1B, 2700078E11RIK, SEC63, IARS, MAP3K3, SH3GLB1, TOR1B, PIK3R4, HSPA8, PIK3R1, STAMBP, KAT2B, FLT1, UBE4A, CREB1, PTPRA, WSB2, GSPT2, ZRANB1, JAK1, CUL4B, TBL1X, RNF41, F2R, BMPR1A

Biological process	*Gene Count*	*Percentage*	*p-Value*	*Genes induced in RasGrf1 KO pancreatic islets (from Additional file 1: Table S1)*
GO:0015031 protein transport	129	7,24	4,11E-15	XPO1, VPS54, USE1, EIF5A, RABGEF1, SCAMP1, MYO6, SCAMP2, GOLT1B, NUPL1, LYST, PEX26, RAB14, PDCD6IP, VPS26B, RAB10, CHMP2A, ARFGAP1, ARL6IP1, SNX9, AFTPH, DERL1, STX7, SNX5, STAM2, RAB6, AHCTF1, SNX4, CCDC91, NAPA, LMAN2, SNX3, LMAN1, MIA3, MACF1, STX18, GORASP1, FOLR1, TMED10, DOPEY2, SEC61A1, AP2M1, RABIF, LIN7C, EPS15, RABEP1, AKTIP, VCP, ARF1, ARF4, TSC2, SYTL4, GOSR1, XPO7, VPS37A, AP3S2, NBEA, RAB3IP, PEX7, COPB2, AP2B1, SRPR, SEH1L, COPB1, GOPC, NECAP1, RHOB, SEC24C, KPNB1, SEC24D, KDELR1, NSF, AP3B1, STX6, SEC23A, NUP88, RAB4A, STXBP1, IPO8, M6PR, MFN2, SENP2, CHMP1A, IPO4, IPO5, KPNA6, KPNA4, COPG, KPNA3, H47, SNX18, YWHAZ, PLDN, NUP160, RAB3D, EXOC8, BET1, SEC63, SEC16B, TMED3, DDX19A, AP3M1, SEC22B, AP3D1, RAB11A, SCG5, SNAP23, APPBP2, PPP3CA, VPS39, TNPO3, RAB2A, GDI1, RAB2B, GDI2, RRBP1, YWHAB, YWHAE, AP2A2, YWHAG, COG6, YWHAH, COG8, RAB34, RAB22A, NOP58, YKT6, ARAP1, SSR3
GO:0043632 modification-dependent macromolecule catabolic process	96	5,39	3,27E-10	MKRN1, PPP2R5C, SAE1, CD2AP, ERLEC1, CNOT4, ZNRF2, CUL3, RABGEF1, RNF149, ITCH, FBXO21, ANAPC5, UBR4, BRAP, DCUN1D1, KDM2B, HUWE1, PSMA6, UBR5, PIAS2, ASB3, PIAS1, RNF20, RAD23B, DERL1, UBE2V2, SKP1A, RBX1, PSMB5, ARIH1, UBE2D3, FBXO6, FBXW2, RNF11, RNF167, FBXO3, FBXO9, RNF14, HECTD1, CDC23, PCNP, UBE2L3, FBXO38, TULP4, PSMC5, VCP, UBA1, UBA2, RNF2, UBE2E2, UBE2G1, MAN1B1, RLIM, OS9, MAP1LC3B, FBXL14, NFX1, UFD1L, HERC4, TOPORS, HERC1, SENP2, WDR48, PJA2, RFWD2, UBE2N, PJA1, UBE2K, UCHL5, UBC, USP20, CAND1, UBB, SIAH2, UBE2S, UBE3B, USP9X, UBE3C, 2700078E11RIK, SUMO3, SUMO1, TRIP12, STAMBP, SPSB1, UBE4A, LRRC41, MARCH5, PSMD14, WSB2, USP47, ZRANB1, CUL4B, USP48, TBL1X, USP45, RNF41
GO:0044257 cellular protein catabolic process	99	5,56	6,69E-10	MKRN1, PPP2R5C, SAE1, CD2AP, ERLEC1, CNOT4, ZNRF2, CUL3, RABGEF1, RNF149, ITCH, FBXO21, ANAPC5, UBR4, BRAP, DCUN1D1, KDM2B, HUWE1, PSMA6, BACE2, UBR5, ASB3, PIAS2, PIAS1, RNF20, RAD23B, DERL1, UBE2V2, SKP1A, RBX1, PSMB5, ARIH1, UBE2D3, PSMB7, FBXO6, RNF11, FBXW2, FBXO3, RNF167, FBXO9, RNF14, HECTD1, CDC23, PCNP, UBE2L3, FBXO38, TULP4, PSMC5, VCP, UBA1, UBA2, RNF2, UBE2E2, UBE2G1, MAN1B1, RLIM, OS9, MAP1LC3B, FBXL14, NFX1, UFD1L, HERC4, TOPORS, HERC1, RFWD2, PJA2, WDR48, SENP2, UBE2N, PJA1, UBE2K, UCHL5, UBC, USP20, ADAM17, CAND1, SIAH2, UBB, UBE2S, UBE3B, USP9X, UBE3C, 2700078E11RIK, SUMO3, SUMO1, TRIP12, STAMBP, UBE4A, SPSB1, LRRC41, MARCH5, PSMD14, WSB2, USP47, ZRANB1, CUL4B, USP48, TBL1X, USP45, RNF41
GO:0030163 protein catabolic process	101	5,67	1,02E-09	MKRN1, PPP2R5C, USE1, SAE1, CD2AP, ERLEC1, CNOT4, ZNRF2, CUL3, RABGEF1, RNF149, ITCH, FBXO21, ANAPC5, UBR4, BRAP, DCUN1D1, KDM2B, HUWE1, PSMA6, BACE2, UBR5, ASB3, PIAS2, PIAS1, RNF20, RAD23B, DERL1, UBE2V2, SKP1A, RBX1, PSMB5, ARIH1, UBE2D3, PSMB7, FBXO6, RNF11, FBXW2, FBXO3, RNF167, FBXO9, RNF14, HECTD1, CDC23, PCNP, UBE2L3, FBXO38, TULP4, PSMC5, VCP, UBA1, UBA2, RNF2, PSMC1, UBE2E2, UBE2G1, MAN1B1, RLIM, OS9, MAP1LC3B, FBXL14, NFX1, UFD1L, HERC4, TOPORS, HERC1, RFWD2, PJA2, WDR48, SENP2, UBE2N, PJA1, UBE2K, UCHL5, UBC, USP20, ADAM17, CAND1, SIAH2, UBB, UBE2S, UBE3B, USP9X, UBE3C, 2700078E11RIK, SUMO3, SUMO1, TRIP12, STAMBP, UBE4A, SPSB1, LRRC41, MARCH5, PSMD14, WSB2, USP47, ZRANB1, CUL4B, USP48, TBL1X, USP45, RNF41

Biological process	*Gene Count*	*Percentage*	*p-Value*	*Genes induced in RasGrf1 KO pancreatic islets (from Additional file 1: Table S1)*
GO:0006464<br>protein modification process	188	10,56	1,41E-09	ILKAP, PRPF4B, NRBP1, STK38, TBK1, CAPZA2, EIF5A, SAE1, CLK1, TGFB2, KDM1A, EPC1, MLL5, MAP3K4, ATG5, CLK2, CLK4, ILK, PIK3C3, PIK3CA, OGT, ITCH, TLK2, RPN2, ASPH, MYST4, PTPRJ, SATB1, BCR, ROCK1, PTPRF, STK24, ROCK2, PTPRR, MECP2, DPAGT1, SUZ12, MAP4K3, MAPK1, MGAT2, HUWE1, MAPK6, BAZ1B, RIOK3, UBR5, PCMTD2, PCMTD1, NEK9, PIAS2, MAPK8, SUV420H1, NEK7, STK16, RAB6, CHEK1, UBE2V2, PXK, TRRAP, RBX1, PTK2, UBE2D3, DUSP19, DUSP16, OTUD7B, B3GNT2, ERO1L, HECTD1, P4HB, YEATS4, MAP2K1, RYK, TGFBR1, LCMT2, TRIO, PCNP, UBE2L3, EHMT2, ATM, PTPN12, NMT2, NOSIP, HDAC3, HDAC2, AKTIP, UBA1, ULK1, RNF2, ULK2, UBA2, PRKAR1B, IKBKB, PARP1, UBE2E2, UBE2G1, CAD, PTEN, OS9, PRMT1, PIGM, SLK, PAK2, PAK3, P4HA1, PRMT5, TGFA, PIGC, BRD4, CDK16, CDK14, PIGA, BRD8, ZDHHC2, IRAK1, SRPK2, TRPM7, PIGX, PRKCI, CDK8, HERC4, PKDCC, DGUOK, HERC1, PIGQ, RBBP6, DAPK1, GAK, UBE2N, PRKD1, SENP2, WDR48, PPM1G, PDIK1L, ACVR2A, MAST2, HIPK1, BTG2, UBE2K, HIPK3, CD81, HIPK2, CAND1, CDK11B, SIAH2, UBE2S, ACVR1, GALNT2, GANAB, UBE3B, RAB3D, USP9X, TIPARP, PPM1A, ABI1, UBE3C, PPM1B, SUMO3, STT3B, SUMO1, GALNT10, SNRK, MAP3K3, MORC3, PIK3R4, QSOX1, BAZ2A, PIK3R1, TRIP12, TES, CSNK1A1, FLT1, KAT2B, UBE4A, CREB1, PTPRA, TRIM28, TRIM23, ETF1, PHF17, DUSP1, CSNK1E, RPS6KA2, PTP4A2, JAK1, RBM14, RNF41, F2R, DUSP6, BMPR1A
GO:0006886<br>intracellular protein transport	59	3,31	1,51E-08	XPO1, AP3S2, NBEA, PEX7, COPB2, AP2B1, SRPR, COPB1, SEC24C, SEC24D, KPNB1, AP3B1, STX6, SEC23A, MYO6, IPO8, M6PR, MFN2, IPO4, IPO5, PEX26, KPNA6, KPNA4, RAB10, KPNA3, COPG, H47, ARL6IP1, SNX9, YWHAZ, STX7, DERL1, STAM2, RAB6, NAPA, MACF1, AP3M1, FOLR1, STX18, TMED10, AP3D1, SCG5, PPP3CA, SEC61A1, AP2M1, RAB2A, YWHAB, YWHAE, YWHAG, AP2A2, YWHAH, VCP, TSC2, SYTL4, NOP58, GOSR1, XPO7, ARAP1, SSR3
GO:0016071<br>mRNA metabolic process	62	3,48	3,02E-08	PRPF4B, CRNKL1, SNRPD1, RBM5, SYNCRIP, SMNDC1, PNN, NONO, PARN, DNAJB11, PRMT5, TARDBP, BAT1A, PTBP2, SRPK2, EFTUD2, HNRNPA2B1, SF1, PRPF39, MBNL1, CDC5L, CSTF2T, HNRPDL, AQR, ROD1, VEGFA, CELF4, ESRP1, CELF2, CELF1, RBM39, PRPF38B, CPSF1, THOC1, TDRD3, STRAP, NHP2L1, TRA2B, TRA2A, SF3B2, HNRNPA3, SF3B1, HNRNPK, CNOT6L, PRPF8, HNRNPD, PABPC1, RBM25, PRPF40A, RBM22, BCAS2, CSTF2, ELAVL1, RNPS1, HNRNPA1, SLBP, DCP1A, PNRC2, SFPQ, ZRANB2, SNRNP27, PUF60
GO:0016568<br>chromatin modification	52	2,92	4,34E-08	KDM6A, CTCF, CBX6, KDM1A, EPC1, MLL5, PRMT5, TLK2, KDM5B, MYST4, MLL1, BRD8, KDM5D, SATB1, RCOR1, MECP2, HMG20A, SUZ12, UBE2N, KDM2B, SMARCE1, BAZ1B, HUWE1, BPTF, SMARCAL1, RNF20, SUV420H1, SMARCA4, ING2, NR3C1, TRRAP, CHD7, CHD1L, PBRM1, BAZ2A, DNMT3A, AEBP2, YEATS4, KAT2B, EHMT2, ATXN7L3, PHF17, HDAC3, HDAC2, RNF2, DNMT1, KDM4C, PHF21A, SETD8, RBM14, NCOR1, RERE
GO:0008380<br>RNA splicing	46	2,58	9,07E-08	TDRD3, PRPF4B, CRNKL1, NHP2L1, STRAP, TRA2B, TRA2A, RBM5, SNRPD1, SYNCRIP, SMNDC1, PNN, NONO, HNRNPA3, SF3B1, HNRNPK, PRMT5, PRPF8, TARDBP, BAT1A, PABPC1, PTBP2, PRPF40A, RBM22, BCAS2, SRPK2, EFTUD2, HNRNPA2B1, SF1, PRPF39, RNPS1, MBNL1, CDC5L, HNRNPA1, AQR, SFPQ, CELF4, ZRANB2, ESRP1, CELF2, CELF1, RBM39, SNRNP27, PRPF38B, PUF60, THOC1
GO:0006397<br>mRNA processing	54	3,03	2,32E-07	PRPF4B, CRNKL1, SNRPD1, RBM5, SYNCRIP, SMNDC1, PNN, NONO, PRMT5, TARDBP, BAT1A, PTBP2, SRPK2, EFTUD2, HNRNPA2B1, SF1, PRPF39, CDC5L, MBNL1, CSTF2T, AQR, ROD1, CELF4, ESRP1, CELF2, CELF1, RBM39, PRPF38B, CPSF1, THOC1, TDRD3, STRAP, NHP2L1, TRA2B, TRA2A, SF3B2, HNRNPA3, SF3B1, HNRNPK, CNOT6L, PRPF8, PABPC1, RBM25, PRPF40A, RBM22, BCAS2, CSTF2, RNPS1, HNRNPA1, SLBP, SFPQ, ZRANB2, SNRNP27, PUF60
GO:0048193<br>Golgi vesicle transport	19	1,07	1,05E-06	SEC23A, STX6, NRBP1, PRKCI, USE1, BET1, NBEA, LMAN1, ERGIC1, CHIC2, COPB1, TMED10, SEC22B, GOSR1, GOLGA5, SEC24C, SEC24D, YKT6, SPAST

Biological process	*Gene Count*	*Percentage*	*p-Value*	*Genes induced in RasGrf1 KO pancreatic islets (from Additional file 1: Table S1)*
GO:0022618 ribonucleoprotein complex assembly	15	0,84	1,52E-06	SRPK2, TDRD3, CRNKL1, TSR1, DICER1, RBM5, MBNL1, SMNDC1, EIF3D, EIF3A, PRMT5, CELF4, CELF2, CELF1, PTBP2
GO:0016070 RNA metabolic process	103	5,78	2,04E-06	CHERP, PRPF4B, SNRPD1, RBM5, SYNCRIP, INTS3, PNN, SMNDC1, NONO, INTS8, GTF2E2, INTS5, DNAJB11, INTS7, TARDBP, INTS6, BAT1A, PTBP2, RRP1, DARS, EMG1, EFTUD2, HNRNPA2B1, AARS, RRP8, CSTF2T, DDIT3, AQR, CELF4, VEGFA, CELF2, CELF1, PIAS2, TDRD3, NHP2L1, STRAP, HNRNPA3, HNRNPK, CNOT6L, HNRNPD, PABPC1, PRPF40A, CSTF2, PAIP1, LCMT2, EPRS, RNPS1, HNRNPA1, DCP1A, PNRC2, POP5, SNRNP27, PUF60, NARS, CRNKL1, DICER1, RG9MTD1, PARN, GTF2A1, PRMT5, TRMT5, CDK5RAP1, SRPK2, CCNH, SARS, SF1, PRPF39, MBNL1, CDC5L, HNRPDL, EIF4G2, TARS, TAF10, EIF4G3, ROD1, NCOA6, ESRP1, RBM39, CPSF1, PRPF38B, THOC1, MED1, TRA2B, TRA2A, RPP14, SF3B2, IARS, TFAM, SF3B1, PRPF8, NFAT5, NSUN2, RBM25, NSA2, RBM22, BCAS2, ELAVL1, SLBP, DIS3, SFPQ, THRAP3, ZRANB2, TCEB3
GO:0000245 spliceosome assembly	10	0,56	2,39E-06	SRPK2, TDRD3, CRNKL1, CELF4, RBM5, CELF2, CELF1, PTBP2, MBNL1, SMNDC1
GO:0010468 regulation of gene expression	301	16,90	2,97E-06	ITGB3BP, MEF2A, STAT5B, NAA15, 2810021G02RIK, MED23, MXI1, NONO, EPC1, SIN3A, CREB3L2, PHTF1, SUPT5H, TADA2A, RREB1, ZFP326, RCOR1, ZHX1, MECP2, HMG20A, MED19, MAPK1, HNF4A, BAZ1B, MED17, ZFP280D, TBRG1, SMARCAL1, VEGFA, MNT, ZZZ3, PIAS2, PIAS1, EEFSEC, IRAK1BP1, ZFP825, SUV420H1, AHCTF1, MTIF2, PLAGL1, DRAP1, PBRM1, TCF4, TCF25, NKX2-2, HNRNPAB, KLF5, YEATS4, AEBP2, DNMT3A, KLF6, TGFBR1, PAIP1, ASXL1, SMAD4, SMAD1, UBP1, NCKAP1, FXR1, GCFC1, ETS1, RNF2, DCP1A, ZFP740, TCF12, NCOR1, NCOR2, RERE, BACH1, COPS2, ELF1, ELF2, FOXA2, FOXA3, PHF20, NFKB1, ZEB1, RLIM, ZFP871, PTEN, CREG1, BRD8, ZFP422, ZBTB20, CTBP2, ACO1, CCNH, SNAPC3, SF1, 4930432O21RIK, FOSB, MBD1, SENP2, HNRPDL, TAF10, BRWD1, CHMP1A, GTF2I, ZFP868, ESRP1, CAND1, RBM39, VOPP1, CLOCK, THOC1, MED1, PTOV1, ZMYND11, MTDH, ZFP110, SUFU, SUMO1, XBP1, NR1D2, MORC3, RB1CC1, GATAD2A, CSDE1, SEPSECS, ETV1, HBP1, BAZ2A, SSRP1, JARID2, TRIM28, TRIM27, ELAVL1, ATRX, PHF17, YWHAH, ILF2, YAF2, HNRNPUL1, SFPQ, BNIP3L, PHF21A, NEUROD1, TCEB3, RBM14, APBB2, PBX2, TOB2, MMS19, IMPACT, RBM5, EIF5A, CNOT2, CNOT1, FOXO3, PDX1, CBX1, CNOT7, CNOT6, CBX6, CBFB, CBX5, CNOT4, PNN, ESF1, KDM1A, GTF2E2, MLL5, EIF4EBP2, GATA6, TIA1, TARDBP, PSIP1, PUM1, PUM2, MLL1, DNAJC1, MYST4, SATB1, PKIG, GABPA, FOXN2, CSDA, JUNB, DDIT3, SLTM, SUZ12, HHEX, RALGAPA1, KDM2B, MGA, DNTTIP2, NFE2L2, SMARCA2, SMARCA4, EID1, TSHZ1, RAG1AP1, STRAP, HDGF, KEAP1, TRRAP, ELK3, ATF2, VDR, MEIS2, JUND, HNRNPD, RNF14, EWSR1, CREBZF, MAFB, IREB2, EHMT2, FOXP1, SREBF2, ATF6, ATF5, HDAC3, PSMC5, HDAC2, PNRC2, EBF1, DNMT1, HIVEP1, TMPO, IKBKB, PUF60, PDCD7, E2F5, E2F6, ARID4B, DICER1, FOXK2, PAX6, ZKSCAN1, CTCF, ZKSCAN3, CBFA2T2, MCM7, GTF2A1, PRMT5, PCGF6, RHOA, KDM5B, NFX1, EGR1, KHDRBS1, ELP4, IRAK1, ELP3, CCNL1, CDK8, STXBP1, RUNX1T1, MBNL1, CDC5L, TOPORS, PURB, PURA, MCM6, UBE2N, ZFP654, EIF4G2, NCOA1, EIF4G3, BTG2, HIPK1, BPTF, HIPK3, HIPK2, NCOA6, WASL, PPP1R15B, PPP1R15A, ZFP161, DPF2, BCLAF1, ING2, NFYC, CTNND1, GON4L, NR3C1, TSC22D1, TFAM, CHD7, TNRC6C, NFAT5, MLXIP, TNRC6B, TNRC6A, ING1, KAT2B, ZFP386, CREB1, ATXN7L3, GMCL1, DACH2, SP3, SP4, THRAP3, ZRANB1, KDM4C, ZFP532, SETD8, TBL1X, ARAP1

Biological process	*Gene Count*	*Percentage*	*p-Value*	*Genes induced in RasGrf1 KO pancreatic islets (from Additional file 1: Table S1)*
GO:0006396<br>RNA processing	74	4,15	3,53E-06	CHERP, PRPF4B, CRNKL1, DICER1, SNRPD1, RBM5, RG9MTD1, SYNCRIP, INTS3, SMNDC1, PNN, NONO, INTS8, INTS5, TARDBP, INTS7, PRMT5, TRMT5, INTS6, BAT1A, PTBP2, CDK5RAP1, SRPK2, RRP1, EMG1, EFTUD2, HNRNPA2B1, AARS, SF1, PRPF39, RRP8, MBNL1, CDC5L, CSTF2T, AQR, ROD1, CELF4, ESRP1, CELF2, CELF1, RBM39, PRPF38B, CPSF1, THOC1, TDRD3, STRAP, NHP2L1, TRA2B, TRA2A, RPP14, SF3B2, HNRNPA3, SF3B1, HNRNPK, CNOT6L, PRPF8, PABPC1, NSUN2, RBM25, NSA2, PRPF40A, RBM22, BCAS2, CSTF2, LCMT2, RNPS1, HNRNPA1, SLBP, DIS3, SFPQ, ZRANB2, POP5, SNRNP27, PUF60
GO:0010556<br>regulation of macromolecule biosynthetic process	292	16,40	1,08E-05	ITGB3BP, MEF2A, STAT5B, NAA15, 2810021G02RIK, MED23, MXI1, NONO, EPC1, SIN3A, CREB3L2, PHTF1, SUPT5H, TADA2A, RREB1, ZFP326, RCOR1, ZHX1, MECP2, HMG20A, MED19, MAPK1, HNF4A, BAZ1B, MED17, ZFP280D, BACE2, SMARCAL1, VEGFA, MNT, ZZZ3, PIAS2, PIAS1, EEFSEC, IRAK1BP1, ZFP825, SUV420H1, AHCTF1, MTIF2, PLAGL1, DRAP1, PBRM1, TCF4, TCF25, NKX2-2, HNRNPAB, KLF5, YEATS4, AEBP2, DNMT3A, KLF6, PAIP1, ASXL1, SMAD4, SMAD1, UBP1, FXR1, GCFC1, ETS1, RNF2, DCP1A, ZFP740, TCF12, NCOR1, NCOR2, RERE, BACH1, COPS2, ELF1, ELF2, FOXA2, FOXA3, PHF20, NFKB1, ZEB1, RLIM, ZFP871, CREG1, BRD8, ZFP422, ZBTB20, CTBP2, ACO1, CCNH, SNAPC3, SF1, 4930432O21RIK, FOSB, MBD1, SENP2, HNRPDL, TAF10, BRWD1, CHMP1A, MAST2, GTF2I, ZFP868, CAND1, RBM39, VOPP1, CLOCK, THOC1, MED1, PTOV1, ZMYND11, MTDH, WRNIP1, ZFP110, SUFU, SUMO1, XBP1, NR1D2, RB1CC1, CSDE1, SEPSECS, GATAD2A, ETV1, HBP1, BAZ2A, SSRP1, JARID2, TRIM28, TRIM27, ATRX, PHF17, YWHAH, ILF2, YAF2, HNRNPUL1, SFPQ, PHF21A, TCEB3, NEUROD1, RBM14, APBB2, PBX2, MMS19, TBK1, IMPACT, EIF5A, CNOT2, CNOT1, FOXO3, PDX1, CBX1, CNOT7, CNOT6, CBX6, CBFB, CBX5, CNOT4, PNN, ESF1, KDM1A, GTF2E2, MLL5, EIF4EBP2, GATA6, TIA1, TARDBP, PSIP1, PUM1, PUM2, MLL1, MYST4, DNAJC1, SATB1, FECH, PKIG, GABPA, FOXN2, CSDA, JUNB, DDIT3, SLTM, SUZ12, HHEX, RALGAPA1, KDM2B, MGA, DNTTIP2, NFE2L2, SMARCA2, SMARCA4, EID1, TSHZ1, STRAP, HDGF, KEAP1, TRRAP, ELK3, ATF2, VDR, MEIS2, JUND, HNRNPD, RNF14, EWSR1, CREBZF, MAFB, IREB2, EHMT2, FOXP1, SREBF2, ATF6, ATF5, HDAC3, PSMC5, HDAC2, PNRC2, EBF1, DNMT1, HIVEP1, TMPO, IKBKB, PUF60, PDCD7, E2F5, E2F6, ARID4B, FOXK2, PAX6, ZKSCAN1, CTCF, ZKSCAN3, CBFA2T2, MCM7, GTF2A1, PRMT5, PCGF6, RHOA, KDM5B, NFX1, EGR1, KHDRBS1, ELP4, IRAK1, ELP3, CCNL1, CDK8, RUNX1T1, CDC5L, TOPORS, PURB, PURA, MCM6, UBE2N, ZFP654, EIF4G2, NCOA1, EIF4G3, BTG2, HIPK1, BPTF, HIPK3, HIPK2, NCOA6, WASL, PPP1R15B, PPP1R15A, ZFP161, DPF2, BCLAF1, ING2, NFYC, CTNND1, GON4L, NR3C1, TSC22D1, TFAM, CHD7, TNRC6C, NFAT5, MLXIP, TNRC6B, TNRC6A, ING1, KAT2B, ZFP386, CREB1, ATXN7L3, GMCL1, DACH2, SP3, SP4, THRAP3, ZRANB1, KDM4C, ZFP532, SETD8, TBL1X, ARAP1
GO:0006650<br>glycerophospholipid metabolic process	23	1,29	2,94E-05	CHKA, PIK3C2A, PIGX, TAZ, ABHD5, PI4KA, MECP2, PIP5K1B, PIGQ, PTEN, PIGM, SH3GLB1, CD81, PIK3C3, PIK3CA, PIGC, PCYT1A, PITPNC1, PIP4K2A, IP6K1, PIP4K2C, PIK3R1, PIGA

Biological process	*Gene Count*	*Percentage*	*p-Value*	*Genes induced in RasGrf1 KO pancreatic islets (from Additional file 1: Table S1)*
GO:0031326 regulation of cellular biosynthetic process	297	16,68	3,89E-05	ITGB3BP, MEF2A, ADCY6, STAT5B, NAA15, 2810021G02RIK, MED23, MXI1, NONO, EPC1, SIN3A, CREB3L2, PHTF1, SUPT5H, TADA2A, RREB1, ZFP326, RCOR1, ZHX1, MECP2, HMG20A, MED19, MAPK1, HNF4A, BAZ1B, BACE2, MED17, ZFP280D, SMARCAL1, VEGFA, MNT, ZZZ3, PIAS2, PIAS1, EEFSEC, IRAK1BP1, ZFP825, SUV420H1, AHCTF1, MTIF2, PLAGL1, DRAP1, PBRM1, TCF4, TCF25, NKX2-2, HNRNPAB, KLF5, YEATS4, AEBP2, DNMT3A, KLF6, PAIP1, ASXL1, SMAD4, SMAD1, UBP1, FXR1, GCFC1, ETS1, RNF2, DCP1A, ZFP740, TCF12, NCOR1, NCOR2, RERE, BACH1, COPS2, ELF1, ELF2, FOXA2, FOXA3, PHF20, NFKB1, ZEB1, RLIM, ZFP871, CREG1, BRD8, ZFP422, ZBTB20, CTBP2, ACO1, CCNH, SNAPC3, SF1, 4930432O21RIK, FOSB, MBD1, SENP2, HNRPDL, TAF10, BRWD1, CHMP1A, MAST2, GTF2I, ZFP868, CAND1, RBM39, VOPP1, CLOCK, THOC1, MED1, PTOV1, ZMYND11, MTDH, WRNIP1, ZFP110, SUFU, SUMO1, XBP1, NR1D2, RB1CC1, GATAD2A, CSDE1, SEPSECS, ETV1, HBP1, BAZ2A, SSRP1, JARID2, TRIM28, TRIM27, ATP1A1, ATRX, PHF17, YWHAH, ILF2, ADCY9, YAF2, HNRNPUL1, SFPQ, PHF21A, TCEB3, NEUROD1, RBM14, APBB2, PBX2, MMS19, TBK1, IMPACT, EIF5A, CNOT2, CNOT1, FOXO3, PDX1, CBX1, CNOT7, CNOT6, CBX6, CBFB, CBX5, CNOT4, PNN, ESF1, KDM1A, GTF2E2, MLL5, EIF4EBP2, GATA6, TIA1, TARDBP, PSIP1, PUM1, PUM2, MLL1, MYST4, DNAJC1, SATB1, FECH, PKIG, GABPA, FOXN2, CSDA, JUNB, DDIT3, SLTM, SUZ12, HHEX, RALGAPA1, KDM2B, MGA, DNTTIP2, NFE2L2, SMARCA2, SMARCA4, EID1, TSHZ1, STRAP, HDGF, KEAP1, TRRAP, ELK3, ATF2, VDR, MEIS2, JUND, HNRNPD, RNF14, EWSR1, CREBZF, MAFB, IREB2, EHMT2, FOXP1, SREBF2, ATF6, ATF5, HDAC3, PSMC5, HDAC2, PNRC2, EBF1, DNMT1, HIVEP1, TMPO, IKBKB, PUF60, PDCD7, HSP90AB1, E2F5, E2F6, ARID4B, FOXK2, GABBR1, PAX6, ZKSCAN1, CTCF, ZKSCAN3, CBFA2T2, MCM7, GTF2A1, PRMT5, PCGF6, RHOA, KDM5B, NFX1, EGR1, KHDRBS1, ELP4, IRAK1, ELP3, CCNL1, CDK8, RUNX1T1, CDC5L, TOPORS, PURB, PURA, MCM6, UBE2N, ZFP654, EIF4G2, NCOA1, EIF4G3, BTG2, HIPK1, BPTF, HIPK3, HIPK2, NCOA6, WASL, PPP1R15B, PPP1R15A, ZFP161, DPF2, BCLAF1, ING2, NFYC, CTNND1, GON4L, NR3C1, TSC22D1, TFAM, CHD7, TNRC6C, NFAT5, MLXIP, TNRC6B, TNRC6A, ING1, KAT2B, ZFP386, CREB1, ATXN7L3, GMCL1, DACH2, SP3, SP4, THRAP3, ZRANB1, KDM4C, ZFP532, SETD8, TBL1X, ARAP1
GO:0006888 ER to Golgi vesicle-mediated transport	12	0,67	5,10E-05	SEC23A, NRBP1, USE1, BET1, SEC22B, GOSR1, SEC24C, LMAN1, YKT6, SEC24D, ERGIC1, SPAST
GO:0010629 negative regulation of gene expression	66	3,71	6,46E-05	COPS2, DICER1, NFKB1, CTCF, CBX1, ZEB1, PDX1, MXI1, CBFA2T2, CBX5, KDM1A, EPC1, SIN3A, PCGF6, MYST4, NFX1, KHDRBS1, EGR1, SATB1, CTBP2, PKIG, ZHX1, GABPA, MECP2, PURB, PURA, SUZ12, HHEX, CHMP1A, HIPK1, BPTF, HIPK3, HIPK2, SMARCA4, EID1, BCLAF1, MTDH, STRAP, SUFU, VDR, TNRC6C, DRAP1, GATAD2A, TCF4, TNRC6B, TNRC6A, TCF25, BAZ2A, HNRNPAB, DNMT3A, JARID2, TRIM28, TRIM27, EHMT2, FOXP1, PSMC5, YAF2, RNF2, BNIP3L, ZRANB1, DNMT1, PHF21A, HIVEP1, TBL1X, NCOR1, NCOR2
GO:0045892 negative regulation of transcription, DNA-dependent	53	2,98	6,72E-05	NFKB1, ZEB1, CBX1, PDX1, MXI1, CBFA2T2, CBX5, KDM1A, EPC1, SIN3A, PCGF6, NFX1, EGR1, SATB1, CTBP2, GABPA, ZHX1, PKIG, MECP2, PURB, PURA, SUZ12, HHEX, CHMP1A, HIPK1, BPTF, HIPK3, HIPK2, SMARCA4, EID1, MTDH, STRAP, SUFU, DRAP1, GATAD2A, TCF4, BAZ2A, TCF25, HNRNPAB, DNMT3A, JARID2, TRIM28, TRIM27, EHMT2, FOXP1, RNF2, DNMT1, PHF21A, ZRANB1, HIVEP1, TBL1X, NCOR1, NCOR2

Biological process	*Gene Count*	*Percentage*	*p-Value*	*Genes induced in RasGrf1 KO pancreatic islets (from Additional file 1: Table S1)*
GO:0051253 negative regulation of RNA metabolic process	53	2,98	7,99E-05	NFKB1, ZEB1, CBX1, PDX1, MXI1, CBFA2T2, CBX5, KDM1A, EPC1, SIN3A, PCGF6, NFX1, EGR1, SATB1, CTBP2, GABPA, ZHX1, PKIG, MECP2, PURB, PURA, SUZ12, HHEX, CHMP1A, HIPK1, BPTF, HIPK3, HIPK2, SMARCA4, EID1, MTDH, STRAP, SUFU, DRAP1, GATAD2A, TCF4, BAZ2A, TCF25, HNRNPAB, DNMT3A, JARID2, TRIM28, TRIM27, EHMT2, FOXP1, RNF2, DNMT1, PHF21A, ZRANB1, HIVEP1, TBL1X, NCOR1, NCOR2
GO:0006457 protein folding	28	1,57	8,60E-05	HSP90AB1, FKBP9, FKBP1A, CCT3, CALR, CANX, SEC63, TOR3A, DNAJB11, SH3GLB1, SLMAP, TOR1B, PDRG1, DNAJC5, ERO1L, DNAJA4, QSOX1, DNAJC1, HSPA8, C80913, AARS, CCT6A, NKTR, CCT7, DNAJB9, CCT8, HSPD1, FKBP2
GO:0010608 posttranscriptional regulation of gene expression	31	1,74	9,04E-05	IMPACT, DICER1, EIF5A, MTIF2, PTEN, TNRC6C, EIF4EBP2, MORC3, TIA1, PUM1, SEPSECS, HNRNPD, PUM2, TNRC6B, TNRC6A, DNAJC1, ACO1, PAIP1, STXBP1, IREB2, ELAVL1, FXR1, NCKAP1, EIF4G2, EIF4G3, VEGFA, TBRG1, PPP1R15B, EEFSEC, APBB2, PPP1R15A
GO:0043414 biopolymer methylation	19	1,07	1,26E-04	SATB1, DNMT3A, RAB3D, LCMT2, MECP2, RAB6, CTCF, EHMT2, ETF1, MBD1, SUZ12, MLL5, PRMT1, BTG2, PRMT5, DNMT1, BAZ2A, MLL1, SUV420H1
GO:0016481 negative regulation of transcription	60	3,37	1,37E-04	COPS2, NFKB1, CBX1, ZEB1, PDX1, MXI1, CBFA2T2, CBX5, KDM1A, EPC1, SIN3A, PCGF6, MYST4, NFX1, KHDRBS1, EGR1, SATB1, CTBP2, PKIG, GABPA, ZHX1, MECP2, PURB, PURA, SUZ12, HHEX, CHMP1A, HIPK1, BPTF, HIPK3, HIPK2, SMARCA4, EID1, BCLAF1, MTDH, STRAP, SUFU, VDR, DRAP1, GATAD2A, TCF4, BAZ2A, TCF25, HNRNPAB, DNMT3A, JARID2, TRIM28, TRIM27, EHMT2, FOXP1, PSMC5, YAF2, RNF2, DNMT1, PHF21A, ZRANB1, HIVEP1, TBL1X, NCOR1, NCOR2
GO:0010605 negative regulation of macromolecule metabolic process	76	4,27	1,72E-04	COPS2, IMPACT, DICER1, NFKB1, CTCF, PDX1, CBX1, ZEB1, MXI1, CBFA2T2, CBX5, KDM1A, EPC1, EIF4EBP2, SIN3A, ATG5, PCGF6, TIA1, MYST4, NFX1, KHDRBS1, EGR1, IBTK, SATB1, CTBP2, PKIG, ZHX1, GABPA, MECP2, PURB, PURA, SUZ12, HHEX, CHMP1A, HIPK1, BPTF, HNF4A, BACE2, HIPK3, HIPK2, SMARCA4, EID1, BCLAF1, MTDH, STRAP, FKBP1A, SUFU, VDR, TNRC6C, DRAP1, GATAD2A, TCF4, TNRC6B, TNRC6A, TCF25, BAZ2A, HNRNPAB, DNMT3A, JARID2, TRIM28, TRIM27, EHMT2, YWHAE, FOXP1, FXR1, PSMC5, YAF2, RNF2, BNIP3L, ZRANB1, DNMT1, PHF21A, HIVEP1, TBL1X, NCOR1, NCOR2
GO:0051246 regulation of protein metabolic process	57	3,20	1,73E-04	XPO1, IMPACT, UBE2G1, EIF5A, CTCF, UBQLN1, EIF4EBP2, ATG5, TIA1, PSMD1, PUM1, PSMD2, RAPGEF4, PUM2, ITCH, DNAJC1, MLL1, ADAM9, IBTK, IRAK1, FECH, ACO1, MECP2, UBE2N, EIF4G2, ACVR2A, EIF4G3, HNF4A, UBE2K, HIPK3, CD81, PIAS1, PPP1R15B, EEFSEC, PPP1R15A, UBE2S, CLN6, EGLN2, FKBP1A, UBE2V2, MTIF2, UBE2D3, TNRC6C, RB1CC1, SEPSECS, TNRC6B, TNRC6A, PAIP1, IREB2, SMAD4, UBE2L3, YWHAE, FXR1, AKTIP, PRKAR1B, NCOR1, UBE2E2

Biological process	*Gene Count*	*Percentage*	*p-Value*	*Genes induced in RasGrf1 KO pancreatic islets (from Additional file 1: Table S1)*
GO:0006605 protein targeting	28	1,57	1,94E-04	ARL6IP1, XPO1, YWHAZ, NBEA, PEX7, MACF1, SRPR, FOLR1, PPP3CA, KPNB1, AP3B1, MYO6, YWHAB, IPO8, YWHAE, MFN2, YWHAG, IPO4, IPO5, TSC2, PEX26, NOP58, KPNA6, KPNA4, XPO7, KPNA3, ARAP1, SSR3
GO:0045449 regulation of transcription	266	14,94	2,01E-04	ITGB3BP, MEF2A, STAT5B, NAA15, 2810021G02RIK, MED23, MXI1, NONO, EPC1, SIN3A, CREB3L2, PHTF1, SUPT5H, TADA2A, RREB1, ZFP326, RCOR1, ZHX1, MECP2, HMG20A, MED19, MAPK1, HNF4A, BAZ1B, MED17, ZFP280D, SMARCAL1, VEGFA, MNT, ZZZ3, PIAS2, PIAS1, IRAK1BP1, ZFP825, SUV420H1, AHCTF1, PLAGL1, DRAP1, PBRM1, TCF4, TCF25, NKX2-2, HNRNPAB, KLF5, YEATS4, AEBP2, DNMT3A, KLF6, ASXL1, SMAD4, SMAD1, UBP1, GCFC1, ETS1, RNF2, ZFP740, DCP1A, TCF12, NCOR1, NCOR2, RERE, BACH1, COPS2, ELF1, ELF2, FOXA2, FOXA3, PHF20, NFKB1, ZEB1, RLIM, ZFP871, CREG1, BRD8, ZFP422, ZBTB20, CTBP2, CCNH, SNAPC3, SF1, 4930432O21RIK, FOSB, MBD1, SENP2, HNRPDL, TAF10, CHMP1A, BRWD1, GTF2I, ZFP868, CAND1, RBM39, VOPP1, CLOCK, THOC1, MED1, PTOV1, ZMYND11, MTDH, ZFP110, SUFU, SUMO1, XBP1, NR1D2, RB1CC1, CSDE1, GATAD2A, ETV1, HBP1, BAZ2A, SSRP1, JARID2, TRIM28, TRIM27, ATRX, PHF17, YWHAH, ILF2, YAF2, HNRNPUL1, SFPQ, PHF21A, TCEB3, NEUROD1, RBM14, APBB2, PBX2, MMS19, CNOT2, CNOT1, FOXO3, PDX1, CBX1, CNOT7, CNOT6, CBX6, CBFB, CBX5, CNOT4, PNN, ESF1, KDM1A, GTF2E2, MLL5, GATA6, TARDBP, PSIP1, MYST4, MLL1, SATB1, PKIG, GABPA, FOXN2, CSDA, JUNB, DDIT3, SLTM, SUZ12, HHEX, RALGAPA1, KDM2B, MGA, DNTTIP2, NFE2L2, SMARCA2, SMARCA4, EID1, TSHZ1, STRAP, HDGF, KEAP1, ELK3, TRRAP, ATF2, VDR, MEIS2, JUND, HNRNPD, RNF14, EWSR1, CREBZF, MAFB, EHMT2, FOXP1, SREBF2, ATF6, ATF5, HDAC3, PSMC5, HDAC2, PNRC2, EBF1, DNMT1, HIVEP1, TMPO, IKBKB, PUF60, PDCD7, E2F5, E2F6, FOXK2, ARID4B, PAX6, ZKSCAN1, CTCF, ZKSCAN3, CBFA2T2, MCM7, GTF2A1, PRMT5, PCGF6, RHOA, KDM5B, NFX1, EGR1, KHDRBS1, ELP4, IRAK1, ELP3, CCNL1, CDK8, RUNX1T1, CDC5L, TOPORS, PURB, PURA, MCM6, UBE2N, ZFP654, NCOA1, BPTF, HIPK1, BTG2, HIPK3, HIPK2, NCOA6, WASL, ZFP161, DPF2, BCLAF1, ING2, NFYC, CTNND1, GON4L, NR3C1, TSC22D1, TFAM, CHD7, NFAT5, MLXIP, ING1, KAT2B, CREB1, ZFP386, ATXN7L3, GMCL1, DACH2, SP3, SP4, THRAP3, ZRANB1, ZFP532, KDM4C, SETD8, TBL1X, ARAP1
GO:0010558 negative regulation of macromolecule biosynthetic process	65	3,65	2,04E-04	COPS2, NFKB1, CBX1, ZEB1, PDX1, MXI1, CBFA2T2, CBX5, KDM1A, EPC1, EIF4EBP2, SIN3A, PCGF6, TIA1, MYST4, NFX1, KHDRBS1, EGR1, SATB1, CTBP2, PKIG, GABPA, ZHX1, MECP2, PURB, PURA, SUZ12, HHEX, CHMP1A, HIPK1, BPTF, HIPK3, BACE2, HIPK2, SMARCA4, EID1, BCLAF1, MTDH, STRAP, SUFU, VDR, DRAP1, GATAD2A, TCF4, TNRC6A, TCF25, BAZ2A, HNRNPAB, DNMT3A, JARID2, TRIM28, TRIM27, EHMT2, FOXP1, FXR1, PSMC5, YAF2, RNF2, ZRANB1, DNMT1, PHF21A, HIVEP1, TBL1X, NCOR1, NCOR2
GO:0031327 negative regulation of cellular biosynthetic process	66	3,71	2,62E-04	COPS2, NFKB1, CBX1, ZEB1, PDX1, MXI1, CBFA2T2, CBX5, KDM1A, EPC1, EIF4EBP2, SIN3A, PCGF6, TIA1, MYST4, NFX1, KHDRBS1, EGR1, SATB1, CTBP2, PKIG, ZHX1, GABPA, MECP2, PURB, PURA, SUZ12, HHEX, CHMP1A, HIPK1, BPTF, HIPK3, BACE2, HIPK2, SMARCA4, EID1, BCLAF1, MTDH, STRAP, SUFU, VDR, DRAP1, GATAD2A, TCF4, TNRC6A, TCF25, BAZ2A, HNRNPAB, DNMT3A, JARID2, TRIM28, TRIM27, ATP1A1, EHMT2, FOXP1, FXR1, PSMC5, YAF2, RNF2, ZRANB1, DNMT1, PHF21A, HIVEP1, TBL1X, NCOR1, NCOR2
GO:0030384 phosphoinositide metabolic process	17	0,95	2,88E-04	PIGX, PIK3C2A, PIP5K1B, PI4KA, PIGQ, PTEN, PIGM, CD81, PIK3C3, PIK3CA, PIGC, PITPNC1, PIP4K2A, IP6K1, PIP4K2C, PIK3R1, PIGA

Biological process	*Gene Count*	*Percentage*	*p-Value*	*Genes induced in RasGrf1 KO pancreatic islets (from Additional file 1: Table S1)*
GO:0009890 negative regulation of biosynthetic process	66	3,71	3,43E-04	COPS2, NFKB1, CBX1, ZEB1, PDX1, MXI1, CBFA2T2, CBX5, KDM1A, EPC1, EIF4EBP2, SIN3A, PCGF6, TIA1, MYST4, NFX1, KHDRBS1, EGR1, SATB1, CTBP2, PKIG, ZHX1, GABPA, MECP2, PURB, PURA, SUZ12, HHEX, CHMP1A, HIPK1, BPTF, HIPK3, BACE2, HIPK2, SMARCA4, EID1, BCLAF1, MTDH, STRAP, SUFU, VDR, DRAP1, GATAD2A, TCF4, TNRC6A, TCF25, BAZ2A, HNRNPAB, DNMT3A, JARID2, TRIM28, TRIM27, ATP1A1, EHMT2, FOXP1, FXR1, PSMC5, YAF2, RNF2, ZRANB1, DNMT1, PHF21A, HIVEP1, TBL1X, NCOR1, NCOR2
GO:0006508 proteolysis	135	7,58	3,73E-04	MKRN1, LGMN, PPP2R5C, SAE1, CD2AP, ERLEC1, ZNRF2, CNOT4, CUL3, RABGEF1, RNF149, ITCH, FBXO21, ADAM9, ANAPC5, UBR4, NRD1, NPEPPS, BRAP, DCUN1D1, KDM2B, PSMA6, HUWE1, UBR5, BACE2, BACE1, DLD, ASB3, PIAS2, PGCP, PIAS1, RNF20, RAD23B, DERL1, UBE2V2, CTSA, SKP1A, RBX1, PSMB5, ARIH1, ECE2, PSMB7, UBE2D3, ECE1, FBXO6, FBXW2, RNF11, FBXO3, RNF167, FBXO9, RNF14, HECTD1, CFLAR, CDC23, PCNP, UBE2L3, FBXO38, TULP4, CRBN, PSMC5, VCP, UBA1, RNF2, UBA2, UBE2E2, UBE2G1, MAN1B1, RLIM, OS9, LONP2, TPP1, MAP1LC3B, 6530401D17RIK, TPP2, FBXL14, CASP2, DPP7, NSF, NFX1, CTSZ, UFD1L, HERC4, TOPORS, HERC1, UBE2N, IMMP1L, RFWD2, PJA2, WDR48, SENP2, C8B, CTSL, PJA1, UBE2K, UBC, UCHL5, USP20, ADAM17, CAND1, UBB, CTSB, SIAH2, UBE2S, PMPCA, PMPCB, UBE3B, USP9X, UBE3C, 2700078E11RIK, CPN1, SUMO3, PCSK2, SUMO1, DNER, PITRM1, PREPL, SCG5, TRIP12, STAMBP, CAPN7, UBE4A, SPSB1, LRRC41, MARCH5, PSMD14, WSB2, USP47, ACE2, ZRANB1, USP48, CUL4B, PRSS23, TBL1X, MBTPS1, USP45, RNF41
GO:0019219 regulation of nucleobase, nucleoside, nucleotide and nucleic acid metabolic process	277	15,55	3,86E-04	ITGB3BP, MEF2A, ADCY6, STAT5B, NAA15, 2810021G02RIK, MED23, MXI1, NONO, EPC1, SIN3A, CREB3L2, PHTF1, SUPT5H, TADA2A, RREB1, ZFP326, RCOR1, ZHX1, MECP2, HMG20A, MED19, MAPK1, HNF4A, BAZ1B, MED17, ZFP280D, SMARCAL1, VEGFA, MNT, ZZZ3, PIAS2, PIAS1, IRAK1BP1, ZFP825, SUV420H1, AHCTF1, PLAGL1, DRAP1, PBRM1, TCF4, TCF25, NKX2-2, HNRNPAB, KLF5, YEATS4, AEBP2, DNMT3A, KLF6, ASXL1, SMAD4, SMAD1, UBP1, GCFC1, ETS1, RNF2, DCP1A, ZFP740, TCF12, NCOR1, NCOR2, RERE, BACH1, COPS2, ELF1, ELF2, FOXA2, FOXA3, PHF20, NFKB1, ZEB1, RLIM, ZFP871, CREG1, BRD8, ZFP422, ZBTB20, CTBP2, CCNH, SNAPC3, SF1, 4930432O21RIK, FOSB, MBD1, SENP2, HNRPDL, TAF10, CHMP1A, BRWD1, GTF2I, ZFP868, ESRP1, CAND1, RBM39, VOPP1, CLOCK, THOC1, MED1, PTOV1, ZMYND11, MTDH, WRNIP1, ZFP110, SUFU, SUMO1, XBP1, NR1D2, RB1CC1, PKD2, CSDE1, GATAD2A, ETV1, HBP1, BAZ2A, SSRP1, JARID2, TRIM28, TRIM27, ELAVL1, ATRX, PHF17, YWHAH, ILF2, ADCY9, YAF2, HNRNPUL1, SFPQ, PHF21A, TCEB3, NEUROD1, RBM14, APBB2, PBX2, MMS19, RBM5, CNOT2, CNOT1, FOXO3, PDX1, CBX1, CNOT7, CNOT6, CBX6, CBFB, CBX5, CNOT4, PNN, ESF1, KDM1A, GTF2E2, MLL5, GATA6, TARDBP, PSIP1, MYST4, MLL1, SATB1, PKIG, GABPA, FOXN2, CSDA, JUNB, DDIT3, SLTM, SUZ12, HHEX, RALGAPA1, KDM2B, MGA, DNTTIP2, NFE2L2, SMARCA2, SMARCA4, EID1, TSHZ1, STRAP, HDGF, KEAP1, ELK3, TRRAP, ATF2, VDR, MEIS2, JUND, HNRNPD, RNF14, EWSR1, CREBZF, MAFB, EHMT2, FOXP1, SREBF2, ATF6, ATF5, HDAC3, PSMC5, HDAC2, PNRC2, EBF1, DNMT1, HIVEP1, TMPO, IKBKB, PUF60, PDCD7, E2F5, E2F6, ARID4B, FOXK2, GABBR1, PAX6, ZKSCAN1, CTCF, ZKSCAN3, CBFA2T2, MCM7, GTF2A1, PRMT5, PCGF6, RHOA, KDM5B, NFX1, EGR1, KHDRBS1, ELP4, IRAK1, ELP3, CCNL1, CDK8, RUNX1T1, CDC5L, MBNL1, TOPORS, PURB, PURA, MCM6, UBE2N, ZFP654, NCOA1, BPTF, HIPK1, BTG2, HIPK3, HIPK2, NCOA6, WASL, ZFP161, SMARCAD1, DPF2, BCLAF1, ING2, NFYC, CTNND1, GON4L, NR3C1, TSC22D1, TFAM, CHD7, NFAT5, MLXIP, ING1, TERF2, KAT2B, CREB1, ZFP386, ATXN7L3, GMCL1, DACH2, SP3, SP4, THRAP3, ZRANB1, KDM4C, ZFP532, SETD8, TBL1X, ARAP1
GO:0033365 protein localization in organelle	22	1,24	3,92E-04	XPO1, PPP3R1, PAX6, IPO8, MFN2, BBS2, SYNE2, MACF1, FOLR1, SRPR, MORC3, IPO4, IPO5, TSC2, NOP58, KPNA6, KPNA4, PPP3CA, KPNA3, XPO7, KPNB1, ARAP1

Biological process	*Gene Count*	*Percentage*	*p-Value*	*Genes induced in RasGrf1 KO pancreatic islets (from Additional file 1: Table S1)*
GO:0031324 negative regulation of cellular metabolic process	72	4,04	4,75E-04	COPS2, IMPACT, NFKB1, PDX1, CBX1, ZEB1, MXI1, CBFA2T2, CBX5, KDM1A, EPC1, EIF4EBP2, SIN3A, ATG5, PCGF6, TIA1, MYST4, NFX1, KHDRBS1, EGR1, IBTK, SATB1, CTBP2, PKIG, ZHX1, GABPA, MECP2, PURB, PURA, SUZ12, HHEX, CHMP1A, HIPK1, BPTF, HNF4A, HIPK3, BACE2, HIPK2, SMARCA4, EID1, BCLAF1, MTDH, STRAP, FKBP1A, SUFU, VDR, DRAP1, GATAD2A, TCF4, TNRC6A, TCF25, BAZ2A, HNRNPAB, DNMT3A, JARID2, TRIM28, TRIM27, ATP1A1, EHMT2, YWHAE, FOXP1, FXR1, PSMC5, YAF2, RNF2, ZRANB1, DNMT1, PHF21A, HIVEP1, TBL1X, NCOR1, NCOR2
GO:0006644 phospholipid metabolic process	31	1,74	5,27E-04	CHKA, SGPP1, ABHD5, PIP5K1B, SGMS1, PTEN, PIGM, SH3GLB1, GATA6, PLA2G12A, PIK3C3, PIGC, PIK3CA, PITPNC1, PCYT1A, IP6K1, PPAP2B, PIK3R1, PIGA, LPGAT1, PIK3C2A, PIGX, TAZ, MECP2, PI4KA, PIGQ, LYST, CD81, SMPD1, PIP4K2A, PIP4K2C
GO:0043066 negative regulation of apoptosis	41	2,30	5,36E-04	XRCC5, STAT5B, SGMS1, GCLM, PTEN, SIN3A, KRAS, ATG5, RB1CC1, TPT1, RHOA, PIK3CA, DNAJC5, GLO1, CASP2, PIK3R1, RASA1, STAMBP, CFLAR, ROCK1, TGFBR1, TMBIM6, AARS, MECP2, POLB, CSDA, ATM, TAX1BP1, DAPK1, ATF5, BFAR, YWHAH, CDKN1B, BTG2, VEGFA, HIPK2, BNIP3L, NEUROD1, MAPK8, APBB2, F2R
GO:0045934 negative regulation of nucleobase, nucleoside, nucleotide and nucleic acid metabolic process	60	3,37	7,70E-04	COPS2, NFKB1, CBX1, ZEB1, PDX1, MXI1, CBFA2T2, CBX5, KDM1A, EPC1, SIN3A, PCGF6, MYST4, NFX1, KHDRBS1, EGR1, SATB1, CTBP2, PKIG, GABPA, ZHX1, MECP2, PURB, PURA, SUZ12, HHEX, CHMP1A, HIPK1, BPTF, HIPK3, HIPK2, SMARCA4, EID1, BCLAF1, MTDH, STRAP, SUFU, VDR, DRAP1, GATAD2A, TCF4, BAZ2A, TCF25, HNRNPAB, DNMT3A, JARID2, TRIM28, TRIM27, EHMT2, FOXP1, PSMC5, YAF2, RNF2, DNMT1, PHF21A, ZRANB1, HIVEP1, TBL1X, NCOR1, NCOR2
GO:0043069 negative regulation of programmed cell death	41	2,30	8,16E-04	XRCC5, STAT5B, SGMS1, GCLM, PTEN, SIN3A, KRAS, ATG5, RB1CC1, TPT1, RHOA, PIK3CA, DNAJC5, GLO1, CASP2, PIK3R1, RASA1, STAMBP, CFLAR, ROCK1, TGFBR1, TMBIM6, AARS, MECP2, POLB, CSDA, ATM, TAX1BP1, DAPK1, ATF5, BFAR, YWHAH, CDKN1B, BTG2, VEGFA, HIPK2, BNIP3L, NEUROD1, MAPK8, APBB2, F2R
GO:0060548 negative regulation of cell death	41	2,30	8,85E-04	XRCC5, STAT5B, SGMS1, GCLM, PTEN, SIN3A, KRAS, ATG5, RB1CC1, TPT1, RHOA, PIK3CA, DNAJC5, GLO1, CASP2, PIK3R1, RASA1, STAMBP, CFLAR, ROCK1, TGFBR1, TMBIM6, AARS, MECP2, POLB, CSDA, ATM, TAX1BP1, DAPK1, ATF5, BFAR, YWHAH, CDKN1B, BTG2, VEGFA, HIPK2, BNIP3L, NEUROD1, MAPK8, APBB2, F2R

Biological process	*Gene Count*	*Percentage*	*p-Value*	*Genes induced in RasGrf1 KO pancreatic islets (from Additional file 1: Table S1)*
GO:0051172<br>negative regulation of nitrogen compound metabolic process	60	3,37	9,86E-04	COPS2, NFKB1, CBX1, ZEB1, PDX1, MXI1, CBFA2T2, CBX5, KDM1A, EPC1, SIN3A, PCGF6, MYST4, NFX1, KHDRBS1, EGR1, SATB1, CTBP2, PKIG, GABPA, ZHX1, MECP2, PURB, PURA, SUZ12, HHEX, CHMP1A, HIPK1, BPTF, HIPK3, HIPK2, SMARCA4, EID1, BCLAF1, MTDH, STRAP, SUFU, VDR, DRAP1, GATAD2A, TCF4, BAZ2A, TCF25, HNRNPAB, DNMT3A, JARID2, TRIM28, TRIM27, EHMT2, FOXP1, PSMC5, YAF2, RNF2, DNMT1, PHF21A, ZRANB1, HIVEP1, TBL1X, NCOR1, NCOR2
GO:0010970<br>microtubule-based transport	8	0,45	1,04E-03	KIF1B, NDEL1, KIF5B, KLC1, RHOT1, PAFAH1B1, DST, NEFM
GO:0032268<br>regulation of cellular protein metabolic process	45	2,53	1,13E-03	IMPACT, EIF5A, CTCF, FKBP1A, MTIF2, UBQLN1, TNRC6C, EIF4EBP2, ATG5, RB1CC1, TIA1, PUM1, SEPSECS, RAPGEF4, PUM2, TNRC6B, TNRC6A, DNAJC1, MLL1, ADAM9, IBTK, IRAK1, FECH, ACO1, PAIP1, IREB2, SMAD4, MECP2, YWHAE, FXR1, UBE2N, EIF4G2, ACVR2A, EIF4G3, AKTIP, HNF4A, HIPK3, PRKAR1B, CD81, PIAS1, EEFSEC, PPP1R15B, PPP1R15A, NCOR1, CLN6
GO:0006417<br>regulation of translation	21	1,18	1,52E-03	ACO1, PAIP1, IMPACT, IREB2, EIF5A, MTIF2, FXR1, EIF4G2, EIF4G3, EIF4EBP2, TNRC6C, TIA1, PUM1, SEPSECS, PUM2, EEFSEC, PPP1R15B, TNRC6B, PPP1R15A, TNRC6A, DNAJC1
GO:0007369<br>gastrulation	17	0,95	1,89E-03	TWSG1, FOXA2, NF2, ARFRP1, SMAD4, SMAD1, NCKAP1, CUL3, FAM48A, ACVR2A, MACF1, RNF2, DLD, TXNRD1, PPAP2B, BMPR1A, ACVR1
GO:0006984<br>ER-nuclear signaling pathway	9	0,51	1,99E-03	DERL1, ATP2A2, VAPB, AARS, ERO1L, NFE2L2, PPP1R15B, PPP1R15A, DDIT3
GO:0016310<br>phosphorylation	95	5,33	2,09E-03	NRBP1, PRPF4B, STK38, TBK1, ATP5B, ATP6AP1, CAPZA2, CAD, CLK1, TGFB2, MAP3K4, SLK, PAK2, PAK3, CLK2, PIK3C3, CLK4, ILK, PIK3CA, TGFA, ATP5L, BRD4, TLK2, IP6K1, ATP6V0D1, CDK16, CDK14, IRAK1, SRPK2, BCR, ROCK1, PIK3C2A, ROCK2, STK24, TRPM7, PRKCI, PI4KA, CDK8, MECP2, PKDCC, DGUOK, DAPK1, GAK, PRKD1, MAP4K3, ACVR2A, PDIK1L, MAPK1, ATP6V1A, MAST2, BAZ1B, MAPK6, HIPK1, RIOK3, HIPK3, CD81, DLD, HIPK2, NEK9, MAPK8, CDK11B, NEK7, ACVR1, STK16, CHEK1, ABI1, PXK, ATP6V0B, PTK2, SNRK, MAP3K3, MORC3, PIK3R4, PIK3R1, TES, CSNK1A1, FLT1, MAP2K1, RYK, TGFBR1, CREB1, PTPRA, TRIM28, TRIO, ATM, ATP6V0E2, CSNK1E, RPS6KA2, ULK1, PRKAR1B, ULK2, JAK1, IKBKB, BMPR1A, F2R

Biological process	*Gene Count*	*Percentage*	*p-Value*	*Genes induced in RasGrf1 KO pancreatic islets (from Additional file 1: Table S1)*
GO:0008088<br>axon cargo transport	6	0,34	2,52E-03	KIF1B, NDEL1, KLC1, PAFAH1B1, DST, NEFM
GO:0006376<br>mRNA splice site selection	5	0,28	2,54E-03	CELF4, CELF2, CELF1, PTBP2, MBNL1
GO:0006413<br>translational initiation	11	0,62	2,92E-03	EIF3C, EIF3D, EIF2S3X, EIF3A, EIF3B, EIF2S2, EIF5, EIF1B, EIF1, DENR, MTIF2
GO:0051493<br>regulation of cytoskeleton organization	20	1,12	3,22E-03	XPO1, ROCK2, CAPZA2, MTAP4, VIL1, RDX, MID1IP1, DSTN, ACTR3, ARPC1A, CDKN1B, ARPC2, RAC1, RHOA, CLASP1, SPNA2, MAPRE1, DST, RASA1, TES
GO:0046486<br>glycerolipid metabolic process	24	1,35	3,41E-03	LPL, CHKA, PIK3C2A, PIGX, TAZ, ABHD5, PI4KA, MECP2, PIP5K1B, PIGQ, PTEN, PIGM, SH3GLB1, CD81, PIK3C3, PIK3CA, PIGC, PCYT1A, PITPNC1, PIP4K2A, IP6K1, PIP4K2C, PIK3R1, PIGA
GO:0030705<br>cytoskeleton-dependent intracellular transport	10	0,56	3,61E-03	KIF1B, NDEL1, KIF5B, KLC1, WASF2, RHOT1, PAFAH1B1, APBB2, DST, NEFM
GO:0030968<br>endoplasmic reticulum unfolded protein response	7	0,39	4,23E-03	DERL1, VAPB, AARS, ERO1L, NFE2L2, PPP1R15A, DDIT3
GO:0034620<br>cellular response to unfolded protein	7	0,39	4,23E-03	DERL1, VAPB, AARS, ERO1L, NFE2L2, PPP1R15A, DDIT3

Biological process	*Gene Count*	*Percentage*	*p-Value*	*Genes induced in RasGrf1 KO pancreatic islets (from Additional file 1: Table S1)*
GO:0050657<br>nucleic acid transport	15	0,84	4,25E-03	XPO1, NUP160, NUP88, HNRNPA2B1, G3BP2, AHCTF1, EIF5A, HNRNPA1, NUPL1, SENP2, DDX19A, SEH1L, BAT1A, XPO7, THOC1
GO:0050658<br>RNA transport	15	0,84	4,25E-03	XPO1, NUP160, NUP88, HNRNPA2B1, G3BP2, AHCTF1, EIF5A, HNRNPA1, NUPL1, SENP2, DDX19A, SEH1L, BAT1A, XPO7, THOC1
GO:0051028<br>mRNA transport	14	0,79	6,38E-03	XPO1, NUP160, NUP88, G3BP2, AHCTF1, EIF5A, HNRNPA1, NUPL1, SENP2, DDX19A, SEH1L, BAT1A, XPO7, THOC1
GO:0070507<br>regulation of microtubule cytoskeleton organization	10	0,56	6,74E-03	XPO1, CDKN1B, ROCK2, CAPZA2, MTAP4, CLASP1, MAPRE1, MID1IP1, DST, TES
GO:0007264<br>small GTPase mediated signal transduction	39	2,19	7,19E-03	CTNNAL1, GNA13, RAB3D, WASF2, ARFRP1, RAB6, IQGAP1, ARL5A, KRAS, NISCH, RAB28, RAC1, RHOA, RAB11A, RHOB, RAPGEF4, RAB2A, GDI1, RAB2B, GDI2, ROCK1, RABIF, ROCK2, RAB4A, G3BP2, TRIM23, BRAP, ARF1, ULK1, RASGRF1, RAB34, ARF4, RAB22A, RAB14, RHOT1, RIT1, ARL8B, RAB10, RASD1
GO:0006606<br>protein import into nucleus	13	0,73	7,20E-03	XPO1, IPO8, IPO4, TSC2, IPO5, KPNA6, NOP58, PPP3CA, KPNA4, XPO7, KPNA3, KPNB1, ARAP1
GO:0001701<br>in utero embryonic development	40	2,25	7,49E-03	GNA13, ZFAND5, 2610005L07RIK, EGLN1, KEAP1, ITGB1, CUL3, RPA1, KDM1A, PRMT1, SIN3A, CHD7, GATA6, PKD2, GATAD2A, PKD1, ZFP830, TERF2, MAP2K1, TGFBR1, GABPA, PTPRR, MBNL1, CSDA, JUNB, BCL2L11, NCKAP1, MFN2, MAPK1, TJP1, NDEL1, SP3, NCOA6, CDK11B, NCOR2, BMPR1A, ADD1, SMARCA4, ACVR1, MED1
GO:0046834<br>lipid phosphorylation	6	0,34	8,51E-03	PIK3C2A, PIK3C3, PI4KA, PIK3CA, IP6K1, PIK3R1
GO:0016311<br>dephosphorylation	24	1,35	1,03E-02	PTPRJ, ILKAP, PTPRF, PTPRA, PPM1A, PTPRR, PPM1B, PTEN, PTPN12, MTMR2, MTMR3, PPM1G, DUSP19, DUSP1, PTPLB, PTP4A2, DUSP16, MTMR9, PPP3CA, MTMR6, MTMR7, MTMR4, INPP5A, DUSP6

Biological process	***Gene Count***	***Percentage***	***p-Value***	***Genes induced in RasGrf1 KO pancreatic islets (from Additional file 1: Table S1)***
GO:0010638 positive regulation of organelle organization	12	0,67	1,09E-02	UBE2N, CDKN1B, ROCK2, ARPC2, CAPZA2, RAC1, RHOA, TGFA, NCOR1, TERF2, DSTN, TES
GO:0000059 protein import into nucleus, docking	6	0,34	1,17E-02	XPO1, IPO4, IPO5, IPO8, XPO7, KPNB1
GO:0017038 protein import	16	0,90	1,29E-02	XPO1, IPO8, PEX7, MFN2, IPO4, IPO5, TSC2, PEX26, KPNA6, NOP58, PPP3CA, KPNA4, XPO7, KPNA3, KPNB1, ARAP1
GO:0033044 regulation of chromosome organization	7	0,39	1,32E-02	UBE2N, MECP2, CTCF, TLK2, NCOR1, TERF2, MLL1
GO:0010628 positive regulation of gene expression	64	3,59	1,41E-02	MEF2A, ELF1, FOXA2, STAT5B, PAX6, NFKB1, CTCF, PDX1, FOXO3, ZEB1, CNOT7, CBFB, KDM1A, EPC1, MLL5, GATA6, MLL1, MYST4, EGR1, ZFP326, MECP2, TOPORS, DDIT3, NCOA1, HNF4A, MED17, VEGFA, HIPK2, CAND1, NFE2L2, CLOCK, MED1, ING2, RAG1AP1, AHCTF1, NFYC, PLAGL1, MEIS2, NFAT5, ETV1, ING1, NKX2-2, HNRNPAB, KLF5, KLF6, KAT2B, MAFB, CREB1, TRIM28, SMAD4, SMAD1, ATXN7L3, SREBF2, YWHAH, ILF2, ETS1, YAF2, DCP1A, EBF1, THRAP3, NEUROD1, RBM14, PBX2, ARAP1
GO:0009144 purine nucleoside triphosphate metabolic process	19	1,07	1,46E-02	ATP1B1, ATP1B3, ATP5B, AK1, ATP6AP1, ATP11A, ATP1A1, DGUOK, ATP6V0B, RP2H, ATP6V1A, ATP2B2, ATP6V0E2, ATP2A2, ATP2A3, ATP9A, ATP5L, ATP6V0D1, ATP8A1
GO:0031110 regulation of microtubule polymerization or depolymerization	8	0,45	1,62E-02	CDKN1B, CAPZA2, MTAP4, CLASP1, MAPRE1, MID1IP1, DST, TES

Biological process	*Gene Count*	*Percentage*	*p-Value*	*Genes induced in RasGrf1 KO pancreatic islets (from Additional file 1: Table S1)*
GO:0045941 positive regulation of transcription	62	3,48	1,72E-02	MEF2A, ELF1, FOXA2, STAT5B, PAX6, NFKB1, PDX1, FOXO3, ZEB1, CNOT7, CBFB, KDM1A, EPC1, MLL5, GATA6, MLL1, MYST4, EGR1, ZFP326, MECP2, TOPORS, DDIT3, NCOA1, HNF4A, MED17, VEGFA, HIPK2, CAND1, NFE2L2, CLOCK, MED1, ING2, AHCTF1, NFYC, PLAGL1, MEIS2, NFAT5, ETV1, ING1, NKX2-2, HNRNPAB, KLF5, KLF6, KAT2B, MAFB, CREB1, TRIM28, SMAD4, SMAD1, ATXN7L3, SREBF2, YWHAH, ILF2, ETS1, YAF2, DCP1A, EBF1, THRAP3, NEUROD1, RBM14, PBX2, ARAP1
GO:0006041 glucosamine metabolic process	5	0,28	1,74E-02	GNPDA1, GNPDA2, GNE, GNPNAT1, NAGK
GO:0040029 regulation of gene expression, epigenetic	14	0,79	1,76E-02	DNMT3A, RAG1AP1, DICER1, MECP2, TRIM27, CTCF, MBD1, EPC1, MLL5, DNMT1, BAZ2A, TNRC6A, MLL1, SMARCA4
GO:0043087 regulation of GTPase activity	17	0,95	1,83E-02	ARFGAP1, GDI1, GDI2, RABGAP1, TBC1D8, AGFG1, TBC1D19, TBC1D15, SMAP2, TBC1D23, ARHGAP6, NDEL1, RASGRF1, TSC2, PAFAH1B1, TBC1D20, ARAP1
GO:0007507 heart development	33	1,85	1,83E-02	RBP4, NRP1, DICER1, PPP3R1, FKBP1A, EGLN1, ITGB1, PTEN, SUFU, TGFB2, ECE2, CHD7, ATG5, GATA6, RB1CC1, PKD2, PKD1, PBRM1, PTPRJ, TGFBR1, ATM, FOXP1, NCKAP1, HHEX, TSC2, NCOA6, GAA, PTCH1, NCOR2, BMPR1A, ACVR1, SMARCA4, MED1
GO:0010604 positive regulation of macromolecule metabolic process	79	4,44	1,99E-02	ELF1, MEF2A, FOXA2, TBK1, STAT5B, PAX6, EIF5A, NFKB1, CTCF, PDX1, FOXO3, ZEB1, CNOT7, CBFB, EPC1, KDM1A, MLL5, GATA6, ITCH, SUPT5H, MLL1, MYST4, ADAM9, EGR1, ZFP326, MECP2, TOPORS, DDIT3, UBE2N, ACVR2A, NCOA1, HNF4A, MED17, CD81, HIPK2, VEGFA, CAND1, NFE2L2, PIAS1, CLOCK, MED1, CLN6, ING2, RAG1AP1, AHCTF1, NFYC, EGLN2, PLAGL1, MEIS2, RB1CC1, NFAT5, ETV1, ING1, NKX2-2, TERF2, HNRNPAB, KLF5, KLF6, KAT2B, MAFB, CREB1, TRIM28, SMAD4, SMAD1, ATXN7L3, SREBF2, YWHAH, AKTIP, ILF2, YAF2, ETS1, DCP1A, EBF1, THRAP3, NEUROD1, RBM14, PBX2, NCOR1, ARAP1
GO:0001944 vasculature development	36	2,02	1,99E-02	GNA13, ZFAND5, NRP1, TIPARP, WASF2, DICER1, NAA15, ELK3, CDH2, PTEN, TGFB2, PTK2, CHD7, ATG5, AGGF1, CTGF, GATAD2A, PKD1, TGFA, RHOB, PPAP2B, RASA1, PTPRJ, KLF5, SGPL1, FLT1, MAP2K1, TGFBR1, ARHGAP24, UBP1, JUNB, MAPK1, NCOA6, VEGFA, ACVR1, SMARCA4
GO:0032271 regulation of protein polymerization	12	0,67	2,12E-02	ACTR3, ARPC1A, CDKN1B, ARPC2, CAPZA2, RAC1, VIL1, RDX, SPNA2, MAPRE1, RASA1, TES

Biological process	*Gene Count*	*Percentage*	*p-Value*	*Genes induced in RasGrf1 KO pancreatic islets (from Additional file 1: Table S1)*
GO:0009142 nucleoside triphosphate biosynthetic process	17	0,95	2,21E-02	ATP1B1, ATP1B3, ATP6AP1, ATP5B, ATP11A, ATP1A1, ATP6V0B, RP2H, ATP6V1A, ATP2B2, ATP6V0E2, ATP2A2, ATP2A3, ATP9A, ATP5L, ATP6V0D1, ATP8A1
GO:0001568 blood vessel development	35	1,97	2,29E-02	GNA13, NRP1, TIPARP, WASF2, DICER1, NAA15, ELK3, CDH2, PTEN, TGFB2, PTK2, CHD7, ATG5, AGGF1, CTGF, GATAD2A, PKD1, TGFA, RHOB, PPAP2B, RASA1, PTPRJ, KLF5, SGPL1, FLT1, MAP2K1, TGFBR1, ARHGAP24, UBP1, JUNB, MAPK1, NCOA6, VEGFA, ACVR1, SMARCA4
GO:0045935 positive regulation of nucleobase, nucleoside, nucleotide and nucleic acid metabolic process	65	3,65	2,38E-02	MEF2A, ELF1, FOXA2, STAT5B, PAX6, NFKB1, PDX1, FOXO3, ZEB1, CNOT7, CBFB, KDM1A, EPC1, MLL5, GATA6, SUPT5H, MLL1, MYST4, EGR1, ZFP326, MECP2, TOPORS, DDIT3, UBE2N, NCOA1, HNF4A, MED17, VEGFA, HIPK2, CAND1, NFE2L2, CLOCK, MED1, ING2, AHCTF1, NFYC, PLAGL1, MEIS2, NFAT5, ETV1, ING1, TERF2, NKX2-2, HNRNPAB, KLF5, KLF6, KAT2B, MAFB, CREB1, TRIM28, SMAD4, SMAD1, ATXN7L3, SREBF2, YWHAH, ILF2, ETS1, YAF2, DCP1A, EBF1, THRAP3, NEUROD1, RBM14, PBX2, ARAP1
GO:0043009 chordate embryonic development	55	3,09	2,44E-02	GNA13, ENAH, FOXA2, PAX6, ZEB1, CUL3, KDM1A, PRMT1, SIN3A, GATA6, ZFP830, ETL4, GABPA, PTPRR, MBNL1, CSDA, JUNB, BCL2L11, MFN2, ACVR2A, MAPK1, NDEL1, NCOA6, CDK11B, ADD1, MED1, SMARCA4, ACVR1, ZFAND5, RBP4, 2610005L07RIK, LIMS1, ABI1, KEAP1, EGLN1, ITGB1, SUFU, RPA1, CHD7, ECE1, PKD2, GATAD2A, PKD1, TERF2, HECTD1, MAP2K1, TGFBR1, ATM, NCKAP1, TJP1, SP3, TSC2, PTCH1, NCOR2, BMPR1A
GO:0006304 DNA modification	7	0,39	2,54E-02	DNMT3A, MLL5, DNMT1, CTCF, MBD1, BAZ2A, MLL1
GO:0051169 nuclear transport	17	0,95	2,88E-02	XPO1, NUP160, IPO8, HNRNPA1, IPO4, IPO5, TSC2, TBRG1, KPNA6, NOP58, MLXIP, PPP3CA, KPNA4, KPNA3, XPO7, KPNB1, ARAP1
GO:0016477 cell migration	34	1,91	2,94E-02	ZFAND5, 2610005L07RIK, NRP1, CAPZA2, ATP5B, WASF2, PAX6, CDH2, CD2AP, ITGB1, PTEN, PEX7, TGFB2, PTK2, CTGF, PXMP3, RAC1, PAFAH1B1, CAP1, DCX, TES, SGPL1, FLT1, TGFBR1, YWHAE, NCKAP1, NDEL1, SYNE2, ULK1, NCK1, LYST, VEGFA, APBB2, ACVR1

Biological process	*Gene Count*	*Percentage*	*p-Value*	*Genes induced in RasGrf1 KO pancreatic islets (from Additional file 1: Table S1)*
GO:0051173<br>positive regulation of nitrogen compound metabolic process	66	3,71	2,95E-02	HSP90AB1, ELF1, MEF2A, FOXA2, STAT5B, PAX6, NFKB1, PDX1, FOXO3, ZEB1, CNOT7, CBFB, KDM1A, EPC1, MLL5, GATA6, SUPT5H, MLL1, MYST4, EGR1, ZFP326, MECP2, TOPORS, DDIT3, UBE2N, NCOA1, HNF4A, MED17, VEGFA, HIPK2, CAND1, NFE2L2, CLOCK, MED1, ING2, AHCTF1, NFYC, PLAGL1, MEIS2, NFAT5, ETV1, ING1, TERF2, NKX2-2, HNRNPAB, KLF5, KLF6, KAT2B, MAFB, CREB1, TRIM28, SMAD4, SMAD1, ATXN7L3, SREBF2, YWHAH, ILF2, ETS1, YAF2, DCP1A, EBF1, THRAP3, NEUROD1, RBM14, PBX2, ARAP1
GO:0044275<br>cellular carbohydrate catabolic process	12	0,67	3,01E-02	ALDOA, GNPDA1, PFKL, PKM2, GAA, ENO2, PDHA1, PFKM, DLAT, OGDH, GAPDH, HIBADH
GO:0032392<br>DNA geometric change	5	0,28	3,21E-02	TOP1, MCM7, SUPV3L1, PURB, MCM6
GO:0046474<br>glycerophospholipid biosynthetic process	10	0,56	3,26E-02	CHKA, PIGM, PIGX, SH3GLB1, ABHD5, CD81, PIGC, PCYT1A, PIGQ, PIGA
GO:0046365<br>monosaccharide catabolic process	11	0,62	3,54E-02	ALDOA, GNPDA1, PFKL, PKM2, ENO2, PDHA1, PFKM, DLAT, OGDH, GAPDH, HIBADH
GO:0010761<br>fibroblast migration	4	0,22	3,63E-02	2610005L07RIK, SGPL1, ZFAND5, SYNE2
GO:0051495<br>positive regulation of cytoskeleton organization	7	0,39	3,67E-02	CDKN1B, ARPC2, CAPZA2, RAC1, RHOA, DSTN, TES

Biological process	*Gene Count*	*Percentage*	*p-Value*	*Genes induced in RasGrf1 KO pancreatic islets (from Additional file 1: Table S1)*
GO:0030258<br>lipid modification	10	0,56	3,69E-02	PIK3C2A, PIK3C3, PI4KA, ADIPOR1, PIK3CA, IP6K1, PTEN, PIK3R1, HADHA, PEX7
GO:0045454<br>cell redox homeostasis	12	0,67	3,74E-02	TXNL1, P4HB, SEPW1, TMX1, DLD, EGLN2, TXNRD1, PDIA5, QSOX1, DDIT3, GLRX2, GLRX
GO:0031325<br>positive regulation of cellular metabolic process	78	4,38	3,78E-02	HSP90AB1, ELF1, MEF2A, FOXA2, TBK1, STAT5B, PAX6, EIF5A, NFKB1, PDX1, FOXO3, ZEB1, CNOT7, CBFB, KDM1A, EPC1, MLL5, GATA6, SUPT5H, MLL1, MYST4, ADAM9, EGR1, ZFP326, MECP2, TOPORS, DDIT3, UBE2N, ACVR2A, NCOA1, HNF4A, MED17, CD81, HIPK2, VEGFA, CAND1, NFE2L2, PIAS1, CLOCK, MED1, CLN6, ING2, ABHD5, AHCTF1, NFYC, PLAGL1, MEIS2, RB1CC1, NFAT5, ETV1, ING1, NKX2-2, TERF2, HNRNPAB, KLF5, KLF6, KAT2B, MAFB, BECN1, CREB1, TRIM28, SMAD4, SMAD1, ATXN7L3, SREBF2, YWHAH, AKTIP, ILF2, YAF2, ETS1, DCP1A, EBF1, THRAP3, NEUROD1, RBM14, PBX2, NCOR1, ARAP1
GO:0001704<br>formation of primary germ layer	9	0,51	3,79E-02	TWSG1, MACF1, NF2, FOXA2, TXNRD1, SMAD1, BMPR1A, NCKAP1, ACVR1
GO:0042981<br>regulation of apoptosis	68	3,82	4,00E-02	XRCC5, STAT5B, RBM5, FOXO3, SGMS1, PTEN, TGFB2, SIN3A, ATG5, RHOA, TPT1, PIK3CA, DNAJC5, DLG5, NDUFS3, CASP2, ROCK1, AARS, MECP2, LGALS12, POLB, CSDA, DDIT3, BCL2L11, DAPK1, BTG2, HIPK1, VEGFA, HIPK2, MNT, ADAM17, MAPK8, THOC1, BCLAF1, NR3C1, ITM2B, GCLM, IKBIP, GCH1, VDR, KRAS, SH3GLB1, RB1CC1, DIABLO, GLO1, TEX261, RASA1, PIK3R1, TRAF3, STAMBP, CFLAR, TM2D1, TGFBR1, TMBIM6, BIRC2, TAX1BP1, ATM, ATF5, BFAR, YWHAH, CDKN1B, VCP, BNIP3L, NEUROD1, APBB2, PDCD7, F2R, DAP3
GO:0009150<br>purine ribonucleotide metabolic process	19	1,07	4,20E-02	ADSS, ATP1B1, ATP1B3, ATP5B, AK1, ATP6AP1, ATP11A, ATP1A1, ATP6V0B, RP2H, ATP6V1A, ATP2B2, ATP6V0E2, ATP2A2, ATP2A3, ATP9A, ATP5L, ATP6V0D1, ATP8A1
GO:0009260<br>ribonucleotide biosynthetic process	18	1,01	4,26E-02	ADSS, ATP1B1, ATP1B3, ATP6AP1, ATP5B, ATP11A, ATP1A1, ATP6V0B, RP2H, ATP6V1A, ATP2B2, ATP6V0E2, ATP2A2, ATP2A3, ATP9A, ATP5L, ATP6V0D1, ATP8A1

Biological process	*Gene Count*	*Percentage*	*p-Value*	*Genes induced in RasGrf1 KO pancreatic islets (from Additional file 1: Table S1)*
GO:0006164 purine nucleotide biosynthetic process	21	1,18	4,61E-02	ADSS, ATP1B1, ATP1B3, ATP5B, ATP6AP1, ADCY6, ATP11A, ATP1A1, ATP6V0B, RP2H, MTHFD1, ATP6V1A, ATP2B2, ATP6V0E2, ADCY9, ATP2A2, ATP2A3, ATP9A, ATP5L, ATP6V0D1, ATP8A1
GO:0006913 nucleocytoplasmic transport	16	0,90	4,70E-02	XPO1, NUP160, IPO8, HNRNPA1, IPO4, IPO5, TSC2, KPNA6, NOP58, MLXIP, PPP3CA, KPNA4, XPO7, KPNA3, KPNB1, ARAP1
GO:0006259 DNA metabolic process	53	2,98	4,86E-02	XRCC5, MMS19, HMGN1, XRCC6, POT1A, CTCF, INTS3, NONO, TOP1, ANKRD17, MLL5, RAD21, SIN3A, MCM7, ORC4L, SLK, CTGF, MLL1, CHTF8, TOPBP1, POLB, MBD1, PURB, TNKS2, MCM6, UBE2N, TBRG1, SMARCAL1, TNFAIP1, MED1, RAD23B, RAG1AP1, WRNIP1, CHEK1, OBFC2A, RBX1, RPA1, ORC2L, CHD1L, ORC6L, FBXO6, BAZ2A, TERF2, SSRP1, DNMT3A, SMC6, ATM, SMC3, FOXP1, ATRX, SFPQ, DNMT1, PARP1
GO:0032507 maintenance of protein location in cell	6	0,34	4,87E-02	SEH1L, MORC3, TNRC6A, KDELR1, SUFU, OS9
GO:0043388 positive regulation of DNA binding	9	0,51	4,90E-02	UBE2N, IRAK1, MTDH, HIPK1, HIPK3, HIPK2, BRD4, IKBKB, CALM1
GO:0043067 regulation of programmed cell death	68	3,82	4,94E-02	XRCC5, STAT5B, RBM5, FOXO3, SGMS1, PTEN, TGFB2, SIN3A, ATG5, RHOA, TPT1, PIK3CA, DNAJC5, DLG5, NDUFS3, CASP2, ROCK1, AARS, MECP2, LGALS12, POLB, CSDA, DDIT3, BCL2L11, DAPK1, BTG2, HIPK1, VEGFA, HIPK2, MNT, ADAM17, MAPK8, THOC1, BCLAF1, NR3C1, ITM2B, GCLM, IKBIP, GCH1, VDR, KRAS, SH3GLB1, RB1CC1, DIABLO, GLO1, TEX261, RASA1, PIK3R1, TRAF3, STAMBP, CFLAR, TM2D1, TGFBR1, TMBIM6, BIRC2, TAX1BP1, ATM, ATF5, BFAR, YWHAH, CDKN1B, VCP, BNIP3L, NEUROD1, APBB2, PDCD7, F2R, DAP3
GO:0048514 blood vessel morphogenesis	28	1,57	4,94E-02	GNA13, NRP1, WASF2, DICER1, TIPARP, NAA15, CDH2, ELK3, PTEN, TGFB2, PTK2, ATG5, AGGF1, CTGF, TGFA, RHOB, RASA1, KLF5, PTPRJ, SGPL1, FLT1, TGFBR1, ARHGAP24, UBP1, JUNB, VEGFA, ACVR1, SMARCA4
GO:0060052 neurofilament cytoskeleton organization	4	0,22	5,05E-02	NDEL1, VPS54, NEFH, NEFM

Biological process	*Gene Count*	*Percentage*	*p-Value*	*Genes induced in RasGrf1 KO pancreatic islets (from Additional file 1: Table S1)*
GO:0060347 heart trabecula formation	4	0,22	5,05E-02	RBP4, FKBP1A, EGLN1, SMARCA4
GO:0046885 regulation of hormone biosynthetic process	3	0,17	5,05E-02	KDM1A, ATP1A1, NR3C1
GO:0043524 negative regulation of neuron apoptosis	10	0,56	5,22E-02	KRAS, ROCK1, HIPK2, AARS, MECP2, RHOA, DNAJC5, GCLM, RASA1, F2R
GO:0008064 regulation of actin polymerization or depolymerization	10	0,56	5,22E-02	ACTR3, ARPC1A, ARPC2, CAPZA2, RAC1, VIL1, RDX, SPNA2, RASA1, DSTN
GO:0031056 regulation of histone modification	5	0,28	5,24E-02	UBE2N, MECP2, CTCF, NCOR1, MLL1
GO:0001707 mesoderm formation	8	0,45	5,77E-02	TWSG1, MACF1, NF2, TXNRD1, SMAD1, BMPR1A, NCKAP1, ACVR1

Biological process	***Gene Count***	***Percentage***	***p-Value***	***Genes induced in RasGrf1 KO pancreatic islets (from Additional file 1: Table S1)***
GO:0045814 negative regulation of gene expression, epigenetic	6	0,34	5,81E-02	EPC1, DNMT3A, TRIM27, MECP2, BAZ2A, SMARCA4
GO:0030832 regulation of actin filament length	10	0,56	5,82E-02	ACTR3, ARPC1A, ARPC2, CAPZA2, RAC1, VIL1, RDX, SPNA2, RASA1, DSTN
GO:0007498 mesoderm development	11	0,62	5,98E-02	ACVR2A, TWSG1, MACF1, NF2, VEGFA, TXNRD1, MESDC2, SMAD1, BMPR1A, NCKAP1, ACVR1
GO:0010557 positive regulation of macromolecule biosynthetic process	64	3,59	6,18E-02	MEF2A, ELF1, FOXA2, TBK1, STAT5B, PAX6, EIF5A, NFKB1, PDX1, FOXO3, ZEB1, CNOT7, CBFB, KDM1A, EPC1, MLL5, GATA6, MLL1, MYST4, EGR1, ZFP326, MECP2, TOPORS, DDIT3, NCOA1, HNF4A, MED17, VEGFA, HIPK2, CAND1, NFE2L2, CLOCK, MED1, ING2, AHCTF1, NFYC, PLAGL1, MEIS2, NFAT5, ETV1, ING1, NKX2-2, HNRNPAB, KLF5, KLF6, KAT2B, MAFB, CREB1, TRIM28, SMAD4, SMAD1, ATXN7L3, SREBF2, YWHAH, ILF2, ETS1, YAF2, DCP1A, EBF1, THRAP3, NEUROD1, RBM14, PBX2, ARAP1
GO:0043623 cellular protein complex assembly	17	0,95	6,19E-02	XPO1, MTSS1, CAPZA2, AHCTF1, IPO8, CALR, TFAM, TAF10, PTK2, IPO4, IPO5, 2610030H06RIK, TUBB5, XPO7, COX18, KPNB1, TES
GO:0019320 hexose catabolic process	10	0,56	6,45E-02	ALDOA, PFKL, PKM2, ENO2, PDHA1, PFKM, DLAT, OGDH, GAPDH, HIBADH
GO:0031018 endocrine pancreas development	5	0,28	6,46E-02	INSM1, FOXA2, NEUROD1, PDX1, NKX2-2

Biological process	*Gene Count*	*Percentage*	*p-Value*	*Genes induced in RasGrf1 KO pancreatic islets (from Additional file 1: Table S1)*
GO:0050821<br>protein stabilization	5	0,28	6,46E-02	MORC3, TBRG1, STXBP1, APBB2, NCKAP1
GO:0031016<br>pancreas development	8	0,45	6,54E-02	INSM1, HHEX, INVS, FOXA2, XBP1, NEUROD1, PDX1, NKX2-2
GO:0032956<br>regulation of actin cytoskeleton organization	11	0,62	6,58E-02	ACTR3, ARPC1A, ARPC2, CAPZA2, RAC1, VIL1, RHOA, RDX, SPNA2, RASA1, DSTN
GO:0006884<br>cell volume homeostasis	4	0,22	6,70E-02	ANXA7, SLC12A7, SLC12A2, ADD1
GO:0016486<br>peptide hormone processing	4	0,22	6,70E-02	PCSK2, ECE2, ECE1, SCG5
GO:0030833<br>regulation of actin filament polymerization	9	0,51	6,92E-02	ACTR3, ARPC1A, ARPC2, CAPZA2, RAC1, VIL1, RDX, SPNA2, RASA1
GO:0051348<br>negative regulation of transferase activity	10	0,56	7,12E-02	DUSP19, CDKN1B, NF2, HIPK3, PKIG, DUSP16, TSC2, SPRED2, DNAJC3, CDK5RAP1
GO:0060537<br>muscle tissue development	20	1,12	7,30E-02	ZFAND5, RBP4, DICER1, TIPARP, EGLN1, FKBP1A, MBNL1, CACNB4, PTEN, FLNB, ITGB1, FOXP1, TGFB2, GPHN, ATG5, GATA6, DNER, RHOA, PPP3CA, F2R

Biological process	*Gene Count*	*Percentage*	*p-Value*	*Genes induced in RasGrf1 KO pancreatic islets (from Additional file 1: Table S1)*
GO:0048332 mesoderm morphogenesis	8	0,45	7,37E-02	TWSG1, MACF1, NF2, TXNRD1, SMAD1, BMPR1A, NCKAP1, ACVR1
GO:0007242 intracellular signaling cascade	104	5,84	7,41E-02	CTNNAL1, GNA13, STK38, STAT5B, ADCY6, ITPKB, IQGAP1, MLL5, EIF4EBP2, RAB28, CTGF, PIK3C3, SPRED2, PIK3CA, RAPGEF4, TLK2, DEPDC6, BCR, ROCK1, ROCK2, PCLO, BRAP, MAP4K3, MAPK1, RASGRF1, UBR5, RAB14, MAPK8, ASB3, PIAS1, ARL8B, RAB10, IRAK1BP1, ARFRP1, RAB6, CHEK1, AZI2, DUSP19, KRAS, RAC1, DUSP16, MAP2K1, RABIF, TRIO, SMAD1, D10ERTD610E, ATM, TULP4, ARF1, ULK1, ARF4, TSC2, RHOT1, RIT1, IKBKB, CACNA1D, WASF2, ARL5A, NISCH, RHOA, RHOB, TGFA, GKAP1, SRPK2, PIK3C2A, RAB4A, PI4KA, ARHGEF16, G3BP2, PRKCI, ADIPOR1, DAPK1, PRKD1, HIPK1, GNB1, HIPK2, CD81, RASD1, RAB3D, NR3C1, RGS11, MAP3K3, RB1CC1, PKD2, RAB11A, PKD1, DCX, RAB2A, GDI1, RAB2B, GDI2, SPSB1, TRIM23, RCAN3, RACGAP1, YWHAH, ADCY9, DUSP1, RPS6KA2, WSB2, RAB34, RAB22A, JAK1, F2R
GO:0031399 regulation of protein modification process	23	1,29	8,55E-02	IBTK, IRAK1, FECH, IMPACT, MECP2, SMAD4, CTCF, FKBP1A, UBQLN1, YWHAE, UBE2N, ACVR2A, ATG5, AKTIP, HNF4A, HIPK3, PRKAR1B, RB1CC1, CD81, RAPGEF4, PIAS1, NCOR1, MLL1
GO:0060343 trabecula formation	4	0,22	8,57E-02	RBP4, FKBP1A, EGLN1, SMARCA4
GO:0051283 negative regulation of sequestering of calcium ion	4	0,22	8,57E-02	IBTK, PKD2, FKBP1A, F2R
GO:0051282 regulation of sequestering of calcium ion	4	0,22	8,57E-02	IBTK, PKD2, FKBP1A, F2R
GO:0051209 release of sequestered calcium ion into cytosol	4	0,22	8,57E-02	IBTK, PKD2, FKBP1A, F2R

Biological process	*Gene Count*	*Percentage*	*p-Value*	*Genes induced in RasGrf1 KO pancreatic islets (from Additional file 1: Table S1)*
GO:0031328<br>positive regulation of cellular biosynthetic process	65	3,65	8,90E-02	HSP90AB1, ELF1, MEF2A, FOXA2, TBK1, STAT5B, PAX6, EIF5A, NFKB1, PDX1, FOXO3, ZEB1, CNOT7, CBFB, KDM1A, EPC1, MLL5, GATA6, MLL1, MYST4, EGR1, ZFP326, MECP2, TOPORS, DDIT3, NCOA1, HNF4A, MED17, VEGFA, HIPK2, CAND1, NFE2L2, CLOCK, MED1, ING2, AHCTF1, NFYC, PLAGL1, MEIS2, NFAT5, ETV1, ING1, NKX2-2, HNRNPAB, KLF5, KLF6, KAT2B, MAFB, CREB1, TRIM28, SMAD4, SMAD1, ATXN7L3, SREBF2, YWHAH, ILF2, ETS1, YAF2, DCP1A, EBF1, THRAP3, NEUROD1, RBM14, PBX2, ARAP1
GO:0006006<br>glucose metabolic process	20	1,12	9,13E-02	ALDOA, RBP4, PFKL, PFKM, PDX1, DLAT, PCK2, OGDH, HIBADH, PCX, PPP1R2, NISCH, PKM2, GYG, GAA, ENO2, PIK3CA, PDHA1, GAPDH, AGL
GO:0033500<br>carbohydrate homeostasis	8	0,45	9,20E-02	CYB5R4, FOXA3, NEUROD1, PTCH1, PFKM, FOXO3, PDX1, NCOR2
GO:0031647<br>regulation of protein stability	6	0,34	9,20E-02	MORC3, TBRG1, STXBP1, APBB2, PTEN, NCKAP1
GO:0000070<br>mitotic sister chromatid segregation	5	0,28	9,30E-02	CHMP1A, NIPBL, SEH1L, AKAP8, SMC4
GO:0006643<br>membrane lipid metabolic process	11	0,62	9,32E-02	SGPL1, UGT8A, PSAP, SGPP1, UGCG, SMPD1, SGMS1, MTAP7, B4GALT6, GAL3ST1, CLN6
GO:0007517<br>muscle organ development	24	1,35	9,50E-02	ZFAND5, RBP4, MTSS1, DICER1, TIPARP, FKBP1A, EGLN1, MBNL1, CACNB4, PTEN, FLNB, ITGB1, FOXP1, TGFB2, FXR1, GPHN, ATG5, GATA6, DNER, RHOA, ETV1, SCN8A, PPP3CA, F2R
GO:0035051<br>cardiac cell differentiation	7	0,39	9,90E-02	ECE2, ATG5, GATA6, DICER1, ITGB1, FOXP1, TGFB2
