# Supplementary material for: Transcriptional profiling reveals functional links between RasGrf1 and Pttg1 in pancreatic beta cells
Source: BMC Genomics. 2014 Nov 25;15:1019. doi: 10.1186/1471-2164-15-1019 (PMC4301450; doi:10.1186/1471-2164-15-1019)
Supplement: Supplementary file 5 — Additional file 5: Table S3A: Altered KEGG pathways identified by DAVID analysis of down-regulated (S3A), differentially expressed genes in pancreatic islets of RasGrf1 knockout mice. The DAVID functional annotation tool (http://david.abcc.ncifcrf.gov/) to identify statistically significant functional associations linking particular gene subsets contained within the list of repressed loci occurring in RasGrf1 KO pancreatic islets (Additional file 1: Table S1, FDR=0.084) to specific KEGG pathways (Kyoto Encyclopaedia of Genes and Genomes; http://www.genome.jp/kegg). The “KEGG Pathway” column identifies the signaling pathway annotated in each case to the corresponding group of loci listed in the column labeled “Genes repressed in RasGrf1 KO pancreatic islets (from Additional file 1 : Table S1)”. The column labeled “Gene Count” indicates the specific number of genes linked to the indicated signaling pathway within the list of repressed genes included in Table S1 (Additional file 1). The values under the column “Percentage” are calculated by referring the “Gene Count” numbers to the total number of genes recognized by DAVID (1943, out of a total 1953 genes, corresponding to 2268 probesets) within that list. The column labeled “p-value” refers to the statistical significance of the functional associations identified, and contains p-values calculated cases using the Hypergeometric Distribution and subsequently corrected by implementing the False Discovery Rate method [90]. (PDF 172 KB) [file 12864_2014_6838_MOESM5_ESM.pdf]

**Table S3A. Altered KEGG pathways identified by DAVID analysis of down-regulated, differentially expressed genes in pancreatic islets of RasGrf1 knockout mice.**

The DAVID functional annotation tool (http://david.abcc.ncifcrf.gov/) to identify statistically significant functional associations linking particular gene subsets contained within the list of repressed loci occurring in RasGrf1 KO pancreatic islets (Additional file 1: Table S1, FDR=0.084) to specific KEGG pathways (Kyoto Encyclopaedia of Genes and Genomes; www.genome.jp/kegg). The "*KEGG Pathway*" column identifies the signaling pathway annotated in each case to the corresponding group of loci listed in the column labeled "*Genes repressed in RasGrf1 KO pancreatic islets (from Additional file 1: Table S1)*". The column labeled "*Gene Count*" indicates the specific number of genes linked to the indicated signaling pathway within the list of repressed genes included in Additional file 1: Table S1. The values under the column "*Percentage*" are calculated by referring the "*Gene Count*" numbers to the total number of genes recognized by DAVID (1943, out of a total 1953 genes, corresponding to 2268 probesets) within that list. The column labeled "*p-value*" refers to the statistical significance of the functional associations identified, and contains p-values calculated cases using the Hypergeometric Distribution and subsequently corrected by implementing the False Discovery Rate method (Hochberg and Benjamini, 1990).

| *KEGG Pathway* | *Gene Count* | *%* | *p Value* | *Genes repressed in RasGrf1 KO pancreatic islets (from Additional file 1: Table S1)* |
|---|---|---|---|---|
| Cytokine-cytokine receptor interaction | 59 | 3,04 | 2,00E-08 | IL6ST, IL13, CXCR3, ACVR1B, TNFRSF11A, CLCF1, IFNG, CCR10, IL1B, TPO, PRL, LTB, LTA, EGFR, IL18RAP, IL25, TNFRSF14, EDAR, IL24, CD40, IL21, CCR8, CCR6, TNFSF13B, CD40LG, IFNB1, PDGFRA, IL12A, PDGFRB, NGFR, EDA, IFNAB, TNF, CCL2, TNFRSF25, TNFRSF12A, CCR1, CSF1, CCL4, CCL6, TNFRSF1A, TNFRSF1B, IFNA1, IL17B, IL10RA, TNFRSF19, FIGF, CD27, CSF1R, AMHR2, IL2RB, IL7, MET, TNFSF11, CXCL14, EPOR, BMPR1B, BMP7, MPL |
| MAPK signaling pathway | 58 | 2,99 | 1,00E-06 | FGF18, FGF17, GNA12, NFKB2, MAP3K7, ACVR1B, MAP3K8, MOS, PLA2G1B, IL1B, FGF2, MAP2K7, FGF3, FGF4, PRKCA, EGFR, CACNG8, CACNG7, CACNG6, RELB, FGF23, FGF22, FGF21, CACNG2, FLNC, MECOM, PRKCC, RASGRF2, PDGFRA, PLA2G2A, PDGFRB, HSPB1, GADD45B, NGF, FGFR2, TNF, FGFR3, CACNB1, MAP4K1, ELK1, TNFRSF1A, ELK4, RAC3, RASGRP1, SOS1, RASGRP2, PPP3CC, NFATC4, NFATC2, PLA2G10, MAP2K2, PTPN5, CACNA1S, CACNA2D2, DUSP3, MAPK13, CACNA1F, DUSP9 |
| Neuroactive ligand-receptor interaction | 56 | 2,88 | 2,71E-06 | GPR83, MCHR1, GRIK1, TACR3, GLRA3, TACR1, VIPR2, GHRHR, EDNRB, HTR1B, NMUR1, S1PR5, GRID2, HTR1D, PRL, HTR5A, CCKBR, NTSR1, NTSR2, GABRR2, SSTR5, CRHR2, GABRR1, CHRM3, CHRM1, F2, HTR6, GPR50, CTSG, CALCR, DRD3, ADORA2A, DRD2, TRHR, DRD4, PTH1R, BDKRB1, P2RY4, HRH3, HRH2, PRSS2, GRPR, NPFFR2, ADRA2B, GABRA2, GABRA1, OPRL1, PTGFR, P2RX6, LTB4R1, P2RX1, ADRA1B, HTR2B, HTR2C, GH, OPRD1 |
| Calcium signaling pathway | 45 | 2,32 | 2,73E-06 | ADCY3, SLC8A3, ADCY4, GNA15, TACR3, ADORA2A, ADCY8, ERBB2, TACR1, TRHR, BDKRB1, ITPKA, EDNRB, PDE1B, HRH2, GRPR, PPP3CC, NOS2, IGH-VJ558, HTR5A, PRKCA, EGFR, SLC8A2, NOS1, CCKBR, SPHK1, NTSR1, PTGFR, CACNA1S, PRKCC, IGHG, P2RX6, CHRM3, P2RX1, CAMK4, CHRM1, HTR6, CALM4, ADRA1B, PDGFRA, PDGFRB, CACNA1F, HTR2B, HTR2C, MYLK |
| Pathways in cancer | 62 | 3,19 | 3,39E-05 | FGF18, WNT3A, FGF17, NFKB2, GLI2, SHH, GLI1, ACVR1B, NOS2, MMP1A, WNT6, FGF2, FGF3, FGF4, PRKCA, EGFR, WNT10B, RXRG, LEF1, FGF23, FADD, FGF22, FGF21, MECOM, PRKCC, PDGFRA, PDGFRB, LAMC1, ITGA2B, FGFR2, BID, WNT16, WNT5B, FGFR3, ERBB2, EGLN3, NFKBIA, SFPI1, TCF7L2, SUFU, TPM3, LAMB3, RAC3, SOS1, BCL2, RUNX1, FIGF, TRAF4, WNT8A, CSF1R, WNT8B, MAP2K2, MET, FZD1, ITGA2, FZD6, WNT2B, LAMA2, WNT7B, CDKN1A, RASSF1, PTCH2 |
| Arrhythmogenic right ventricular cardiomyopathy (ARVC) | 22 | 1,13 | 6,18E-05 | CACNG8, CACNG7, CACNG6, CACNB1, ITGA2, GJA1, LEF1, ACTN1, ACTN2, ACTN3, CACNG2, ITGB3, ITGA4, CACNA1S, TCF7L2, CACNA2D2, LAMA2, DSG2, ITGB6, CACNA1F, SGCA, ITGA2B |

| *KEGG Pathway* | *Gene Count* | *%* | *p Value* | *Genes repressed in RasGrf1 KO pancreatic islets (from Additional file 1: Table S1)* |
|---|---|---|---|---|
| Dilated cardiomyopathy | 25 | 1,29 | 6,63E-05 | ADCY3, ADCY4, TNF, MYL3, ADCY8, CACNB1, ITGB3, TPM3, ITGB6, IGH-VJ558, CACNG8, CACNG7, CACNG6, ITGA2, MYH7, ITGA4, CACNG2, TNNI3, CACNA2D2, CACNA1S, LAMA2, IGHG, CACNA1F, SGCA, ITGA2B |
| Basal cell carcinoma | 18 | 0,93 | 8,00E-05 | WNT10B, WNT16, WNT5B, WNT3A, FZD1, LEF1, GLI2, TCF7L2, SHH, SUFU, FZD6, WNT2B, GLI1, WNT7B, PTCH2, WNT6, WNT8A, WNT8B |
| Hedgehog signaling pathway | 16 | 0,82 | 7,51E-04 | DHH, WNT10B, WNT16, WNT5B, WNT3A, GLI2, SHH, SUFU, GLI1, WNT2B, WNT7B, PTCH2, BMP7, WNT6, WNT8A, WNT8B |
| Hypertrophic cardiomyopathy (HCM) | 21 | 1,08 | 9,52E-04 | TNF, MYL3, CACNG8, CACNG7, CACNG6, CACNB1, ITGA2, MYH7, CACNG2, ITGB3, ITGA4, TNNI3, CACNA1S, CACNA2D2, TPM3, LAMA2, ACE, ITGB6, CACNA1F, SGCA, ITGA2B |
| Natural killer cell mediated cytotoxicity | 27 | 1,39 | 1,08E-03 | IFNAB, BID, KLRA16, PRF1, KLRC2, TNF, CD247, IFNA1, RAC3, KLRA8, SOS1, IFNG, PPP3CC, NFATC4, NFATC2, IGH-VJ558, TYROBP, PRKCA, PTPN6, MAP2K2, NCR1, PRKCC, HCST, IGHG, IFNB1, KLRB1C, LCP2 |
| Melanogenesis | 23 | 1,18 | 1,66E-03 | ADCY3, PRKCA, ADCY4, WNT16, WNT10B, WNT5B, GNAO1, CREB3, MAP2K2, ADCY8, WNT3A, FZD1, LEF1, TCF7L2, PRKCC, FZD6, WNT2B, EDNRB, WNT7B, CALM4, WNT6, WNT8A, WNT8B |
| Hematopoietic cell lineage | 20 | 1,03 | 2,40E-03 | FCER2A, CD3G, TNF, IL7, CSF1, ITGA2, MME, ITGB3, ITGA4, FCGR1, GP9, IGHG, CD19, CD2, IL1B, TPO, EPOR, IGH-VJ558, ITGA2B, CSF1R |
| Purine metabolism | 31 | 1,60 | 2,87E-03 | ADCY3, XDH, ADCY4, POLR2F, POLR2E, ENPP1, ADCY8, ENPP3, NT5C1B, POLA2, CANT1, PDE6C, PDE1B, PDE4A, ENTPD5, GUCY1A3, ENTPD6, ENTPD4, ENTPD1, PAPSS2, POLR1A, PDE10A, PDE3A, PDE6H, PDE6G, POLR3D, POLD3, NME4, PDE7B, PDE2A, PKLR |
| Gap junction | 19 | 0,98 | 7,35E-03 | ADCY3, PRKCA, EGFR, ADCY4, TUBB2A, ADCY8, DRD2, MAP2K2, TUBA3A, GJA1, PRKCC, TUBA8, SOS1, PDGFRA, GUCY1A3, PDGFRB, TUBB6, HTR2B, HTR2C |
| Wnt signaling pathway | 28 | 1,44 | 9,44E-03 | CER1, WNT16, WNT5B, NKD2, WNT3A, TCF7L2, MAP3K7, RAC3, FRAT1, PPP3CC, NFATC4, SOX17, NFATC2, WNT6, WNT8A, WNT8B, PRKCA, TBL1XR1, WNT10B, FZD1, LEF1, PRKCC, FZD6, WNT2B, DKK4, WNT7B, SFRP1, SIAH1A |
| Regulation of actin cytoskeleton | 37 | 1,91 | 1,26E-02 | FGFR2, FGF18, FGFR3, FGF17, GNA12, PIP5K1C, BDKRB1, MYL10, ITGB3, IQGAP1, RAC3, SOS1, ITGB6, MOS, FGF2, FGF3, FGF4, EGFR, LIMK1, MAP2K2, ITGA2, ACTN1, FGF23, NCKAP1L, ACTN2, FGF22, FGF21, ITGA4, ACTN3, MYH9, CHRM3, CHRM1, F2, PDGFRA, PDGFRB, MYLK, ITGA2B |
| Fc gamma R-mediated phagocytosis | 20 | 1,03 | 1,37E-02 | PRKCA, DNM3, WASF3, PPAP2C, NCF1, LIMK1, HCK, SPHK1, PIP5K1C, PRKCD, FCGR1, PRKCC, IGHG, MYO10, FCGR2B, GAB2, INPP5D, PPAP2A, DNM1, IGH-VJ558 |
| Toll-like receptor signaling pathway | 20 | 1,03 | 1,52E-02 | IFNAB, TNF, MAP2K2, NFKBIA, TLR4, FADD, CD40, TLR6, CCL4, TLR7, TLR9, MAP3K7, IRAK4, IFNA1, MAPK13, IFNB1, MAP3K8, IL12A, IL1B, MAP2K7 |
| Focal adhesion | 34 | 1,75 | 1,54E-02 | ERBB2, PIP5K1C, ELK1, COL2A1, MYL10, ITGB3, LAMB3, RAC3, SOS1, BCL2, COMP, ITGB6, COL6A2, COL11A2, FIGF, COL11A1, PRKCA, EGFR, PARVG, MET, ITGA2, ACTN1, ACTN2, ITGA4, ACTN3, FLNC, COL5A1, PRKCC, LAMA2, PDGFRA, PDGFRB, LAMC1, MYLK, ITGA2B |
| Complement and coagulation cascades | 16 | 0,82 | 2,05E-02 | F12, F10, C3, C4B, CBX3, BDKRB1, PROC, FGG, C4BP, FGA, SERPINF2, KLKB1, F2, SERPINC1, C2, CFD |
| Fc epsilon RI signaling pathway | 17 | 0,88 | 2,13E-02 | PRKCA, TNF, PLA2G10, MAP2K2, IL13, PRKCD, IGHG, GAB2, MAPK13, RAC3, SOS1, PLA2G2A, PLA2G1B, INPP5D, MAP2K7, IGH-VJ558, LCP2 |
| Adherens junction | 16 | 0,82 | 2,30E-02 | EGFR, PTPN6, WASF3, ERBB2, MET, LEF1, ACTN1, ACTN2, ACTN3, TCF7L2, IQGAP1, MAP3K7, ACVR1B, SORBS1, RAC3, INSR |
| ECM-receptor interaction | 17 | 0,88 | 2,37E-02 | ITGA2, COL2A1, ITGB3, ITGA4, COL5A1, SDC3, GP9, LAMA2, SDC1, LAMB3, COMP, ITGB6, COL6A2, LAMC1, COL11A2, COL11A1, ITGA2B |
| Glycine, serine and threonine metabolism | 9 | 0,46 | 2,41E-02 | CHDH, SHMT1, SDS, TDH, BHMT, PHGDH, SARDH, AGXT, GLDC |
| Cardiac muscle contraction | 16 | 0,82 | 2,85E-02 | FXYD2, CACNG8, MYL3, CACNG7, CACNG6, CACNB1, MYH7, ATP1A2, CACNG2, TNNI3, CACNA1S, CACNA2D2, COX5B, TPM3, GM15393, CACNA1F |
| Sphingolipid metabolism | 10 | 0,51 | 4,40E-02 | SPTLC1, PPAP2C, SPHK1, GALC, PPAP2A, SMPD4, NEU2, SMPD3, GLB1, ASAH2 |
| GnRH signaling pathway | 18 | 0,93 | 4,62E-02 | ADCY3, PRKCA, EGFR, ADCY4, PLA2G10, ADCY8, MAP2K2, ELK1, MMP14, CACNA1S, PRKCD, MAPK13, SOS1, PLA2G1B, PLA2G2A, CALM4, CACNA1F, MAP2K7 |

| ***KEGG Pathway*** | ***Gene Count*** | ***%*** | ***p Value*** | ***Genes repressed in RasGrf1 KO pancreatic islets (from Additional file 1: Table S1)*** |
|---|---|---|---|---|
| Glycosphingolipid biosynthesis | 7 | 0,36 | 4,83E-02 | B4GALT2, ST3GAL4, B3GNT4, B3GALT1, ST8SIA1, FUT1, GGTA1 |
