# Supplementary material for: Transcriptional profiling reveals functional links between RasGrf1 and Pttg1 in pancreatic beta cells
Source: BMC Genomics. 2014 Nov 25;15:1019. doi: 10.1186/1471-2164-15-1019 (PMC4301450; doi:10.1186/1471-2164-15-1019)
Supplement: Supplementary file 6 — Additional file 6: Table S3B: Altered KEGG pathways identified by DAVID analysis of up-regulated, differentially expressed genes in pancreatic islets of RasGrf1 knockout mice. The DAVID functional annotation tool (http://david.abcc.ncifcrf.gov/) to identify statistically significant functional associations linking particular gene subsets contained within the list of induced loci occurring in RasGrf1 KO pancreatic islets (Additional file 1: Table S1, FDR=0.084) to specific KEGG pathways (Kyoto Encyclopaedia of Genes and Genomes; http://www.genome.jp/kegg). The “KEGG Pathway” column identifies the signaling pathway annotated in each case to the corresponding group of loci listed in the column labeled “Genes repressed in RasGrf1 KO pancreatic islets (from Additional file 1 : Table S1)”. The column labeled “Gene Count” indicates the specific number of genes linked to the indicated signaling pathway within the list of induced genes included in Table S1 (Additional file 1). The values under the column “Percentage” are calculated by referring the “Gene Count” numbers to the total number of genes recognized by DAVID (1777, out of a total 1799 genes, corresponding to 2256 probesets) within that list. The column labeled “p-value” refers to the statistical significance of the functional associations identified, and contains p-values calculated cases using the Hypergeometric Distribution and subsequently corrected by implementing the False Discovery Rate method [90]. (PDF 166 KB) [file 12864_2014_6838_MOESM6_ESM.pdf]

Table S3B. Altered KEGG pathways identified by DAVID analysis of up-regulated, differentially expressed genes in pancreatic islets of RasGrf1 knockout mice.

The DAVID functional annotation tool (http://david.abcc.ncifcrf.gov/) to identify statistically significant functional associations linking particular gene subsets contained within the list of induced loci occurring in RasGrf1 KO pancreatic islets (Additional file 1: Table S1, FDR=0.084) to specific KEGG pathways (Kyoto Encyclopaedia of Genes and Genomes; www.genome.jp/kegg). The "*KEGG Pathway*" column identifies the signaling pathway annotated in each case to the corresponding group of loci listed in the column labeled "*Genes repressed in RasGrf1 KO pancreatic islets (from Additional file 1: Table S1)*". The column labeled "*Gene Count*" indicates the specific number of genes linked to the indicated signaling pathway within the list of induced genes included in Additional file 1: Table S1. The values under the column "*Percentage*" are calculated by referring the "*Gene Count*" numbers to the total number of genes recognized by DAVID (1777, out of a total 1799 genes, corresponding to 2256 probesets) within that list. The column labeled "*p-value*" refers to the statistical significance of the functional associations identified, and contains p-values calculated cases using the Hypergeometric Distribution and subsequently corrected by implementing the False Discovery Rate method (Hochberg and Benjamini, 1990).

KEGG Pathway	***Gene Count***	***%***	***p Value***	***Genes induced in RasGrf1 KO pancreatic islets (from Additional file 1: Table S1)***
Ubiquitin mediated proteolysis	32	1,80	5,22E-06	UBE3B, UBE2G1, SAE1, KEAP1, UBE3C, SKP1A, RBX1, CUL3, UBE2D3, ITCH, FBXW11, TRIP12, UBE4A, ANAPC5, CDC23, HERC4, HERC1, UBE2L3, BIRC2, RFWD2, UBE2N, TRIM37, HUWE1, UBE2K, UBA1, UBA2, UBR5, PIAS2, PIAS1, CUL4B, UBE2S, UBE2E2
Spliceosome	29	1,63	1,80E-05	CHERP, CRNKL1, CCDC12, NHP2L1, TRA2B, TRA2A, SNRPD1, SMNDC1, SF3B2, HNRNPA3, SF3B1, HNRNPK, PRPF8, BAT1A, RBM25, HSPA8, PRPF40A, RBM22, BCAS2, EFTUD2, CDC5L, HNRNPA1, HNRNPU, AQR, THOC2, SNRNP27, PUF60, PRPF38B, THOC1
Lysosome	27	1,52	6,52E-05	ATP6AP1, LGMN, AP3S2, CTSA, ACP2, ATP6V0B, GLB1, IDS, AP3M1, TPP1, NAGA, AP3D1, ATP6V0D1, AP3B1, CTSZ, PSAP, GUSB, M6PR, MANBA, GNS, CTSL, LAMP1, LAMP2, SMPD1, GAA, NEU1, CTSB
Oocyte meiosis	24	1,35	6,60E-04	YWHAZ, MAP2K1, ANAPC5, PPP2R5A, PPP2R5C, ADCY6, PPP3R1, YWHAB, CDC23, SKP1A, YWHAE, ITPR1, SMC3, RBX1, 1500003O03RIK, MAPK1, YWHAG, YWHAH, SLK, ADCY9, RPS6KA2, PPP3CA, FBXW11, CALM1
SNARE interactions in vesicular transport	12	0,67	7,62E-04	STX6, STX7, STX18, USE1, BET1, SEC22B, VAMP4, VAMP3, VAMP2, SNAP23, GOSR1, YKT6
Cell cycle	25	1,40	1,33E-03	YWHAZ, E2F5, CHEK1, SKP1A, RBX1, TGFB2, ORC2L, MCM7, RAD21, ORC4L, ORC6L, ANAPC5, CCNH, YWHAB, SMAD4, CDC23, YWHAE, SMC3, ATM, MCM6, YWHAG, HDAC2, CDKN1B, YWHAH, CCND2
Citrate cycle (TCA cycle)	10	0,56	2,23E-03	SDHA, PCX, ACO1, SUCLG2, CS, DLD, PDHA1, DLAT, OGDH, PCK2
Proteasome	12	0,67	4,88E-03	PSMB5, PSMF1, PSMB7, PSMD14, PSMC5, PSMA6, PSMD11, PSMC1, PSMD1, PSMD2, PSME4, PSMD7
Sphingolipid metabolism	11	0,62	6,29E-03	SGPL1, UGT8A, SGPP1, UGCG, SMPD1, NEU1, SGMS1, B4GALT6, PPAP2B, GAL3ST1, GLB1
Renal cell carcinoma	15	0,84	7,21E-03	MAP2K1, EGLN2, FLCN, TGFB2, RBX1, MAPK1, KRAS, PAK2, PAK3, ETS1, RAC1, VEGFA, PIK3CA, TGFA, PIK3R1

KEGG Pathway	*Gene Count*	*%*	*p Value*	*Genes induced in RasGrf1 KO pancreatic islets (from Additional file 1: Table S1)*
Pancreatic cancer	15	0,84	9,29E-03	MAP2K1, TGFBR1, SMAD4, NFKB1, TGFB2, MAPK1, KRAS, RAC1, VEGFA, PIK3CA, TGFA, JAK1, MAPK8, IKBKB, PIK3R1
Maturity onset diabetes of the young	8	0,45	1,07E-02	HHEX, HNF4A, FOXA2, FOXA3, PAX6, NEUROD1, PDX1, NKX2-2
Riboflavin metabolism	6	0,34	1,17E-02	MTMR2, RFK, ACP2, MTMR6, MTMR7, PHPT1
Chronic myeloid leukemia	15	0,84	1,48E-02	CTBP2, BCR, MAP2K1, TGFBR1, STAT5B, SMAD4, NFKB1, TGFB2, MAPK1, CDKN1B, HDAC2, KRAS, PIK3CA, IKBKB, PIK3R1
Neurotrophin signaling pathway	21	1,18	2,79E-02	IRAK1, YWHAZ, MAP2K1, YWHAB, NFKB1, FOXO3, YWHAE, ZFP110, MAPK1, YWHAG, YWHAH, KRAS, MAP3K3, RPS6KA2, RAC1, RHOA, PIK3CA, MAPK8, IKBKB, PIK3R1, CALM1
RNA degradation	12	0,67	2,99E-02	DIS3, PARN, CNOT6L, DCP1A, ENO2, CNOT2, CNOT1, HSPD1, CNOT7, CNOT6, ZCCHC7, CNOT4
N-Glycan biosynthesis	10	0,56	3,25E-02	STT3B, MGAT2, TUSC3, GANAB, MAN1A2, ALG1, ALG2, MAN1B1, DPAGT1, RPN2
VEGF signaling pathway	14	0,79	3,25E-02	MAP2K1, PPP3R1, 1500003O03RIK, MAPK1, PTK2, KRAS, PLA2G12A, RAC1, VEGFA, NFAT5, PLA2G6, PIK3CA, PPP3CA, PIK3R1
Adherens junction	14	0,79	3,25E-02	PTPRJ, PTPRF, TGFBR1, WASF2, SMAD4, LMO7, CTNND1, IQGAP1, MAPK1, TJP1, PVRL3, RAC1, RHOA, WASL
Wnt signaling pathway	23	1,29	3,32E-02	CSNK1A1, CTBP2, ROCK1, PPP2R5A, ROCK2, PPP2R5C, PPP3R1, SMAD4, SKP1A, RBX1, 1500003O03RIK, SFRP5, SENP2, PRICKLE1, CCND2, CSNK1E, RAC1, NFAT5, RHOA, MAPK8, PPP3CA, TBL1X, FBXW11
Thiamine metabolism	4	0,22	3,63E-02	MTMR2, MTMR6, MTMR7, PHPT1
Endocytosis	29	1,63	3,75E-02	CHMP2A, ARFGAP1, LDLR, STAM2, PIP5K1B, VPS37A, EEA1, SMAP2, AP2B1, SH3GLB1, RAB11A, ITCH, HSPA8, AP2M1, STAMBP, FLT1, TGFBR1, RAB4A, PRKCI, EPS15, CHMP1A, AP2A2, RABEP1, RAB22A, PDCD6IP, ARAP1, EPN2, F2R, RNF41
Cysteine and methionine metabolism	8	0,45	3,80E-02	GOT2, ADI1, DNMT3A, MAT1A, DNMT1, MTAP, AHCYL1, SMS
Fc gamma R-mediated phagocytosis	16	0,90	5,43E-02	MAP2K1, WASF2, PIP5K1B, AMPH, ARPC1A, MAPK1, MYO10, ARPC2, CFL2, RAC1, PLA2G6, PIK3CA, MARCKS, WASL, PPAP2B, PIK3R1
Phosphatidylinositol signaling system	13	0,73	6,05E-02	PIK3C2A, PIP5K1B, PI4KA, ITPKB, PTEN, ITPR1, PIK3C3, PIK3CA, PIP4K2A, PIP4K2C, PIK3R1, INPP5A, CALM1
MAPK signaling pathway	35	1,97	6,15E-02	PPM1A, PPP3R1, NFKB1, PPM1B, CACNB4, ATF2, TGFB2, 1500003O03RIK, KRAS, MAP3K4, PAK2, MAP3K3, PLA2G12A, JUND, RAC1, DUSP16, PPP3CA, RASA1, HSPA8, MAP2K1, TGFBR1, PTPRR, FLNB, TAB2, DDIT3, MAP4K3, MAPK1, DUSP1, RPS6KA2, RASGRF1, PLA2G6, MAPK8, IKBKB, CACNA1
