# Supplementary material for: Transcriptional profiling reveals functional links between RasGrf1 and Pttg1 in pancreatic beta cells
Source: BMC Genomics. 2014 Nov 25;15:1019. doi: 10.1186/1471-2164-15-1019 (PMC4301450; doi:10.1186/1471-2164-15-1019)
Supplement: Supplementary file 7 — Additional file 7: Table S4: Concurrent transcriptional alterations at different tissues of neuroectodermal origin in RasGrf1 KO mice. List of 29 different loci showing significant level of parallel, concomitant differential gene expression in two or more separate microarray hybridization analyses of samples from the indicated tissue locations of RasGrf1 KO mice. Origin of the microarray expression data for each tissue was as follows: Pancreatic islets: MOE430A arrays, Table S1 (Additional file 1) in this work. LCM-purified hippocampus pyramidal cells: MOE430A arrays, [32]. Retina: MOE430_2 arrays, [18]. Olfactory bulb, full hippocampus, and brain cortex: Mouse Exon 1.0 ST arrays, unpublished data. Red: Upregulation. Blue: Downregulation. LCM: Laser capture microdissection. No arrow: no significant transcriptional change detected. (PDF 99 KB) [file 12864_2014_6838_MOESM7_ESM.pdf]

Table S4. Concurrent transcriptional alterations at different tissues of neuroectodermal origin in RasGrf1 KO mice.

List of 29 different loci showing significant level of parallel, concomitant differential gene expression in two or more separate microarray hybridization analyses of samples from the indicated tissue locations of RasGrf1 KO mice. Origin of the microarray expression data for each tissue was as follows: Pancreatic islets: MOE430A arrays, Additional file 1: Table S1 in this work. LCM-purified hippocampus pyramidal cells: MOE430A arrays, [32]. Retina: MOE430_2 arrays, [18]. Olfactory bulb, full hippocampus, and brain cortex: Mouse Exon 1.0 ST arrays, unpublished data. Red: Upregulation. Blue: Downregulation. LCM: Laser capture microdissection. No arrow: no transcriptional change detected.

Loci from Table S1	Pancreatic islets	Retina	LCM-purified hippocampus pyramidal cells	Olfactory bulb	Cerebral cortex	Hippocampus
Aebp2	↑		↑			
Aldh7a1	↓	↓	↓			
Ap3s2	↑	↓				
ATM	↑	↑				
Clasp 2	↓		↓			
Col6a2	↓			↑		
Crb1	↓	↑				
Fam32a	↑		↑			
Fcgr2b	↓				↑	
Gdpd3	↓	↓				
Ivd	↑	↑				
Kcnj6	↓		↓			
Kpna3	↑		↓			
Mid1	↓					↓
Mtmr4	↑	↓				
Paip1	↑	↑				
Pja2	↑		↓			
Prpf4b	↑		↓			
Pttg1	↓	↓		↓	↓	↓
Rad23b	↑	↓				
Rbms	↑	↑				
Sptlc1	↓		↓			
Strap	↑		↓			
Stx3	↓	↑				
Supt16h	↓		↓			
Tcf12	↑		↓			
Ube2v2	↑		↓			
Ywhag	↑		↓			
Ywhaz	↑		↓			
