# Supplementary material for: Transcriptional profiling reveals functional links between RasGrf1 and Pttg1 in pancreatic beta cells
Source: BMC Genomics. 2014 Nov 25;15:1019. doi: 10.1186/1471-2164-15-1019 (PMC4301450; doi:10.1186/1471-2164-15-1019)
Supplement: Supplementary file 8 — Additional file 8: Table S5A: Transcription factors identified by functional annotation of differentially expressed, repressed genes in RasGrf1 KO pancreatic islets. The GeneCodis functional annotation tool (http://genecodis.cnb.csic.es/) was used to identify specific subsets within the list of repressed genes of RasGrf1 KO pancreatic islets (Additional file 1: Table S1, FDR=0.08; 2198 recognized repressed loci, out of a total 2268 probesets listed) that share co-occurrent functional annotations linking them to specific Transcription Factors (TransFac database) at high statistically significant p-values. The “Transcription Factor” column identifies individual transcription factors recognized by GeneCodis as capable of controlling expression of the corresponding groups of loci listed in each case under the column labeled “Genes repressed in RasGrf1 KO pancreatic islets”. The column labeled “Gene Count” indicates the specific number of genes identified in each of those groups. Values in the “Percentage” column are calculated referring the “Gene Count” column numbers to the total number of repressed, input genes (2198 repressed loci from Additional file 1: Table S1) recognized by the functional annotation software. The column labeled “p-value” refers to the statistical significance of the functional associations identified. (PDF 287 KB) [file 12864_2014_6838_MOESM8_ESM.pdf]

Table S5A. Transcription factors identified by functional annotation of differentially expressed, repressed genes in RasGrf1 KO pancreatic islets.

The GeneCodis functional annotation tool (http://genecodis.cnb.csic.es/) was used to identify specific subsets within the list of repressed genes of RasGrf1 KO pancreatic islets (Additional file 1: Table S1, FDR=0.08; 2198 recognized repressed loci, out of a total 2268 probesets listed) that share co-occurrent functional annotations linking them to specific Transcription Factors (TransFac database) at high statistically significant p-values. The “*Transcription Factor*” column identifies individual transcription factors recognized by GeneCodis as capable of controlling expression of the corresponding groups of loci listed in each case under the column labeled “*Genes repressed in RasGrf1 KO pancreatic islets*”. The column labeled “*Gene Count*” indicates the specific number of genes identified in each of those groups. Values in the “*Percentage*” column are calculated referring the “*Gene Count*” column numbers to the total number of repressed, input genes (2198 repressed loci from Additional file 1: Table S1) recognized by the functional annotation software. The column labeled “*p-value*” refers to the statistical significance of the functional associations identified.

Transcription factor	***Gene Count***	***Percentage***	***p-Value***	***Genes repressed in RasGrf1 KO pancreatic islets***
E12_Q6	274	12,47	5,96E-65	Clcnka,Rag1,Myo1e,Pdgfra,Gucy1a3,Coro2a,Barhl1,Kcnn3,Phox2a,Fxyd1,Atoh7,Arhgap8,Htr2c,Rgs3,Hoxa11,Tmod4,Cldn9,Gnb1l,Krt15,Esrrg,Cckbr,Pacsin3,Lsp1,Rgs6,Dscaml1,Shh,Gna12,Lef1,Bcl2,Gpt,Blnk,Gfap,Slc12a5,Irx4,Cuedc1,Hils1,Flnc,Otos,Itpka,Sox4,Cbfa2t3,Lmx1a,Cacna2d2,Vgll4,Syngr1,Dhrs3,Pax9,Eln,Dusp3,Nrip3,Maf1,Hoxb5,Mid1,Cldn8,Nkx2-5,Runx1,Heyl,Prop1,Ngfr,Ank1,Pou4f3,Kcnj4,Slc1a1,Pigr,Musk,Irx5,Lta,Doc2b,Slc22a8,B4galt5,Il7,Mark1,Slc29a2,Wnt10b,Fgf22,Rasgrf2,Plekhb1,Nfe2,Trim7,Col2a1,Klf13,Gadd45b,Chml,Actn3,Gnao1,Barx2,Prss2,Wnt6,Esr1,Gpr87,Hr,Slc4a1,Prkd2,Iqgap1,Prss12,Foxn1,Bmf,Hoxa7,Nos1,Stc1,Syngr3,Sftpc,Wnt16,Kcnj9,Pou2f3,Sfrp1,Dhh,Il17b,Slc4a8,Hoxb6,Prm1,Kcnd1,Nln,Sell,Myl3,Dsg2,Gjb2,Pcdh7,Gli2,Cdh16,Rarg,Crb3,Stc2,Otx2,Adcy8,Grid2,Gpha2,Syt3,Ms4a10,Dusp9,Mtx1,Tlk2,Lbx1,Gnl1,Npas2,Dll3,Tecta,Gjb4,Coro1c,Ube2l6,Bmpr1b,Prkcq,Rph3a,Srgap2,Mllt6,Chrm1,Hn1,Trim8,Itgb6,Clcn6,Ntng2,Ankrd1,Pitx1,Pdlim2,Tbx19,Arrdc1,Sema6a,Slc16a6,Elf5,Igf2,Bcl11b,Nppa,Fbp1,Acvr1b,Cxcl14,Cyhr1,Smoc2,Sema3b,Nr4a3,Bmp7,Kif3c,Dlx2,Gpd1,Polh,Dusp13,Slc25a10,Trex2,Smpd3,Egln3,Kcnip1,Serpinf2,Tacr1,Kcnip2,Hrh3,Tpm3,Ldb3,Tll2,Wnt7b,S100a9,Sema6c,Ptgfr,Klhl1,Mtss1,Nfatc4,Wnt2b,Sox17,Mycl1,Sorbs1,Odf3,Dkk4,Col12a1,Scn5a,Tle4,Epha2,Edar,Crat,Col11a2,Col18a1,Sema3a,Magi1,Gad2,Wnt3a,Slco2a1,Chrnd,Trim15,Magel2,Hira,Atp6v0a4,F12,Ccl20,Pde2a,Itga2,Gab2,Kcnh3,Evx1,Caln1,Eya3,Nkx6-2,Foxi1,Ddr1,Tcf15,Dll4,Sigirr,Mmp16,Ror1,Ptprcap,Pnkp,Ltb,Aak1,Cer1,Actn2,Cldn14,Cdh13,Elovl3,Bmx,Khdrbs2,Ovol1,Tead2,Drd3,Sox14,Ckb,Osbpl9,Cyp26a1,Nppc,Syt8,Tnni2,Crabp2,Hs6st3,Pthlh,Kcnd2,Sox10,Matn4,Gng13,Sdc1,Ckm,Tnfrsf12a,Pde3a,Adra1b,Ncam1,Dll1,Fgf4,Mylk,Rasgrp2,Ddit4l

LEF1_Q2	264	12,01	5,48E-49	Clcnka,Enam,Pdgfra,Thsd1,Rasl11b,Atoh7,Adamts4,Rgs3,Myo10,Hoxa11,Gnb1l,Ecel1,Esrrg,Pacsin3,Traf4,Gpc6,Col11a1,Shh,Gna12,Lhx5,Lef1,Mmp14,Hsf4,Dtna,Tnfrsf19,Il10ra,Ntn1,Nxn,Usf1,Cacna2d2,Vgll4,Sufu,Syngr1,Six3,Il21,Sln,Nt5c1b,Nrip3,Bik,Htr2b,Tbx3,Plp1,Hoxb5,Nfib,Mid1,Runx1,Ccr1,Sema7a,Pou4f3,Irx5,Fgf17,Tacstd2,Ppan,Chodl,Nfya,Slc22a8,Calcr,Wnt10b,Trim29,Cdca3,Sox5,Nfe2,Prm3,Col2a1,Klf13,Gpr12,Nog,Slc22a12,Barx2,Wnt6,Hr,Slc4a1,Dpysl4,Hspa12b,Ndst3,Wnt8b,Fzd1,Gas7,Hoxa7,Stc1,Cd160,Bmp1,Cyp19a1,Hmx1,Hoxb6,Ndrg2,Nln,Myl3,Il18rap,Dsg2,Kif5a,Angptl2,Chd1,Vax1,Kcnj1,Hoxb4,Cubn,Pcdh7,Gli2,Loxl3,Psme3,Cbx3,Rarg,Stc2,Otx2,Ipo11,Slc26a6,Rora,Nkx2-3,Mep1a,Grid2,Cdc42ep4,Lasp1,Mtx1,Adcyap1,Myh8,Cdk5r1,Tlk2,Lbx1,Epha7,Dhcr24,Dll3,Tecta,Coro1c,Enpp1,Inpp4a,Ddr2,Baz2a,Asb12,Bmpr1b,Srgap2,Mllt6,Chrm1,Fgf18,Lhx1,Hn1,Scgb3a2,Stard3,Atcay,Itgb6,Sox21,Kcnk4,F2,Sez6,Mme,Jph2,Sftpd,Sema6a,Cst8,Elf5,Fbxo31,Bcl11b,Nppa,Acvr1b,Pou1f1,Tlr7,Bach2,Homer2,Eif4g2,Slitrk1,Nr4a3,Bmp7,Kif3c,Rab8b,Mll2,Zdhhc14,Sos1,Eomes,Slc25a10,Hoxa4,Smpd3,Pitpnc1,Tpm3,Tll2,Met,Sema6c,Nfatc4,Gpr133,Egr3,Mycl1,Sorbs1,Col12a1,Dpf1,Lamb3,Sall3,Six4,Tle4,Hoxa5,Epha2,Edar,Lrrtm1,Scn3a,Col11a2,Ap1g2,Col5a1,Col18a1,Bcar3,Sema3a,Wnt3a,Serpinc1,T,Esrrb,Hira,Slc26a3,Gja1,Itga2,Abcc5,Ogg1,Gab2,Tnfsf13b,Eya3,Ddr1,Trpv4,Pde10a,Ddx5,Tcf15,Dll4,Mmp16,Ror1,Cdh13,Cdc25c,Bmx,Tcf7l2,Ptch2,Nr2f2,Ap4b1,Sf3b4,Khdrbs2,Gnat1,Wnt8a,Ovol1,Prrx1,Dlx1,Tead2,Col9a1,Stxbp4,Sox14,Ckb,Otc,Spo11,Mycbp,Ndst2,Atoh1,Rbp2,Map4k5,Hs6st3,Hoxc6,Sec14l2,Zfpm2,Pthlh,Plac1,Kcnd2,Sox10,Barx1,Sdc1,Fgfr3,Entpd7,Tnmd,Rpl27a,Smarcc1,Dll1,Wfdc2,Acy1,Slc6a1
MAZ_Q6	230	10,46	7,27E-46	Entpd1,Fgf3,Barhl1,Fxyd1,Tuba8,Htr2c,Adamts4,Rgs3,Cldn9,Lcn2,Ecel1,Krt15,Lcat,Esrrg,Pacsin3,Traf4,Myoz1,Blvrb,Col11a1,Shh,Rxrg,Klhdc3,Gpr3,Lef1,Mmp14,Slc12a5,Flnc,Il1rn,Itpka,Brdt,Krt13,Sox4,Slc6a12,Cbfa2t3,Usf1,Lmx1a,Cacng2,Vgll4,Nfkbia,Sufu,Ppap2a,Irx3,Tbx3,Hoxb5,Sema7a,Kcnj4,Cldn19,Adam15,Fgf17,Adcy4,Calcr,Jph1,Cdca3,Plekhb1,Sox5,Pcolce,Mycn,Hrk,Il16,Gadd45b,Gnao1,Fcgr2b,Esr1,Hr,Prkd2,Prss12,Foxn1,Bmf,Hoxa7,Nos1,Stc1,Mxd3,C4b,Bmp1,Hpca,Gmfg,Pou2f3,Erf,Hmx1,Dhh,Hoxb6,Frat1,Ndrg2,Fbxo36,Kcnd1,Tob2,Myl3,Rfx1,Chd1,Hoxb4,Pcdh7,Loxl3,Cbx3,Nfkb2,Rarg,Stc2,Otx2,Rora,Grid2,Gpha2,Syt3,Cdc42ep4,Plxdc2,Dusp9,Lasp1,Mtx1,Mrc2,Tlk2,Lbx1,Gdf15,Epha7,Npas2,Col25a1,Dll3,Mybph,Ascl2,Ddr2,Baz2a,Fxyd2,Tsnaxip1,Ptf1a,Mllt6,Chrm1,Ptbp1,Pitx3,Lhx1,Trim8,Fmo2,Kcns1,Ptpn5,Sox21,Cdkn1a,Amhr2,F2,Slc16a6,Cyyr1,Igf2,Bcl11b,Nppa,Cxcl14,Bach2,Homer2,Eif4g2,Slitrk1,Nr4a3,Bmp7,Il13,Zdhhc14,Eomes,Hoxa4,Smpd3,Pitpnc1,Kcnip2,Hrh3,Tpm3,Ldb3,Dock6,Pappa,Suv39h1,Chrd,Neto1,Klhl1,Rhobtb2,Nfatc4,Egr3,Sox17,Sorbs1,Col12a1,Arf4,Scn5a,Six4,Epha2,Phgdh,Lrrtm1,Col11a2,Col18a1,Qprt,Bcar3,Slco2a1,Il24,Fgfbp1,Kcna7,Map3k8,Pde2a,Cd151,Cpne6,Ogg1,Kcnh3,Mip,Xpo1,Evx1,Caln1,Hcn2,Supt16h,Fank1,Ddx5,Tcf15,Dll4,Ror1,Tnfrsf1a,Tnni3,Tcf7l2,Tead3,Nr2f2,Evc,Trh,Dlx1,Tead2,Drd3,Hnf4g,Adora2a,Cyp26a1,Ndst2,Kcnc3,Pgr,Tnni2,Hs6st3,Sec14l2,Zfpm2,Sox10,Sdc1,Padi1,Tnfrsf12a,Atp1a2,Hspb1,Smarcc1,Ncam1,Wfdc2,Fgf4,Rasgrp2

SP1_Q6	260	11,83	4,37E-43	Ace,Irak4,Thsd1,Fgf3,Phox2a,Atoh7,Klk8,Traf4,Gpc6,Blvrb,Gna12,Cyp24a1,Ier3,Klhdc3,Gpr3,Man1a2,Mmp14,Mrpl17,Gpt,Hsf4,Slc12a5,Tpcn1,Dyrk1b,Ntn1,Sox4,Rab2b,Pon2,Cbfa2t3,Atrn,Nxn,Rassf1,Usf1,Lmx1a,Nfkbia,Cdc37,Comtd1,Eln,Dusp3,Hspb7,Rad9b,Maf1,Tnf,Capn5,Irx3,Tbx3,Insr,Nfib,Casr,Nkx2-5,Srd5a2,Sema7a,Map2k7,Pou4f3,Cldn19,Lhx3,Irx5,Lta,Doc2b,Fgf17,Mgst3,Fut1,Nfya,Fgf22,B4galt2,Hcst,Cdca3,Homer1,Plekhb1,Sox5,Pcolce,Col2a1,Klf13,Shmt1,Itsn1,Hr,Prkd2,Slc25a13,Vps18,Iqgap1,Prss12,Gas7,Aldh7a1,Gng4,Vamp1,Mxd3,Hpca,Erf,Dhh,Gpr50,Timp1,Ndrg2,Relb,Map3k7,Fbxo36,Kcnd1,Tob2,Top2a,Dsg2,Rfx1,Phospho1,Vax1,Zfp36l2,Park2,Rgs20,Loxl3,Foxh1,Il6st,Nfkb2,Rarg,Crb3,Mxd4,Kcna1,Stc2,Slc26a6,Adcy8,Nkx2-3,Exo1,Cdc42ep4,Lasp1,Mtx1,Grik1,Cdk5r1,Tlk2,Gnl1,Epha7,Dll3,Coro1c,Ascl2,Inpp4a,Hsd17b7,Baz2a,Tbx5,Tsnaxip1,Hyal3,Gabra1,Mllt6,Cldn1,Pitx3,Fgf21,Hn1,Atp12a,Alox12,Stard3,Trim8,Cdkn1a,Amhr2,Pdlim2,Sez6,Spag4,Sema6a,Mst1,Mvd,Rem1,Igf2,Bcl11b,Fbp1,Cxcl14,Bach2,Eif4g2,Actn1,Sema3b,Nr4a3,Bmp7,Gpd1,Polh,Batf,Trex2,Hoxa4,Cit,Smpd3,Stx17,Kcnip1,Dlx3,Kcnip2,Hrh3,Rel,Tll2,Gprc5d,Suv39h1,Sema6c,Gldc,Neto1,Col7a1,Mtss1,Egr3,Wnt2b,Sox17,Cspg4,Cited4,Col12a1,Dpf1,Catsper2,Six4,Tle4,Six6,Ap1g2,Pmf1,Cnnm4,Kcnn1,Magi1,Pdlim7,Abcc3,Hs3st3b1,Creb3,Pdcd1,Ogg1,Gna15,Epor,Xpo1,Loxl4,Eya3,Fbn2,Sntb2,Supt16h,Ddx5,Dll4,Sigirr,Per3,Ppih,Cmklr1,Abhd1,Elk1,Tcf7l2,Tead3,Ptch2,Insm2,Nr2f2,Sf3b4,Ovol1,Trh,Prrx1,Tead2,Osbpl9,Slc35a5,Nppc,Ndst2,Rab3d,Kcnc3,Pgr,Ptpn6,Hs6st3,Hoxc6,Oprd1,Elac2,Zfpm2,Pthlh,Kcnd2,Gng13,Sdc1,Nell2,Entpd7,Tnfrsf12a,Crtac1,Hspb1,Lrpap1,Ncam1,Wfdc2,Fgf4,Acy1,Rasgrp2
NFAT_Q4_01	197	8,96	2,27E-41	Hmgcr,Myo1e,Pdgfra,Barhl1,Arhgap8,Adamts4,Rgs3,Hoxa11,Gnb1l,Ecel1,Esrrg,Pacsin3,Fyb,Ier3,Tmc1,Man1a2,Mmp14,Cuedc1,Dtna,Tnfrsf19,Flnc,Il1rn,Slc6a12,Cacng2,Vgll4,Nfkbia,Nr5a2,Dhrs3,Six3,Ccl4,Il21,Sln,Cacnb1,Hspb7,Ifng,Tnf,Tbx3,Nfib,Runx1,Sema7a,Pou4f3,Cldn19,Gabrr1,Irx5,Doc2b,Fgf17,Adcy4,Ppan,Chodl,B4galt5,Calcr,Rasgrf2,Rwdd3,Homer1,Ccl2,Sox5,Col2a1,Il16,Klf13,Nog,Gadd45b,Gnao1,Col16a1,Chdh,Satb2,Esr1,Prkd2,Slc25a13,Nrip2,Ccrl1,Prss12,Aspa,Gas7,Hoxa7,Nos1,Stc1,Tnfrsf1b,Pcdh12,Prf1,Pou2f3,Dhh,Il17b,Grwd1,Hoxb6,Ndrg2,Ptges,Tob2,Gtpbp1,Myl3,Angptl2,Gsta4,Vax1,Kcnj1,Hoxb4,Cdh16,Il6st,Nfkb2,Rarg,Pde1b,Kcna1,Stc2,Rora,Exo1,Mtx1,Myh8,Mrc2,Npas2,Sstr5,Lpin2,Tecta,Coro1c,Enpp1,Aqp2,Ddr2,Fxyd2,Tbx5,Hyal3,Pitx3,Lhx1,Trim8,Dbn1,Ntng2,Sox21,Cdkn1a,Mme,Sftpd,Sema6a,Slc16a6,Elf5,Rad23a,Slitrk1,Rgs1,Nr4a3,Enpp3,Il13,Kcne2,Rab8b,Mll2,Sos1,Eomes,Hoxa4,Smpd3,Pitpnc1,Nfatc2,Kcnip2,Tpm3,Ldb3,Mtss1,Nfatc4,Xpnpep2,Cspg4,Hesx1,Col12a1,Lamb3,Sall3,Adm,Six6,Hoxa5,Phgdh,Crat,Lrrtm1,Ndufb9,Scn3a,Bcar3,Sema3a,Serpinc1,Slc35a2,Esrrb,Map3k8,Hs3st3b1,Itga2,Tnfsf11,Xpo1,Caln1,Supt16h,Fank1,Dll4,Tnfrsf1a,Bcl2l14,Bmx,Khdrbs2,Ovol1,Mobp,Prrx1,Dlx1,Sox14,Hnf4g,Csrp3,Hs6st3,Plunc,Pthlh,Plac1,Sox10,Gata4,Atp1a2,Crtac1,Ddit4l

AP4_Q5	171	7,78	6,67E-41	Ace,Rag1,Grpr,Sh3bgrl3,Kcnn3,Tuba8,Arhgap8,Htr2c,Adamts4,Tmod4,Esrrg,Pacsin3,Lsp1,Traf4,Rgs6,Myoz1,Rxrg,Lef1,Irx4,Tpcn1,Dtna,Slc35c2,Tnfrsf19,Flnc,Sox4,Cbfa2t3,Rassf1,Usf1,Cacng2,Hspb3,Dbh,Dhrs3,Ccl4,Eln,Cacnb1,Tbx3,P2ry4,Plp1,Insr,Hoxb5,Mid1,Runx1,Sema7a,Ank1,Lhx3,Musk,Lta,Fgf17,Adcy4,Wnt10b,Jph1,Rasgrf2,Cdca3,Sox5,Mycn,Col2a1,Klf13,Actn3,Barx2,Itsn1,Wnt6,Esr1,Slc4a1,Nes,Tal2,Vps18,Bmf,Aspa,Wnt8b,Gas7,Vamp1,Bmp1,Hpca,Erf,Il17b,Fbxo36,Myl3,Angptl2,Kcnmb1,Adcy8,Scn4a,Lbx1,Dll3,Mybph,Aqp2,Baz2a,Anxa8,Prkcq,Pitx3,Cyp46a1,Fgf21,Hn1,Htr5a,Pdha2,Trim8,Atp6v1e2,Ntng2,Ankrd1,Pdlim2,Sez6,Sct,Cacna1s,Bcl11b,Nppa,Bach2,Eif4g2,Nr4a3,Gpd1,Eomes,Crb1,Slc8a3,Mos,Kcnip2,Tpm3,Ldb3,Dock6,Usp13,Extl1,Wnt7b,Chrd,Col7a1,Klhl1,Mtss1,Rhobtb2,Mgll,Wnt2b,Sorbs1,Ptprs,Arf4,Catsper2,Scn5a,Tle4,Phf7,Hoxa5,Epha2,Lrrtm1,Il24,Esrrb,Map3k8,Ogg1,Gab2,Asb16,Epor,Loxl4,Eya3,Chrng,Ddr1,Supt16h,Pde10a,Ddx5,Dll4,Aak1,Csf1r,Cdh13,Cmklr1,Ptch2,Dlx1,Tead2,Mycbp,Ndst2,Ptpn6,Tnni2,Hoxc6,Plac1,Sox10,Ckm,Atp1a2,Pde3a,Dll1,Mylk,Ddit4l
TATA_01	146	6,64	2,22E-31	Clca4,Ace,Enam,Slc2a4,Rasl11b,Sh3bgrl3,Kcnn3,Fxyd1,Atoh7,Rgs3,Hoxa11,Esrrg,Sult1b1,Pacsin3,Havcr2,Gpc6,Angptl4,Sprr1b,Galnt3,Lhx5,Aqp3,Irx4,Slc35c2,Il1rn,Otos,Ppp1r3a,Anxa1,Cacng2,Nfkbia,Hspb3,Nr5a2,Ccl4,Asb18,Hspb7,Ifng,Tnf,Tbx3,Hoxb5,Nfib,Mid1,Pou4f3,Lta,Mgst3,Jph1,Esr2,Sox5,Nog,Prss2,Satb2,Col10a1,Marco,Ndst3,Nos1,Stc1,Hoxb6,Cyp7a1,Ndrg2,Fbxo36,Top2a,Nr0b1,Zfp36l2,Hoxb4,Ifnb1,Rarg,Otx2,Rora,Grid2,Myh8,Lbx1,Gdf15,Epha7,Atp6v1b2,Bmpr1b,Amelx,Gabra1,Srgap2,Afp,Fgf21,Hn1,Afm,Cyyr1,Nppa,Pou1f1,Bach2,Homer2,Rgs1,Aldob,Nr4a3,Il13,Slc12a3,Prl,Blk,Eomes,Mos,Tacr1,Tpm3,Neu2,Pappa,Gprc5d,S100a9,Klhl1,Gpr133,Accn5,Hesx1,Dkk4,Tle4,Col11a2,Sema3a,Apoc2,Slco2a1,Art5,Pdlim7,Abcc6,Abcc5,Asb16,Mip,Ddx5,Actn2,Psca,Ptch2,Insm2,Nr2f2,Tnfrsf25,Dlx1,Stxbp4,Ckb,Cyp26a1,Nppc,Atoh1,Rbp2,Tnni2,Crabp2,Hoxc6,Plunc,Slc3a1,Pthlh,Ascl3,Ckm,Tnmd,Crtac1,Fgf4,Cyp1a2,Mylk,Lpo,Ddit4l,Coch
FOXO4_01	184	8,37	8,11E-31	Hs3st1,Myo1e,Enam,Entpd1,Pdgfra,Barhl1,Fxyd1,Rgs3,Hoxa11,Esrrg,Traf4,Rxrg,Lhx5,Bcl2,Irx4,Cuedc1,Dtna,Tnfrsf19,Ntn1,Nxn,Usf1,Anxa1,Cacna2d2,Cacng2,Vgll4,Dhrs3,Nt5c1b,Bik,Tbx3,Plp1,Insr,Hoxb5,Nfib,Runx1,Btg4,Slc1a1,Musk,Fgf17,Mgst3,Slc22a8,Mark1,Rwdd3,Sox5,Lcp2,Gpr12,Gnao1,Satb2,Hr,Slc4a1,Dpysl4,Prkd2,Ccrl1,Bmf,Aspa,Stc1,Hoxb6,Nfe2l3,Nln,Chd1,Vax1,Hoxb4,Rgs20,Pcdh7,Il6st,Gkn1,Kcna1,Stc2,Otx2,Slc26a6,Rora,Grid2,Plxdc2,Mtx1,Mrc2,Lbx1,Epha7,Npas2,Lamc1,Alb,Coro1c,Mfap5,Aqp2,Ascl2,Cntnap4,Inpp4a,Tbx5,Gabra1,Mllt6,Lor,Lhx1,Scgb3a2,Trim8,Aqp9,Sox21,Htr1d,Dnajc5b,Pitx1,Kcne1,Cldn18,Sema6a,Slc16a6,Elf5,Bcl11b,Acvr1b,Adarb2,Rad23a,Homer2,Slitrk1,Nr4a3,Dlx2,Zdhhc14,Sos1,Crb1,Hoxa4,Smpd3,Rp1,Stx17,Pitpnc1,Kcnip2,Myh7,Pappa,Sema6c,Serpina6,Ndufb2,Mgll,Mycl1,Sorbs1,Hesx1,Dkk4,Sall3,Hoxd1,Epha2,Edar,Lrrtm1,Scn3a,Ap1g2,Kcnn1,Sema3a,Phf3,Gad2,Gpr63,Serpinc1,Pdgfrb,Itga2,Abcc5,Gab2,Evx1,Caln1,Fbn2,Sntb2,Ptcra,Gabra2,Tcf15,Htr1b,Aak1,Cdc25c,Bmx,Tcf7l2,Nr2f2,Sf3b4,Prrx1,Dlx1,Tead2,Col9a1,Hnf4g,Ckb,Spo11,Cyp26a1,Atoh1,Rbp2,Map4k5,Fga,Hs6st3,Hoxc6,Zfpm2,Tnmd,Ncam1,Dll1,Pde6c,Mylk,Slc6a1,Agr2,Ddit4l,Coch

PAX4_03	145	6,60	3,35E-30	Entpd1,Slc2a4,Rasl11b,Barhl1,Kcnn3,Arhgap8,Traf4,Blvrb,Shh,Klhdc3,Mmp14,Rarres2,Slc12a5,Dtna,Flnc,Krt13,Ntn1,Cbfa2t3,Trpv2,Prdx2,Lmx1a,Cacng2,Eln,Nrip3,Cacnb1,Capn5,Tbx3,Nfib,Sema7a,Map2k7,Oprl1,Mpl,Fgf17,B4galt2,Homer1,Klf13,Gadd45b,Gnao1,Wnt6,Hr,Slc4a1,Prkd2,Iqgap1,Bmf,Hspa12b,Abcg4,S100a1,Sfrp1,Erf,Il17b,Hoxb6,Nfe2l3,Ndrg2,Ptges,Fbxo36,Tob2,Myl3,Rfx1,Phospho1,Ager,Hoxb4,Psme3,Kcnmb1,Cbx3,Rarg,Kcna1,Otx2,Rora,Lasp1,Mtx1,Grik1,Lbx1,Dhcr24,Gjb4,Mybph,Inpp4a,Baz2a,Tbx5,Mllt6,Chrm1,Trim8,Clcn6,Htr1d,Cdkn1a,Sez6,Acr,Sema6a,Igf2,Bach2,Rad23a,Eif4g2,Sema3b,Nr4a3,Bmp7,Kif3c,Zdhhc14,Trex2,Hoxa4,Pitpnc1,Sphk1,Rgs14,Tpm3,Procr,Sema6c,Chrd,Guca2b,Mtss1,Rhobtb2,Nfatc4,Gpr133,Egr3,Dpf1,Six4,Hoxa5,Col11a2,Bcar3,Wnt3a,Hsd17b1,Itga2,Cpne6,Creb3,Evx1,Foxi1,Supt16h,Dll4,Pnkp,Aak1,Abhd1,Tead3,Prrx1,Dlx1,Tead2,Col9a1,Sox14,Adora2a,Rab3d,Ptpn6,Tnni2,Hs6st3,Hoxc6,Zfpm2,Sox10,Crtac1,Agr2,Rasgrp2
ETS2_B	127	5,78	2,55E-28	Myo1e,Irak4,Pdgfra,Map4k1,Htr2c,Adamts4,Rgs3,Myo10,Hoxa11,Gnb1l,Lsp1,Cd6,Aqp3,Blnk,Cuedc1,Slc35c2,Flnc,Il1rn,Snx22,Rassf1,Cacng2,Fgfr2,Cdc37,Cd19,Mid1,Ncf2,Prop1,Ccr1,Fxyd5,Mpl,Fpgs,Musk,Adam15,Adcy4,Mark1,Hcst,Ccl2,Fcgr2b,Gpr87,Marco,Gas7,Slamf1,Nos1,Figf,Prf1,Erf,Timp1,Hoxb6,Il18rap,Kcnj1,Pcdh7,Fgf23,Loxl3,Rora,Fcho1,Fzd6,Npas2,Dhcr24,Dll3,Coro1c,Enpp1,Baz2a,Mllt6,Lhx1,Slc20a2,Cdkn1a,Pdlim2,Actn1,Nr4a3,Klrc2,Bmp7,Il13,Gpd1,Polh,Mll2,Trex2,Hoxa4,Pitpnc1,Rgs14,Nfatc2,Tpm3,Bub1b,Tll2,S100a9,Sema6c,Mgll,Egr3,Cited4,Arf4,Hoxd1,Stat4,Epha2,Lgals4,Scn3a,Ap1g2,Cnnm4,Gad2,Il24,Tyrobp,Pla1a,Ccl20,Pdgfrb,Creb3,Gab2,Tnfsf13b,Evx1,Caln1,Tcf15,Dll4,Sigirr,Ptprcap,Slpi,Ltb,Cxcr3,Muc13,Cmklr1,Bmx,Tead3,Elk4,Sox14,Ckb,Ptpn6,Tnni2,Gata4,Mark4,Pde3a,Pde6c
AP1_C	126	5,73	8,48E-28	Pdgfra,Ids,Rasl11b,Arhgap8,Rgs3,Hoxa11,Cldn9,Krt15,Esrrg,Pacsin3,Itm2b,Angptl4,Sprr1b,Gpr3,Gfap,Synpo,Dtna,Flnc,Il1rn,Krt13,Slc6a12,Hspb3,Syngr1,Dhrs3,Il21,Nt5c1b,Nrip3,Hspb7,Runx1,Adam15,Mgst3,Mark1,Trim29,Il16,Gadd45b,Col16a1,Wnt6,Esr1,Gpr87,Foxn1,Wnt8b,Stc1,Aebp1,Figf,Sftpc,Prf1,Nfe2l3,Ndrg2,Kcnd1,Vax1,Hoxb4,Gli2,Gkn1,Pde1b,Crb3,Stc2,Ipo11,Ly6d,Adcy8,Tlk2,Lamc1,Mybph,Ascl2,Baz2a,Atp6v1b2,Chrm1,Gjb5,Lor,Trim8,Htr1d,Dnajc5b,Cdkn1a,Pdlim2,Mme,Sftpd,Slc16a6,Cxcl14,Sema3b,Dusp13,Pitpnc1,Ldb3,Usp13,Wnt7b,Pappa,Procr,Met,Col7a1,Nfatc4,Lynx1,Cspg4,Acpp,Bdkrb1,Lamb3,Scn5a,Adm,Camk4,Epha2,Phf3,Capn12,Il24,Gja1,Pdgfrb,Hs3st3b1,Cd151,Itga2,Gab2,Cdh23,Kcnh3,Loxl4,Ddr1,Xdh,Csf1r,Dlx1,Rab3d,Odf1,Map4k5,Syt8,Dlg4,Tnfrsf12a,Mark4,Cst7,Pde6h,Dll1,Mylk,Agr2,Tff1
MEF2_02	102	4,64	1,26E-27	Hs3st1,Pdgfra,Slc2a4,Gabrr2,Kcnn3,Fxyd1,Atoh7,Rgs3,Tmod4,Esrrg,Pacsin3,Adhfe1,Shh,Mmp14,Slc12a5,Dtna,Slc35c2,Sox4,Ppp1r3a,Dbh,Sln,Arr3,Hoxb5,Nfib,Musk,Mark1,Rwdd3,Sox5,Nog,Gadd45b,Gnao1,Barx2,Itsn1,Esr1,Slc12a1,Slamf1,Stc1,Kcnj9,Hoxb6,Ndrg2,Myl3,Kcnj1,Hoxb4,Slc26a6,Grik1,Adcyap1,Lbx1,Epha7,Enpp1,Cntnap4,Rph3a,Gabra1,Mllt6,Ntng2,Cdkn1a,Gp9,Jph2,Sema6a,Tlr7,Slitrk1,Rgs1,Bmp7,Trhr,Slc8a3,Kcnip2,Usp13,Extl1,Mgll,Cited4,Sall3,Tle4,Six6,Hoxa5,Kcnn1,Phf3,Mpg,Lama2,Recql,Kcna7,Art5,Esrrb,Asb16,Dll4,Aak1,Cldn14,Iapp,Prrx1,Drd3,Hnf4g,Ndst2,Csrp3,Atoh1,Map4k5,Wfdc1,Tnni2,Zfpm2,Ckm,Gata4,Nell2,Atp1a2,Hrasls,Ncam1

MYOD_Q6	115	5,23	3,02E-27	Myo1e,Gucy1a3,Sh3bgrl3,Kcnn3,Atoh7,Arhgap8,Htr2c,Rgs3,Cldn9,Pacsin3,Dscaml1,Rxrg,Lef1,Gfap,Slc12a5,Irx4,Cuedc1,Tpcn1,Tnfrsf19,Cbfa2t3,Usf1,Syngr1,Cacnb1,Mid1,Runx1,Heyl,Ank1,Lhx3,Musk,Irx5,Doc2b,Fgf17,Rasgrf2,Sox5,Klf13,Actn3,Barx2,Wnt6,Hr,Klk4,Hoxa7,Vamp1,Pcdh12,Hpca,Sftpc,Erf,Il17b,Slc4a8,Sell,Rfx1,Mxd4,Grid2,Mtx1,Tlk2,Dll3,Mybph,Baz2a,Anxa8,Mllt6,Lor,Lhx1,Hn1,Sez6,Igf2,Cxcl14,Eif4g2,Gpd1,Slc25a10,Crb1,Slc8a3,Trex2,Rps18,Tacr1,Ldb3,Chrd,Col7a1,Mycl1,Hesx1,Ctsg,Col12a1,Six6,Hoxa5,Crat,Chrnd,Hira,Ccl20,Itga2,Gab2,Epor,Xpo1,Nkx6-2,Foxi1,Sntb2,Chrng,Dnm3,Supt16h,Dll4,Cer1,Csf1r,Cdh13,Nr2f2,Ovol1,Tead2,Sox14,Mycbp,Cyp26a1,Nppc,Tnni2,Hs6st3,Kcnd2,Ckm,Tnmd,Ncam1,Dll1,Ddit4l
ERR1_Q2	119	5,41	7,94E-26	Slc2a4,Kcnn3,Fxyd1,Rgs3,Lcn2,Esrrg,Apex2,Dnm1,Gfap,Synpo,Itpka,Sox4,Rassf1,Tlr4,Oxa1l,Syngr1,Nr5a2,Comtd1,Dusp3,Hspb7,Nr1i2,Insr,Hoxb5,Fgg,Sema7a,Adam15,Mgst3,Dnajb8,Nfya,Slc29a2,Sox5,Nog,Pdzk1,Slc25a13,Vamp1,Syngr3,Gmfg,Ndrg2,Gtpbp1,Nr0b1,Myl3,Kif5a,Rfx1,Prrx2,Gjb2,Pcdh7,Cdh16,Elf3,Slc26a6,Grid2,Limk1,Epha7,Npas2,Mfap5,Tbx5,Bmpr1b,Rph3a,Mllt6,Afp,Lor,Lhx1,Prdm9,Itgb6,Ntng2,Cox5b,Cdkn1a,F2,Gtf2ird1,Polr3d,Sct,Jph2,Acr,Slc16a6,Ppp3cc,Calcb,Slitrk1,Mrpl34,Kcnip2,Hrh3,Tpm3,Ldb3,Bub1b,Tll2,Catsper2,Scn5a,Six6,Hoxa5,Ap1g2,Pacsin1,Bcar3,Kcnn1,Capn12,Slco2a1,Serpinc1,Esrrb,Ccl20,Slc26a3,Creb3,Abcc5,Dnase1,Htr1b,Cdh13,Tnni3,Tcf7l2,Sf3b4,Trap1,Dlx1,Ckb,Odf1,Map4k5,Crabp2,Fgfr3,Tnfrsf12a,Rpl27a,Crtac1,Mylk,Auh,Rasgrp2,Ddit4l
NF1_Q6	104	4,73	5,86E-25	Slc2a4,Sh3bgrl3,Htr2c,Wisp2,Lcn2,Esrrg,Dscaml1,Lef1,Gfap,Cuedc1,Sox4,Ahsg,Nfkbia,Hspb3,Syngr1,Dhrs3,Cacnb1,P2ry4,Plp1,Nfib,Mid1,Runx1,Mpl,Musk,Irx5,Fgf17,Chodl,Il16,Klf13,Gnao1,Esr1,Hr,Stc1,C4b,Figf,Wnt16,Erf,Ndrg2,Slco1c1,Myl3,Ager,Akap4,Pcdh7,Rora,Grid2,Mtx1,Adcyap1,Myh8,Erbb2,Alb,Ascl2,Cntnap4,Srgap2,Fgf21,Stard3,Trim8,Aqp9,Atp6v1e2,Ntng2,Pitx1,Sema6a,Adarb2,Cxcl14,Bach2,Cyp17a1,Gpd1,Rab8b,Trex2,Rtn4ip1,Dlx3,Nfatc2,Tpm3,Met,Chrd,Mtss1,Mycl1,Cited4,Lamb3,Lrrtm1,Col11a2,Slco2a1,Magel2,Hira,Cpne6,Klf1,Gab2,Mip,Supt16h,Xdh,Ror1,Per3,Pnkp,Aak1,Dlx1,Ndst2,Rbp2,Map4k5,Syt8,Pthlh,Hrasls,Ncam1,Fgf4,Agr2,Ddit4l
CHX10_01	96	4,37	5,80E-23	Hs3st1,Entpd1,Pdgfra,Rasl11b,Barhl1,Kcnn3,Htr2c,Hoxa11,Pacsin3,Dscaml1,Ier3,Lhx5,Aqp3,Pitpnm2,Irx4,Lmx1a,Cacng2,Six3,Ppap2a,Sln,Nfib,Mid1,Prop1,Sema7a,Map2k7,Pou4f3,Irx5,Tacstd2,Dnajb8,Nfya,Esr2,Rwdd3,Sox5,Hrk,Gnao1,Col10a1,Wnt8b,Stc1,Bmp1,Sfrp1,Gtpbp1,Hoxb4,Prodh2,Otx2,Serpinb11,Rora,Myh8,Lbx1,Npas2,Lamc1,Tecta,Bmpr1b,Lamb1,Afp,Ntng2,Dnajc5b,Ankrd1,Pitx1,Sez6,Tbx19,Cyyr1,Adarb2,Bach2,Slitrk1,Dlx2,Prl,Eomes,Mef2b,Hoxa4,Pitpnc1,Sema6c,Mtss1,Hesx1,Col12a1,Adamts10,Sall3,Tle4,Hoxa5,Epha2,Lrrtm1,Col11a2,Pmf1,Sema3a,Wnt3a,Serpinc1,Ogg1,Tnfsf13b,Caln1,Dll4,Aak1,Ovol1,Prrx1,Dlx1,Pgr,Ckm,Nell2
AREB6_01	101	4,60	2,75E-22	Myo1e,Pdgfra,Wisp2,Adamts4,Esrrg,Lsp1,Shh,Ier3,Lhx5,Mmp14,Irx4,Hils1,Tnfrsf19,Il1rn,Rab2b,Lmx1a,Dhrs3,Cacnb1,Nfib,Nkx2-5,Sema7a,Map2k7,Btg4,Fgf17,Il7,Rasgrf2,Esr2,Sox5,Nfe2,Col2a1,Actn3,Bmf,Stc1,Sfrp1,Dhh,Hoxb6,Kcnd1,Ager,Hoxb4,Park2,Pcdh7,Cdh16,Crb3,Kcna1,Grid2,Syt3,Tlk2,Lbx1,Epha7,Npas2,Tecta,Cntnap4,Fxyd2,Gabra1,Mllt6,Chrm1,Hn1,Pdha2,Itgb6,Ntng2,Dnajc5b,Ankrd1,Sez6,Tbx19,Slc16a6,Bcl11b,Spdef,Bmp7,Rab8b,Dusp13,Slc8a3,Egln3,Tacr1,Myh7,S100a9,Lynx1,Mgll,Egr3,Lamb3,Six4,Wnt3a,Atp6v0a4,Kcnh3,Evx1,Loxl4,Ddr1,Dll4,Cer1,Cdh13,Tead3,Khdrbs2,Gnat1,Ovol1,Csrp3,Hs6st3,Hoxc6,Pthlh,Sox10,Nell2,Ncam1,Agr2

PITX2_Q2	89	4,05	3,76E-22	Sh3bgrl3,Barhl1,Tuba8,Adamts4,Hoxa11,Rxrg,Dnm1,Bcl2,Slc12a5,Sox4,Ppap2c,Lmx1a,Sufu,Syngr1,Dhrs3,Dusp3,Nrip3,Ifng,Irx3,Mid1,Runx1,Pou4f3,Irx5,Slc22a6,Hrk,Col2a1,Nog,Gadd45b,Actn3,Itsn1,Hr,Slc12a1,Tal2,Ndst3,Aspa,Stc1,Proc,Hoxb6,Ndrg2,Fbxo36,Tob2,Angptl2,Vax1,Kcnj1,Hoxb4,Gli2,Gkn1,Otx2,Rora,Grid2,Fzd6,Tlk2,Ddr2,Atp6v1b2,Amelx,Gabra1,Srgap2,Mllt6,Slc4a5,Cacna1s,Sema6a,Spdef,Prl,Rp1,Usp13,Mtss1,Cngb1,Hesx1,Tle4,Epha2,Ndufb9,Scn3a,Sema3a,Wnt3a,Slc35a2,Atp6v0a4,Pdlim7,Creb3,Mip,Myog,Supt16h,Dll4,Prrx1,Sox14,Nppc,Dlg4,Fgfr3,Pde6c,Mylk
HNF3_Q6	93	4,23	4,23E-21	Pdgfra,Rgs3,Hoxa11,Tmod4,Pacsin3,Itm2b,Blnk,Ntn1,Sox4,Ahsg,Pla2g1b,Cacng2,Cd19,Cldn8,Itih1,Map2k7,Pou4f3,Musk,Irx5,Adam15,Jph1,B4galt2,Sox5,Lcp2,Col2a1,Il16,Gadd45b,Gnao1,Prss2,Esr1,Hr,Slc12a1,Foxn1,Bmf,Proc,Sftpc,Nfe2l3,Cyp7a1,Vax1,Pla2g10,Otx2,Grid2,Grik1,Lbx1,Epha7,Gjb4,Ascl2,Cntnap4,Syn3,Chrm1,Kcnk4,Cdkn1a,Pdlim2,Havcr1,Sema6a,Elf5,Bcl11b,Adarb2,Rad23a,Homer2,Nr4a3,Zdhhc14,Serpina6,Nfatc4,Mgll,Sox17,Hesx1,Adamts10,Tle4,Sema3a,Slco2a1,Gab2,Gabra2,Htr1b,Cer1,Tcf7l2,Nr2f2,Drd3,Bpil1,Sox14,Hnf4g,Ndst2,Atoh1,Fga,Crabp2,Hoxc6,Matn4,Entpd7,Tnmd,Pde6h,Ncam1,Mylk,Slc6a1
OLF1_01	70	3,18	5,36E-21	Entpd1,Barhl1,Madcam1,Adamts4,Rgs3,Hoxa11,Esrrg,Cckbr,Traf4,Myoz1,Pitpnm2,Rab2b,Gipc3,Nfkbia,Dusp3,Cd19,Adcy4,Hcst,Trim29,Sox5,Tst,Nog,Gnao1,Hr,Adra2b,Hpca,Grwd1,Kcnd1,Psme3,Stc2,Plxdc2,Lasp1,Mtx1,Grik1,Adcyap1,Tlk2,Ascl2,Cntnap4,Baz2a,Fxyd2,Mllt6,Mpst,Cyp46a1,Trim8,Elf5,Sema3b,Zdhhc14,Smpd3,Wnt7b,Mtss1,Nfatc4,Mycl1,Dpf1,Col11a2,Col18a1,Pacsin1,Magel2,Pdgfrb,Ddr1,Dll4,Cmklr1,Prrx1,Dlx1,Odf1,Ptpn6,Pthlh,Ckm,Entpd7,Atp1a2,Acy1
FREAC2_01	98	4,46	3,68E-20	Slc2a4,Kcnn3,Hoxa11,Esrrg,Klhdc3,Galnt3,Nmur1,Clgn,Synpo,Cuedc1,Dtna,Tnfrsf19,Ntn1,Cacng2,Dhrs3,Il21,Bik,Insr,Nfib,Mid1,Cldn8,Runx1,Pou4f3,Gabrr1,Doc2b,Nfya,Jph1,Sox5,Lcp2,Il16,Gnao1,Satb2,Hr,Slc4a1,Aspa,Hoxa7,Mbtps1,Gmfg,Ndrg2,Nln,Chd1,Zfp36l2,Hoxb4,Il6st,Rarg,Mxd4,Kcna1,Slc26a6,Rora,Grid2,Slu7,Lasp1,Adcyap1,Tlk2,Erbb2,Anxa2,Mllt6,Trim8,Kcnk4,Cdkn1a,Slc16a6,Bcl11b,Bach2,Ptgis,Rgs1,Crb1,Trex2,Rps18,Rp1,Pitpnc1,Pappa,Klhl1,Mgll,Wnt2b,Mycl1,Hesx1,Sall3,Hoxa5,Ap1g2,Gpr63,Art5,Map3k8,Gja1,Asb16,Ddx5,Tcf15,Mmp16,Ror1,Ltb,Tcf7l2,Dlx1,Fen1,Map4k5,Hoxc6,Plunc,Mylk,Auh,Coch
RSRFC4_Q2	68	3,09	2,60E-19	Pdgfra,Slc2a4,Rgs3,Myoz1,Slc12a5,Ppp1r3a,Hspb3,Six3,Sln,Cacnb1,Arr3,Musk,Tacstd2,Mgst3,Slc29a2,Sox5,Hrk,Nog,Actn3,Satb2,Itsn1,Esr1,Col10a1,Slamf1,Stc1,Kcnj9,Ndrg2,Tob2,Myl3,Phospho1,Hoxb4,Ifnb1,Slc26a6,Adcyap1,Myh8,Tlk2,Epha7,Inpp4a,Trim8,Cdkn1a,Sema6a,Trhr,Slc8a3,Hoxa4,Tpm3,Usp13,Gpr133,Dkk4,Lrrtm1,Kcnn1,Slco2a1,Kcna7,Art5,Asb16,Loxl4,Myog,Cldn14,Tnni3,Prrx1,Drd3,Ckb,Csrp3,Wfdc1,Zfpm2,Ckm,Hspb1,Fgf4,Rasgrp2
TEF1_Q6	64	2,91	2,28E-17	Pdgfra,Sh3bgrl3,Rgs3,Traf4,Slc6a13,Klhdc3,Man1a2,Slc12a5,Atrn,Fgfr2,Mid1,Nnmt,Prop1,Cd2,Musk,Cdca3,Nog,Esr1,Nes,Foxn1,Abcg4,Vax1,Il6st,Nfkb2,Rarg,Elf3,Otx2,Slc26a6,Mtx1,Ovgp1,Erbb2,Lamc1,Mfap5,Anxa8,Stard3,Ankrd1,Cacna1s,Spdef,Smoc2,Nr4a3,Kcne2,Kcnip2,Myh7,Mtss1,Mgll,Lamb3,Six4,Epha2,Klk6,Col11a2,Wnt3a,T,Itga2,Loxl4,Ddr1,Dnm3,Ptprcap,Ovol1,Prrx1,Cyp26a1,Syt8,Fga,Adam33,Rasgrp2
CREL_01	44	2,00	4,29E-16	Madcam1,Traf4,Ier3,Cuedc1,Slc6a12,Nfkbia,Wnt10b,Hcst,Sox5,Gadd45b,Actn3,Gnao1,Col16a1,Gng4,Tnfrsf1b,Pcdh12,Pou2f3,Relb,Ptges,Ifnb1,Nfkb2,Stc2,Tsnaxip1,Mllt6,Slc16a6,Actn1,Il13,Sos1,Smpd3,Rel,Tpm3,Six4,Ndufb9,Col11a2,Ap1g2,Map3k8,Ccl20,Ddr1,Ltb,Csf1r,Tcf7l2,Nr2f2,Sox10,Gata4

NFKB_Q6	43	1,96	4,49E-16	Rgs3,Hoxa11,Prkcd,Il1rn,Slc6a12,Nfkbia,Sufu,Runx1,Fgf17,Sox5,Gnao1,Bmf,Hoxa7,Nos1,Ndrg2,Ptges,Ifnb1,Nfkb2,Stc2,Plxdc2,Lasp1,Fxyd2,Mllt6,Hcfc1,Actn1,Sema3b,Rel,Pappa,Epha2,Pla1a,Map3k8,Ogg1,Xpo1,Ddr1,Tcf15,Ltb,Nr2f2,Cyp26a1,Sec14l2,Pthlh,Sox10,Ascl3,Gata4
AML_Q6	53	2,41	1,77E-15	Rag1,Arhgap8,Wisp2,Cd6,Dyrk1b,Trpv2,Ccl4,Ifng,Hoxb5,Runx1,Ccr1,Btg4,Tacstd2,Ccl2,Sox5,Wnt8b,Commd5,Gpr50,Il17b,Hoxb6,Il18rap,Kcnj1,Hoxb4,Mtx1,Anxa8,Tbx5,Bmpr1b,Sla2,Slc16a6,Spdef,Slitrk1,Rgs1,Bmp7,Il13,Gpd1,Batf,Pitpnc1,Rgs14,Rel,Wnt7b,Pappa,Ptprs,Ap1g2,Cnnm4,Bcar3,Napsa,Sntb2,Supt16h,Cxcr3,Hoxc6,Gata4,Tnfrsf12a,Pde6h
MEIS1_01	83	3,78	5,69E-15	Entpd1,Pdgfra,Esrrg,Rgs6,Col11a1,Rxrg,Irx4,Tnfrsf19,Il1rn,Sox4,Usf1,Anxa1,Sufu,Dhrs3,Hspb7,Plp1,Runx1,Pou4f3,Chodl,Slc22a8,Jph1,Sox5,Il16,Gnao1,Barx2,Hr,Nrip2,Vps18,Abcg4,Bmp1,Prm1,Ager,Park2,Psme3,Mxd4,Stc2,Adcy8,Rora,Grik1,Tlk2,Gnl1,Baz2a,Atp6v1b2,Tbx5,Srgap2,Lhx1,Pdha2,Itgb6,Cacna1s,Slc16a6,Nppa,Bach2,Nr4a3,Smpd3,Kcnip2,Sema6c,Col7a1,Klhl1,Mtss1,Nfatc4,Mgll,Sorbs1,Cspg4,Dpf1,Tle4,Crat,Esrrb,Gab2,Xpo1,Evx1,Foxi1,Dnm3,Dll4,Tcf7l2,Tead3,Sox14,Ucp3,Padi1,Entpd7,Adra1b,Ncam1,Dll1,Coch
NFKAPPAB_01	41	1,87	5,82E-15	Madcam1,Kcnn3,Arhgap8,Hoxa11,Traf4,Il1rn,Ntn1,Slc6a12,Tnf,Lta,Fgf17,Wnt10b,Hcst,Sox5,Gadd45b,Actn3,Gnao1,Col16a1,Bmf,Gng4,Tnfrsf1b,Relb,Zfp36l2,Ifnb1,Nfkb2,Stc2,Hcfc1,Actn1,Il13,Smpd3,Col11a2,Map3k8,Kcnh3,Ddr1,Ltb,Nr2f2,Atoh1,Sec14l2,Sox10,Ascl3,Gata4
SREBP1_Q6	41	1,87	6,88E-15	Slc2a4,Kcnn3,Fxyd1,Hoxa11,Pacsin3,Traf4,Mmp14,Gfap,Flnc,Lmx1a,Cacnb1,B4galt2,Gnao1,Esr1,Slc4a1,Prkd2,Abcg4,Erf,Dhh,Grwd1,Ndrg2,Ager,Kcnmb1,Rarg,Grid2,Cdc42ep4,Lasp1,Lbx1,Inpp4a,Baz2a,Eif4g2,Rel,Guca2b,Nfatc4,Sorbs1,Six4,Ap1g2,Evx1,Ddr1,Tead2,Kcnk7
NFY_Q6_01	99	4,50	1,04E-14	Clcnka,Rag1,Myo1e,Enam,Ndufs8,Slc2a4,Barhl1,Gabrr2,Htr2c,Rgs3,Adhfe1,Shh,Tpcn1,Sox4,Fgfr2,Sufu,Irx3,Tbx3,Mid1,Runx1,Btg4,Chodl,Nfya,Prkd2,Slc25a13,Slc25a19,Wnt8b,Hoxa7,Vamp1,Stc1,Sfrp1,Hmx1,Gpr50,Hoxb6,Top2a,Gtpbp1,Vax1,Kif23,Psme3,Kcna1,Slc26a6,Myh8,Epha7,Dhcr24,Gjb4,Ascl2,Ddr2,Tsnaxip1,Amelx,Rph3a,Ptf1a,Gabra1,Chrm1,Gjb5,Pitx3,Lhx1,Tradd,Acr,Mvd,Adarb2,Eif4g2,Aldob,Dlx2,Rab8b,Slc8a3,Cit,Rtn4ip1,Egln3,Dlx3,Kcnip2,Tll2,Suv39h1,Serpina6,Nfatc4,Catsper2,Tle4,Six6,Pdgfrb,Tnfsf11,Ogg1,Klf1,Xpo1,Nkx6-2,Per3,Glra3,Cdc25c,Ptch2,Dlx1,Osbpl9,Imp4,Atoh1,Fbxl8,Crabp2,Hoxc6,Elac2,Tacc3,Entpd7,Ncam1,Fgf4
P53_02	40	1,82	1,62E-14	Lrdd,Gabrr2,Adamts4,Hoxa11,Krt15,Esrrg,Traf4,Dscaml1,Dtna,Dyrk1b,Sox4,Anxa1,Cacng2,Sema7a,Trim29,Sox5,Gadd45b,Myl3,Otx2,Mrc2,Slc1a3,Chrm1,Pitx3,Cdkn1a,Egfr,Bcl11b,Eif4g2,Slitrk1,Nr4a3,Pitpnc1,Rhobtb2,Cited4,Crat,Magel2,Abcc5,Mip,Ddr1,Prrx1,Hs6st3,Gata4
NFKB_C	42	1,91	1,76E-14	Arhgap8,Hoxa11,Lcn2,Lcat,Prkcd,Cuedc1,Il1rn,Slc6a12,Nfkbia,Cdc37,Plp1,Fgf17,Sox5,Gadd45b,Gnao1,Bmf,Gng4,Hoxb6,Ndrg2,Ptges,Ifnb1,Nfkb2,Lasp1,Tsnaxip1,Mllt6,Hcfc1,Sema3b,Il13,Smpd3,Pappa,Egr3,Lamb3,Col11a2,Pla1a,Map3k8,Ddr1,Ltb,Sec14l2,Pthlh,Sox10,Ascl3,Gata4
NFKAPPAB65_01	40	1,82	1,85E-14	Madcam1,Traf4,Ier3,Cuedc1,Slc6a12,Nfkbia,Fgf17,Wnt10b,Sox5,Gadd45b,Actn3,Col16a1,Bmf,Gng4,Tnfrsf1b,Pcdh12,Pou2f3,Relb,Ptges,Il6st,Ifnb1,Nfkb2,Tsnaxip1,Mllt6,Slc16a6,Il13,Smpd3,Rel,Six4,Ndufb9,Col11a2,Map3k8,Ddr1,Ltb,Csf1r,Nr2f2,Pthlh,Sox10,Ascl3,Gata4

HSF1_01	56	2,55	2,16E-14	Slc2a4,Asah2,Ecel1,Pacsin3,Tnfrsf19,Flnc,Cbfa2t3,Cacna2d2,Hoxb5,Pou4f3,Lhx3,Adam15,B4galt5,Wnt10b,Plekhb1,Chml,Wnt8b,Stc1,Chd1,Cbx3,Il6st,Otx2,Adcy8,Plxdc2,Grik1,Fxyd2,Cyp46a1,Hcfc1,Ptgis,Gipc2,Eif4g2,Rgs1,Nr4a3,Eomes,Nfam1,Nfatc4,Mgll,Egr3,Six4,Adm,Epha2,Sema3a,Gad2,Abcc5,Xpo1,Mmp16,Actn2,Glra3,Pip5k1c,Mobp,Spo11,Hs6st3,Oprd1,Pthlh,Hspb1,Rasgrp2
NFKB_Q6_01	43	1,96	3,39E-14	Madcam1,Arhgap8,Traf4,Prkcd,Il1rn,Ntn1,Slc6a12,Nfkbia,Lta,Fgf17,Il7,Sox5,Gadd45b,Nrip2,Bmf,Gng4,Tnfrsf1b,Pou2f3,Ndrg2,Relb,Ptges,Il6st,Ifnb1,Nfkb2,Lasp1,Actn1,Sema3b,Il13,Batf,Hoxa4,Smpd3,Rel,Tpm3,Pappa,Il12a,Ddr1,Ltb,Csf1r,Nr2f2,Pthlh,Ascl3,Gata4,Lpo
PU1_Q6	65	2,96	5,26E-14	Irak4,Arhgap8,Mrpl17,Bcl2,Cuedc1,Flnc,Rab2b,Cbfa2t3,Trpv2,Tlr4,Vgll4,Itga2b,Eln,Tnf,Htr2b,Ncf2,Lta,Adam15,Adcy4,Pold3,Nfya,Csf1,Sox5,Lcp2,Wnt6,Hr,Erf,Dhh,Tob2,Angptl2,Nfkb2,Mxd4,Stc2,Rora,Grid2,Sstr5,Coro1c,Hyal3,Sla2,Pdlim2,Slc16a6,Il13,Rab8b,Mll2,Trex2,Tpm3,Ctsg,Lair1,Mr1,Lgals4,Tyrobp,Psmd9,Creb3,Tnfsf11,Evx1,Ptprcap,Cxcr3,Muc13,Ckb,Ptpn6,Hoxc6,Sec14l2,Pthlh,Ncam1,Matn3
CEBP_01	40	1,82	7,81E-14	Enam,Rgs3,Hoxa11,Cacng2,Cacnb1,Tnf,Tacstd2,Sox5,Col2a1,Hoxb6,Angptl2,Gsta4,Vax1,Kcnj1,Cdh16,Rora,Lbx1,Npas2,Sstr5,Plek,Bcl11b,Il13,Mll2,S100a9,Mtss1,Nfatc4,Adm,Lrrtm1,Bcar3,Sema3a,Slc35a2,Hs3st3b1,Caln1,Sntb2,Dnm3,Supt16h,Cdh13,Atoh1,Tnmd,Dll1
GATA_C	54	2,46	1,35E-13	Trim10,Phox2a,Krt15,Lcat,Esrrg,Msr1,Blvrb,Klhdc3,Tnfrsf19,Tm4sf5,Pla2g1b,Prdx2,Nr5a2,Nfib,Mid1,Bspry,Ctse,Il7,Spink4,Sox5,Nfe2,Slc4a1,Vps18,Hoxa7,Stc1,Slco1c1,Phospho1,Hoxb4,Adcyap1,Aqp2,Tbx5,Hyal3,Ptf1a,Amhr2,Polr3d,Mst1,Myh7,Tpm3,Cited4,Hoxd1,Fmo4,Trim15,Slc35a2,Pklr,Klf1,Myog,Supt16h,Reg4,Otc,Map4k5,Hs6st3,Hoxc6,Plac1,Sox10
S8_01	39	1,77	2,22E-13	Grpr,Htr2c,B3galt1,Irx4,Six3,Cldn8,Prop1,Esr2,Sox5,Hrk,Prss12,Wnt8b,Hoxa7,Stc1,Bmp1,Grwd1,Hoxb6,Otx2,Lbx1,Cntnap4,Lamb1,Kcns1,Dnajc5b,Sez6,Cxcl14,Eomes,Nfatc4,Hesx1,Sall3,Tle4,Lrrtm1,Scn3a,Sema3a,Ddx5,Ovol1,Prrx1,Dlx1,Hoxc6,Zfpm2
IK2_01	40	1,82	6,28E-13	Atoh7,Itm2b,Bcl2,Blnk,P2ry4,Hoxb5,Fpgs,Fgf17,Hcst,Bmf,Aspa,Wnt8b,Relb,Hoxb4,Slc26a6,Rora,Ascl2,Tbx19,Elf5,Cxcl14,Slitrk1,Actn1,Nfatc4,Col12a1,Epha2,Ap1g2,T,P2rx1,Slc35a2,Supt16h,Cxcr3,Elk4,Ovol1,Dlx1,Tead2,Cyp26a1,Odf1,Hs6st3,Hoxc6,Zfpm2
AML1_01	37	1,68	7,26E-13	Rag1,Entpd1,Adamts4,Cd6,Mmp14,Ifng,Mpl,Btg4,Tacstd2,Sox5,Slc15a3,Pdzk1,Bmf,Wnt8b,Diablo,Kcnj1,Mtx1,Coro1c,Anxa8,Tbx5,Ankrd1,Rgs1,Cyp17a1,Gpd1,Batf,Ly9,Tll2,S100a9,Mr1,Ap1g2,Bcar3,Dnm3,Supt16h,Hoxc6,Crtac1,Pde6h,Fgf4
AML1_Q6	37	1,68	7,26E-13	Rag1,Entpd1,Adamts4,Cd6,Mmp14,Ifng,Mpl,Btg4,Tacstd2,Sox5,Slc15a3,Pdzk1,Bmf,Wnt8b,Diablo,Kcnj1,Mtx1,Coro1c,Anxa8,Tbx5,Ankrd1,Rgs1,Cyp17a1,Gpd1,Batf,Ly9,Tll2,S100a9,Mr1,Ap1g2,Bcar3,Dnm3,Supt16h,Hoxc6,Crtac1,Pde6h,Fgf4
COREBINDINGFACTOR_Q6	39	1,77	1,21E-12	Rag1,Cd6,Ccl4,Runx1,Ccr1,Mpl,Btg4,Tacstd2,Sox5,Pdzk1,Diablo,Hoxb6,Myl3,Kcnj1,Hoxb4,Mtx1,Enpp1,Anxa8,Tbx5,Bmpr1b,Sla2,Rgs1,Nr4a3,Il13,Cyp17a1,Gpd1,Batf,Ly9,Pitpnc1,S100a9,Ptprs,Bcar3,Napsa,Sntb2,Supt16h,Drd3,Hoxc6,Crtac1,Lpo

AP4_Q6	35	1,59	1,36E-12	Tmod4,Traf4,Rxrg,Cbfa2t3,Dhrs3,Hoxb5,Runx1,Rasgrf2,Klf13,Nes,Gas7,Pcdh12,Lbx1,Dll3,Mybph,Anxa8,Prkcq,Pitx3,Bcl11b,Nppa,Tpm3,Col7a1,Wnt2b,Sorbs1,Ptprs,Epha2,Asb16,Epor,Loxl4,Eya3,Chrng,Ptch2,Ndst2,Pde3a,Mylk
NFY_Q6	36	1,64	2,44E-12	Myo1e,Enam,Barhl1,Htr2c,Shh,Cyp24a1,Nfya,Slc12a1,Wnt8b,Sfrp1,Hmx1,Gpr50,Top2a,Alb,Ddr2,Ptf1a,Pitx3,Lhx1,Acr,Cit,Dlx3,Tll2,Suv39h1,Nfatc4,Six4,Tle4,Tnfsf11,Klf1,Nkx6-2,Dlx1,Osbpl9,Imp4,Atoh1,Crabp2,Elac2,Entpd7
TCF4_Q5	36	1,64	3,44E-12	Hoxa11,Tnfrsf19,Fgf17,Slc22a8,Sox5,Wnt6,Hoxb6,Myl3,Vax1,Hoxb4,Cubn,Pcdh7,Coro1c,Itgb6,Mme,Elf5,Nppa,Nr4a3,Bmp7,Tll2,Nfatc4,Mycl1,Sorbs1,Six4,Ap1g2,Col18a1,T,Ogg1,Gab2,Tcf7l2,Prrx1,Dlx1,Sox14,Spo11,Map4k5,Hoxc6
HNF3B_01	34	1,55	5,67E-12	Hoxa11,Ier3,Lhx5,Anxa1,Il21,Tnf,Nfib,Lta,Lcp2,Foxn1,Hoxa7,Vax1,Rarg,Otx2,Rora,Lbx1,Gabra1,Mllt6,Slc16a6,Bcl11b,Gipc2,Hoxa4,Abt1,Klhl1,Nfatc4,Accn5,Hesx1,Tle4,Ap1g2,Pla1a,Drd3,Sox14,Zfpm2,Tnmd
ZIC3_01	37	1,68	7,67E-12	Hoxa11,Esrrg,Cuedc1,Ntn1,Dhrs3,Hspb7,Musk,Wnt10b,Mycn,Gnao1,Hoxa7,Cd160,Hpca,Angptl2,Loxl3,Tlk2,Gabra1,Kcnk4,Chst3,Klhl10,Cyyr1,Ptpn14,Calcb,Sema3b,Rel,Dock6,Nfam1,Col7a1,Nfatc4,Six4,P2rx1,Dll4,Tead3,Ptch2,Cyp26a1,Kcnd2,Nell2
GATA6_01	36	1,64	9,30E-12	Pdgfra,Rgs3,Krt15,Esrrg,Aqp3,Cox8c,Nr5a2,Mid1,Ctse,Il7,Spink4,Sox5,Nog,Iqgap1,Stc1,Sfrp1,Hoxb6,Vax1,Foxh1,Otx2,Adcyap1,Ascl2,Ddr2,Tbx5,Hoxa4,Mos,Sorbs1,Tle4,Esrrb,Atp6v0a4,Gabra2,Reg4,Nr2f2,Prrx1,Zfpm2,Plac1
CART1_01	33	1,50	1,10E-11	Pdgfra,Hoxa11,Esrrg,Sox4,Rab2b,Prdx2,Hoxb5,Mid1,Tacstd2,Sox5,Prss12,Nos1,Stc1,Hoxb6,Otx2,Epha7,Cntnap4,Ptf1a,Cyp46a1,Elf5,Pappa,Neto1,Hesx1,Sall3,Hoxa5,Slc26a3,Ogg1,Dll4,Ror1,Tead3,Prrx1,Zfpm2,Crtac1
HNF1_01	36	1,64	1,49E-11	Pdgfra,Cuedc1,Nr5a2,Btg4,Cldn19,Hrk,Col16a1,Satb2,Cdh16,Crb3,Npas2,Alb,Anxa13,Fxyd2,Afp,Ptbp1,Afm,Havcr1,Hoxa4,Chrd,Serpina6,Guca2b,Hesx1,Tle4,Sema3a,Pla1a,Slc26a3,Dlx1,Col9a1,Cyp26a1,Fga,Hoxc6,Plunc,Zfpm2,Slc3a1,Ckm
E2A_Q2	34	1,55	1,55E-11	Atoh7,Tmod4,Otos,Cbfa2t3,Dhrs3,Mid1,Nkx2-5,Runx1,Pou4f3,Musk,Irx5,Sftpc,Tlk2,Lbx1,Pitx3,Hn1,Ankrd1,Igf2,Eif4g2,Dlx2,Kcnip2,Ldb3,Nfatc4,Wnt2b,Scn5a,Epha2,Crat,Chrnd,Gab2,Evx1,Loxl4,Cyp26a1,Tnni2,Ckm
HMGIY_Q6	35	1,59	2,11E-11	Ecel1,Il1rn,Slc6a12,Vgll4,Nfkbia,Irx5,Doc2b,Pold3,B4galt5,Sox5,Il16,Gas7,Nos1,Hoxb6,Ndrg2,Klkb1,Il18rap,Hoxb4,Plxdc2,Grik1,Mrc2,Dll3,Fxyd2,Slitrk1,Klrc2,Batf,Nfatc4,Mgll,Il24,Map3k8,Tnfsf11,Aak1,Hoxc6,Pthlh,Ncam1
TATA_C	40	1,82	2,11E-11	Rasl11b,Barhl1,Esrrg,Pacsin3,Anxa1,Nr5a2,Ppap2a,Tbx3,Nkx2-5,Esr2,Sox5,Stc1,Gpr50,Hoxb6,Fbxo36,Cuedc2,Il1b,Hoxb4,Fgf23,Otx2,Aqp8,Bmpr1b,Pdha2,Bcl11b,Nppa,Slitrk1,Aldob,Nfatc2,Gprc5d,Sema3a,Pdgfrb,Ddx5,Aak1,Hnf4g,Atoh1,Rbp2,Crabp2,Hoxc6,Plunc,Mylk
OCT_C	50	2,27	2,51E-11	Enam,Kcnn3,Bcl2,Blnk,Irx4,Dtna,Cbfa2t3,Cacng2,Fgfr2,Mid1,Sema7a,Lhx3,Irx5,Sox5,Itsn1,Wnt6,Foxn1,Hoxa7,Nos1,Pou2f3,Sfrp1,Hoxb6,Hoxb4,Otx2,Grid2,Col25a1,Dll3,Pou1f1,Nr4a3,Lipg,Rel,Tll2,Sema6c,Klhl1,Sorbs1,Sgca,Sall3,Stat4,Adm,Hoxa5,Cnnm4,Gab2,Dll4,Tead3,Prrx1,Dlx1,Adora2a,Atoh1,Ascl3,Dll1

SOX9_B1	52	2,37	3,04E-11	Rgs3,Traf4,Sox4,Nxn,Fgfr2,Nfib,Mid1,Ngfr,Adam15,Sox5,Gnao1,Slc4a1,Bmf,Ndst3,Stc1,Pcdh12,S100a1,Sfrp1,Il17b,Ndrg2,Prm1,Top2a,Myl3,Vax1,Plxdc2,Mfap5,Prkcq,Mllt6,Hn1,Trim8,Cdkn1a,Bcl11b,Slitrk1,Tpm3,Sema6c,Mtss1,Mycl1,Arf4,Cd151,Loxl4,Fbn2,Fank1,Cer1,Dlx1,Sox14,Fen1,Ndst2,Tnni2,Hoxc6,Fgfr3,Ncam1,Mylk
GATA1_02	34	1,55	3,41E-11	Pdgfra,Esrrg,Tm4sf5,Pla2g1b,Prdx2,Sufu,Nr5a2,Cacna1f,Nfib,Irx5,Jph1,B4galt2,Plekhb1,Satb2,Slc4a1,Wnt16,Hoxb6,Pcdh7,Stc2,Rora,Grid2,Aqp2,Tbx5,Pdha2,Amhr2,Sema6a,Aldob,Il13,Slc8a3,Mos,Suv39h1,Wnt2b,Reg4,Otc
TCF1P_Q6	34	1,55	3,98E-11	Tuba8,Hoxa11,Klk8,Esrrg,Pacsin3,Prdx2,Ppap2a,Tbx3,Sema7a,Fxyd5,Lta,Jph1,Gpr87,Prkd2,Fzd1,Adcy8,Grik1,Epha7,Srgap2,Pola2,Cdkn1a,Bcl11b,Kif3c,Hoxa4,Mtss1,Col18a1,Sema3a,Art5,Atp6v0a4,Ccl20,Ddx5,Nppc,Hs6st3,Hoxc6
STAT5B_01	43	1,96	4,60E-11	Kcnn3,Fxyd1,Adamts4,Rgs3,Hoxa11,Gif,Ecel1,Hsf4,Gfap,Synpo,Nfkbia,Cldn8,Lta,Prol1,Ccl2,Pcolce,Lcp2,Slamf1,Stc1,Syt12,Vax1,Ipo11,Tlr7,Bach2,Homer2,Nr4a3,Batf,Smpd3,Nfatc4,Cited4,Ap1g2,Lama2,Trim15,Abcc5,Tnfsf11,Ogg1,Dll4,Sigirr,Iapp,Cyp26a1,Fga,Ascl3,Sdc1
SREBP_Q3	34	1,55	4,63E-11	Ndufs8,Fxyd1,Gfap,Tnfrsf19,Mid1,Adcy4,Col2a1,Gnao1,Dhh,Nfe2l3,Ager,Kcnj1,Hoxb4,Kcnmb1,Otx2,Grid2,Lbx1,Inpp4a,Baz2a,Mllt6,Lhx1,Amhr2,Mst1,Eif4g2,Sema3b,Guca2b,Nfatc4,Pdgfrb,Eya3,Myog,Tead2,Tnni2,Pthlh,Adra1b
CACCCBINDINGFACTOR_Q6	36	1,64	4,67E-11	Rgs6,Shh,Gfap,Slc12a5,Flnc,Hoxb5,Lin7b,Calcr,Hrk,Klf13,Gnao1,Hr,Slc25a13,Bmf,Abcg4,Diablo,Kcnd1,Nln,Rarg,Otx2,Lbx1,Epha7,Chrm1,Cdkn1a,Sema3b,Rgs14,Mtss1,Mycl1,Sorbs1,Six4,Magel2,Mip,Xpo1,Dlx1,Hoxc6,Sdc1
NF1_Q6_01	36	1,64	6,19E-11	Slc2a4,Sox4,Nfkbia,Cacnb1,Hoxb5,Mid1,Runx1,Prop1,Jph1,Klf13,Figf,Wnt16,Tlk2,Enpp1,Cntnap4,Stard3,Ntng2,Eif4g2,Gpd1,Trex2,Dlx3,Mtss1,Mycl1,Lrrtm1,Col11a2,Bcar3,Slco2a1,Aak1,Sox14,Rbp2,Map4k5,Syt8,Fgf4,Cyp1a2,Rasgrp2,Ddit4l
E47_01	35	1,59	6,67E-11	Coro2a,Arhgap8,Rxrg,Flnc,Otos,Cbfa2t3,Cacna2d2,Runx1,Kcnj4,Musk,Irx5,Wnt6,Hoxa7,Pou2f3,Erf,Stc2,Dusp9,Coro1c,Trim8,Bcl11b,Nr4a3,Kcnip2,Hrh3,Chrd,Mtss1,Wnt2b,Sorbs1,Scn5a,Epha2,Dll4,Cer1,Khdrbs2,Cyp26a1,Ckm,Ddit4l
PAX2_02	35	1,59	7,70E-11	Esrrg,Dtna,Flnc,Cacna2d2,Nfkbia,Dhrs3,Hoxb5,Nfib,Chodl,Nfya,Col10a1,Erf,Ager,Hoxb4,Park2,Pcdh7,Adcy8,Grid2,Lasp1,Tbx5,Stard3,Trim8,Egfr,Cldn18,Slc16a6,Crb1,Mgll,Phgdh,Lrrtm1,Xpo1,Tcf7l2,Zfpm2,Sox10,Sdc1,Dll1
POU6F1_01	34	1,55	8,19E-11	Hs3st1,Grpr,Hoxa11,Tmod4,Gnb1l,Gpr3,Dtna,Sox4,Ppp1r3a,Slc22a8,Calcr,Sox5,Hoxa7,Hoxb6,Gtpbp1,Hoxb4,Pcdh7,Otx2,Rora,Adcyap1,Myh8,Col25a1,Ptf1a,Ntng2,Gtf2ird1,Lipg,Hoxa5,Tnfsf13b,Myog,Dlx1,Sox14,Hoxc6,Zfpm2,Ncam1
AP1FJ_Q2	37	1,68	8,22E-11	Hoxa11,Gif,Rgs6,Blvrb,Cyp24a1,Dnm1,Synpo,Flnc,Il1rn,Cacng2,Pax9,Nrip3,Figf,Nln,Lamc1,Coro1c,Mybph,Baz2a,Tsnaxip1,Gabra1,Trim8,Hcfc1,Slc16a6,Dusp13,Stx17,Pitpnc1,Usp13,Pappa,Klhl1,Lamb3,Six4,Tle4,Epha2,Pdgfrb,Hs3st3b1,Gab2,Plac1
PTF1BETA_Q6	33	1,50	8,42E-11	Hoxa11,Cckbr,Traf4,Gpc6,Cd6,Bcl2,Sox4,Dhrs3,Klf13,Slc25a13,Stc1,Hoxb6,Relb,Vax1,Kcnj1,Hoxb4,Pcdh7,Syt3,Gabra1,Srgap2,Nppa,Slitrk1,Nr4a3,Slc8a3,Trex2,Lrrtm1,Xpo1,Pde10a,Ror1,Tnfrsf1a,Cdh13,Gnat1,Dlg4

MYOD_Q6_01	33	1,50	9,82E-11	Coro2a,Pacsin3,Lef1,Runx1,Pou4f3,Irx5,Actn3,Wnt6,Prss12,Bmp1,Sftpc,Sell,Mtx1,Lbx1,Pitx3,Hn1,Sez6,Igf2,Bmp7,Trex2,Tacr1,Kcnip2,Hrh3,Rel,Ldb3,Dock6,Nfatc4,Wnt2b,Mycl1,Epha2,Chrnd,Cyp26a1,Ckm
IRF_Q6	41	1,87	9,85E-11	Htr2c,Lsp1,Snx22,Usf1,Vgll4,Plp1,Pigr,Musk,Adam15,Sox5,Slc15a3,Satb2,Fcgr2b,Esr1,Prkd2,Aspa,Hoxb6,Sell,Hoxb4,Pcdh7,Ifnb1,Rarg,Zbp1,Tlk2,Ddr2,Ube2l6,Edil3,Tlr7,Zdhhc14,Eomes,Ifit2,Sorbs1,Sema3a,Pdgfrb,Tnfsf13b,Myog,Tcf15,Ncf1,Tcf7l2,Elk4,Dll1
NFY_01	33	1,50	1,13E-10	Myo1e,Barhl1,Rgs3,Cyp24a1,Lhx5,Nfya,Slc25a13,Wnt8b,Sfrp1,Gpr50,Zfp36l2,Kif23,Ddr2,Ptf1a,Lhx1,Acr,Cit,Dlx3,Serpina6,Nfatc4,Catsper2,Tle4,Pdgfrb,Klf1,Xpo1,Nkx6-2,Ptch2,Insm2,Gnat1,Dlx1,Elac2,Tacc3,Ncam1
CACBINDINGPROTEIN_Q6	32	1,46	1,64E-10	Slc2a4,Kcnn3,Adamts4,Dtna,Krt13,Cacng2,Klf13,Gnao1,Hr,Hoxb6,Fbxo36,Rfx1,Otx2,Lasp1,Mtx1,Mybph,Inpp4a,Chrm1,Clcn6,Acr,Acvr1b,Nr4a3,Kif3c,Procr,Dpf1,Six4,Supt16h,Prrx1,Dlx1,Hoxc6,Sdc1,Ncam1
HNF4_DR1_Q3	34	1,55	1,65E-10	Clcnka,Fxyd1,Lcat,Prkcd,Dusp3,Insr,Mpl,Sox5,Shmt1,Pdzk1,Hoxb6,Prrx2,Prodh2,Slc26a6,Tlk2,Erbb2,Mllt6,Sema3b,Nr4a3,Egr3,Hoxa5,Klk6,Pdlim7,F12,Pklr,F10,Ovol1,Mobp,Otc,Ndst2,Crabp2,Gata4,Fgfr3,Tnfrsf12a
SREBP1_01	51	2,32	1,99E-10	Hoxa11,Cyp27a1,Dnm1,Blnk,Cbfa2t3,Tlr4,Vgll4,Syngr1,Nr5a2,Dusp3,Hoxb5,Nfib,Mid1,Jph1,Sox5,Pdzk1,Vps18,Hoxa7,S100a1,Fbxo36,Hoxb4,Cdh16,Stc2,Adcy8,Ddr2,Atp6v1b2,Rph3a,Htr5a,Stard3,Rgs1,Actn1,Smoc2,Bmp7,Dlx2,Lipg,Rel,Ptgfr,Gab2,Xpo1,Ddr1,Supt16h,Cd164,C2,Dlx1,Sox14,Fen1,Slc35a5,Ucp3,Crabp2,Hoxc6,Sox10
HNF4_Q6	34	1,55	2,20E-10	Hoxa11,Lcat,Aqp3,Nr5a2,Sln,Dusp3,Insr,Mid1,Nfe2,Pdzk1,Bmf,C4b,Hpca,Gtpbp1,Cbx7,Mxd4,Tlk2,Col25a1,Lhx1,Gipc2,Sema3b,Gpd1,Glyat,Xpnpep2,Adm,Hoxa5,Trim15,Pklr,Evx1,Mmp16,Slpi,Mobp,Rab3d,Gata4
GATA4_Q3	33	1,50	2,38E-10	Polr1a,Esrrg,Gpc6,Tnfrsf19,Htr2b,Sox5,Hoxa7,Nos1,Stc1,Figf,Wnt16,Erf,Fgf23,Nfkb2,Otx2,Gpha2,Lbx1,Chrm1,Atp12a,Slc16a6,Rgs1,Actn1,Slc8a3,Egr3,Scn3a,Sema3a,Hira,Creb3,Evx1,Dll4,Tcf7l2,Nr2f2,Ovol1
HEB_Q6	34	1,55	2,50E-10	Tmod4,Traf4,Rxrg,Cbfa2t3,Dhrs3,Eln,Sema7a,Musk,Sox5,Klf13,Col16a1,Pdzk1,Itsn1,Wnt6,Gas7,Hpca,Angptl2,Psme3,Baz2a,Anxa8,Trim8,Ntng2,Klhl10,Sez6,Gpd1,Dock6,Wnt2b,Sorbs1,Catsper2,Kcnh3,Aak1,Ndst2,Ckm,Mylk
PAX4_02	33	1,50	3,72E-10	Htr2c,Hoxa11,Aqp3,Pitpnm2,Irx4,Sox4,Six3,Pou4f3,Tacstd2,Sox5,Klf13,Hoxb6,Gtpbp1,Zfp36l2,Otx2,Rora,Grid2,Tecta,Tbx5,Lamb1,Afp,Slc20a2,Ntng2,Sez6,Cacng8,Slitrk1,Eomes,Ptgfr,Sema3a,Ogg1,Dlx1,Sox14,Hoxc6
HNF4_01	34	1,55	3,84E-10	Traf4,Prkcd,Flnc,Dusp3,Insr,Sox5,Pdzk1,Hr,C4b,Prrx2,Cbx7,Slc26a6,Tlk2,Baz2a,Tbx5,Mllt6,Ntng2,F2,Sct,Bcl11b,Gipc2,Sema3b,Gpd1,Klhl1,Lgals4,Pdlim7,Slc26a3,Pklr,F10,Ovol1,Rbp2,Crabp2,Gata4,Rasgrp2
CEBP_Q3	33	1,50	4,87E-10	Asah2,Htr2c,Mid1,Irx5,Wnt10b,Gnao1,Slc12a1,Myl3,Gsta4,Ager,Rora,Myh8,Erbb2,Alb,Ascl2,Tbx5,Aqp9,Bcl11b,Lipg,Suv39h1,Nfatc4,Gys1,Cited4,Hoxa5,Ruvbl2,Pdgfrb,Per3,Nr2f2,Atoh1,Hoxc6,Plac1,Ncam1,Ddit4l
T3R_Q6	33	1,50	4,87E-10	Barhl1,Kcnn3,Shh,Lhx5,Synpo,Cacna2d2,Syngr1,Tbx3,Fxyd5,Fgf17,Sox5,Nog,Sf3b5,Vax1,Rarg,Epha7,Npas2,Baz2a,Tbx5,Mllt6,Sct,Jph2,Slitrk1,Slc8a3,Sema6c,Ccl20,Gnat1,Dlx1,Drd3,Tnfrsf12a,Atp1a2,Fgf4,Ddit4l
SMAD3_Q6	32	1,46	6,22E-10	Rasl11b,Esrrg,Synpo,Cbfa2t3,Dusp3,Irx5,Wnt10b,Chdh,Wnt8b,Fzd1,Ager,Fgf23,Otx2,Mrc2,Baz2a,Chrm1,Pitx3,Atp6v1e2,Cdkn1a,Pdlim2,Dlx3,Mycl1,Crat,Col11a2,Cpne6,Actn2,Tead3,Nr2f2,Sox14,Ptpn6,Crabp2,Hoxc6
LEF1_Q6	35	1,59	6,53E-10	Thsd1,Rgs3,Pacsin3,Tnfrsf19,Fgf17,Nfya,Slc22a8,Sox5,Wnt6,Hr,Dpysl4,Kif5a,Hoxb4,Otx2,Cdk5r1,Coro1c,Mllt6,Sez6,Elf5,Acvr1b,Bmp7,Nfatc4,Mycl1,Sorbs1,Six4,Col18a1,Bcar3,T,Eya3,Tcf7l2,Prrx1,Sox14,Kcnd2,Sdc1,Smarcc1

PAX_Q6	34	1,55	6,59E-10	Rgs3,Upk2,Havcr2,Blvrb,Ier3,Flnc,Irx3,Runx1,Musk,B4galt5,Chml,Actn3,Sftpc,Dhh,Fbxo36,Gtpbp1,Cdh16,Pdha2,Batf,Kcnip2,Tpm3,Egr3,Hoxa5,Sema3a,Chrnd,T,Hira,Ptch2,Prrx1,Sox14,Hnf4g,Osbpl9,Ptpn6,Plac1
ZIC2_01	32	1,46	6,93E-10	Barhl1,Dhrs3,Cd19,Musk,Nfya,Mycn,Nog,Gnao1,Hr,Hpca,Tob2,Hoxb4,Rarg,Kcna1,Plxdc2,Tlk2,Baz2a,Gabra1,Mllt6,Pdlim2,Sema3b,Rel,Sema6c,Col7a1,Lrrtm1,Ogg1,Dll4,Ptch2,Ckb,Cyp26a1,Kcnd2,Nell2
HNF6_Q6	32	1,46	6,93E-10	Pdgfra,Hoxa11,Sox4,Pax9,Tnf,Runx1,Lta,Ccl2,Gnao1,Pdzk1,Slc25a13,Tob2,Nln,Cbx3,Cdh16,Otx2,Lbx1,Col25a1,Tecta,Ankrd1,Pitx1,Bcl11b,Slitrk1,Dlx2,Klhl1,Tle4,Sema3a,Ogg1,Caln1,Cldn14,Hnf4g,Hoxc6
DBP_Q6	32	1,46	1,25E-09	Grpr,Barhl1,Pacsin3,Gpc6,Sox4,Pla2g1b,Mid1,Itih1,Calcr,Sftpc,Rarg,Otx2,Grik1,Epha7,Sox21,Sez6,Sema6a,Nr4a3,Mll2,Stx17,Mycl1,Tle4,Slc35a2,Cd151,Drd3,Bpil1,Sox14,Otc,Map4k5,Matn4,Tnmd,Mylk
LMO2COM_01	32	1,46	1,25E-09	Myo1e,Barhl1,Kcnn3,Hoxa11,Pax9,Runx1,Nfe2,Wnt6,Slc4a1,Commd5,Rarg,Otx2,Adcy8,Gjb4,Mllt6,Trim8,Sema6a,Igf2,Smpd3,Kcnip2,Mycl1,Col12a1,Epha2,Wnt3a,Chrnd,Gab2,Evx1,Sox14,Cyp26a1,Crabp2,Gng13,Ckm
ZID_01	30	1,36	1,51E-09	Myo10,Esrrg,Apex2,Cacng2,Nt5c1b,Plp1,Ngfr,Nfya,B4galt2,Actn3,Wnt6,Hr,Erf,Phospho1,Cdk5r1,Lbx1,Enpp1,Ddr2,Pappa,Sema6c,Chrd,Bcar3,Hira,Creb3,Supt16h,Ddx5,Htr1b,Map4k5,Nell2,Ddit4l
P53_DECAMER_Q2	33	1,50	1,66E-09	Lrdd,Adamts4,Hoxa11,Krt15,Blvrb,Anxa1,Cacng2,Trim29,Bmf,Stc1,Vax1,Nkx2-3,Tecta,Gjb4,Baz2a,Tbx5,Mllt6,Slc1a3,Cdkn1a,Egfr,Cldn18,Bcl11b,Slitrk1,Pitpnc1,Dlx3,Ldb3,Rhobtb2,Cited4,Tle4,Ap1g2,Il24,Abcc5,Prrx1
TCF11MAFG_01	44	2,00	1,70E-09	Rag1,Tbp,Itm2b,Angptl4,Blvrb,Irx4,Dtna,Flnc,Krt13,Hspb3,Cacnb1,Lta,Sox5,Esr1,Iqgap1,Kcnd1,Myl3,Ifnb1,Rarg,Ipo11,Kcna6,Limk1,Mrc2,Lamc1,Baz2a,Pdha2,Slc16a6,Aldob,Nr4a3,Gpd1,Dusp13,Slc8a3,Tpm3,Arf4,Six6,Abcc6,Cpne6,Gab2,Kcnh3,Loxl4,Sntb2,Aak1,Crygn,Cst7
COUP_01	32	1,46	1,86E-09	Esrrg,Traf4,Prkcd,Tpcn1,Asgr2,Dhrs3,Dusp3,Bmf,C4b,Prrx2,Gjb2,Pcdh7,Mybph,Baz2a,Tbx5,Mllt6,Nr4a3,Pitpnc1,Klhl1,Lgals4,Serpinc1,Slc26a3,Pklr,Ltb,Nr2f2,Ovol1,Ndst2,Rbp2,Crabp2,Gata4,Fgfr3,Tnfrsf12a
ARP1_01	25	1,14	1,93E-09	Esrrg,Traf4,Gpr3,Slc12a5,Nr5a2,Il21,Arr3,Prop1,Jph1,Nfe2,Col16a1,Hpca,Otx2,Cdk5r1,Asb12,Mllt6,Dnajc5b,Slitrk1,Pitpnc1,Sorbs1,Pmf1,Serpinc1,Htr1b,Dlx1,Ndst2
STAT3_02	24	1,09	2,15E-09	Kcnn3,Arhgap8,Rgs3,Upk2,Heyl,Irx5,Lta,Hoxb4,Epha7,Chrm1,Nfam1,Egr3,Kcnh3,Fbn2,Tcf7l2,Slc35a5,Ndst2,Hs6st3,Hoxc6,Matn4,Sdc1,Nell2,Crtac1,Ncam1
GR_Q6_01	32	1,46	2,36E-09	Pacsin3,Mmp14,Cuedc1,Hoxb5,Nog,Wnt8b,Loxl3,Otx2,Rora,Cdc42ep4,Lasp1,Lbx1,Epha7,Tecta,Elf5,Gipc2,Eif4g2,Dlx2,Mll2,Slc25a10,Dlx3,Gpr133,Phf3,Fank1,Cer1,Sf3b4,Tead2,Fen1,Mycbp,Ndst2,Sdc1,Tnmd
AFP1_Q6	32	1,46	2,36E-09	Pdgfra,Grpr,Barhl1,Kcnn3,Htr2c,Ecel1,Pacsin3,Dscaml1,Irx4,Dtna,Sox4,Map2k7,Btg4,Dnajb8,Nog,Satb2,Esr1,Stc1,Epha7,Lamc1,Tecta,Egfr,Acvr1b,Dlx2,Hoxa4,Pmf1,Dll4,Cdh13,Prrx1,Dlx1,Hoxc6,Zfpm2
ER_Q6_02	33	1,50	2,40E-09	Hck,Gpr3,Gfap,Fgfr2,Dusp3,Mid1,Map2k7,Slc22a6,B4galt2,Ndrg2,Kif5a,Rfx1,Grid2,Epha7,Tbx5,Pitx1,Kcnk4,Cdkn1a,Sct,Jph2,Slc16a6,Slc8a3,Hoxa5,Capn12,Creb3,Eya3,Tcf7l2,Cyp26a1,Csrp3,Crabp2,Gata4,Fgfr3,Tnfrsf12a
POU1F1_Q6	30	1,36	2,57E-09	Esrrg,Aqp3,Mrpl17,Sox4,Cacng2,Vgll4,Il21,Hoxb5,Runx1,Calcr,Syt12,Gtpbp1,Otx2,Rora,Dusp9,Adcyap1,Cntnap4,Cyp46a1,Slitrk1,Prl,Hesx1,Sall3,Hoxa5,Bcar3,Pde2a,Dlx1,Sox14,Hoxc6,Pthlh,Tnfrsf12a
ZIC1_01	32	1,46	2,67E-09	Traf4,Lef1,Cuedc1,Ntn1,Nfya,Wnt10b,Hcst,Klf13,Gnao1,Slc4a1,Wnt8b,Hoxa7,Hpca,Kcnd1,Kcna1,Rora,Plxdc2,Tlk2,Sema3b,Kif3c,Rgs14,Col7a1,Pacsin1,P2rx1,Atp6v0a4,Supt16h,Dll4,Ptch2,Ovol1,Cyp26a1,Kcnd2,Nell2

ISRE_01	30	1,36	3,42E-09	Kcnn3,Htr2c,Lsp1,Angptl4,Blnk,Usf1,Cacna2d2,Pigr,Adam15,Homer1,Esr1,Prkd2,Aspa,Wnt8b,Pcdh7,Ifnb1,Zbp1,Erbb2,Pitx3,Tlr7,Met,Ifit2,Sorbs1,Col12a1,Aif1,Cd151,Tnfsf13b,Tcf15,Tcf7l2,Ovol1
ER_Q6	33	1,50	3,90E-09	Barhl1,Tuba8,Esrrg,Gpr3,Gfap,Nfkbia,Dusp3,Mid1,Map2k7,Col10a1,Wnt8b,Ndrg2,Loxl3,Cdh16,Adcyap1,Npas2,Jph2,Slc16a6,Trex2,Tpm3,Hoxa5,Gad2,Esrrb,Atp6v0a4,Loxl4,Pglyrp2,Tcf7l2,Nr2f2,Wnt8a,Ovol1,Atoh1,Plac1,Ckm
BRN2_01	31	1,41	3,93E-09	Esrrg,Rgs6,Shh,Gpr3,Irx4,Fgfr2,Nt5c1b,Nog,Gnao1,Ndst3,Diablo,Il18rap,Vax1,Adcyap1,Npas2,Slitrk1,Smoc2,Nr4a3,Rab8b,Nfatc4,Mgll,Col12a1,Sall3,Phf3,Sntb2,Mmp16,Ptprcap,Aak1,Dlx1,Pthlh,Fgfr3
MYC_Q2	74	3,37	3,97E-09	Barhl1,Hoxa11,Klhdc3,Lef1,Slc12a5,Dtna,Dyrk1b,Brdt,Lmx1a,Nrip3,Hoxb5,Sema7a,Fpgs,Jph1,B4galt2,Esr2,Adcy3,Col2a1,Shmt1,Gadd45b,Satb2,Diablo,Hoxa7,Mxd3,Rab3il1,Hpca,Erf,Relb,Slco1c1,Nln,Gtpbp1,Hoxb4,Psme3,Rarg,Mxd4,Stc2,Nkx2-3,Syt3,Cdk5r1,Gnl1,Col25a1,Fxyd2,Chrm1,Pitx3,Polr3d,Bcl11b,Gata5,Nr4a3,Bmp7,Dlx2,Gpd1,Polh,Hoxa4,Hrh3,Mycl1,Tle4,Camk4,Hira,Nthl1,Gja1,Xpo1,Sntb2,Supt16h,Ddx5,Cd164,Elk1,Dlx1,Fen1,Slc35a5,Oprd1,Sdc1,Gata4,Acy1,Rasgrp2
LHX3_01	36	1,64	4,12E-09	Grpr,Barhl1,Kcnn3,Pacsin3,Ier3,Irx4,Lmx1a,Mid1,Irx5,Nfya,Hrk,Gnao1,Wnt8b,Gtpbp1,Otx2,Rora,Lbx1,Tecta,Kcns1,Tbx19,Sema6a,Prl,Pitpnc1,Mtss1,Hesx1,Col12a1,Adamts10,Sall3,Tle4,Lrrtm1,Sema3a,Tnfsf13b,Caln1,Dll4,Prrx1,Hoxc6
SRY_02	31	1,41	4,29E-09	Hs3st1,Esrrg,Dtna,Tnfrsf19,Krt13,Fgfr2,Dhrs3,Hoxb5,Nfib,Tacstd2,Sox5,Gnao1,Stc1,Chd1,Mrc2,Epha7,Tecta,Tbx5,Cldn18,Eif4g2,Nr4a3,Sema6c,Hesx1,Sall3,Six4,Ap1g2,Fbn2,Tcf15,Mobp,Dll1,Mylk
MEF2_03	31	1,41	4,29E-09	Slc2a4,Hoxa11,Slc6a13,Dyrk1b,Ppp1r3a,Hspb3,Nfib,Sema7a,Musk,Mgst3,Sox5,Esr1,Slamf1,Kcnj9,Ndrg2,Slc26a6,Lbx1,Epha7,Trhr,Slc8a3,Extl1,Kcnn1,Art5,Asb16,Aak1,Cldn14,Elk4,Prrx1,Wfdc1,Atp1a2,Mylk
IK1_01	33	1,50	4,29E-09	Itm2b,Prop1,Fgf17,Gadd45b,Relb,Hoxb4,Ifnb1,Nfkb2,Slc26a6,Rora,Tbx19,Sema6a,Elf5,Cxcl14,Slitrk1,Actn1,Nr4a3,Col12a1,T,P2rx1,Ccl20,Abcc5,Pde10a,Ddx5,Dll4,Mmp16,Elk4,Dlx1,Cyp26a1,Hoxc6,Zfpm2,Ascl3,Crtac1
VDR_Q3	30	1,36	4,31E-09	Slc2a4,Arhgap8,Cyp24a1,Mmp14,Slc12a5,Krt13,Fgfr2,Pou4f3,Hrk,Bmf,Stc1,Dhh,Fbxo36,Kcnd1,Pcdh7,Rarg,Grid2,Plxdc2,Chrm1,Bcl11b,Cxcl14,Eif4g2,Nr4a3,Dlx3,Kcnip2,Xpo1,Tcf15,Sox10,Atp1a2,Ncam1
TAL1BETAE47_01	30	1,36	4,31E-09	Rasl11b,Atoh7,Htr2c,Hoxa11,Upk2,Hck,Lef1,Cbfa2t3,Mark1,Sox5,Hrk,Wnt6,Hoxa7,Pcdh12,Hoxb6,Park2,Grid2,Cdc42ep4,Npas2,Gpd1,Kcnip2,Hrh3,Scn5a,Six6,Pmf1,Cdh23,Eya3,Dll4,Haao,Zfpm2
FOXJ2_01	27	1,23	4,75E-09	Hoxa11,Esrrg,Rgs6,Ier3,Dtna,Slc35c2,Ntn1,Sln,Tnf,Nfib,Cldn8,Lta,Lcp2,Gnao1,Foxn1,Hoxa7,Otx2,Rora,Ptf1a,Bcl11b,Mgll,Hesx1,Tle4,Drd3,Fen1,Hoxc6,Ncam1
NKX25_01	22	1,00	5,02E-09	Rasl11b,Phox2a,Bcl2,Cuedc1,Lmx1a,Jph1,Pdzk1,Wnt8b,Hoxb4,Stc2,Egfr,Bcl11b,Ppp3cc,Rgs1,Hoxd1,Lrrtm1,T,Ddr1,Dlx1,Ucp3,Crabp2,Crtac1
TBP_01	31	1,41	5,55E-09	Slc2a4,Sh3bgrl3,Fxyd1,Esrrg,Pacsin3,Lhx5,Slc35c2,Ppp1r3a,Hspb3,Asb18,Hoxb5,Sox5,Nog,Col10a1,Ndrg2,Nr0b1,Hoxb4,Ifnb1,Myh8,Lbx1,Epha7,Ptf1a,Fgf21,Gpr133,Dkk4,Art5,Abcc6,Asb16,Ckb,Hoxc6,Slc3a1
NKX61_01	30	1,36	6,34E-09	Pdgfra,Sh3bgrl3,Kcnn3,Myo10,Hoxa11,Esrrg,Irx4,Dtna,Sox4,Fgfr2,Mid1,Sox5,Hrk,Satb2,Wnt8b,Hoxa7,Tbx5,Trim8,Ntng2,Nr4a3,Kcnip2,Hesx1,Sall3,Tle4,Lrrtm1,Col11a2,Gad2,Prrx1,Hoxc6,Zfpm2

FOXO1_02	30	1,36	7,26E-09	Hoxa11,Irx4,Dtna,Ntn1,Dhrs3,Nfib,Gnao1,Satb2,Hr,Ccrl1,Mbtps1,Rfx1,Hoxb4,Il6st,Kcna1,Rora,Slu7,Mtx1,Mllt6,Trim8,Cdkn1a,Pitpnc1,Wnt2b,Sall3,Ddx5,Tcf15,Ror1,Mobp,Map4k5,Dll1
GFI1_01	46	2,09	7,83E-09	Grpr,Esrrg,Msr1,Traf4,Rxrg,Dtna,Sox4,Six3,Il21,Ppap2a,Irx3,Hoxb5,Mid1,Cldn8,Map2k7,Rasgrf2,Sox5,Il16,Itsn1,Esr1,Hoxa7,Stc1,Hoxb6,Adcy8,Rora,Ddr2,Anxa2,Gabra1,Chrm1,Polh,Crb1,Procr,Gprc5d,Klhl1,Six6,Hoxa5,Slco2a1,Slc26a3,Caln1,Cdh13,Nr2f2,Zfpm2,Pthlh,Ascl3,Ncam1,Agr2
PR_Q2	31	1,41	8,13E-09	Pdgfra,Atoh7,Rgs3,Shh,Irx4,Dtna,Pon2,Ahsg,Cacna2d2,Jph1,Adcy3,Sox5,Wnt8b,Kcnj1,Cdc42ep4,Lbx1,Gabra1,Pdha2,Cxcl14,Gipc2,Nr4a3,Dlx2,Kcnip1,Six4,Hoxa5,Epha2,Sema3a,Prrx1,Dlx1,Map4k5,Tnmd
LBP1_Q6	28	1,27	9,10E-09	Sh3bgrl3,Kcnn3,Tmod4,Pacsin3,Rxrg,Tpcn1,Usf1,Tbx3,Wnt10b,Sox5,Actn3,Barx2,Vamp1,Hpca,Mybph,Baz2a,Anxa8,Pitx3,Trim8,Eif4g2,Crb1,Kcnip2,Ldb3,Gab2,Loxl4,Supt16h,Mycbp,Tnni2
GATA3_01	30	1,36	9,25E-09	Trim10,Pla2g1b,Nr5a2,Cacna1f,Rad9b,Irx5,B4galt2,Sox5,Satb2,Esr1,Slc4a1,Slc25a13,Fzd1,Il17b,Hoxb6,Rora,Grid2,Erbb2,Aqp2,Tbx5,Pdha2,Amhr2,Il13,Slc8a3,Dlx3,Sorbs1,Trim15,Prrx1,Drd3,Sox10
HNF4_01_B	30	1,36	9,25E-09	Clcnka,Fxyd1,Asah2,Traf4,Prkcd,Dusp3,Insr,Pdzk1,Hr,Prrx2,Prodh2,Slc26a6,Cdk5r1,Tlk2,Erbb2,Baz2a,Lhx1,Sos1,Lgals4,Serpinc1,Hira,Evx1,Bcl2l14,Nr2f2,F10,Drd3,Otc,Rbp2,Crabp2,Gata4
GR_Q6	31	1,41	1,03E-08	Rgs3,Hoxa11,Gnb1l,Crhr2,Prdx2,Mid1,Fgf17,B4galt5,Calcr,Jph1,Nog,Esr1,Vax1,Gpha2,Lamb1,Chrm1,Cdkn1a,Jph2,Cxcl14,Gipc2,Dlx3,Gys1,Ruvbl2,Pde2a,Prrx1,Dlx1,Ndst2,Kcnd2,Sdc1,Tnmd,Ncam1
HNF1_C	29	1,32	1,05E-08	Lhx5,Slc1a1,Slc22a8,Aspa,Stc1,Proc,Kcnj1,Cdh16,Rora,Anxa13,Afp,Afm,Aqp9,F2,Havcr1,Guca2b,Sorbs1,Hesx1,Sall3,Tle4,Slc26a3,Pklr,Supt16h,Dlx1,Ndst2,Fga,Hoxc6,Zfpm2,Ncam1
CDC5_01	32	1,46	1,10E-08	Hoxa11,Esrrg,Slc6a13,Shh,Il21,Musk,Hrk,Col16a1,Gpr87,Rora,Nkx2-3,Cldn1,Dnajc5b,Bcl11b,Bach2,Rgs1,Mos,Usp13,Mgll,Hesx1,Sall3,Chrna5,Slco2a1,Ccl20,Supt16h,Dll4,Prrx1,Odf1,Atoh1,Hoxc6,Zfpm2,Sdc1
CRX_Q4	32	1,46	1,10E-08	Entpd1,Esrrg,Slc12a5,Flnc,Six3,Btg4,Irx5,Hrk,Satb2,Wnt6,Hr,Nos1,Angptl2,Zfp36l2,Kcna1,Rora,Grid2,Pitx1,Amhr2,Slitrk1,Kcnip2,Bdkrb1,Tle4,Wnt3a,Atp6v0a4,Abcc6,Tnfsf11,Mip,Tcf7l2,Prrx1,Dlg4,Hoxc6
OCT1_Q6	31	1,41	1,14E-08	Kcnn3,Rgs3,Gnb1l,Esrrg,Bcl2,Blnk,Irx4,Dtna,Irx3,Mid1,Sox5,Satb2,Hoxa7,Nos1,Pou2f3,Col25a1,Anxa2,Chrm1,Ankrd1,Rel,Tll2,Sema6c,Col12a1,Stat4,Scn3a,Col11a2,Gab2,Prrx1,Dlx1,Adora2a,Ascl3
RREB1_01	28	1,27	1,33E-08	Barhl1,Kcnn3,Slc12a5,Flnc,Cacng2,Runx1,Gnao1,Dhh,Ndrg2,Fbxo36,Myl3,Hoxb4,Kcna1,Otx2,Mtx1,Lbx1,Jph2,Rad23a,Myh7,Nfatc4,Egr3,Capn12,Cpne6,Supt16h,Dll4,Ovol1,Dlx1,Tead2
IK3_01	28	1,27	1,33E-08	Flnc,Lmx1a,Nfkbia,Nnmt,Adam15,Fgf17,Dnajb8,Hcst,Gnao1,Ptges,Hoxb4,Slc26a6,Stard3,Slitrk1,Actn1,Cyp17a1,Mtss1,Egr3,Ap1g2,Sema3a,T,Ccl20,Spinlw1,Xpo1,C2,Prrx1,Tnni2,Ascl3
AP1_Q4	32	1,46	1,38E-08	Hoxa11,Gpr3,Dnm1,Synpo,Il1rn,Pax9,Nrip3,Hspb7,Limk1,Lamc1,Coro1c,Mybph,Baz2a,Trim8,Cdkn1a,Slc16a6,Dusp13,Stx17,Pitpnc1,Usp13,Pappa,Lamb3,Epha2,Esrrb,Pdgfrb,Hs3st3b1,Cd151,Gab2,Adora2a,Plac1,Tnfrsf12a,Rpl27a
GATA1_04	30	1,36	1,50E-08	Pdgfra,Msr1,Synpo,Tnfrsf19,Prdx2,Nr5a2,Nfe2,Wnt6,Slc4a1,Ndrg2,Pcdh7,Rora,Grid2,Aqp2,Tbx5,Amhr2,Mst1,Kif3c,Slc8a3,Pappa,Rhobtb2,Hoxd1,Adm,Slc35a2,Esrrb,Klf1,Cldn14,Drd3,Hnf4g,Map4k5
HNF1_Q6	30	1,36	1,70E-08	Pdgfra,Ppp1r3a,Runx1,Rasgrf2,Nog,Satb2,Aspa,Cdh16,Crb3,Prodh2,Rora,Npas2,Alb,Anxa13,Fxyd2,Afp,Afm,Aqp9,F2,Serpina6,Guca2b,Sall3,Tle4,Napsa,Sema3a,Dlx1,Fga,Hoxc6,Plunc,Zfpm2

PIT1_Q6	29	1,32	1,71E-08	Entpd1,Htr2c,Esrrg,Sox4,Cacng2,Hoxb5,Mid1,Sema7a,Musk,Nog,Gnao1,Wnt8b,Hoxb6,Mxd4,Otx2,Rora,Epha7,Cntnap4,Slitrk1,Rgs1,Prl,Hoxa4,Scn3a,Ap1g2,Sema3a,Gab2,Ptch2,Hoxc6,Fgfr3
STAT1_03	29	1,32	1,71E-08	Hsd17b4,Arhgap9,Traf4,Sox4,Gadd45b,Col16a1,Slamf1,Gng4,Stc1,Erf,Dhh,Hoxb6,Vax1,Cbx3,Grik1,Tlk2,Tbx5,Gabra1,Chrm1,Afp,Mrpl34,Rab8b,Smpd3,Map3k8,Ddr1,Dlx1,Odf1,Sec14l2,Dll1
HSF2_01	29	1,32	1,96E-08	Pacsin3,Flnc,Cbfa2t3,Cacna2d2,Mid1,Pou4f3,Col16a1,Stc1,Pcdh7,Otx2,Grik1,Cdk5r1,Tbx5,Trim8,Gipc2,Eif4g2,Nr4a3,Trex2,Nfam1,Nfatc4,Egr3,Six4,Abcc5,Actn2,Mobp,Spo11,Hs6st3,Oprd1,Rasgrp2
HFH1_01	30	1,36	2,16E-08	Hoxa11,Slc12a5,Dtna,Ntn1,Nfib,Cldn8,Tacstd2,Lcp2,Satb2,Hr,Aspa,Chd1,Vax1,Hoxb4,Pcdh7,Otx2,Slu7,Lamc1,Trim8,Dnajc5b,Slc16a6,Bcl11b,Hesx1,Sall3,Ap1g2,Htr1b,Sox14,Atoh1,Hoxc6,Ncam1
HNF4ALPHA_Q6	31	1,41	2,32E-08	Clcnka,Pdgfra,Fxyd1,Traf4,Dhrs3,Dusp3,Ccr1,Nog,Actn3,Pdzk1,Hoxb6,Prrx2,Pcdh7,Slc26a6,Cdk5r1,Col25a1,Lhx1,Sema6a,Nr4a3,Kcnip2,Xpnpep2,Pacsin1,F12,Abcc6,Supt16h,Nr2f2,Ndst2,Rab3d,Rbp2,Hoxc6,Gata4
CEBPB_01	31	1,41	2,62E-08	Rgs3,Sprr1b,Gpr3,C3,Cacnb1,Tnf,Wnt10b,Wnt6,Slc12a1,Bmf,Stc1,Phospho1,Il1b,Pde1b,Stc2,Rora,Epha7,Col25a1,Alb,Ascl2,Sftpd,Myh7,Mtss1,Nfatc4,Gys1,Hoxa5,Ruvbl2,Tnfsf13b,Bmx,Dlx1,Lpo
SP1_Q4_01	29	1,32	2,87E-08	Ace,Phox2a,Gpr3,Rab2b,Cdc37,Lhx3,Plekhb1,Klf13,Iqgap1,Gas7,Dhh,Map3k7,Fbxo36,Mxd4,Lasp1,Cdk5r1,Gnl1,Coro1c,Ascl2,Bcl11b,Hrh3,Wnt2b,Loxl4,Abhd1,Elk1,Ptch2,Tead2,Osbpl9,Kcnc3
NFE2_01	36	1,64	3,47E-08	Ids,Arhgap8,Myo10,Hoxa11,Esrrg,Itm2b,Angptl4,Synpo,Dtna,Syngr1,Dhrs3,Nrip3,Adam15,Figf,Sftpc,Il18rap,Pde1b,Ipo11,Limk1,Lamc1,Mybph,Baz2a,Cdkn1a,Slc16a6,Dusp13,Usp13,Procr,Epha2,Capn12,Hs3st3b1,Gab2,Cdh23,Eya3,Dlx1,Rab3d,Cst7
OSF2_Q6	30	1,36	3,52E-08	Traf4,Mmp14,Dyrk1b,Ifng,Hoxb5,Runx1,Col2a1,Il16,Wnt6,Stc1,Hoxb6,Kcnj1,Hoxb4,Stc2,Slc26a6,Coro1c,Tbx5,Sla2,Dlx2,Gpd1,Dusp13,Rgs14,Rel,Wnt7b,Hoxa5,Atp6v0a4,Supt16h,Prrx1,Ncam1,Fgf4
NKX62_Q2	29	1,32	3,67E-08	Pdgfra,Barhl1,Kcnn3,Hoxa11,Ier3,Irx4,Dtna,Six3,Sln,Otx2,Npas2,Tbx5,Ptf1a,Kcns1,Slitrk1,Hoxa4,Hesx1,Col11a2,Wnt3a,Ogg1,Xpo1,Myog,Bmx,Dlx1,Sox14,Atoh1,Pgr,Hoxc6,Zfpm2
AP2ALPHA_01	28	1,27	3,76E-08	Dscaml1,Mmp14,Nfib,Lta,Jph1,Sox5,Klf13,Hr,Gas7,Erf,Relb,Rarg,Mxd4,Tlk2,Gnl1,Map2k2,Drd2,Pdlim2,Sos1,Sema6c,Neto1,Scn5a,Loxl4,Ddr1,Dll4,Tead2,Sox10,Barx1
MMEF2_Q6	32	1,46	3,80E-08	Sh3bgrl3,Hoxa11,Pacsin3,Slc12a5,Lmx1a,Dhrs3,Six3,Hspb7,Htr2b,Calcr,Sox5,Nog,Esr1,Hoxb6,Grik1,Prkcq,Gabra1,Hoxa4,Tacr1,Usp13,Sall3,Lrrtm1,Col11a2,Sema3a,Slco2a1,Cldn14,Prrx1,Sox14,Wfdc1,Sdc1,Ckm,Hspb1
AP3_Q6	30	1,36	3,89E-08	Upk2,Msr1,Rgs6,Lhx5,Irx4,Dtna,Cacng2,Fgf17,Wnt8b,Hoxb6,Gsta4,Mxd4,Otx2,Adcy8,Rora,Trim8,Atp6v1e2,Tbx19,Sema6a,Cxcl14,Tacr1,Pappa,Ptgfr,Phf3,Evx1,Abhd1,Zfpm2,Ascl3,Ttll3,Mylk
NFY_C	28	1,27	4,22E-08	Rag1,Myo1e,Ndufs8,Esrrg,Shh,Cldn8,Nfya,Satb2,Slc12a1,Wnt8b,Sfrp1,Kif23,Mtx1,Ddr2,Ptf1a,Lhx1,Tradd,Acr,Blk,Nfatc4,Tle4,Ogg1,Klf1,Atoh1,Fbxl8,Elac2,Ncam1,Fgf4
AP1_Q6	30	1,36	4,87E-08	Ids,Hoxa11,Gpr3,Gfap,Dtna,Flnc,Il1rn,Dhrs3,Nrip3,Hspb7,Mark1,Wnt6,Sftpc,Ndrg2,Lamc1,Mybph,Baz2a,Trim8,Cdkn1a,Slc16a6,Stx17,Pitpnc1,Usp13,Pappa,Nfatc4,Epha2,Pdgfrb,Gab2,Adora2a,Rab3d
FOXD3_01	27	1,23	4,87E-08	Hoxa11,Ier3,Gpr3,Dtna,Ntn1,Cldn8,Runx1,Tacstd2,Gnao1,Proc,Otx2,Rora,Cntnap4,Lhx1,Atcay,Gtf2ird1,Bcl11b,Klhl1,Hesx1,Tle4,Lrrtm1,Evx1,Tcf7l2,Drd3,Atoh1,Hoxc6,Plunc

MEF2_01	22	1,00	4,89E-08	Pdgfra,Esrrg,Slc12a5,Spic,Wnt8b,Kcnj9,Il17b,Phospho1,Hoxb4,Grik1,Ascl2,Trim8,Itgb6,Jph2,Bmp7,Kcnn1,Dll4,Sox14,Wfdc1,Zfpm2,Ckm,Gata4
SMAD4_Q6	28	1,27	5,39E-08	Pdgfra,Adamts4,Rgs3,Slc12a5,Cbfa2t3,Dhrs3,Pou4f3,Diablo,Ager,Akap4,Loxl3,Nfkb2,Otx2,Rora,Col25a1,Tbx5,Chrm1,Trim8,Fmo2,Atp6v1e2,Nfatc4,Epha2,Evx1,Foxi1,Cdh13,Tnfrsf12a,Fgf4,Mylk
OCT1_03	27	1,23	5,46E-08	Pdgfra,Atoh7,Cacng2,Nfkbia,Il21,Ppap2a,Asb18,Hoxb5,Mid1,Dnajb8,Jph1,Sox5,Wnt8b,Grwd1,Hoxb4,Otx2,Bach2,Nr4a3,Dlx2,Hoxa4,Sema3a,Aak1,Dlx1,Drd3,Sox14,Tnmd,Mylk
PAX4_04	27	1,23	5,46E-08	Rgs3,Sox4,Cacng2,Nfib,Map2k7,Gnao1,Itsn1,Sf3b5,Wnt8b,Stc1,Erf,Nln,Prrx2,Hoxb4,Otx2,Adcyap1,Col25a1,Gabra1,Bach2,Slitrk1,Pappa,Sall3,Six4,Hoxa5,Supt16h,Elk1,Mylk
GATA1_03	29	1,32	5,74E-08	Gnb1l,Klhdc3,Mid1,Jph1,Sox5,Pcolce,Nfe2,Slc4a1,Wnt8b,Hoxa7,Pou2f3,Hoxb6,Ndrg2,Kcnd1,Pcdh7,Otx2,Lbx1,Tbx5,Tbx19,Elf5,Igf2,Pappa,Adm,Fmo4,Nkx6-2,Mmp16,Dlg4,Zfpm2,Gata4
CEBP_Q2_01	31	1,41	6,27E-08	Gabrr2,Asah2,Sprr1b,Cuedc1,Asgr2,Dbh,Cldn8,Wnt10b,Sox5,Wnt6,Esr1,Slc12a1,Stc1,Erf,Pcdh7,Stc2,Alb,Ascl2,Fmo2,Egfr,Lipg,Gys1,Cited4,Hoxa5,Ruvbl2,Tnfsf13b,Xdh,Bmx,Dlx1,Fga,Hoxc6
SRF_Q5_01	29	1,32	6,44E-08	Myo1e,Slc2a4,Flnc,Hoxb5,Mid1,Srd5a2,Fgf17,Aspa,Il17b,Prm1,Hoxb4,Pcdh7,Kcna1,Npas2,Coro1c,Itgb6,Slc16a6,Nppa,Actn1,Tpm3,Nfatc4,Egr3,Pdlim7,Caln1,Nr2f2,Ckm,Tnmd,Dll1,Mylk
FAC1_01	26	1,18	7,11E-08	Barhl1,Blnk,Dyrk1b,Maf1,Nfib,Runx1,Pou4f3,Sox5,Bmf,Gpr50,Hoxb6,Rfx1,Hoxb4,Otx2,Tbx5,Amelx,Srgap2,Trex2,Pappa,Sall3,Six4,Sema3a,Pdgfrb,Tcf7l2,Nr2f2,Map4k5
OCT1_Q5_01	30	1,36	7,58E-08	Kcnn3,Bcl2,Blnk,Irx4,Mid1,Itsn1,Wnt6,Hoxa7,Nos1,Pou2f3,Sfrp1,Hoxb6,Hoxb4,Otx2,Col25a1,Anxa2,Lipg,Rel,Tll2,Sema6c,Stat4,Hoxa5,Cnnm4,Pla1a,Gab2,Prrx1,Dlx1,Adora2a,Pthlh,Ascl3
AP1_Q2	31	1,41	8,71E-08	Hoxa11,Cyp24a1,Dnm1,Flnc,Il1rn,Sufu,Pax9,Nrip3,Hspb7,Figf,Nln,Lamc1,Coro1c,Mybph,Baz2a,Trim8,Slc16a6,Dusp13,Stx17,Pitpnc1,Usp13,Pappa,Klhl1,Lamb3,Six4,Epha2,Pdgfrb,Gab2,Adora2a,Map4k5,Plac1
E47_02	28	1,27	9,80E-08	Atoh7,Dscaml1,Gna12,Gfap,Irx4,Cuedc1,Hspb3,Ngfr,Doc2b,Wnt10b,Plekhb1,Bmf,Hoxa7,Stc2,Dusp9,Npas2,Baz2a,Anxa8,Prkcq,Mllt6,Chrm1,Klhl1,Scn5a,Foxi1,Mmp16,Bmx,Tead2,Drd3
CDPCR1_01	20	0,91	1,10E-07	Slc12a5,Tbx3,Nfib,Col2a1,Nog,Sftpc,Kcnd1,Vax1,Hoxb4,Kcna1,Rph3a,Ntng2,Kcns1,Tle4,Phf3,Cpne6,Tcf7l2,Insm2,Hoxc6,Gata4
ETS_Q4	28	1,27	1,10E-07	Irak4,Map4k1,Adamts4,Rgs3,Blnk,Cbfa2t3,Fgfr2,Fxyd5,Adcy4,Mark1,Hcst,Ccl2,Lcp2,Klf13,Gmfg,Pcdh7,Fcho1,Baz2a,Pdlim2,Il13,Rgs14,Scn3a,Ap1g2,Tnfsf11,Gab2,Sigirr,Tead3,Sox14
ZF5_01	28	1,27	1,10E-07	Phox2a,Dscaml1,Itpka,Ntn1,Ngfr,Sema7a,Irx5,B4galt2,Klf13,Gadd45b,Hpca,Syt12,Frat1,Tlk2,Lbx1,Coro1c,Mllt6,Ptbp1,Trim8,Sez6,Smpd3,Nfatc4,Adm,Hs3st3b1,Nr2f2,Gfer,Mycbp,Hs6st3
MEF2_Q6_01	29	1,32	1,29E-07	Pdgfra,Slc2a4,Slc6a13,Hspb3,Sox5,Nog,Esr1,Kcnj9,Slc26a6,Lbx1,Epha7,Inpp4a,Slitrk1,Trhr,Slc8a3,Usp13,Scn3a,Kcnn1,Kcna7,Art5,Myog,Dll4,Aak1,Cldn14,Iapp,Wfdc1,Zfpm2,Ckm,Gata4
RSRFC4_01	29	1,32	1,45E-07	Pdgfra,Rgs3,Arr3,Musk,Mgst3,Sox5,Itsn1,Esr1,Slamf1,Ndrg2,Tob2,Myl3,Hoxb4,Slc26a6,Adcyap1,Epha7,Inpp4a,Slc8a3,Extl1,Kcnn1,Art5,Asb16,Cldn14,Csrp3,Wfdc1,Tnni2,Zfpm2,Ckm,Hrasls
VDR_Q6	28	1,27	1,57E-07	Adamts4,Klk8,Esrrg,Itm2b,Cyp24a1,Pitpnm2,Irx4,Usf1,Pou4f3,Fgf17,Calcr,Trim29,Sox5,Hpca,Hoxb6,Fbxo36,Vax1,Ntng2,Polr3d,Mst1,Sema3b,Slc25a10,Six4,Hoxa5,Col11a2,Xpo1,Ovol1,Cyp26a1

RP58_01	27	1,23	1,89E-07	Atoh7,Ntn1,Cbfa2t3,Hspb7,Runx1,Pcolce,Klf13,Col16a1,Slc4a1,Gas7,Myl3,Park2,Rarg,Lbx1,Epha7,Pitx3,Dnajc5b,Tlr7,Col7a1,Wnt2b,Sorbs1,Adamts10,Pmf1,Pde10a,Cmklr1,Ptch2,Tnni2
MZF1_01	27	1,23	2,41E-07	Entpd1,Kcnn3,Blvrb,Klhdc3,Krt13,Syngr1,Nrip3,Hr,Ndrg2,Hoxb4,Otx2,Mtx1,Itgb6,Sema6a,Bcl11b,Cxcl14,Eif4g2,Kif3c,Zdhhc14,Hoxa4,Mgll,Six4,Xpo1,Prrx1,Dlx1,Padi1,Rasgrp2
NGFIC_01	27	1,23	2,41E-07	Ace,Lef1,Ntn1,Jph1,Hrk,Hoxa7,Erf,Relb,Vax1,Kcnj1,Kcna1,Stc2,Lasp1,Gnl1,Coro1c,Bcl11b,Actn1,Kcnip2,Rel,Egr3,Tle4,Cnnm4,Pdcd1,Kcnh3,Glra3,Kcnd2,Tnfrsf12a
MTF1_Q4	28	1,27	2,49E-07	Gpc6,Rgs6,Dnm1,Cacna1f,Calcr,Hr,Sfrp1,Syt12,Pcdh7,Mrc2,Mllt6,Chrm1,Eif4g2,Polh,Sema6c,Chrd,Mtss1,Cngb1,Mgll,Col12a1,Sema3a,Cyp26a1,Nppc,Hoxc6,Tnmd,Crtac1,Dll1,Mylk
AP4_Q6_01	28	1,27	2,79E-07	Tmod4,Rxrg,Cbfa2t3,Usf1,Ank1,Fgf17,Mycn,Klf13,Barx2,Wnt6,Gas7,Hpca,Erf,Anxa8,Hn1,Trim8,Sez6,Stx17,Chrd,Wnt2b,Sorbs1,Chrng,Dll4,Csf1r,Mycbp,Tnni2,Ckm,Mylk
IRF7_01	29	1,32	2,80E-07	Ecel1,Angptl4,Lhx5,Sox4,Mpl,Sox5,Nog,Esr1,Nos1,Mxd3,Ifnb1,Pde1b,Tlk2,Erbb2,Epha7,Htr5a,Nr4a3,Zdhhc14,Trex2,Plek2,Ifit2,Col12a1,Sall3,Col11a2,Map3k8,Tcf15,Dll4,Elk4,Dlx1
SRF_Q4	30	1,36	2,81E-07	Myo1e,Lsp1,Mmp14,Hoxb5,Mid1,Srd5a2,Fgf17,Aspa,Il17b,Prm1,Hoxb4,Pcdh7,Kcnmb1,Kcna1,Npas2,Coro1c,Jph2,Nppa,Actn1,Tpm3,Egr3,Hoxa5,Slc35a2,Gab2,Caln1,Nr2f2,Ckm,Tnmd,Dll1,Mylk
STAT5A_03	28	1,27	3,09E-07	Kcnn3,Ecel1,Tmc1,Flnc,Dhrs3,Irx3,Rwdd3,Stc1,Figf,Hpca,Hoxb6,Grid2,Plxdc2,Lbx1,Actn1,Nr4a3,Hoxa4,Tpm3,S100a9,Mtss1,Nfatc4,Epha2,Bcar3,Slc35a2,Itga2,Sf3b4,Hoxc6,Coch
E4F1_Q6	53	2,41	3,83E-07	Myo1e,Grpr,Gpc6,Tex14,Blvrb,Gpr3,Lhx5,Kif17,Satb2,Nupl2,Diablo,Gng4,Nos1,Cd160,Syngr3,Erf,Grwd1,Relb,Dsg2,Rfx1,Phospho1,Umps,Pcdh7,Psme3,Cbx3,Nfkb2,Ipo11,Nkx2-3,Kcna6,Mrc2,Gnl1,Tbx5,Slc20a2,Cdkn1a,Spag4,Bcl11b,Sema3b,Nr4a3,Tacr1,Klhl1,Ndufb2,Egr3,Arf4,Phf7,Epha2,Lrrtm1,Evx1,Dnm3,Elk4,Trap1,Drd4,Ckb,Sox10
AP1_Q4_01	29	1,32	3,87E-07	Hoxa11,Esrrg,Itm2b,Synpo,Dtna,Syngr1,Dhrs3,Adam15,Mark1,Col16a1,Wnt6,Ndrg2,Gkn1,Adcy8,Lamc1,Mybph,Baz2a,Lor,Cdkn1a,Slc16a6,Wnt7b,Pappa,Col7a1,Gja1,Cdh23,Csf1r,Rab3d,Map4k5,Mark4
STAT6_01	28	1,27	4,28E-07	Ecel1,Tmc1,Flnc,Nfkbia,Dhrs3,Irx3,Musk,Irx5,Rwdd3,Gas7,Nos1,Stc1,Figf,Hoxb6,Ndrg2,Grid2,Plxdc2,Lbx1,Fxyd2,Actn1,Hoxa4,Mtss1,Nfatc4,Slc35a2,Itga2,Tnfsf11,Sf3b4,Coch
AREB6_02	28	1,27	4,28E-07	Shh,Irx4,Dtna,Sox4,Musk,Wnt10b,Wnt6,Gpr87,Bmf,S100a1,Myl3,Grid2,Syt3,Ptf1a,Chrm1,Tbx19,Egln3,Hrh3,Scn5a,Crat,Col11a2,Sema3a,Ddr1,Dll4,Cdh13,Khdrbs2,Gnat1,Sox10
TAL1BETAITF2_01	27	1,23	4,72E-07	Rasl11b,Atoh7,Htr2c,Rgs3,Hoxa11,Lef1,Flnc,Cbfa2t3,Sln,Mid1,Musk,Mark1,Sox5,Hrk,Pcdh12,Hoxb6,Fbxo36,Grid2,Cdc42ep4,Bcl11b,Gpd1,Sorbs1,Cdh23,Eya3,Dll4,Haao,Zfpm2
HFH8_01	25	1,14	4,87E-07	Sh3bgrl3,Hoxa11,Esrrg,Dtna,Slc35c2,Cacng2,Tnf,Nfib,Cldn8,Runx1,Lta,Lcp2,Hr,Aspa,Chd1,Otx2,Rora,Lasp1,Lbx1,Slc16a6,Bcl11b,Mgll,Hesx1,Ap1g2,Tcf7l2
GATA1_05	29	1,32	5,24E-07	Hoxa11,Gnb1l,Krt15,Nr5a2,Itga2b,Map2k7,Il7,Sox5,Nfe2,Hoxa7,Stc1,Sfrp1,Hoxb6,Otx2,Adcyap1,Anxa13,Rgs1,Bmp7,Pappa,Chrm3,Hesx1,Esrrb,Nkx6-2,Gabra2,Nr2f2,Map4k5,Zfpm2,Plac1,Gata4
AP2GAMMA_01	26	1,18	5,74E-07	Dscaml1,Nfib,Sox5,Klf13,Hr,Gas7,Erf,Relb,Angptl2,Rarg,Mxd4,Tlk2,Gnl1,Map2k2,Drd2,Pdlim2,Sos1,Myh7,Sema6c,Mycl1,Scn5a,Six4,Dll4,Tead2,Sox10,Barx1

NRF2_Q4	27	1,23	5,81E-07	Ids,Arhgap8,Esrrg,Itm2b,Blvrb,Synpo,Flnc,Adam15,Nog,Foxn1,Ndrg2,Kcnd1,Kif5a,Rarg,Limk1,Adcyap1,Lamc1,Baz2a,Slc16a6,Dusp13,Pitpnc1,Usp13,Capn12,Ogg1,Cdh23,Kcnh3,Loxl4
IRF1_01	27	1,23	5,81E-07	Blnk,Usf1,Vgll4,Sema7a,Fxyd5,Pigr,Plekhb1,Nog,Gnao1,Esr1,Aspa,Wnt8b,Mxd3,Hoxb6,Vax1,Ifnb1,Tlk2,Trim8,Pitx1,Blk,Stx17,Neto1,Sorbs1,Tnfsf13b,Aak1,Elk4,Dlx1
SRF_C	26	1,18	6,37E-07	Myo1e,Slc2a4,Rgs3,Hoxb5,Srd5a2,Aspa,Il17b,Prm1,Hoxb4,Kcnmb1,Npas2,Itgb6,Jph2,Actn1,Tpm3,Ldb3,Egr3,Hoxa5,Pdlim7,Nkx6-2,Pnkp,Aak1,Nr2f2,Ckm,Dll1,Mylk
IRF1_Q6	28	1,27	6,45E-07	Esrrg,Lsp1,Tnfrsf19,Usf1,Lmx1a,Vgll4,Adam15,Fcgr2b,Prkd2,Bmf,Aspa,Myl3,Hoxb4,Cbx3,Slc26a6,Grid2,Erbb2,Lamc1,Pitx3,Neto1,Sorbs1,Tyrobp,Tnfsf13b,Sntb2,Tcf15,Dlx1,Col9a1,Sox14
AR_Q6	25	1,14	6,85E-07	Coro2a,Shh,Lhx5,Mid1,Slc22a6,Slc12a1,Pou2f3,Prm1,Angptl2,Vax1,Kcnmb1,Epha7,Klhl10,Sez6,Bmp7,Mll2,Tpm3,Epha2,Supt16h,Dll4,Sox14,Ndst2,Fga,Hoxc6,Mylk
FOXO4_02	27	1,23	7,16E-07	Hs3st1,Dtna,Flnc,Ntn1,Nxn,Insr,Runx1,Esr2,Lcp2,Gnao1,Satb2,Hr,Hoxb4,Il6st,Plxdc2,Aqp2,Trim8,Acvr1b,Eif4g2,Pitpnc1,Pappa,Ndufb2,Sall3,Scn3a,Tcf15,Rbp2,Dll1
SRY_01	23	1,05	7,60E-07	Entpd1,Esrrg,Usf1,Dhrs3,Sox5,Dpysl4,Kcna1,Plxdc2,Mtx1,Lbx1,Ascl2,Tbx5,Trim8,Elf5,Eif4g2,Sorbs1,Gab2,Tcf7l2,Prrx1,Cyp26a1,Hoxc6,Ncam1,Pde6c
MAZR_01	25	1,14	7,63E-07	Entpd1,Slc2a4,Fxyd1,Pacsin3,Cacnb1,Fgf17,Hr,Hpca,Hmx1,Hoxb6,Rfx1,Stc2,Syt3,Grik1,Tlk2,Dll3,Coro1c,Mybph,Baz2a,Mllt6,Clcn6,Neto1,Dll4,Tcf7l2,Hoxc6
ICSBP_Q6	27	1,23	7,90E-07	Entpd1,Angptl4,Tnfrsf19,Usf1,Vgll4,Il21,Plp1,Pigr,Slc15a3,Satb2,Esr1,Prkd2,Aspa,Cyp7a1,Kcnj1,Ifnb1,Zbp1,Erbb2,Ascl2,Tlr7,Sos1,Sorbs1,Cited4,Aif1,Tnfsf13b,Tcf15,Elk4
XBP1_01	19	0,86	7,91E-07	Hoxa11,Gpr3,Lhx5,Mmp14,Hoxb5,Syt3,Adcyap1,Npas2,Sox21,Bcl11b,Rgs1,Sema3b,Nr4a3,Gys1,Ruvbl2,Magel2,Sntb2,Dnm3,Prrx1
ATF3_Q6	47	2,14	8,08E-07	Barhl1,Traf4,Tex14,Itm2b,Gpr3,Irx4,Tnfrsf19,Syngr1,Ccl4,Sln,Nt5c1b,Dusp3,Mid1,Runx1,Klf13,Prss12,Nupl2,Syngr3,Relb,Rfx1,Angptl2,Umps,Fgf23,Loxl3,Adcy8,Cdc42ep4,Gnl1,Trim8,Dnajc5b,Cdkn1a,Egfr,Adarb2,Rad23a,Il13,Pitpnc1,Tpm3,Mtss1,Ndufb2,Epha2,Gab2,Trap1,Osbpl9,Nppc,Kcnk7,Pthlh,Tnfrsf12a,Adra1b
OCT1_06	28	1,27	8,59E-07	Hs3st1,Hoxa11,Msr1,Pacsin3,Bcl2,Irx3,Nfib,Sox5,Chml,Gnao1,Wnt6,Hoxa7,Stc1,Ndrg2,Hoxb4,Inpp4a,Anxa8,Cars,Sema6a,Sema6c,Scn3a,Prrx1,Dlx1,Hnf4g,Odf1,Map4k5,Ascl3,Fgfr3
SPZ1_01	27	1,23	8,66E-07	Ecel1,Shh,Irx5,Fgf17,Hr,Il17b,Hoxb6,Hoxb4,Nfkb2,Mrc2,Baz2a,Kcnk4,Nppa,Eif4g2,Slitrk1,Pitpnc1,Tpm3,Six4,Hira,Xpo1,Sigirr,Nr2f2,Prrx1,Pgr,Sox10,Smarcc1,Mylk
AHRARNT_01	19	0,86	9,14E-07	Rgs6,Sox4,Runx1,Il7,Gnao1,Syt12,Rarg,Stc2,Ptf1a,Gabra1,Lhx1,Sez6,Slitrk1,Pitpnc1,Egr3,Hoxa5,Dll4,Tcf7l2,Atoh1
GR_01	23	1,05	9,51E-07	Htr2c,Rgs3,Cuedc1,Fgf17,Jph1,Wnt8b,Kcnj1,Epha7,Cdkn1a,Cxcl14,Dlx2,Dlx3,Sorbs1,Sema3a,Dll4,Cer1,Prrx1,Tead2,Fen1,Ndst2,Hoxc6,Sdc1,Tnmd
GATA_Q6	24	1,09	1,15E-06	Trim10,Pdgfra,Phox2a,Lcat,Esrrg,Pla2g1b,Nr5a2,Il7,Jph1,Spink4,Slc4a1,Vps18,Hoxb6,Aqp2,Tbx5,Mos,Suv39h1,Hoxd1,Adm,Trim15,Pklr,Tnfsf13b,Epor,Plac1

STAT_Q6	27	1,23	1,18E-06	Ecel1,Traf4,Mmp14,Irx4,Fgfr2,Slamf1,Nkx2-3,Npas2,Cox5b,Dnajc5b,Jph2,Sema6a,Bach2,Nr4a3,Smpd3,Neto1,Nfatc4,Six4,Scn3a,Ccl20,Abcc5,Tnfsf11,Ptch2,Iapp,Ndst2,Sec14l2,Pthlh
CIZ_01	25	1,14	1,18E-06	Blnk,Nr5a2,Plp1,Col16a1,Adra2b,Nrip2,Pcdh12,Pcdh7,Tecta,Slc4a5,Bcl11b,Batf,Slc8a3,Trex2,Pitpnc1,Pappa,Edar,Sema3a,Reg4,Nr2f2,Prrx1,Odf1,Zfpm2,Ncam1,Mylk
HEN1_01	23	1,05	1,21E-06	Ace,Hoxa11,Esrrg,Traf4,Capn5,Tbx3,Hoxb5,Ank1,Doc2b,Klf13,Kcnd1,Hoxb4,Dusp9,Nr4a3,Tpm3,Klhl1,Mgll,Ptprs,Asb16,Dll4,Sox14,Plac1,Dll1
HFH4_01	36	1,64	1,63E-06	Myo1e,Pdgfra,Myo10,Hoxa11,Tmod4,Esrrg,Dtna,Ntn1,Tbx3,Runx1,Fgf17,Tacstd2,Col10a1,Nfe2l3,Fbxo36,Hoxb4,Otx2,Lbx1,Cntnap4,Trim8,Itgb6,Bcl11b,Nr4a3,Bmp7,Hoxa4,Mgll,Hesx1,Sema3a,Sntb2,Htr1b,Tcf7l2,Nr2f2,Drd3,Cyp26a1,Atoh1,Ncam1
STAT5A_04	22	1,00	1,64E-06	Blnk,Tnfrsf19,Nr5a2,Irx3,Map2k7,Hoxb6,Slco1c1,Grid2,Dusp9,Ntng2,Ankrd1,Actn1,Cyp17a1,Ap1g2,Slc26a3,Aak1,Drd3,Atoh1,Hoxc6,Zfpm2,Tnmd,Ncam1
MEIS1BHOXA9_01	19	0,86	1,66E-06	Hoxa11,Gnb1l,Esrrg,Irx3,Chodl,Hrk,Nog,Gnao1,Wnt6,Nfkb2,Otx2,Klrc2,Klhl1,Tle4,Actn2,Cdh13,Nr2f2,Drd3,Hnf4g
LFA1_Q6	26	1,18	1,81E-06	Slc2a4,Adamts4,Lcat,Gpr3,Tpcn1,Nfkbia,Nr5a2,Cacnb1,Sox5,Gnao1,Dhh,Pla2g10,Nkx2-3,Mybph,Sema6a,Bcl11b,Six4,Adm,Hira,Pdlim7,Myog,Prrx1,Dlx1,Ckb,Hoxc6,Rasgrp2
STAT5A_02	18	0,82	1,81E-06	Hoxa11,Gfap,Il1rn,Oxa1l,Runx1,Stc1,Hoxb6,Ipo11,Grid2,Fzd6,Cyp46a1,Stard3,Nr4a3,Nfatc2,Cited4,Ap1g2,Hnf4g,Sdc1
MZF1_02	25	1,14	2,00E-06	Map4k1,Gpc6,Slc6a12,Hr,Nos1,Vamp1,Hmx1,Vax1,Cbx3,Stc2,Mrc2,Mllt6,Bcl11b,Cxcl14,Kcnip2,Rel,Nfatc4,Egr3,Lrrtm1,Xpo1,Supt16h,Ror1,Tcf7l2,Atp1a2,Rasgrp2
GCM_Q2	25	1,14	2,00E-06	Barhl1,Htr2c,Traf4,Blvrb,Slc6a13,Gpr3,Slc6a12,Wnt10b,Hcst,Col2a1,Gnao1,Stc1,Vax1,Kcnj1,Loxl3,Col25a1,Mfap5,Pitx3,Trim8,Ankrd1,Epha2,Lrrtm1,Dll4,Kcnc3,Adra1b
GNCF_01	13	0,59	2,00E-06	Insr,Nfib,Nfe2,Nog,Gnao1,Hoxb6,Slco1c1,Myl3,Mllt6,Bcl11b,Slitrk1,Aak1,Hoxc6
STAT5A_01	26	1,18	2,00E-06	Kcnn3,Adamts4,Ecel1,Hsf4,Synpo,Ccl2,Pcolce,Stc1,Vax1,Col25a1,Kcnk4,Tlr7,Nr4a3,Batf,Nfatc4,Cited4,Lama2,Trim15,Abcc5,Tnfsf11,Ogg1,Dll4,Sigirr,Cyp26a1,Fga,Sdc1
PAX3_B	23	1,05	2,16E-06	Barhl1,Tex14,Slc6a12,Tbx3,Relb,Nkx2-3,Npas2,Trim8,Cdkn1a,Bcl11b,Nr4a3,Ndufb2,Egr3,Sema3a,Il24,Xpo1,Supt16h,Elk1,Tead3,Hoxc6,Sox10,Rpl27a,Dll1
SRF_Q6	28	1,27	2,23E-06	Myo1e,Lsp1,Hoxb5,Srd5a2,Dhh,Il17b,Prm1,Hoxb4,Pcdh7,Kcnmb1,Kcna1,Npas2,Coro1c,Aqp2,Jph2,Nppa,Actn1,Tpm3,Ldb3,Nfatc4,Egr3,Hoxa5,Pdlim7,Nr2f2,Ckm,Atp1a2,Tnmd,Mylk
AP4_01	26	1,18	2,40E-06	Hoxa11,Col11a1,P2ry4,Nfib,Sema7a,Wnt6,Cacng6,Angptl2,Lbx1,Baz2a,Stard3,Tlr7,Kif3c,Crb1,Accn5,Sorbs1,Ptprs,Dpf1,Epor,Evx1,Loxl4,Cdh13,Cmklr1,Dlx1,Ucp3,Pde3a
MYCMAX_B	26	1,18	2,40E-06	Hsf4,Sox4,Sema7a,Slc1a1,Klf13,Hpca,Syt12,Vax1,Ascl2,Ptf1a,Lamb1,Pitx3,Hn1,Trim8,Bcl11b,Cxcl14,Zdhhc14,Nfatc4,Egr3,Catsper2,Phf7,Epha2,Hs3st3b1,Dll4,Crabp2,Acy1

SOX5_01	26	1,18	2,40E-06	Hs3st1,Esrrg,Dtna,Fgfr2,Dhrs3,Hoxb5,Nfib,Tacstd2,Mark1,Sox5,Satb2,Prss12,Stc1,Myl3,Pcdh7,Mrc2,Mllt6,Pitx1,Sema6a,Sema6c,Hesx1,Sall3,Sema3a,Dlx1,Ncam1,Mylk
RORA1_01	26	1,18	2,95E-06	Lcn2,Rgs6,Shh,Dtna,Slc22a6,Nfya,Il7,Nog,Vamp1,Ndrg2,Myl3,Kif5a,Adcyap1,Epha7,Npas2,Mllt6,Gtf2ird1,Slc8a3,Tpm3,Pacsin1,Esrrb,Nr2f2,Khdrbs2,Trap1,Nell2,Mylk
CEBPB_02	29	1,32	3,02E-06	Pdgfra,Asah2,Cyp24a1,Cuedc1,Nfkbia,Dbh,P2ry4,Sox5,Esr1,Slc12a1,Bmf,Erf,Pcdh7,Mrc2,Ascl2,Ddr2,Chrm1,Fmo2,Aqp9,Havcr1,S100a9,Gys1,Bdkrb1,Cited4,Hoxa5,Ruvbl2,Dlx1,Fga,Sox10
STAT_01	26	1,18	3,25E-06	Adamts4,Gif,Lta,Ccl2,Pcolce,Klf13,Il18rap,Vax1,Hoxb4,Il6st,Ipo11,Lasp1,Bach2,Homer2,Nr4a3,Batf,Cited4,Trim15,Abcc5,Tnfsf11,Myog,Ckb,Fga,Ascl3,Sdc1,Lpo
NFAT_Q6	25	1,14	3,30E-06	Slc6a12,Sox5,Klf13,Gnao1,Col16a1,Pcdh12,Ndrg2,Hoxb4,Ifnb1,Lasp1,Col25a1,Ddr2,Sox21,Elf5,Slitrk1,Kif3c,Nfatc4,Epha2,Esrrb,Dll4,Tnfrsf1a,Nr2f2,Plac1,Sox10,Gata4
P300_01	25	1,14	3,30E-06	Kcnn3,Esrrg,Nfkbia,Plp1,Adam15,Vax1,Gjb2,Rarg,Lbx1,Gabra1,Mllt6,Slitrk1,Smpd3,Rgs14,Wnt7b,Egr3,Mycl1,Six4,Kcnh3,Nkx6-2,Tnfrsf1a,Dlx1,Hoxc6,Tnfrsf12a,Rasgrp2
CEBPDELTA_Q6	25	1,14	3,30E-06	Ace,Hoxa11,Sprr1b,Apex2,Dtna,Slc35c2,Il1rn,Asb18,Mid1,Nnmt,Wnt10b,Wnt6,Tal2,Stc1,Pde1b,Stc2,Rora,Rph3a,Egfr,Gpd1,Vipr2,Lipg,S100a9,Sema3a,Cer1
CEBPGAMMA_Q6	26	1,18	3,54E-06	Entpd1,Pdgfra,Pacsin3,Mid1,B4galt2,Cdca3,Actn3,Pdzk1,Stc1,Kcna1,Alb,Gabra1,Trim8,Sema6a,Adarb2,Chrna6,Hoxa4,Mgll,Hesx1,Sall3,Aak1,Dlx1,Sox14,Hnf4g,Osbpl9,Ndst2
HIF1_Q5	25	1,14	3,63E-06	Dscaml1,Klhdc3,Lhx5,Bcl2,Slc12a5,Irx4,Slc6a12,Cacna2d2,Mpl,Fgf17,Gadd45b,Erf,Psme3,Rora,Npas2,Bcl11b,Pitpnc1,Lrrtm1,Recql,Hira,Creb3,Dll4,Sox14,Entpd7,Slc6a1
MYOD_01	25	1,14	3,63E-06	Coro2a,Kcnn3,Atoh7,Htr2c,Cldn9,Rxrg,Cacna2d2,Pax9,Musk,Itsn1,Wnt6,Stc2,Rora,Grid2,Prkcq,Mllt6,Hrh3,Scn5a,Col11a2,Col18a1,Caln1,Chrng,Aak1,Cyp26a1,Ckm
HAND1E47_01	26	1,18	3,87E-06	Mmp14,Sln,Ank1,Musk,Jph1,Sox5,Lcp2,Nog,Tal2,Wnt8b,Fzd1,Stc1,Hoxb6,Sell,Nkx2-3,Lhx1,Klhl10,Nfatc2,Hrh3,Wnt2b,Col12a1,Phf3,Nr2f2,Elk4,Hoxc6,Ddit4l
TAL1ALPHAE47_01	25	1,14	4,00E-06	Atoh7,Htr2c,Hoxa11,Hck,Cbfa2t3,Sln,Mark1,Hrk,Wnt6,Pcdh12,Gjb2,Stc2,Grid2,Cdc42ep4,Npas2,Bcl11b,Gpd1,Scn5a,Six6,Pmf1,Cdh23,Eya3,Chrng,Dll4,Haao
FREAC3_01	25	1,14	4,43E-06	Hoxa11,Esrrg,Lhx5,Lef1,Dtna,Tnfrsf19,Ntn1,Lcp2,Nog,Gnao1,Vax1,Otx2,Trim8,Itgb6,Cdkn1a,Bcl11b,Spdef,Hoxa4,Pappa,Lamb3,Slc35a2,Tcf15,Tcf7l2,Map4k5,Plunc
AP1_Q2_01	28	1,27	4,53E-06	Gpr3,Gfap,Flnc,Il1rn,Adam15,Mgst3,Wnt6,Gpr87,Ndrg2,Adcy8,Dusp9,Mybph,Ddr2,Srgap2,Chrm1,Cdkn1a,Cxcl14,Homer2,Sema3b,Ldb3,Usp13,Nfatc4,Lamb3,Hs3st3b1,Gab2,Adora2a,Dlg4,Tnfrsf12a
ALPHACP1_01	25	1,14	4,88E-06	Enam,Cyp24a1,Sds,Rad9b,Mid1,Nfya,Cdca3,Slc25a13,Wnt8b,Sfrp1,Erf,Otx2,Dhcr24,Slc25a10,Dlx3,Nfatc4,Esrrb,Tnfsf11,Klf1,Tpcn2,Xpo1,Nkx6-2,Elk1,Sox14,Atoh1
CEBPA_01	25	1,14	5,39E-06	Asah2,Htr2c,Sprr1b,Cuedc1,Nxn,Cldn8,Wnt10b,Jph1,Chml,Esr1,Slc12a1,Stc1,Otx2,Ascl2,Trim8,Aqp9,Egfr,Rhobtb2,Gys1,Ruvbl2,Tnfsf11,Tnfsf13b,Fbn2,Nr2f2,Dlx1
NKX25_02	26	1,18	5,64E-06	Fgf3,Htr2c,Esrrg,Aqp3,Vgll4,Hspb3,Wnt8b,Hoxb6,Rora,Dnajc5b,Sez6,Tbx19,Slc16a6,Cxcl14,Nr4a3,Gprc5d,Klhl1,Hesx1,Col12a1,Gja1,Cer1,Prrx1,Dlx1,Atoh1,Hoxc6,Nell2

MYB_Q6	24	1,09	6,17E-06	Esrrg,Synpo,Hoxb5,Cldn8,Sema7a,Cdca3,Aspa,Stc1,Hoxb4,Rarg,Stc2,Ddr2,Slc16a6,Eif4g2,Mll2,Mgll,Phf7,Odf4,Cdh13,Gnat1,Dlx1,Kcnk7,Hoxc6,Sdc1
HIF1_Q3	23	1,05	6,28E-06	Dscaml1,Klhdc3,Lhx5,Bcl2,Slc12a5,Mpl,Fgf17,Jph1,Hoxa7,Stc1,Erf,Psme3,Bcl11b,Mgll,Gys1,Lrrtm1,Ruvbl2,Recql,Nr2f2,Sox14,Entpd7,Mylk,Slc6a1
STAT4_01	25	1,14	6,51E-06	Gif,Blnk,Tnfrsf19,Il21,Hspb7,Map2k7,Erf,Dhh,Myl3,Hoxb4,Grid2,Pdlim2,Actn1,Pitpnc1,Gprc5d,Cspg4,Ap1g2,Slc26a3,Fank1,Tcf15,Aak1,Hnf4g,Csrp3,Hoxc6,Ncam1
HOXA4_Q2	26	1,18	7,45E-06	Fgf3,Mmp14,Apex2,Rwdd3,Sox5,Col10a1,Stc1,Zfp36l2,Otx2,Adcyap1,Myh8,Npas2,Tecta,Bmpr1b,Ankrd1,Cacna1s,Eif4g2,Slitrk1,Eomes,Hoxa4,Lrrtm1,Ddx5,Cldn14,Prrx1,Dlx1,Hoxc6
COUP_DR1_Q6	24	1,09	7,49E-06	Clcnka,Fxyd1,Lcat,Traf4,Apex2,Prkcd,Tpcn1,Insr,Pdzk1,Rfx1,Prrx2,Prodh2,Slc26a6,Erbb2,Cxcl14,Nr4a3,Klk6,Pdlim7,F12,Slc26a3,Nr2f2,Ovol1,Cyp26a1,Crabp2
SP1_Q6_01	23	1,05	7,69E-06	Ace,Phox2a,Gpr3,Rab2b,Cdc37,Irx3,Map2k7,Adam15,Shmt1,Iqgap1,Gas7,Relb,Lasp1,Cdk5r1,Coro1c,Ascl2,Wnt2b,Cspg4,Cnnm4,Loxl4,Ptch2,Tead2,Elac2
OCT1_05	25	1,14	7,83E-06	Kcnn3,Rgs3,Esrrg,Bcl2,Dtna,Fgfr2,Mid1,Sema7a,Sox5,Satb2,Nos1,Pou2f3,Col25a1,Enpp1,Nr4a3,Trex2,Tll2,Sema6c,Col12a1,Stat4,Hoxa5,Cnnm4,Prrx1,Dlx1,Ascl3
HLF_01	25	1,14	8,58E-06	Sprr1b,Cacng2,Ifng,Ndst3,Erf,Hoxb6,Otx2,Rora,Mrc2,Alb,Inpp4a,Fgf21,Trim8,Aqp9,Egfr,Zdhhc14,Hoxa4,Hrh3,Rhobtb2,Wnt3a,Ddx5,Nr2f2,Dlx1,Hoxc6,Pthlh
ATF4_Q2	25	1,14	8,58E-06	Tex14,Itm2b,Cyp24a1,Flnc,Nrip3,Hcst,Klf13,Bmf,Nupl2,Syngr3,Ndrg2,Relb,Cbx3,Cdk5r1,Gnl1,Mybph,Baz2a,Gabra1,Hn1,Nr4a3,Sorbs1,Epha2,Capn12,Cdh23,Xpo1
CDPCR3HD_01	24	1,09	9,02E-06	Slc12a5,Tbx3,Nfib,Dnajb8,Sox5,Nog,Col16a1,Sftpc,Kcnd1,Hoxb4,Cbx3,Kcna1,Rph3a,Ntng2,Kcns1,Eif4g2,Egr3,Dpf1,Cpne6,Caln1,Cdh13,Tcf7l2,Hnf4g,Gata4
POU3F2_01	15	0,68	9,91E-06	Hoxa11,Sox4,Syngr1,Il21,Aspa,Hoxb6,Vax1,Epha7,Bcl11b,Mos,Klhl1,Sall3,Supt16h,Prrx1,Pthlh
AP2_Q3	25	1,14	1,14E-05	Usf1,Cacng2,Tbx3,Mycn,Slc4a1,Gas7,Erf,Relb,Kcnd1,Kcna1,Cpsf4,Kcna6,Cdk5r1,Lbx1,Bcl11b,Eif4g2,Polh,Pitpnc1,Mgll,Col11a2,Hira,Creb3,Abhd1,Barx1,Crtac1
EVI1_05	20	0,91	1,18E-05	Arhgap8,Tmod4,Esrrg,Sufu,Hoxb5,Mid1,Irx5,Il7,Spink4,Sox5,Gnao1,Satb2,Vamp1,Ptgfr,Mgll,Sall3,Elovl3,Sox14,Zfpm2,Plac1
NERF_Q2	24	1,09	1,20E-05	Map4k1,Arhgap8,Adamts4,Rgs3,Fgfr2,Mid1,Tro,Fxyd5,Mpl,Pou4f3,Cdca3,Klf13,Fcgr2b,Diablo,Fbxo36,Kcnd1,Pcdh7,Loxl3,Baz2a,Hn1,Egr3,Sorbs1,Creb3,Ptpn6
E4BP4_01	28	1,27	1,23E-05	Enam,Gabrr2,Atoh7,Esrrg,Rgs6,Slc35c2,Vgll4,Ifng,Slc22a8,Ndst3,Wnt8b,Nos1,Erf,Kcnj1,Il6st,Alb,Inpp4a,Trim8,Zdhhc14,Hoxa4,Hrh3,Rhobtb2,Accn5,Ddx5,Hoxc6,Pthlh,Crtac1,Mylk
AMEF2_Q6	25	1,14	1,24E-05	Rgs3,Esrrg,Pacsin3,Asb18,Hoxb5,Nfib,Mid1,Sox5,Nog,Gnao1,Hoxb6,Slitrk1,Tacr1,Usp13,Sall3,Sema3a,Slco2a1,Slc35a2,Asb16,Prrx1,Col9a1,Hoxc6,Slc3a1,Sdc1,Ckm
STAT1_02	23	1,05	1,25E-05	Hsd17b4,Arhgap9,Sox4,Usf1,Stc1,Erf,Dhh,Hoxb6,Vax1,Cbx3,Tlk2,Gabra1,Chrm1,Mrpl34,Rab8b,Smpd3,Map3k8,Sf3b4,Dlx1,Odf1,Sec14l2,Plac1,Dll1
CDX2_Q5	25	1,14	1,34E-05	Hoxa11,Tmod4,Gnb1l,Irx4,Dtna,Sox4,Nfib,Pou4f3,Wnt8b,Hoxa7,Hoxb6,Pcdh7,Epha7,Tbx5,Sez6,Bach2,Pappa,Accn5,Tle4,Pmf1,Cer1,Dlx1,Sox14,Hoxc6,Zfpm2

CP2_01	25	1,14	1,34E-05	Rgs3,Cox8c,Dhrs3,B4galt2,Hr,Foxn1,Bmf,Dhh,Kif5a,Anxa8,Alox12,Atp6v1e2,Jph2,Gpd1,Slc8a3,Guca2b,Phf7,Col11a2,Pde2a,Ddr1,Dll4,Tcf7l2,Sox10,Crtac1,Rasgrp2
SF1_Q6	30	1,36	1,35E-05	Ndufs8,Slc2a4,Syngr1,Comtd1,Insr,Sema7a,Mgst3,Ndrg2,Myl3,Kif5a,Pcdh7,Cdh16,Mtx1,Rph3a,Cyp46a1,Ntng2,Ptpn5,Cox5b,Polr3d,Ppp3cc,Gipc2,Slitrk1,Ldb3,Bub1b,Catsper2,Scn5a,Trap1,Ckb,Auh,Rasgrp2
PR_02	17	0,77	1,38E-05	Rgs3,Fgf17,Jph1,Sox5,Wnt8b,Kcnj1,Otx2,Epha7,Tecta,Cdkn1a,Elf5,Cxcl14,Dlx2,Sema3a,Prrx1,Ndst2,Sdc1
NKX3A_01	22	1,00	1,41E-05	Kcnn3,Lhx5,Slc35c2,Six3,Pax9,Ifng,Mid1,Tacstd2,Sox5,Lcp2,Gpr12,Nog,Stc1,Myh8,Cntnap4,Chrm1,Spdef,Slitrk1,Procr,Lrrtm1,Hoxc6,Zfpm2
TFIII_Q6	22	1,00	1,41E-05	Esrrg,Pax9,Capn5,Bmf,Ndrg2,Fbxo36,Psme3,Rarg,Mrc2,Coro1c,Baz2a,Chrm1,Klhl1,Nfatc4,Six4,Abcc6,Kcnh3,Xpo1,Ptch2,Nr2f2,Prrx1,Atp1a2
OCT_Q6	25	1,14	1,44E-05	Kcnn3,Blnk,Irx4,Mid1,Itsn1,Wnt6,Hoxa7,Pou2f3,Hoxb6,Hoxb4,Otx2,Col25a1,Anxa2,Lipg,Rel,Tll2,Sema6c,Stat4,Cnnm4,Gab2,Prrx1,Dlx1,Adora2a,Pthlh,Ascl3
CHOP_01	23	1,05	1,49E-05	Htr2c,Hoxa11,Slc12a5,Dyrk1b,Otos,Sox4,Nfkbia,Slc6a2,Stc1,Cyp7a1,Pcdh7,Lasp1,Inpp4a,Cars,Gabra1,Fgf21,Smoc2,S100a9,Adm,Hoxa5,Evx1,Supt16h,Slc35a5
FOXO1_01	24	1,09	1,53E-05	Hoxa11,Esrrg,Lhx5,Dtna,Nfib,Runx1,Nfya,Lcp2,Bmf,Chd1,Hoxb4,Plxdc2,Lasp1,Lhx1,Trim8,Bcl11b,Crb1,Pappa,Mgll,Hesx1,Sall3,Kcnn1,Gpr63,Dlx1
MYB_Q5_01	24	1,09	1,53E-05	Htr2c,Esrrg,Nmur1,Cldn8,Sema7a,Cdca3,Sox5,Shmt1,Mxd3,Gmfg,Rarg,Gnl1,Ddr2,Srgap2,Slc16a6,Dlx2,Mll2,Guca2b,Mgll,Six4,Odf4,Cpne6,Xpo1,Gnat1
HMEF2_Q6	17	0,77	1,77E-05	Hoxb5,Kcnj9,Il17b,Phospho1,Epha7,Jph2,Slitrk1,Kcnn1,Slco2a1,Art5,Dll4,Sox14,Wfdc1,Zfpm2,Slc3a1,Ckm,Gata4
HP1SITEFACTOR_Q6	23	1,05	2,17E-05	Barhl1,Rgs3,Hoxa11,Esrrg,Sox4,Nr5a2,Nog,Stc1,Acrv1,Afp,Lhx1,Atp6v1e2,Sez6,Eif4g2,Hoxa4,Usp13,Tle4,Phf3,Xpo1,Fbn2,Dlx1,Drd3,Mycbp
CEBP_C	21	0,96	2,18E-05	Pdgfra,Sox4,Cacng2,Nfkbia,Sema7a,Slc22a8,Wnt10b,Slc12a1,Stc2,Rora,Ddr2,Sez6,Bcl11b,Lrrtm1,Ror1,Ltb,Glra3,Ovol1,Dlx1,Ascl3,Gata4
LMO2COM_02	24	1,09	2,21E-05	Trim10,Lcat,Pla2g1b,Nr5a2,Cacna1f,Runx1,Sema7a,Sox5,Slc4a1,Vps18,Sftpc,Ndrg2,Grid2,Aqp2,Tbx5,Hyal3,Amhr2,Tbx19,Slc8a3,Dlx3,Adm,Trim15,Tnfsf13b,Map4k5
FOXM1_01	24	1,09	2,42E-05	Atoh7,Arhgap9,Chodl,Sox5,Pdzk1,Hoxa7,Hoxb6,Nln,Lbx1,Lhx1,Pitx1,Slitrk1,Mtss1,Nfatc4,Sall3,Tle4,Sema3a,Ogg1,Gab2,Cdc25c,Dlx1,Hnf4g,Nppc,Ncam1
FREAC7_01	20	0,91	2,45E-05	Hoxa11,Pacsin3,Ier3,Dtna,Nfib,Mid1,Cldn8,Nfya,Lcp2,Hoxb4,Ptf1a,Alpl,Trim8,Bcl11b,Rp1,Mgll,Hesx1,Ogg1,Tcf7l2,Gata4
SP1_Q2_01	24	1,09	2,64E-05	Ace,Phox2a,Gpr3,Rab2b,Tbx3,Klf13,Gas7,Relb,Mxd4,Lasp1,Cdk5r1,Coro1c,Ascl2,Tsnaxip1,Bcl11b,Dlx3,Kcnip2,Hrh3,Six4,Loxl4,Ptch2,Nr2f2,Osbpl9,Pthlh
OCT1_B	25	1,14	3,13E-05	Kcnn3,Bcl2,Blnk,Irx4,Mid1,Satb2,Itsn1,Wnt6,Pou2f3,Hoxb6,Otx2,Col25a1,Lipg,Rel,Tll2,Rhobtb2,Ifit2,Stat4,Cnnm4,Gab2,Cdh13,Prrx1,Adora2a,Pthlh,Ascl3
PXR_Q2	24	1,09	3,13E-05	Oxa1l,Syngr1,Nr5a2,Dhrs3,Sln,Adam15,Rwdd3,Slc6a6,Prss12,Ndrg2,Tob2,Gtpbp1,Rarg,Npas2,Mllt6,Dnajc5b,Nr4a3,Ap1g2,Serpinc1,Supt16h,Ovol1,Prrx1,Gata4,Ftcd

EGR1_01	24	1,09	3,13E-05	Ace,Lef1,Tbx3,Runx1,Pcolce,Hrk,Hr,Fzd1,Hoxa7,Erf,Relb,Mrc2,Coro1c,Klhl10,Mgll,Egr3,Tle4,Hoxa5,Cnnm4,Ddx5,Dll4,Glra3,Kcnd2,Tnfrsf12a
EGR_Q6	25	1,14	3,37E-05	Ace,Traf4,Il21,Klf13,Erf,Relb,Map3k7,Fbxo36,Vax1,Adcy8,Coro1c,Rad23a,Cyhr1,Dlx3,Neto1,Mgll,Six4,Cnnm4,Nthl1,Kcnh3,Pde10a,Dll4,Abhd1,Nr2f2,Tnfrsf12a
POU3F2_02	24	1,09	3,40E-05	Enam,Shh,Prdx2,Musk,Itsn1,Aspa,Gtpbp1,Otx2,Col25a1,Pou1f1,Lipg,Hoxa4,Rp1,Sall3,Adm,Sema3a,Phf3,Supt16h,Cdh13,Tead3,Dlx1,Atoh1,Hs6st3,Hoxc6
CEBP_Q2	22	1,00	3,63E-05	Pdgfra,Asah2,Htr2c,Ppp1r3a,Ahsg,Nfkbia,Itsn1,Vamp1,Stc1,Slco1c1,Otx2,Ascl2,Inpp4a,Ptgis,Lipg,Ptx3,Suv39h1,Rhobtb2,Gys1,Ruvbl2,Nr2f2,Odf1
TGIF_01	22	1,00	3,63E-05	Pdgfra,Irx4,Tnfrsf19,Sox4,Sema7a,Chodl,Jph1,Bmp1,Psme3,Adcy8,Tlk2,Lbx1,Lhx1,Slc16a6,Nppa,Nr4a3,Col7a1,Klhl1,Lrrtm1,Gab2,Foxi1,Dnm3
TST1_01	24	1,09	3,66E-05	Htr2c,Gnb1l,Esrrg,Cacna2d2,Fgfr2,Il21,Irx3,Wnt6,Bmf,Nfkb2,Otx2,Adcyap1,Lbx1,Epha7,Tbx5,Ccnf,Nr4a3,Usp13,Mgll,Hoxd1,Hoxa5,Abcc6,Nr2f2,Hoxc6
ER_Q6_01	24	1,09	3,66E-05	Kcnn3,Gnb1l,Shh,Gpr3,Apex2,Gfap,Mid1,Map2k7,Nog,Pdzk1,Gas7,Ndrg2,Gtpbp1,Zfp36l2,Otx2,Grid2,Slc16a6,Trex2,Tpm3,Esrrb,Atp6v0a4,Ddr1,Ckb,Sdc1
MYB_Q3	22	1,00	3,96E-05	Hoxa11,Esrrg,Nmur1,Sema7a,Cdca3,Sox5,Shmt1,Stc1,Mxd3,C4b,Gmfg,Ager,Rarg,Gnl1,Srgap2,Slc16a6,Mll2,Mgll,Col12a1,Phf7,Xpo1,Gnat1
PPAR_DR1_Q2	23	1,05	3,99E-05	Tpcn1,Insr,Col16a1,Pdzk1,Slc25a13,Hoxb6,Prrx2,Pcdh7,Prodh2,Erbb2,Mllt6,Cxcl14,Nr4a3,Gpd1,Klk6,F12,Ror1,Nr2f2,Ovol1,Rbp2,Dlg4,Crabp2,Tnfrsf12a
SP1_01	23	1,05	4,35E-05	Klk8,Gpr3,Bcl2,Nfkbia,B4galt2,Kcnd1,Ascl2,Tbx5,Tsnaxip1,Alox12,Rem1,Bcl11b,Mtss1,Col12a1,Six4,Pdlim7,Dnm3,Dll4,Ptch2,Nr2f2,Osbpl9,Ndst2,Kcnd2
EN1_01	14	0,64	4,65E-05	Pdgfra,Hoxa11,Rgs6,Mmp14,Irx4,Rab2b,Eln,Fgf17,Nos1,Otx2,Nr4a3,Eomes,Hoxa4,Myog
OCT1_01	24	1,09	4,68E-05	Kcnn3,Mid1,Sox5,Itsn1,Wnt6,Foxn1,Hoxb6,Otx2,Col25a1,Dll3,Lipg,Egln3,Rel,Klhl1,Rhobtb2,Sgca,Hoxa5,Pdgfrb,Gab2,Nr2f2,Dlx1,Adora2a,Atoh1,Ascl3
RORA2_01	17	0,77	5,17E-05	Shh,Mmp14,Apex2,Dtna,Ppp1r3a,Trim29,Hpca,Adcyap1,Epha7,Npas2,Trex2,Tle4,Ap1g2,Sema3a,Esrrb,Khdrbs2,Mylk
EVI1_02	15	0,68	5,24E-05	Esrrg,Hspb3,Il7,Rasgrf2,Spink4,Vamp1,Otx2,Bcl11b,Ptgfr,Crat,Pklr,Evx1,Ndst2,Map4k5,Zfpm2
SMAD_Q6	23	1,05	5,60E-05	Entpd1,Tbp,Gpr3,Bcl2,Cacng2,Sufu,Eln,Nfe2,Bmf,Cacng6,Hpca,Kcna1,Otx2,Adcy8,Rora,Chrm1,Atp6v1e2,Sox21,Eif4g2,Slc8a3,Gprc5d,Lamb3,Evx1
DR4_Q2	22	1,00	6,14E-05	Rasl11b,Adamts4,Nfkbia,Nr5a2,Sox5,Ndst3,Amhr2,Lipg,Hrh3,Sema6c,Adm,Col11a2,Tead3,Dlx1,Stxbp4,Crabp2,Nell2,Tnfrsf12a,Atp1a2,Acy1,Apoc1,Rasgrp2
PBX1_02	16	0,73	6,32E-05	Irx4,Il7,Nog,Gnao1,Col16a1,Otx2,Adcyap1,Tecta,Igf2,Prl,Mtss1,Egr3,Mr1,Tle4,Zfpm2,Pthlh
NCX_01	18	0,82	7,12E-05	Mmp14,Pitpnm2,Cacna2d2,Runx1,Esr1,Spic,Gng4,Nos1,Otx2,Eomes,Hoxa4,Evx1,Bmx,Ptch2,Hoxc6,Kcnd2,Ascl3,Gata4
TTF1_Q6	23	1,05	7,17E-05	Cacng2,Hoxb5,Fgf17,Esr1,Wnt8b,Hoxb6,Ndrg2,Stc2,Adcyap1,Pdha2,Drd2,Nppa,Trex2,Pitpnc1,Wnt7b,Hesx1,Slc35a2,Ror1,Elk4,Dlx1,Sox14,Hoxc6,Crtac1

GABP_B	49	2,23	9,26E-05	Rgs3,Hoxa11,Tbp,Rab2b,Trpv2,Tlr4,Usf1,Zfp64,Tro,Fxyd5,Ppan,Mark1,Lcp2,Iqgap1,Bmf,Diablo,Commd5,Ccr6,Prf1,Erf,Grwd1,Fbxo36,Pcdh7,Loxl3,Crb3,Fcho1,Lamc1,Coro1c,Pdlim2,Polr3d,Il13,Polh,Rps18,Rgs14,Suv39h1,Catsper2,Creb3,Tnfsf11,Gab2,Supt16h,Ddx5,Aak1,Cxcr3,Tead3,Sf3b4,Elk4,Tnni2,Sox10,Ncam1
RFX1_02	22	1,00	1,03E-04	Fxyd1,Rgs6,Dtna,Itpka,Usf1,Dusp3,Itsn1,Gas7,Nos1,Kcnj9,Grwd1,Foxh1,Tsnaxip1,Ttll1,Rem1,Batf,Slc8a3,Kcnip2,Met,Rhobtb2,Nfatc4,Odf1
BACH2_01	24	1,09	1,04E-04	Pacsin3,Gpr3,Gfap,Synpo,Dhrs3,Gadd45b,Sftpc,Hoxb6,Ndrg2,Gkn1,Baz2a,Dnajc5b,Cdkn1a,Slc16a6,Ldb3,Col7a1,Acpp,Bdkrb1,Capn12,Hs3st3b1,Cdh23,Rab3d,Dlg4,Tnfrsf12a
AP1_Q6_01	23	1,05	1,08E-04	Ids,Hoxa11,Esrrg,Pacsin3,Itm2b,Dtna,Flnc,Il1rn,Syngr1,Nrip3,Hspb7,Lamc1,Trim8,Dusp13,Pitpnc1,Usp13,Nfatc4,Epha2,Capn12,Gja1,Pdgfrb,Gab2,Map4k5
HEN1_02	18	0,82	1,18E-04	Phox2a,Esrrg,Cbfa2t3,Capn5,Tbx3,Hoxb5,Doc2b,Adam15,Vamp1,Dll3,Baz2a,Eif4g2,Nr4a3,Crb1,Klhl1,Asb16,Loxl4,Sox14
HSF_Q6	24	1,09	1,30E-04	Ecel1,Mid1,Musk,Foxn1,Gas7,Stc1,Gpr50,Gjb2,Cbx3,Mxd4,Rora,Adcyap1,Chrm1,Cyp46a1,Lhx1,Nr4a3,Kcnip1,Sema6c,Egr3,Six4,Ap1g2,Gad2,Xpo1,Hs6st3
FOXO3_01	21	0,96	1,34E-04	Hs3st1,Dtna,Ntn1,Nxn,Cacng2,Il21,Nfib,Lcp2,Hr,Stc2,Otx2,Aqp2,Trim8,Bcl11b,Dlx2,Sall3,Ap1g2,Sema3a,Pdgfrb,Rbp2,Dll1
PAX5_01	16	0,73	1,40E-04	Pacsin3,Traf4,Flnc,Calcr,Hpca,Angptl2,Cdh16,Rora,Erbb2,Eif4g2,Kcnip1,Ccl20,Gab2,Tcf7l2,Ptch2,Dlx1
MEIS1AHOXA9_01	14	0,64	1,52E-04	Esrrg,Sox4,Irx3,Chodl,Hrk,Nog,Ndst3,Figf,Otx2,Adcy8,Klhl1,Nfatc4,Tle4,Abcc6
PEA3_Q6	21	0,96	1,57E-04	Irak4,Adamts4,Blnk,Ncf2,Mark1,Prf1,Sell,Fgf23,Lamc1,Trim8,Ankrd1,Gpd1,Mr1,Epha2,Gab2,Bcl2l14,Elk4,Fen1,Ptpn6,Tnni2,Ncam1
CP2_02	21	0,96	1,57E-04	Upk2,Traf4,Klhdc3,Cox8c,Trpv2,Rassf1,Irx3,Wnt10b,Nrip2,Dhh,Hoxb6,Kif5a,Trim8,Hcfc1,Bcl11b,Actn1,Pappa,Nfatc4,Ddr1,Ptcra,Pthlh
FXR_Q3	13	0,59	1,60E-04	Atoh7,Il21,Nrip3,Abcg4,Kif5a,Pcdh7,Stc2,Otx2,Mllt6,Lhx1,Mos,Met,Hoxc6
BACH1_01	23	1,05	1,69E-04	Gpr3,Gfap,Il1rn,Syngr1,Mark1,Foxn1,Ndrg2,Gkn1,Lamc1,Mybph,Trim8,Dnajc5b,Cdkn1a,Col7a1,Acpp,Bdkrb1,Lamb3,Capn12,Hs3st3b1,Cdh23,Rab3d,Dlg4,Tnfrsf12a
EGR2_01	18	0,82	1,71E-04	Itpka,Sox4,Runx1,Pcolce,Hrk,Erf,Hoxb6,Ptges,Kcnj1,Mrc2,Gnl1,Gabra1,Kcnip2,Chrd,Egr3,Scn5a,Pdgfrb,Kcnh3
TCF11_01	20	0,91	1,72E-04	Cacng2,Ccr1,Irx5,Satb2,Fbxo36,Kcna1,Lamc1,Itgb6,Elf5,Nr4a3,Dlx3,Sorbs1,Edar,Il24,Pklr,Gab2,Supt16h,Col9a1,Hoxc6,Dll1
OCT1_04	21	0,96	1,98E-04	Hs3st1,Itm2b,Irx4,Dtna,Slc35c2,Mid1,Runx1,Sox5,Gnao1,Ccrl1,Tal2,Bcl11b,Rhobtb2,Sall3,Gab2,Cdh13,Dlx1,Atoh1,Hoxc6,Zfpm2,Ncam1
DR3_Q4	14	0,64	2,14E-04	Myo1e,Adamts4,Cyp24a1,Cacng2,Hspb7,Hpca,Hoxb6,Pcdh7,Trim8,Aqp9,Col12a1,Col11a2,Slc26a3,Xpo1
COMP1_01	14	0,64	2,14E-04	Ppap2a,Nfib,Pde1b,Dhcr24,Slc1a3,Nr4a3,Sall3,Tle4,Pla1a,Tcf7l2,Gnat1,Dlx1,Hoxc6,Ncam1
GATA1_01	21	0,96	2,15E-04	Cacng2,Sufu,Il21,Hoxb5,Actn3,Hr,Il17b,Tob2,Rarg,Rora,Grid2,Lhx1,Bmp7,Slc8a3,Lipg,Suv39h1,Hira,Hs3st3b1,Creb3,Supt16h,Ndst2

TITF1_Q3	21	0,96	2,31E-04	Pdgfra,Bcl2,Il21,Mid1,Jph1,Homer1,Sox5,Gnao1,Wnt8b,Bmp1,Wnt16,Kcnd1,Vax1,Otx2,Tecta,Cldn1,Clcn6,Egfr,Zdhhc14,Mgll,Lrrtm1
PPARA_02	14	0,64	2,39E-04	Esrrg,Hr,Erf,Dhh,Rfx1,Tsnaxip1,Cacna1s,Serpinf2,Hrh3,Hoxa5,Sema3a,Loxl4,Supt16h,Psca
MSX1_01	18	0,82	2,68E-04	Hoxa11,Sox4,Map2k7,Bspry,Gpr12,Nog,Wnt6,Pcdh12,Hoxb4,Epha7,Prdm9,Eomes,Bcar3,Dlx1,Drd3,Hoxc6,Plac1,Dll1
LYF1_01	22	1,00	2,75E-04	Adamts4,Irx4,Tnfrsf19,Aspa,Stc1,Bmp1,Kcnd1,Rarg,Rph3a,Dlx2,Gpd1,S100a9,Epha2,Slc35a2,Pde2a,Pdgfrb,Itga2,Evx1,Cdh13,Tead2,Drd3,Odf1
WHN_B	21	0,96	2,88E-04	Myo1e,Esrrg,Lef1,Tbx3,Irx5,Hoxa7,Tob2,Vax1,Pcdh7,Nfkb2,Stc2,Jph2,Nr4a3,Pappa,Egr3,Camk4,Capn12,Supt16h,Ddx5,Dll4,Hoxc6
AP2_Q6	21	0,96	2,88E-04	Gpr3,Bcl2,Lmx1a,Sufu,Pold3,Hcst,Gas7,Relb,Angptl2,Pitx3,Lhx1,Zdhhc14,Camk4,Col11a2,Cnnm4,Hs3st3b1,Creb3,Ddx5,Tead2,Sdc1,Rasgrp2
EVI1_04	21	0,96	2,88E-04	Dhrs3,Nt5c1b,Mid1,Sox5,Stc1,Rarg,Cyp46a1,Dnajc5b,Sema6a,Bcl11b,Cyp17a1,Prl,Ptgfr,Nfatc4,Wnt2b,Lrrtm1,Cer1,Ptch2,Nr2f2,Atoh1,Hoxc6
E2F1_Q3_01	21	0,96	3,11E-04	Slc12a5,Itpka,Ntn1,Klf13,Satb2,Hr,Dhh,Vax1,Zfp36l2,Kcna1,Mrc2,Tlk2,Mllt6,Rad23a,Nr4a3,Egr3,Nkx6-2,Tcf15,Ror1,Nr2f2,Ncam1
NRSF_01	12	0,55	3,57E-04	Barhl1,Slc12a5,Pou4f3,Lhx3,Cdk5r1,Rph3a,Htr5a,Drd2,Sez6,Kcnip2,Glra3,Drd3
PAX4_01	22	1,00	3,93E-04	Dnm1,Nfkbia,Capn5,Acox2,Klf13,Slc4a1,Hoxa7,Il17b,Hoxb6,Fbxo36,Cdh16,Mxd4,Grik1,Lbx1,Gtf2ird1,Sema3b,Nr4a3,Nfatc4,Xpo1,Evx1,Dll4,Atoh1
AP1_01	22	1,00	4,53E-04	Ids,Arhgap8,Gfap,Dtna,Syngr1,Dhrs3,Nrip3,Mark1,Sftpc,Ly6d,Lamc1,Baz2a,Cdkn1a,Dusp13,Stx17,Usp13,Col7a1,Epha2,Cdh23,Dlx1,Rab3d,Tnfrsf12a
AREB6_04	21	0,96	4,85E-04	Htr2c,Hoxa11,Esrrg,Sox4,Sln,Irx5,Lta,Hr,Bmf,Fzd1,Vax1,Pcdh7,Otx2,Rora,Sla2,Nfatc4,Wnt2b,Slco2a1,Xpo1,Cmklr1,Sox10
FREAC4_01	15	0,68	5,34E-04	Hoxa11,Lef1,Gabrr1,Hr,Rarg,Tecta,Cdkn1a,Cldn18,Ap1g2,Evx1,Ror1,Tcf7l2,Hoxc6,Plunc,Tnmd
AREB6_03	20	0,91	5,55E-04	Hoxa11,Lsp1,Dscaml1,Gna12,Cuedc1,Vgll4,Ngfr,Nfe2,Dsg2,Crb3,Tecta,Chrm1,Slc16a6,Igf2,Gpd1,Klhl1,Ccl20,Mmp16,Pnkp,Khdrbs2
CMYB_01	20	0,91	5,96E-04	Barhl1,Cacng2,Nfya,Nfe2,Hr,Hoxb4,Gnl1,Lhx1,Polh,Mll2,Tpm3,Mgll,Egr3,Scn5a,Phf7,Crat,Ruvbl2,Myog,Dlx1,Ndst2
MYCMAX_02	21	0,96	6,41E-04	Hoxa11,Lef1,Hoxb5,Cd2,Sema7a,Col2a1,Satb2,Diablo,Hoxa7,Hpca,Umps,Psme3,Cdk5r1,Fzd6,Bach2,Xpo1,Dlx1,Oprd1,Sox10,Gata4,Acy1
AR_Q2	13	0,59	6,48E-04	Six3,Pax9,Nfe2,Il16,Grwd1,Hoxb6,Ager,Kcnj1,Fgf23,Adcyap1,Dlx3,Map4k5,Hoxc6
MYOGNF1_01	8	0,36	6,72E-04	Bmf,Hoxb6,Epha7,Sez6,Mtss1,Mycl1,Fgf4,Mylk
E2F_03	19	0,86	6,76E-04	Pdgfra,Klhdc3,Nrip3,Tbx3,Jph1,Mxd3,Kcna6,Pola2,Cdkn1a,Sez6,Eif4g2,Bmp7,Trex2,Suv39h1,Fmo4,Dll4,Glra3,Kcnd2,Pkmyt1
MYOGENIN_Q6	20	0,91	6,81E-04	Sh3bgrl3,Fxyd1,Krt15,Tpcn1,Cacnb1,Heyl,Wnt10b,Col2a1,Erf,Trim8,Ankrd1,Sez6,Eif4g2,Kcnip2,Ldb3,Epha2,Loxl4,Nkx6-2,Dll4,Cyp26a1

PBX1_01	20	0,91	7,31E-04	Hoxa11,Lef1,Nfib,Calcr,Vax1,Pcdh7,Bmpr1b,Aqp9,Sct,Prl,Pappa,Tle4,Six6,Ap1g2,Supt16h,Dlx1,Hnf4g,Ropn1,Zfpm2,Wfdc2
OCT1_07	15	0,68	7,64E-04	Kcnn3,Esrrg,Irx4,Dtna,Fgfr2,Mid1,Runx1,Itsn1,Slc25a13,Nos1,Cntnap4,Bcl11b,Sall3,Adora2a,Atoh1
YY1_01	20	0,91	7,81E-04	Myo1e,Ecel1,Lcp2,Slc25a13,Tal2,Il17b,Prm1,Nln,Tlk2,Gabra1,Srgap2,Actn1,Rab8b,Tpm3,Neto1,Egr3,Scn3a,Dlx1,Sox14,Map4k5
TEL2_Q6	18	0,82	8,24E-04	Adamts4,Rgs3,Fxyd5,Musk,Mark1,Ccl2,Diablo,Slu7,Fcho1,Coro1c,Rgs14,Stat4,Ogg1,Tead3,Sf3b4,Elk4,Sox14,Ptpn6
MAF_Q6	21	0,96	8,83E-04	Pdgfra,Esrrg,Brdt,Bmf,Gas7,Hoxb4,Rora,Tlk2,Coro1c,Slc16a6,Actn1,Mgll,Six4,Aif1,Ogg1,Gab2,Caln1,Supt16h,Cdh13,Map4k5,Hoxc6
MAX_01	20	0,91	8,93E-04	Hoxa11,Brdt,Nrip3,Hoxb5,Hoxa7,Rab3il1,Psme3,Mxd4,Syt3,Cdk5r1,Gata5,Hira,Gja1,Xpo1,Sntb2,Supt16h,Cd164,Fen1,Oprd1,Acy1
IRF2_01	13	0,59	9,61E-04	Usf1,Vgll4,Fxyd5,Pigr,Esr1,Prkd2,Aspa,Ifnb1,Blk,Rel,Sorbs1,Tnfsf13b,Dlx1
DR1_Q3	19	0,86	9,62E-04	Clcnka,Dusp3,Insr,Sox5,Pdzk1,Hoxb6,Nfe2l3,Prrx2,Pcdh7,Prodh2,Tlk2,Erbb2,Nr4a3,Pdlim7,F12,Nr2f2,Ovol1,Crabp2,Fgfr3
LXR_Q3	9	0,41	9,90E-04	Slc2a4,Rasl11b,Phox2a,Blvrb,Nfkbia,Map2k7,Rarg,Acy1,Apoc1
HNF3ALPHA_Q6	18	0,82	1,02E-03	Hoxa11,Pacsin3,Pla2g1b,Irx5,Lcp2,Gnao1,Foxn1,Cyp7a1,Otx2,Syn3,Cdkn1a,Bcl11b,Spdef,Nr4a3,Mgll,Tcf7l2,Ndst2,Atoh1
E2F1_Q6_01	19	0,86	1,02E-03	Map4k1,Tnf,Irx3,Pold3,Jph1,Gadd45b,Actn3,Hr,Fzd1,Gng4,Mxd3,Erf,Dhh,Enpp1,Pola2,Suv39h1,Mtss1,Hira,Pkmyt1
AP2REP_01	16	0,73	1,04E-03	Adamts4,Lcat,Myoz1,Irx4,Dyrk1b,Tacstd2,Jph1,Col16a1,Bmp1,Rora,Cd3g,Chrm1,Jph2,Pde10a,Hoxc6,Ddit4l
AP2_Q6_01	19	0,86	1,09E-03	Fxyd1,Gpr3,Itpka,Klf13,Gpr12,Erf,Rfx1,Angptl2,Cpsf4,Tlk2,Mllt6,Dbn1,Eif4g2,Kif3c,Egr3,Col12a1,Cnnm4,Nr2f2,Rasgrp2
MYCMAX_01	19	0,86	1,17E-03	Hoxa11,Slc12a5,Nrip3,Hoxb5,Col2a1,Satb2,Diablo,Rab3il1,Psme3,Mxd4,Syt3,Chrm1,Pitx3,Mycl1,Hira,Gja1,Oprd1,Sdc1,Acy1
IPF1_Q4	19	0,86	1,25E-03	Atoh7,Sox4,Hoxb5,Hrk,Tal2,Otx2,Aqp9,Bach2,Ppp3cc,Mos,Hesx1,Sall3,Tle4,Entpd5,Cldn14,Nr2f2,Sf3b4,Tnmd,Dll1
SP3_Q3	19	0,86	1,54E-03	Barhl1,Phox2a,Tmod4,Slc12a5,Itpka,Runx1,Mpl,Hoxb6,Ager,Otx2,Bmp7,Zdhhc14,Kcnip2,Nfatc4,Odf3,Scn5a,Mip,Tnni2,Rasgrp2
AHR_Q5	17	0,77	1,56E-03	Runx1,Fbxo36,Nr0b1,Tbx5,Ptf1a,Chrm1,Mst1,Bcl11b,Slitrk1,Pitpnc1,Rel,Gad2,Eya3,Supt16h,Nr2f2,Atoh1,Hoxc6
FOX_Q2	18	0,82	1,56E-03	Hoxa11,Esrrg,Ntn1,Nt5c1b,Gnao1,Foxn1,Vax1,Otx2,Rora,Cdkn1a,Bcl11b,Nr4a3,Klhl1,Mgll,Hesx1,Htr1b,Tcf7l2,Ncam1
PR_01	13	0,59	1,68E-03	Rgs3,Fgf17,Jph1,Wnt8b,Kcnj1,Otx2,Epha7,Tecta,Gipc2,Hoxa5,Ndst2,Hoxc6,Sdc1
ETS1_B	19	0,86	1,86E-03	Adamts4,Rgs3,Flnc,Cbfa2t3,Adam15,Lcp2,Klf13,Dhh,Fbxo36,Kcnd1,Pcdh7,Loxl3,Baz2a,Pdlim2,Slitrk1,Il13,Rgs14,Tnfsf11,Tnni2

SREBP1_02	10	0,45	1,99E-03	Pacsin3,Gfap,Dhrs3,Prkd2,Ndrg2,Inpp4a,Baz2a,Sez6,Chrm3,Ddr1
ARNT_01	19	0,86	2,25E-03	Hoxa11,Lef1,Nrip3,Hoxb5,Jph1,Shmt1,Hoxa7,Rab3il1,Slco1c1,Hoxb4,Psme3,Syt3,Bcl11b,Gata5,Hira,Xpo1,Cd164,Slc35a5,Gata4
LXR_DR4_Q3	9	0,41	2,50E-03	Slc2a4,Adamts4,Blvrb,Nr5a2,Col16a1,Syt3,Col11a2,Nell2,Apoc1
USF2_Q6	18	0,82	3,00E-03	Brdt,Lmx1a,Nrip3,B4galt2,Col2a1,Rab3il1,Hpca,Slco1c1,Rarg,Nkx2-3,Fxyd2,Chrm1,Pitx3,Gpd1,Mycl1,Fen1,Oprd1,Rasgrp2
E2F_Q6_01	17	0,77	3,09E-03	Barhl1,Htr2c,Klhdc3,Tbx3,Mxd3,Pcdh7,Kcna6,Cdkn1a,Bmp7,Polh,Suv39h1,Sorbs1,Fmo4,Dll4,Glra3,Kcnd2,Pkmyt1
MYCMAX_03	18	0,82	3,39E-03	Hoxa11,Lef1,Nrip3,Hoxb5,Shmt1,Hoxa7,Rab3il1,Relb,Slco1c1,Hoxb4,Syt3,Gata5,Bmp7,Dlx2,Gja1,Xpo1,Cd164,Oprd1
AR_01	12	0,55	3,46E-03	Fxyd1,Gnb1l,Slc29a2,Il16,Angptl2,Mxd4,Pitx3,Cdkn1a,Slitrk1,Atp6v0a4,Ndst2,Sox10
ATF_01	19	0,86	3,62E-03	Gpr3,Flnc,Irx3,Nupl2,Gng4,Erf,Rfx1,Ipo11,Adcy8,Adcyap1,Gnl1,Tbx5,Lhx1,Tacr1,Egr3,Epha2,Lrrtm1,Evx1,Drd4
HFH3_01	16	0,73	3,85E-03	Hoxa11,Slc35c2,Tnf,Mid1,Cldn8,Lhx3,Lta,Gnao1,Otx2,Bcl11b,Nr4a3,Hesx1,Htr1b,Tcf7l2,Rbp2,Ncam1
CREB_Q4	18	0,82	4,03E-03	Tex14,Gpr3,Irx4,Kif17,Irx3,Klf13,Syngr3,Erf,Relb,Map3k7,Cbx3,Adcy8,Gnl1,Nr4a3,Egr3,Epha2,Evx1,Osbpl9
E2F_Q3_01	17	0,77	4,21E-03	Map4k1,Tbx3,Jph1,Mxd3,Erf,Pcdh7,Kcna6,Pola2,Bmp7,Suv39h1,Egr3,Tle4,Fmo4,Glra3,Hs6st3,Kcnd2,Pkmyt1
PAX8_B	10	0,45	5,05E-03	Hoxb5,Pou4f3,Hr,Hoxb4,Gabra1,Actn1,Pappa,Supt16h,Tcf7l2,Elk4
E2F_Q3	16	0,73	5,28E-03	Barhl1,Lhx5,Tbx3,Jph1,Shmt1,Mxd3,Kcna1,Adcy8,Lasp1,Pola2,Sez6,Trex2,Fmo4,Hs6st3,Kcnd2,Pkmyt1
CREB_Q2	18	0,82	5,36E-03	Tex14,Irx4,Kif17,Klf13,Gadd45b,Syngr3,Erf,Relb,Map3k7,Cbx3,Adcy8,Mrc2,Gnl1,Nr4a3,Egr3,Epha2,Evx1,Osbpl9
NKX22_01	14	0,64	5,60E-03	Rgs3,Esrrg,Lhx5,Pitpnm2,Esr1,Wnt8b,Myl3,Alb,Chrm1,Smoc2,Cited4,Sall3,Lrrtm1,Glra3
E2F1_Q4	17	0,77	5,65E-03	Hoxa11,Synpo,Nfya,Jph1,Hr,Hoxa7,Zfp36l2,Cbx3,Grik1,Pola2,Trex2,Cit,Fmo4,Osbpl9,Hs6st3,Kcnd2,Pkmyt1
USF_C	19	0,86	6,22E-03	Slc12a5,Sema7a,B4galt2,Adcy3,Col2a1,Satb2,Diablo,Hpca,Nln,Mxd4,Nkx2-3,Syt3,Chrm1,Gpd1,Hoxa4,Mycl1,Camk4,Elk1,Sdc1
ATF6_01	11	0,50	6,93E-03	Lhx5,Flnc,Irx3,Klf13,Gng4,Erf,Kcna6,Lhx1,Sema3b,Egr3,Arf4
USF_02	18	0,82	7,01E-03	Map4k1,Hoxa11,Blnk,Brdt,Nrip3,Hoxb5,Runx1,C4b,Rab3il1,Umps,Stc2,Fzd6,Ube2l6,Sema3a,Xpo1,Cd164,Fen1,Sox10
FXR_IR1_Q6	10	0,45	7,17E-03	Sox5,Gpr12,Vamp1,Erf,Ndrg2,Adcy8,Ovgp1,Npas2,Dlx3,Kcnip2
YY1_Q6	26	1,18	7,46E-03	Ndufs8,Dyrk1b,Usf1,Irx5,Nfya,Gnao1,Slc25a19,Gtpbp1,Rfx1,Vax1,Otx2,Slc26a6,Srgap2,Ptbp1,Hn1,Trim8,Pitx1,Eif4g2,Snrpn,Pmf1,Cd164,Ubc,Abhd1,Gfer,Hoxc6,Slc6a1
NMYC_01	18	0,82	7,75E-03	Barhl1,Bcl2,Slc12a5,Sema7a,Jph1,B4galt2,Adcy3,Col2a1,Gadd45b,Hpca,Gtpbp1,Mxd4,Chrm1,Gpd1,Hrh3,Mycl1,Gja1,Dlx1
SRF_01	7	0,32	7,95E-03	Il17b,Prm1,Hoxb4,Kcnmb1,Actn1,Egr3,Tnmd

CDP_02	10	0,45	8,46E-03	Hoxa11,Tbx3,Gnao1,Hoxa7,Ptf1a,Slc1a3,Gtf2ird1,Sema6a,Ogg1,Hnf4g
PAX8_01	5	0,23	1,02E-02	Hr,Pcdh7,Actn1,Supt16h,Tcf7l2
CDPCR3_01	6	0,27	1,13E-02	Ntn1,Six3,Mark1,Npas1,Hoxa4,Cer1
EVI1_06	4	0,18	1,29E-02	Vamp1,Il17b,Pdha2,Map4k5
MIF1_01	13	0,59	1,59E-02	Tpcn1,Kif17,Dhrs3,Btg4,Sox5,Grwd1,Park2,Grid2,Cars,Gprc5d,Glra3,Ptch2,Mycbp
ZF5_B	16	0,73	1,66E-02	Slc12a5,Pou4f3,B4galt2,Hr,Rasgrp1,Rora,Pitx3,Sema6a,Cacng7,Mycl1,Col11a2,Pde10a,Dll4,Cyp26a1,Fgfr3,Crtac1
GRE_C	10	0,45	1,70E-02	Gnb1l,Fgf17,Jph1,Myl3,Angptl2,Gpd1,Sema3a,Gab2,Prrx1,Hoxc6
E2F1_Q4_01	15	0,68	1,71E-02	Map4k1,Jph1,Mxd3,Erf,Pcdh7,Kcna6,Pola2,Bmp7,Suv39h1,Egr3,Tle4,Fmo4,Glra3,Kcnd2,Pkmyt1
CREBP1_01	12	0,55	1,78E-02	Gabrr2,Rgs6,Ndst3,Wnt8b,Erf,Alb,Inpp4a,Trim8,Hrh3,Rhobtb2,Pthlh,Crtac1
FOXJ2_02	15	0,68	1,79E-02	Esrrg,Nfib,Ccl2,Nog,Satb2,Col10a1,Hoxa7,Rora,Tbx5,Amelx,Dlx2,Klhl1,Hesx1,Sema3a,Pthlh
HOX13_01	5	0,23	1,82E-02	Hoxb5,Trim8,Pde2a,Kcnh3,Prrx1
EGR3_01	7	0,32	1,85E-02	Pcolce,Hrk,Erf,Mrc2,Gnl1,Egr3,Scn5a
E2F_Q4_01	15	0,68	1,87E-02	Map4k1,Tbx3,Jph1,Mxd3,Erf,Pcdh7,Ipo11,Kcna6,Pola2,Bmp7,Suv39h1,Fmo4,Glra3,Kcnd2,Pkmyt1
E2F1_Q3	15	0,68	1,96E-02	Barhl1,Irx3,Tbx3,Pold3,Shmt1,Mxd3,Kcna1,Adcy8,Kcna6,Lasp1,Fmo4,Hira,Hs6st3,Nell2,Pkmyt1
ELK1_02	51	2,32	2,12E-02	Tbp,Klhdc3,Lef1,Slc35c2,Usf1,Cdc37,Irx3,Zfp64,Tro,Ppan,Nfya,Cdca3,Vps18,Diablo,Commd5,Grwd1,Fbxo36,Psme3,Foxh1,Crb3,Lasp1,Coro1c,Cars,Mllt6,Stard3,Cox5b,Tradd,Polr3d,Rad23a,Polh,Mef2b,Rps18,Tpm3,Pex6,Hira,Nthl1,Ogg1,Evx1,Lox l4,Sntb2,Supt16h,Ddx5,Sigirr,Aak1,Sf3b4,Elk4,Slc35a5,Fbxl8,Pthlh,Polr2f,Wfdc2
OCT1_02	15	0,68	2,16E-02	Entpd1,Kcnn3,Esrrg,Dtna,Otos,Runx1,Chml,Stc1,Ptf1a,Ankrd1,Cxcl14,Prl,Sema3a,Dlx1,Col9a1
AR_03	6	0,27	2,23E-02	Jph1,Kcnj1,Cxcl14,Dlx3,Map4k5,Sdc1
PAX5_02	3	0,14	2,25E-02	Coro2a,Usp13,Rasgrp2
RFX1_01	15	0,68	2,25E-02	Ids,Traf4,Tpcn1,Kif17,Anxa1,Bik,Fzd1,Zfp36l2,Park2,Myh8,Cars,Tbx5,Slc8a3,Adm,Prrx1
CREBP1_Q2	15	0,68	2,45E-02	Gpr3,Irx4,Klf13,Diablo,Gng4,Syngr3,Erf,Cbx3,Gnl1,Nr4a3,Tacr1,Egr3,Epha2,Trap1,Osbpl9
ELF1_Q6	15	0,68	2,45E-02	Tlr4,Csf1,Klf13,Iqgap1,Erf,Hoxb4,Kif3c,Rel,Six4,Mr1,Tyrobp,Aif1,Creb3,Supt16h,Ncam1
EVI1_03	6	0,27	2,45E-02	Esrrg,Il7,Spink4,Vamp1,Zfpm2,Plac1
STAT6_02	16	0,73	2,46E-02	Rgs3,Flnc,Pax9,Erf,Dhh,Hoxb4,Adcyap1,Epha7,Mllt6,Pdlim2,Spdef,Pitpnc1,Cited4,Epha2,Pdgfrb,Tcf15
ELK1_01	15	0,68	3,08E-02	Map4k1,Dscaml1,Sox4,Loxl3,Crb3,Plxdc2,Enpp1,Gabra1,Slitrk1,Stat4,Tle4,Epha2,Evx1,Tead3,Tnni2
USF_01	15	0,68	3,21E-02	Hoxa11,Brdt,Nrip3,Hoxb5,Esr2,Hoxa7,Rab3il1,Hoxb4,Rarg,Syt3,Gata5,Xpo1,Cd164,Fen1,Oprd1
STAT1_01	6	0,27	3,24E-02	Arhgap8,Traf4,Gabra1,Sec14l2,Pthlh,Sdc1
E2F_Q2	11	0,50	3,27E-02	Kif17,Usf1,Lmx1a,B4galt2,Gng4,Hpca,Dhcr24,Ptf1a,Tpm3,Mycl1,Mycbp
HTF_01	6	0,27	3,53E-02	Npas2,Bcl11b,Rgs1,Dnm3,Prrx1,Sox14
E2F4DP1_01	14	0,64	3,84E-02	Lhx5,Pold3,Mxd3,Map3k7,Cbx3,Kcna6,Sez6,Sema6a,Suv39h1,Fmo4,Hira,Hs6st3,Nell2,Pkmyt1
CDP_01	7	0,32	3,85E-02	Esrrg,Irx4,Calcr,Alb,Atp6v1b2,Afp,Hoxc6

ATF1_Q6	15	0,68	3,93E-02	Htr2c,Esrrg,Irx4,Dusp3,Erf,Loxl3,Stc2,Kcna6,Mllt6,Lhx1,Adarb2,Hoxa4,Kcnip2,Osbpl9,Tnfrsf12a
AHR_01	6	0,27	4,53E-02	Slc22a8,Angptl2,Kcna6,Chrm1,Supt16h,Ncam1
CREBP1CJUN_01	15	0,68	4,60E-02	Traf4,Tex14,Gpr3,Irx4,Nupl2,Syngr3,Relb,Umps,Adcy8,Gnl1,Ndufb2,Epha2,Trap1,Osbpl9,Ncam1
ATF_B	12	0,55	4,64E-02	Traf4,Tex14,Gpr3,Irx4,Nupl2,Adcy8,Gnl1,Tacr1,Ndufb2,Epha2,Trap1,Osbpl9
CREB_Q2_01	13	0,59	4,78E-02	Traf4,Irx4,Syngr3,Erf,Relb,Cdc42ep4,Gnl1,Pinx1,Pitpnc1,Tacr1,Epha2,Evx1,Nppc
ALX4_01	2	0,09	7,59E-02	Col2a1,Tcf7l2
