# Supplementary material for: Transcriptional profiling reveals functional links between RasGrf1 and Pttg1 in pancreatic beta cells
Source: BMC Genomics. 2014 Nov 25;15:1019. doi: 10.1186/1471-2164-15-1019 (PMC4301450; doi:10.1186/1471-2164-15-1019)
Supplement: Supplementary file 9 — Additional file 9: Table S5B: Transcription factors identified by functional annotation of differentially expressed, induced genes in RasGrf1 KO pancreatic islets. The GeneCodis functional annotation tool tool (http://genecodis.cnb.csic.es/) was used to identify specific subsets within the list of induced genes of RasGrf1 KO pancreatic islets (Additional file 1: Table S1, FDR=0.08; 2230 recognized overexpressed loci, out of a total 2257 probesets listed) that share co-occurrent functional annotations linking them to specific Transcription Factors (TransFac database) at high statistically significant p-values. The “Transcription Factor” column identifies individual transcription factors recognized by GeneCodis as capable of controlling expression of the corresponding groups of loci listed in each case under the column labeled “Genes induced in RasGrf1 KO pancreatic islets”. The column labeled “Gene Count” indicates the specific number of genes identified in each of those groups. Values in the “Percentage” column are calculated referring the “Gene Count” column numbers to the total number of induced, input genes (2198 repressed loci from Additional file 1: Table S1) recognized by the functional annotation software. The column labeled “p-value” refers to the statistical significance of the functional associations identified. (PDF 322 KB) [file 12864_2014_6838_MOESM9_ESM.pdf]

TABLE S5B. Transcription factors identified by functional annotation of differentially expressed, induced genes in RasGrf1 KO pancreatic islets.

The GeneCodis functional annotation tool (http://genecodis.cnb.csic.es/) was used to identify specific subsets within the list of induced genes of RasGrf1 KO pancreatic islets (Additional file 1: Table S1, FDR=0.08; 2230 recognized overexpressed loci, out of a total 2257 probesets listed) that share co-occurrent functional annotations linking them to specific Transcription Factors (TransFac database) at high statistically significant p-values. The "*Transcription Factor*" column identifies individual transcription factors recognized by GeneCodis as capable of controlling expression of the corresponding groups of loci listed in each case under the column labeled "*Genes induced in RasGrf1 KO pancreatic islets*". The column labeled "*Gene Count*" indicates the specific number of genes identified in each of those groups. Values in the "*Percentage*" column are calculated referring the "*Gene Count*" column numbers to the total number of induced, input genes (2230 induced loci from Additional file 1: Table S1) recognized by the functional annotation software. The column labeled "*p-value*" refers to the statistical significance of the functional associations identified.

Transcription factor	***Gene Count***	***Percentage***	***p-Value***	***Genes induced in RasGrf1 KO pancreatic islets***
SP1_Q6	324	14,53	1,12E-84	Calm1,Pabpc4,Gata6,Nefh,Rab28,Nnt,Luc7l2,Elk3,Ssr1,G3bp2,Etv1,Sec24c,Dap3,Slc7a4,Cldn7,Ptpn12,Ywhaz,Wasf2,Hnrpdl,Nup153,Senp2,Pabpc1,Man1a2,Fkbp2,Trrap,Mgat2,Pcnp,Peg3,Gad1,Pcbp2,Rab2b,Adss,Ubap1,Ndufs3,Ywhag,Ncoa6,Nkx2-2,Ganab,Gnb2,Spred2,Fosb,Cpt2,Pank3,Tpt1,Arf1,Atf5,Tpp2,Kpnb1,Sin3a,Khdrbs1,Ppp2r2a,H2afv,Sp4,Foxa3,Cdh2,Prickle1,Rnf44,Tcf4,Mtmr3,Ywhae,Eif5a,Ppap2b,Psmd1,Arih1,Npepps,Cad,Flot2,Herc4,Nosip,Ccnt2,Nf2,Lrrfip2,Npc2,Manba,Map3k3,Keap1,Atp6v0b,Abat,Gabarapl1,Cryzl1,Uchl5,Phtf1,Cdkn1b,Rhoa,Wac,Rabgap1,Stxbp1,Rcn2,Ppp2r5a,Tcta,Rab10,Mapk6,Gtf2i,Flt1,Tjp1,Ttc15,Pafah1b1,Crtap,Iqgap1,Lman1,Cacna1d,Extl2,Vapb,Ap2m1,Gng4,Hmgn1,Kcnb1,Rab11a,Trim28,Atf2,Zcchc7,Lman2,Hivep1,Kpna4,Scgn,Set,Nfyc,Cfl2,Rab3ip,Rab34,Tob2,Atp6ap1,Mafb,Eif2s2,Cpeb4,Itgb1,Myadm,Add1,Ap3d1,Hspd1,Calu,Glo1,Pias1,Elp3,Tra2a,Marcks,Ywhab,Mll5,Topors,Cs,Dusp1,Lasp1,Drap1,Trim25,Tlk2,Eif4b,Denr,Hnf4a,Stat5b,Kpna3,Rad23b,Mapre1,Matr3,Klf5,Xrcc5,Junb,Rab22a,Baz2a,Fbxo3,Sp3,Arhgap6,Wasl,Cbfb,Timp3,Mast2,Lamp2,Psmc5,Purb,Polr1d,Dars,Sec63,Myo18a,Syncrip,Ccng2,Ythdf2,Dnmt3a,Ube2d3,Pnrc1,Trim8,Numa1,Dnaja4,Bcl7c,Smarce1,Anxa4,Enah,Foxa2,Adam17,Gtf2a1,Pgrmc1,Mecp2,Ndel1,Brd2,Poldip3,Jund,Calr,Hdac3,Eno2,Pgrmc2,Eif4g2,Phf8,Neurod1,Sumo3,Srp68,Hmgcs1,Rod1,Atp1b1,Psmb7,Txnrd1,Cbx6,Cbx5,Eps15,Hmg20a,Kif1b,Dpf2,Rapgef4,Epc1,Srebf2,Pck2,Inadl,Kcnip1,Mtmr4,Gtf2e2,P4ha1,Ccni,Creb1,Klhl7,Dnajb2,Ubp1,Map4k3,Strn3,Mtss1,Meis2,Cd2bp2,Rbx1,Ptov1,Gbf1,Serpini1,Csda,Grinl1a,Mdm1,Mnt,Golga3,Ube2n,Rps19,Rnf41,Hspa8,Gphn,Cap1,Cnot7,Man1b1,Polb,Top1,Trim41,Capn7,Tmpo,Herc1,Rpa1,Rnf11,Ptpra,Xpo1,Chka,Slc12a2,Dnajc7,Trpm7,Epn2,Pcm1,Nrd1,Canx,Wdr13,Ctcf,Hhex,Ndrg1,Kctd5,Prkci,Osbp,Smarca2,Mcm7,Cnot4,Vamp2,Atp2a2,Fbxw11,Efemp2,Hdlbp,Fgfr1op2,Slc35a5,Gltscr2,Ap3m1,Appbp2,Hipk1,Rasa1,Rab3d,Cct3,Hbp1,Slbp,Cd2ap,Nfat5,Ogdh,Hs2st1,Cldn4,Cct7,Pura,Fkbp9,Rnf2,Tcf12,Apoa1bp,Fbxl14,Rrbp1,Rbbp6,Vps39,Eif5,Oraov1,Lrpap1,Ncam1,Psip1,Oxr1,Pum1,Akap8,Ireb2,Ogt,Ppm1a,Pak2,Ube2l3,Cirbp,Dlg3

Transcription factor	*Gene Count*	*Percentage*	*p-Value*	*Genes induced in RasGrf1 KO pancreatic islets*
LEF1_Q2	272	12,20	1,48E-58	Fam20c,Csnk1e,Mfn2,Mcart1,Tnks2,Bace1,Syne2,Prkrir,Smad1,Casp2,Elk3,Myo10,Insm1,Etv1,Hdac2,Nek7,Ttc8,Ppp4r1,Ptpn12,Ywhaz,Wasf2,Hnrpdl,Armcx2,Pank1,Mansc1,Gch1,Gad1,Tgfbr1,Nisch,Adss,Ndufs3,Ywhag,Sufu,Eif4ebp2,Glrx,Lifr,Foxp1,Nkx2-2,Ganab,Psme4,Creb3l2,Ckap4,Xpo7,Lmo7,Pank3,Rplp0,Nav2,Smarca4,Kpnb1,Gcnt2,Khdrbs1,Sp4,Bnip3l,Rhob,Cdh2,Rnf44,Tcf4,Clasp1,Ywhae,Kbtbd2,Arih1,Npepps,Rere,Zdhhc2,Nosip,Ptbp2,Myef2,Npc2,Cald1,Phtf1,Chic2,Cyfip2,Taz,Rbm14,Rcn2,Mkrn1,Smarcad1,Rab10,Mapk6,Hectd1,Nono,Gtf2i,Sfpq,Extl2,Trim2,Mxi1,Rbm5,Rab11a,Hivep1,Psmf1,Cdc37l1,Phc1,Elavl4,Ddc,Psmd2,Set,Dhx40,Cfl2,Actr3,Ankrd10,Mafb,Wsb2,Rcn1,Scg3,Ccnl1,Cramp1l,Arid1a,Tra2a,Marcks,Rcor1,Numb,Mll5,Cs,Dusp1,Lasp1,Hdgf,Pax6,Sf3b1,Tlk2,Atp8a1,Fam32a,Stat5b,Mapre1,Xrcc5,Esm1,Brd8,Ube2e2,Rab22a,Ank3,Ptprj,Baz2a,Arhgap6,Rnf14,Cbfb,Lamp2,Adam9,Ywhah,Dars,Sec63,Syncrip,Ccng2,Hibadh,Ythdf2,Dnmt3a,Glud1,Ube2d3,Pnrc1,Bcl7c,Glrx2,Smarce1,Asph,Foxa2,Pcsk2,Gtf2a1,Bach1,Taf10,Slc38a2,Smyd2,Jund,Ahcyl1,Eif4g2,Neurod1,Bet1,Hmgcs1,Elavl1,Atp1b1,Txnrd1,Kif1b,Dpf2,Epc1,Tbc1d8,Srebf2,Ndufs2,Pck2,Ncald,Pitpnc1,Invs,Rbpms,Mgea5,Tpm3,Btg2,Klhl7,Sf1,Pik3r1,Pnrc2,Map4k3,Slc4a2,Meis2,Acsl5,Sec61a1,Fhl1,Cd2bp2,Tia1,Ppp2r5c,Ptov1,Gbf1,Serpini1,Psmb5,Grinl1a,Mdm1,Osbpl2,Dock7,Dapk1,Psmd11,Ap2b1,Nbea,Apbb2,Gna13,Cap1,Cnot7,Oxct1,Bag2,Leprotl1,Top1,Herc1,Rnf11,Enpp2,Robo1,Pbx2,Fech,Pxk,Dnajc7,Pcm1,Ctcf,Arhgap24,Atp1b3,Atrx,Ctnnd1,Ush1c,Bcl9,Dcx,Rb1cc1,Egln2,F2r,Smarca2,Mcm7,Cnot4,Vamp2,Fbxw11,Satb1,Vip,Hbp1,Tes,Mbnl1,Bcl2l11,Dhx30,Abi1,Ubqln1,Rit1,Tnfaip1,Tgfb2,Wdr1,Rock2,Cldn4,Pura,Rnf2,Tcf12,Fbxl14,Anxa6,Rrbp1,Rbbp6,Eif5,Paip1,Psip1,Ets1,Ireb2,Ogt,Chchd7,Cbfa2t2,Dlg3,Mmd,Pnn
MAZ_Q6	231	10,36	6,60E-51	Csnk1e,Nefh,Bace1,Hnrnpa3,Syne2,Ncor1,Luc7l2,Elk3,Insm1,Ssr1,Strn4,G3bp2,Etv1,Sec24c,Acvr1,Bicd1,Ywhaz,Hnrpdl,Senp2,Pabpc1,Fkbp2,Trrap,Pank1,Terf2,Clock,Pcbp2,Ubap1,Ywhag,Sufu,Cdk8,Nkx2-2,Ddit3,Snrk,Spred2,Fosb,Pank3,Etf1,Tpt1,Nav2,Smarca4,Kpnb1,Sin3a,Sp4,Bnip3l,Rhob,Cdh2,Ppp3ca,Rnf44,Ocln,Tcf4,Ywhae,Eif5a,Thrap3,Itpr1,M6pr,Atp1a1,Npepps,Rere,Herc4,Nosip,Map3k3,Cald1,Galnt2,Uchl5,Rhoa,Wac,Mkrn1,Tcta,Rab10,Hectd1,Pafah1b1,Ptprr,Lman1,Mxi1,Smndc1,Atf2,Zcchc7,Mllt10,Phc1,Elavl4,Psmd2,Set,Nfyc,Scn8a,Dhx40,Gabbr1,Actr3,Tob2,Myadm,Ap3d1,Hspd1,Arid1a,Marcks,Ywhab,Mll5,Itm2c,Cs,Lasp1,Gkap1,Pax6,Ewsr1,Trim25,Ccnd2,Tlk2,Denr,Hnf4a,Rad23b,Matr3,Rad21,Junb,Ptprj,Baz2a,Sp3,Cd47,Ankrd17,Lamp2,Zzz3,Polr1d,Dars,Sec63,Syncrip,Ccng2,Dnmt3a,Ube2d3,Pnrc1,Trim8,Gpr56,Ythdf3,Foxa2,Ctdspl,Nrp1,Adam17,Pcsk2,Gtf2a1,Tnpo3,Aldoa,Ldlr,Taf10,Brd2,Ulk1,Hdac3,Pgrmc2,Eif4g2,Dnmt1,Nck1,Aebp2,Cbx6,Hmg20a,Kif1b,Dpf2,Ndufs2,Pitpnc1,Rbpms,Mgea5,P4ha1,Tpm3,Stx6,Ipo4,Amph,Sf1,Map4k3,Slc4a2,Son,Meis2,Serpini1,Arf4,Topbp1,Mnt,Sec24d,Ube2n,Kdelr1,Hspa8,Gphn,Atp2a3,Pja1,Nptx1,Galnt4,Trim41,Dusp6,Slc12a7,Pbx2,Xpo1,Adipor1,Crnkl1,Jarid2,Epn2,Wdr13,Sumo1,Ctcf,Arhgap24,Rhot1,Rock1,Ctnnd1,Bcl9,Ptprf,Dcx,Vamp4,Pak3,Rb1cc1,Ilk,Prkci,Smarca2,Rbp4,Cnot4,Atp2a2,Efemp2,Dpagt1,Satb1,Pafah1b2,Hbp1,Ubqln1,Tgfb2,Rock2,Egr1,Cldn4,Pura,Rnf2,Tcf12,Anxa6,Vldlr,Ncam1,Cd44,Psip1,Chchd7,Ppm1a,Lpp,Ube2l3,Dlg3,Mmd,Pnn,Ctgf
ELK1_02	146	6,55	3,24E-47	Rplp2,Csnk1e,Arpc2,Pabpc4,Tnks2,Bace1,Cox17,Smu1,Elk3,Sec24c,Cldn7,Wasf2,Pabpc1,Rbm22,Psma6,Mgat2,Itgb3bp,Pcbp2,Sec23ip,Cox15,Ube4a,Ddit3,Spred2,Cpt2,Pank3,Tm9sf1,Dguok,Clasp1,Ywhae,Eif5a,Srpr,Herc4,Nosip,Lrrfip2,Phtf1,Agl,Rhoa,Becn1,Dnajc1,Mkrn1,Tcta,Smarcad1,Extl2,Diablo,Zcchc7,Lman2,Sms,Atm,Dhx40,Cpeb4,Pcyt1a,Ube2v2,Pla2g6,Calu,Ppp1r11,Ywhab,Pdap1,Itm2c,Ufd1l,Rnps1,Lasp1,Mki67ip,Ccnd2,Sf3b1,Psmc1,Mapre1,Supt5h,Fbxo3,Ankrd17,Purb,Ythdf2,Tdrd3,Pigc,Ythdf3,Cstf2t,Gtf2a1,Tnpo3,Aldoa,Taf10,Pmpcb,Slc39a6,Dnmt1,Elavl1,Tax1bp1,Psmb7,Hmg20a,Rnf20,Invs,Fbxo38,Mtmr4,Immt,Tpm3,Mtap,Stx6,Ipo4,Map4k3,Son,Meis2,Spg21,Sec61a1,Cd2bp2,Grinl1a,Acp2,Golga3,Arfgap1,Ube2n,Ap3b1,Rnf41,Cap1,Hccs,Leprotl1,Trim41,Galnt10,Nars,Slc12a2,Dnajc7,Rabgef1,Prkag2,Nrd1,Sumo1,Ykt6,Mtmr9,Rb1cc1,Cherp,Kctd5,Mcm7,Scamp2,Atp2a2,Slc35a5,Gltscr2,Uba2,Pafah1b2,Hbp1,Dhx30,Polr1c,Mtmr2,Wdr1,Egr1,Cct7,Rnf2,Nagk,Tsc2,Rbbp6,Ccndbp1,Mtif2,Ube2l3

Transcription factor	*Gene Count*	*Percentage*	*p-Value*	*Genes induced in RasGrf1 KO pancreatic islets*
E4F1_Q6	103	4,62	4,28E-36	Tnks2,Ddx3y,G3bp2,Cldn7,Senp2,Pcnp,Terf2,Nr3c1,Ubap1,Ywhag,Nkx2-2,Gnb2,Fosb,Etf1,E2f5,Tpt1,Ppp2r2a,Rlf,Rnf44,Eif5a,Dock9,Arih1,Srpr,Rere,Rasd1,Cald1,Becn1,Mapk6,Flt1,Pafah1b1,Diablo,Gng4,Gak,Mllt10,Phc1,Sms,Lcmt2,Ppp1r11,Elovl5,Dusp1,Ccnd2,Junb,Ankrd17,Atp6v0d1,Purb,Sec63,Ube2d3,Pnrc1,Dnaja4,Smarce1,Gnb1,Pcsk2,Gtf2a1,Ndel1,Taf10,Poldip3,Slc38a2,Jund,Phf8,Tax1bp1,Atp6v1a,Kif1b,Epc1,Zfr,Ccni,Creb1,Klhl7,Sf1,Ndufa10,Ptov1,Arf4,Dnajc9,Kdelr1,Ikbkb,Polb,Stambp,Anxa7,Ppp1r15a,Adipor1,Sumo1,Ctcf,Pak3,Osbp,Vamp2,Atp2a2,Rasgrf1,Vip,Ubb,Xbp1,Mbnl1,Bcl2l11,Cd2ap,Tnfaip1,Tgfb2,Wdr1,Ogdh,Egr1,Usp48,Rbbp6,Golga5,Chgb,Ppm1a,Pak2
GABP_B	107	4,80	5,08E-35	Mfn2,Cox17,Smu1,Elk3,Sec24c,Ptpn12,Nup153,Rbm22,Pcbp2,Sec23ip,Rab2b,Cox15,Ndufs3,Ube4a,Cdk8,Cdc5l,Foxp1,Ddit3,Gnb2,Cpt2,Etf1,E2f5,Tm9sf1,Dguok,Cdh2,Ppp3ca,Rnf44,Eif5a,Herc4,Agl,Rhoa,Tcta,Flt1,Ttc15,Bclaf1,Iqgap1,Cacna1d,Extl2,Diablo,Lman2,Atm,Cpeb4,Lcmt2,Ube2v2,Ppp1r11,Ywhab,Ufd1l,Elovl5,Rnps1,Pax6,Kif5b,Mapre1,Supt5h,Esm1,Junb,Ankrd17,Cbfb,Syncrip,Ube2d3,Pigc,Ythdf3,Tusc3,Tnpo3,Taf10,Slc39a6,Dnmt1,Rod1,Tax1bp1,Atp6v1a,Psmb7,Inadl,Rnf20,Invs,Mtmr4,Kif3b,Acsl5,Spg21,Cd2bp2,Acp2,Ube2n,Hspa8,Cap1,Ikbkb,Ap3s2,Leprotl1,Trim41,Pik3r4,Galnt10,Dnajc7,Gspt2,Prkag2,Canx,Sumo1,Ykt6,Ndrg1,Cherp,Scamp2,Dnajb9,Pex7,Pafah1b2,Mbnl1,Wdr1,Cct7,Tcf12,Oraov1,Ncam1,Ube2l3
NRF1_Q6	111	4,98	5,30E-35	Mcart1,Arpc2,Tnks2,Prkrir,Casp2,Sec24c,Rabif,Txnl1,Hnrpdl,Pabpc1,Fkbp2,Rnf149,Clock,Ube4a,Sufu,Ndufs6,Cdc5l,Ddit3,Exoc8,Spint2,Atf5,Foxa3,Ppp1r15b,Ywhae,Eif5a,Kbtbd2,Herc4,Rhoa,Wac,Rabgap1,Rbm14,Tcta,Rab10,Tbc1d15,Bclaf1,Golga4,Trim28,Zcchc7,Set,Atm,Rab3ip,Eif5b,Eif2s2,Cog6,Hspd1,Pias1,Topors,Rwdd1,Sdha,Ewsr1,Kif5b,Rad23b,Rad21,Ndrg3,Tollip,Atp6v0d1,Cbfb,Psmc5,Dnajc3,Hibadh,Ube2d3,Pnrc1,Atp5l,Abhd5,Gtf2a1,Mecp2,Trim37,Brd2,Gopc,Pgrmc2,Neurod1,Stk38,Dnmt1,Cbx6,Cbx5,Dpf2,Hint1,Mgea5,Gtf2e2,P4ha1,Ccni,Itch,E2f6,Sf1,Son,Tia1,Arf4,Sae1,Nbea,Top1,Capn7,Dusp6,Ctcf,Cherp,Egln2,Scamp2,Cnot4,Dnajb9,Fgfr1op2,Rasa1,Cct3,Plekha1,Nudt11,Sec14l1,Abi1,Nfat5,Nudt4,Pdcd6ip,Akap8,Ppm1a,Cirbp
FOXO4_01	180	8,07	3,93E-32	Gata6,Nap1l1,Tnks2,Nnt,Rreb1,Scrn3,Smad1,G3bp2,Etv1,Colec12,Acvr1,Cldn7,Bicd1,Ptpn12,Gch1,Nr3c1,Pcbp2,Stard5,Adss,Mospd2,Ncoa6,Eif4ebp2,Glrx,Foxp1,Dner,Nkx2-2,Psme4,Ckap4,Fosb,Xpo7,Elf1,Smarca4,Arf1,H2afv,Sp4,Rhob,Tcf4,Ppap2b,Kbtbd2,Itpr1,Dock9,Sh3glb1,Rasd1,Map3k3,Gabarapl1,Nap1l5,Cdkn1b,Agl,Rabgap1,Taz,Fxr1,Rab10,Flt1,Tjp1,Riok3,Mxi1,Rbm5,Hivep1,Elavl4,Cfl2,Hhip,Gne,Wsb2,Cpeb4,Scg3,Ppm1b,Gns,Pik3c2a,Pias1,Marcks,Rcor1,Mll5,Grsf1,Dusp1,Hdgf,Pax6,Stat5b,Rad21,Klf5,Esm1,Brd8,Ank3,Arhgap6,Cbfb,Zzz3,Myo18a,Ccng2,Hibadh,Wbp2,Pnrc1,Trim8,Ythdf3,Foxa2,Atf6,Nrp1,Pcsk2,Gtf2a1,Tnpo3,Pgrmc1,Blcap,Cpeb3,Arid4b,Slc38a2,Ulk1,Cnot2,Atp6v1a,Ubl3,Cbx6,Dicer1,Nsf,Inadl,Ncald,Pitpnc1,Clk4,Btg2,Stx6,Pik3r1,Meis2,Sst,Ptov1,Sqstm1,Calb1,Cyb561,Osbpl2,Hccs,Nfe2l2,Nptx1,Top1,Dusp6,Enpp2,Robo1,Mapk8,Pxk,Jarid2,Epn2,Prkag2,Sumo1,Rock1,Asxl1,Ctnnd1,Bcl9,Ulk2,Hhex,Znrf2,Dcx,Vdr,Pak3,Ndrg1,Prkci,Smarca2,Mcm7,Atp2a2,Slmap,Ppp3r1,Satb1,Appbp2,Hbp1,Srpk2,Stx18,Nudt11,Mbnl1,Bcl2l11,Dhx30,Polr1c,Mtmr2,Ubqln1,Rit1,Tgfb2,Shoc2,Egr1,Pura,Vldlr,Ncam1,Pum1,Ets1,Ireb2,Ogt,Chchd7,Lpp,Cbfa2t2
YY1_Q6	74	3,32	2,97E-30	Arpc2,Tnks2,Rab28,Syne2,Ncor1,Elk3,Strn4,Etv1,Sfxn1,Dap3,Peg3,Sec23a,Ubap1,Ncoa6,Foxp1,Arf1,Ppp2r2a,Ppp1r15b,Tcf4,Mtmr3,Ywhae,Kpna6,Cdkn1b,Rab10,Pafah1b1,Csnk1a1,Sfpq,Rbm5,Elavl4,Nfyc,Gtpbp1,Cpeb4,Ap3d1,Pias1,Tra2a,Mll5,Ccnd2,Rad21,Ndrg3,Rab22a,Wasl,Psmc5,Racgap1,Trim8,Irak1,Clk2,Eif4g2,Aebp2,Cbx5,Kif1b,Rapgef4,Epc1,Nsf,Map3k4,Clk4,Pnrc2,Map4k3,Strn3,Tia1,Gbf1,Psmb5,Mnt,Hspa8,Nbea,Jarid2,Wdr13,Ctcf,Atrx,Ubc,Snx5,Tardbp,Eif5,Pum1,Akap8
MYC_Q2	114	5,11	5,59E-30	Mcart1,Pabpc4,Hnrnpa3,Ncl,Insm1,Ssr1,Suclg2,Etv1,Hnrpdl,Nup153,Pabpc1,Armcx2,Sec23ip,Adss,Polr3c,Ncoa6,Nkx2-2,Gnb2,Pank3,Rlf,Rnf44,Tcf4,Kbtbd2,Sumf1,Tbl1x,Cad,Atp6v0b,Rabgap1,Fxr1,Tbc1d15,Cacna1d,Diablo,Zcchc7,Set,Hhip,Nfx1,Gtpbp1,Cpeb4,Napa,Ap3d1,Pla2g6,Hspd1,Topors,Elovl5,Dusp1,Pax6,Ewsr1,Eif4b,Bfar,Ankrd17,Zzz3,Syncrip,Dnmt3a,Wbp2,Bcl7c,Gtf2a1,Aldoa,Asna1,Trim37,Brd2,Slc38a2,Neurod1,Atp6v1a,Cbx6,Cbx5,Epc1,Ddx18,Mtap,Stx6,Ipo4,Spg21,Csda,Mnt,Sae1,Ctbp2,Rps19,Gna13,Nptx1,Top1,Rpa1,Xpo1,Trpm7,Canx,Atp1b3,Mpp3,Bcl9,Plagl1,Ptprf,Hhex,Znrf2,Egln2,Ilk,Cnot4,Dpagt1,Dnajb9,Slc35a5,Ap3m1,Hbp1,Alg1,Nudt11,Mbnl1,Tgfb2,Pdcd6ip,Rnf146,Mthfd1,Snx5,Anxa6,Lamp1,Tsc2,Rbbp6,Armcx1,Vldlr,Ogt,Ppm1a

Transcription factor	*Gene Count*	*Percentage*	*p-Value*	*Genes induced in RasGrf1 KO pancreatic islets*
NFY_Q6_01	123	5,52	1,94E-28	Luc7l2,Casp2,Insm1,G3bp2,Phpt1,Ywhaz,Wasf2,Spc25,Peg3,Sufu,Eif4ebp2,Mapk1,Ganab,Ddit3,Spred2,Fosb,Cox6a2,Etf1,Nav2,Tpp2,Kpnb1,Rhob,Rnf44,M6pr,Dock9,Nf2,Map3k3,Abat,Rhoa,Rbm14,Ppp2r5a,Tcta,Nup88,Gtf2i,Gca,Atf2,Dstn,Elavl4,Kpna4,Ddc,Nfyc,Atm,Gtpbp1,Cpeb4,Ppm1b,Piga,Elp4,Pla2g6,Calu,Marcks,Numb,Mll5,Rpn2,Ufd1l,Elovl5,Pax6,Kif5b,Fam32a,Brd8,Junb,Ank3,Rplp1,Lamp2,Ccng2,Wbp2,Ube2d3,Racgap1,Dnajb11,Dld,Mecp2,Cpeb3,Ivd,Eif4g2,Vcp,Hmgcs1,Atp1b1,Cbx5,Kif1b,Srebf2,Iars,Rps9,Invs,Mtmr4,Sfrp5,Sf1,Pik3r1,Pnrc2,Ubp1,Son,Ppp2r5c,Fads1,Gbf1,Gphn,Abca7,Xpo1,Jarid2,Nrd1,Sumo1,Bcl9,Dcx,Pak3,Hist3h2a,Osbp,Mcm7,Anapc5,Atp2a2,Cct3,Ubb,Xbp1,Tes,Mbnl1,Ogdh,Cct7,Anxa6,Vldlr,Ncam1,Akap8,Ppm1a,Cbfa2t2,Cirbp,Dlg3,Mmd,Pnn
NFAT_Q4_01	156	7,00	3,18E-24	Calm1,Ndufc1,Gata6,Syne2,Ddx3y,Rreb1,Scrn3,Elk3,Ncl,Etv1,Stx7,Colec12,Lpl,Man1a2,Gad1,Nr3c1,Pcbp2,Mospd2,Cdk8,Sytl4,Cdc5l,Foxp1,Nkx2-2,Gcnt2,Rhob,Clcn3,Tcf4,Itpr1,Dock9,Tbl1x,Npepps,Rere,Cad,Ccnt2,Cald1,Phtf1,Nap1l5,Rhoa,Cyfip2,Wac,Taz,Abcb7,Stxbp1,Tcta,Mapk6,Nono,Sfpq,Extl2,Mxi1,Rab11a,Hivep1,Dstn,Elavl4,Ddc,Dhx40,Rab3ip,Tob2,Gtpbp1,Cpeb4,Ppm1b,Ptp4a2,Ace2,Ap3d1,Calu,Tra2a,Rcor1,Numb,Itm2c,Rps10,Pax6,Trim25,Klf5,Arhgap6,Wasl,Mast2,Adam9,Syncrip,Racgap1,Trim8,Smarce1,Asph,Lims1,Abhd5,Blcap,Mecp2,Cpeb3,Trim37,Slc38a2,Smyd2,Pgrmc2,Vcp,Aig1,Atp1b1,Aebp2,Atp6v1a,Ubl3,Dicer1,Dpf2,N4bp1,Epc1,Tbc1d8,Ndufs2,Dnajc13,Cul3,Pitpnc1,Gtf2e2,Ccni,Tpm3,Creb1,Amph,Map4k3,Strn3,Slc4a2,Mtss1,Ptov1,Serpini1,Psmb5,Calb1,Slk,Gas2,Smpd1,Rnf41,Anp32e,Vamp3,Enpp2,Ptpra,Scamp1,Xpo1,Jarid2,Prkag2,Nrd1,Atp1b3,Ctnnd1,Bcl9,Pak3,Smarca2,Serpinh1,Atp2a2,Gdap1,Xbp1,Srpk2,Mbnl1,Aco1,Rit1,Tnfaip1,Tgfb2,Nfat5,Tcf12,Fbxl14,Rrbp1,Psip1,Ets1,Mtif2,Ogt,Chchd7,Ctgf
TCF11MAFG_01	64	2,87	1,88E-22	Ndufc1,Mcart1,Ssr1,Txnl1,Itm2b,Rabep1,Psma6,Sec23a,Ywhag,Foxp1,Tbl1x,Npepps,Lrrfip2,Gabarapl1,Nap1l5,Rab10,Pafah1b1,Iqgap1,Cdc37l1,Cfl2,Eef2,Pcyt1a,Ufd1l,Psmd7,Baz2a,Psmc5,Gtf2a1,Aldoa,Pgrmc1,Bach1,Brd2,Vcp,Atp1b1,Txnrd1,Cbx6,Hint1,Mtmr4,Ero1l,Tpm3,Meis2,Sqstm1,Arf4,Mnt,Sec24d,Psmd11,Anp32e,Atp2a3,Leprotl1,Abca7,Pbx2,Dnajc7,Jarid2,Ctnnd1,Osbp,Smarca2,Satb1,Hdlbp,Rasgrf1,Nudt11,Aco1,Cd2ap,Eif5,Cirbp,Dlg3
MEIS1_01	93	4,17	1,29E-21	Gata6,Bace1,Elk3,Etv1,Dap3,Psma6,Gad1,Ncoa6,Sufu,Ddit3,Ckap4,Xpo7,Nav2,Ppp3ca,Prickle1,Mtmr3,Camsap1,Eif5a,Psmd1,Cnot6l,Npepps,Nf2,Traf3,Map3k3,Cald1,Dnajc1,Fxr1,Pafah1b1,Extl2,Riok3,Rbm5,Ap2m1,Golga4,Elavl4,Kpna4,Ddc,Scgn,Arpc1a,Pias1,Elovl5,Pax6,Tlk2,Klf5,Baz2a,Cd47,Rnf14,Atp6v0d1,Dars,Dnaja4,Nrp1,Abhd5,Taf10,Cnot2,Hmgcs1,Dnmt1,Cbx6,Kif1b,Map1lc3b,Srebf2,Ncald,Stx6,Itch,Ubp1,Strn3,Mtss1,Meis2,Rbx1,Ppp2r5c,Gbf1,Usp20,Gphn,Gna13,Top1,Enpp2,Xpo1,Pcp4,Slc12a2,Crnkl1,Bcl9,Pak3,Osbp,Smarca2,Vil1,Cnot4,Vip,Srpk2,Mbnl1,Cct7,Pura,Vldlr,Ncam1,Psip1,Ets1
PAX4_03	122	5,47	4,69E-21	Arpc2,Gata6,Cryba2,Luc7l2,Elk3,Acvr1,Ptpn12,Ywhaz,Wasf2,Gch1,Nr3c1,Tgfbr1,Ywhag,Gnb2,Xpo7,Kpnb1,Sp4,Tcf4,Clasp1,Ywhae,Eif5a,Ppap2b,Thrap3,Cdkn1b,Rhoa,Wac,Taz,Ppp2r5a,Tcta,Rab10,Flt1,Pafah1b1,Iqgap1,Cacna1d,Mxi1,Rab11a,Atf2,Mllt10,Elavl4,Atp5b,Scn8a,Tob2,Itpkb,Ppm1b,Add1,Pias1,Mll5,Cs,Lasp1,Hdgf,Pax6,Ccnd2,Kif5b,Hnf4a,Rad23b,Mthfr,Klf5,Folr1,Baz2a,Fbxo3,Arhgap6,Wasl,Cbfb,Mast2,Myo18a,Syncrip,Ythdf2,Ube2d3,Trim8,Anxa4,Ythdf3,Foxa2,Pcsk2,Brd4,Tnpo3,Brd2,Slc39a6,Eno2,Eif4g2,Atp6v1a,Cbx6,Epc1,Srebf2,Ncald,Pitpnc1,Mgea5,Tpm3,Map4k3,Strn3,Mtss1,Meis2,Fhl1,Cd2bp2,Sqstm1,Serpini1,Csda,Mnt,Pip5k1b,Pja1,Trim41,Anxa7,Rnf11,Dnajc7,Wdr13,Ctcf,Rock1,Ctnnd1,Ptprf,Hhex,Dcx,Egln2,Smarca2,Cnot4,Fgfr1op2,Rasa1,Rab3d,Hbp1,Mbnl1,Abi1,Ogdh,Rac1,Dlg3
ETS2_B	108	4,84	1,64E-20	Arpc2,Tnks2,Cox17,Elk3,Myo10,Sec24c,Dap3,Lifr,Foxp1,Etf1,E2f5,Tpp2,Kpnb1,Gcnt2,Clcn3,Eif5a,Itpr1,M6pr,Srpr,Traf3,Nfkb1,Cald1,Galnt2,Cdkn1b,Chic2,Rab10,Flnb,Flt1,Pafah1b1,Gorasp2,Cacna1d,Extl2,Rab11a,Zcchc7,Mllt10,Phc1,Psap,Elavl4,Scn8a,Actr3,Itgb1,Pdap1,Pax6,Trim25,Stat5b,Esm1,Junb,Baz2a,Sp3,Ywhah,Polr1d,Myo18a,Tusc3,Nrp1,Calr,Aig1,Dnmt1,Nck1,Ndufs2,Ncald,Pitpnc1,Lin7c,Tpm3,Sf1,Pik3r1,Slc4a2,Meis2,Acsl5,Sec61a1,Fhl1,Dctn2,Ppp2r5c,Serpini1,Arf4,Cyb561,Ap2b1,Cap1,Ikbkb,Atp2a3,Trim41,Pik3r4,Enpp2,Robo1,Pten,Prkag2,Canx,Ctcf,Hhex,Znrf2,Dcx,Pak3,Fbxw11,Vip,Plekha1,Mbnl1,Cd2ap,Nfat5,Egr1,Pura,Tcf12,Fbxl14,Vldlr,Vps39,Psip1,Pum1,Ets1,Rac1,Ube2l3

Transcription factor	*Gene Count*	*Percentage*	*p-Value*	*Genes induced in RasGrf1 KO pancreatic islets*
E12_Q6	174	7,80	4,20E-20	Calm1,Pabpc4,Nefh,Tnks2,Bace1,Surf4,Syne2,Ddx3y,Rreb1,Ncor1,Elk3,Strn4,G3bp2,Etv1,Hdac2,Colec12,Cldn7,Lpl,Ywhaz,Trim23,Armcx2,Rabep1,Mansc1,Gch1,Terf2,Gad1,Map2k1,Nr3c1,Nmt2,Ncoa6,Gnb2,Spred2,Fosb,Cox6a2,Spint2,Nav2,Atf5,Sin3a,Ppp2r2a,Foxa3,Cdh2,Ocln,Eif5a,Cnot6l,Npepps,Zdhhc2,Abhd4,Keap1,Atp6v0b,Galnt2,Uchl5,Agl,Taz,Abcb7,Stxbp1,Nup88,Flnb,Gtf2i,Tjp1,Iqgap1,Gca,Gorasp2,Cacna1d,Golga4,Kcnb1,Atf2,Hivep1,Dstn,Psmf1,Elavl4,Sms,Atm,Dhx40,Hhip,Pla2g6,Pias1,Arid1a,Hdgf,Pax6,Tlk2,Kif5b,Hnf4a,Mthfr,Gcg,Rab22a,Ank3,Ptprj,Wasl,Atp6v0d1,Polr1d,Myo18a,Syncrip,Ythdf2,Dnmt3a,Wbp2,Ube2d3,Trim8,Atf6,Ugcg,Aldoa,Ttc13,Cpeb3,Trim37,Brd2,B4galt6,Ahcyl1,Hdac3,Aig1,Neurod1,Hmgcs1,Atp1b1,Cbx6,N4bp1,Rapgef4,Tbc1d8,Srebf2,Zfr,Kcnip1,Invs,Map3k4,Mtmr4,Tpm3,Pik3r1,Map4k3,Strn3,Mtss1,Meis2,Sqstm1,Serpini1,Csda,Calb1,Mdm1,Osbpl2,Dapk1,Pip5k1b,Nbea,Gna13,Nptx1,Trim41,Dusp6,Enpp2,Pcp4,Prkag2,Pcm1,Canx,Arhgap24,Rock1,Asxl1,Ctnnd1,Bcl9,Egln1,Vdr,Pak3,Clic4,Smarca2,Mcm7,Scamp2,Cnot4,Vamp2,Fbxw11,Hdlbp,Ap3m1,Cd2ap,Tnfaip1,Wdr1,Usp48,Nagk,Armcx1,Ncam1,Psip1,Oxr1,Chchd7,Pak2,Macf1
ATF3_Q6	71	3,18	3,32E-19	Calm1,Insm1,G3bp2,Cldn7,Itm2b,Ywhaz,Senp2,Peg3,Ubap1,Fosb,Ppp2r2a,Bnip3l,Rnf44,Tbl1x,Arih1,Srpr,Gabarapl1,Dnajc1,Flt1,Tjp1,Pafah1b1,Cfl2,Hhip,Atp6ap1,Eef2,Thoc1,Mll5,Elovl5,Cs,Dusp1,Mapre1,Esm1,Rnf14,Pnrc1,Trim8,Pcsk2,Ndel1,Arid4b,Jund,Ncald,Dnajc13,Pitpnc1,Ccni,Tpm3,Mtss1,Ndufa10,Ap2a2,Sst,Ppp2r5c,Slk,Ikbkb,Top1,Herc1,Rnf11,Ppp1r15a,Ctnnd1,Pak3,Fbxw11,Vip,Hipk1,Plekha1,Tes,Mbnl1,Ubqln1,Cd2ap,Tnfaip1,Ogdh,Usp48,Chgb,Ppm1a,Cbfa2t2
AP1_C	105	4,71	4,38E-19	Mcart1,Nefh,Tnks2,Ids,Cryba2,Elk3,Itm2b,Ywhaz,Wasf2,Ywhag,Psme4,Ddit3,Syp,Arf1,Ppp2r2a,Rnf44,Psmd1,Cnot6l,Tbl1x,Arih1,Npepps,Suox,Rere,Lrrfip2,Abhd4,Traf3,Gabarapl1,Pafah1b1,Csnk1a1,Ptprr,Ap2m1,Dstn,Kpna4,Psmd2,Dhx40,Rab34,Ywhab,Pdap1,Ewsr1,Trim25,Tlk2,Psmd7,Stat5b,Klf5,Ank3,Baz2a,Mast2,Trim8,Gpr56,Asph,Aldoa,Bach1,Mecp2,Irak1,Brd2,Slc38a2,Ulk1,Hdac3,Eno2,Aig1,Atp1b1,Atp6v1a,N4bp1,Zfr,Pitpnc1,Mrps23,Klhl7,Ctnnal1,Strn3,Ap2a2,Dctn2,Sqstm1,Mnt,Sec24d,Psmd11,Bag2,Anxa7,Scamp1,Ppp1r15a,Nrd1,Asxl1,Mpp3,Dcx,Vdr,Rb1cc1,Osbp,Smarca2,Vil1,Rbp4,Vamp2,Atp2a2,Fbxw11,Rab3d,Srpk2,Nudt11,Ubqln1,Rit1,Rock2,Cldn4,Tcf12,Pkm2,Macf1,Lpp,Cbfa2t2,Cirbp
FREAC2_01	92	4,13	1,01E-18	Nnt,Rreb1,Scrn3,Smad1,Elk3,Cpn1,Etv1,Colec12,Wasf2,Pank1,Nr3c1,Eif4ebp2,Lifr,Gnb2,Xpo7,Tpp2,Gcnt2,Sin3a,Ppp3ca,Ocln,Tcf4,Ppap2b,Kbtbd2,Cad,Rasd1,Cald1,Nap1l5,Cdkn1b,Taz,Trim2,Atf2,Dstn,Mbtps1,Dhx40,Cfl2,Hhip,Gnpnat1,Pik3c2a,Pias1,Marcks,Mll5,Dusp1,Lasp1,Tlk2,Rad21,Brd8,Junb,Hibadh,Pnrc1,Trim8,Gtf2a1,Blcap,Cpeb3,Slc38a2,Ulk1,Phf8,Neurod1,Matn2,Rod1,Ubl3,Nap1l3,Ing1,Pitpnc1,Mgea5,Cd2bp2,Sqstm1,Pja1,Enpp2,Robo1,Scamp1,Pxk,Wdr13,Sumo1,Ctcf,Bcl9,Egln1,Ndrg1,Smarca2,Mcm7,Hipk1,Hbp1,Srpk2,Mbnl1,Cd2ap,Zranb1,Pura,Tcf12,Ets1,Chchd7,Lpp,Cbfa2t2,Ctgf
CREB_02	43	1,93	5,33E-18	Cldn7,Ywhaz,Slc35e1,Spred2,Fosb,Tpt1,Ppp2r2a,Rnf44,Eif5a,Arih1,Smarcad1,Flt1,Pafah1b1,Sms,Dhx40,Hhip,Eef2,Thoc1,Napa,Dusp1,Ube2d3,Ythdf3,Pcsk2,Gtf2a1,Poldip3,Jund,Phf8,Srebf2,Ccni,Sec24d,Kdelr1,Cnot7,Polb,Ppp1r15a,Pak3,Osbp,Vamp2,Cd2ap,Tnfaip1,Usp48,Armcx1,Chgb,Ppm1a
ATF_01	44	1,97	1,17E-17	Luc7l2,Cldn7,Peg3,Terf2,Nkx2-2,Spred2,Fosb,Etf1,Bnip3l,Clcn3,Rnf44,Srpr,Cald1,Pafah1b1,Gng4,Gak,Hhip,Marcks,Elovl5,Dusp1,Pnrc1,Dnajb11,Gtf2a1,Ndel1,Jund,Elavl1,Ncald,Dnajb2,Son,Sec61a1,Sst,Ikbkb,Stambp,Nptx1,Top1,Ppp1r15a,Hdlbp,Vip,Mbnl1,Cd2ap,Ogdh,Usp48,Chgb,Ppm1a
SOX9_B1	61	2,74	3,23E-17	Rreb1,G3bp2,Etv1,Sec24c,Ptpn12,Ywhaz,Pank1,Clock,Pcbp2,Mospd2,Eif4ebp2,Cdk8,Nkx2-2,Ilf2,Tcf4,Rere,Herc4,Map3k3,Cald1,Cdkn1b,Trim2,Hmgn1,Phc1,Elavl4,Dhx40,Cfl2,Rcn1,Arid1a,Marcks,Kpna3,Ank3,Myo18a,Pnrc1,Trim8,Pcsk2,Elavl1,Kif1b,N4bp1,Gtf2e2,Tpm3,Itch,Mtss1,Tia1,Arf4,Polb,Top1,Dusp6,Hhex,Dcx,Clic4,Pafah1b2,Plekha1,Bcl2l11,Vldlr,Ncam1,Psip1,Snapc3,Pum1,Ets1,Pak2,Cbfa2t2
SREBP1_01	59	2,65	1,88E-15	Hnrnpa3,Rabep1,Polr3c,Foxp1,Ddit3,Wdfy1,Gnb2,Aars,Npepps,Manba,Nap1l5,Smndc1,Atf2,Hivep1,Psap,Set,Atp6ap1,Eef2,Napa,Ap3d1,Topors,Cs,Eif4b,Supv3l1,Tollip,Rab22a,Rnf14,Ankrd17,Atp6v0d1,Ythdf3,Blcap,Asna1,Stk16,Ahcyl1,Calr,Atp6v1a,Cbx6,Epc1,Stx6,Acp2,Sec24d,Sae1,Oxct1,Xpo1,Trpm7,Pcm1,Canx,Ctcf,Atrx,Ptprf,Rasgrf1,Slc35a5,Alg1,Ubqln1,Pdcd6ip,Pura,Rnf146,Lamp1,Dlg3

Transcription factor	*Gene Count*	*Percentage*	*p-Value*	*Genes induced in RasGrf1 KO pancreatic islets*
NFE2_01	47	2,11	5,92E-15	Mfn2,Mcart1,Nefh,Ids,Cryba2,Myo10,Itm2b,Rabep1,Map2k1,Ywhag,Clasp1,Psmd1,Tbl1x,Lrrfip2,Abhd4,Traf3,Gabarapl1,Gaa,Flt1,Ptprr,Psmd2,Psmd7,Ndrg3,Baz2a,Mast2,Aldoa,Bach1,Irak1,Hdac3,N4bp1,Rbpms,Dctn2,Sqstm1,Mnt,Sec24d,Psmd11,Bag2,Anxa7,Pcp4,Nrd1,Vdr,Rb1cc1,Fbxw11,Rab3d,Nudt11,Rit1,Cd44
ERR1_Q2	93	4,17	7,13E-15	Rreb1,Pank1,Ywhag,Ncoa6,Lifr,Dner,Ddit3,Rnf44,Gosr1,Abat,Gabarapl1,Taz,Vdac1,Rab10,Tbc1d15,Extl2,Ap2m1,Golga4,Elavl4,Atp5b,Gtpbp1,Ppm1b,Ace2,Myadm,Hspd1,Calu,Mll5,Cs,Pax6,Kif5b,Mapre1,Rab22a,Ank3,Arhgap6,Rnf14,Adam9,Ywhah,Pla2g12a,Hibadh,Ythdf2,Dnmt3a,Glud1,Ube2d3,Pnrc1,Rps6ka2,Ythdf3,Kcnmb2,Atf6,Nrp1,Pcsk2,Aldoa,Blcap,Ahcyl1,Elavl1,Atp1b1,Atp6v1a,Ubl3,Cbx6,Rbpms,Immt,Tpm3,Btg2,Kif3b,Amph,Map4k3,Meis2,Gabpa,Sec61a1,Fhl1,Dctn2,Gbf1,Dlat,Mnt,Smpd1,Gna13,Hipk2,Pex26,Jarid2,Canx,Arhgap24,Rhot1,Asxl1,Bcl9,Rb1cc1,Clic4,Vamp2,Satb1,Srpk2,Zranb1,Cldn4,Rnf2,Rrbp1,Psip1
AP1_Q6_01	40	1,79	1,25E-14	Ids,Elk3,Itm2b,Psme4,Ddit3,Lrrfip2,Abhd4,Gabarapl1,Csnk1a1,Ptprr,Psmd2,Gabbr1,Pdap1,Psmd7,Stat5b,Ank3,Trim8,Aldoa,Irak1,Brd2,Atp6v1a,Pitpnc1,Ctnnal1,Ap2a2,Dctn2,Sqstm1,Sec24d,Anxa7,Scamp1,Ppp1r15a,Nrd1,Rb1cc1,Smarca2,Vil1,Fbxw11,Srpk2,Nudt11,Ubqln1,Rock2,Lpp
MAF_Q6	39	1,75	1,40E-13	Tgfbr1,Cdk8,Cdc5l,Nkx2-2,Creb3l2,Kpnb1,Rnf44,Itpr1,Flot2,Chic2,Flt1,Ap2m1,Lman2,Phc1,Elavl4,Itpkb,Ttc3,Numb,Pax6,Tlk2,Esm1,Sp3,Lamp2,Syncrip,Pnrc1,Tusc3,Aldoa,Pgrmc2,Hmgcs1,Rod1,Eps15,Slc4a2,Meis2,Ppp2r5c,Rassf3,Cnot7,Vamp3,Satb1,Nfat5
AHR_Q5	34	1,52	1,53E-13	Tnks2,Ywhaz,Hnrpdl,Trim23,Nr3c1,Pcbp2,Mospd2,Ddit3,Fosb,Pank3,Spint2,Cdkn1b,Rhoa,Wac,Tcta,Rab10,Vapb,Mllt10,Dstn,Elavl4,Set,Dhx40,Asph,Mecp2,Cbx6,Pitpnc1,Immt,Sf1,Meis2,Mnt,Fgfr1op2,Sec14l1,Vldlr,Cirbp
PU1_Q6	62	2,78	1,74E-13	Hnrnpa3,Rreb1,Strn4,Etv1,Ywhaz,Wasf2,Pabpc1,Map2k1,Rab2b,Cdk8,Cdc5l,Nkx2-2,Spred2,Etf1,Nav2,Prickle1,Cnot6l,Rhoa,Tcta,Cacna1d,Mllt10,Sms,Tob2,Cpeb4,Scg3,Arid1a,Mll5,Elovl5,Pax6,Esm1,Ptprj,Lamp2,Dnmt3a,Ube2d3,Gpr56,Nrp1,Aldoa,Rod1,Nck1,Tbc1d8,Tpm3,Meis2,Dlat,Ctbp2,Atp2a3,Galnt4,Tmpo,Jarid2,Atp1b3,Rock1,Ctnnd1,Hhex,Egln2,Vip,Dhx30,Abi1,Nfat5,Cct7,Tcf12,Ncam1,Psip1,Zfp161
AP2_Q6	37	1,66	1,96E-13	Gata6,Insm1,Pabpc1,Terf2,Sufu,Cdk8,Ube2s,Sp4,Zdhhc2,Wac,Rab10,Mapk6,Trim28,Rab3ip,Mafb,Arpc1a,Cpeb4,Myadm,Arid1a,Mapre1,Rab22a,Sp3,Crbn,Mast2,Zzz3,Dnmt3a,Cbx6,Meis2,Mnt,Galnt10,Ctcf,Efemp2,Hdlbp,Rasa1,Usp48,Rrbp1,Mmd
NRF2_Q4	37	1,66	2,29E-13	Ids,Cryba2,Itm2b,Rabep1,Map2k1,Ywhag,Psme4,Psmd1,Tbl1x,Lrrfip2,Abhd4,Pafah1b1,Psmd2,Pias1,Psmd7,Baz2a,Mast2,Psmc5,Gtf2a1,Aldoa,Bach1,Brd2,Slc38a2,Vcp,Atp1b1,Txnrd1,Pitpnc1,Dctn2,Sqstm1,Mnt,Sec24d,Bag2,Canx,Rb1cc1,Nudt11,Cd44,Ube2l3
CREB_Q2_01	34	1,52	3,83E-13	Cldn7,Senp2,Peg3,Ube4a,Spred2,Fosb,Tpt1,Ppp2r2a,Clcn3,Rnf44,Cald1,Flt1,Pafah1b1,Hhip,Thoc1,Dusp1,Ccnd2,Esm1,Syncrip,Pnrc1,Gtf2a1,Stk16,Jund,Srebf2,Pitpnc1,Cnot7,Ikbkb,Nptx1,Ppp1r15a,Appbp2,Cd2ap,Egr1,Chgb,Ppm1a
TATA_01	102	4,57	4,17E-13	Csnk1e,Arpc2,Pabpc4,Nefh,Cryba2,Syne2,Nnt,Ncor1,Etv1,Lpl,Bicd1,Pank1,Gch1,Itgb3bp,Peg3,Foxp1,Rplp0,Elf1,Ppp2r2a,Ube2s,Foxa3,Tulp4,Tcf4,Itpr1,Npc2,Cald1,Rhoa,Dnajc1,Tcta,Flt1,Trim2,Ece2,Dstn,Psap,Scgn,Scn8a,Cfl2,Actr3,Eef2,Wsb2,Cpeb4,Prom1,Pias1,Rcor1,Ttr,Mapre1,Gcg,Esm1,Junb,Ank3,Crbn,Zzz3,Glud1,Dnaja4,Gtf2a1,Blcap,Bach1,Irak1,Calr,Aig1,Hmgcs1,Pum2,Rapgef4,Nap1l3,Usp47,Ing1,Ncald,Cul3,Pex3,Invs,P4ha1,Tpm3,Ctnnal1,Meis2,Rps19,Nptx1,Tmpo,Herc1,Enpp2,Mapk8,Ppp1r15a,Tm4sf4,Dnajc7,Jarid2,Trpm7,Ctcf,Dcx,Serpinh1,Rbp4,Fbxw11,Hdlbp,Vip,Plekha1,Tes,Rit1,Tgfb2,Zranb1,Pura,Tcf12,Snx5,Chchd7,Ctgf
CREB_Q2	37	1,66	4,38E-13	Smad1,Cldn7,Senp2,Pabpc1,Nr3c1,Fosb,Etf1,Bnip3l,Clcn3,Rnf44,Arih1,Pafah1b1,Gak,Sms,Hhip,Pdap1,Elovl5,Dusp1,Adam9,Pnrc1,Gtf2a1,Elavl1,Srebf2,Dnajb2,Ndufa10,Sst,Dnajc9,Ikbkb,Nptx1,Ppp1r15a,Ctcf,Hhex,Pak3,Cd2ap,Ogdh,Egr1,Chgb
ARNT_02	36	1,61	4,40E-13	Ncl,Insm1,Sec23ip,Polr3c,Gnb2,Rnf44,Kbtbd2,Rabgap1,Smndc1,Nfx1,Cpeb4,Topors,Eif4b,Ankrd17,Syncrip,Asna1,Brd2,Atp6v1a,Cbx6,Cbx5,Epc1,Stx6,Spg21,Sae1,Nptx1,Trpm7,Canx,Ptprf,Egln2,Slc35a5,Alg1,Pdcd6ip,Pura,Rnf146,Lamp1,Vldlr
USF_02	37	1,66	1,01E-12	Ssr1,Etv1,Pabpc1,Sec23ip,Ncoa6,Ddit3,Gnb2,Rlf,Kbtbd2,Sumf1,Hivep1,Nfx1,Cpeb4,Topors,Dusp1,Eif4b,Supv3l1,Dnmt3a,Gtf2a1,Asna1,Stk16,Calr,Neurod1,Atp6v1a,Cbx5,Epc1,Pik3r1,Sqstm1,Mdm1,Nptx1,Xpo1,Egln2,Tgfb2,Tcf12,Mthfd1,Vldlr,Ppm1a

Transcription factor	*Gene Count*	*Percentage*	*p-Value*	*Genes induced in RasGrf1 KO pancreatic islets*
AP4_Q5	107	4,80	1,33E-12	Ndufc1,Pja2,Etv1,Colec12,Acvr1,Ywhaz,Hnrpdl,Bace2,Pcbp2,Foxp1,Nkx2-2,Wdfy1,Gnb2,Cox6a2,Etf1,Ppp3ca,Prickle1,Tcf4,Dock9,Nosip,Abhd4,Manba,Traf3,Map3k3,Nap1l5,Cdkn1b,Taz,Dnajc1,Fxr1,Mxi1,Set,Cpeb4,Elp4,Myadm,Ap3d1,Arid1a,Pdap1,Dusp1,Ccnd2,Denr,Stat5b,Supt5h,Klf5,Tollip,Baz2a,Cd47,Rnf14,Atp6v0d1,Polr1d,Myo18a,Ythdf2,Pvrl3,Ube2d3,Trim8,Brd4,Bach1,Cpeb3,Eif4g2,Atp6v1a,Cbx6,Kif1b,Dpf2,Mtmr4,Tpm3,Btg2,Amph,Smarcal1,Slc4a2,Mtss1,Meis2,Spg21,Ppp2r5c,Arf4,Nbea,Apbb2,Ryk,Vamp3,Ptk2,Slc12a2,Epn2,Arhgap24,P2rx4,Rock1,Trip11,Prpf39,Hhex,Dcx,Pak3,Ndrg1,Smarca2,Appbp2,Pex7,Hbp1,Xbp1,Mbnl1,Rit1,Tgfb2,Zranb1,Egr1,Pura,Tcf12,Anxa6,Psip1,Ets1,Mtif2,Ppm1a,Cbfa2t2
ARNT_01	36	1,61	1,70E-12	Etv1,Adss,Nkx2-2,Rlf,Rnf44,Kbtbd2,Atp6v0b,Tbc1d15,Zcchc7,Cpeb4,Hspd1,Pax6,Eif4b,Syncrip,Dnmt3a,Brd2,Neurod1,Atp6v1a,Cbx5,Epc1,Stx6,Mnt,Nptx1,Rpa1,Xpo1,Trpm7,Egln2,Dnajb9,Slc35a5,Mbnl1,Tgfb2,Snx5,Lamp1,Rbbp6,Vldlr,Pak2
CEBP_Q3	36	1,61	1,70E-12	Syne2,Ssr1,Mospd2,Ddit3,Rhob,Tbl1x,Herc4,Lrrfip2,Map3k3,Cald1,Chic2,Mxi1,Elavl4,Cfl2,Rab3ip,Hhip,Kpna3,Capza2,Junb,Ube2e2,Syncrip,Usp9x,Brd2,Calr,Pum2,Meis2,Acsl5,Mnt,Nfe2l2,Pten,Jarid2,Tgfb2,Pura,Tcf12,Rrbp1,Ncam1
HLF_01	36	1,61	2,30E-12	Csnk1e,Calm1,Hnrnpa3,Pcbp2,Ywhag,Cdk8,Foxp1,Ddit3,Xpo7,Prickle1,Clasp1,Zdhhc2,Smarcad1,Elavl4,Eef2,Pdap1,Ube2e2,Fbxo3,Trim8,Foxa2,Usp9x,Pcsk2,Mecp2,Neurod1,Srebf2,Ing1,Klhl7,Pik3r1,Meis2,Nptx1,Top1,Robo1,Epn2,Sumo1,Hist3h2a,Clic4
ATF4_Q2	36	1,61	2,30E-12	Smad1,Itm2b,Pabpc1,Cdc5l,Psme4,Fosb,Prickle1,Rnf44,Smarcad1,Pafah1b1,Ap2m1,Pdap1,Elovl5,Dusp1,Psmd7,Baz2a,Pnrc1,Gpr56,Gtf2a1,Aldoa,Slc38a2,Phf8,Srebf2,Ndufa10,Ikbkb,Nptx1,Leprotl1,Xpo1,Ppp1r15a,Ctcf,Hhex,Pak3,Ogdh,Usp48,Lpp,Cirbp
YY1_02	33	1,48	2,49E-12	Tnks2,Ncor1,Strn4,Dap3,Peg3,Arf1,Tcf4,Ywhae,Ptbp2,Rab10,Nfyc,Eef2,Ap3d1,Mll5,Rad21,Ndrg3,Wasl,Zzz3,Gtf2a1,Ubl3,Epc1,Map3k4,E2f6,Strn3,Tia1,Glg1,Gbf1,Hspa8,Wdr13,Ctcf,Fbxo9,Mtmr2,Pum1
NFMUE1_Q6	34	1,52	2,51E-12	Tnks2,Ncor1,Strn4,Dap3,Arf1,Ppp1r15b,Tcf4,Ptbp2,Clk1,Bclaf1,Sfpq,Riok3,Rbm5,Nfyc,Ap3d1,Rab22a,Wasl,Ube2d3,Cstf2t,Irak1,Eif4g2,Phf8,Cbx5,Epc1,E2f6,Sf1,Tia1,Gbf1,Psmb5,Dnajc7,Vamp2,Usp48,Eif5,Rac1
CREBP1CJUN_01	36	1,61	2,55E-12	G3bp2,Sec24c,Cldn7,Ywhaz,Senp2,Peg3,Ppp2r2a,Bnip3l,Clcn3,Rnf44,Arih1,Flt1,Pafah1b1,Phc1,Hhip,Eef2,Thoc1,Elovl5,Dusp1,Syncrip,Pnrc1,Jund,Elavl1,Ncald,Ndufa10,Sst,Ikbkb,Ppp1r15a,Cd2ap,Tnfaip1,Ogdh,Egr1,Usp48,Ncam1,Chgb,Ppm1a
USF_01	35	1,57	2,68E-12	Hnrnpa3,Ssr1,Etv1,Polr3c,Gnb2,Rlf,Kbtbd2,Sumf1,Zcchc7,Set,Nfx1,Topors,Dusp1,Eif4b,Dnmt3a,Gtf2a1,Trim37,Brd2,Slc38a2,Neurod1,Atp6v1a,Cbx6,Epc1,Stx6,Sae1,Gna13,Nptx1,Rpa1,Xpo1,Alg1,Rnf146,Snx5,Lamp1,Rbbp6,Ppm1a
HSF_Q6	38	1,70	2,76E-12	Gata6,Ywhag,Foxp1,Nkx2-2,Creb3l2,Fosb,Sin3a,Ppp2r2a,Ywhae,Map3k3,Pafah1b1,Atf2,Hspd1,Ank3,Ankrd17,Gtf2a1,Tnpo3,Cct8,Taf10,Atp6v1a,Dpf2,Jak1,Kcnip1,P4ha1,Ptov1,Hspa8,Gphn,Ptpra,Robo1,Xpo1,Canx,Serpinh1,Cct3,Ubb,Ubqln1,Nudt4,Cct7,Pura
MYOD_Q6	81	3,63	3,29E-12	Calm1,Nefh,Tnks2,Surf4,Etv1,Acvr1,Cldn7,Ywhaz,Hnrpdl,Trim23,Mansc1,Stard5,Xpo7,Etf1,Spint2,Ppp3ca,Clcn3,Prickle1,Ywhae,Tbl1x,Npepps,Galnt2,Cdkn1b,Cyfip2,Taz,Nup88,Tjp1,Elavl4,Atp5b,Scn8a,Elp4,Mll5,Pdap1,Hdgf,Ccnd2,Tlk2,Ptprj,Baz2a,Cd47,Ankrd17,Myo18a,Ythdf2,Dnmt3a,Ube2d3,Adcy6,Pcsk2,Brd4,Eif4g2,Neurod1,Atp1b1,Kif1b,Srebf2,Invs,Strn3,Spg21,Nbea,Gphn,Apbb2,Ryk,Xpo1,Prkag2,Pcm1,Arhgap24,Rock1,Bcl9,Hhex,Egln1,Vdr,Pak3,Atp2a2,Fbxw11,Srpk2,Mbnl1,Cd2ap,Zranb1,Pura,Tcf12,Ncam1,Psip1,Oxr1,Ppm1a
HNF3_Q6	72	3,23	3,30E-12	Gata6,Syne2,Rreb1,Cpn1,Acvr1,Itm2b,Rnf149,Tgfbr1,Ywhag,Foxp1,Nkx2-2,Fosb,Aldh9a1,Gcnt2,Ppp2r2a,H2afv,Sp4,Rnf44,Tcf4,Dock9,Rere,Cald1,Gabarapl1,Tjp1,Ptprr,Mxi1,Elavl4,Itpkb,Tm7sf3,Scg3,Pik3c2a,Pias1,Dusp1,Hdgf,Hnf4a,Stat5b,Rad21,Klf5,Junb,Cbfb,Wbp2,Pnrc1,Foxa2,Pcsk2,Gtf2a1,Cpeb3,Trim37,Ulk1,C8b,Neurod1,Cbx6,Pum2,Meis2,Osbpl2,Dusp6,Jarid2,Epn2,Ctcf,Asxl1,Smarca2,Atp2a2,Fbxw11,Srpk2,Dhx30,Abi1,Pura,Rnf146,Pitrm1,Ncam1,Ogt,Chchd7,Ctgf

Transcription factor	*Gene Count*	*Percentage*	*p-Value*	*Genes induced in RasGrf1 KO pancreatic islets*
CREB_Q4	35	1,57	3,52E-12	Smad1,G3bp2,Cldn7,Senp2,Pabpc1,Fosb,Ppp2r2a,Bnip3l,Clcn3,Rnf44,Pafah1b1,Gak,Hhip,Thoc1,Elovl5,Dusp1,Ankrd17,Pnrc1,Gtf2a1,Slc38a2,Elavl1,Srebf2,Ndufa10,Sst,Dnajc9,Ikbkb,Nptx1,Ppp1r15a,Ctcf,Hhex,Pak3,Cd2ap,Ogdh,Chgb,Ppm1a
YY1_01	35	1,57	4,10E-12	Luc7l2,Dap3,Ppp2r2a,Rnf44,Tcf4,Ywhae,Traf3,Atp6v0b,Cald1,Rhoa,Taz,Tcta,Pafah1b1,Dstn,Mll5,Tlk2,Matr3,Rab22a,Ankrd17,Zzz3,Syncrip,Gtf2a1,Phf8,Rod1,Kif1b,Epc1,Tpm3,Ppp2r5c,Gbf1,Ctcf,Fbxw11,Dpagt1,Pex7,Chchd7,Dlg3
AP1_Q6	36	1,61	4,28E-12	Mcart1,Nefh,Ids,Cryba2,Elk3,Ywhaz,Psme4,Lrrfip2,Abhd4,Traf3,Dstn,Psmd2,Pdap1,Psmd7,Stat5b,Baz2a,Trim8,Aldoa,Hdac3,Atp1b1,Pitpnc1,Ap2a2,Dctn2,Psmd11,Ap2b1,Anxa7,Vdr,Rb1cc1,Smarca2,Fbxw11,Rab3d,Ubqln1,Rit1,Rock2,Tcf12,Lpp
CREBP1_Q2	34	1,52	4,30E-12	Smad1,Cldn7,Senp2,Peg3,Ubap1,Fosb,Ppp2r2a,Thrap3,Flt1,Pafah1b1,Diablo,Gng4,Gak,Hhip,Eef2,Napa,Elovl5,Dusp1,Ccnd2,Adam9,Pnrc1,Gtf2a1,Slc38a2,Elavl1,Ncald,Ikbkb,Nptx1,Ppp1r15a,Hhex,Mbnl1,Cd2ap,Egr1,Chgb,Ppm1a
MAX_01	35	1,57	5,47E-12	Ssr1,Suclg2,Etv1,Pabpc1,Adss,Gnb2,Rlf,Tcf4,Kbtbd2,Sumf1,Cad,Zcchc7,Nfx1,Cpeb4,Topors,Dusp1,Eif4b,Syncrip,Dnmt3a,Asna1,Slc38a2,Neurod1,Atp6v1a,Cbx5,Epc1,Nptx1,Top1,Xpo1,Hhex,Hbp1,Tgfb2,Mthfd1,Snx5,Lamp1,Ppm1a
CHX10_01	71	3,18	6,10E-12	Syne2,Rreb1,Smad1,Suclg2,Etv1,Stx7,Bace2,Itgb3bp,Ubap1,Lgals12,Nkx2-2,Gnb2,Fosb,Sgpp1,Ocln,Eif5a,Ppap2b,Cald1,Cyfip2,Mllt10,Elavl4,Hhip,Gtpbp1,Cpeb4,Ace2,Elp4,Marcks,Cs,Hdgf,Pax6,Stat5b,Folr1,Arhgap6,Adam9,Ccng2,Enah,Lims1,Nrp1,Pcsk2,Pgrmc1,Arl2bp,Bach1,Slc39a6,Clk2,Calr,Aig1,Neurod1,Myst4,Pitpnc1,Invs,Mtss1,Mnt,Gas2,Gna13,Pja1,Capn7,Robo1,Pbx2,Canx,Ctnnd1,Dcx,Smarca2,Atp2a2,Zbed3,Srpk2,Mbnl1,Cldn4,Pura,Actr10,Vldlr,Ogt
MYCMAX_03	34	1,52	9,62E-12	Pabpc4,Hnrnpa3,Ssr1,Pabpc1,Sec23ip,Adss,Rlf,Kbtbd2,Tbc1d15,Zcchc7,Nfx1,Topors,Pax6,Eif4b,Ankrd17,Syncrip,Dnmt3a,Gtf2a1,Trim37,Brd2,Slc38a2,Neurod1,Cbx6,Cbx5,Epc1,Nptx1,Rpa1,Xpo1,Plagl1,Tgfb2,Rnf146,Snx5,Lamp1,Ppm1a
MEF2_02	69	3,09	1,03E-11	Prkrir,Etv1,Spc25,Pank1,Itgb3bp,Pcbp2,Ywhag,Lifr,Foxp1,Fosb,Cox6a2,Tpp2,Ppp2r2a,Sp4,Clasp1,Ssrp1,Wac,Fxr1,Crtap,Mxi1,Hivep1,Elavl4,Cpeb4,Cog6,Scg3,Ppm1b,Rcor1,Mll5,Kpna3,Klf5,Junb,Ndrg3,Sp3,Dnajc3,Asph,Irak1,Ndel1,Atp6v1a,Myst4,Cul3,Mrps23,P4ha1,Creb1,Ipo4,Smarcal1,Dnajb2,Glg1,Nbea,Atp2a3,Top1,Vamp3,Enpp2,Epn2,Ctnnd1,Plagl1,Smarca2,Fbxw11,Slmap,Rasgrf1,Hipk1,Hbp1,Bcl2l11,Nfat5,Rnf146,Ncam1,Ets1,Hipk3,Ppm1a,Ctgf
ZF5_B	34	1,52	1,10E-11	Insm1,Clock,Ncoa6,Ddit3,Etf1,Nav2,Ppp3ca,Mtmr3,Nf2,Rbm14,Rab10,Psmd2,Nfyc,Hhip,Thoc1,Marcks,Mthfd2,Mapre1,Junb,Syncrip,Rps6ka2,Foxa2,Mecp2,Vcp,Cbx6,Epc1,Srebf2,Rbpms,Mtmr4,Slc4a2,Meis2,Gphn,Wdr1,Ppm1a
AP1_Q4	36	1,61	1,93E-11	Mcart1,Nefh,Cryba2,Elk3,Rabep1,Map2k1,Lifr,Lrrfip2,Abhd4,Ptprr,Golga4,Dstn,Pdap1,Stat5b,Rad23b,Baz2a,Trim8,Bach1,Irak1,Hdac3,Pitpnc1,Ap2a2,Dctn2,Psmd11,Ap2b1,Bag2,Anxa7,Vdr,Rb1cc1,Smarca2,Fbxw11,Srpk2,Ubqln1,Rock2,Tcf12,Lpp
EGR_Q6	36	1,61	2,21E-11	Insm1,Gad1,Adss,Ywhag,Nkx2-2,Kpnb1,Sp4,Cdh2,Ppp3ca,Rnf44,Ttc15,Lman1,Cacna1d,Kcnb1,Nfyc,Calu,Matr3,Sec63,Ythdf2,Pcsk2,Brd2,Calr,Pgrmc2,Mnt,Gphn,Anp32e,Nrd1,Ctcf,Hhex,Ilk,Vamp2,Efemp2,Bcl2l11,Egr1,Tsc2,Vldlr
ATF6_01	24	1,08	2,46E-11	Tnks2,Armcx2,Sec23a,Slc35e1,Nkx2-2,Tpt1,Elf1,Gng4,Phc1,Dhx40,Syncrip,Brd2,Jund,Vcp,Dnajb2,Ptov1,Gbf1,Arf4,Sec24d,Kdelr1,Nptx1,Top1,Osbp,Egr1
CREB_01	34	1,52	2,81E-11	G3bp2,Cldn7,Ywhaz,Senp2,Peg3,Fosb,Dguok,Ppp2r2a,Bnip3l,Rnf44,Arih1,Flt1,Pafah1b1,Hhip,Eef2,Thoc1,Elovl5,Dusp1,Pnrc1,Jund,Elavl1,Srebf2,Ncald,Ndufa10,Sst,Ikbkb,Ppp1r15a,Appbp2,Cd2ap,Tnfaip1,Ogdh,Usp48,Chgb,Ppm1a
CMYB_01	33	1,48	4,31E-11	Hnrnpa3,Dap3,Ywhaz,Pabpc1,Atp2b2,Gnb2,Spint2,Atf5,Kpnb1,Wac,Snrpd1,Rbm14,Pafah1b1,Mxi1,Mllt10,Itpkb,Cpeb4,Klf5,Trim37,Jund,Aebp2,Cbx6,Epc1,Tpm3,Pnrc2,Csda,Sae1,Ap2b1,Canx,Rock1,Dhx30,Pura,Rbbp6
BACH1_01	35	1,57	5,21E-11	Elk3,Wasf2,Psme4,Ddit3,Syp,Abhd4,Traf3,Gabarapl1,Ptprr,Cfl2,Rab34,Psmd7,Mast2,Trim8,Gpr56,Aldoa,Mecp2,Brd2,Hdac3,Eno2,Atp1b1,Ctnnal1,Ap2a2,Dctn2,Sqstm1,Mnt,Sec24d,Psmd11,Bag2,Ppp1r15a,Rb1cc1,Osbp,Rab3d,Srpk2,Lpp
ATF1_Q6	33	1,48	1,10E-10	Cldn7,Ywhaz,Sec23a,Foxp1,Ddit3,Creb3l2,Spred2,Fosb,Dguok,Bnip3l,Rnf44,Cald1,Flt1,Pafah1b1,Cfl2,Hhip,Eef2,Elovl5,Rnps1,Jund,Ncald,Ccni,Sst,Ppp2r5c,Ikbkb,Top1,Fbxw11,Vip,Plekha1,Cd2ap,Ogdh,Usp48,Chgb

Transcription factor	*Gene Count*	*Percentage*	*p-Value*	*Genes induced in RasGrf1 KO pancreatic islets*
ZF5_01	32	1,43	1,99E-10	Calm1,Pabpc4,Fkbp2,Ddit3,Tpp2,Kpnb1,Rlf,Map3k3,Drap1,Tlk2,Stat5b,Junb,Ythdf2,Trim8,Bcl7c,Gnb1,Elavl1,Aebp2,Kif1b,Nsf,Ube2n,Cap1,Abcc8,Trpm7,Ctcf,Ilk,Rasgrf1,Hipk1,Sec14l1,Hs2st1,Usp48,Lamp1
PAX3_B	29	1,30	2,67E-10	Nnt,Pcnp,Eif5a,Rasd1,Stxbp1,Hmgn1,Hivep1,Elovl5,Junb,Zzz3,Pnrc1,Trim8,Smarce1,Ndel1,Slc38a2,Cbx6,Epc1,Ap2b1,Ikbkb,Polb,Xpo1,Canx,Ctcf,Xbp1,Egr1,Pura,Rnf146,Chgb,Pak2
EGR1_01	33	1,48	2,68E-10	Hnrpdl,Adss,Gnb2,Rhob,Cdh2,Ywhae,Eif5a,Cnot6l,Rhoa,Tcta,Pafah1b1,Hmgn1,Kcnb1,Nfyc,Scn8a,Matr3,Pcsk2,Brd2,Srebf2,Map3k4,Sf1,Mnt,Gphn,Pja1,Ctcf,Hhex,Egln2,Vamp2,Efemp2,Gltscr2,Sec14l1,Egr1,Rbbp6
ATF_B	28	1,26	2,80E-10	Ywhaz,Peg3,Spred2,Fosb,Ppp2r2a,Rnf44,Flt1,Pafah1b1,Hhip,Eef2,Elovl5,Dusp1,Pnrc1,Jund,Elavl1,Ncald,Sst,Ikbkb,Ppp1r15a,Pak3,Appbp2,Vip,Mbnl1,Cd2ap,Tnfaip1,Ogdh,Chgb,Ppm1a
VDR_Q6	32	1,43	3,00E-10	Ppp4r1,Itm2b,Pabpc1,Clock,Ddit3,Gnb2,Kpnb1,Mapk6,Ccnl1,Pax6,Lamp2,Syncrip,Dnmt3a,Tusc3,Kif1b,Rapgef4,Ndufs2,Ccni,Stx6,Pik3r1,Cd2bp2,Mnt,Tmpo,Rnf11,Xpo1,Nrd1,Rock1,Asxl1,Hhex,Rock2,Tcf12,Rbbp6
E2F1_Q3_01	32	1,43	3,41E-10	Calm1,Elk3,Insm1,Fkbp2,Slc25a11,Gnb2,Kpnb1,Eif5a,Nono,Elavl4,Psmd2,Marcks,Mll5,Tlk2,Junb,Polr1d,Syncrip,Ythdf2,Gtf2a1,Tnpo3,Dnmt1,Meis2,Topbp1,Dnajc9,Vamp3,Dusp6,Trpm7,Fbxo9,Mcm7,Satb1,Eif5,Ncam1
SMAD4_Q6	31	1,39	3,42E-10	Calm1,Wasf2,Clock,Gnb2,Ppp3ca,Tcf4,Ptbp2,Traf3,Cald1,Ap2m1,Diablo,Dhx40,Mafb,Dach2,Arid1a,Mll5,Elovl5,Psmc5,Trim8,Phf8,Epc1,Creb1,Meis2,Sst,Wdr13,Hhex,Znrf2,Vdr,Pak3,Atp2a2,Pura
CREB_Q4_01	29	1,30	4,18E-10	Cldn7,Ywhaz,Senp2,Creb3l2,Spred2,Fosb,Tpt1,Ppp2r2a,Rnf44,Arih1,Pafah1b1,Hhip,Thoc1,Elovl5,Dusp1,Gtf2a1,Jund,Srebf2,Ccni,Sst,Ikbkb,Nptx1,Ppp1r15a,Cd2ap,Tnfaip1,Ogdh,Usp48,Chgb,Ppm1a
NGFIC_01	31	1,39	4,49E-10	Luc7l2,G3bp2,Hnrpdl,Pabpc1,Fkbp2,Adss,Etf1,Sin3a,Rhob,Cdh2,Eif5a,Cnot6l,Kcnb1,Nfyc,Lasp1,Matr3,Syncrip,Pcsk2,Srebf2,Map3k4,Mnt,Pja1,Nrd1,Dcx,Egln2,Efemp2,Nudt11,Sec14l1,Egr1,Rrbp1,Rbbp6
NRF2_01	29	1,30	6,64E-10	Tnks2,Sec24c,Cox15,Ddit3,Cpt2,Eif5a,Herc4,Agl,Dnajc1,Iqgap1,Diablo,Cpeb4,Itm2c,Rnps1,Rps10,Fbxo3,Dnmt1,Hmg20a,Mtmr4,Cd2bp2,Acp2,Ube2n,Cap1,Dnajc7,Cherp,Scamp2,Fgfr1op2,Pafah1b2,Mtmr2
CEBPB_01	33	1,48	6,66E-10	Csnk1e,Etv1,Sars,Mospd2,Lgals12,Foxp1,Ddit3,Pank3,Etf1,Bnip3l,Rhob,Tbl1x,Flot2,Cfl2,Ace2,Ccnl1,Marcks,Rcor1,Pdap1,Dusp1,Klf5,Syncrip,Usp9x,Aldoa,Klhl7,Ctnnal1,Pik3r1,Mtss1,Acsl5,Vamp3,Mbnl1,Fbxl14,Ppm1a
GATA1_01	31	1,39	6,86E-10	Clock,Atp2b2,Sufu,Kpnb1,Ppp2r2a,Ywhae,Rere,Map3k3,Wac,Rcn2,Rab10,Rbm5,Kpna4,Nfyc,Tob2,Ank3,Sp3,Ankrd17,Syncrip,Gnb1,Tnpo3,Blcap,Aebp2,Kif1b,Srebf2,Immt,Kif3b,Oxct1,Enpp2,Ctcf,Hhex
SRF_Q4	34	1,52	6,94E-10	Insm1,G3bp2,Ncoa6,Foxp1,Nkx2-2,Fosb,Tcf4,Ppap2b,Tbl1x,Rere,Cald1,Cdkn1b,Taz,Dstn,Elavl4,Cfl2,Stat5b,Junb,Asph,Tpm3,Meis2,Csda,Anp32e,Herc1,Dusp6,Csrp1,Atrx,Serpinh1,Mbnl1,Rock2,Egr1,Anxa6,Lpp,Ctgf
STAT6_02	32	1,43	7,51E-10	Cox17,Clock,Gnb2,Traf3,Map3k3,Flt1,Hivep1,Cpeb4,Arid1a,Trim25,Polr1d,Ttc13,Slc38a2,Pitpnc1,Meis2,Dctn2,Ppp2r5c,Serpini1,Gphn,Vamp3,Dnajc7,Ctcf,Hhex,Znrf2,Fbxw11,Satb1,Mbnl1,Cd2ap,Nfat5,Zranb1,Vps39,Pum1
DR3_Q4	22	0,99	7,56E-10	Ppp4r1,Ywhag,Slc35e1,Kpnb1,Eif5a,Rbm14,Kcnb1,Ttc3,Pax6,Trim8,Ndufs2,Nbea,Trim41,Tmpo,Xpo1,Prkag2,Rock1,Asxl1,Ush1c,Bcl9,Atp2a2,Rbbp6
ETS_Q4	31	1,39	7,59E-10	Elk3,Sec24c,Foxp1,Ddit3,Etf1,E2f5,Tpp2,Itpr1,Srpr,Chic2,Extl2,Actr3,Pdap1,Pax6,Stat5b,Esm1,Junb,Baz2a,Ndufs2,Kif3b,Acsl5,Sec61a1,Cd2bp2,Cap1,Ikbkb,Trim41,Pik3r4,Ptk2,Canx,Tcf12,Ube2l3
NERF_Q2	31	1,39	8,71E-10	Elk3,Ddit3,Spred2,Tpp2,Atp1a1,Srpr,Lrrfip2,Uchl5,Agl,Flt1,Extl2,Diablo,Actr3,Itpkb,Topors,Elovl5,Pax6,Stat5b,Junb,Baz2a,Tusc3,Nrp1,Ndufs2,Slc4a2,Gbf1,Cap1,Ikbkb,Pak3,Rasa1,Tcf12,Ube2l3
AP1_01	33	1,48	9,18E-10	Mcart1,Ids,Wasf2,Ywhag,Lrrfip2,Abhd4,Traf3,Gabarapl1,Ptprr,Psmd2,Ewsr1,Psmd7,Baz2a,Aldoa,Bach1,Mecp2,Irak1,Slc38a2,Ulk1,Hdac3,Atp1b1,Dctn2,Sqstm1,Psmd11,Bag2,Anxa7,Vdr,Rb1cc1,Smarca2,Rbp4,Rab3d,Nudt11,Rit1

Transcription factor	*Gene Count*	*Percentage*	*p-Value*	*Genes induced in RasGrf1 KO pancreatic islets*
CHOP_01	30	1,35	9,97E-10	Itgb3bp,Sars,Adss,Etf1,Ppp2r2a,Itpr1,Tbl1x,Atm,Rab3ip,Lasp1,Gcg,Syncrip,Glud1,Usp9x,Tnpo3,Slc38a2,Rod1,Iars,Immt,Slc4a2,Csda,Gphn,Nfe2l2,Ctnnd1,Hhex,Vdr,Slc35a5,Srpk2,Mbnl1,Bcl2l11
NF1_Q6	69	3,09	1,01E-09	Calm1,G3bp2,Colec12,Acvr1,Bicd1,Lifr,Nkx2-2,Ddit3,Ckap4,Nav2,Gcnt2,Rlf,Tcf4,Arih1,Rere,Lrrfip2,Cald1,Tbrg1,Hectd1,Cacna1d,Psmf1,Hhip,Cpeb4,Mll5,Cs,Arhgap6,Timp3,Psmc5,Syncrip,Pdha1,Trim8,Foxa2,Abhd5,Tnpo3,Pum2,Rapgef4,Nap1l3,Ncald,Tpm3,Mtap,Pnrc2,Ubp1,Map4k3,Mef2a,Mtss1,Meis2,Cd2bp2,Mnt,Atp2a3,Enpp2,Robo1,Ptk2,Phyhipl,Prpf39,Klhl2,Smarca2,Rbp4,Vamp2,Hipk1,Pex7,Hbp1,Stx18,Mbnl1,Aco1,Rit1,Rrbp1,Ncam1,Cbfa2t2,Dlg3
CDX2_Q5	32	1,43	1,24E-09	Smad1,Ncor1,Elk3,Lpl,Gad1,Map2k1,Foxp1,Xpo7,Ptprr,Trim2,Elavl4,Cfl2,Wsb2,Elp4,Pdap1,Pax6,Gcg,Ndrg3,Dars,Pnrc1,Foxa2,Nrp1,Pcsk2,Meis2,Mnt,Robo1,Srpk2,Mbnl1,Zranb1,Vldlr,Chchd7,Cbfa2t2
SOX5_01	31	1,39	1,98E-09	Tnks2,Rreb1,G3bp2,Mospd2,Eif4ebp2,Rhob,Tcf4,Rere,Mxi1,Elavl4,Dhx40,Hhip,Rcn1,Cpeb4,Ppm1b,Ank3,Wasl,Nrp1,Pcsk2,Tnpo3,Atp6v1a,Cnot7,Nfe2l2,Dusp6,Hhex,Hbp1,Pura,Tcf12,Ncam1,Pum1,Cbfa2t2
OLF1_01	47	2,11	2,67E-09	Calm1,Luc7l2,Elk3,Fkbp2,Rab2b,Ubap1,Ywhag,Gnb2,Atf5,Gcnt2,Ppp2r2a,Sp4,Rnf44,Atp1a1,Nf2,Rab10,Flt1,Wsb2,Scg3,Pias1,Arid1a,Mll5,Pdap1,Lasp1,Tlk2,Kif5b,Baz2a,Sp3,Trim8,Smarce1,Adcy6,Cbx6,Epc1,Ndufs2,Mtss1,Meis2,Ube2n,Enpp2,Jarid2,Wdr13,Dcx,Nudt4,Egr1,Rbbp6,Chchd7,Lpp,Dlg3
PAX4_01	32	1,43	2,75E-09	Pabpc4,Smad1,Ywhaz,Pabpc1,Ddit3,Gnb2,Kpnb1,Sp4,Eif5a,Suox,Nf2,Cacna1d,Mxi1,Mapre1,Syncrip,Jund,Elavl1,Cbx6,Ccni,Dnajb2,Mnt,Ctbp2,Xpo1,Galnt10,Jarid2,Nrd1,Plagl1,Rbp4,Plekha1,Rbbp6,Psip1,Dlg3
E2F_Q2	24	1,08	3,27E-09	Pabpc4,Dap3,Rabif,Rlf,Wac,Ppp2r5a,Sfpq,Gng4,Mast2,Ythdf2,Gnb1,Atp1b1,Kif1b,Tpm3,Cap1,Anp32e,Vamp3,Adipor1,Csrp1,Phyhipl,Hipk1,Rnf2,Usp48,Pum1
AREB6_01	69	3,09	3,29E-09	Tnks2,Strn4,G3bp2,Ppp4r1,Cldn7,Mgat2,Itgb3bp,Gad1,Rab2b,Foxp1,Ppp2r2a,Clcn3,Dock9,Herc4,Nosip,Lrrfip2,Cdkn1b,Tbrg1,Flnb,Ptprr,Cacna1d,Ing2,Mllt10,Hivep1,Phc1,Elavl4,Itpkb,Ppm1b,Dach2,Pias1,Tra2a,Tlk2,Gcg,Klf5,Ndrg3,Polr1d,Dnmt3a,Ube2d3,Numa1,Tdrd3,Nrp1,Pgrmc1,Brd2,Ulk1,Neurod1,Dicer1,Ndufs2,Sf1,Acsl5,Nbea,Cnot7,Pcp4,Jarid2,Trpm7,Plagl1,Ptprf,Dcx,Rb1cc1,Kctd5,Fbxw11,Srpk2,Mbnl1,Tgfb2,Pura,Tcf12,Eif5,Ncam1,Oxr1,Chchd7
TAXCREB_01	22	0,99	3,77E-09	Luc7l2,Peg3,Fosb,Arih1,Cdkn1b,Pafah1b1,Hhip,Marcks,Dusp1,Ccnd2,Pnrc1,Gtf2a1,Poldip3,Jund,Dnajb2,Dlat,Ctcf,Asxl1,Osbp,Vamp2,Appbp2,Cd2ap
GFI1_01	45	2,02	4,69E-09	Colec12,Trim23,Pank1,Sars,Ppp2r2a,Tcf4,Ywhae,Map3k3,Cryzl1,Rhoa,Tcta,Atf2,Elavl4,Kpna3,Wasl,Ccng2,Hibadh,Pvrl3,Cpeb3,Brd2,Phf8,Jak1,Srebf2,Nap1l3,Iars,Mtmr4,Map4k3,Meis2,Gas2,Top1,Herc1,Mapk8,Pten,Atrx,Ctnnd1,Bcl9,Dcx,Hbp1,Srpk2,Mbnl1,Zranb1,Pura,Tcf12,Ncam1,Ets1
VDR_Q3	29	1,30	5,45E-09	Calm1,Fkbp2,Pcbp2,Ywhag,Ncoa6,Xpo7,Tpt1,Sin3a,Ndufa6,Eif5a,Kcnb1,Arid1a,Timp3,Sec63,Gtf2a1,Jund,Eif4g2,Meis2,Xpo1,Ctcf,Asxl1,Egln2,Prkci,Satb1,Dhx30,Cct7,Rrbp1,Rbbp6,Ncam1
E2F1_Q4	29	1,30	6,23E-09	Hnrnpa3,Luc7l2,Insm1,Wasf2,Nup153,Spred2,Kpnb1,Sin3a,Cdh2,Eif5a,Itpr1,Kpna6,Cdkn1b,Stxbp1,Trim28,Nfyc,Arpc1a,Ufd1l,Ece1,Ube2d3,Slc38a2,Cbx5,Epc1,Meis2,Ctcf,Fbxo9,Slbp,Ogdh,Rbbp6
MAZR_01	28	1,26	6,37E-09	Gata6,Luc7l2,Hnrpdl,Fkbp2,Clock,Gnb2,Etf1,Eif5a,Pafah1b1,Atf2,Nfyc,Mll5,Pax6,Tlk2,Mthfr,Junb,Baz2a,Cbfb,Dnmt3a,Aebp2,Sf1,Slc4a2,Son,Meis2,Nptx1,Trim41,Rock1,Ptprf
SRF_Q6	32	1,43	8,09E-09	Csnk1e,Insm1,G3bp2,Foxp1,Nkx2-2,Fosb,Sin3a,Ppap2b,Rere,Cald1,Cdkn1b,Agl,Taz,Elavl4,Cfl2,Actr3,Myadm,Stat5b,Matr3,Junb,Syncrip,Ube2d3,Asph,Kif1b,Tpm3,Herc1,Tes,Mbnl1,Rock2,Egr1,Anxa6,Lpp

Transcription factor	*Gene Count*	*Percentage*	*p-Value*	*Genes induced in RasGrf1 KO pancreatic islets*
NFY_01	29	1,30	8,10E-09	Wasf2,Rhob,M6pr,Abat,Gtf2i,Dstn,Elavl4,Numb,Mll5,Rpn2,Ufd1l,Junb,Wbp2,Racgap1,Pnrc1,Dnajb11,Vcp,Srebf2,Ppp2r5c,Fads1,Abca7,Dusp6,Xpo1,Jarid2,Atp2a2,Ubb,Ncam1,Ppm1a,Dlg3
HSF1_01	43	1,93	1,18E-08	Smad1,Elk3,Elf2,Gch1,Gad1,Ywhag,Sp4,Nosip,Cacna1d,Elavl4,Hspd1,Elovl5,Dusp1,Ube2e2,Arhgap6,Pnrc1,Hcfc1,Adcy6,Tnpo3,Cct8,Eif4g2,Atp6v1a,Srebf2,Rbpms,P4ha1,Fhl1,Ppp2r5c,Dapk1,Hspa8,Enpp2,Ptpra,Scamp1,Xpo1,Atp1b3,Bcl9,Pak3,Serpinh1,Hipk1,Cct3,Tnfaip1,Pura,Eif5,Ogt
TITF1_Q3	29	1,30	1,19E-08	Etv1,Wasf2,Foxp1,Nkx2-2,Tcf4,Itpr1,Lrrfip2,Ptbp2,Mxi1,Mllt10,Cfl2,Itpkb,Cpeb4,Dach2,Tra2a,Pax6,Mthfr,Ube2d3,Cnot2,Aebp2,Psmd11,Cnot7,Herc1,Jarid2,Hhex,Smarca2,Satb1,Srpk2,Pura
NFY_C	28	1,26	1,66E-08	Rreb1,Acvr1,Wasf2,Spc25,Ddit3,Tpp2,M6pr,Map3k3,Rbm14,Gtf2i,Gca,Elavl4,Nfyc,Ppm1b,Piga,Rpn2,Pax6,Brd8,Wbp2,Racgap1,Dnajb11,Cbx5,Pik3r1,Ppp2r5c,Abca7,Mbnl1,Ncam1,Dlg3
MZF1_02	28	1,26	2,17E-08	G3bp2,Ncoa6,Foxp1,Tpt1,Sp4,Ywhae,Ppap2b,Pafah1b1,Mllt10,Elavl4,Nfyc,Dhx40,Arid1a,Mll5,Ewsr1,Dnmt3a,Cbx6,Mgea5,P4ha1,Slc4a2,Son,Meis2,Oxct1,Xpo1,Asxl1,Ptprf,Hbp1,Chchd7
PAX4_02	29	1,30	2,27E-08	Csnk1e,Elk3,Etv1,Mtmr6,Pabpc1,Bace2,Tgfbr1,Foxp1,Tcf4,Ppap2b,Cyfip2,Mllt10,Atm,Gtpbp1,Cpeb4,Marcks,Cs,Pax6,Folr1,Ncald,Meis2,Cap1,Jarid2,Arhgap24,Dcx,Atp2a2,Vip,Mtif2,Cbfa2t2
PTF1BETA_Q6	28	1,26	2,42E-08	Csnk1e,Gata6,Man2c1,Lpl,Pank3,Ppp2r2a,Ywhae,Tbl1x,Lrrfip2,Ptbp2,Cald1,Hhip,Mll5,Ank3,Ankrd17,Dnmt3a,Dicer1,Ncald,Mtmr4,Robo1,Xpo1,Ctcf,Atp1b3,Smarca2,Rasa1,Pura,Pias2,Pum1
E2F1_Q6_01	28	1,26	2,42E-08	Casp2,Ncl,Insm1,Mgat2,Ncoa6,Cdc5l,Sin3a,Gng4,Atm,Ufd1l,Pax6,Klf5,Arhgap6,Mcm6,Syncrip,Numa1,Dnmt1,Cbx5,Mtss1,Meis2,Topbp1,Dnajc9,Rps19,Tmpo,Sumo1,Ctcf,Rb1cc1,Mcm7
AP1_Q2	31	1,39	3,28E-08	Mcart1,Nefh,Cryba2,Elk3,Sufu,Cdkn1b,Ptprr,Golga4,Dstn,Pdap1,Stat5b,Rad23b,Rab22a,Baz2a,Trim8,Irak1,Hdac3,Pitpnc1,Meis2,Ap2a2,Dctn2,Psmd11,Anxa7,Vdr,Smarca2,Fbxw11,Srpk2,Ubqln1,Rock2,Tcf12,Lpp
CDPCR3HD_01	28	1,26	3,52E-08	Hnrnpa3,Smad1,Etv1,Ywhag,Nkx2-2,Gnb2,Rlf,Rab10,Ing2,Dach2,Stat5b,Esm1,Aldoa,Slc38a2,Eif4g2,Aebp2,Ubl3,Srebf2,Son,Rbx1,Serpini1,Galnt4,Robo1,Trpm7,Rasgrf1,Cbfa2t2,Dlg3,Ctgf
FOXO1_02	28	1,26	3,52E-08	Scrn3,Etv1,Eif4ebp2,Lifr,Nkx2-2,Xpo7,Gcnt2,Sin3a,Tbl1x,Rasd1,Mbtps1,Cfl2,Ppm1b,Pik3c2a,Mll5,Rad21,Brd8,Trim8,Ulk1,Pitpnc1,Stx6,Meis2,Sqstm1,Asxl1,Ndrg1,Hbp1,Pura,Chchd7
STAT_01	29	1,30	4,09E-08	Sec24c,Mospd2,Foxa3,Arih1,Rab10,Flt1,Bclaf1,Cacna1d,Ap2m1,Phc1,Set,Gabbr1,Lasp1,Trim25,Ccnd2,Ank3,Myo18a,Asph,Nrp1,Mecp2,Bet1,Ndufs2,Grinl1a,Enpp2,Asxl1,Bcl9,Cnot4,Vip,Rasa1
CEBPB_02	32	1,43	4,35E-08	Csnk1e,Smad1,Ptpn12,Mospd2,Foxp1,Ckap4,Bnip3l,Rhob,Map3k3,Cdkn1b,Smarcad1,Rab3ip,Ppm1b,Pdcl,Pdap1,Dusp1,Ccnd2,Kpna3,Rad23b,Klf5,Ube2e2,Syncrip,Rbpms,Nfe2l2,Top1,Ndrg1,Smarca2,Stx18,Mbnl1,Ubqln1,Tcf12,Rrbp1
ZIC2_01	28	1,26	4,46E-08	Gata6,Slc25a11,Ywhag,Foxp1,Nkx2-2,Becn1,Sfpq,Rab11a,Dhx40,Tob2,Tlk2,Rab22a,Baz2a,Timp3,Syncrip,Calr,Zmynd11,Cbx6,Calb1,Osbpl2,Wdr13,Ctnnd1,Dhx30,Pura,Rbbp6,Ogt,Rac1,Macf1
AP1_Q4_01	30	1,35	4,51E-08	Nefh,Cryba2,Elk3,Itm2b,Psme4,Ddit3,Psmd1,Abhd4,Traf3,Ap2m1,Baz2a,Aldoa,Bach1,Hdac3,Atp1b1,Sqstm1,Mnt,Sec24d,Psmd11,Bag2,Anxa7,Scamp1,Ppp1r15a,Vdr,Rb1cc1,Rbp4,Fbxw11,Rab3d,Srpk2,Rit1

Transcription factor	*Gene Count*	*Percentage*	*p-Value*	*Genes induced in RasGrf1 KO pancreatic islets*
WHN_B	28	1,26	6,45E-08	Insm1,Senp2,Slc35e1,Nkx2-2,Dlg5,Ppp3ca,Arih1,Uchl5,Extl2,Rab11a,Tob2,Snx9,Hspd1,Sf3b1,Junb,Ankrd17,Ythdf2,Arid4b,Nsf,Mtmr4,Top1,Herc1,Jarid2,Sumo1,Ctcf,Nudt11,Mbnl1,Dhx30
E4BP4_01	32	1,43	7,20E-08	Csnk1e,Calm1,Nmt2,Ywhag,Cdk8,Xpo7,Prickle1,Clasp1,Chic2,Elavl4,Eef2,Gnpnat1,Hspd1,Mll5,Fbxo3,Trim8,Ythdf3,Pcsk2,Mecp2,Irak1,Srebf2,Pck2,Ing1,Pik3r1,Atp2a3,Nptx1,Epn2,Sumo1,Hist3h2a,Pura,Ogt,Zfp161
NMYC_01	29	1,30	7,20E-08	Pabpc4,Etv1,Nup153,Pabpc1,Adss,Nkx2-2,Pank3,Atp6v0b,Cacna1d,Zcchc7,Hhip,Gtpbp1,Hspd1,Eif4b,Zzz3,Dnmt3a,Wbp2,Bcl7c,Ipo4,Mnt,Ctbp2,Gna13,Top1,Plagl1,Dnajb9,Hbp1,Mthfd1,Anxa6,Rbbp6
ETS1_B	28	1,26	7,25E-08	Elk3,Sec24c,Pabpc1,Foxp1,Spred2,Etf1,E2f5,Tpp2,Srpr,Galnt2,Cacna1d,Extl2,Itpkb,Pax6,Esm1,Junb,Baz2a,Stk16,Rod1,Ndufs2,Cap1,Ikbkb,Trim41,Ptk2,Canx,Rock1,Vps39,Ube2l3
SRF_C	27	1,21	7,86E-08	G3bp2,Acvr1,Ywhaz,Foxp1,Nkx2-2,Fosb,Cald1,Taz,Dstn,Elavl4,Cfl2,Actr3,Stat5b,Matr3,Junb,Syncrip,Tpm3,Cap1,Top1,Herc1,Dusp6,Ctnnd1,Mbnl1,Rock2,Egr1,Anxa6,Lpp
CETS1P54_01	26	1,17	9,74E-08	Rplp2,Tnks2,Cox17,Sec24c,Itgb3bp,Eif5a,Herc4,Nosip,Dnajc1,Extl2,Cpeb4,Calu,Itm2c,Baz2a,Fbxo3,Dnmt1,Hmg20a,Acp2,Cap1,Pik3r4,Prkag2,Nrd1,Ctcf,Pafah1b2,Mtmr2,Mtif2
HSF2_01	27	1,21	9,92E-08	Smad1,Gad1,Ywhag,Herc4,Cacna1d,Elavl4,Hspd1,Elovl5,Dusp1,Ewsr1,Ube2e2,Trim8,Cct8,Eif4g2,Atp6v1a,Srebf2,Rbpms,Fhl1,Hspa8,Enpp2,Ptpra,Jarid2,Bcl9,Serpinh1,Hipk1,Cct3,Eif5
DR4_Q2	27	1,21	9,92E-08	Rreb1,Fkbp2,Gad1,Polr3c,Foxp1,Gnb2,Npepps,Rab10,Elavl4,Pias1,Adam9,Ube2d3,Taf10,Arid4b,Eno2,Atp1b1,Pum2,Ndufs2,Son,Meis2,Ctbp2,Rnf11,Jarid2,Arhgap24,Dcx,Rb1cc1,Plekha1
TEF1_Q6	43	1,93	1,16E-07	Man1a2,Lifr,Ddit3,Sin3a,Nap1l5,Chic2,Rbm14,Ap2m1,Elavl4,Scn8a,Cfl2,Itgb1,Myadm,Arid1a,Mll5,Hdgf,Ccnd2,Folr1,Ank3,Ptprj,Mast2,Jund,Atp1b1,Kif1b,Epc1,Mtss1,Mnt,Slk,Chka,Adipor1,Gdi1,Wdr13,Arhgap24,Asxl1,Pik3c3,Ptprf,Prkci,Smarca2,Appbp2,Rock2,Rnf2,Dlg3,Ctgf
E2F1_Q4_01	26	1,17	1,23E-07	Casp2,Ncl,Insm1,Nup153,Ncoa6,Kpnb1,Sin3a,Ccnt2,Dhx40,Ufd1l,Klf5,Arhgap6,Mcm6,Numa1,Dnmt1,Cbx5,Meis2,Topbp1,Rps19,Top1,Pik3r4,Ctcf,Fbxo9,Atrx,Mcm7,Chgb
SP1_Q4_01	27	1,21	1,41E-07	Hnrpdl,Rab2b,Ncoa6,Tcf4,Iqgap1,Cacna1d,Kcnb1,Trim28,Nfyc,Pias1,Cs,Lasp1,Drap1,Timp3,Bcl7c,Smarce1,Rod1,Creb1,Klhl7,Golga3,Chka,Ndrg1,Vamp2,Atp2a2,Hipk1,Slbp,Nfat5
PAX2_02	28	1,26	1,99E-07	Tnks2,Luc7l2,Elk3,Tgfbr1,Dner,Ppp3ca,Tcf4,Rhoa,Tcta,Pafah1b1,Trim2,Elavl4,Cfl2,Marcks,Lasp1,Trim8,Meis2,Ppp2r5c,Serpini1,Gas2,Galnt4,Rnf11,Xpo1,Ctnnd1,Hipk1,Pura,Tcf12,Eif5
CREB_Q3	27	1,21	2,01E-07	Spred2,Prickle1,Rnf44,Eif5a,Arih1,Cdkn1b,Smarcad1,Tbc1d15,Dhx40,Thoc1,Hspd1,Rnps1,Dusp1,Ccnd2,Esm1,Arid4b,Jund,Srebf2,Meis2,Sec24d,Ap2b1,Dnajc7,Nrd1,Hist3h2a,Tnfaip1,Usp48,Ncam1
PITX2_Q2	55	2,47	2,23E-07	Insm1,Acvr1,Gad1,Clock,Ubap1,Sufu,Lifr,Gnb2,Sp4,Ppp3ca,Ocln,Tcf4,Clasp1,Itpr1,Npepps,Phc1,Rab3ip,Tob2,Cpeb4,Ppm1b,Mll5,Tlk2,Stat5b,Klf5,Esm1,Adam9,Prpf8,Brd4,Mecp2,Jund,Hmg20a,Ndufs2,Nsf,Ncald,Rbpms,Slc4a2,Mtss1,Meis2,Ube2n,Ctbp2,Cnot7,Dusp6,Robo1,Tm4sf4,Jarid2,Ctcf,Znrf2,Dcx,Smarca2,Slmap,Ppp3r1,Dhx30,Cd2ap,Pura,Actr10
LYF1_01	28	1,26	2,46E-07	Hnrnpa3,Syne2,Pank1,Terf2,Pcbp2,Gnb2,Prickle1,Tcf4,Clasp1,Eif5a,Atp6v0b,Cald1,Nrp1,Smyd2,Eno2,Dpf2,Ndufs2,Slc4a2,Serpini1,Mnt,Dusp6,Jarid2,Gdi1,Rock1,Asxl1,Ptprf,Fbxw11,Tes
LMO2COM_01	27	1,21	2,50E-07	Trim23,Cdh2,Dock9,Npepps,Map3k3,Keap1,Tjp1,Kcnb1,Myadm,Mll5,Ccnd2,Kif5b,Ptprj,Dnmt3a,Trim8,B4galt6,Atp1b1,Invs,Map3k4,Ppp2r5c,Calb1,Nbea,Vdr,Zranb1,Psip1,Oxr1,Ppm1a

Transcription factor	*Gene Count*	*Percentage*	*p-Value*	*Genes induced in RasGrf1 KO pancreatic islets*
BACH2_01	29	1,30	2,58E-07	Tnks2,Cryba2,Psme4,Ddit3,Rplp0,Syp,Arih1,Rasd1,Gabarapl1,Sms,Rab34,Stat5b,Baz2a,Gpr56,Aldoa,Bach1,Ndel1,Brd2,Hdac3,Eno2,Ctnnal1,Mnt,Sec24d,Psmd11,Vdr,Rab3d,Ubqln1,Rit1,Lpp
HNF1_Q6	27	1,21	2,78E-07	Fam20c,Rreb1,Ncor1,Clock,Tulp4,Prickle1,Lrrfip2,Cyfip2,Rab3ip,Cs,Ccnd2,Hnf4a,Nrp1,Ppp2r5c,Gas2,Pbx2,Slc12a2,Tm4sf4,Ctcf,Arhgap24,Bcl9,Egr1,Pura,Tcf12,Chchd7,Lpp,Cbfa2t2
AP2_Q6_01	26	1,17	3,56E-07	Pabpc4,Pabpc1,Mgat2,Terf2,Ncoa6,Ube2s,Foxa3,Ppp2r5a,Trim28,Nfyc,Mafb,Pias1,Arid1a,Mll5,Tlk2,Rad21,Dnmt3a,Cpeb3,Eif4g2,Mgea5,Adipor1,Ctcf,Znrf2,Hdlbp,Cirbp,Dlg3
AP1FJ_Q2	29	1,30	4,84E-07	Mcart1,Cryba2,Map2k1,Pafah1b1,Ptprr,Golga4,Dstn,Gabbr1,Pdap1,Stat5b,Rad23b,Rab22a,Baz2a,Trim8,Hcfc1,Irak1,Hdac3,Atp6v1a,Pitpnc1,Meis2,Psmd11,Anxa7,Vdr,Smarca2,Fbxw11,Ubqln1,Rock2,Tcf12,Lpp
USF_Q6	26	1,17	5,02E-07	Polr3c,Pank3,Sumf1,Hmgn1,Nfx1,Dusp1,Ewsr1,Zzz3,Dnmt3a,Gtf2a1,Brd2,Slc38a2,Neurod1,Epc1,Ncald,Stx6,Ctbp2,Gna13,Nptx1,Canx,Egln2,Slc35a5,Pura,Rnf146,Anxa6,Rbbp6
E2F_Q3_01	25	1,12	6,45E-07	Casp2,Ncl,Insm1,Nup153,Ncoa6,Kpnb1,Sin3a,Dhx40,Pias1,Ufd1l,Klf5,Arhgap6,Mcm6,Numa1,Dnmt1,Cbx5,Meis2,Topbp1,Rps19,Top1,Pik3r4,Ctcf,Fbxo9,Mcm7,Chgb
AP1_Q2_01	29	1,30	7,18E-07	Elk3,Wasf2,Rnf149,Ncoa6,Psme4,Syp,Tcf4,Elavl4,Rab34,Klf5,Ank3,Mast2,Dnaja4,Mecp2,Irak1,Ndel1,Brd2,Eno2,Atp1b1,Rbpms,Sqstm1,Psmd11,Bag2,Rb1cc1,Smarca2,Rit1,Cldn4,Tcf12,Lpp
USF_Q6_01	24	1,08	7,22E-07	Ssr1,Etv1,Hnrpdl,Gnb2,Pank3,Rnf44,Eif5a,Kbtbd2,Nfx1,Ewsr1,Ankrd17,Dnmt3a,Wbp2,Gtf2a1,Neurod1,Stx6,Gna13,Nptx1,Egln2,Cnot4,Wdr1,Lamp1,Vldlr,Ppm1a
CP2_01	27	1,21	7,32E-07	Fkbp2,Cdk8,Slc35e1,Creb3l2,Cdh2,Dock9,Abhd4,Cald1,Rhoa,Rab10,Mll5,Pax6,Ewsr1,Esm1,Foxa2,Vcp,Epc1,Sf1,Glg1,Ptov1,Hipk2,Atp2a3,Smarca2,Vamp2,Efemp2,Satb1,Pak2
ER_Q6_01	27	1,21	7,32E-07	Nnt,Lifr,Nkx2-2,Bnip3l,Rhob,Cald1,Tbc1d15,Psmf1,Gtpbp1,Pdha1,Dnmt3a,Aldoa,Srp68,Immt,Tpm3,Mnt,Gna13,Hipk2,Jarid2,Asxl1,Dcx,Satb1,Srpk2,Pole3,Pura,Fbxl14,Rrbp1
ALPHACP1_01	26	1,17	7,64E-07	Wasf2,Pank1,Gnb2,Ilf2,Cald1,Abat,Elavl4,Ddc,Ppm1b,Ccng2,Pnrc1,Dnajb11,Brd2,Vcp,Sf1,Pnrc2,Son,Stambp,Abca7,Dusp6,Xpo1,Pcp4,Jarid2,Atp2a2,Ubb,Dlg3
NFKB_Q6_01	29	1,30	7,67E-07	Etv1,Dap3,Nr3c1,Sin3a,Eif5a,Baz2b,Gng4,Hivep1,Cpeb4,Myadm,Arid1a,Lasp1,Ccnd2,Ptprj,Ube2d3,Gnb1,Pcsk2,Atp1b1,N4bp1,Tpm3,Creb1,Ipo4,Sec61a1,Gphn,Top1,Dusp6,Slc12a2,Ilk,Nfat5
CEBPA_01	26	1,17	8,21E-07	Csnk1e,Clock,Mospd2,Mxi1,Cfl2,Rab3ip,Hhip,Mll5,Ube2e2,Sp3,Syncrip,Trim8,Usp9x,Pcsk2,Trim37,Calr,Cbx6,Pum2,Meis2,Jarid2,Rb1cc1,Smarca2,Mbnl1,Nfat5,Cldn4,Rrbp1
SRY_02	26	1,17	8,21E-07	G3bp2,Ywhag,Nkx2-2,Tcf4,Kbtbd2,Mxi1,Elavl4,Dhx40,Cfl2,Rcn1,Pias1,Wasl,Myo18a,Nrp1,Ulk1,Eif4g2,Nsf,Pik3r1,Dusp6,Pak3,Mcm7,Satb1,Nudt11,Pura,Pum1,Cbfa2t2
FOXO1_01	26	1,17	8,21E-07	Gata6,Nnt,Scrn3,Smad1,Etv1,Eif4ebp2,Nkx2-2,H2afv,Taz,Mll5,Dusp1,Lasp1,Ccng2,Trim8,Ythdf3,Slc38a2,Ubl3,Asxl1,Hhex,Ndrg1,Mcm7,Hipk1,Hbp1,Pura,Chchd7,Cbfa2t2
SP1_01	26	1,17	8,21E-07	Gad1,Kpnb1,Sp4,Mtmr3,Eif5a,Rab10,Nfyc,Myadm,Arid1a,Ece1,Sec63,Syncrip,Dnmt3a,Ube2d3,Eps15,Mtss1,Gbf1,Mnt,Epn2,Egln2,Osbp,Dpagt1,Hs2st1,Tcf12,Nagk,Vldlr
MYB_Q5_01	26	1,17	8,21E-07	Ndufc1,Rreb1,G3bp2,Ddit3,Xpo7,Gcnt2,Prickle1,Ywhae,Elavl4,Pla2g6,Pias1,Mll5,Ccnd2,Klf5,Brd8,Ankrd17,Myo18a,Aldoa,Ndel1,Mdm1,Gphn,Xpo1,Canx,Hhex,Smarca2,Tcf12

Transcription factor	*Gene Count*	*Percentage*	*p-Value*	*Genes induced in RasGrf1 KO pancreatic islets*
GCM_Q2	25	1,12	9,51E-07	Gata6,Pank1,Pcbp2,Ncoa6,Tbl1x,Ccnt2,Rbm5,Kcnb1,Arid1a,Klf5,Ptprj,Ankrd17,Ythdf2,Ube2d3,Trim8,Usp9x,Neurod1,Map4k3,Mnt,Fbxo9,Efemp2,Rasgrf1,Srpk2,Mbnl1,Pura
CP2_02	25	1,12	1,05E-06	Etv1,Phpt1,Fkbp2,Nisch,Ywhag,Flot2,Cald1,Rab10,Hectd1,Cs,Junb,Mast2,Trim8,Hcfc1,Bach1,Epc1,Creb1,Ppp2r5c,Mnt,Cap1,Dusp6,Ctnnd1,Dcx,Cldn4,Rbbp6
ELF1_Q6	25	1,12	1,05E-06	Arpc2,Pabpc1,Spred2,Ppp3ca,Cnot6l,Ttc15,Iqgap1,Myadm,Marcks,Pax6,Esm1,Lims1,Nrp1,Nck1,Tbc1d8,Sf1,Acsl5,Ikbkb,Atp2a3,Jarid2,Ctcf,Ctnnd1,Ncam1,Pum1,Rac1
HP1SITEFACTOR_Q6	25	1,12	1,17E-06	Smad1,Ncor1,Gcnt2,Prickle1,Nosip,Cyfip2,Rab3ip,Rcor1,Cs,Syncrip,Foxa2,Eif4g2,Pum2,Kif1b,Ncald,Pik3r1,Pja1,Vamp3,Xpo1,Ctcf,Hhex,Rbp4,Hbp1,Mbnl1,Vldlr
FOXM1_01	26	1,17	1,38E-06	Pabpc1,Ddit3,Khdrbs1,Foxa3,Abhd4,Cdkn1b,Rhoa,Tcta,Elavl4,Pax6,Syncrip,Neurod1,Atp1b1,Slc4a2,Mtss1,Meis2,Psmd11,Anp32e,Jarid2,Ctcf,Klhl2,Rasgrf1,Tcf12,Ncam1,Pum1,Chchd7
TTF1_Q6	26	1,17	1,53E-06	Rreb1,Wasf2,Trrap,Map2k1,Tcf4,Ptbp2,Flt1,Mxi1,Elavl4,Cfl2,Hhip,Mafb,Klf5,Ccng2,Adcy6,Blcap,Brd2,Cnot2,Pitpnc1,Creb1,Strn3,Slc4a2,Dock7,Cap1,Jarid2,Wdr13
CEBPDELTA_Q6	25	1,12	1,61E-06	Smad1,Itgb3bp,Foxp1,Fosb,Arih1,Rab3ip,Ccnl1,Marcks,Pdap1,Hdgf,Ccnd2,Ube2e2,Timp3,Foxa2,Usp9x,Pcsk2,Arid4b,Neurod1,Srebf2,Pik3r1,Dctn2,Vamp3,Pten,Mbnl1,Ppm1a
HNF1_01	26	1,17	1,68E-06	Fam20c,Gata6,Rreb1,Pabpc1,Clock,Prickle1,Cald1,Rab3ip,Cs,Ttr,Hnf4a,Stat5b,Foxa2,Nrp1,E2f6,Ppp2r5c,Gas2,Nfe2l2,Pbx2,Slc12a2,Tm4sf4,Arhgap24,Bcl9,Egr1,Chchd7,Cbfa2t2
AP2_Q3	26	1,17	1,85E-06	Pabpc1,Clock,Nr3c1,Ncoa6,Etf1,Psmd1,Map3k3,Hectd1,Gorasp2,Mafb,Purb,Eif4g2,Ubl3,Epc1,Pitpnc1,Mgea5,P4ha1,Sf1,Mnt,Dnajc7,Ctcf,Hdlbp,Nfat5,Pura,Fbxl14,Ube2l3
ETF_Q6	15	0,67	2,06E-06	Mfn2,Dap3,Kpna6,Rere,Uchl5,Wac,Nfyc,Arid1a,Mast2,Ythdf2,Gnb1,Epc1,Vamp3,Rnf2,Usp48
CEBP_Q2	24	1,08	2,07E-06	Csnk1e,Ptpn12,Fkbp2,Clock,Foxp1,Ddit3,Tcf4,Cryzl1,Cfl2,Rab3ip,Pdap1,Pax6,Ube2e2,Usp9x,Slc38a2,Rbpms,Nfe2l2,Jarid2,Clic4,Smarca2,Stx18,Ubqln1,Pura,Tcf12
TEL2_Q6	23	1,03	2,13E-06	Sec24c,Ddit3,E2f5,Herc4,Agl,Extl2,Diablo,Rnps1,Junb,Tusc3,Ndufs2,Acsl5,Cd2bp2,Ube2n,Cap1,Ikbkb,Trim41,Pik3r4,Ptk2,Canx,Scamp2,Pafah1b2,Tcf12
AHRARNT_01	18	0,81	2,18E-06	Tnks2,Elk3,Insm1,Phpt1,Trim23,Pabpc1,Nr3c1,Mospd2,Hectd1,Mxi1,Elavl4,Pax6,Adam9,Gtf2a1,Tnpo3,Pitpnc1,Mbnl1,Ctgf
MYCMAX_02	26	1,17	2,21E-06	Pabpc4,Insm1,Etv1,Hnrpdl,Adss,Tcf4,Kbtbd2,Cad,Fxr1,Diablo,Hdgf,Eif4b,Ankrd17,Timp3,Syncrip,Bach1,Cbx5,Top1,Galnt4,Enpp2,Xpo1,Dpagt1,Mbnl1,Tgfb2,Mthfd1,Ppm1a
STAT5B_01	32	1,43	2,46E-06	Sec24c,Foxa3,Arih1,Flt1,Bclaf1,Extl2,Ap2m1,Phc1,Arid1a,Trim25,Ccnd2,Ank3,Myo18a,Asph,Nrp1,Pcsk2,Ugcg,Mecp2,Poldip3,Bet1,Ndufs2,Ncald,Pik3r1,Grinl1a,Smpd1,Enpp2,Birc2,Trpm7,Asxl1,Bcl9,Plagl1,Vip
OCT1_02	24	1,08	2,51E-06	Insm1,Lpl,Zhx1,Foxp1,Gal3st1,Tcf4,Rab10,Cacna1d,Elavl4,Ywhab,Pax6,Ank3,Ptprj,Arhgap6,Dnaja4,Pcsk2,Blcap,Rps19,Dcx,Smarca2,Mbnl1,Pura,Rnf2,Chchd7
AP2GAMMA_01	24	1,08	2,79E-06	Calm1,Pabpc4,Cldn7,Hnrpdl,Pabpc1,Nr3c1,Atf5,Sp4,Trim28,Mafb,Cpeb4,Arid1a,Tlk2,Ythdf2,Jund,Cbx6,P4ha1,Jarid2,Ctcf,Rock1,Znrf2,Pak3,Egln2,Nfat5
MYB_Q6	24	1,08	3,10E-06	Pabpc1,Tpp2,Gcnt2,Ube2s,Prickle1,Ywhae,Rhoa,Snrpd1,Tcta,Pafah1b1,Phc1,Mll5,Klf5,Ankrd17,Myo18a,Aldoa,Eif4g2,Mdm1,Mnt,Jarid2,Hhex,Fbxl14,Cd44,Dlg3
FOXO4_02	25	1,12	3,15E-06	Scrn3,Smad1,Nr3c1,Xpo7,Rasd1,Nap1l5,Taz,Cfl2,Klf5,Brd8,Trim8,Ugcg,Eif4g2,Pitpnc1,Meis2,Sqstm1,Enpp2,Sumo1,Asxl1,Dcx,Mcm7,Slmap,Hbp1,Pura,Chchd7

Transcription factor	*Gene Count*	*Percentage*	*p-Value*	*Genes induced in RasGrf1 KO pancreatic islets*
SRF_Q5_01	25	1,12	3,15E-06	G3bp2,Foxp1,Nkx2-2,Fosb,Ppap2b,Rere,Cald1,Cdkn1b,Taz,Dstn,Elavl4,Cfl2,Actr3,Myadm,Stat5b,Junb,Timp3,Tpm3,Csda,Csrp1,Ctnnd1,Mbnl1,Egr1,Anxa6,Lpp
STAT4_01	25	1,12	3,15E-06	Arpc2,Lpl,Clock,Gnb2,Phtf1,Flt1,Extl2,Cpeb4,Polr1d,Ube2d3,Slc38a2,Calr,Ubl3,Pitpnc1,Gphn,Leprotl1,Hhex,Slmap,Satb1,Mbnl1,Nfat5,Nudt4,Ncam1,Ets1,Hipk3
MYCMAX_B	25	1,12	3,43E-06	Ncl,Insm1,Hnrpdl,Pabpc1,Etf1,Kpnb1,Ube2s,Eif5a,Ewsr1,Ythdf2,Ube2d3,Trim8,Foxa2,Gtf2a1,Pgrmc2,Neurod1,Nsf,Spg21,Sec61a1,Ptov1,Rps19,Prkag2,Znrf2,Rnf146,Snx5
SMAD_Q6	25	1,12	3,43E-06	Csnk1e,Elk3,Sufu,Ppp1r15b,Cald1,Phc1,Dach2,Arid1a,Mll5,Ufd1l,Sp3,Nrp1,Gtf2a1,Smyd2,Eif4g2,Neurod1,Cbx5,Ccni,Pik3r1,Slc4a2,Anp32e,Rock1,Cnot4,Fgfr1op2,Mbnl1
MTF1_Q4	25	1,12	3,43E-06	Tnks2,Elk3,Xpo7,H2afv,Ppp3ca,Eif5a,Atf2,Mllt10,Marcks,Foxa2,Mecp2,Hdac3,Eif4g2,Mtmr4,Creb1,Mtss1,Mnt,Rhot1,Ctnnd1,Bcl9,Smarca2,Fgfr1op2,Nfat5,Egr1,Pura
TCF11_01	23	1,03	3,49E-06	Camsap1,Lman1,Cfl2,Cpeb4,Pdap1,Tceb3,Ccnd2,Esm1,Rab22a,Nrp1,Pcsk2,Vcp,Aig1,Atp1b1,Txnrd1,Kif1b,Zfr,Mnt,Smarca2,Srpk2,Rit1,Tnfaip1,Ppm1a
PAX4_04	23	1,03	3,49E-06	Etv1,Foxp1,Nkx2-2,Tcf4,Ppap2b,Cald1,Cryzl1,Rab10,Hhip,Pik3c2a,Marcks,Lims1,Usp9x,Cnot2,Dicer1,Ccni,Mnt,Jarid2,Rock1,Asxl1,Pura,Tcf12,Chchd7
MZF1_01	24	1,08	3,64E-06	Fam20c,Gata6,Hnrnpa3,Gad1,Cdk8,Eif5a,Flt1,Pafah1b1,Cacna1d,Mxi1,Mllt10,Gabbr1,Arid1a,Mll5,Foxa2,Brd4,Brd2,Eif4g2,Cbx6,Meis2,Csda,Pbx2,Xpo1,Ctgf
SPZ1_01	25	1,12	3,72E-06	Pabpc1,Ubap1,Xpo7,Tpt1,Kpnb1,Sp4,Snrpd1,Hivep1,Ccnd2,Baz2a,Syncrip,Ythdf2,Pcsk2,Gtf2a1,Eif4g2,Eps15,Pitpnc1,Tpm3,Meis2,Ppp2r5c,Xpo1,Ctcf,Cnot4,Efemp2,Zranb1
SP1_Q6_01	23	1,03	3,84E-06	Sec24c,Rab2b,Adss,Fosb,Tcf4,Iqgap1,Trim28,Pias1,Ece1,Lasp1,Drap1,Klf5,Bcl7c,Smarce1,Adam17,Ulk1,Eps15,Ptov1,Golga3,Chka,Vamp2,Atp2a2,Nfat5
ZID_01	23	1,03	4,26E-06	Elk3,Myo10,Adss,Foxp1,Sp4,Tcf4,Phtf1,Pafah1b1,Ap2m1,Kpna4,Marcks,Pax6,Mecp2,Phf8,Meis2,Serpini1,Ube2n,Ctcf,Mpp3,Rb1cc1,Atp2a2,Mbnl1,Dhx30
CDC5_01	26	1,17	4,47E-06	Rreb1,Ywhag,Foxp1,Clasp1,Cald1,Nap1l5,Mxi1,Itpkb,Cpeb4,Elp3,Rcor1,Sec63,Nrp1,Rod1,Pik3r1,Nptx1,Enpp2,Pbx2,Atrx,Ctnnd1,Vdr,Smarca2,Hipk1,Mbnl1,Pura,Actr10
SRY_01	21	0,94	4,55E-06	Tnks2,Fkbp2,Ckap4,Ppap2b,Sh3glb1,Gabarapl1,Mxi1,Elavl4,Pik3c2a,Pax6,Ank3,Zzz3,Trim8,Cpeb3,Arid4b,Eif4g2,Dusp6,Bcl9,Vdr,Atp2a2,Ncam1
TCF1P_Q6	24	1,08	4,84E-06	Pabpc4,Gata6,Tpp2,Sp4,Uchl5,Nap1l5,Wac,Elavl4,Scgn,Atm,Cpeb4,Mll5,Pax6,Foxa2,Blcap,Hmgcs1,Clk4,Pik3r1,Mef2a,Mtss1,Meis2,Jarid2,Satb1,Mtif2
STAT_Q6	25	1,12	4,88E-06	Mgat2,Fosb,Prickle1,Camsap1,Cald1,Agl,Mkrn1,Lman2,Arpc1a,Hspd1,Topors,Ank3,Asph,Pcsk2,Tnpo3,Rod1,Grinl1a,Anxa7,Enpp2,Trpm7,Rock1,Scamp2,Zranb1,Pdcd6ip,Ube2l3
FOX_Q2	23	1,03	5,16E-06	Gata6,Rreb1,Eif4ebp2,Aldh9a1,Tpp2,Gcnt2,H2afv,Tbl1x,Gabarapl1,Rab3ip,Cpeb4,Scg3,Pik3c2a,Ttr,Ccnd2,Junb,Ctcf,Smarca2,Mcm7,Atp2a2,Pura,Ncam1,Chchd7
HAND1E47_01	25	1,12	5,33E-06	Calm1,Lpl,Tgfbr1,Creb3l2,Cald1,Rab10,Elavl4,Elp4,Rwdd1,Stat5b,Klf5,Arhgap6,Ankrd17,Ube2d3,Adcy6,Taf10,Tmpo,Dcx,Vip,Stx18,Mbnl1,Pura,Fbxl14,Armcx1,Cbfa2t2
E2F_03	23	1,03	5,68E-06	Luc7l2,Nup153,Peg3,Slc25a11,Eif5a,Ccnt2,Ufd1l,Klf5,Arhgap6,Mcm6,Eif4g2,Dnmt1,Cbx5,Topbp1,Dnajc9,Anp32e,Top1,Vamp3,Ctcf,Plagl1,Egln2,Mcm7,Slbp

Transcription factor	*Gene Count*	*Percentage*	*p-Value*	*Genes induced in RasGrf1 KO pancreatic islets*
USF_C	26	1,17	5,74E-06	Pabpc4,Suclg2,Etv1,Pabpc1,Eif5a,Cacna1d,Diablo,Set,Hhip,Pax6,Eif4b,Dnmt3a,Wbp2,Gtf2a1,Ipo4,Mnt,Rps19,Gna13,Nptx1,Mpp3,Znrf2,Cnot4,Dnajb9,Mthfd1,Snx5,Anxa6
HNF1_C	23	1,03	6,96E-06	Fam20c,Cpn1,Bace2,Clock,Nkx2-2,Tcf4,Tbl1x,Lrrfip2,Mxi1,Rab3ip,Marcks,Foxa2,Nrp1,Ppp2r5c,Pbx2,Slc12a2,Arhgap24,Rbp4,Mbnl1,Egr1,Ncam1,Lpp,Cbfa2t2
MIF1_01	20	0,90	7,17E-06	Ttc8,E2f5,Ywhae,Dnajc1,Arid1a,Ccnd2,Pla2g12a,Tusc3,Hmgcs1,Cbx6,Kif3b,Gbf1,Rnf41,Dpagt1,Ubb,Dhx30,Hs2st1,Cd44,Bbs2,Rac1
XBP1_01	17	0,76	7,30E-06	Armcx2,Mgat2,Nkx2-2,Elf1,Tm9sf1,Tcf4,Srpr,Gtf2a1,Srp68,Epc1,Itch,Sec61a1,Sec24d,Kdelr1,Osbp,Hbp1,Pura
P53_DECAMER_Q2	25	1,12	7,55E-06	Elk3,Etv1,Wasf2,Senp2,Dock9,Mxi1,Rbm5,Cramp1l,Rcor1,Baz2a,Tdrd3,Phf8,Hmgcs1,Ubl3,Tbc1d8,Ing1,Pitpnc1,Strn3,Rps19,Pxk,Dnajc7,Kctd5,Smarca2,Cnot4,Dhx30
ZIC3_01	25	1,12	7,55E-06	Foxp1,Nkx2-2,Thrap3,Itpr1,Becn1,Taz,Rab11a,Piga,Pax6,Tlk2,Folr1,Timp3,Mast2,Syncrip,Foxa2,Zmynd11,Osbpl2,Vamp3,Ctnnd1,Efemp2,Dhx30,Hs2st1,Pura,Ogt,Rac1
IRF1_Q6	25	1,12	7,55E-06	Syne2,Wasf2,Trim23,Cald1,Riok3,Elavl4,Calu,Pias1,Rcor1,Pax6,Atp6v0d1,P4ha1,Slc4a2,Tia1,Serpini1,Rps19,Slc12a7,Jarid2,Epn2,Cnot4,Mbnl1,Nfat5,Zranb1,Pkig,Rac1
DBP_Q6	24	1,08	7,57E-06	Mcart1,Cpn1,Itgb3bp,Ganab,Ppp2r2a,Rere,Lrrfip2,Cald1,Nap1l5,Mxi1,Atp5b,Itpkb,Snx9,Syncrip,Gopc,Pum2,Dusp6,Jarid2,Smarca2,Srpk2,Mbnl1,Dhx30,Nfat5,Pura
FOXO3_01	23	1,03	8,26E-06	Scrn3,Tbl1x,Tjp1,Atf2,Cfl2,Pax6,Ttr,Brd8,Junb,Hibadh,Trim8,Ulk1,Meis2,Sqstm1,Enpp2,Sumo1,Fbxo9,Prkci,Mcm7,Hbp1,Mbnl1,Pura,Chchd7
E2F_Q6	22	0,99	8,85E-06	Ncl,Insm1,Nup153,Atf5,Kpnb1,Snrpd1,Phc1,Prpf4b,Ufd1l,Pax6,Sp3,Arhgap6,Mcm6,Dnmt1,Cbx5,Meis2,Topbp1,Dnajc9,Tmpo,Sumo1,Ctcf,Mcm7
HNF3ALPHA_Q6	22	0,99	8,85E-06	Gata6,Rreb1,Foxp1,Aldh9a1,Tpp2,Gcnt2,H2afv,Cald1,Gabarapl1,Tjp1,Elavl4,Itpkb,Pik3c2a,Foxa2,Gtf2a1,C8b,Pum2,Dusp6,Smarca2,Abi1,Pura,Chchd7
HFH4_01	33	1,48	8,99E-06	Gata6,Myo10,Insm1,Etv1,Fosb,Aldh9a1,Sin3a,H2afv,Tcf4,Ppap2b,Map3k3,Cald1,Gabarapl1,Tjp1,Cacna1d,Cpeb4,Scg3,Pik3c2a,Dusp1,Rad21,Trim8,Foxa2,Cpeb3,Enpp2,Asxl1,Ndrg1,Smarca2,Atp2a2,Nudt11,Pura,Rnf146,Ncam1,Chchd7
E2F_Q4	22	0,99	9,65E-06	Ncl,Insm1,Nup153,Atf5,Kpnb1,Snrpd1,Phc1,Ufd1l,Pax6,Sp3,Arhgap6,Mcm6,Dnmt1,Cbx5,Epc1,Meis2,Topbp1,Dnajc9,Tmpo,Sumo1,Ctcf,Mcm7
IK3_01	22	0,99	9,65E-06	Arpc2,Lifr,Ppap2b,Baz2b,Hectd1,Hivep1,Gabbr1,Wsb2,Ace2,Esm1,Ank3,Timp3,Mecp2,Mtss1,Rbx1,Serpini1,Dhrs4,Vamp3,Xpo1,Dcx,Egr1,Pura
EGR2_01	20	0,90	9,73E-06	Pabpc4,Smad1,Colec12,Hnrpdl,Nkx2-2,Sin3a,Itpr1,Rere,Map3k3,Pkd1,Klf5,Syncrip,Mnt,Atp2a2,Gltscr2,Sec14l1,Egr1,Pdcd6ip,Rrbp1,Rbbp6
PEA3_Q6	23	1,03	9,74E-06	Mfn2,Sec24c,Wasf2,Extl2,Elavl4,Itpkb,Tceb3,Pax6,Ank3,Trim8,Numa1,Nrp1,Epc1,Ndufs2,Sf1,Acsl5,Ctbp2,Cap1,Hhex,Hipk1,Zranb1,Ncam1,Pum1
HFH1_01	24	1,08	9,77E-06	Rreb1,Smad1,Itgb3bp,Eif4ebp2,Elf1,Cacna1d,Hhip,Scg3,Pik3c2a,Dusp1,Rad21,Esm1,Trim8,Nrp1,Gtf2a1,Taf10,Slc38a2,Phf8,Cd2bp2,Sumo1,Hhex,Atp2a2,Pura,Ncam1
CEBP_C	21	0,94	1,14E-05	Etv1,Acvr1,Ptpn12,Nup153,Sars,Foxp1,Etf1,Rlf,Rere,Nosip,Nfx1,Pik3c2a,Rad23b,Klf5,Numa1,Aldoa,Neurod1,Pik3r1,Acsl5,Rps19,Ctgf

Transcription factor	*Gene Count*	*Percentage*	*p-Value*	*Genes induced in RasGrf1 KO pancreatic islets*
EFC_Q6	25	1,12	1,14E-05	Rreb1,Strn4,Ywhag,Fosb,Tm9sf1,Sin3a,Ywhae,Phtf1,Nono,Cacna1d,Psmf1,Elavl4,Pcyt1a,Arid1a,Pax6,Ccnd2,Stat5b,Cbx6,Slc4a2,Meis2,Gbf1,Slk,Dhx30,Hs2st1,Ube2l3
E2F_02	22	0,99	1,15E-05	Ncl,Peg3,Cdc5l,Atf5,H2afv,Snrpd1,Mapk6,Phc1,Prpf4b,Pax6,Sp3,Arhgap6,Mcm6,Syncrip,Dnmt1,Topbp1,Dnajc9,Tmpo,Sumo1,Plagl1,Mcm7,Dlg3
E2F1DP1_01	22	0,99	1,15E-05	Ncl,Peg3,Cdc5l,Atf5,H2afv,Snrpd1,Mapk6,Phc1,Prpf4b,Pax6,Sp3,Arhgap6,Mcm6,Syncrip,Dnmt1,Topbp1,Dnajc9,Tmpo,Sumo1,Plagl1,Mcm7,Dlg3
E2F4DP2_01	22	0,99	1,15E-05	Ncl,Peg3,Cdc5l,Atf5,H2afv,Snrpd1,Mapk6,Phc1,Prpf4b,Pax6,Sp3,Arhgap6,Mcm6,Syncrip,Dnmt1,Topbp1,Dnajc9,Tmpo,Sumo1,Plagl1,Mcm7,Dlg3
CREL_01	25	1,12	1,22E-05	Arpc2,Etv1,Ywhaz,Clock,Sin3a,Eif5a,Taz,Mapk6,Baz2b,Gng4,Hivep1,Elavl4,Rab34,Sec63,Ube2d3,Pcsk2,Blcap,Smyd2,Tpm3,Creb1,Rps19,Dusp6,Slc12a2,Nfat5,Ctgf
E2F1DP2_01	22	0,99	1,25E-05	Ncl,Peg3,Cdc5l,Atf5,H2afv,Snrpd1,Mapk6,Phc1,Prpf4b,Pax6,Sp3,Arhgap6,Mcm6,Syncrip,Dnmt1,Topbp1,Dnajc9,Tmpo,Sumo1,Plagl1,Mcm7,Dlg3
GATA2_01	15	0,67	1,25E-05	Fkbp2,Kpnb1,Ppp2r2a,Foxa3,Ywhae,Pafah1b1,Rab34,Tra2a,Sp3,Syncrip,Foxa2,Blcap,Hdac3,Znrf2,Fbxw11
AREB6_04	24	1,08	1,25E-05	Gata6,Spg20,H2afv,Tcf4,M6pr,Nfkb1,Tjp1,Mxi1,Cdc37l1,Elavl4,Mafb,Hnf4a,Klf5,Junb,Purb,Foxa2,Son,Meis2,Dusp6,Xpo1,Vamp4,Smarca2,Rasgrf1,Ctgf
ER_Q6	25	1,12	1,31E-05	Lpl,Clock,Mapk6,Mxi1,Psmf1,Elavl4,Myadm,Rcor1,Mll5,Dnmt3a,Ythdf3,Aldoa,Cpeb3,Slc38a2,Atp6v1a,Invs,Tpm3,Glg1,Dcx,Rb1cc1,Satb1,Rasgrf1,Ubqln1,Nudt4,Rock2
SREBP1_Q6	24	1,08	1,35E-05	Kpnb1,Mtmr3,Clasp1,Rhoa,Rab10,Cacna1d,Pias1,Mll5,Lasp1,Rps10,Baz2a,Ldlr,Eno2,Eif4g2,Atp6v1a,Kif1b,Srebf2,Nsf,Immt,Rbx1,Serpini1,Nbea,Nfat5,Macf1
NFAT_Q6	23	1,03	1,36E-05	Elk3,Acvr1,Pcbp2,Foxp1,Rhoa,Rbm14,Tcta,Cfl2,Cpeb4,Marcks,Lasp1,Sp3,Arhgap6,Ankrd17,Mast2,Lims1,Ptov1,Nbea,Anp32e,Jarid2,Ctnnd1,Tnfaip1,Mtif2
P300_01	23	1,03	1,36E-05	Foxp1,Ppp3ca,Rere,Ptbp2,Elavl4,Junb,Syncrip,Tusc3,Blcap,Irak1,Jund,Hmgcs1,Pum2,Srebf2,Map3k4,Kif3b,Serpini1,Ctbp2,Ikbkb,Jarid2,Prkag2,Canx,Srpk2
SF1_Q6	29	1,30	1,39E-05	Pabpc4,Rreb1,Pank1,Stard5,Dner,Thrap3,Kpna6,Abat,Gabarapl1,Tbc1d15,Atp5b,Fbxo21,Hspd1,Cs,Drap1,Ywhah,Kcnmb2,Aldoa,Blcap,Atp1b1,Ubl3,Immt,Gabpa,Mnt,Epn2,Rhot1,Vamp2,Plekha1,Zranb1
NFY_Q6	23	1,03	1,49E-05	Wasf2,Ganab,Gnb2,Cox6a2,Nav2,M6pr,Rhoa,Tcta,Gca,Elavl4,Nfyc,Ppm1b,Numb,Pax6,Racgap1,Vcp,Son,Ctbp2,Abca7,Jarid2,Atrx,Mbnl1,Dlg3
NFKB_C	25	1,12	1,52E-05	Dap3,Nup153,Sin3a,Foxa3,Ppp3ca,Eif5a,Lrrfip2,Rhoa,Taz,Tcta,Smarcad1,Rab10,Gng4,Dach2,Lasp1,Ptprj,Pigc,Hcfc1,Pcsk2,Mnt,Gphn,Gna13,Top1,Nfat5,Ctgf
GATA1_05	25	1,12	1,52E-05	Gata6,Syne2,Rreb1,Etv1,Cldn7,Ptpn12,Rabep1,Psma6,Nr3c1,Sin3a,Cdh2,Ptprr,Elavl4,Cpeb4,Klf5,Eno2,Ubl3,Pum2,Usp47,Hint1,Ctcf,Plagl1,Srpk2,Plekha1,Tgfb2
E2F_Q4_01	22	0,99	1,63E-05	Luc7l2,Casp2,Ncl,Peg3,Ncoa6,Kpnb1,Eif5a,Ufd1l,Klf5,Mcm6,Dnmt1,Cbx5,Meis2,Topbp1,Rps19,Anp32e,Top1,Pik3r4,Ctcf,Fbxo9,Mcm7,Slbp
STAT1_02	22	0,99	1,79E-05	Cpt2,Foxa3,Agl,Csnk1a1,Bclaf1,Set,Snx9,Hspd1,Nucb1,Itm2c,Cs,Pax6,Tlk2,Pigc,Bet1,Ubl3,Inadl,Ccni,Stx6,Ube2n,Cap1,Hhex
IK2_01	25	1,12	1,96E-05	Etv1,Itm2b,Ptpn12,Gnb2,Kpnb1,Ppap2b,Cdkn1b,Hivep1,Ace2,Calu,Pias1,Folr1,Ank3,Atp6v0d1,Adcy6,Smyd2,Hdac3,Vamp3,Jarid2,Gdi1,Pak3,Vip,Tes,Ets1,Ctgf

Transcription factor	*Gene Count*	*Percentage*	*p-Value*	*Genes induced in RasGrf1 KO pancreatic islets*
AP2ALPHA_01	22	0,99	1,96E-05	Pabpc4,Luc7l2,Cldn7,Hnrpdl,Pabpc1,Nr3c1,Atf5,Sp4,Mafb,Arid1a,Tlk2,Sp3,Ythdf2,Jund,Cbx6,P4ha1,Jarid2,Ctcf,Znrf2,Pak3,Egln2,Nfat5
AHRARNT_02	6	0,27	2,76E-05	Mapre1,Hdac3,Cbx6,Hint1,Meis2,Egr1
E2F1DP1RB_01	21	0,94	2,91E-05	Ncl,Peg3,Atf5,Kpnb1,Snrpd1,Mapk6,Phc1,Pax6,Sp3,Arhgap6,Mcm6,Dnmt1,Cbx5,Meis2,Topbp1,Dnajc9,Tmpo,Sumo1,Ctcf,Atrx,Mcm7
E2F1_Q6	21	0,94	3,19E-05	Ncl,Cdc5l,Atf5,Kpnb1,H2afv,Snrpd1,Phc1,Prpf4b,Pax6,Klf5,Sp3,Arhgap6,Mcm6,Syncrip,Dnmt1,Topbp1,Dnajc9,Tmpo,Sumo1,Ctcf,Mcm7
GR_Q6_01	23	1,03	3,28E-05	Rreb1,Terf2,Khdrbs1,Cdh2,Npepps,Cald1,Cdkn1b,Elavl4,Dhx40,Rcn1,Ppm1b,Pdap1,Lasp1,Atp8a1,Ank3,Syncrip,Bach1,Slc38a2,Eif4g2,Rbx1,Sec24d,Trip11,Ets1
HNF4_DR1_Q3	23	1,03	3,28E-05	Fam20c,Rreb1,Senp2,Eif4ebp2,Ckap4,Foxa3,Gtf2i,Golga4,Lman2,Psmf1,Hspd1,Ttr,Tlk2,Lamp2,Hibadh,Ahcyl1,Atp1b1,Mtmr4,Dctn2,Gbf1,Asxl1,Ush1c,Pura
SP1_Q2_01	23	1,03	3,57E-05	Rab2b,Ncoa6,Cdh2,Cacna1d,Kcnb1,Trim28,Lasp1,Ptprj,Fbxo3,Sp3,Dnmt3a,Bcl7c,Enah,Creb1,Chka,Egln2,Vamp2,Atp2a2,Efemp2,Hipk1,Nfat5,Hs2st1,Tcf12
OCT_C	34	1,52	3,69E-05	Strn4,Etv1,Lpl,Bace2,Itgb3bp,Foxp1,Nkx2-2,Gcnt2,Ube2s,Rhob,Tcf4,Cryzl1,Nap1l5,Nono,Ywhab,Pax6,Ank3,Irak1,Poldip3,Jund,Cnot2,Epc1,Srebf2,Ing1,Cul3,Csda,Top1,Pten,Jarid2,Asxl1,Hist3h2a,Egln2,Fbxl14,Cbfa2t2
OCT1_04	22	0,99	3,69E-05	Etv1,Itm2b,Foxp1,Cacna1d,Elavl4,Cpeb4,Pax6,Ank3,Ankrd17,Irak1,Cnot2,Nsf,Ncald,Topbp1,Mnt,Pten,Gdi1,Plagl1,Smarca2,Mbnl1,Pura,Ncam1
USF2_Q6	22	0,99	3,69E-05	Pabpc4,Sec23ip,Adss,Polr3c,Rlf,Atp6v0b,Pax6,Eif4b,Zzz3,Slc38a2,Cbx5,Epc1,Stx6,Csda,Ctbp2,Bcl9,Ptprf,Hhex,Ilk,Dpagt1,Ap3m1,Tgfb2
PBX1_02	16	0,72	3,76E-05	Smad1,Khdrbs1,Rhob,Tbl1x,Nosip,Ccnd2,Sp3,Hibadh,Usp47,Mtss1,Meis2,Sst,Cnot7,Bcl9,Hhex,Wdr1
GR_Q6	23	1,03	3,82E-05	Pabpc4,Insm1,Wasf2,Mospd2,Lifr,Dlg5,Rere,Elavl4,Rcn1,Klf5,Syncrip,Adcy6,Slc38a2,Got2,Klhl7,Meis2,Trip11,Plagl1,Mcm7,Srpk2,Mbnl1,Ncam1,Ppm1a
AHR_01	11	0,49	3,86E-05	Nr3c1,Pcbp2,Pank3,Kpnb1,Itpr1,Atf2,Mnt,Vamp3,Fbxw11,Egr1,Ncam1
MEF2_Q6_01	23	1,03	4,13E-05	Pabpc4,Etv1,Foxp1,Tpp2,Ppp2r2a,Rhob,Rere,Fxr1,Crtap,Elavl4,Ppm1b,Rcor1,Kpna3,Dnaja4,Asph,Adcy6,Aldoa,Ctbp2,Atp2a3,Smarca2,Rasgrf1,Hipk1,Hipk3
TATA_C	26	1,17	4,56E-05	Lpl,Pank1,Gch1,Tgfbr1,Foxp1,Tcf4,Rhoa,Tcta,Hivep1,Cfl2,Eef2,Wsb2,Mapre1,Bach1,Jund,Neurod1,Epc1,Invs,Topbp1,Slc12a2,Jarid2,Satb1,Vip,Srpk2,Tgfb2,Pura
CIZ_01	21	0,94	4,90E-05	Ywhag,Nkx2-2,Ckap4,Tulp4,Prickle1,Tcf4,Tbl1x,Hivep1,Ccnd2,Esm1,Lamp2,Pitpnc1,Meis2,Psmd11,Tmpo,Bcl9,Smarca2,Mbnl1,Pura,Ncam1,Chchd7
GR_01	19	0,85	5,53E-05	G3bp2,Khdrbs1,Rere,Hivep1,Dstn,Elavl4,Rcn1,Cpeb4,Klf5,Arhgap6,Dnajc3,Syncrip,Adcy6,Vcp,Pum2,Rbx1,Enpp2,Tcf12,Ets1
LHX3_01	26	1,17	5,67E-05	Rreb1,Suclg2,Stx7,Bace2,Pik3ca,Nono,Mllt10,Hhip,Gtpbp1,Elp4,Marcks,Junb,Rab22a,Nrp1,Pcsk2,Clk2,Myst4,Pitpnc1,Mtss1,Gna13,Robo1,Dcx,Vil1,Srpk2,Mbnl1,Pura
NKX25_02	23	1,03	5,69E-05	Gad1,Ppap2b,Tbl1x,Nap1l5,Flt1,Cfl2,Hhip,Ube2e2,Ank3,Pvrl3,Meis2,Gna13,Rnf11,Trpm7,Dcx,Egln1,Fbxw11,Vip,Ubqln1,Tcf12,Vldlr,Chchd7,Dlg3

Transcription factor	*Gene Count*	*Percentage*	*p-Value*	*Genes induced in RasGrf1 KO pancreatic islets*
CEBP_01	23	1,03	5,69E-05	Syne2,Nr3c1,Foxp1,Nkx2-2,Fosb,Gcnt2,Dock9,Tbl1x,Cpeb4,Tra2a,Cs,Kpna3,Arhgap6,Neurod1,Mtss1,Mnt,Dapk1,Top1,Jarid2,Atrx,Hbp1,Pura,Chchd7
STAT5A_02	15	0,67	5,94E-05	Foxp1,Foxa3,Mat1a,Cdc37l1,Ccnd2,Dars,Syncrip,Pcsk2,Zfr,Pik3r1,Gabpa,Enpp2,Asxl1,Vip,Mbnl1
LFA1_Q6	22	0,99	6,00E-05	Bace1,Etv1,Fkbp2,Gad1,Pcbp2,Creb3l2,M6pr,Npc2,Flt1,Ccnl1,Arid1a,Anxa4,Ubl3,Dpf2,Ndufs2,Mgea5,Immt,Mnt,Asxl1,Nfat5,Rbbp6,Ctgf
CREBP1_01	17	0,76	7,05E-05	Csnk1e,Calm1,Ywhag,Clasp1,Chic2,Elavl4,Eef2,Gnpnat1,Fbxo3,Trim8,Pcsk2,Mecp2,Irak1,Srebf2,Epn2,Sumo1,Zfp161
RSRFC4_Q2	36	1,61	7,18E-05	Pabpc4,Nnt,Ncor1,Pank1,Itgb3bp,Pcbp2,Foxp1,Tpp2,Ppp2r2a,Tcf4,Eif5a,Fxr1,Crtap,Elavl4,Cfl2,Tob2,Cpeb4,Rcor1,Tlk2,Kpna3,Gcg,Hibadh,Trim8,Dnaja4,Asph,Aldoa,Usp47,Cul3,Mrps23,Tpm3,Atp2a3,Smarca2,Fbxw11,Rasgrf1,Nfat5,Pura
CACCCBINDINGFACTOR_Q6	23	1,03	7,18E-05	Calm1,G3bp2,Fkbp2,Gnb2,Sp4,Ywhae,Eif5a,Tbl1x,Diablo,Pla2g6,Rad23b,Ptprj,Ythdf2,Tusc3,Dld,Stk16,Brd2,Jund,Mtss1,Mnt,Xpo1,Jarid2,Efemp2
STAT3_02	16	0,72	7,33E-05	Ddit3,Fosb,Nav2,Tm9sf1,Kpnb1,Eif5a,Set,Calu,Meis2,Mnt,Ap2b1,Gphn,Slc35a5,Egr1,Ncam1,Ctgf
ICSBP_Q6	22	0,99	7,58E-05	Gata6,Syne2,Nkx2-2,Col4a1,Eif5a,Itpr1,Nfkb1,Mxi1,Hivep1,Cpeb4,Rcor1,Ndrg3,Syncrip,Irak1,Meis2,Ppp2r5c,Top1,Enpp2,Slc12a7,Ptk2,Pura,Rrbp1
P53_02	22	0,99	7,58E-05	Elk3,Etv1,Gad1,Tfam,Ddit3,Gcnt2,Cald1,Lztr1,Atp6ap1,Cramp1l,Eif4g2,Ndufs2,Pitpnc1,Meis2,Acsl5,Rps19,Enpp2,Hipk1,Mbnl1,Dhx30,Usp48,Lpp
PIT1_Q6	21	0,94	8,01E-05	Acvr1,Psmd1,Lrrfip2,Fxr1,Hivep1,Elavl4,Hhip,Cpeb4,Marcks,Pax6,Junb,Nrp1,Usp9x,Atp1b1,Mrps23,Meis2,Dock7,Vamp3,Dusp6,Gdi1,Pura
PR_Q2	22	0,99	8,87E-05	Pank1,Bace2,Syp,Ppp2r2a,Rere,Cdkn1b,Mxi1,Hivep1,Nek9,Rcn1,Cpeb4,Rwdd1,Ctdspl,Brd4,Cnot2,Hmgcs1,Kcnip1,Bcl9,Znrf2,Tcf12,Dlg3,Ctgf
RORA1_01	22	0,99	8,87E-05	Abat,Gabarapl1,Extl2,Elavl4,Ap3d1,Hspd1,Rcor1,Ythdf2,Asph,Nrp1,Stk38,Atp6v1a,Ubl3,Tpm3,Creb1,Jarid2,Mpp3,Bcl9,Vamp2,Satb1,Zranb1,Psip1
MYCMAX_01	21	0,94	9,43E-05	Ncl,Suclg2,Etv1,Hnrpdl,Pabpc1,Cad,Diablo,Hhip,Ewsr1,Syncrip,Bcl7c,Neurod1,Cbx5,Mnt,Rps19,Top1,Asxl1,Mpp3,Tgfb2,Mthfd1,Snx5
LXR_DR4_Q3	11	0,49	1,06E-04	Ywhaz,Fkbp2,Foxp1,Gnb2,Mafb,Pias1,Pum2,Ndufs2,Jarid2,Ctcf,Rb1cc1
STAT6_01	22	0,99	1,12E-04	Man2c1,Lpl,Psma6,Tcf4,Traf3,Wac,Mapk6,Ppm1b,Dach2,Marcks,Pcyox1,Slc38a2,Cnot2,N4bp1,Ipo4,Mtss1,Nfe2l2,Fbxo9,Atp2a2,Satb1,Vip,Plekha1
AP3_Q6	22	0,99	1,12E-04	Smad1,Etv1,Gad1,Nmt2,Npc2,Cald1,Tbk1,Mllt10,Hivep1,Tra2a,Trim8,Nrp1,Jund,Dpf2,Rapgef4,Meis2,Gas2,Jarid2,Ctnnd1,Hdlbp,Mbnl1,Anxa6
FREAC7_01	18	0,81	1,26E-04	Csnk1e,Mcart1,Elk3,Rhob,Taz,Cpeb4,Pik3c2a,Pias1,Junb,Trim8,Dnaja4,Gtf2a1,Vcp,Cbx6,Ctnnal1,Tgfb2,Chchd7,Ctgf
NCX_01	17	0,76	1,26E-04	Ssr1,Etv1,Nup153,Peg3,Gng4,Elavl4,Mll5,Arhgap6,Tnpo3,Rnf11,Dnajc7,Ctcf,Plagl1,Dcx,Appbp2,Hbp1,Chchd7
SP3_Q3	21	0,94	1,28E-04	Sec24c,Pabpc1,Foxp1,Ywhae,Flt1,Rab34,Junb,Sp3,Arhgap6,Ankrd17,Lamp2,Dnmt3a,Cpeb3,Hdac3,Slc4a2,Kdelr1,Pbx2,Dnajc7,Hhex,Pura,Chchd7
NKX62_Q2	21	0,94	1,28E-04	Stx7,Bace2,Lgals12,Nkx2-2,Rplp0,Cald1,Cyfip2,Elavl4,Marcks,Irak1,Stk38,Myst4,Cap1,Atp2a3,Xpo1,Smarca2,Atp2a2,Cldn4,Actr10,Ets1,Chchd7

Transcription factor	*Gene Count*	*Percentage*	*p-Value*	*Genes induced in RasGrf1 KO pancreatic islets*
TEF_Q6	21	0,94	1,28E-04	Csnk1e,Foxp1,Lmo7,Ube2s,Abcb7,Cacna1d,Gnpnat1,Mll5,Pax6,Esm1,Trim8,Nrp1,Taf10,Cbx6,Pum2,Myst4,Atp2a3,Robo1,Ctcf,Rb1cc1, Pura
FAC1_01	19	0,85	1,32E-04	Wasf2,Foxp1,Nkx2-2,Mtmr3,Csnk1a1,Cacna1d,Junb,Cbfb,Ccng2,Foxa2,Gtf2a1,Gbf1,Mnt,Robo1,Jarid2,Smarca2,Cnot4,Hipk1,Rbbp6
TAL1BETAITF2_01	21	0,94	1,38E-04	Nap1l1,Strn4,Foxp1,Ddit3,Fosb,Hectd1,Riok3,Elavl4,Scgn,Arpc1a,Atp8a1,Cd81,Dars,Brd4,Trim37,Serpini1,Dapk1,Dcx,Pak3,Tcf12,Macf 1
GATA4_Q3	21	0,94	1,38E-04	Ywhag,Lifr,Tpt1,Hhip,Cpeb4,Asb3,Smarce1,Nrp1,Gtf2a1,Hint1,Btg2,Sf1,Meis2,Ppp2r5c,Pip5k1b,Jarid2,Ctnnd1,Mbnl1,Pura,Ets1,Chchd 7
E2F1_Q3	20	0,90	1,47E-04	Ncl,Gch1,Peg3,Atf5,Snrpd1,Phc1,Pias1,Lasp1,Pax6,Eif4b,Klf5,Sp3,Arhgap6,Mcm6,Dnmt1,Topbp1,Dnajc9,Tmpo,Sumo1,Mcm7
E2F4DP1_01	20	0,90	1,47E-04	Ncl,Peg3,Cdc5l,Atf5,H2afv,Snrpd1,Mapk6,Phc1,Prpf4b,Pax6,Sp3,Arhgap6,Mcm6,Syncrip,Dnmt1,Topbp1,Dnajc9,Tmpo,Sumo1,Mcm7
EVI1_04	21	0,94	1,48E-04	Csnk1e,Rhob,Clasp1,Ppap2b,Rere,Elavl4,Rcor1,Klf5,Sp3,Arhgap6,Dnaja4,Mrps23,Ipo4,Meis2,Vamp3,Gdi1,Mbnl1,Tgfb2,Eif5,Chchd7,Hi pk3
BRN2_01	21	0,94	1,48E-04	Itgb3bp,Clock,Tcf4,Taz,Extl2,Diablo,Hivep1,Ppm1b,Cs,Cbfb,Kcnmb2,Ubl3,Immt,Mdm1,Vamp3,Dusp6,Bcl9,Dcx,Mbnl1,Rit1,Nfat5
MYB_Q3	20	0,90	1,58E-04	Ndufc1,Rreb1,Foxp1,Ddit3,Prickle1,Ywhae,Trim2,Mll5,Pdap1,Sf3b1,Klf5,Ankrd17,Tnpo3,Aldoa,Ndel1,Cnot2,Gphn,Xpo1,Canx,Hhex
HMGIY_Q6	21	0,94	1,59E-04	Insm1,Gad1,Foxp1,Prickle1,Ptbp2,Wac,Taz,Mapk6,Pafah1b1,Rab34,Ppm1b,Klf5,Esm1,Ube2e2,Vamp3,Dusp6,Scamp1,Atp1b3,Ctnnd1, Mbnl1,Ncam1
NKX22_01	17	0,76	1,62E-04	Gata6,Armcx2,Foxp1,Nkx2-2,Itpr1,Tbrg1,Mapk6,Arpc1a,Cpeb4,Tra2a,Stat5b,Aebp2,Pik3r1,Hhex,Srpk2,Zranb1,Cct7
TFIII_Q6	19	0,85	1,81E-04	Ncoa6,Nkx2-2,Spred2,Kpnb1,Sin3a,Pafah1b1,Mafb,Ccnd2,Baz2a,Foxa2,Strap,Sf1,Map4k3,Xpo1,Appbp2,Tcf12,Cd44,Cbfa2t2,Mmd
E2F_Q3	19	0,85	1,81E-04	Ncl,Insm1,Nup153,Gch1,Peg3,Kpnb1,Pias1,Ufd1l,Lasp1,Klf5,Arhgap6,Mcm6,Dnmt1,Cbx5,Meis2,Topbp1,Ctcf,Atrx,Mcm7
RFX1_01	20	0,90	1,84E-04	Ids,E2f5,Prickle1,Hectd1,Psmf1,Cfl2,Piga,Pax6,Tusc3,Brd2,Hmgcs1,Gbf1,Pik3r4,Dpagt1,Ubb,Zranb1,Hs2st1,Pura,Tcf12,Bbs2
AP2REP_01	17	0,76	2,14E-04	Acvr1,Pank1,Atp2b2,Ywhag,Gnb2,Eif5a,Cald1,Arid1a,Ube2e2,Arhgap6,Dnmt3a,Brd4,Irak1,Phyhipl,Nudt11,Bcl2l11,Pum1
FOXD3_01	19	0,85	2,31E-04	Rreb1,Smad1,Ywhag,Ckap4,E2f5,Cald1,Hhip,Scg3,Pik3c2a,Marcks,Mll5,Hnf4a,Junb,Nrp1,Hhex,Atp2a2,Fgfr1op2,Tgfb2,Chchd7
STAT5A_03	21	0,94	2,31E-04	Lpl,Psma6,Polr3c,Tcf4,Traf3,Flt1,Dach2,Marcks,Pcyox1,Cnot2,N4bp1,Tpm3,Mtss1,Dctn2,Nfe2l2,Fbxo9,Atp2a2,Satb1,Vip,Plekha1,Usp4 8
TFIIA_Q6	22	0,99	2,42E-04	Phpt1,Ywhaz,Ckap4,Fosb,Rnf44,Trim28,Cs,Pax6,Syncrip,Elavl1,Rod1,Cbx6,Itch,Meis2,Dnajc7,Mcm7,Vamp2,Atp2a2,Srpk2,Rock2,Egr1,E if5
IK1_01	22	0,99	2,59E-04	Arpc2,Itm2b,Lifr,Foxp1,Gnb2,Sp4,Ppap2b,Itpr1,Rab10,Baz2b,Hivep1,Pias1,Arid1a,Kpna3,Fbxo3,Smyd2,Atp1b1,Dhrs4,Top1,Vamp3,Tes, Pole3
AREB6_02	21	0,94	2,87E-04	Cldn7,Armcx2,Cox6a2,Ocln,Uchl5,Flnb,Atf2,Hnf4a,Myo18a,Dnmt3a,Wbp2,Tdrd3,Hmgcs1,Dnajc13,Mtmr4,Pik3r1,Nbea,Pcp4,Epn2,Ctcf, Usp48
POU3F2_02	21	0,94	3,08E-04	Rreb1,Strn4,Foxp1,Cryzl1,Gtpbp1,Pik3c2a,Irak1,Ing1,Mtmr4,Pik3r1,Mdm1,Gas2,Top1,Pten,Gdi1,Asxl1,Smarca2,Fbxw11,Satb1,Ubb,Ch chd7
MYOD_01	20	0,90	3,13E-04	Etv1,Prickle1,Cryzl1,Taz,Kcnb1,Elavl4,Polr1d,Dars,Wbp2,Ube2d3,Bach1,Trim37,Invs,Calb1,Egln1,Zranb1,Egr1,Psip1,Pak2,Cbfa2t2
SREBP_Q3	20	0,90	3,13E-04	Peg3,Gnb2,Kpnb1,Srpr,Rhoa,Dnajc1,Rab10,Cacna1d,Set,Eif5b,Mll5,Elovl5,Baz2a,Blcap,Ldlr,Eif4g2,Srebf2,Serpini1,Wdr13,Hipk1

Transcription factor	*Gene Count*	*Percentage*	*p-Value*	*Genes induced in RasGrf1 KO pancreatic islets*
HNF3B_01	19	0,85	3,14E-04	Mcart1,Sin3a,Tbl1x,Cald1,Baz2b,Rab3ip,Itpkb,Cpeb4,Pik3c2a,Dusp1,Ttr,Dnaja4,Foxa2,Gtf2a1,Ctnnal1,Atp2a3,Rbp4,Chchd7,Lpp
RORA2_01	15	0,67	3,15E-04	Clock,Nmt2,Eif5a,Abat,Inpp5a,Ap3d1,Marcks,Rcor1,Asph,Mgea5,Creb1,Meis2,Gas2,Ube2l3,Cirbp
SRF_01	9	0,40	3,28E-04	Fosb,Cald1,Taz,Actr3,Junb,Cap1,Wdr1,Egr1,Anxa6
TAL1ALPHAE47_01	20	0,90	3,33E-04	Acvr1,Foxp1,Ddit3,Cdh2,Hectd1,Riok3,Elavl4,Scgn,Arpc1a,Mll5,Dars,Brd4,Trim37,Amph,Dapk1,Dcx,Fbxw11,Tcf12,Ogt,Macf1
PR_01	14	0,63	3,41E-04	Khdrbs1,Hivep1,Dstn,Elavl4,Rcn1,Rwdd1,Arhgap6,Syncrip,Adcy6,Fbxo9,Vamp2,Ets1,Chchd7,Dlg3
FREAC3_01	20	0,90	3,57E-04	Scrn3,Luc7l2,Etv1,Eif4ebp2,Xpo7,Traf3,Cald1,Rab3ip,Rpn2,Ttr,Gcg,Trim8,Ulk1,Meis2,Sqstm1,Bcl9,Prpf39,Dcx,Mcm7,Pura
LEF1_Q6	22	0,99	3,57E-04	Elk3,Insm1,Lifr,Pank3,Khdrbs1,Rnf44,Clasp1,Arih1,Suox,Hectd1,Hivep1,Phc1,Dhx40,Mafb,Arid1a,Ptprj,Syncrip,Bcl7c,Jund,Cnot7,Dcx,Cldn4
SREBP1_02	11	0,49	3,66E-04	Atp2b2,Gnb2,Ywhae,Eif5a,Mll5,Elovl5,Baz2a,Jund,Srebf2,Wdr13,Cd2ap
IRF1_01	20	0,90	4,11E-04	Gata6,Syne2,Mgat2,Ywhag,Ganab,Ddit3,Col4a1,Dock9,Ptprr,Mxi1,Elavl4,Itpkb,Tlk2,Kpna3,Trim8,Creb1,Vamp3,Rock1,Ncoa1,Rit1
MSX1_01	17	0,76	4,16E-04	Calm1,Rreb1,Smad1,Gad1,Foxp1,Nkx2-2,Bnip3l,Lims1,Aig1,Cbx5,Kif1b,Slc4a2,Gna13,Cap1,Cherp,Kctd5,Cbfa2t2
HNF4ALPHA_Q6	21	0,94	4,24E-04	Mcart1,Rreb1,Cdk8,Mxi1,Golga4,Prpf4b,Pax6,Ttr,Nrp1,N4bp1,Rapgef4,Rbpms,Son,Gbf1,Dock7,Plagl1,Hhex,Satb1,Rab3d,Pura,Chchd7
STAT5A_01	20	0,90	4,39E-04	Sec24c,Foxa3,Flt1,Bclaf1,Cacna1d,Phc1,Ank3,Nrp1,Pcsk2,Mecp2,Bet1,Ndufs2,Ncald,Smpd1,Enpp2,Asxl1,Plagl1,Atp2a2,Vip,Pura
TBP_01	20	0,90	4,71E-04	Csnk1e,Nnt,Ncor1,Bicd1,Pank1,Itgb3bp,Ppp2r2a,Cfl2,Cpeb4,Rcor1,Kpna3,Gcg,Dnaja4,Usp47,Cul3,Ctnnal1,Rps19,Ctcf,Fbxw11,Rit1
COUP_01	20	0,90	5,03E-04	Fam20c,Creb3l2,Foxa3,Mxi1,Golga4,Mllt10,Pax6,Baz2a,Dnmt3a,Nrp1,Aldoa,Pitpnc1,Invs,Mtmr4,Gbf1,Asxl1,Satb1,Pura,Rrbp1,Rbbp6
T3R_Q6	20	0,90	5,03E-04	Mtmr3,Rere,Mxi1,Pax6,Junb,Rab22a,Baz2a,Ythdf3,Atf6,Dusp16,Mtmr4,Acsl5,Mnt,Hipk2,Rhot1,Bcl9,Dcx,Mcm7,Vamp2,Ogt
RP58_01	19	0,85	5,17E-04	Calm1,Colec12,Acvr1,Nr3c1,Foxp1,Nkx2-2,Creb3l2,Spred2,Prickle1,Ppap2b,Scgn,Ppm1b,Klf5,Trim37,Dapk1,Atp2a2,Dhx30,Tcf12,Dlg3
HFH8_01	18	0,81	5,25E-04	Smad1,Etv1,Xpo7,Elf1,Rasd1,Itpkb,Cpeb4,Scg3,Marcks,Dusp1,Lasp1,Rad21,Gtf2a1,Cbx6,Scamp1,Sumo1,Asxl1,Pura
GATA6_01	20	0,90	5,36E-04	Gata6,Itgb3bp,Foxp1,Cdh2,Itpr1,Iqgap1,Elavl4,Cs,Adcy6,Lims1,Eno2,Pum2,Rbpms,Btg2,Sfrp5,Pip5k1b,Atp2a3,Jarid2,Ctcf,Dcx
STAT1_03	19	0,85	5,52E-04	Sin3a,Foxa3,Csnk1a1,Bclaf1,Gng4,Snx9,Hspd1,Nucb1,Itm2c,Cs,Pax6,Tlk2,Bet1,Ubl3,Ccni,Stx6,Ube2n,Hhex,Fbxw11
HIF1_Q3	18	0,81	6,08E-04	Insm1,Pabpc1,Nav2,Rab10,Golga4,Mll5,Ccnd2,Asb3,Zzz3,Ero1l,Itch,Pten,Atp1b3,Ptprf,Scamp2,Ap3m1,Bcl2l11,Snx5
NKX61_01	19	0,85	6,37E-04	Ncor1,Myo10,Clock,Tcf4,Elp4,Marcks,Ccnd2,Trim8,Pcsk2,Clk2,Cul3,Fhl1,Vamp3,Dusp6,Ctnnd1,Atp2a2,Zranb1,Pura,Chchd7
CEBPGAMMA_Q6	20	0,90	6,50E-04	Pabpc1,Itgb3bp,Ywhag,Rplp0,Cdkn1b,Dhx40,Gnpnat1,Trim8,Smarce1,Srp68,Ncald,Ctnnal1,Sst,Ctbp2,Tmpo,Ptk2,Jarid2,Hs2st1,Pura,Eif5
OSF2_Q6	20	0,90	6,50E-04	Nkx2-2,Etf1,Sin3a,Tulp4,Clcn3,Tcf4,Mtmr3,Itpr1,Pafah1b1,Slc8a1,Dach2,Mll5,Neurod1,Srebf2,Mtmr4,Atp2a2,Hipk1,Tcf12,Ncam1,Akap8
AR_Q6	18	0,81	6,53E-04	Smad1,Nkx2-2,Sin3a,Camsap1,Tbl1x,Dhx40,Actr3,Syncrip,Ube2d3,Pum2,Tpm3,Ap2b1,Nbea,Dnajc7,Mbnl1,Pura,Rbbp6,Tm9sf2

Transcription factor	*Gene Count*	*Percentage*	*p-Value*	*Genes induced in RasGrf1 KO pancreatic islets*
IPF1_Q4	19	0,85	6,77E-04	Sytl4,Ppp2r2a,Nono,Elavl4,Atp6ap1,Tceb3,Rnf14,Syncrip,Nrp1,Epc1,Srebf2,Meis2,Entpd5,Smarca2,Rasgrf1,Mbnl1,Pura,Tcf12,Cbfa2t2
PXR_Q2	20	0,90	6,91E-04	Smad1,Fkbp2,Tob2,Gtpbp1,Dars,Kcnmb2,Nrp1,Aldoa,Neurod1,Atp1b1,Hmg20a,Map4k3,Meis2,Gphn,Gdi1,Bcl9,Plekha1,Pura,Macf1,Ctgf
E47_01	20	0,90	6,91E-04	Foxp1,Cdh2,Map3k3,Taz,Kcnb1,Elavl4,Mll5,Ccnd2,Cd47,Ube2d3,Trim8,Ttc13,Hdac3,Aig1,Slc4a2,Mtss1,Calb1,Pak3,Psip1,Macf1
E2F_Q6_01	18	0,81	6,91E-04	Luc7l2,Casp2,Ncl,Peg3,Nkx2-2,Kpnb1,Eif5a,Ufd1l,Klf5,Mcm6,Dnmt1,Cbx5,Topbp1,Rps19,Anp32e,Dusp6,Ctcf,Mcm7
CEBP_Q2_01	21	0,94	7,43E-04	Mospd2,Ywhag,Foxp1,Rhob,Clasp1,Herc4,Map3k3,Cald1,Cfl2,Klf5,Ube2e2,Adam9,Syncrip,Usp9x,Calr,Acsl5,Nfe2l2,Pten,Mbnl1,Tcf12,Fbxl14
STAT5A_04	16	0,72	7,59E-04	Arpc2,Nkx2-2,Ppp2r2a,Itpr1,Arih1,Phtf1,Nono,Wsb2,Brd4,Calr,Dpf2,Apbb2,Bcl9,Mbnl1,Ncam1,Ets1
HIF1_Q5	19	0,85	7,65E-04	Arpc2,Insm1,Pabpc1,Nav2,Kpnb1,Vapb,Mll5,Ptprj,Zzz3,Hdac3,Pitpnc1,P4ha1,Ero1l,Itch,Atp1b3,Ptprf,Bcl2l11,Wdr1,Snx5
HNF4_01_B	19	0,85	7,65E-04	Rhob,Galnt2,Rcn2,Gtf2i,Golga4,Hdgf,Tlk2,Baz2a,Lamp2,Glud1,Srebf2,Invs,Mtmr4,Acsl5,Dctn2,Gbf1,Asxl1,Ush1c,Tcf12
NFKB_Q6	20	0,90	7,78E-04	Dap3,Pabpc1,Sufu,Atf5,Sin3a,Baz2b,Lasp1,Ptprj,Hcfc1,Pcsk2,Atp1b1,Mnt,Top1,Scamp1,Xpo1,Atp1b3,Ilk,Satb1,Nfat5,Ctgf
NRSF_01	11	0,49	8,23E-04	Nefh,Cryba2,Atp2b2,Dner,Pcsk2,Sst,Calb1,Chka,Phyhipl,Rasgrf1,Vip
HFH3_01	17	0,76	8,71E-04	Csnk1e,Etv1,Tjp1,Elavl4,Rab3ip,Cpeb4,Scg3,Pik3c2a,Dusp1,Rad21,Myst4,Scamp1,Smarca2,Atp2a2,Fbxw11,Ncam1,Chchd7
POU1F1_Q6	18	0,81	9,74E-04	Gata6,Smad1,Bace2,Zhx1,Cyfip2,Cacna1d,Hivep1,Elavl4,Gtpbp1,Pias1,Ank3,Nrp1,Dicer1,Iars,Meis2,Dock7,Dcx,Pura
CACBINDINGPROTEIN_Q6	18	0,81	9,74E-04	Arpc2,Kpnb1,Itpr1,Cacna1d,Lasp1,Mthfr,Foxa2,Ivd,Cbx6,Epc1,Ndufs2,Strn3,Dctn2,Csda,Mnt,Rock1,Smarca2,Ncam1
IRF_Q6	24	1,08	9,88E-04	Gata6,Syne2,Ywhag,Col4a1,Eif5a,Npepps,Ccnt2,Mxi1,Cpeb4,Tlk2,Nrp1,Creb1,Meis2,Acsl5,Top1,Slc12a7,Ptk2,Asxl1,Hipk1,Pura,Tcf12,Rrbp1,Psip1,Chgb
MEF2_03	19	0,85	9,91E-04	Ncor1,Cdk8,Foxp1,Tpp2,Rhob,Crtap,Hivep1,Elavl4,Cpeb4,Kpna3,Creb1,Nbea,Atp2a3,Smarca2,Fbxw11,Rasgrf1,Ap3m1,Nfat5,Pura
MEIS1AHOXA9_01	12	0,54	1,05E-03	Gcnt2,Nap1l5,Elavl4,Nrp1,Cnot2,Kif1b,Dicer1,Ncald,Pik3r1,Jarid2,Asxl1,Satb1
HOXA4_Q2	20	0,90	1,06E-03	Syne2,Elk3,Nek7,Zhx1,Eif5a,M6pr,Actr3,Cpeb4,Arid1a,Cs,Klf5,Syncrip,Nrp1,Eif4g2,Dicer1,Pik3r1,Cap1,Atp2a3,Dusp6,Pak3
FXR_Q3	11	0,49	1,27E-03	Gnb2,Ocln,Itpr1,Ubl3,Kif3b,Meis2,Fbxo9,Vamp2,Efemp2,Satb1,Macf1
DR1_Q3	18	0,81	1,27E-03	Fam20c,Calm1,Mcart1,Mospd2,Ckap4,Golga4,Psmf1,Hspd1,Tlk2,Lamp2,Hibadh,Dnmt3a,Dctn2,Gbf1,Nfe2l2,Asxl1,Satb1,Rbbp6
LMO2COM_02	19	0,85	1,27E-03	Gata6,Fkbp2,Nr3c1,Lifr,Elf1,Elavl4,Cpeb4,Sp3,Myo18a,Adcy6,Tnpo3,Hint1,Rbpms,Sfrp5,Map4k3,Pip5k1b,Gspt2,Ctcf,Mbnl1
NFKAPPAB65_01	19	0,85	1,27E-03	Dap3,Ywhaz,Clock,Sin3a,Eif5a,Mapk6,Baz2b,Gng4,Hivep1,Sec63,Ube2d3,Pcsk2,Blcap,Atp1b1,Creb1,Rps19,Slc12a2,Ilk,Nfat5
GRE_C	12	0,54	1,40E-03	Rab10,Mapk6,Rab11a,Elavl4,Sf3b1,Pik3r1,Dcx,Mcm7,Egr1,Tcf12,Slc7a2,Ctgf
COUP_DR1_Q6	18	0,81	1,44E-03	Rreb1,Eif4ebp2,Foxa3,Golga4,Psmf1,Hspd1,Pax6,Ttr,Lamp2,Hibadh,Ube2d3,Ahcyl1,Dctn2,Glg1,Gbf1,Asxl1,Rrbp1,Rbbp6
RREB1_01	17	0,76	1,51E-03	Calm1,Nkx2-2,Gnb2,Kpnb1,Sp4,Rere,Mxi1,Mllt10,Elavl4,Rad23b,Timp3,Hibadh,Foxa2,Ahcyl1,Gspt2,Bcl9,Pura

Transcription factor	*Gene Count*	*Percentage*	*p-Value*	*Genes induced in RasGrf1 KO pancreatic islets*
RSRFC4_01	19	0,85	1,74E-03	Pank1,Itgb3bp,Foxp1,Tpp2,Crtap,Elavl4,Cfl2,Tob2,Wsb2,Cpeb4,Kpna3,Dnaja4,Cul3,Mrps23,Atp2a3,Smarca2,Fbxw11,Rasgrf1,Nfat5
SMAD3_Q6	18	0,81	1,75E-03	Acvr1,Foxp1,Dock9,Fxr1,Arid1a,Baz2a,Ndufs2,Mtmr4,Slc4a2,Dctn2,Csda,Apbb2,Top1,Jarid2,Pak3,Tgfb2,Zranb1,Pura
TST1_01	19	0,85	1,84E-03	Gata6,Smad4,Baz2b,Elavl4,Cfl2,Mll5,Pax6,Klf5,Nipa2,Rnf14,Pgrmc1,P4ha1,Nipsnap3b,Jarid2,Gdi1,Atrx,Dcx,Smarca2,Pura
HNF6_Q6	18	0,81	1,85E-03	Mtmr6,Rere,Herc4,Tob2,Ttr,Ccnd2,Esm1,Wasl,Pik3r1,Sst,Slc12a2,Jarid2,Hbp1,Srpk2,Mbnl1,Abi1,Vldlr,Chchd7
CDP_02	11	0,49	1,88E-03	Tcf4,Cpeb4,Marcks,Cs,Ccnd2,Kif1b,Srebf2,Jarid2,Dcx,Srpk2,Mbnl1
STAT1_01	8	0,36	1,90E-03	Rab10,Set,Arpc1a,Parn,Tnpo3,Brd2,Grinl1a,Asxl1
AML1_01	18	0,81	2,09E-03	Col4a1,Prickle1,Atp6v0b,Tbk1,Diablo,Scn8a,Hdgf,Pax6,Ank3,Gtf2a1,Ubl3,Kif1b,Meis2,Acsl5,Cap1,Atp2a2,Hipk1,Tcf12
AML1_Q6	18	0,81	2,09E-03	Col4a1,Prickle1,Atp6v0b,Tbk1,Diablo,Scn8a,Hdgf,Pax6,Ank3,Gtf2a1,Ubl3,Kif1b,Meis2,Acsl5,Cap1,Atp2a2,Hipk1,Tcf12
OCT1_01	19	0,85	2,18E-03	Etv1,Lpl,Foxp1,Nkx2-2,Ube2s,Rhob,Tcf4,Cryzl1,Pax6,Ank3,Irak1,Srebf2,Ing1,Dusp6,Pten,Jarid2,Asxl1,Hist3h2a,Tcf12
PPAR_DR1_Q2	18	0,81	2,21E-03	Sec24c,Lpl,Gch1,Golga4,Pax6,Lamp2,Hibadh,Dnmt3a,Dctn2,Gbf1,Nfe2l2,Asxl1,Hhex,Satb1,Rock2,Rrbp1,Rbbp6,Mmd
PAX5_01	13	0,58	2,21E-03	Ncoa6,Creb3l2,Cald1,Rab11a,Prpf4b,Arid1a,Timp3,Polr1d,Ube2d3,Eif4g2,Kcnip1,Mnt,Asxl1
PAX6_01	10	0,45	2,26E-03	Etv1,Itgb3bp,Tcf4,Usp9x,Serpini1,Ube2n,Jarid2,Sumo1,Srpk2,Mbnl1
MMEF2_Q6	20	0,90	2,35E-03	Csnk1e,Sin3a,Clasp1,Rhoa,Tcta,Fxr1,Flt1,Crtap,Mxi1,Elavl4,Cfl2,Rcor1,Kpna3,Junb,Hibadh,Dnaja4,Asph,Foxa2,Topbp1,Cbfa2t2
TGIF_01	17	0,76	2,35E-03	Dap3,Acvr1,Nr3c1,Tgfbr1,Ppp3ca,Traf3,Mxi1,Tlk2,Rnf14,Aebp2,Map1lc3b,Itch,Rbx1,Serpini1,Gphn,Slc12a2,Ctcf
GATA_C	28	1,26	2,41E-03	Gata6,Etv1,Acvr1,Cldn7,Lifr,Elf1,Itpr1,Ptprr,Elavl4,Gabbr1,Cs,Myo18a,Aldoa,Eno2,Hmgcs1,Rbpms,Tpm3,Meis2,Atp2a3,Dusp6,Robo1,Ctnnd1,Bcl9,Dcx,Fbxw11,Satb1,Mbd1,Chchd7
ER_Q6_02	19	0,85	2,42E-03	Fam20c,Nefh,Elk3,Mafb,Myadm,Mll5,Pax6,Mapre1,Hibadh,Glud1,Elavl1,Btg2,Fhl1,Dctn2,Mnt,Smpd1,Hipk2,Dcx,Satb1
GATA1_03	18	0,81	2,46E-03	Gata6,Etv1,Itgb3bp,Foxp1,Zdhhc2,Nf2,Ap2m1,Rab11a,Elavl4,Gabbr1,Eno2,Mgea5,Sfrp5,Meis2,Ctcf,Egln1,Prkci,Mbnl1
NF1_Q6_01	19	0,85	2,70E-03	Acvr1,Nav2,Mll5,Tlk2,Stat5b,Ube2d3,Eif4g2,Atp6v1a,Ndufs2,Ncald,Mtss1,Rnf41,Cnot7,Bcl9,Klhl2,Hipk1,Hbp1,Mbnl1,Cbfa2t2
LXR_Q3	8	0,36	2,75E-03	Fkbp2,Mafb,Adam17,Ccni,Tmpo,Jarid2,Ctcf,Rb1cc1
OCT1_05	18	0,81	2,93E-03	Elk3,Lpl,Rhob,Prom1,Ywhab,Pax6,Foxk2,Ing1,Itch,Topbp1,Dusp6,Pten,Ctnnd1,Hist3h2a,Atp2a2,Pura,Tcf12,Lpp
COREBINDINGFACTOR_Q6	19	0,85	3,00E-03	Col4a1,Etf1,Eif5a,Taz,Tbk1,Pafah1b1,Diablo,Hhip,Add1,Pax6,Ank3,Calr,Ubl3,Srebf2,Pitpnc1,Meis2,Enpp2,Atp2a2,Armcx1
LBP1_Q6	16	0,72	3,00E-03	Prickle1,Dock9,Cdkn1b,Elp4,Arid1a,Ccnd2,Baz2a,Ythdf2,Trim8,Eif4g2,Kif1b,Nbea,Vamp3,P2rx4,Zranb1,Ppm1a
AFP1_Q6	18	0,81	3,08E-03	Cpn1,Stx7,Bace2,Foxp1,Nkx2-2,Sp4,Cald1,Agl,Vapb,Pcsk2,Cnot2,Pum2,Mtmr4,Meis2,Dusp6,Jarid2,Dcx,Mbd1
NFKAPPAB_01	18	0,81	3,25E-03	Arpc2,Dap3,Sin3a,Eif5a,Taz,Gng4,Hivep1,Ube2d3,Hcfc1,Gnb1,Pcsk2,Atp1b1,Creb1,Gphn,Slc12a2,Jarid2,Nfat5,Ctgf
ZIC1_01	18	0,81	3,25E-03	Gata6,Ywhag,Nkx2-2,Eif5a,Thrap3,Cdkn1b,Taz,Rab11a,Tlk2,Cpeb3,Zmynd11,Aebp2,Slc4a2,Wdr13,Ctnnd1,Efemp2,Mbnl1,Dhx30
PAX8_B	10	0,45	3,29E-03	Cald1,Hivep1,Ahcyl1,Pum2,Iars,Tia1,Mnt,Srpk2,Pura,Ppm1a
CART1_01	16	0,72	3,36E-03	Bace2,Rab2b,Lgals12,Traf3,Cald1,Cfl2,Elp4,Tceb3,Ttr,Pvrl3,Meis2,Rps19,Pak3,Smarca2,Cldn4,Hipk3

Transcription factor	*Gene Count*	*Percentage*	*p-Value*	*Genes induced in RasGrf1 KO pancreatic islets*
EVI1_02	11	0,49	3,76E-03	Arpc2,Gad1,Rasd1,Abcb7,Gcg,Syncrip,Kcnmb2,Nrp1,Hipk1,Srpk2,Chchd7
EVI1_05	14	0,63	3,80E-03	Bace2,Sufu,Dnajc1,Cacna1d,Elavl4,Nrp1,Mef2a,Meis2,Hhex,Hipk1,Srpk2,Mbnl1,Zranb1,Chchd7
S8_01	18	0,81	3,82E-03	Rreb1,Etv1,Foxp1,Fosb,Gcnt2,Ppap2b,Cald1,Cyfip2,Mllt10,Cs,Nrp1,Myst4,Rnf11,Mapk8,Ctnnd1,Mbnl1,Pura,Ogt
EGR3_01	8	0,36	3,88E-03	Hnrpdl,Klf5,Ythdf3,Gltscr2,Sec14l1,Egr1,Pdcd6ip,Rbbp6
MEIS1BHOXA9_01	12	0,54	4,05E-03	Insm1,Itgb3bp,Rplp0,Tbl1x,Rab10,Nrp1,Pcsk2,Pik3r1,Gbf1,Rasgrf1,Hipk1,Mbnl1
HNF4_01	18	0,81	4,25E-03	Sec24c,Lpl,Gch1,Ube4a,Nkx2-2,Creb3l2,Rhob,Ptbp2,Golga4,Lman2,Hspd1,Tlk2,Baz2a,Creb1,Gbf1,Calb1,Asxl1,Satb1
AR_03	7	0,31	4,46E-03	Khdrbs1,Rere,Elavl4,Rcn1,Cpeb4,Syncrip,Adcy6
RFX1_02	17	0,76	4,65E-03	Rreb1,Strn4,Itgb3bp,Gnb2,Tm9sf1,Itpr1,Cryzl1,Phtf1,Ptprr,Phc1,Dhx40,Ttr,Ncald,Osbpl2,Stx18,Pkig,Pum1
FOXJ2_02	16	0,72	4,78E-03	Lifr,Foxp1,Tcf4,Pafah1b1,Trim2,Mxi1,Ap2m1,Ppm1b,Ttc3,Pax6,Pvrl3,Ipo4,Atp2a3,Satb1,Dnajb9,Pura
POU6F1_01	17	0,76	5,19E-03	Mcart1,Etv1,Polr3c,Foxp1,Rere,Traf3,Cyfip2,Cfl2,Gtpbp1,Marcks,Dusp1,Cbx6,Ipo4,Meis2,Mbnl1,Ncam1,Chchd7
OCT1_06	18	0,81	5,23E-03	Syne2,Prkrir,Foxp1,Cacna1d,Elp4,Rcor1,Ank3,Atp1b1,Mnt,Dhrs4,Kctd5,Vamp2,Satb1,Hipk1,Srpk2,Mbnl1,Zranb1,Cbfa2t2
AML_Q6	24	1,08	5,41E-03	Lifr,Etf1,Kpnb1,Tulp4,Mtmr3,Eif5a,Tbl1x,Ccnt2,Flt1,Pafah1b1,Cdc37l1,Hhip,Add1,Dusp1,Gtf2a1,Calr,Srebf2,Pitpnc1,Mtmr4,Pik3r1,Atp2a2,Tcf12,Rbbp6,Armcx1
AR_01	11	0,49	6,03E-03	Psma6,Pank1,Lifr,Foxa3,Rab10,Dstn,Itpkb,Sf3b1,Got2,Amph,Appbp2
AP4_Q6_01	17	0,76	6,42E-03	Etv1,Acvr1,Ywhaz,Ppp3ca,Nosip,Cdkn1b,Taz,Ccnd2,Myo18a,Ythdf2,Ube2d3,Trim8,Brd4,Bach1,Spg21,Zranb1,Psip1
TAL1BETAE47_01	16	0,72	6,70E-03	Foxp1,Ddit3,Ppap2b,Hectd1,Riok3,Elavl4,Scgn,Arpc1a,Mll5,Dars,Brd4,Trim37,Pik3r1,Dapk1,Dcx,Tcf12
HNF4_Q6	17	0,76	7,50E-03	Etv1,Dap3,Fkbp2,Creb3l2,Nav2,Traf3,Gtpbp1,Numb,Tlk2,Xrcc5,Mtmr4,Ipo4,Meis2,Ush1c,Rab3d,Pura,Ctgf
OCT1_Q6	17	0,76	7,89E-03	Etv1,Lpl,Tcf4,Traf3,Prom1,Pax6,Kpna3,Ank3,Jund,Itch,Rps19,Vamp3,Dusp6,Pten,Hist3h2a,Atp2a2,Cbfa2t2
PR_02	11	0,49	8,11E-03	Khdrbs1,Rere,Dstn,Cpeb4,Rwdd1,Arhgap6,Syncrip,Adcy6,Fbxo9,Ets1,Dlg3
OCT1_03	15	0,67	8,68E-03	Elk3,Nek7,Lifr,Rplp0,Tcf4,Tbl1x,Pias1,Ube2d3,Nrp1,Poldip3,Meis2,Rnf11,Mbnl1,Rit1,Chchd7
E47_02	16	0,72	8,72E-03	Syne2,Trim23,Armcx2,Mll5,Baz2a,Myo18a,Hdac3,Aig1,Amph,Strn3,Meis2,Calb1,Pcm1,Bcl9,Egln1,Pura
OCT1_07	12	0,54	8,76E-03	Etv1,Bace2,Traf3,Cryzl1,Elavl4,Atm,Cpeb4,Elp4,Irak1,Kif3b,Gdi1,Asxl1
ELK1_01	16	0,72	9,15E-03	Elk3,Lifr,Cnot6l,Herc4,Zcchc7,Itpkb,Cpeb4,Pax6,Sp3,Ube2d3,Slc39a6,Mgea5,Prkag2,Pak3,Dnajb9,Rac1
AR_Q2	10	0,45	1,05E-02	Rreb1,Rere,Rab11a,Hivep1,Cpeb4,Rwdd1,Syncrip,Ythdf3,Trip11,Appbp2
AP4_01	16	0,72	1,31E-02	Pja2,Pcbp2,Foxp1,Ddit3,Sin3a,Prickle1,Mtmr3,Map3k3,Atp8a1,Baz2a,Ctdspl,Ptk2,Trpm7,Epn2,Nrd1,Cbfa2t2
NKX25_01	10	0,45	1,61E-02	Nkx2-2,Tcf4,Cfl2,Pax6,Klf5,Irak1,Slc39a6,Ctcf,Bcl9,Egln2
AMEF2_Q6	16	0,72	1,73E-02	Pabpc4,Etv1,Bace2,Clasp1,Flt1,Crtap,Kpna3,Gtf2a1,Jund,Usp47,Ctbp2,Birc2,Fbxw11,Nfat5,Pura,Rbbp6

Transcription factor	*Gene Count*	*Percentage*	*p-Value*	*Genes induced in RasGrf1 KO pancreatic islets*
HEN1_01	13	0,58	1,83E-02	Pabpc1,Foxp1,Sin3a,Prickle1,Map3k3,Ythdf2,Ube2d3,Cbx6,Tpm3,Rock1,Dcx,Pura,Tcf12
OCT_Q6	16	0,72	1,89E-02	Etv1,Lpl,Foxp1,Ube2s,Rhob,Traf3,Cryzl1,Pax6,Jund,Srebf2,Top1,Dusp6,Pten,Atp2a2,Tcf12,Cbfa2t2
OCT1_Q5_01	16	0,72	2,16E-02	Etv1,Lpl,Foxp1,Ube2s,Rhob,Traf3,Cryzl1,Pax6,Jund,Srebf2,Ppp2r5c,Dusp6,Pten,Atp2a2,Tcf12,Cbfa2t2
IRF7_01	16	0,72	2,25E-02	Pabpc4,Gata6,Ppp2r2a,Cald1,Hivep1,Tlk2,Kpna3,Ythdf2,Nrp1,Dicer1,Tia1,Sst,Gna13,Slc12a7,Gdi1,Tcf12
SEF1_C	2	0,09	2,28E-02	Atf2,Meis2
HMX1_01	5	0,22	2,32E-02	Trrap,Nkx2-2,Junb,Arid4b,Amph
AR_02	4	0,18	2,76E-02	Rwdd1,Adcy6,Got2,Chchd7
HTF_01	6	0,27	2,76E-02	Armcx2,Nkx2-2,Elf1,Sec61a1,Gbf1,Ubqln1
E2A_Q2	14	0,63	2,87E-02	Acvr1,Armcx2,Golga4,Elavl4,Tlk2,Cpeb3,Eif4g2,Rapgef4,Apbb2,Epn2,Vdr,Atp2a2,Psip1,Macf1
COMP1_01	9	0,40	2,89E-02	Cald1,Numb,Gtf2a1,Meis2,Dcx,Serpinh1,Mbnl1,Tcf12,Ncam1
CDP_01	7	0,31	2,91E-02	Rreb1,Nkx2-2,Rere,Herc4,Ttr,Foxa2,Egr1
PPARA_02	9	0,40	3,07E-02	Creb3l2,Kpnb1,Kcnb1,Mafb,Rad23b,Cbx6,Mnt,Dnajc7,Pura
HEB_Q6	15	0,67	3,23E-02	Colec12,Acvr1,Nosip,Abhd4,Ccnd2,Baz2a,Myo18a,Ythdf2,Ube2d3,Trim8,Brd4,Tgfb2,Zranb1,Macf1,Cbfa2t2
FOXJ2_01	12	0,54	3,49E-02	E2f5,Rab3ip,Cpeb4,Scg3,Pik3c2a,Hnf4a,Ctnnal1,Scamp1,Atp2a2,Tgfb2,Ncam1,Chchd7
FXR_IR1_Q6	8	0,36	3,57E-02	Insm1,Cldn7,Nono,Rnf14,Kctd5,Vamp2,Eif5,Macf1
E2F_01	6	0,27	3,82E-02	Gata6,Casp2,Ptbp2,Elavl4,Dnmt1,Topbp1
GATA3_01	14	0,63	3,82E-02	Fkbp2,Spred2,Elf1,Rabgap1,Elavl4,Pias1,Klf5,Myo18a,Syncrip,Mecp2,Rbpms,Meis2,Glg1,Mbnl1
FREAC4_01	10	0,45	3,82E-02	Colec12,Sin3a,Kbtbd2,Cald1,Dusp1,Pnrc1,Ulk1,Pum2,Ctcf,Pura
TCF4_Q5	14	0,63	4,10E-02	Prkrir,Smad1,Elk3,Pank1,Lifr,Nav2,Rere,Phc1,Scg3,Arid1a,Adam9,Ppp2r5c,Pcm1,Cbfa2t2
MYOGENIN_Q6	14	0,63	4,10E-02	Elk3,Dock9,Tjp1,Atp5b,Arid1a,Rplp1,Ptprj,Trim8,Ttc13,Eif4g2,Atp1b1,Epn2,Vamp2,Tnfaip1
CRX_Q4	15	0,67	4,69E-02	Lifr,Clasp1,Cpeb4,Pax6,Stat5b,Esm1,Tkt,Sst,Ctbp2,Jarid2,Atp1b3,Cnot4,Satb1,Srpk2,Pura
EVI1_06	3	0,13	5,44E-02	Arpc2,Tcf4,Rnf11
